# The Human 2-Cys Peroxiredoxins form Widespread, Cysteine-Dependent- and Isoform-Specific Protein-Protein Interactions

**DOI:** 10.3390/antiox10040627

**Published:** 2021-04-20

**Authors:** Loes van Dam, Marc Pagès-Gallego, Paulien E. Polderman, Robert M. van Es, Boudewijn M. T. Burgering, Harmjan R. Vos, Tobias B. Dansen

**Affiliations:** 1 Center for Molecular Medicine, Molecular Cancer Research, University Medical Center Utrecht, Universiteitsweg 100, 3584 CG Utrecht, The Netherlands; l.vandam-2@umcutrecht.nl (L.v.D.); m.pagesgallego@umcutrecht.nl (M.P.-G.); p.e.polderman@umcutrecht.nl (P.E.P.); r.m.vanes-4@umcutrecht.nl (R.M.v.E.); b.m.t.burgering@umcutrecht.nl (B.M.T.B.); h.r.vos-3@umcutrecht.nl (H.R.V.); 2 Oncode Institute, University Medical Center Utrecht, Universiteitsweg 100, 3584 CG Utrecht, The Netherlands

**Keywords:** redox proteomics, S-peroxiredoxinylation, peroxiredoxin, redox signaling, redox relay, hydrogen peroxide, protein thiol oxidation, cysteine sulfenic acid, thiol disulfide exchange

## Abstract

Redox signaling is controlled by the reversible oxidation of cysteine thiols, a post-translational modification triggered by H_2_O_2_ acting as a second messenger. However, H_2_O_2_ actually reacts poorly with most cysteine thiols and it is not clear how H_2_O_2_ discriminates between cysteines to trigger appropriate signaling cascades in the presence of dedicated H_2_O_2_ scavengers like peroxiredoxins (PRDXs). It was recently suggested that peroxiredoxins act as peroxidases and facilitate H_2_O_2_-dependent oxidation of redox-regulated proteins via disulfide exchange reactions. It is unknown how the peroxiredoxin-based relay model achieves the selective substrate targeting required for adequate cellular signaling. Using a systematic mass-spectrometry-based approach to identify cysteine-dependent interactors of the five human 2-Cys peroxiredoxins, we show that all five human 2-Cys peroxiredoxins can form disulfide-dependent heterodimers with a large set of proteins. Each isoform displays a preference for a subset of disulfide-dependent binding partners, and we explore isoform-specific properties that might underlie this preference. We provide evidence that peroxiredoxin-based redox relays can proceed via two distinct molecular mechanisms. Altogether, our results support the theory that peroxiredoxins could play a role in providing not only reactivity but also selectivity in the transduction of peroxide signals to generate complex cellular signaling responses.

## 1. Introduction

In order to adapt to a changing environment, cells continuously translate extracellular cues into appropriate cellular responses through cascades of protein-protein interactions and post-translational modifications known as signal transduction. A recently discovered form of signal transduction, termed redox signaling, uses hydrogen peroxide (H_2_O_2_) as a second messenger, and proceeds through the reversible oxidation of specific cysteine thiols in proteins (for a review, see ref. [1]). To function as a reliable second messenger, H_2_O_2_ should be able to discriminate which cysteines it needs to oxidize specifically in order to trigger the proper signaling cascade. Although numerous H_2_O_2_-regulated proteins and processes have been discovered, it is unclear how exactly redox signaling achieves the required reactivity and specificity, which are fundamental requirements for coherent cellular signaling.

H_2_O_2_ is considered the major reactive oxygen species (ROS) for signaling because of its relative stability compared to other cellular reactive oxygen species (ROS, i.e., O_2_^•−^ and ^•^OH) [2]. However, this relative stability also means that H_2_O_2_ reacts poorly with most cysteine thiols, with rate constants ranging from 20 to 200 M^−1^s^−1^ [3,4,5]. Additionally, dedicated H_2_O_2_ scavengers like peroxiredoxins (PRDXs) are estimated to eliminate >99% of cellular H_2_O_2_ [6], because their catalytic cysteines react with many orders of magnitude faster with H_2_O_2_ than other thiols in cysteine side chains in proteins, including those found to be redox regulated. Peroxiredoxins are highly abundant and ubiquitous proteins, with isoforms localized to cytoplasm, mitochondria, endoplasmic reticulum (ER) and other cellular compartments [7]. The poor reactivity of thiols with H_2_O_2_ combined with the effective elimination of H_2_O_2_ by peroxiredoxins seems to challenge the idea that reactivity and selectivity in redox signaling can be achieved by a simple molecule like H_2_O_2_.

Peroxiredoxins do not only scavenge H_2_O_2_; in fact, oxidized 2-Cys peroxiredoxins have also been shown to act as peroxidases and facilitate H_2_O_2_-dependent protein oxidation via disulfide exchange reactions. For example, in *Saccharomyces cerevisiae,* Tsa1 and Orp1 peroxidases relay towards the Yap1 transcription factor [8] and a similar mechanism was identified for Sty1 in in *Schizosaccharomyces pombe* [9]. In mammalian cells, the ASK1 kinase and STAT3 transcription factor are oxidized by PRDX1 and PRDX2, respectively [10,11], and ER-localized PRDX4 is known to induce disulfide formation through the oxidation of protein disulfide isomerase (PDI) [12].

**Figure 1:**
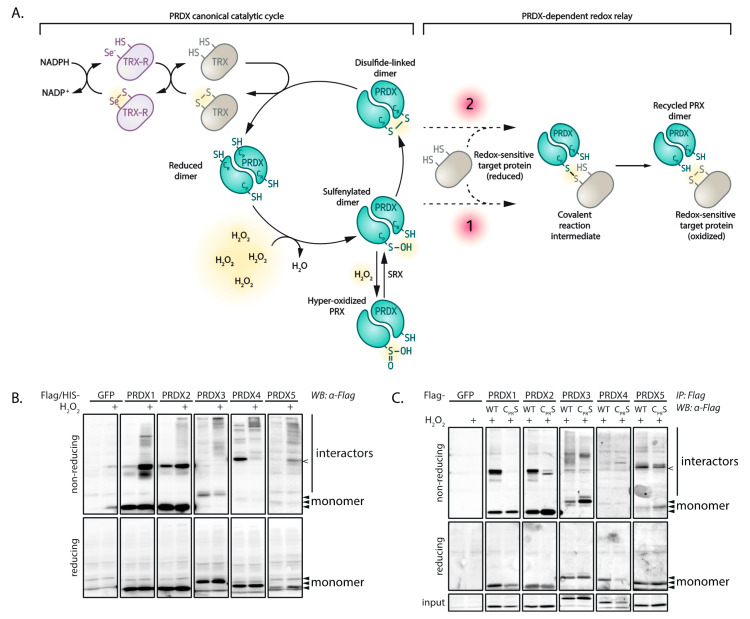
Peroxiredoxins form many H_2_O_2_- and cysteine-dependent interactions. (**A**) Scheme depicting the canonical oxidation/reduction cycle of 2-Cys peroxiredoxins and the possibilities for forming covalent reaction intermediates with target proteins (1 and 2). NADPH; nicotinamide adenine dinucleotide phosphate, TRX; thioredoxin, TRX-R; thioredoxin reductase, PRDX; peroxiredoxin. (**B**) HEK293T cells expressing Flag-tagged peroxiredoxin isoforms were treated for 2 min with H_2_O_2_ and analyzed by immunoblotting. H_2_O_2_ concentrations were 100 µM for PRDX1, 3, 4 and 5, and 25 µM for PRDX2. Non-reducing SDS-PAGE and immunoblotting shows overall H_2_O_2_-induced protein interactions for each peroxiredoxin isoform, reflected by the formation of PRDX-S-S-X conjugates. Immuno-precipitated Flag-peroxiredoxin isoforms also form PRDX-S-S-X conjugates in a cysteine-dependent manner (**C**). All immunoblots shown in this figure are from the same gel and membrane with different exposure for each isoform, representative of multiple experiments (*n* ≥ 3). IP: immunoprecipitation; WB: Western blotting; input: cleared cell lysate as used for immunoprecipitation, reduced sample.

Others have shown a more widespread role for peroxiredoxins in H_2_O_2_-induced thiol oxidation [13,14]. In this so-called peroxiredoxin-based relay model, the extremely reactive peroxidatic cysteine of peroxiredoxins first reacts with H_2_O_2_ and subsequently the oxidized peroxiredoxin catalyzes the oxidation of low reactivity thiols in redox-regulated proteins (see Figure 1A). This mechanism could explain how so many intrinsically unreactive protein thiols can be found to be reversibly oxidized in response to H_2_O_2_-dependent redox signaling, despite the presence of a highly abundant and reactive H_2_O_2_ scavenging system.

Although the peroxiredoxin-based relay model may explain how the reactivity of H_2_O_2_ with protein thiols is overcome, it does not as yet explain how the selectivity in H_2_O_2_-dependent redox signaling is achieved. In order to produce relevant biological signals, selective substrate targeting is required to achieve proper signaling outputs. In redox signaling, this would mean that in the presence of numerous potential substrates, specific subsets of redox-regulated proteins should be oxidized dependent on, for instance, the subcellular localization or the local concentration of H_2_O_2_. Mammalian cells express five 2-Cys peroxiredoxin isoforms, each with their own localization, oxidation kinetics and structural differences around their catalytic sites. We therefore hypothesized that reactivity and selectivity in redox signaling could also be provided by the different 2-Cys peroxiredoxins.

According to this line of reasoning, peroxiredoxins would be expected to participate in temporary covalent complexes with isoform-specific subsets of target proteins, mediated by disulfides that form between their catalytic cysteine and a cysteine in these target proteins (see Figure 1A). To test this hypothesis, we used a systematic mass-spectrometry-based approach to identify cysteine-dependent interactors of the five human 2-Cys peroxiredoxins. Indeed, our results suggest that all five human 2-Cys peroxiredoxins are capable of forming disulfide-dependent heterodimers with a large set of proteins, and that each peroxiredoxin isoform displays a preference for a subset of disulfide-dependent binding partners. We explore what isoform-specific properties underlie these observations and we provide evidence that peroxiredoxin-based redox relays can proceed via two distinct molecular mechanisms. These findings support the idea that peroxiredoxins could play a role in providing not only reactivity but also selectivity in the transduction of peroxide signals to generate complex cellular signaling responses.

## 2. Materials and Methods

### 2.1. Cell Lines and Culture

HEK293T cells (Manassas, VA, USA, ATCC)were cultured in bicarbonate-buffered DMEM (Basel, Switzerland, Lonza, BE12-604Q), supplemented with 10% FBS (Alkmaar, Netherlands, Bodinco BDC-40506-C05), 2 mM L-glutamine (Basel, Switzerland, Lonza, BE17-605E) and 100 U/mL penicillin-streptomycin (Basel, Switzerland, Lonza, DE17-602E) and kept at 37 °C and under a 6% CO_2_ atmosphere. Transfections of HEK293T cells were carried out using PEI (St. Louis, MO, USA, Sigma-Aldrich, P3640) or FugeneHD reagent (Madison, WI, USA, Promega, E2311) following the manufacturer’s instructions. After two days, cells were harvested for further analysis.

### 2.2. Plasmids and Reagents

Human PRDX1-5 with *att* recombination sites were cloned from cDNA using the following primers. PRDX1 (NCBI RefSeq NM_002574)):_Fwd 5′-GGGGACAAGTTTGTACAAAAAAGCAGGCTTAATGTCTTCAGGAAATGCTAAAATTGGGC-3, Rev 5′-GGGGACCACTTTGTACAAGAAAGCTGGGTCCTACTTCTGCTTGGAGAAATATTCTTTGCT-3′. PRDX2 (NCBI RefSeq NM_005809): Fwd 5′-GGGGACAAGTTTGTACAAAAAAGCAGGCTTAATGGCCTCCGGTAACGC-3′, Rev 5′-GGGGACCACTTTGTACAAGAAAGCTGGGTTCTAATTGTGTTTGGAGAAATATTCCTTGCTGT-3′. PRDX3 (NCBI RefSeq NM_006793): _Fwd 5′-GGGGACAAGTTTGTACAAAAAAGCAGGCTTAATGGCGGCTGCTGTAGG-3′, Rev 5′-GGGGACCACTTTGTACAAGAAAGCTGGGTTCTACTGATTTACCTTCTGAAAGTACTCTTTGGAAG-3′. PRDX4 (NCBI RefSeq NM_006406):_Fwd 5′-GGGGACAAGTTTGTACAAAAAAGCAGGCTTAATGGAGGCGCTGCCG-3′, Rev 5′-GGGGACCACTTTGTACAAGAAAGCTGGGTTTTTCAGTTTATCGAAATACTTCAGCTTTCCAG-3′. PRDX5 (NCBI RefSeq NM_0129094): Fwd 5′-GGGGACAAGTTTGTACAAAAAAGCAGGCTTAATGGGACTAGCTGGCGTG-3′, Rev 5′-GGGGACCACTTTGTACAAGAAAGCTGGGTTTCAGAGCTGTGAGATGATATTGGGTG-3′. Using Gateway technology (Invitrogen, now owned by Waltham, MA, USA, Thermo Scientific) entry clones were generated. The peroxidatic and resolving cysteine mutants of PRDX1-5 were created by site-directed mutagenesis PCR using the following primers: PRDX1_C52S_F-5′-CTTTGTGTCCCCCACGGAG-3′, PRDX1_C52S._R-5′-CTCCGTGGGGGACACAAAG-3′, PRDX1_C173S_F-5′-GGGAAGTGTCCCCAGCTGG-3′, PRDX1_C173S._R-5′-CCAGCTGGGGACACTTCCC-3′, PRDX2_C51S_F-5′-TCACTTTTGTGTCTCCCACCGAGATCATCGCG-3′, PRDX2_C51S._R-5′-CGCGATGATCTCGGTGGGAGACACAAAAGTGA-3′, PRDX2_C172S_F-5′-CATGGGGAAGTTTCTCCCGCTGGCT-3′, PRDX2_C172S._R-5′-AGCCAGCGGGAGAAACTTCCCCATG-3′, PRDX3_C108S_F-5′-TCACCTTTGTGTCTCCTACAGAAATTGTTGCT-3′, PRDX3_C108S._R-5′-AGCAACAATTTCTGTAGGAGACACAAAGGTGA-3′, PRDX3_C229S_F-5′-ACACATGGAGAAGTCTCTCCAGCGAACTGGACA-3′, PRDX3_C229S._R-5′-TGTCCAGTTCGCTGGAGAGACTTCTCCATGTGT-3′, PRDX4_C124S_F-5′-ATTTCACATTTGTGTCTCCAACTGAAATTATCGCTTTTGG-3′, PRDX4_C124S._R-5′-CCAAAAGCGATAATTTCAGTTGGAGACACAAATGTGAAAT-3′, PRDX4_C245S_F-5′-GGAGAAGTCTCCCCTGCTGGCTGGAA-3′, PRDX4_C245S._R-5′-TTCCAGCCAGCAGGGGAGACTTCTCC-3′, PRDX5_C47S_F-5′-TTCACCCCTGGATCTTCCAAGACACACCTG-3′, PRDX5_C47S._R-5′-CAGGTGTGTCTTGGAAGATCCAGGGGTGAA-3′, PRDX5_C151S_F-5′-CAGGCCTCACCTCCAGCCTGGCA-3′, PRDX5_C151S._R-5′-TGCCAGGCTGGAGGTGAGGCCTG-3′. Gateway technology (Waltham, MA, USA, Thermo Scientific) was used to create N-terminally tagged FLAG-HIS-PRDX1-3 and 5, and C-terminally tagged PRDX4-FLAG-HIS constructs (backbone pcDNA3). Furthermore, 30% H_2_O_2_ (St. Louis, MO, USA, Sigma-Aldrich 31642) was freshly diluted to a stock of 10 or 100 mM in H_2_O for every experiment. Unless stated otherwise, H_2_O_2_ (St. Louis, MO, USA, Sigma-Aldrich) treatments were 25 μM (PRDX2) and 100 μM (other isoforms) for 2 min. 

### 2.3. Co-Immunoprecipitation Experiments and Western Blotting

After treatment with H_2_O_2_, cells were scraped in 100 mM N-ethylmaleimide (NEM, St. Louis, MO, USA, Sigma-Aldrich E3876) in PBS for 5 min at 37 °C to trap free thiols in their in vivo redox state during sample preparation and collected by centrifugation at 1200 rpm for 3 min. Cells were lysed using a buffer containing 50 mM Tris-HCl pH 7.5, 1% TX100, 1.5 mM MgCl_2_, 5 mM EDTA, 100 mM NaCl, NaF, Leupeptin and Aprotinin (all from St. Louis, MO, USA, Sigma-Aldrich). Furthermore, 100 mM iodoacetamide (St. Louis, MO, USA, Sigma-Aldrich) was added to the lysis buffer to prevent post-lysis cysteine oxidation and to inactivate disulfide reducing enzymes. After centrifugation at 14,000 rpm for 10 min, 5% of the supernatant was kept as a control (denoted ‘input’ in figures) and the remaining supernatant was used for immunoprecipitation with anti-Flag-M2 affinity gel (St. Louis, MO, USA, Sigma-Aldrich A222). After a 2 h incubation, immunoprecipitates were washed 4 times with lysis buffer containing 1 M NaCl and samples were boiled for 5 min in sample buffer with or without the reducing agent β-mercaptoethanol. Samples were separated on a 10% polyacrylamide gel and transferred to immobilon-FL membranes (Burlington, MA, USA, Merck) before staining.

### 2.4. Antibodies

The following antibodies were used in this study: anti-Flag antibody and anti-Flag-M2 beads (St. Louis, MO, USA Sigma-Aldrich F3165 and A222, respectively), monoclonal anti-HA antibody (12CA5) was prepared using hybridoma cell lines, anti-tubulin (Burlington, MA, USA, Merck Millipore CP06), anti-peroxiredoxin-SO_3_ (Cambridge, MA, USA, Abcam ab16830), anti-peroxiredoxin 1 (Cambridge, MA, USA, Abcam ab15571), anti-peroxiredoxin 2 (Cambridge, MA, USA, Abcam ab15572), anti-peroxiredoxin 3 (Cambridge, MA, USA, Abcam ab73349), anti-peroxiredoxin 4 (Cambridge, MA, USA, Abcam ab59542) and anti-peroxiredoxin 5 (Cambridge, MA, USA, Abcam ab16944). Detection of 2 fluorescent secondary antibodies was done simultaneously using the LI-COR Biosciences Odyssey Infrared Imaging System or the Amersham Typhoon NIR Plus Biomolecular Imager (Chicago IL, USA, GE Healthcare), detection of HRP-coupled secondary antibodies was performed using the FUJIFILM Luminescent Image Analyzer LAS-3000.

### 2.5. Mass Spectrometry Sample Preparation

For the identification of cysteine-dependent interactors the lysate of 4 × 20 cm dishes were used for each pulldown on 75 μL of Flag-M2 beads similar to the immunoprecipitation experiments described above. All immunoprecipitations were performed using three biological replicates. After washing, beads were resuspended with 8 M urea in 1 M ammonium bicarbonate (ABC), reduced and alkylated in 10 mM TCEP and 40 mM chloroacetamide (CAA) for 30 min at RT. After fourfold dilution with 1 M ABC, proteins were digested overnight on-bead with 250 ng Trypsin/LysC (Madison, WI, USA, Promega V5071) per sample at 37 °C. Samples were cleaned up with in-house-made C18 stagetips.

### 2.6. Mass Spectrometry

Mass spectrometry was performed as previously described [15]. Peptides were separated on a 30-cm pico-tip column (75 μm ID, New Objective) and were packed in-house with 3 μm aquapur gold C-18 material (Dr. Maisch) using a 140-min gradient (7–80% ACN 0.1% FA), delivered by an easy-nLC 1000 (LC 120, Waltham, MA, USA, Thermo Scientific), and electro-sprayed directly into an Orbitrap Fusion Tribrid Mass Spectrometer (LC 120, Waltham, MA, USA, Thermo Scientific). Raw files were analyzed with MaxQuant software version 1.5.2.8 with oxidation of methionine, alkylation with N-ethylmaleimide and carbamidomethylation set as variable modifications. The human protein database of UniProt was searched with both the peptide as well as the protein false discovery rate set to 1%. The mass spectrometry proteomics data were uploaded into the ProteomeXchange Consortium via the PRIDE [16] partner repository with the dataset identifier PXD024114. Downstream analysis was done using R version 4.0.2.

### 2.7. Data Filtering and proDA Modeling

The code used was uploaded to GitHub at https://github.com/loesoe/peroxiredoxin (accessed on 2 July 2020). In short, LFQ data from the MaxQuant proteinGroups file and corresponding protein information was used. Proteins were filtered for reverse hits and standard contaminants. Next, we selected proteins that were identified with three or more unique peptides and were measured in at least one sample in two or more replicates. Data was log_2_-transformed and normalized using quantile normalization to simultaneously correct for overall protein content and immunoprecipitation (IP) efficiency. ProDA model fitting was performed using the number of proteins in the data as the number of subsamples. To test for differential protein abundance, a proDa model was fit to compare WT against mutant for each peroxiredoxin.

### 2.8. Threshold Cutoff Calculation

To determine the cutoff for out data, we fitted a proDA model for wild-type and mutant peroxiredoxin, without considering the isoform. Next, we repeated this 100 times, but instead with randomized labels. The difference at which 5% of the randomized data was included was determined as the cutoff.

### 2.9. Known Redox-Sensitive Proteins

A reference proteome containing 75,071 human entries from Uniprot tagged with the keyword reference proteome was downloaded from https://www.uniprot.org/help/reference_proteome (accessed on 2 August 2020). Proteins that were previously identified in a screen for redox-sensitive proteins in human cell lines (HEK293T and HCT116) were taken from [17].

### 2.10. Upset Plot and Venn Diagrams

To visualize the isoform-specific set of intersections we used the UpsetR package [18]. Venn diagrams were created using the Venn version 1.9 package and colored manually.

### 2.11. Localization Analysis

We analyzed the localization of isoform-specific interactors using the neighborhood/compartment predictions data for A431 cells from https://www.subcellbarcode.org (accessed on 2 January 2020) [19]. Peroxiredoxin interactors with the highest fold change (>10-fold) were matched with their neighborhood data and their fold enrichment were calculated compared to the cell line data. Main localization of peroxiredoxin isoforms from this tool are as follows: PRDX1 PRDX2 and PRDX5, cytosol; PRDX3, mitochondria; PRDX4, unclassified.

### 2.12. Sequence Similarity

PRDX Sequences were loaded as a FASTA file. Pairwise alignment was calculated using EMBOSS needle (https://www.ebi.ac.uk/Tools/psa/emboss_needl, accessed on 2 July 2016), which uses the Needleman–Wunsch global alignment algorithm to find the optimum global alignment. Similarity (the percentage of matches between the two aligned sequences) was plotted for each peroxiredoxin isoform pair.

### 2.13. AA Composition and Motifs

The sequences for PRDX isoform-specific interactors were retrieved from Uniprot (https://www.uniprot.org/uploadlists, accessed on 2 January 2021). Sequences were loaded using the Biostrings package, sequences were shuffled 100 times as a background using the universalmotif package. Motifs were extracted of 9 amino acids centered around each cysteine. The amino acid composition was calculated for the isoform-specific sequences, the shuffled background and total protein using the alphabetFrequency function. Fold enrichment and Benjamini–Hochberg adjusted *p*-values were calculated per peroxiredoxin isoform using the amino acid composition of all other isoforms as a control. Motifs were analyzed using motif-x (rmotifx package) using the shuffled sequences as a background [20].

## 3. Results

### 3.1. All Five Human 2-Cys Peroxiredoxins Have Many H_2_O_2_- and Cysteine-Dependent Interactors

As described above, peroxiredoxin-catalyzed cysteine oxidation proceeds through the (transient) formation of a disulfide bond between peroxiredoxins and target proteins, as has been shown for PRDX1 and PRDX2 [13]. If all human 2-Cys peroxiredoxins are involved in redox relay signaling, disulfide-dependent heterodimers (i.e., PRDX-S-S-X) would be expected to be formed upon oxidation of PRDX1-5. These disulfide-dependent heterodimers would show up as peroxiredoxin-containing high-molecular weight bands upon separation on non-reducing SDS-PAGE followed by Western blotting. Indeed, a number of high-molecular weight bands containing PRDX1-5 can be detected upon a 2-min pulse of H_2_O_2_ (Figure 1B). These Flag-PRDX1-5-containing complexes are indeed sensitive to reduction, confirming the presence of disulfides. For PRDX1, 3, 4 and 5 we used 100 µM H_2_O_2_, since that concentration showed many interaction partners in another study investigating peroxiredoxin binding partners in this cell type [13]. PRDX2 shows substantial hyperoxidation at 100 µM H_2_O_2_ and we therefore used 25 µM H_2_O_2_ for the experiments using PRDX2 (Figure S1).

The covalent, disulfide-dependent heterodimeric complexes of Flag-PRDX1-5 and their interaction partners could be isolated by immunoprecipitation (Figure 1C). Mutation of the catalytic cysteines (C_P_ and C_R_) to serine (PRDX C_PR_S) abolished the formation of the majority of H_2_O_2_-induced disulfide-dependent binding partners for all peroxiredoxin isoforms (Figure 1C), indicating that the catalytic cysteines in all 2-Cys peroxiredoxins form disulfide-dependent complexes with several other proteins upon oxidation. The PRDX1–5 containing complexes migrate different distances than the disulfide dependent homodimers (for PRDX 1–4). Note that for PRDX4 and PRDX5 a band runs at about twice their MW (Figure 1B), which is also present in the C_PR_S mutant; hence this cannot be the oxidized peroxiredoxin homodimer. For PRDX5 this is not unexpected since this is the only a-typical 2-Cys peroxiredoxin and forms an intramolecular rather than intermolecular disulfide upon oxidation by H_2_O_2_. In summary, these results show that the five 2-Cys peroxiredoxin isoforms form many H_2_O_2_-induced, disulfide-dependent interactions that can be isolated by immunoprecipitation.

### 3.2. A Proteome-Wide Screen Identifies the Interactome of Human 2-Cys Peroxiredoxins

**Figure 2:**
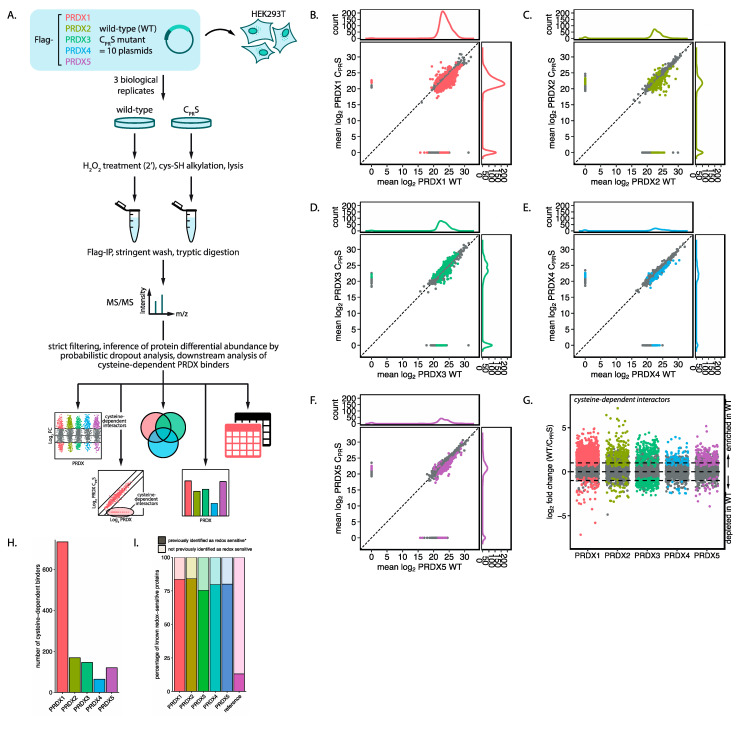
A proteome-wide screen to identify the interactome of human 2-Cys peroxiredoxins. (**A**) Schematic representation of our workflow. Cells expressing Flag-PRDX isoforms (either wild-type or mutant) were treated with H_2_O_2_ and alkylated with NEM and IA prior to and during lysis, respectively. H_2_O_2_ concentrations were 100 µM for PRDX1, 3, 4 and 5 and 25 µM for PRDX2. Flag-peroxiredoxins and their interacting proteins were pulled down using immobilized anti-Flag-M2 and subsequently exposed to a stringent high-salt wash and binding partners were measured by mass spectrometry. After stringent filtering we inferred protein differential abundance by probabilistic dropout modeling. PRDX; peroxiredoxin, WT; wild-type, C_P_; peroxidatic cysteine; C_R_; resolving cysteine, IP; immunoprecipitation, MS/MS; tandem mass spectrometry. (**B–F**) Marginal scatter plots for peroxiredoxins 1–5, depicting the mean log_2_ LFQ intensity for both wild-type and C_PR_S mutant peroxiredoxin. Colored proteins are identified with a *p*-value <0.05, and the distribution of these proteins are also visualized in the marginal density plots. Proteins interacting with both WT and mutant peroxiredoxin appear on a diagonal, while proteins that bind only WT or the C_PR_S mutant appear along the horizontal or vertical axis, respectively, and can be found at the edges of the marginal plots. (**G**) Scatter plot of log_2_ fold change between wild-type and C_PR_S-peroxiredoxin isoforms. Horizontal lines are positioned at log_2_ fold change of 1 and –1 (i.e., a 2-fold change). Colored proteins are identified with a *p*-value <0.05. (**H**) Number of cysteine-dependent interaction partners per peroxiredoxin isoform identified with *p*-value <0.05 and log_2_ fold change >1 in our screen. (**I**) Bar chart representing the percentage of interactors per peroxiredoxin that are previously identified as redox-sensitive in a large-scale mass-spectrometry-based screen. The reference proteome contains 75071 human UniProt entries with “reference proteome” as keyword.

Having confirmed the ability of all five 2-Cys peroxiredoxins to form intermolecular disulfide-dependent complexes, we wondered about the scale of the interactome and the identities of the disulfide-dependent interacting proteins. To answer these questions, we performed an unbiased, quantitative mass-spectrometry-based screen to identify cysteine-dependent interactors for each peroxiredoxin isoform. A workflow for this screen is shown in Figure 2A. In short, cells expressing Flag-tagged peroxiredoxin were exposed to a short pulse of H_2_O_2_ followed by cell lysis. To prevent post-lysis oxidation and reduction, free thiols are quenched before and after lysis using N-ethylmaleimide (NEM) and iodoacetamide (IA), respectively. Flag-PRDX1-5 were pulled-down along with their interactors and subsequently exposed to a stringent high-salt wash to diminish non-covalent interactors. We then identified the interacting proteins using quantitative tandem mass spectrometry (MS/MS) followed by strict filtering and data analysis.

Figure 2B–F display scatter plots of the mean log_2_ intensities of the interacting proteins identified for PRDX1–5 wild-type (WT) and corresponding PRDX-C_PR_S mutants from three biological replicates. Marginal line graphs of the data distribution visualize data points that are hidden by overcrowding. Proteins interacting with both WT and mutant peroxiredoxin appear on a diagonal, while proteins interacting with only WT will have no intensity in the corresponding C_PR_S mutant and are thus visible off the diagonal. This data indicates that all five isoforms interact with a large number of proteins, and that many of those interactions are dependent on the peroxiredoxin catalytic cysteines. For most peroxiredoxin isoforms, but especially PRDX1, PRDX2 and PRDX3, the number of proteins that bind exclusively to wild-type is higher than to the corresponding mutant (visualized in the marginal plots of Figure 2B–F and Figure S2), suggesting that their interaction is cysteine-dependent. PRDX4 and PRDX5 seem to have a lower number of cysteine-dependent binders than the other isoforms. As yet we do not have a biological explanation for what the C_PR_S-specific interactors would represent.

A major challenge that is inherent to mass spectrometry data analysis, especially in proteome-wide protein-protein interaction studies like these, is that not all proteins are identified or quantified in each biological replicate and sample, therefore the data contains many missing values (an average of 34% per sample) [21]. It is well known that many of these missing values are non-random and that their absence correlates with a low overall intensity. If the hypothesis that proteins bind to peroxiredoxin in a largely disulfide-dependent manner holds true, missing values are actually expected to occur more often in the PRDX-C_PR_S mutant pull-downs. Thus, non-random missing data could hold important information in this experiment. In our analysis done for Figure 2B–F we simply ignored the missing values. Although this approach produces a general picture of the data, for a detailed analysis it is not optimal. Several MS data analysis approaches replace missing values with a reasonable value (imputation). However, imputing non-random missing values can overestimate peptide abundances and obscure available information, meaning that imputed values will be considered with the same confidence as measured values. This will lead to biased results, skewing data in a sample-dependent manner. For these reasons, we reanalyzed our data using a method called proDA (inference of protein differential abundance by probabilistic dropout analysis). This method aims to combine the sigmoidal dropout curve for missing values with the information from the observed values without direct value imputation. This allows for a more robust analysis that combines both the information from measured and missing values [22].

Cysteine-dependent interactors for each peroxiredoxin are visualized by plotting the fold change in intensity for each interactor pulled down with either WT, C_PR_S or both, on a log_2_ scale. In the proDA analysis, a large log_2_ fold change means that the protein was detected with high abundance in the WT peroxiredoxin pulldown and with no or low abundance in C_PR_S peroxiredoxin. Specifically, the difference between means (log_2_ fold change) is calculated based on the distribution of measured values and, when values are missing, the distribution of these is based on the average detection limit of the sample. To determine a suitable cutoff for our data, we randomized the data-labels a hundred times between WT and C_PR_S data and recalculated the log_2_ fold change. With the randomized data, we calculate that with a log_2_ fold change threshold of >0.401, 5% of the randomized data is within the 95^th^ quantile (i.e., an FDR of 5%,); on the other hand, 25.2% of the non-randomized data are included using the same threshold. In order to increase confidence, we used a more stringent cutoff of log_2_ fold change >1 (FDR of 0.02%, Figure S3). Therefore, proteins are considered to be significantly enriched when detected with at least twice the abundance (i.e., log_2_ fold change >1) as compared to the average expression of a protein around the limit of detection of the experiment, and a *p*-value <0.05. A total of 1233 proteins pass these criteria as catalytic cysteine-dependent binders of peroxiredoxins (Figure 2G and Supplementary Table S1). Collectively, the results from our screen indicate that a large number of proteins form disulfide-dependent heterodimers with the five 2-Cys peroxiredoxin isoforms.

We set out to characterize the isoform-specific interaction partners of peroxiredoxins in more detail. Specifically, we asked what proportion has previously been identified in a large-scale mass-spectrometry-based screen for redox-sensitive proteins [17]. Of the proteins identified in our screen, 80.5% contained cysteines that have previously been reported as sensitive to oxidative modification, compared to 13% in the reference proteome (Figure 2I). We also observed this enrichment for redox-sensitive proteins for each individual peroxiredoxin isoform.

### 3.3. Peroxiredoxin Isoforms Interact with a Specific Set of Target Proteins

**Figure 3:**
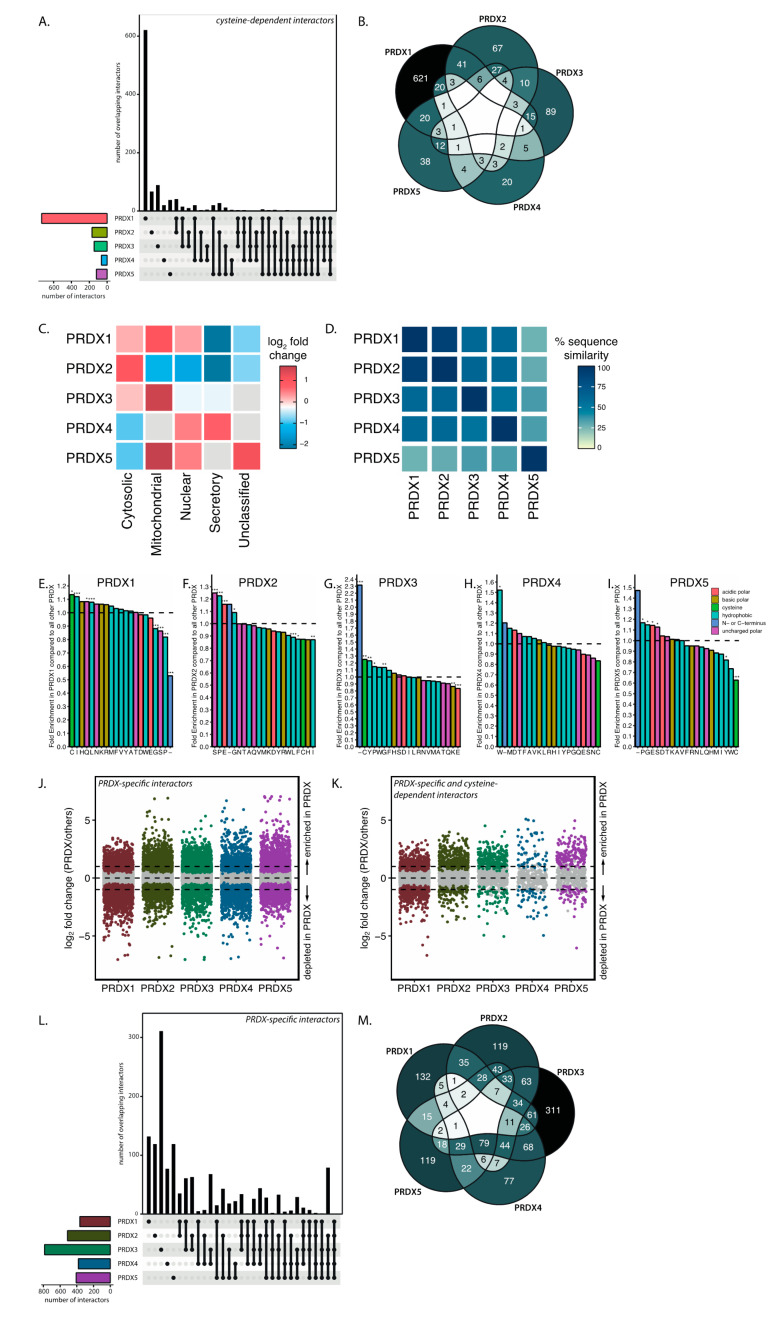
Peroxiredoxin isoforms interact with a specific set of target proteins. (**A**) UpSet plot and (**B**) Venn diagram to visualize cysteine-dependent target protein set intersections per peroxiredoxin isoform in a matrix layout. A color gradient from light (no overlap) to dark (high overlap) indicates the amount of overlap in the Venn diagram. (**C**) Localization of the top isoform-specific cysteine-dependent binders (>10-fold better binding to WT as compared to CprS) using the SubCellBarcode resource. Missing values are colored grey. (**D**) Sequence similarity for peroxiredoxins 1–5 calculated using the pairwise alignment tool EMBOSS needle. (**E–I**) Bar charts representing fold enrichment of the local amino acid composition around each cysteine in isoform-specific, cysteine-dependent peroxiredoxin interactors compared to the interactors of all other peroxiredoxin isoforms. Eight amino acids were extracted centered around each cysteine, with sequences shuffled 100 times as a control. * *p* < 0.05; ** *p* < 0.01; *** *p* < 0.001. (**J**) Scatter plot of log_2_ fold change comparing each peroxiredoxin isoform to all other isoforms. Horizontal lines are positioned at log_2_ fold change of 1 and –1 (i.e., a 2-fold change). Colored proteins are identified with Benjamini–Hochberg adjusted *p* < 0.05. (**K**) Similar to (**J**) but filtered for cysteine-dependent binders as analyzed in Figure 2G. (**L**) UpSet plot and (**M**) Venn diagram visualizing peroxiredoxin isoform-specific target protein set intersections in a matrix layout. A color gradient from light (no overlap) to dark (high overlap) indicates the amount of overlap in the Venn diagram.

The subcellular localization of the five 2-Cys peroxiredoxin isoforms, as well as their reaction kinetics, are not identical, and we therefore predicted that this could be reflected in their cysteine-dependent interactomes. We therefore next asked what the extent of overlap is between cysteine-dependent binding partners of different peroxiredoxin isoforms. A quantitative analysis of the intersections between the interactomes is shown in a matrix layout using an Upset plot (Figure 3A). Figure 3B presents the overlap in a more traditional color-coded Venn diagram. Interestingly, in this comparison we found that each peroxiredoxin isoform has a largely differential set of cysteine-dependent binding partners. This suggests that each of the peroxiredoxins catalyze the oxidation of a specific set of substrates.

Next, we investigated whether the subcellular localization of isoform-specific cysteine-dependent binding partners corresponds with the known localization of peroxiredoxin isoforms. To do so, we used SubCellBarCode [19], a resource that documents the subcellular localization of proteins in multiple cell lines. Indeed, most peroxiredoxin-binding proteins are overrepresented in the compartment where the peroxiredoxin isoform they interact with is reportedly localized (Figure 3C). For example, mitochondrial PRDX3, ER-localized PRDX4 and nuclear PRDX5 show an enrichment of proteins in the mitochondrial, secretory and nuclear compartments, respectively. The analysis is thus in line with the hypothesis that isoform-specific preferred localization could, at least to some extent, explain the peroxiredoxin specificity of binding partners. A note of caution is due here since the generalizability to other cell lines is uncertain; additionally, not all databases report the same predominant localization for the peroxiredoxins [23,24,25]. This may vary depending on the cell state, tissue type, cell cycle and post-translational modifications (for an overview, see [26]).

PRDX1 and PRDX2 share 90% sequence similarity (Figure 3D) and have the same reported subcellular localization (cytoplasm), but we found approximately one third of PRDX2 interactors to overlap with PRDX1 interaction partners, suggesting that localization is apparently not the sole determinant of peroxiredoxin isoform-specific disulfide-dependent binding. Differences in the molecular interface surrounding the cysteines of the binding partners that bind the catalytic cysteine of the peroxiredoxin isoforms could be the underlying mechanism behind this specific binding. For instance, it is known that deprotonation of the cysteine thiol at physiological pH is governed by the local environment, and the thiolate is more readily oxidized [27]. Recently, it was suggested that positively charged amino acid side chains (arginine, lysine, histidine) and the N-terminus can stabilize the thiolate of a proximal cysteine [17]. We therefore investigated whether the local environment around the cysteines of peroxiredoxin isoform-specific interactors is enriched for certain amino acids that can potentially alter the reactivity of a proximal cysteine. A challenge here is that our screen does not report on which specific cysteine of a binding partner is involved in the interaction with the peroxiredoxin, and we therefore analyzed all cysteines present in the binding partner, which would likely dilute any specific motifs. To investigate the presence of characteristic molecular environments, we extracted the eight amino acids flanking all cysteine residues of cysteine-dependent interactors for each peroxiredoxin isoform. Subsequently, we examined the amino acids surrounding each cysteine and calculated the fold change in the presence of each amino acid in one peroxiredoxin compared to all other isoforms. The amino acid enrichment or depletion in possible binding sites of specific peroxiredoxin isoforms is shown in Figure 3E–I. In general, these calculations suggest that the local amino acid composition of interactors of a specific peroxiredoxin looks different from that of the interactors of the other peroxiredoxin isoforms. Note that this analysis is based purely on the primary sequence of the binding partners, and that a 3D structure and pinpointing which cysteine actually forms the interaction would likely reveal a clearer picture of differences in the molecular interface, but we consider that beyond the scope of this study.

The analysis of peroxiredoxin interactors described above is based on the comparison of cysteine-dependent binders to each peroxiredoxin isoform. It is possible, however, that proteins bind to one peroxiredoxin isoform in a cysteine-dependent manner, but independent of cysteines to another peroxiredoxin isoform. To analyze the peroxiredoxin-specificity for cysteine-dependent interactors with a different approach, we re-analyzed our dataset in order to identify isoform-specific interactors. For each peroxiredoxin, we fit a new probabilistic dropout model using proDA, now testing which proteins are enriched in each peroxiredoxin isoform compared to the other isoforms irrespective of cysteine-dependency. Isoform-dependent interactors for each peroxiredoxin are visualized by plotting the log_2_ fold change in abundance comparing PRDX1–5 (i.e., the difference between the analyzed peroxiredoxin and all other isoforms on a log_2_ scale). Proteins that were enriched in pulldowns for one peroxiredoxin isoform (WT) but not in the other isoforms (with a log ratio of >1) are considered peroxiredoxin-specific binders. These data show a large number of proteins that bind to peroxiredoxins in an isoform-specific manner, irrespective of whether this is cysteine-dependent (Figure 3J). Again, we see that all peroxiredoxin isoforms have a large set of peroxiredoxin-specific interactors, which accords with our earlier observations, suggesting that each peroxiredoxin isoform has a largely differential set of cysteine-dependent binding partners (Figure 3A,B). Overlaying this analysis with the results obtained for cysteine-dependency in Figure 2G results in Figure 3K, which shows preferential binding partners for each peroxiredoxin isoform that do not bind to the C_PR_S mutant of that isoform. This is shown in the Upset plot and Venn diagram in Figure 3L and M, respectively.

Although we use a stringent wash buffer (containing 1 M NaCl) after the immunopurification of PRDX1–5 to enrich for disulfide-dependent covalent interactions, a large number of proteins were found to interact with the PRDX C_PR_S mutants (Supplemental Figure S4, light bars). The peroxiredoxin specificity of our data including both cysteine-dependent as well as -independent binders reassures that the observed interactions are not an artefact of post-lysis binding to all peroxiredoxins or the anti-Flag coated beads in which case they would be expected to largely overlap. When we look at the top 100 proteins with the lowest *p*-values and allow them to cluster per peroxiredoxin isoform, we also observe different patterns of interacting proteins for the five peroxiredoxin isoforms (Supplemental Figure S5).

These results support the idea that all five peroxiredoxin isoforms bind a specific set of cysteine-dependent interactors, which could suggest that peroxiredoxin isoforms each control the oxidation of a different subset of the proteome through redox relay signaling. The cysteine-dependent binding partners of peroxiredoxins contain a large fraction of proteins that have been reported as redox-sensitive. The localization of peroxiredoxin interactors, together with apparent differences in the local environment of interactor cysteines, could potentially explain isoform specificity.

### 3.4. The Peroxidatic Cysteine Is Sufficient to Form Cysteine-Dependent, Peroxiredoxin-Specific Interactions

Oxidized peroxiredoxins could in principle relay oxidizing equivalents to other thiols via two molecular mechanisms. The first mechanism involves the condensation of the sulfenylated peroxidatic cysteine (C_P_-SOH) of peroxiredoxins directly with the cysteine thiol of a target protein (Figure 1A, 1). A second possible mechanism involves a disulfide exchange reaction of the disulfide between the peroxidatic and resolving cysteine (C_P_-S-S-C_R_) in oxidized peroxiredoxins with a target protein thiol (Figure 1A, 2). The SOH-mediated mechanism in principle only needs the peroxidatic cysteine of peroxiredoxin, whereas the S-S-mediated route is dependent on both catalytic cysteines. To test which of these mechanisms is involved in the formation of S-S-dependent PRDX-target heterodimers, we performed another mass-spectrometry-based screen similar to the one described above, but now comparing C_R_S mutants to the catalytic dead mutants (C_PR_S) of each peroxiredoxin isoform. The C_R_S mutant could in theory still relay through the (C_P_-SOH) but not the C_P_-S-S-C_R_ dependent mechanism.

**Figure 4:**
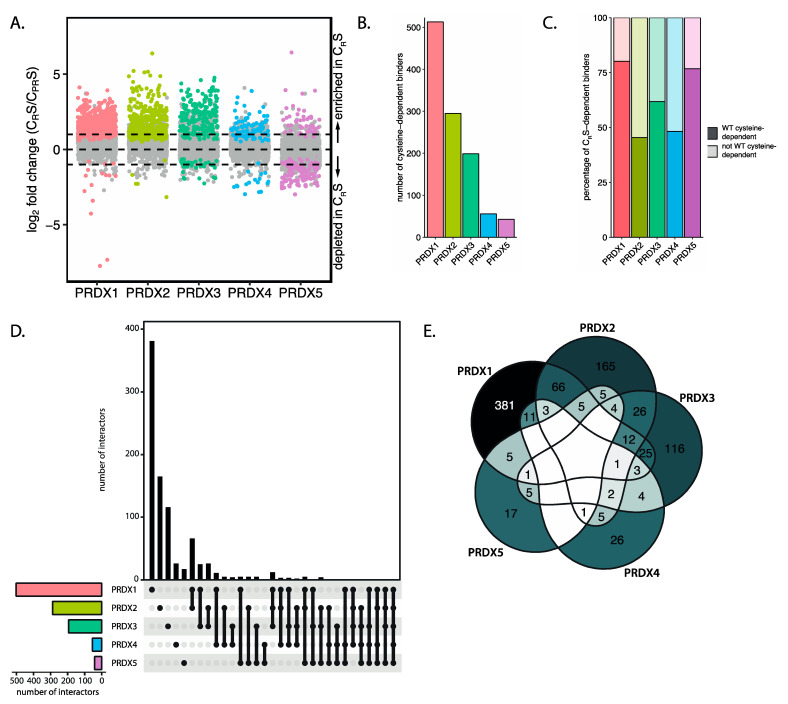
The peroxidatic cysteine is sufficient to form many cysteine-dependent, peroxiredoxin-specific interactions. (**A**) Scatter plot of the log_2_ fold change in binding of proteins to each of the C_R_S-mutant peroxiredoxin isoforms compared to the C_PR_S-mutant of the same peroxiredoxin isoform. Horizontal lines are positioned at log_2_ fold change of 1 and –1 (i.e., a 2-fold change). Colored proteins are identified with a *p*-value <0.05. (**B**) Number of peroxidatic cysteine C_P_-dependent interaction partners per peroxiredoxin isoform identified with *p*-value <0.05 and log_2_ fold change >1 in our screen. (**C**) Bar chart representing the percentage of peroxidatic cysteine C_P_-dependent binders that are also identified as cysteine-dependent binders in wild-type peroxiredoxin (Figure 2). (**D**) UpSet plot and (**E**) Venn diagram that visualize peroxidatic cysteine C_P_-dependent target protein set intersections per peroxiredoxin isoform in a matrix layout. A color gradient from white (no overlap) to blue (high overlap) indicates the amount of overlap in the Venn diagram.

Peroxiredoxin C_R_S mutants of all five isoforms can still participate in disulfide-dependent interactions with many proteins: a total of 1032 cysteine-dependent binding partners (compared to 1145 for wild-type) was identified (Figure 4A,B and Supplemental Table S3). This strongly suggests that many peroxiredoxin binding partners are capable of binding through the C_P_-SOH-mediated mechanism. We then analyzed how many of the cysteine-dependent binders of PRDX-C_R_S are also identified as WT cysteine-dependent binders. We found that of the proteins identified to form cysteine-dependent interactions with PRDX-C_R_S, over 60% for PRDX1, PRDX3 and PRDX5 and approximately half for PRDX2 and 4, also do so using WT peroxiredoxins as bait (Figure 4C). When comparing peroxidatic cysteine-dependent binding partners for the five peroxiredoxin isoforms we again found that each has a largely different set of target proteins (Figure 4D,E). This confirms our conclusion regarding the peroxiredoxin-isoform-specific binding partners that we based on the mass-spectrometry screen comparing wild-type peroxiredoxins.

Thus, for many cysteine-dependent interactors of the peroxiredoxins, the peroxidatic cysteine suffices to mediate the interaction in the absence of the resolving catalytic cysteine. This does not mean however that the interaction cannot be established starting from C_P_-S-S-C_R_ under normal conditions.

### 3.5. Peroxiredoxins Bind Target Proteins Via Two Distinct Mechanisms

**Figure 5:**
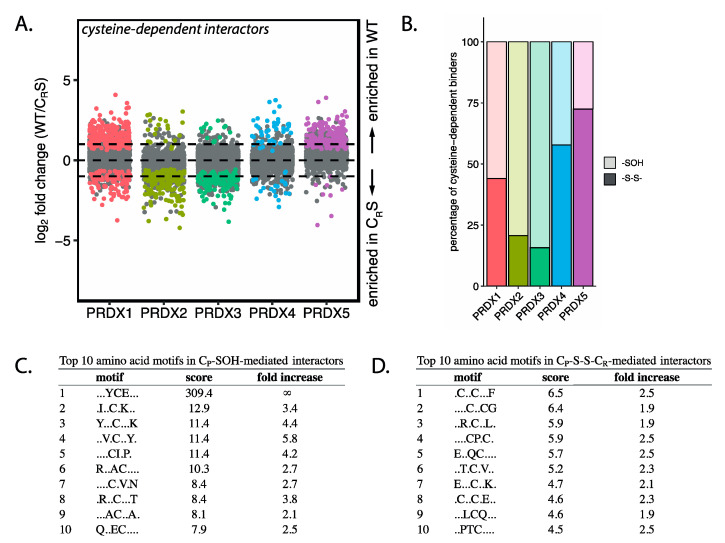
Peroxiredoxins interact via two distinct mechanisms. (**A**) Analysis comparing cysteine-dependent interactors of wild-type and C_R_S-mutant peroxiredoxins showing the log_2_ fold change in binding of proteins to wild-type compared to the C_R_S-mutant peroxiredoxin isoforms. Horizontal lines are positioned at log_2_ fold change of 1 and –1 (i.e., a 2-fold change). Colored proteins are identified as cysteine-dependent binders in the analyses presented in Figure 2 and Figure 4. (**B**) Bar chart representing the percentage of C_P_-S-S-C_R_-mediated and C_P_-SOH cysteine-dependent interactors per peroxiredoxin isoform. Proteins interacting with the C_R_S-mutant only are not included in this analysis. Local amino acid motifs around each cysteine of C_P_-SOH cysteine-dependent interactors (**C**) and C_P_-S-S-C_R_-mediated interactors of peroxiredoxins (**D**) with sequences shuffled 100 times as a control.

We observed that cysteine-dependent heterodimerization of peroxiredoxins with many target proteins also occurs in mutants lacking the resolving cysteine. However, this might not apply to all identified interactors, and in principle, a C_P_-S-S-C_R_ could be required for a subset of the cysteine-dependent peroxiredoxin binding partners. We questioned whether, for each peroxiredoxin, all binding partners follow the C_P_-SOH-mediated mechanism, or whether some might use the C_P_-S-S-C_R_-mediated route. We compared cysteine-dependent binders of wild-type peroxiredoxins to cysteine-dependent binders of the C_R_S mutant (Figure 5A and Table S4). Proteins that do not bind the resolving cysteine mutant C_R_S, but that do bind the wild-type peroxiredoxin, likely depend on the C_P_-S-S-C_R_ -mediated relay mechanism. We found that the majority of cysteine-dependent interactors of the WT and C_R_S mutant datasets overlap. However, a number of proteins are exclusively found to interact with the WT peroxiredoxins and hence depend on a C_P_-S-S-C_R_ mediated relay. We also identified a small number of interactors that are only pulled down with the C_R_S mutant, but not with the WT peroxiredoxin.

Next, we compared the extent of C_P_-S-S-C_R_- and C_P_-SOH-mediated binding between peroxiredoxin isoforms. As shown in Figure 5B, there are large differences in the distribution of the relay mechanisms between isoforms. The percentage of C_P_-S-S-C_R_-mediated binders ranges from 21% to 16% for PRDX2 and PRDX3, respectively, while PRDX1 and PRDX4 have 44% and 58% C_P_-S-S-C_R_ interactors. Interestingly, as much as 73% of PRDX5 interactors follow the C_P_-S-S-C_R_-mechanism. It is not unlikely that the absence of the resolving cysteine may actually stabilize or facilitate disulfide formation with cysteines in other proteins.

A possible explanation as to why disulfides with binding partners are formed preferably starting from either C_P_-SOH or C_P_-S-S-C_R_ might lie in the amino acid region surrounding the cysteine in an interacting protein. Since C_P_-SOH and C_P_-S-S-C_R_ are structurally distinct, the local environment of an interacting cysteine could determine whether an interaction is favorable. We investigated whether the local environments of cysteines in interactors that preferentially bind to C_P_-SOH or C_P_-S-S-C_R_ can be distinguished. Interactors of all peroxiredoxin isoforms were separated into two groups based on their preference for either C_P_-SOH or C_P_-S-S-C_R_-mediated interaction. For both groups, we extracted the eight amino acids flanking all cysteine residues. Using the motif-x algorithm [20], we analyzed potential motifs in each group of interactors, using the other group as a background. While both C_P_-SOH and C_P_-S-S-C_R_-mediated interactors are enriched in the CxxC motif, a well-known motif in redox proteins (Tables S5 and S6), differences in enriched motifs found in C_P_-SOH and C_P_-S-S-C_R_-mediated interactors could be found (Figure 5C,D); for example, the YCE motifs enriched in C_P_-SOH-mediated interactors as compared to C_P_-S-S-C_R_-mediated interactors. This supports the idea that proteins might preferentially form interactions with peroxiredoxin C_P_-SOH or C_P_-S-S-C_R_ based on their local amino acid composition.

Based on these observations, we conclude that oxidized peroxiredoxins bind their interaction partners starting from the -SOH- or -S-S- state, and that a subset of interactors can only bind peroxiredoxin in the -S-S- state. A possible explanation for this might be found in amino acid motifs surrounding cysteine residues in the interaction partners. -S-S- vs. -SOH dependent relay could point to an additional level of specificity in peroxiredoxin-based interaction that may have implications for cellular redox regulation.

## 4. Discussion

Peroxiredoxin-catalyzed oxidation has been suggested to answer the question as to how thiols with low intrinsic reactivity can be oxidized by low levels of H_2_O_2_ despite the presence of abundant and highly reactive peroxidases. Here we show that all peroxiredoxin isoforms are capable of forming numerous cysteine-dependent heterodimers. Our in-depth mass-spectrometry and complementary bioinformatics approach provides, first of all, a resource of potential 2-Cys peroxiredoxin-catalyzed cysteine oxidation substrates. Many of the proteins that we identified as cysteine-dependent peroxiredoxin binders were indeed identified previously to contain redox sensitive cysteines [17]. This overlap could point at a major role for peroxiredoxins in cysteine oxidation in other proteins. It is not clear what follows after peroxiredoxin-dependent cysteine oxidation, but one could think of three possible scenarios following intermolecular disulfide formation between peroxiredoxin and a target protein. 1) The intermolecular disulfide could be rapidly resolved by disulfide exchange to the resolving cysteine of the peroxiredoxin, forming the canonical C_P_-S-S-C_R_ and leaving the target reduced. 2) The intermolecular disulfide could be resolved by disulfide exchange to another cysteine in the binding protein (or protein complex), forming an intra- or intermolecular disulfide in that protein and leaving peroxiredoxin reduced. 3) The intermolecular disulfide dependent complex of peroxiredoxin and its target could represent a novel type of post-translational modification on cysteine, that for instance alters the function of the target, that we would like to coin S-peroxiredoxinylation (S-PRDXylation). Others have shown that many of the PRDX1 and PRDX2-dependent binders were also identified in a TRX-trap pull down, confirming that proteins binding to peroxiredoxin in a cysteine-dependent manner indeed become oxidized [13]. This observation probably does not exclude S-PRDXylation. Widespread S-PRDXylation could also be in accordance with the identification of many oxidation-sensitive cysteines in redox proteomics studies, as these would not distinguish S-PRDXylation from other intra- or intermolecular disulfides [28]. Besides a role as a post-translational modification impacting the function of specific proteins, widespread peroxiredoxinylation could in principle also serve as a redox buffer.

Our data furthermore provides evidence that the peroxiredoxin-dependent redox relay model could also explain how selectivity in redox signaling can be achieved. Selectivity stems from the observation that each peroxiredoxin isoform interacts with a largely specific subset of proteins. This could in part depend on isoform-specific subcellular localization, but the relatively low overlap in binders for PRDX1 and PRDX2, which share the same subcellular localization and a high sequence similarity, suggests that this is not the only determinant for binding of a protein to a specific peroxiredoxin isoform. Analysis of local structural differences surrounding the region around the cysteine of the binding protein could also contribute to selective binding of proteins to the different peroxiredoxins. A second layer of specificity is suggested by the observation that peroxiredoxin-mediated relays can proceed through two distinct molecular mechanisms, starting from either C_P_-SOH or C_P_-S-S-C_R_, and that peroxiredoxin isoforms and targets display varying preferences for these mechanisms. Each peroxiredoxin has different kinetics for C_P_-SOH and C_P_-S-S-C_R_ formation and reduction, and these kinetics could dictate which cysteines in target proteins can be oxidized under specific conditions. For instance, at low levels of peroxide, PRDX2 would be the first to form C_P_-SOH, whereas only under conditions where TRX activity is limiting, oxidized peroxiredoxin in the C_P_-S-S-C_R_ form would be sufficiently abundant to oxidize another set of targets. Interestingly, when we look at isoform-specific differences in the frequency of C_P_-SOH and C_P_-S-S-C_R_-mediated interactors, we find that there are large differences in the distribution of these relay mechanisms between isoforms.

A fair number of proteins seems to bind peroxiredoxins independent of its catalytic cysteines (Figure 3L, grey dots), despite high-salt washing. The peroxiredoxin isoform dependent specificity irrespective of cysteine-dependency suggests that these interactions are probably not artefacts of the used method. This leaves the possibility that some of these proteins could function as adaptor proteins to facilitate peroxiredoxin-dependent relays to cysteine-dependent binding proteins. Although this would need to be explored, adaptor proteins have been shown to be involved in peroxidase-dependent redox relays. For instance, Orp1-dependent Yap1 oxidation is dependent on the presence of the adapter protein Ybp1, shielding oxidized Orp1 from reduction [29,30]. Similarly, the PRDX2-STAT2 redox relay depends on association with the membrane-associated scaffold protein ANXA2 [31]. We indeed also identify ANXA2 as a PRDX2-specific and cysteine-dependent interactor in our screen (Table S2). It is conceivable that many more peroxiredoxin-based redox relays may proceed via the formation of ternary complexes with scaffold proteins. This would not only increase the chances that a peroxiredoxin finds a target, but would also add another level of specificity, coming from the interaction of specific peroxiredoxin isoforms with specific scaffolds for the relay of oxidation to subsets of target proteins.

Taken together, our observations regarding widespread cysteine-dependent binding of proteins to the 2-Cys peroxiredoxins provides a model that could explain both the reactivity and selectivity of the extensive cysteine oxidation observed in response to low amounts of H_2_O_2_.

## 5. Conclusions

In conclusion, our findings suggest that all five human 2-Cys peroxiredoxins can form disulfide-dependent heterodimers with a large set of proteins, and that each peroxiredoxin isoform displays a preference for a subset of disulfide-dependent binding partners. We highlight the isoform-specific characteristics that might justify this preference. Furthermore, we propose that peroxiredoxin-based redox relays can progress via one of two molecular mechanisms starting from either C_P_-SOH or C_P_-S-S-C_R_. These findings provide a framework for peroxiredoxin biology and implicate a widespread role for peroxiredoxins in selectively transducing peroxide signals in order to generate appropriate signaling responses.

## 6. Limitations of this Study

The cut-offs used in the analysis of our mass-spectrometry screen are quite stringent, and whether proteins bind only in a cysteine-dependent manner or to only a certain peroxiredoxin isoform may not be as unambiguous. Furthermore, to keep the number of mass-spectrometry samples manageable the analysis was performed at a single timepoint following a single concentration of H_2_O_2_ treatment. It is not unthinkable that proteins found to interact specifically to one peroxiredoxin isoform in this study will in fact interact with others when analyzed at other timepoints or H_2_O_2_ concentrations. The use of the H_2_O_2_ treated C_PR_S mutants as a control rather than also including untreated samples for WT PRDX1–5 (for the same reason of keeping the number of mass-spectrometry samples manageable) may obscure whether proteins also bind peroxiredoxins under basal conditions, be it cysteine dependent or not. Future work will be needed to carefully validate each protein found as a cysteine-dependent peroxiredoxin interactor.

It is not unthinkable that differences in the level of overexpression of the Flag-tagged PRDX1–5 or their mutants may lead to variation in the number of proteins pulled down. In general, the C_PR_S mutants of each PRDX1–5 isoform had very similar expression and IP efficiency as compared to their wildtype isoform counterparts (see for instance Figure 1C, reducing IP and input), suggesting that whether an interactor is identified as a cysteine-dependent binder is not much affected by variable expression levels. The levels of overexpression of Flag-PRDX1–5 compared to each are somewhat variable, and for his reason MS/MS data was log_2_-transformed followed by quantile normalization to simultaneously correct for overall protein content and IP efficiency in an attempt to lower the chance that differences in expression levels affect our analysis.

The analysis of the chemical environment of cysteines oxidized through peroxiredoxin dependent relays would greatly benefit from knowing which cysteine in a binding partner is being oxidized. Here we have analyzed all cysteines in the interactors which obviously dilutes any specific pattern. Combining peroxiredoxin-interactome screens as described here with redox proteomics or ways to keep the disulfide between peroxiredoxin and its targets intact and suitable for analysis by MS/MS in future studies could be a way to achieve this. It is difficult to unambiguously exclude that proteins no longer bind to the used peroxiredoxin mutants due to structural changes other than loss of the cysteine thiol. For the resolving cysteine mutants at least, a recent study shows that the cysteine to serine mutation has only a limited effect on the rate of oxidation of the peroxidatic cysteine in PRDX2 [32]. Characterization of the functional consequences of specific peroxiredoxin-based interactions is outside the scope of this study. However, it would be interesting to investigate the mechanisms and fate of these complexes in more detail. Additionally, further work is needed to link the mechanisms of peroxiredoxin specificity to biological cues that determine downstream signaling.

## Data Availability

The data presented in this study are openly available from the corresponding authors upon reasonable request. The mass spectrometry proteomics data have been deposited into the ProteomeXchange Consortium via the PRIDE [16] partner repository with the dataset identifier PXD024114. Publicly available datasets were analyzed in this study. These data can be found here: https://oximouse.hms.harvard.edu and https://doi.org/10.1016/j.molcel.2018.11.035.

## Supplementary Materials

The following are available online at https://www.mdpi.com/article/10.3390/antiox10040627/s1, Figure S1: Immunoprecipitated Flag-PRDX Western blots, Figure S2: ridge plot for the mean log ratio of wild-type and CPRS-peroxiredoxin, Figure S3: log_2_ fold change distribution of data and randomized data, Figure S4: bar chart specifying the number of cysteine-dependent interactors within the peroxiredoxin isoform-specific interactors, Figure S5: heatmaps op the top 100 isoform-specific binders, Table S1: cysteine-dependent interactors (wild-type), Table S2: isoform-specific cysteine-dependent interactors, Table S3: peroxidatic cysteine-dependent interactors (C_R_S), Table S4: all cysteine-dependent interactors (wild-type and C_R_S), Table S5: amino acid motifs in C_P_-SOH-mediated interactors, Table S6: amino acid motifs in C_P_-S-S-C_R_-mediated interactors.

## List of Abbreviations

-S-S-	Disulfide
-SOH	Sulfenic acid
C_P_	Peroxidatic cysteine
C_R_	Resolving cysteine
ER	Endoplasmic reticulum
FDR	False discovery rate
Grx	Glutaredoxin
H_2_O_2_	Hydrogen peroxide
MS/MS	Tandem mass spectrometry
MW	Molecular weight
NADPH	Nicotinamide adenine dinucleotide phosphate
PDI	Protein disulfide isomerase
PRDX	Peroxiredoxin
ROS	Reactive oxygen species
S-PRDXylation	S-peroxiredoxinylation
Trx	Thioredoxin
WT	Wild-type
