# Supplementary figures and images for: The Human 2-Cys Peroxiredoxins form Widespread, Cysteine-Dependent- and Isoform-Specific Protein-Protein Interactions
Source: Antioxidants (Basel). 2021 Apr 20;10(4):627. doi: 10.3390/antiox10040627 (PMC8073576; doi:10.3390/antiox10040627)

S1

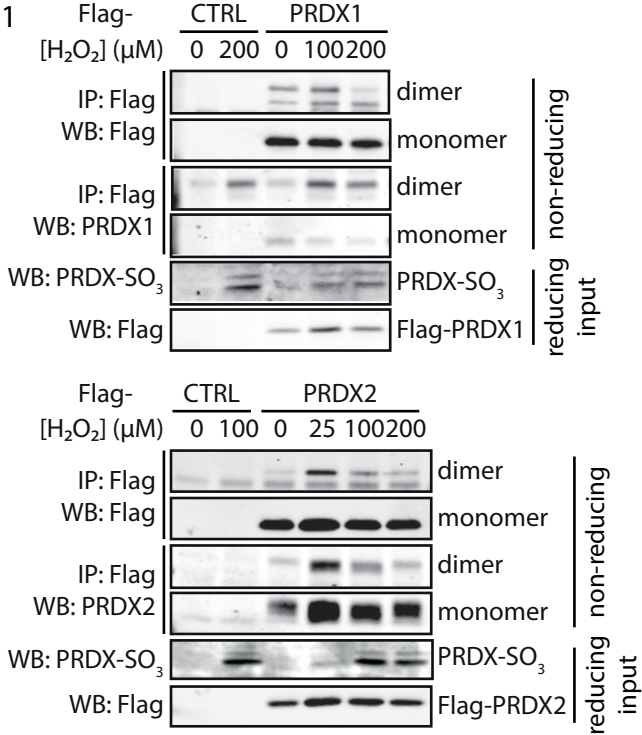

S2

number of proteins

PRDX1

PRDX2

PRDX3

PRDX4

PRDX5

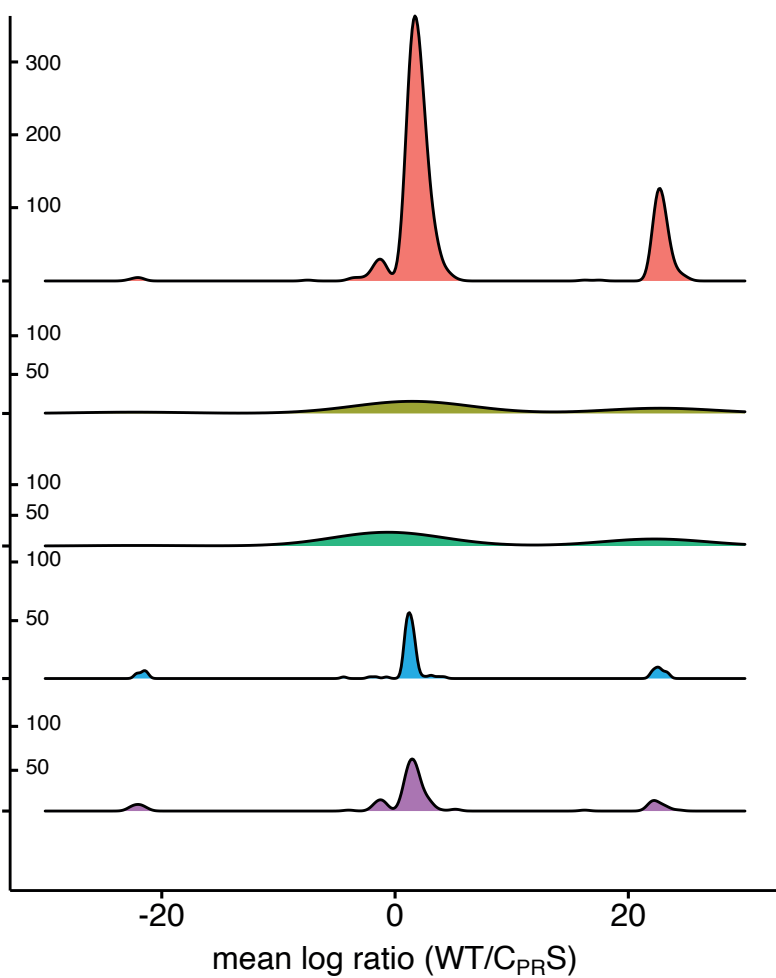

S3

 $\log_2$  difference WT/C<sub>PR</sub>S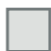

Random

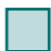

Real

5% 0.02% FDR

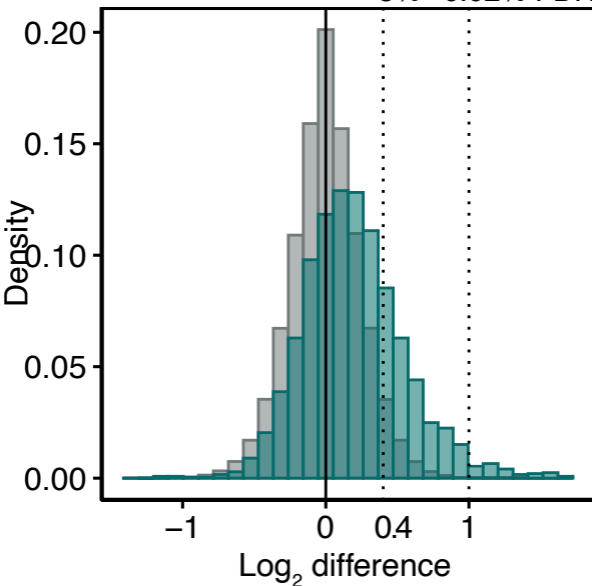

S5

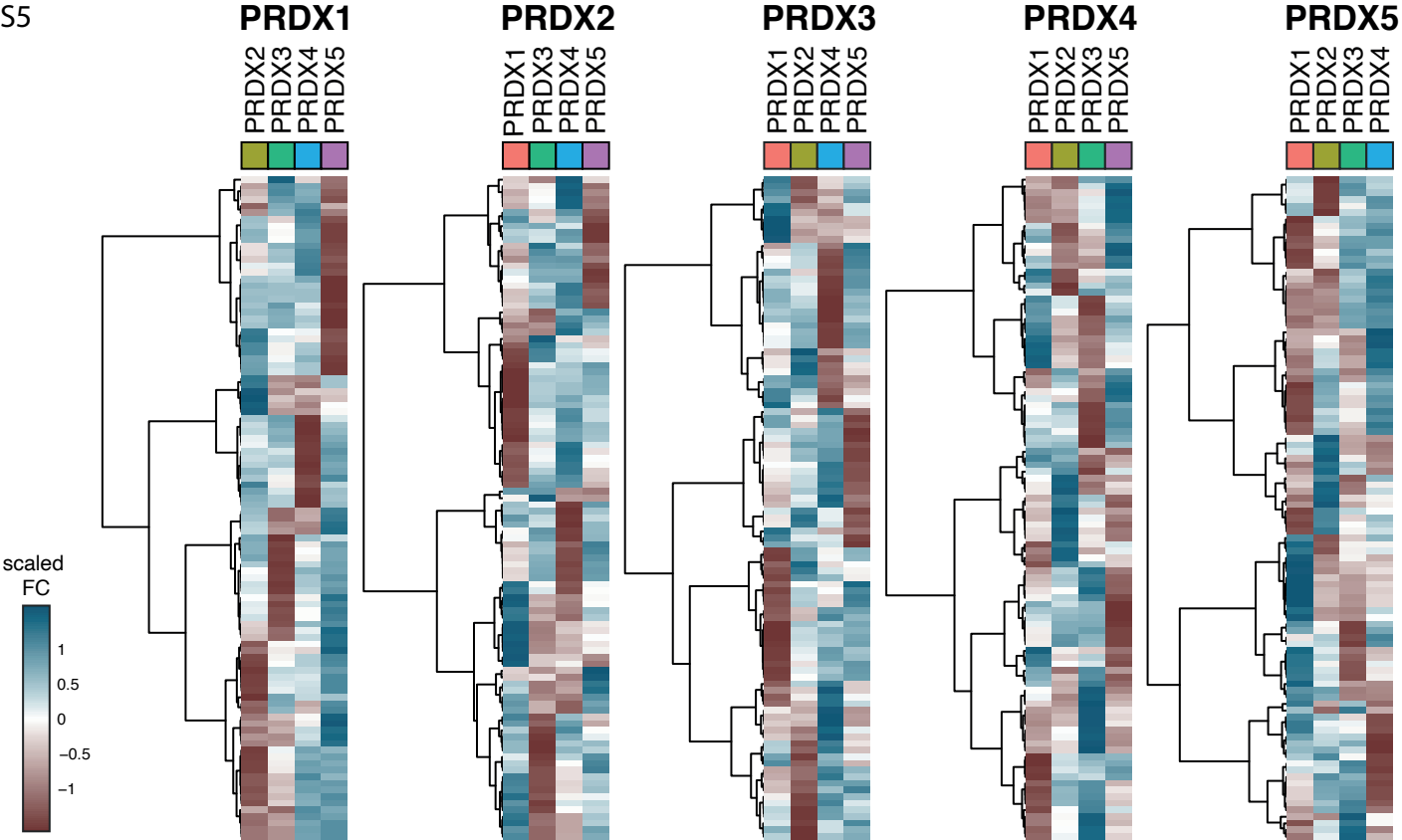

S4

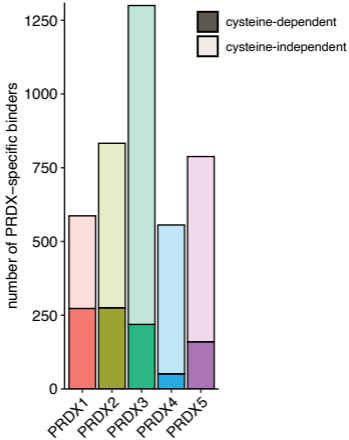

Supplement: Supplementary file 1 [file antioxidants-10-00627-s001.zip › antioxidants-1173803-supplementary.pdf]
