# Supplementary material for: The Human 2-Cys Peroxiredoxins form Widespread, Cysteine-Dependent- and Isoform-Specific Protein-Protein Interactions
Source: Antioxidants (Basel). 2021 Apr 20;10(4):627. doi: 10.3390/antiox10040627 (PMC8073576; doi:10.3390/antiox10040627)
Supplement: Supplementary file 1 [file AllSupplTables.pdf › AllSupplTables.pdf]

Table S1. Cysteine-dependent interactors (wild-type PRDX/PRDX-$C_{PR}S$)

uniprot ID	gene	protein	log ratio	p-value
**PRDX1 cysteine-dependent interactors**				
P15924	DSP	Desmoplakin	4.9	<0.001
Q15149	PLEC	Plectin	4.8	<0.001
Q15185	PTGES3	Prostaglandin E synthase 3	4.8	<0.001
P08559	PDHA1	Pyruvate dehydrogenase E1 component subunit alpha, somatic form, mitochondrial	4.5	<0.001
P62273	RPS29	40S ribosomal protein S29	4.4	<0.001
A0A024R4E5	HDLBP	High density lipoprotein binding protein	4.4	<0.001
Q9Y2V2	CARHSP1	Calcium-regulated heat-stable protein 1	4.4	<0.001
P55084	HADHB	Trifunctional enzyme subunit beta, mitochondrial	4.4	<0.001
P49792	RANBP2	E3 SUMO-protein ligase RanBP2	4.3	<0.001
P24666	ACP1	Low molecular weight phosphotyrosine protein phosphatase	4.3	<0.001
Q9GZT3	SLIRP	SRA stem-loop-interacting RNA-binding protein, mitochondrial	4.1	<0.001
Q15654	TRIP6	Thyroid receptor-interacting protein 6	4.0	<0.001
E7EX90	DCTN1	Dynactin subunit 1	4.0	<0.001
Q01081	U2AF1	Splicing factor U2AF 35 kDa subunit	4.0	0.001
Q9BY32	ITPA	Inosine triphosphate pyrophosphatase	3.9	<0.001
Q14008	CKAP5	Cytoskeleton-associated protein 5	3.8	<0.001
Q9P2J5	LARS1	Leucine--tRNA ligase, cytoplasmic	3.8	<0.001
P48047	ATP5PO	ATP synthase subunit O, mitochondrial	3.8	<0.001
Q16513	PKN2	Serine/threonine-protein kinase N2	3.7	<0.001
P30048	PRDX3	Thioredoxin-dependent peroxide reductase, mitochondrial	3.7	<0.001
A0A087WZ13	RAVER1	Ribonucleoprotein PTB-binding 1	3.7	<0.001
P39023	RPL3	60S ribosomal protein L3	3.7	<0.001
P13667	PDIA4	Protein disulfide-isomerase A4	3.6	<0.001
P61011	SRP54	Signal recognition particle 54 kDa protein	3.6	<0.001
C9JZR2	CTNND1	Catenin delta-1	3.6	<0.001
Q53H12	AGK	Acylglycerol kinase, mitochondrial	3.6	<0.001
P46940	IQGAP1	Ras GTPase-activating-like protein IQGAP1	3.5	<0.001
E3W994	CLASP2	CLIP-associating protein 2	3.5	<0.001
P62316	SNRPD2	Small nuclear ribonucleoprotein Sm D2	3.5	<0.001
P53621	COPA	Coatomer subunit alpha	3.5	<0.001
Q99615	DNAJC7	DnaJ homolog subfamily C member 7	3.5	<0.001
P62829	RPL23	60S ribosomal protein L23	3.5	<0.001
Q9UQE7	SMC3	Structural maintenance of chromosomes protein 3	3.4	<0.001
Q9H0C8	ILKAP	Integrin-linked kinase-associated serine/threonine phosphatase 2C	3.4	<0.001
Q14684	RRP1B	Ribosomal RNA processing protein 1 homolog B	3.4	<0.001
Q7Z6Z7	HUWE1	E3 ubiquitin-protein ligase HUWE1	3.4	<0.001
P31689	DNAJA1	DnaJ homolog subfamily A member 1	3.4	<0.001
Q7L2E3	DHX30	ATP-dependent RNA helicase DHX30	3.3	<0.001
E7EVH7	E7EVH7	Kinesin light chain	3.3	<0.001
Q14204	DYNC1H1	Cytoplasmic dynein 1 heavy chain 1	3.3	<0.001
Q9ULV4	CORO1C	Coronin-1C	3.3	<0.001
P63165	SUMO1	Small ubiquitin-related modifier 1	3.3	<0.001
P49790	NUP153	Nuclear pore complex protein Nup153	3.3	<0.001
P15170	GSPT1	Eukaryotic peptide chain release factor GTP-binding subunit ERF3A	3.3	<0.001
P23258	TUBG1	Tubulin gamma-1 chain	3.3	0.001
Q92879	CELF1	CUGBP Elav-like family member 1	3.2	<0.001
Q14683	SMC1A	Structural maintenance of chromosomes protein 1A	3.2	<0.001
P55809	OXCT1	Succinyl-CoA:3-ketoacid coenzyme A transferase 1, mitochondrial	3.2	<0.001
O75531	BANF1	Barrier-to-autointegration factor	3.2	0.001
O14964	HGS	Hepatocyte growth factor-regulated tyrosine kinase substrate	3.2	0.001
Q9NY93	DDX56	Probable ATP-dependent RNA helicase DDX56	3.2	0.001
O94760	DDAH1	N(G),N(G)-dimethylarginine dimethylaminohydrolase 1	3.2	0.002
Q08211	DHX9	ATP-dependent RNA helicase A	3.2	0.030
Q9C0C2	TNKS1BP1	182 kDa tankyrase-1-binding protein	3.1	<0.001
Q01082	SPTBN1	Spectrin beta chain, non-erythrocytic 1	3.1	<0.001
H3BPE1	MACF1	Microtubule-actin cross-linking factor 1, isoforms 1/2/3/5	3.1	<0.001
Q9BQG0	MYBBP1A	Myb-binding protein 1A	3.1	<0.001
Q7Z406	MYH14	Myosin-14	3.1	<0.001
P22307	SCP2	Non-specific lipid-transfer protein	3.1	<0.001
P62266	RPS23	40S ribosomal protein S23	3.1	<0.001
Q5SW79	CEP170	Centrosomal protein of 170 kDa	3.1	<0.001
Q6P1L8	MRPL14	39S ribosomal protein L14, mitochondrial	3.1	<0.001
P55735	SEC13	Protein SEC13 homolog	3.1	0.001
P21964	COMT	Catechol O-methyltransferase	3.1	0.001
Q99460	PSMD1	26S proteasome non-ATPase regulatory subunit 1	3.1	0.001
P11387	TOP1	DNA topoisomerase 1	3.1	0.002
Q5JTH9	RRP12	RRP12-like protein	3.0	<0.001
Q96I25	RBM17	Splicing factor 45	3.0	<0.001
E7ESP9	NEFM	160 kDa neurofilament protein	3.0	<0.001
P62851	RPS25	40S ribosomal protein S25	3.0	<0.001
Q13561	DCTN2	Dynactin subunit 2	3.0	<0.001
Q9UHI6	DDX20	Probable ATP-dependent RNA helicase DDX20	3.0	<0.001
Q9UNE7	STUB1	E3 ubiquitin-protein ligase CHIP	3.0	<0.001
Q06124	PTPN11	Tyrosine-protein phosphatase non-receptor type 11	3.0	<0.001
P19525	EIF2AK2	Interferon-induced, double-stranded RNA-activated protein kinase	3.0	0.001
Q96KB5	PBK	Lymphokine-activated killer T-cell-originated protein kinase	3.0	0.004
Q9HD26	GOPC	Golgi-associated PDZ and coiled-coil motif-containing protein	3.0	0.005
A0A0A0MRT6	ABI1	Abl interactor 1	2.9	<0.001
Q8N9T8	KRI1	Protein KRI1 homolog	2.9	<0.001
Q8N1F7	NUP93	Nuclear pore complex protein Nup93	2.9	<0.001
E9PD53	SMC4	Structural maintenance of chromosomes protein	2.9	<0.001
P46060	RANGAP1	Ran GTPase-activating protein 1	2.9	<0.001
Q99961	SH3GL1	Endophilin-A2	2.9	<0.001
Q96N67	DOCK7	Dedicator of cytokinesis protein 7	2.9	<0.001
O94979	SEC31A	Protein transport protein Sec31A	2.9	<0.001
Q9H2G2	SLK	STE20-like serine/threonine-protein kinase	2.9	<0.001
Q9H0B6	KLC2	Kinesin light chain 2	2.9	<0.001
Q9UG54	MAP3K7	Mitogen-activated protein kinase kinase kinase	2.9	<0.001
P14174	MIF	Macrophage migration inhibitory factor	2.9	0.001
P27708	CAD	CAD protein [Includes: Glutamine-dependent carbamoyl-phosphate synthase	2.9	0.004
P68133	ACTA1	Actin, alpha skeletal muscle	2.9	0.015
Q7L1Q6	BZW1	Basic leucine zipper and W2 domain-containing protein 1	2.8	<0.001
I3L2Z5	MAZ	Myc-associated zinc finger protein	2.8	<0.001
Q5JRX3	PITRM1	Presequence protease, mitochondrial	2.8	<0.001
O43837	IDH3B	Isocitrate dehydrogenase [NAD] subunit beta, mitochondrial	2.8	<0.001
P63272	SUPT4H1	Transcription elongation factor SPT4	2.8	<0.001
P57740	NUP107	Nuclear pore complex protein Nup107	2.8	<0.001
P08708	RPS17	40S ribosomal protein S17	2.8	<0.001
Q9UG63	ABCF2	ATP-binding cassette sub-family F member 2	2.8	<0.001
P48444	ARCN1	Coatomer subunit delta	2.8	<0.001
P41252	IARS1	Isoleucine--tRNA ligase, cytoplasmic	2.8	<0.001
P36915	GNL1	Guanine nucleotide-binding protein-like 1	2.8	<0.001
Q9UHB6	LIMA1	LIM domain and actin-binding protein 1	2.8	<0.001
A0A1W2PRV5	SMN2	Survival motor neuron protein	2.8	0.001
Q9BV44	THUMPD3	THUMP domain-containing protein 3	2.8	0.002
O75874	IDH1	Isocitrate dehydrogenase [NADP] cytoplasmic	2.8	0.002
O95801	TTC4	Tetratricopeptide repeat protein 4	2.8	0.004
P43897	TSFM	Elongation factor Ts, mitochondrial	2.8	0.004
Q13813	SPTAN1	Spectrin alpha chain, non-erythrocytic 1	2.8	0.005
Q5VYK3	ECPAS	Proteasome adapter and scaffold protein ECM29	2.8	0.005
Q9NR30	DDX21	Nucleolar RNA helicase 2	2.8	0.009
Q02241	KIF23	Kinesin-like protein KIF23	2.7	<0.001
P61163	ACTR1A	Alpha-centractin	2.7	<0.001
Q8WVM8	SCFD1	Sec1 family domain-containing protein 1	2.7	<0.001
P20042	EIF2S2	Eukaryotic translation initiation factor 2 subunit 2	2.7	<0.001
P83731	RPL24	60S ribosomal protein L24	2.7	<0.001
A0A2R8YDQ9	SUCLA2	Succinate--CoA ligase [ADP-forming] subunit beta, mitochondrial	2.7	<0.001
P35658	NUP214	Nuclear pore complex protein Nup214	2.7	<0.001
Q86V48	LUZP1	Leucine zipper protein 1	2.7	<0.001
O60493	SNX3	Sorting nexin-3	2.7	0.001
Q15717	ELAVL1	ELAV-like protein 1	2.7	0.003
P23919	DTYMK	Thymidylate kinase	2.7	0.003
P46013	MKI67	Proliferation marker protein Ki-67	2.7	0.003
Q92974	ARHGEF2	Rho guanine nucleotide exchange factor 2	2.7	0.003
Q15075	EEA1	Early endosome antigen 1	2.7	0.007
P06132	UROD	Uroporphyrinogen decarboxylase	2.7	0.012
Q14152	EIF3A	Eukaryotic translation initiation factor 3 subunit A	2.6	<0.001
Q6P2E9	EDC4	Enhancer of mRNA-decapping protein 4	2.6	<0.001
O75369	FLNB	Filamin-B	2.6	<0.001
Q96RP9	GFM1	Elongation factor G, mitochondrial	2.6	<0.001
Q9NR12	PDLIM7	PDZ and LIM domain protein 7	2.6	<0.001
A0A3B3IRP5	CDC73	Parafibromin	2.6	<0.001
P78344	EIF4G2	Eukaryotic translation initiation factor 4 gamma 2	2.6	<0.001
F8WB06	ATXN2	Ataxin-2	2.6	<0.001
P49207	RPL34	60S ribosomal protein L34	2.6	<0.001
P48634	PRRC2A	Protein PRRC2A	2.6	<0.001
P42704	LRPPRC	Leucine-rich PPR motif-containing protein, mitochondrial	2.6	0.001
P33991	MCM4	DNA replication licensing factor MCM4	2.6	0.001
E7ETK0	RPS24	40S ribosomal protein S24	2.6	0.003
O60762	DPM1	Dolichol-phosphate mannosyltransferase subunit 1	2.6	0.003
P29144	TPP2	Tripeptidyl-peptidase 2	2.6	0.003
P27694	RPA1	Replication protein A 70 kDa DNA-binding subunit	2.6	0.003
Q92615	LARP4B	La-related protein 4B	2.6	0.004
P13807	GYS1	Glycogen [starch] synthase, muscle	2.6	0.004
P51452	DUSP3	Dual specificity protein phosphatase 3	2.6	0.004
Q6P587	FAHD1	Acylpyruvase FAHD1, mitochondrial	2.6	0.005
O76021	RSL1D1	Ribosomal L1 domain-containing protein 1	2.6	0.008
Q9H840	GEMIN7	Gem-associated protein 7	2.6	0.010
O00425	IGF2BP3	Insulin-like growth factor 2 mRNA-binding protein 3	2.5	<0.001
F8W8I6	TIA1	Nucleolysin TIA-1 isoform p40	2.5	<0.001
A0A087WTZ5	UBXN1	UBX domain-containing protein 1	2.5	<0.001
P04637	TP53	Cellular tumor antigen p53	2.5	<0.001
P61160	ACTR2	Actin-related protein 2	2.5	<0.001
Q9UHV9	PFDN2	Prefoldin subunit 2	2.5	<0.001
Q8IWZ3	ANKHD1	Ankyrin repeat and KH domain-containing protein 1	2.5	<0.001
P00568	AK1	Adenylate kinase isoenzyme 1	2.5	<0.001
A0A087WVM4	MTHFD1L	Formyltetrahydrofolate synthetase	2.5	<0.001
M0R0F0	RPS5	40S ribosomal protein S5	2.5	<0.001
Q15042	RAB3GAP1	Rab3 GTPase-activating protein catalytic subunit	2.5	<0.001
P61081	UBE2M	NEDD8-conjugating enzyme Ubc12	2.5	<0.001
A0A0U1RRM6	ENAH	Protein enabled homolog	2.5	<0.001
P16615	ATP2A2	Sarcoplasmic/endoplasmic reticulum calcium ATPase 2	2.5	<0.001
P27824	CANX	Calnexin	2.5	0.001
P78316	NOP14	Nucleolar protein 14	2.5	0.002
P52788	SMS	Spermine synthase	2.5	0.003
P37108	SRP14	Signal recognition particle 14 kDa protein	2.5	0.003
Q6IAA8	LAMTOR1	Ragulator complex protein LAMTOR1	2.5	0.004
P08243	ASNS	Asparagine synthetase [glutamine-hydrolyzing]	2.5	0.005
A0A087X1A5	STAU1	Double-stranded RNA-binding protein Staufen homolog 1	2.5	0.005
Q8WUH6	TMEM263	Transmembrane protein 263	2.5	0.005
Q8TCG1	CIP2A	Protein CIP2A	2.5	0.006
Q9Y5Y2	NUBP2	Cytosolic Fe-S cluster assembly factor NUBP2	2.5	0.007
Q86U42	PABPN1	Polyadenylate-binding protein 2	2.5	0.007
O15446	POLR1G	DNA-directed RNA polymerase I subunit RPA34	2.5	0.008
Q8TAT6	NPLOC4	Nuclear protein localization protein 4 homolog	2.5	0.008
P07196	NEFL	Neurofilament light polypeptide	2.5	0.008
Q9Y5K6	CD2AP	CD2-associated protein	2.5	0.008
P19105	MYL12A	Myosin regulatory light chain 12A	2.5	0.012
E9PF10	NUP155	Nuclear pore complex protein Nup155	2.5	0.012
Q96J01	THOC3	THO complex subunit 3	2.5	0.015
Q14690	PDCD11	Protein RRP5 homolog	2.5	0.015
P61758	VBP1	Prefoldin subunit 3	2.5	0.015
P47897	QARS1	Glutamine--tRNA ligase	2.4	<0.001
O95782	AP2A1	AP-2 complex subunit alpha-1	2.4	<0.001
Q02252	ALDH6A1	Methylmalonate-semialdehyde dehydrogenase [acylating], mitochondrial	2.4	<0.001
Q14157	UBAP2L	Ubiquitin-associated protein 2-like	2.4	<0.001
B1AK87	CAPZB	F-actin-capping protein subunit beta	2.4	<0.001
Q14151	SAFB2	Scaffold attachment factor B2	2.4	<0.001
B7Z7F3	RANBP3	Ran-binding protein 3	2.4	0.001
P19623	SRM	Spermidine synthase	2.4	0.001
Q9H9A6	LRRC40	Leucine-rich repeat-containing protein 40	2.4	0.001
Q96PK6	RBM14	RNA-binding protein 14	2.4	0.001
P48735	IDH2	Isocitrate dehydrogenase [NADP], mitochondrial	2.4	0.002
P39748	FEN1	Flap endonuclease 1	2.4	0.003
P49841	GSK3B	Glycogen synthase kinase-3 beta	2.4	0.006
Q71UI9	H2AZ2	Histone H2A.V	2.4	0.006
A0A0C4DGX4	CUL1	Cullin-1	2.4	0.007
F5H6E2	MYO1C	Unconventional myosin-Ic	2.4	0.007
A0A2R8Y804	CTNNB1	Catenin beta-1	2.4	0.008
Q8WWH5	TRUB1	Probable tRNA pseudouridine synthase 1	2.4	0.008
Q07021	C1QBP	Complement component 1 Q subcomponent-binding protein, mitochondrial	2.4	0.008
Q16698	DECR1	2,4-dienoyl-CoA reductase, mitochondrial	2.4	0.009
P40939	HADHA	Trifunctional enzyme subunit alpha, mitochondrial	2.4	0.009
O14497	ARID1A	AT-rich interactive domain-containing protein 1A	2.4	0.010
G3V1C3	API5	Apoptosis inhibitor 5	2.4	0.011
O95373	IPO7	Importin-7	2.4	0.011
A0A3B3ISG5	IDE	Insulin-degrading enzyme	2.4	0.012
Q16204	CCDC6	Coiled-coil domain-containing protein 6	2.4	0.024
Q8N1G4	LRRC47	Leucine-rich repeat-containing protein 47	2.3	<0.001
O15160	POLR1C	DNA-directed RNA polymerases I and III subunit RPAC1	2.3	<0.001
Q99543	DNAJC2	DnaJ homolog subfamily C member 2	2.3	<0.001
P19367	HK1	Hexokinase-1	2.3	<0.001
Q07666	KHDRBS1	KH domain-containing, RNA-binding, signal transduction-associated protein 1	2.3	<0.001
Q9BSH4	TACO1	Translational activator of cytochrome c oxidase 1	2.3	<0.001
Q5TDH0	DDI2	Protein DDI1 homolog 2	2.3	<0.001
B5MCF9	PES1	Pescadillo homolog	2.3	<0.001
P46776	RPL27A	60S ribosomal protein L27a	2.3	<0.001
O75569	PRKRA	Interferon-inducible double-stranded RNA-dependent protein kinase activator A	2.3	<0.001
P13861	PRKAR2A	cAMP-dependent protein kinase type II-alpha regulatory subunit	2.3	<0.001
Q6Y7W6	GIGYF2	GRB10-interacting GYF protein 2	2.3	<0.001
O43447	PPIH	Peptidyl-prolyl cis-trans isomerase H	2.3	0.001
P43686	PSMC4	26S proteasome regulatory subunit 6B	2.3	0.001
Q16352	INA	Alpha-internexin	2.3	0.001
Q96D71	REPS1	RalBP1-associated Eps domain-containing protein 1	2.3	0.002
P42765	ACAA2	3-ketoacyl-CoA thiolase, mitochondrial	2.3	0.002
Q99959	PKP2	Plakophilin-2	2.3	0.002
O00399	DCTN6	Dynactin subunit 6	2.3	0.002
Q9Y3I0	RTCB	RNA-splicing ligase RtcB homolog	2.3	0.002
Q9Y2L1	DIS3	Exosome complex exonuclease RRP44	2.3	0.004
O75592	MYCBP2	E3 ubiquitin-protein ligase MYCBP2	2.3	0.004
P09110	ACAA1	3-ketoacyl-CoA thiolase, peroxisomal	2.3	0.008
P38432	COIL	Coilin	2.3	0.008
O95747	OXSR1	Serine/threonine-protein kinase OSR1	2.3	0.008
Q5HYI8	RABL3	Rab-like protein 3	2.3	0.009
P22626	HNRNPA2B1	Heterogeneous nuclear ribonucleoproteins A2/B1	2.3	0.010
P62318	SNRPD3	Small nuclear ribonucleoprotein Sm D3	2.3	0.011
Q9BXP5	SRRT	Serrate RNA effector molecule homolog	2.3	0.011
P50570	DNM2	Dynamin-2	2.3	0.013
Q9BQ39	DDX50	ATP-dependent RNA helicase DDX50	2.3	0.014
O43143	DHX15	Pre-mRNA-splicing factor ATP-dependent RNA helicase DHX15	2.3	0.019
Q13573	SNW1	SNW domain-containing protein 1	2.3	0.039
Q5JTZ9	AARS2	Alanine--tRNA ligase, mitochondrial	2.2	<0.001
P27144	AK4	Adenylate kinase 4, mitochondrial	2.2	<0.001
P22314	UBA1	Ubiquitin-like modifier-activating enzyme 1	2.2	<0.001
Q13509	TUBB3	Tubulin beta-3 chain	2.2	<0.001
P54578	USP14	Ubiquitin carboxyl-terminal hydrolase 14	2.2	<0.001
Q13136	PPFIA1	Liprin-alpha-1	2.2	<0.001
P30520	ADSS2	Adenylosuccinate synthetase isozyme 2	2.2	<0.001
Q99497	PARK7	Parkinson disease protein 7	2.2	<0.001
P37802	TAGLN2	Transgelin-2	2.2	<0.001
Q71RC2	LARP4	La-related protein 4	2.2	<0.001
P30153	PPP2R1A	Serine/threonine-protein phosphatase 2A 65 kDa regulatory subunit A alpha isoform	2.2	<0.001
P51114	FXR1	Fragile X mental retardation syndrome-related protein 1	2.2	<0.001
D6RBD7	EEF1E1	Eukaryotic translation elongation factor 1 epsilon-1	2.2	<0.001
P08754	GNAI3	Guanine nucleotide-binding protein G(i) subunit alpha	2.2	0.001
Q02543	RPL18A	60S ribosomal protein L18a	2.2	0.001
Q9Y6Y8	SEC23IP	SEC23-interacting protein	2.2	0.001
P28838	LAP3	Cytosol aminopeptidase	2.2	0.001
Q9NSK0	KLC4	Kinesin light chain 4	2.2	0.001
M0QXL5	FBL	rRNA 2'-O-methyltransferase fibrillarin	2.2	0.002
P35268	RPL22	60S ribosomal protein L22	2.2	0.002
Q08J23	NSUN2	RNA cytosine C(5)-methyltransferase NSUN2	2.2	0.002
Q13464	ROCK1	Rho-associated protein kinase 1	2.2	0.004
H0YDU8	PPP5C	Serine/threonine-protein phosphatase	2.2	0.010
Q92973	TNPO1	Transportin-1	2.2	0.012
Q15084	PDIA6	Protein disulfide-isomerase A6	2.2	0.012
Q15750	TAB1	TGF-beta-activated kinase 1 and MAP3K7-binding protein 1	2.2	0.012
O43660	PLRG1	Pleiotropic regulator 1	2.2	0.013
Q01813	PFKP	ATP-dependent 6-phosphofructokinase, platelet type	2.2	0.015
Q9BUQ8	DDX23	Probable ATP-dependent RNA helicase DDX23	2.2	0.016
Q13257	MAD2L1	Mitotic spindle assembly checkpoint protein MAD2A	2.2	0.019
P82921	MRPS21	28S ribosomal protein S21, mitochondrial	2.2	0.020
Q7Z434	MAVS	Mitochondrial antiviral-signaling protein	2.2	0.024
P31323	PRKAR2B	cAMP-dependent protein kinase type II-beta regulatory subunit	2.2	0.050
Q52LJ0	FAM98B	Protein FAM98B	2.1	<0.001
A0A1C7CYX9	DPYSL2	Dihydropyrimidinase-related protein 2	2.1	<0.001
Q14247	CTTN	Src substrate cortactin	2.1	<0.001
P50502	ST13	Hsc70-interacting protein	2.1	<0.001
P55795	HNRNPH2	Heterogeneous nuclear ribonucleoprotein H2	2.1	<0.001
Q8WXF1	PSPC1	Paraspeckle component 1	2.1	<0.001
Q09666	AHNAK	Neuroblast differentiation-associated protein AHNAK	2.1	<0.001
Q9BQ69	MACROD1	ADP-ribose glycohydrolase MACROD1	2.1	<0.001
B4DDF4	CNN2	Calponin	2.1	0.001
Q5T7U1	GTF3C5	General transcription factor 3C polypeptide 5	2.1	0.001
P46459	NSF	Vesicle-fusing ATPase	2.1	0.001
O76003	GLRX3	Glutaredoxin-3	2.1	0.001
A0A0A0MRM9	NOLC1	Nucleolar and coiled-body phosphoprotein 1	2.1	0.003
P00519	ABL1	Tyrosine-protein kinase ABL1	2.1	0.003
P49959	MRE11	Double-strand break repair protein MRE11	2.1	0.004
P51570	GALK1	Galactokinase	2.1	0.004
P46777	RPL5	60S ribosomal protein L5	2.1	0.005
P11388	TOP2A	DNA topoisomerase 2-alpha	2.1	0.009
P62906	RPL10A	60S ribosomal protein L10a	2.1	0.012
Q9NXH9	TRMT1	tRNA	2.1	0.012
Q9BXJ9	NAA15	N-alpha-acetyltransferase 15, NatA auxiliary subunit	2.1	0.017
B4DLN1	B4DLN1	cDNA FLJ60124, highly similar to Mitochondrial dicarboxylate carrier	2.1	0.017
Q9BT25	HAUS8	HAUS augmin-like complex subunit 8	2.1	0.018
O14654	IRS4	Insulin receptor substrate 4	2.1	0.021
O43707	ACTN4	Alpha-actinin-4	2.1	0.026
P18031	PTPN1	Tyrosine-protein phosphatase non-receptor type 1	2.1	0.027
A0A3B3IUD2	MSTO1	Protein misato homolog 1	2.1	0.036
Q53H96	PYCR3	Pyrroline-5-carboxylate reductase 3	2.1	0.042
P19022	CDH2	Cadherin-2	2.1	0.043
Q8WWK9	CKAP2	Cytoskeleton-associated protein 2	2.0	<0.001
Q9NZI8	IGF2BP1	Insulin-like growth factor 2 mRNA-binding protein 1	2.0	<0.001
P25490	YY1	Transcriptional repressor protein YY1	2.0	<0.001
P25325	MPST	3-mercaptopyruvate sulfurtransferase	2.0	<0.001
Q96AG4	LRRC59	Leucine-rich repeat-containing protein 59	2.0	<0.001
Q8WYP5	AHCTF1	Protein ELYS	2.0	<0.001
X1WI28	RPL10	60S ribosomal protein L10	2.0	<0.001
E7EPN9	PRRC2C	Protein PRRC2C	2.0	<0.001
P13804	ETFA	Electron transfer flavoprotein subunit alpha, mitochondrial	2.0	<0.001
E7EVA0	MAP4	Microtubule-associated protein	2.0	<0.001
P26640	VARS1	Valine--tRNA ligase	2.0	<0.001
P62280	RPS11	40S ribosomal protein S11	2.0	<0.001
P31942	HNRNPH3	Heterogeneous nuclear ribonucleoprotein H3	2.0	<0.001
Q14126	DSG2	Desmoglein-2	2.0	<0.001
Q9H6T3	RPAP3	RNA polymerase II-associated protein 3	2.0	0.001
P46063	RECQL	ATP-dependent DNA helicase Q1	2.0	0.001
Q9UGI8	TES	Testin	2.0	0.001
A0A0G2JNZ2	SCRIB	Protein scribble homolog	2.0	0.001
P24534	EEF1B2	Elongation factor 1-beta	2.0	0.001
Q86UK7	ZNF598	E3 ubiquitin-protein ligase ZNF598	2.0	0.001
P62258	YWHAE	14-3-3 protein epsilon	2.0	0.001
O75152	ZC3H11A	Zinc finger CCCH domain-containing protein 11A	2.0	0.002
O15305	PMM2	Phosphomannomutase 2	2.0	0.002
Q86Y56	DNAAF5	Dynein assembly factor 5, axonemal	2.0	0.002
P55265	ADAR	Double-stranded RNA-specific adenosine deaminase	2.0	0.002
J3QR09	RPL19	Ribosomal protein L19	2.0	0.002
Q99569	PKP4	Plakophilin-4	2.0	0.003
Q14258	TRIM25	E3 ubiquitin/ISG15 ligase TRIM25	2.0	0.004
A5YKK6	CNOT1	CCR4-NOT transcription complex subunit 1	2.0	0.008
J3KTA4	DDX5	DEAD box protein 5	2.0	0.010
Q9GZS1	POLR1E	DNA-directed RNA polymerase I subunit RPA49	2.0	0.012
P35579	MYH9	Myosin-9	2.0	0.018
O14744	PRMT5	Protein arginine N-methyltransferase 5	2.0	0.018
Q9Y2Z4	YARS2	Tyrosine--tRNA ligase, mitochondrial	2.0	0.019
Q86VS8	HOOK3	Protein Hook homolog 3	2.0	0.020
Q9UGV2	NDRG3	Protein NDRG3	2.0	0.020
F5GWT4	WNK1	Non-specific serine/threonine protein kinase	2.0	0.020
D6R938	CAMK2D	Calcium/calmodulin-dependent protein kinase	2.0	0.021
Q16822	PCK2	Phosphoenolpyruvate carboxykinase [GTP], mitochondrial	2.0	0.021
Q9GZL7	WDR12	Ribosome biogenesis protein WDR12	2.0	0.023
Q8NCA5	FAM98A	Protein FAM98A	2.0	0.024
Q92665	MRPS31	28S ribosomal protein S31, mitochondrial	2.0	0.025
A0A0A6YYL4	CORO7-PAM16	Coronin	2.0	0.025
Q13625	TP53BP2	Apoptosis-stimulating of p53 protein 2	2.0	0.026
Q96AC1	FERMT2	Fermitin family homolog 2	2.0	0.028
Q15031	LARS2	Probable leucine--tRNA ligase, mitochondrial	2.0	0.029
Q9BTY7	HGH1	Protein HGH1 homolog	2.0	0.032
Q5UIP0	RIF1	Telomere-associated protein RIF1	2.0	0.032
Q96DH6	MSI2	RNA-binding protein Musashi homolog 2	2.0	0.036
Q9HAV4	XPO5	Exportin-5	2.0	0.037
Q9BYG3	NIFK	MKI67 FHA domain-interacting nucleolar phosphoprotein	2.0	0.039
P15880	RPS2	40S ribosomal protein S2	1.9	<0.001
P52948	NUP98	Nuclear pore complex protein Nup98-Nup96	1.9	<0.001
A0A087WXM6	RPL17	60S ribosomal protein L17	1.9	<0.001
O43159	RRP8	Ribosomal RNA-processing protein 8	1.9	<0.001
Q9Y2Z0	SUGT1	Protein SGT1 homolog	1.9	<0.001
P43246	MSH2	DNA mismatch repair protein Msh2	1.9	<0.001
Q16527	CSRP2	Cysteine and glycine-rich protein 2	1.9	<0.001
P46939	UTRN	Utrophin	1.9	<0.001
Q9BV20	MRI1	Methylthioribose-1-phosphate isomerase	1.9	<0.001
Q7RTV0	PHF5A	PHD finger-like domain-containing protein 5A	1.9	<0.001
Q8ND83	SLAIN1	SLAIN motif-containing protein 1	1.9	<0.001
Q96MU7	YTHDC1	YTH domain-containing protein 1	1.9	0.001
P18615	NELFE	Negative elongation factor E	1.9	0.001
P52701	MSH6	DNA mismatch repair protein Msh6	1.9	0.001
Q9UBT2	UBA2	SUMO-activating enzyme subunit 2	1.9	0.001
O60684	KPNA6	Importin subunit alpha-7	1.9	0.002
Q8NCW5	NAXE	NAD(P)H-hydrate epimerase	1.9	0.003
Q9H0H5	RACGAP1	Rac GTPase-activating protein 1	1.9	0.005
A0A087X0K9	TJP1	Tight junction protein ZO-1	1.9	0.005
P50995	ANXA11	Annexin A11	1.9	0.005
Q9BYN8	MRPS26	28S ribosomal protein S26, mitochondrial	1.9	0.007
O75436	VPS26A	Vacuolar protein sorting-associated protein 26A	1.9	0.007
Q12849	GRSF1	G-rich sequence factor 1	1.9	0.010
O14974	PPP1R12A	Protein phosphatase 1 regulatory subunit 12A	1.9	0.011
Q9BVP2	GNL3	Guanine nucleotide-binding protein-like 3	1.9	0.011
Q9BQ67	GRWD1	Glutamate-rich WD repeat-containing protein 1	1.9	0.012
Q13144	EIF2B5	Translation initiation factor eIF-2B subunit epsilon	1.9	0.019
Q86WJ1	CHD1L	Chromodomain-helicase-DNA-binding protein 1-like	1.9	0.020
O60701	UGDH	UDP-glucose 6-dehydrogenase	1.9	0.022
Q9BR76	CORO1B	Coronin-1B	1.9	0.025
A0A087WY71	AP2M1	AP-2 complex subunit mu	1.9	0.028
Q9ULT8	HECTD1	E3 ubiquitin-protein ligase HECTD1	1.9	0.029
P50542	PEX5	Peroxisomal targeting signal 1 receptor	1.9	0.029
Q5TFE4	NT5DC1	5'-nucleotidase domain-containing protein 1	1.9	0.030
O00487	PSMD14	26S proteasome non-ATPase regulatory subunit 14	1.9	0.030
Q8WX93	PALLD	Palladin	1.9	0.031
Q13618	CUL3	Cullin-3	1.9	0.031
O15020	SPTBN2	Spectrin beta chain, non-erythrocytic 2	1.9	0.032
O00267	SUPT5H	Transcription elongation factor SPT5	1.9	0.033
Q9BVJ6	UTP14A	U3 small nucleolar RNA-associated protein 14 homolog A	1.9	0.034
O95785	WIZ	Protein Wiz	1.9	0.035
Q7L0Y3	TRMT10C	tRNA methyltransferase 10 homolog C	1.9	0.036
Q96FV9	THOC1	THO complex subunit 1	1.9	0.038
O14617	AP3D1	AP-3 complex subunit delta-1	1.9	0.038
Q14966	ZNF638	Zinc finger protein 638	1.9	0.038
Q12948	FOXC1	Forkhead box protein C1	1.9	0.040
Q2TAL8	QRICH1	Glutamine-rich protein 1	1.9	0.040
Q9NP97	DYNLRB1	Dynein light chain roadblock-type 1	1.9	0.041
Q13363	CTBP1	C-terminal-binding protein 1	1.9	0.048
O75643	SNRNP200	U5 small nuclear ribonucleoprotein 200 kDa helicase	1.9	0.049
P49368	CCT3	T-complex protein 1 subunit gamma	1.8	<0.001
Q5T6F2	UBAP2	Ubiquitin-associated protein 2	1.8	<0.001
Q10567	AP1B1	AP-1 complex subunit beta-1	1.8	<0.001
P55060	CSE1L	Exportin-2	1.8	<0.001
O75792	RNASEH2A	Ribonuclease H2 subunit A	1.8	<0.001
Q9Y490	TLN1	Talin-1	1.8	<0.001
P30084	ECHS1	Enoyl-CoA hydratase, mitochondrial	1.8	<0.001
A0A1B0GWF2	STXBP1	Syntaxin-binding protein 1	1.8	<0.001
A0A0A0MRM8	MYO6	Unconventional myosin-6	1.8	<0.001
Q6YN16	HSDL2	Hydroxysteroid dehydrogenase-like protein 2	1.8	<0.001
Q16658	FSCN1	Fascin	1.8	<0.001
P46778	RPL21	60S ribosomal protein L21	1.8	<0.001
Q13451	FKBP5	Peptidyl-prolyl cis-trans isomerase FKBP5	1.8	<0.001
Q8WUM4	PDCD6IP	Programmed cell death 6-interacting protein	1.8	<0.001
A0A1W2PPZ5	TCEA1	Transcription elongation factor A protein 1	1.8	<0.001
O95239	KIF4A	Chromosome-associated kinesin KIF4A	1.8	0.001
K4DI93	CUL4B	Cullin 4B, isoform CRA_e	1.8	0.001
Q9BRX2	PELO	Protein pelota homolog	1.8	0.001
O43390	HNRNPR	Heterogeneous nuclear ribonucleoprotein R	1.8	0.001
Q14166	TTLL12	Tubulin--tyrosine ligase-like protein 12	1.8	0.001
P61313	RPL15	60S ribosomal protein L15	1.8	0.001
P12004	PCNA	Proliferating cell nuclear antigen	1.8	0.001
H7C5E4	XRN1	5'-3' exoribonuclease 1	1.8	0.001
Q13185	CBX3	Chromobox protein homolog 3	1.8	0.001
E9PDU5	WDR6	WD repeat-containing protein 6	1.8	0.001
O95336	PGLS	6-phosphogluconolactonase	1.8	0.001
Q9NZB2	FAM120A	Constitutive coactivator of PPAR-gamma-like protein 1	1.8	0.002
Q9BUF5	TUBB6	Tubulin beta-6 chain	1.8	0.002
P26373	RPL13	60S ribosomal protein L13	1.8	0.002
Q08752	PPID	Peptidyl-prolyl cis-trans isomerase D	1.8	0.002
Q9UPN7	PPP6R1	Serine/threonine-protein phosphatase 6 regulatory subunit 1	1.8	0.003
F8W0J6	NAP1L1	Nucleosome assembly protein 1-like 1	1.8	0.003
P26368	U2AF2	Splicing factor U2AF 65 kDa subunit	1.8	0.003
Q9P0K7	RAI14	Ankycorbin	1.8	0.004
P52272	HNRNPM	Heterogeneous nuclear ribonucleoprotein M	1.8	0.004
Q9UHB9	SRP68	Signal recognition particle subunit SRP68	1.8	0.005
O95861	BPNT1	3'(2'),5'-bisphosphate nucleotidase 1	1.8	0.005
Q13595	TRA2A	Transformer-2 protein homolog alpha	1.8	0.005
P12277	CKB	Creatine kinase B-type	1.8	0.006
Q9Y6G9	DYNC1LI1	Cytoplasmic dynein 1 light intermediate chain 1	1.8	0.006
Q9BSJ8	ESYT1	Extended synaptotagmin-1	1.8	0.006
P53597	SUCLG1	Succinate--CoA ligase [ADP/GDP-forming] subunit alpha, mitochondrial	1.8	0.006
O75131	CPNE3	Copine-3	1.8	0.006
P55010	EIF5	Eukaryotic translation initiation factor 5	1.8	0.006
P52732	KIF11	Kinesin-like protein KIF11	1.8	0.007
Q15021	NCAPD2	Condensin complex subunit 1	1.8	0.009
Q14161	GIT2	ARF GTPase-activating protein GIT2	1.8	0.009
Q8IY81	FTSJ3	pre-rRNA 2'-O-ribose RNA methyltransferase FTSJ3	1.8	0.014
Q92616	GCN1	eIF-2-alpha kinase activator GCN1	1.8	0.015
O95347	SMC2	Structural maintenance of chromosomes protein 2	1.8	0.022
P25205	MCM3	DNA replication licensing factor MCM3	1.8	0.026
P53582	METAP1	Methionine aminopeptidase 1	1.8	0.027
P30154	PPP2R1B	Serine/threonine-protein phosphatase 2A 65 kDa regulatory subunit A beta isoform	1.8	0.032
Q92878	RAD50	DNA repair protein RAD50	1.8	0.032
P54920	NAPA	Alpha-soluble NSF attachment protein	1.8	0.034
Q92797	SYMPK	Symplekin	1.8	0.035
Q05048	CSTF1	Cleavage stimulation factor subunit 1	1.8	0.035
O60832	DKC1	H/ACA ribonucleoprotein complex subunit DKC1	1.8	0.036
Q06210	GFPT1	Glutamine--fructose-6-phosphate aminotransferase [isomerizing] 1	1.8	0.036
Q53EL6	PDCD4	Programmed cell death protein 4	1.8	0.043
Q9UQN3	CHMP2B	Charged multivesicular body protein 2b	1.8	0.043
E9PKP7	UBTF	Nucleolar transcription factor 1	1.8	0.046
P17252	PRKCA	Protein kinase C alpha type	1.8	0.049
A0A087WY55	VTA1	Chromosome 6 open reading frame 55, isoform CRA_b	1.7	<0.001
Q9UPQ9	TNRC6B	Trinucleotide repeat-containing gene 6B protein	1.7	<0.001
P49321	NASP	Nuclear autoantigenic sperm protein	1.7	<0.001
Q8TD19	NEK9	Serine/threonine-protein kinase Nek9	1.7	<0.001
Q86YP4	GATAD2A	Transcriptional repressor p66-alpha	1.7	<0.001
Q9H7E9	C8orf33	UPF0488 protein C8orf33	1.7	<0.001
P35221	CTNNA1	Catenin alpha-1	1.7	<0.001
Q9UQ80	PA2G4	Proliferation-associated protein 2G4	1.7	<0.001
Q7Z4H3	HDDC2	5'-deoxynucleotidase HDDC2	1.7	<0.001
P60228	EIF3E	Eukaryotic translation initiation factor 3 subunit E	1.7	0.001
Q15003	NCAPH	Condensin complex subunit 2	1.7	0.001
P62333	PSMC6	26S proteasome regulatory subunit 10B	1.7	0.001
Q9Y5A9	YTHDF2	YTH domain-containing family protein 2	1.7	0.001
P60660	MYL6	Myosin light polypeptide 6	1.7	0.001
Q15785	TOMM34	Mitochondrial import receptor subunit TOM34	1.7	0.001
Q8N163	CCAR2	Cell cycle and apoptosis regulator protein 2	1.7	0.002
C9JRJ5	LIMD1	LIM domain-containing protein 1	1.7	0.002
Q96GD0	PDXP	Pyridoxal phosphate phosphatase	1.7	0.003
P33176	KIF5B	Kinesin-1 heavy chain	1.7	0.004
P35249	RFC4	Replication factor C subunit 4	1.7	0.004
Q9NYJ8	TAB2	TGF-beta-activated kinase 1 and MAP3K7-binding protein 2	1.7	0.004
Q96F86	EDC3	Enhancer of mRNA-decapping protein 3	1.7	0.005
O60783	MRPS14	28S ribosomal protein S14, mitochondrial	1.7	0.006
P56192	MARS1	Methionine--tRNA ligase, cytoplasmic	1.7	0.006
Q9NR45	NANS	Sialic acid synthase	1.7	0.007
Q9Y237	PIN4	Peptidyl-prolyl cis-trans isomerase NIMA-interacting 4	1.7	0.007
Q00610	CLTC	Clathrin heavy chain 1	1.7	0.007
P62249	RPS16	40S ribosomal protein S16	1.7	0.007
Q1KMD3	HNRNPUL2	Heterogeneous nuclear ribonucleoprotein U-like protein 2	1.7	0.007
Q13283	G3BP1	Ras GTPase-activating protein-binding protein 1	1.7	0.010
P25685	DNAJB1	DnaJ homolog subfamily B member 1	1.7	0.013
Q9H3U1	UNC45A	Protein unc-45 homolog A	1.7	0.015
O15226	NKRF	NF-kappa-B-repressing factor	1.7	0.018
P35659	DEK	Protein DEK	1.7	0.018
Q8WTT2	NOC3L	Nucleolar complex protein 3 homolog	1.7	0.027
A0A087WVZ9	POLR2E	DNA-directed RNA polymerase II subunit E	1.7	0.027
Q9Y6A5	TACC3	Transforming acidic coiled-coil-containing protein 3	1.7	0.032
Q9H814	PHAX	Phosphorylated adapter RNA export protein	1.7	0.034
Q96G46	DUS3L	tRNA-dihydrouridine(47) synthase [NAD(P)(+)]-like	1.7	0.037
E9PB90	HK2	Hexokinase	1.7	0.040
Q86VP6	CAND1	Cullin-associated NEDD8-dissociated protein 1	1.7	0.042
Q7Z478	DHX29	ATP-dependent RNA helicase DHX29	1.7	0.043
Q4KMP7	TBC1D10B	TBC1 domain family member 10B	1.7	0.044
P29558	RBMS1	RNA-binding motif, single-stranded-interacting protein 1	1.7	0.044
Q9H845	ACAD9	Complex I assembly factor ACAD9, mitochondrial	1.7	0.045
Q9HCD5	NCOA5	Nuclear receptor coactivator 5	1.7	0.048
P78347	GTF2I	General transcription factor II-I	1.7	0.049
Q5JSZ5	PRRC2B	Protein PRRC2B	1.6	<0.001
P61158	ACTR3	Actin-related protein 3	1.6	<0.001
Q9NSD9	FARSB	Phenylalanine--tRNA ligase beta subunit	1.6	<0.001
Q00796	SORD	Sorbitol dehydrogenase	1.6	<0.001
Q14974	KPNB1	Importin subunit beta-1	1.6	<0.001
P30566	ADSL	Adenylosuccinate lyase	1.6	0.001
P38117	ETFB	Electron transfer flavoprotein subunit beta	1.6	0.001
K7EJL1	AP1M1	AP-1 complex subunit mu-1	1.6	0.001
P42166	TMPO	Lamina-associated polypeptide 2, isoform alpha	1.6	0.001
O15371	EIF3D	Eukaryotic translation initiation factor 3 subunit D	1.6	0.001
P33993	MCM7	DNA replication licensing factor MCM7	1.6	0.001
P46779	RPL28	60S ribosomal protein L28	1.6	0.002
O43264	ZW10	Centromere/kinetochore protein zw10 homolog	1.6	0.002
O00170	AIP	AH receptor-interacting protein	1.6	0.002
Q9ULX3	NOB1	RNA-binding protein NOB1	1.6	0.002
A0A0G2JH68	DIAPH1	Protein diaphanous homolog 1	1.6	0.002
M0R3C3	TECR	Very-long-chain enoyl-CoA reductase	1.6	0.002
Q9BW92	TARS2	Threonine--tRNA ligase, mitochondrial	1.6	0.003
Q9C0C9	UBE2O	(E3-independent) E2 ubiquitin-conjugating enzyme	1.6	0.004
Q15637	SF1	Splicing factor 1	1.6	0.004
P52294	KPNA1	Importin subunit alpha-5	1.6	0.004
Q15424	SAFB	Scaffold attachment factor B1	1.6	0.005
A6NDG6	PGP	Glycerol-3-phosphate phosphatase	1.6	0.005
P60174	TPI1	Triosephosphate isomerase	1.6	0.006
B4E1N1	ARMC6	Armadillo repeat-containing protein 6	1.6	0.006
Q15417	CNN3	Calponin-3	1.6	0.008
Q9UI30	TRMT112	Multifunctional methyltransferase subunit TRM112-like protein	1.6	0.008
Q9Y450	HBS1L	HBS1-like protein	1.6	0.009
P43490	NAMPT	Nicotinamide phosphoribosyltransferase	1.6	0.009
Q9BZE4	GTPBP4	Nucleolar GTP-binding protein 1	1.6	0.010
A0A087X2D5	MRPL45	39S ribosomal protein L45, mitochondrial	1.6	0.011
Q9H2P0	ADNP	Activity-dependent neuroprotector homeobox protein	1.6	0.011
Q13247	SRSF6	Serine/arginine-rich splicing factor 6	1.6	0.015
O00571	DDX3X	ATP-dependent RNA helicase DDX3X	1.6	0.015
Q9NW13	RBM28	RNA-binding protein 28	1.6	0.015
Q15813	TBCE	Tubulin-specific chaperone E	1.6	0.015
P19338	NCL	Nucleolin	1.6	0.017
O00231	PSMD11	26S proteasome non-ATPase regulatory subunit 11	1.6	0.018
Q8NB90	SPATA5	ATPase family protein 2 homolog	1.6	0.021
F8W8R3	POLD2	DNA polymerase delta subunit 2	1.6	0.024
O43719	HTATSF1	HIV Tat-specific factor 1	1.6	0.026
Q9BY77	POLDIP3	Polymerase delta-interacting protein 3	1.6	0.027
Q9H0E2	TOLLIP	Toll-interacting protein	1.6	0.044
O75150	RNF40	E3 ubiquitin-protein ligase BRE1B	1.6	0.045
Q8IWB7	WDFY1	WD repeat and FYVE domain-containing protein 1	1.6	0.046
P22102	GART	Trifunctional purine biosynthetic protein adenosine-3 [Includes: Phosphoribosylamine--glycine ligase	1.5	<0.001
Q15233	NONO	Non-POU domain-containing octamer-binding protein	1.5	<0.001
Q14669	TRIP12	E3 ubiquitin-protein ligase TRIP12	1.5	<0.001
P31948	STIP1	Stress-induced-phosphoprotein 1	1.5	<0.001
P48643	CCT5	T-complex protein 1 subunit epsilon	1.5	<0.001
P29692	EEF1D	Elongation factor 1-delta	1.5	<0.001
Q02790	FKBP4	Peptidyl-prolyl cis-trans isomerase FKBP4	1.5	0.001
P61586	RHOA	Transforming protein RhoA	1.5	0.001
O43237	DYNC1LI2	Cytoplasmic dynein 1 light intermediate chain 2	1.5	0.001
P07814	EPRS1	Bifunctional glutamate/proline--tRNA ligase	1.5	0.001
O95816	BAG2	BAG family molecular chaperone regulator 2	1.5	0.001
Q9NPI6	DCP1A	mRNA-decapping enzyme 1A	1.5	0.002
Q9BZE9	ASPSCR1	Tether containing UBX domain for GLUT4	1.5	0.003
P63241	EIF5A	Eukaryotic translation initiation factor 5A-1	1.5	0.004
P41240	CSK	Tyrosine-protein kinase CSK	1.5	0.005
Q8IWX8	CHERP	Calcium homeostasis endoplasmic reticulum protein	1.5	0.006
O75534	CSDE1	Cold shock domain-containing protein E1	1.5	0.006
O95573	ACSL3	Long-chain-fatty-acid--CoA ligase 3	1.5	0.006
Q13428	TCOF1	Treacle protein	1.5	0.006
P78371	CCT2	T-complex protein 1 subunit beta	1.5	0.006
Q9NYZ3	GTSE1	G2 and S phase-expressed protein 1	1.5	0.007
Q8IYI6	EXOC8	Exocyst complex component 8	1.5	0.009
B4DY08	HNRNPC	Heterogeneous nuclear ribonucleoproteins C1/C2	1.5	0.009
Q9NQT5	EXOSC3	Exosome complex component RRP40	1.5	0.010
P50213	IDH3A	Isocitrate dehydrogenase [NAD] subunit alpha, mitochondrial	1.5	0.014
Q9UQ35	SRRM2	Serine/arginine repetitive matrix protein 2	1.5	0.016
O43395	PRPF3	U4/U6 small nuclear ribonucleoprotein Prp3	1.5	0.018
Q02878	RPL6	60S ribosomal protein L6	1.5	0.018
O00273	DFFA	DNA fragmentation factor subunit alpha	1.5	0.019
P63010	AP2B1	AP-2 complex subunit beta	1.5	0.023
Q9BWD1	ACAT2	Acetyl-CoA acetyltransferase, cytosolic	1.5	0.023
Q9H2U1	DHX36	ATP-dependent DNA/RNA helicase DHX36	1.5	0.024
O43252	PAPSS1	Bifunctional 3'-phosphoadenosine 5'-phosphosulfate synthase 1	1.5	0.028
P48507	GCLM	Glutamate--cysteine ligase regulatory subunit	1.5	0.032
Q9HC35	EML4	Echinoderm microtubule-associated protein-like 4	1.4	<0.001
P62633	CNBP	Cellular nucleic acid-binding protein	1.4	<0.001
Q8N0X7	SPART	Spartin	1.4	<0.001
P25786	PSMA1	Proteasome subunit alpha type-1	1.4	<0.001
P40227	CCT6A	T-complex protein 1 subunit zeta	1.4	<0.001
Q5JXI8	FHL1	Four and a half LIM domains protein 1	1.4	<0.001
Q86TB9	PATL1	Protein PAT1 homolog 1	1.4	<0.001
O94826	TOMM70	Mitochondrial import receptor subunit TOM70	1.4	0.001
Q99613	EIF3C	Eukaryotic translation initiation factor 3 subunit C	1.4	0.001
Q9UJU6	DBNL	Drebrin-like protein	1.4	0.001
Q96HC4	PDLIM5	PDZ and LIM domain protein 5	1.4	0.002
Q9UNY4	TTF2	Transcription termination factor 2	1.4	0.002
P33240	CSTF2	Cleavage stimulation factor subunit 2	1.4	0.002
E9PQV9	DCUN1D5	DCN1-like protein	1.4	0.003
P42696	RBM34	RNA-binding protein 34	1.4	0.004
Q9UHD1	CHORDC1	Cysteine and histidine-rich domain-containing protein 1	1.4	0.004
A0A3B3IRI2	CTPS1	CTP synthase	1.4	0.005
Q14C86	GAPVD1	GTPase-activating protein and VPS9 domain-containing protein 1	1.4	0.005
P62244	RPS15A	40S ribosomal protein S15a	1.4	0.006
Q12904	AIMP1	Aminoacyl tRNA synthase complex-interacting multifunctional protein 1	1.4	0.007
Q9NTI5	PDS5B	Sister chromatid cohesion protein PDS5 homolog B	1.4	0.008
Q9NSV4	DIAPH3	Protein diaphanous homolog 3	1.4	0.008
P49588	AARS1	Alanine--tRNA ligase, cytoplasmic	1.4	0.009
P46783	RPS10	40S ribosomal protein S10	1.4	0.009
A0A140T9R1	FLOT1	Flotillin	1.4	0.011
Q9UKM9	RALY	RNA-binding protein Raly	1.4	0.014
P51572	BCAP31	B-cell receptor-associated protein 31	1.4	0.015
O43290	SART1	U4/U6.U5 tri-snRNP-associated protein 1	1.4	0.015
O94906	PRPF6	Pre-mRNA-processing factor 6	1.4	0.017
Q9P2R3	ANKFY1	Rabankyrin-5	1.4	0.019
O00154	ACOT7	Cytosolic acyl coenzyme A thioester hydrolase	1.4	0.027
P27540	ARNT	Aryl hydrocarbon receptor nuclear translocator	1.4	0.028
P52597	HNRNPF	Heterogeneous nuclear ribonucleoprotein F	1.4	0.028
O95453	PARN	Poly(A)-specific ribonuclease PARN	1.4	0.030
C9J4Z3	RPL37A	60S ribosomal protein L37a	1.4	0.032
Q9UKD2	MRTO4	mRNA turnover protein 4 homolog	1.4	0.033
P18583	SON	Protein SON	1.4	0.035
Q92667	AKAP1	A-kinase anchor protein 1, mitochondrial	1.4	0.042
O14980	XPO1	Exportin-1	1.4	0.049
P62913	RPL11	60S ribosomal protein L11	1.3	<0.001
Q13045	FLII	Protein flightless-1 homolog	1.3	<0.001
Q9BTE6	AARSD1	Alanyl-tRNA editing protein Aarsd1	1.3	<0.001
F6T1Q0	PDE12	2',5'-phosphodiesterase 12	1.3	0.001
Q7LBC6	KDM3B	Lysine-specific demethylase 3B	1.3	0.001
P50991	CCT4	T-complex protein 1 subunit delta	1.3	0.001
Q15365	PCBP1	Poly(rC)-binding protein 1	1.3	0.001
Q2TAM5	RELA	RELA protein	1.3	0.002
Q14677	CLINT1	Clathrin interactor 1	1.3	0.002
P57772	EEFSEC	Selenocysteine-specific elongation factor	1.3	0.003
Q5QJE6	DNTTIP2	Deoxynucleotidyltransferase terminal-interacting protein 2	1.3	0.004
Q8IWS0	PHF6	PHD finger protein 6	1.3	0.004
P09525	ANXA4	Annexin A4	1.3	0.005
Q01469	FABP5	Fatty acid-binding protein 5	1.3	0.008
P62753	RPS6	40S ribosomal protein S6	1.3	0.009
Q9Y3S2	ZNF330	Zinc finger protein 330	1.3	0.011
Q9UNM6	PSMD13	26S proteasome non-ATPase regulatory subunit 13	1.3	0.012
Q8IZ83	ALDH16A1	Aldehyde dehydrogenase family 16 member A1	1.3	0.012
A0A1B0GV47	KIF21A	Kinesin-like protein KIF21A	1.3	0.012
C9JP00	MBNL1	Muscleblind-like protein 1	1.3	0.013
P62241	RPS8	40S ribosomal protein S8	1.3	0.013
P34897	SHMT2	Serine hydroxymethyltransferase, mitochondrial	1.3	0.013
A0A2R8Y880	RBBP6	E3 ubiquitin-protein ligase RBBP6	1.3	0.014
P49736	MCM2	DNA replication licensing factor MCM2	1.3	0.018
H0Y5D5	CIZ1	Cip1-interacting zinc finger protein	1.3	0.019
Q9NWH9	SLTM	SAFB-like transcription modulator	1.3	0.019
Q13838	DDX39B	Spliceosome RNA helicase DDX39B	1.3	0.019
Q8WWQ0	PHIP	PH-interacting protein	1.3	0.021
Q9UKF6	CPSF3	Cleavage and polyadenylation specificity factor subunit 3	1.3	0.022
Q99426	TBCB	Tubulin-folding cofactor B	1.3	0.022
Q13148	TARDBP	TAR DNA-binding protein 43	1.3	0.022
P35250	RFC2	Replication factor C subunit 2	1.3	0.022
P51153	RAB13	Ras-related protein Rab-13	1.3	0.023
O94927	HAUS5	HAUS augmin-like complex subunit 5	1.3	0.023
P11177	PDHB	Pyruvate dehydrogenase E1 component subunit beta, mitochondrial	1.3	0.032
P11172	UMPS	Uridine 5'-monophosphate synthase	1.3	0.033
O75717	WDHD1	WD repeat and HMG-box DNA-binding protein 1	1.3	0.034
P09543	CNP	2',3'-cyclic-nucleotide 3'-phosphodiesterase	1.3	0.034
F2Z2T2	XPA	DNA repair protein-complementing XP-A cells	1.3	0.036
Q16543	CDC37	Hsp90 co-chaperone Cdc37	1.3	0.039
Q9BWF3	RBM4	RNA-binding protein 4	1.3	0.043
Q15154	PCM1	Pericentriolar material 1 protein	1.3	0.045
P13797	PLS3	Plastin-3	1.2	<0.001
Q9UHD8	SEPTIN9	Septin-9	1.2	0.001
P10606	COX5B	Cytochrome c oxidase subunit 5B, mitochondrial	1.2	0.003
Q92597	NDRG1	Protein NDRG1	1.2	0.004
Q13347	EIF3I	Eukaryotic translation initiation factor 3 subunit I	1.2	0.004
P36578	RPL4	60S ribosomal protein L4	1.2	0.004
A0A0C4DGA6	HLTF	Helicase-like transcription factor	1.2	0.005
Q9UMS4	PRPF19	Pre-mRNA-processing factor 19	1.2	0.007
P62701	RPS4X	40S ribosomal protein S4, X isoform	1.2	0.008
Q6PKG0	LARP1	La-related protein 1	1.2	0.008
P23246	SFPQ	Splicing factor, proline- and glutamine-rich	1.2	0.009
Q5BKZ1	ZNF326	DBIRD complex subunit ZNF326	1.2	0.012
O60884	DNAJA2	DnaJ homolog subfamily A member 2	1.2	0.012
Q8WVJ2	NUDCD2	NudC domain-containing protein 2	1.2	0.013
P55884	EIF3B	Eukaryotic translation initiation factor 3 subunit B	1.2	0.018
P62269	RPS18	40S ribosomal protein S18	1.2	0.022
O75832	PSMD10	26S proteasome non-ATPase regulatory subunit 10	1.2	0.023
G5EA36	CDC27	Cell division cycle 27, isoform CRA_c	1.2	0.027
P40937	RFC5	Replication factor C subunit 5	1.2	0.038
O43615	TIMM44	Mitochondrial import inner membrane translocase subunit TIM44	1.2	0.039
P14866	HNRNPL	Heterogeneous nuclear ribonucleoprotein L	1.2	0.045
Q13435	SF3B2	Splicing factor 3B subunit 2	1.2	0.047
P17987	TCP1	T-complex protein 1 subunit alpha	1.1	0.003
P09622	DLD	Dihydrolipoyl dehydrogenase, mitochondrial	1.1	0.003
P62195	PSMC5	26S proteasome regulatory subunit 8	1.1	0.004
Q96C36	PYCR2	Pyrroline-5-carboxylate reductase 2	1.1	0.005
O15047	SETD1A	Histone-lysine N-methyltransferase SETD1A	1.1	0.007
Q14847	LASP1	LIM and SH3 domain protein 1	1.1	0.009
P49327	FASN	Fatty acid synthase	1.1	0.009
Q15555	MAPRE2	Microtubule-associated protein RP/EB family member 2	1.1	0.014
A0A2R8Y855	SMARCE1	SWI/SNF-related matrix-associated actin-dependent regulator of chromatin subfamily E member 1	1.1	0.015
O15294	OGT	UDP-N-acetylglucosamine--peptide N-acetylglucosaminyltransferase 110 kDa subunit	1.1	0.017
Q15126	PMVK	Phosphomevalonate kinase	1.1	0.020
Q9Y5X1	SNX9	Sorting nexin-9	1.1	0.021
P45974	USP5	Ubiquitin carboxyl-terminal hydrolase 5	1.1	0.023
Q9UPT5	EXOC7	Exocyst complex component 7	1.1	0.023
P54136	RARS1	Arginine--tRNA ligase, cytoplasmic	1.1	0.024
Q32MZ4	LRRFIP1	Leucine-rich repeat flightless-interacting protein 1	1.1	0.026
Q9H0D6	XRN2	5'-3' exoribonuclease 2	1.1	0.027
[illegible]	[illegible]	[illegible]	[illegible]	[illegible]

P53618	COPB1	Coatomer subunit beta	1.1	0.028
P35580	MYH10	Myosin-10	1.1	0.030
Q9NVH2	INTS7	Integrator complex subunit 7	1.1	0.030
Q13907	IDI1	Isopentenyl-diphosphate Delta-isomerase 1	1.1	0.031
Q9BSD7	NTPCR	Cancer-related nucleoside-triphosphatase	1.1	0.033
O95232	LUC7L3	Luc7-like protein 3	1.1	0.033
Q9UN86	G3BP2	Ras GTPase-activating protein-binding protein 2	1.1	0.038
Q9UJC3	HOOK1	Protein Hook homolog 1	1.1	0.042
P56545	CTBP2	C-terminal-binding protein 2	1.1	0.043
A0A2R8Y5F1	TSC2	Tuberin	1.1	0.044
Q15008	PSMD6	26S proteasome non-ATPase regulatory subunit 6	1.1	0.045
Q7Z417	NUFIP2	Nuclear fragile X mental retardation-interacting protein 2	1.0	0.001
Q8ND24	RNF214	RING finger protein 214	1.0	0.008
P53396	ACLY	ATP-citrate synthase	1.0	0.008
Q13155	AIMP2	Aminoacyl tRNA synthase complex-interacting multifunctional protein 2	1.0	0.009
P38919	EIF4A3	Eukaryotic initiation factor 4A-III	1.0	0.010
Q9UN37	VPS4A	Vacuolar protein sorting-associated protein 4A	1.0	0.015
P26639	TARS1	Threonine--tRNA ligase 1, cytoplasmic	1.0	0.020
Q9Y613	FHOD1	FH1/FH2 domain-containing protein 1	1.0	0.028
P18858	LIG1	DNA ligase 1	1.0	0.032
P49591	SARS1	Serine--tRNA ligase, cytoplasmic	1.0	0.035
P62917	RPL8	60S ribosomal protein L8	1.0	0.037
**PRDX2 cysteine-dependent interactors**				
Q9BQA1	WDR77	Methylosome protein 50	7.2	<0.001
Q9NQX3	GPHN	Gephyrin [Includes: Molybdopterin adenylyltransferase	6.4	<0.001
E7EVA0	MAP4	Microtubule-associated protein	6.1	<0.001
O14744	PRMT5	Protein arginine N-methyltransferase 5	6.0	<0.001
Q9C0C2	TNKS1BP1	182 kDa tankyrase-1-binding protein	5.8	<0.001
P07355	ANXA2	Annexin A2	5.8	<0.001
E9PGZ1	CALD1	Caldesmon	5.3	<0.001
Q7Z434	MAVS	Mitochondrial antiviral-signaling protein	5.2	<0.001
Q96KB5	PBK	Lymphokine-activated killer T-cell-originated protein kinase	4.6	<0.001
O60664	PLIN3	Perilipin-3	4.4	<0.001
Q9UHV9	PFDN2	Prefoldin subunit 2	4.4	<0.001
Q8NC51	SERBP1	Plasminogen activator inhibitor 1 RNA-binding protein	4.4	<0.001
Q9NXV2	KCTD5	BTB/POZ domain-containing protein KCTD5	4.4	0.033
Q14247	CTTN	Src substrate cortactin	4.0	<0.001
O96007	MOCS2	Molybdopterin synthase catalytic subunit	4.0	0.014
Q9Y266	NUDC	Nuclear migration protein nudC	3.8	<0.001
Q5T6F2	UBAP2	Ubiquitin-associated protein 2	3.7	<0.001
Q9NPH2	ISYNA1	Inositol-3-phosphate synthase 1	3.6	0.001
Q9NUQ3	TXLNG	Gamma-taxilin	3.5	<0.001
Q9Y570	PPME1	Protein phosphatase methylesterase 1	3.5	<0.001
P11802	CDK4	Cyclin-dependent kinase 4	3.5	<0.001
Q13541	EIF4EBP1	Eukaryotic translation initiation factor 4E-binding protein 1	3.5	0.001
Q9Y6A5	TACC3	Transforming acidic coiled-coil-containing protein 3	3.5	0.001
Q9ULW0	TPX2	Targeting protein for Xklp2	3.5	0.002
O14965	AURKA	Aurora kinase A	3.4	<0.001
P10599	TXN	Thioredoxin	3.4	<0.001
Q5SW79	CEP170	Centrosomal protein of 170 kDa	3.4	<0.001
Q8NFC6	BOD1L1	Biorientation of chromosomes in cell division protein 1-like 1	3.3	<0.001
P61758	VBP1	Prefoldin subunit 3	3.3	0.001
Q32MZ4	LRRFIP1	Leucine-rich repeat flightless-interacting protein 1	3.2	<0.001
Q9HA64	FN3KRP	Ketosamine-3-kinase	3.1	<0.001
P46821	MAP1B	Microtubule-associated protein 1B	3.1	<0.001
P35520	CBS	Cystathionine beta-synthase	3.1	<0.001
Q9P2B4	CTTNBP2NL	CTTNBP2 N-terminal-like protein	3.1	0.001
Q16204	CCDC6	Coiled-coil domain-containing protein 6	3.1	0.004
A6NGP5	JPT2	Jupiter microtubule-associated homolog 2	3.0	<0.001
P52888	THOP1	Thimet oligopeptidase	3.0	<0.001
Q13428	TCOF1	Treacle protein	3.0	<0.001
Q8IYS1	PM20D2	Peptidase M20 domain-containing protein 2	3.0	0.001
P26599	PTBP1	Polypyrimidine tract-binding protein 1	2.9	<0.001
P40222	TXLNA	Alpha-taxilin	2.9	0.002
Q13542	EIF4EBP2	Eukaryotic translation initiation factor 4E-binding protein 2	2.9	0.024
E7EPN9	PRRC2C	Protein PRRC2C	2.8	<0.001
A0A087WTM1	ROBO1	Roundabout homolog 1	2.8	0.002
Q96F45	ZNF503	Zinc finger protein 503	2.8	0.003
P22061	PCMT1	Protein-L-isoaspartate(D-aspartate) O-methyltransferase	2.8	0.005
Q6WKZ4	RAB11FIP1	Rab11 family-interacting protein 1	2.7	0.003
Q8IWB7	WDFY1	WD repeat and FYVE domain-containing protein 1	2.7	0.004
O95104	SCAF4	SR-related and CTD-associated factor 4	2.7	0.005
P52907	CAPZA1	F-actin-capping protein subunit alpha-1	2.6	<0.001
Q9H773	DCTPP1	dCTP pyrophosphatase 1	2.6	0.001
Q9H2U2	PPA2	Inorganic pyrophosphatase 2, mitochondrial	2.6	0.002
B7Z7F3	RANBP3	Ran-binding protein 3	2.6	0.004
E7EV99	ADD1	Alpha-adducin	2.6	0.005
P30740	SERPINB1	Leukocyte elastase inhibitor	2.6	0.005
A0A0A0MRN5	OGFR	Opioid growth factor receptor	2.6	0.005
P48634	PRRC2A	Protein PRRC2A	2.5	<0.001
O60547	GMDS	GDP-mannose 4,6 dehydratase	2.5	0.005
F5H8D7	XRCC1	DNA repair protein XRCC1	2.5	0.006
P85037	FOXK1	Forkhead box protein K1	2.5	0.013
O75223	GGCT	Gamma-glutamylcyclotransferase	2.5	0.020
Q9BRP1	PDCD2L	Programmed cell death protein 2-like	2.4	0.005
Q8N3X1	FNBP4	Formin-binding protein 4	2.4	0.006
P12955	PEPD	Xaa-Pro dipeptidase	2.4	0.021
Q8N6M0	OTUD6B	Deubiquitinase OTUD6B	2.4	0.022
O15511	ARPC5	Actin-related protein 2/3 complex subunit 5	2.4	0.043
Q06124	PTPN11	Tyrosine-protein phosphatase non-receptor type 11	2.3	<0.001
Q12948	FOXC1	Forkhead box protein C1	2.3	0.002
O00629	KPNA4	Importin subunit alpha-3	2.3	0.010
O43809	NUDT21	Cleavage and polyadenylation specificity factor subunit 5	2.3	0.011
Q9H0S4	DDX47	Probable ATP-dependent RNA helicase DDX47	2.3	0.017
Q96EA4	SPDL1	Protein Spindly	2.3	0.033
A0A1B0GW38	CBL	E3 ubiquitin-protein ligase CBL	2.2	<0.001
H0YNW5	DUT	Deoxyuridine 5'-triphosphate nucleotidohydrolase	2.2	<0.001
Q16576	RBBP7	Histone-binding protein RBBP7	2.2	<0.001
Q86V48	LUZP1	Leucine zipper protein 1	2.2	<0.001
P35237	SERPINB6	Serpin B6	2.2	<0.001
P48507	GCLM	Glutamate--cysteine ligase regulatory subunit	2.2	0.002
Q9P258	RCC2	Protein RCC2	2.2	0.002
Q8TAQ2	SMARCC2	SWI/SNF complex subunit SMARCC2	2.2	0.008
O43143	DHX15	Pre-mRNA-splicing factor ATP-dependent RNA helicase DHX15	2.2	0.008
Q96IZ0	PAWR	PRKC apoptosis WT1 regulator protein	2.2	0.015
P49903	SEPHS1	Selenide, water dikinase 1	2.2	0.028
Q6FI81	CIAPIN1	Anamorsin	2.2	0.033
Q8N806	UBR7	Putative E3 ubiquitin-protein ligase UBR7	2.2	0.035
Q6PKG0	LARP1	La-related protein 1	2.1	<0.001
J3KN29	PSMD9	26S proteasome non-ATPase regulatory subunit 9	2.1	0.001
Q96K76	USP47	Ubiquitin carboxyl-terminal hydrolase 47	2.1	0.014
O60826	CCDC22	Coiled-coil domain-containing protein 22	2.1	0.019
Q99598	TSNAX	Translin-associated protein X	2.1	0.026
Q9NVP1	DDX18	ATP-dependent RNA helicase DDX18	2.1	0.029
Q8WVC2	RPS21	40S ribosomal protein S21	2.1	0.029
O00566	MPHOSPH10	U3 small nucleolar ribonucleoprotein protein MPP10	2.1	0.035
C9J0I9	ZC3HC1	Nuclear-interacting partner of ALK	2.0	<0.001
Q9Y3F4	STRAP	Serine-threonine kinase receptor-associated protein	2.0	<0.001
Q93034	CUL5	Cullin-5	2.0	0.001
A0AVT1	UBA6	Ubiquitin-like modifier-activating enzyme 6	2.0	0.003
P54646	PRKAA2	5'-AMP-activated protein kinase catalytic subunit alpha-2	2.0	0.006
Q6IQ49	SDE2	Replication stress response regulator SDE2	2.0	0.018
Q5JRA6	MIA3	Transport and Golgi organization protein 1 homolog	2.0	0.018
Q9ULX6	AKAP8L	A-kinase anchor protein 8-like	2.0	0.026
A4D1S0	KLRG2	Killer cell lectin-like receptor subfamily G member 2	2.0	0.043
Q9H3K6	BOLA2	BolA-like protein 2	1.9	<0.001
Q5QPM7	PSMF1	Proteasome inhibitor PI31 subunit	1.9	<0.001
Q96F86	EDC3	Enhancer of mRNA-decapping protein 3	1.9	0.002
Q15417	CNN3	Calponin-3	1.9	0.002
P10301	RRAS	Ras-related protein R-Ras	1.9	0.017
P19174	PLCG1	1-phosphatidylinositol 4,5-bisphosphate phosphodiesterase gamma-1	1.9	0.025
Q9UL15	BAG5	BAG family molecular chaperone regulator 5	1.9	0.028
Q8WYA6	CTNNBL1	Beta-catenin-like protein 1	1.9	0.038
Q5T4K5	CRTC2	CREB-regulated transcription coactivator 2	1.9	0.040
P82921	MRPS21	28S ribosomal protein S21, mitochondrial	1.9	0.040
B1AK87	CAPZB	F-actin-capping protein subunit beta	1.8	<0.001
P53985	SLC16A1	Monocarboxylate transporter 1	1.8	<0.001
Q9H3S7	PTPN23	Tyrosine-protein phosphatase non-receptor type 23	1.8	0.030
Q9BT25	HAUS8	HAUS augmin-like complex subunit 8	1.8	0.039
P49458	SRP9	Signal recognition particle 9 kDa protein	1.8	0.048
B4DDF4	CNN2	Calponin	1.7	0.008
J3QQZ9	PNPO	Pyridoxal 5'-phosphate synthase	1.7	0.016
Q13547	HDAC1	Histone deacetylase 1	1.7	0.017
Q92783	STAM	Signal transducing adapter molecule 1	1.7	0.040
Q9HCN8	SDF2L1	Stromal cell-derived factor 2-like protein 1	1.7	0.040
Q9NXF7	DCAF16	DDB1- and CUL4-associated factor 16	1.7	0.041
P11413	G6PD	Glucose-6-phosphate 1-dehydrogenase	1.6	0.001
Q9UNF1	MAGED2	Melanoma-associated antigen D2	1.6	0.001
O00273	DFFA	DNA fragmentation factor subunit alpha	1.6	0.002
Q9UHD1	CHORDC1	Cysteine and histidine-rich domain-containing protein 1	1.6	0.002
Q9C0C9	UBE2O	(E3-independent) E2 ubiquitin-conjugating enzyme	1.6	0.004
O14929	HAT1	Histone acetyltransferase type B catalytic subunit	1.6	0.008
P49790	NUP153	Nuclear pore complex protein Nup153	1.6	0.010
P63167	DYNLL1	Dynein light chain 1, cytoplasmic	1.6	0.031
Q9UQE7	SMC3	Structural maintenance of chromosomes protein 3	1.6	0.035
Q14240	EIF4A2	Eukaryotic initiation factor 4A-II	1.6	0.048
E9PGT1	TSN	Component 3 of promoter of RISC	1.6	0.048
P45985	MAP2K4	Dual specificity mitogen-activated protein kinase kinase 4	1.5	<0.001
P22314	UBA1	Ubiquitin-like modifier-activating enzyme 1	1.5	0.015
Q9UI12	ATP6V1H	V-type proton ATPase subunit H	1.4	0.001
Q9BTE6	AARSD1	Alanyl-tRNA editing protein Aarsd1	1.4	0.001
P42771	CDKN2A	Cyclin-dependent kinase inhibitor 2A	1.4	0.001
Q15365	PCBP1	Poly(rC)-binding protein 1	1.4	0.001
Q07666	KHDRBS1	KH domain-containing, RNA-binding, signal transduction-associated protein 1	1.4	0.017
Q9UN37	VPS4A	Vacuolar protein sorting-associated protein 4A	1.4	0.018
O14974	PPP1R12A	Protein phosphatase 1 regulatory subunit 12A	1.4	0.041
Q96D09	GPRASP2	G-protein coupled receptor-associated sorting protein 2	1.3	0.005
P51452	DUSP3	Dual specificity protein phosphatase 3	1.3	0.006
Q8WWK9	CKAP2	Cytoskeleton-associated protein 2	1.3	0.009
A0MZ66	SHTN1	Shootin-1	1.3	0.024
P22234	PAICS	Multifunctional protein ADE2 [Includes: Phosphoribosylaminoimidazole-succinocarboxamide synthase	1.3	0.025
Q92667	AKAP1	A-kinase anchor protein 1, mitochondrial	1.3	0.034
O75663	TIPRL	TIP41-like protein	1.3	0.036
Q9NYZ3	GTSE1	G2 and S phase-expressed protein 1	1.3	0.043
G3XAG1	ZNF512	Zinc finger protein 512	1.3	0.050
P13797	PLS3	Plastin-3	1.2	0.001
Q92945	KHSRP	Far upstream element-binding protein 2	1.2	0.003
Q9H7E9	C8orf33	UPF0488 protein C8orf33	1.2	0.008
Q96HC4	PDLIM5	PDZ and LIM domain protein 5	1.2	0.010
E9PLA9	CAPRIN1	Caprin-1	1.2	0.014
O95816	BAG2	BAG family molecular chaperone regulator 2	1.2	0.030
Q7Z6Z7	HUWE1	E3 ubiquitin-protein ligase HUWE1	1.2	0.038
Q92598	HSPH1	Heat shock protein 105 kDa	1.1	0.007
Q2NKX8	ERCC6L	DNA excision repair protein ERCC-6-like	1.1	0.014
Q86YP4	GATAD2A	Transcriptional repressor p66-alpha	1.1	0.017
Q68EM7	ARHGAP17	Rho GTPase-activating protein 17	1.1	0.021
O00170	AIP	AH receptor-interacting protein	1.1	0.031
Q92769	HDAC2	Histone deacetylase 2	1.1	0.044
Q96P47	AGAP3	Arf-GAP with GTPase, ANK repeat and PH domain-containing protein 3	1.0	0.007
Q13200	PSMD2	26S proteasome non-ATPase regulatory subunit 2	1.0	0.009
O60343	TBC1D4	TBC1 domain family member 4	1.0	0.018
P49321	NASP	Nuclear autoantigenic sperm protein	1.0	0.019
**PRDX3 cysteine-dependent interactors**				
F8VYE8	PPP1CC	Serine/threonine-protein phosphatase	4.4	<0.001
P05386	RPLP1	60S acidic ribosomal protein P1	4.3	<0.001
Q8N5K1	CISD2	CDGSH iron-sulfur domain-containing protein 2	4.1	<0.001
P11766	ADH5	Alcohol dehydrogenase class-3	4.0	<0.001
P51148	RAB5C	Ras-related protein Rab-5C	3.9	0.001
Q7L2H7	EIF3M	Eukaryotic translation initiation factor 3 subunit M	3.7	<0.001
P06132	UROD	Uroporphyrinogen decarboxylase	3.7	0.001
P40616	ARL1	ADP-ribosylation factor-like protein 1	3.6	<0.001
Q15084	PDIA6	Protein disulfide-isomerase A6	3.6	<0.001
Q6DKI1	RPL7L1	60S ribosomal protein L7-like 1	3.6	0.001
Q07820	MCL1	Induced myeloid leukemia cell differentiation protein Mcl-1	3.5	0.001
Q6IBS0	TWF2	Twinfilin-2	3.4	<0.001
H3BTA2	PPP4C	Serine/threonine-protein phosphatase	3.4	<0.001
P19022	CDH2	Cadherin-2	3.4	0.003
Q07021	C1QBP	Complement component 1 Q subcomponent-binding protein, mitochondrial	3.3	<0.001
P07355	ANXA2	Annexin A2	3.3	<0.001
P30041	PRDX6	Peroxiredoxin-6	3.3	0.001
P22061	PCMT1	Protein-L-isoaspartate(D-aspartate) O-methyltransferase	3.3	0.002
Q9NS69	TOMM22	Mitochondrial import receptor subunit TOM22 homolog	3.2	0.001
P14174	MIF	Macrophage migration inhibitory factor	3.2	0.002
Q5SZR4	TDRKH	Tudor and KH domain containing, isoform CRA_a	3.2	0.007
Q15814	TBCC	Tubulin-specific chaperone C	3.2	0.013
A0A2R8YFH5	SEC23B	Protein transport protein SEC23	3.2	0.042
Q9NWV4	CZIB	CXXC motif containing zinc binding protein	3.1	0.002
Q86WA6	BPHL	Valacyclovir hydrolase	3.1	0.003
P51809	VAMP7	Vesicle-associated membrane protein 7	3.1	0.006
Q9ULX3	NOB1	RNA-binding protein NOB1	3.0	0.001
Q9Y5Y2	NUBP2	Cytosolic Fe-S cluster assembly factor NUBP2	3.0	0.001
Q5SY16	NOL9	Polynucleotide 5'-hydroxyl-kinase NOL9	3.0	0.001
Q92990	GLMN	Glomulin	3.0	0.001
Q96EA4	SPDL1	Protein Spindly	3.0	0.008
Q5VW32	BROX	BRO1 domain-containing protein BROX	3.0	0.009
G5EA06	MRPS27	28S ribosomal protein S27, mitochondrial	3.0	0.025
Q92785	DPF2	Zinc finger protein ubi-d4	2.9	0.004
Q9H6R0	DHX33	ATP-dependent RNA helicase DHX33	2.9	0.027
Q9BSJ8	ESYT1	Extended synaptotagmin-1	2.8	<0.001
O43809	NUDT21	Cleavage and polyadenylation specificity factor subunit 5	2.8	0.003
B3KUS5	USP30	Ubiquitin carboxyl-terminal hydrolase	2.8	0.007
H3BRY6	INTS14	Integrator complex subunit 14	2.8	0.029
A6NDU8	C5orf51	UPF0600 protein C5orf51	2.8	0.034
P61106	RAB14	Ras-related protein Rab-14	2.7	<0.001
P78346	RPP30	Ribonuclease P protein subunit p30	2.7	0.001
Q9Y4B6	DCAF1	DDB1- and CUL4-associated factor 1	2.7	0.002
Q9UKK9	NUDT5	ADP-sugar pyrophosphatase	2.7	0.002
Q9NYK5	MRPL39	39S ribosomal protein L39, mitochondrial	2.7	0.004
F8W038	C17orf49	Chromosome 17 open reading frame 49	2.7	0.007
Q66PJ3	ARL6IP4	ADP-ribosylation factor-like protein 6-interacting protein 4	2.7	0.007
Q5JRI1	SRSF10	Serine/arginine-rich-splicing factor 10	2.7	0.036
Q7Z434	MAVS	Mitochondrial antiviral-signaling protein	2.6	<0.001
Q9Y3D0	CIAO2B	Cytosolic iron-sulfur assembly component 2B	2.6	0.005
P57076	CFAP298	Cilia- and flagella-associated protein 298	2.6	0.007
Q9NZ45	CISD1	CDGSH iron-sulfur domain-containing protein 1	2.6	0.029
Q8IYS1	PM20D2	Peptidase M20 domain-containing protein 2	2.5	0.006
Q9NP61	ARFGAP3	ADP-ribosylation factor GTPase-activating protein 3	2.5	0.006
Q9BTE1	DCTN5	Dynactin subunit 5	2.5	0.012
Q92804	TAF15	TATA-binding protein-associated factor 2N	2.5	0.017
Q9HD26	GOPC	Golgi-associated PDZ and coiled-coil motif-containing protein	2.5	0.018
C9JAW5	C9JAW5	HIG1 domain-containing protein	2.5	0.018
M0R026	ILVBL	2-hydroxyacyl-CoA lyase 2	2.4	0.002
B4DJ81	NDUFS1	NADH-ubiquinone oxidoreductase 75 kDa subunit, mitochondrial	2.4	0.003
Q15291	RBBP5	Retinoblastoma-binding protein 5	2.4	0.004
Q8IVS2	MCAT	Malonyl-CoA-acyl carrier protein transacylase, mitochondrial	2.4	0.004
D6RA00	ENOPH1	Enolase-phosphatase E1	2.4	0.004
A0A087WU06	TUBGCP3	Gamma-tubulin complex component	2.4	0.009
Q16698	DECR1	2,4-dienoyl-CoA reductase, mitochondrial	2.4	0.009
F5H008	VPS33B	Vacuolar protein sorting-associated protein 33B	2.4	0.013
Q9H9P8	L2HGDH	L-2-hydroxyglutarate dehydrogenase, mitochondrial	2.4	0.017
A0A0A0MS29	MFF	Mitochondrial fission factor	2.4	0.027
P10599	TXN	Thioredoxin	2.3	0.002
Q16718	NDUFA5	NADH dehydrogenase [ubiquinone] 1 alpha subcomplex subunit 5	2.3	0.003
Q9P287	BCCIP	BRCA2 and CDKN1A-interacting protein	2.3	0.004
Q9NP97	DYNLRB1	Dynein light chain roadblock-type 1	2.3	0.009
P54646	PRKAA2	5'-AMP-activated protein kinase catalytic subunit alpha-2	2.3	0.012
Q14161	GIT2	ARF GTPase-activating protein GIT2	2.3	0.013
O75146	HIP1R	Huntingtin-interacting protein 1-related protein	2.3	0.016
P00492	HPRT1	Hypoxanthine-guanine phosphoribosyltransferase	2.3	0.016
Q8N6M0	OTUD6B	Deubiquitinase OTUD6B	2.3	0.018
Q8WVC2	RPS21	40S ribosomal protein S21	2.3	0.022
P18669	PGAM1	Phosphoglycerate mutase 1	2.3	0.040
A0A0A0MR02	VDAC2	Outer mitochondrial membrane protein porin 2	2.2	0.001
Q8N6T3	ARFGAP1	ADP-ribosylation factor GTPase-activating protein 1	2.2	0.003
Q9BQC3	DPH2	2-(3-amino-3-carboxypropyl)histidine synthase subunit 2	2.2	0.009
Q96IU4	ABHD14B	Protein ABHD14B	2.2	0.022
O95295	SNAPIN	SNARE-associated protein Snapin	2.2	0.023
Q8N6R0	EEF1AKNMT	eEF1A lysine and N-terminal methyltransferase	2.2	0.025
Q9BYN0	SRXN1	Sulfiredoxin-1	2.2	0.044
P11802	CDK4	Cyclin-dependent kinase 4	2.1	0.001
A0A0A0MTB8	WDR36	WD repeat-containing protein 36	2.1	0.010
X6RM00	ERC1	ELKS/Rab6-interacting/CAST family member 1	2.1	0.016
P30740	SERPINB1	Leukocyte elastase inhibitor	2.1	0.017
Q13509	TUBB3	Tubulin beta-3 chain	2.1	0.017
H3BV80	RNPS1	RNA-binding protein with serine-rich domain 1	2.1	0.022
Q5T749	KPRP	Keratinocyte proline-rich protein	2.1	0.034
Q92667	AKAP1	A-kinase anchor protein 1, mitochondrial	2.0	0.003
Q8NBU5	ATAD1	ATPase family AAA domain-containing protein 1	2.0	0.011
Q96AT9	RPE	Ribulose-phosphate 3-epimerase	2.0	0.015
Q9UQR0	SCML2	Sex comb on midleg-like protein 2	2.0	0.018
Q9UHD2	TBK1	Serine/threonine-protein kinase TBK1	2.0	0.020
Q9HCN8	SDF2L1	Stromal cell-derived factor 2-like protein 1	2.0	0.022
O43815	STRN	Striatin	2.0	0.025
Q9BZX2	UCK2	Uridine-cytidine kinase 2	2.0	0.029
P04843	RPN1	Dolichyl-diphosphooligosaccharide--protein glycosyltransferase subunit 1	1.9	0.002
P62879	GNB2	Guanine nucleotide-binding protein G(I)/G(S)/G(T) subunit beta-2	1.9	0.007
Q2M2I8	AAK1	AP2-associated protein kinase 1	1.9	0.020
O75600	GCAT	2-amino-3-ketobutyrate coenzyme A ligase, mitochondrial	1.9	0.038
Q96S44	TP53RK	EKC/KEOPS complex subunit TP53RK	1.8	0.033
O15269	SPTLC1	Serine palmitoyltransferase 1	1.7	0.009
Q96BW9	TAMM41	Phosphatidate cytidylyltransferase, mitochondrial	1.7	0.027
Q96A49	SYAP1	Synapse-associated protein 1	1.7	0.033
O43237	DYNC1LI2	Cytoplasmic dynein 1 light intermediate chain 2	1.7	0.036
Q9HB71	CACYBP	Calcyclin-binding protein	1.7	0.041
Q9H974	QTRT2	Queuine tRNA-ribosyltransferase accessory subunit 2	1.7	0.048
A0A0A0MR09	PTPN9	Tyrosine-protein phosphatase non-receptor type 9	1.7	0.050
P62979	RPS27A	Ubiquitin-40S ribosomal protein S27a	1.6	0.004
Q9Y679	AUP1	Lipid droplet-regulating VLDL assembly factor AUP1	1.6	0.034
E9PJN0	ACOT8	Acyl-coenzyme A thioesterase 8	1.6	0.044
P35268	RPL22	60S ribosomal protein L22	1.6	0.045
Q8TDH9	BLOC1S5	Biogenesis of lysosome-related organelles complex 1 subunit 5	1.5	0.004
B7WPG3	HNRNPLL	Heterogeneous nuclear ribonucleoprotein L-like	1.5	0.007
O14828	SCAMP3	Secretory carrier-associated membrane protein 3	1.5	0.034
Q9H0E2	TOLLIP	Toll-interacting protein	1.5	0.037
Q14318	FKBP8	Peptidyl-prolyl cis-trans isomerase FKBP8	1.4	0.009
P27824	CANX	Calnexin	1.4	0.011
P18031	PTPN1	Tyrosine-protein phosphatase non-receptor type 1	1.4	0.026
Q9UL15	BAG5	BAG family molecular chaperone regulator 5	1.4	0.041
Q9UBE0	SAE1	SUMO-activating enzyme subunit 1	1.4	0.043
Q6PKG0	LARP1	La-related protein 1	1.3	0.009
P21964	COMT	Catechol O-methyltransferase	1.3	0.011
P62081	RPS7	40S ribosomal protein S7	1.3	0.017
P35520	CBS	Cystathionine beta-synthase	1.3	0.026
O60784	TOM1	Target of Myb protein 1	1.2	0.005
Q5QPM7	PSMF1	Proteasome inhibitor PI31 subunit	1.2	0.011
P50454	SERPINH1	Serpin H1	1.2	0.013
Q5TDH0	DDI2	Protein DDI1 homolog 2	1.2	0.014
P31040	SDHA	Succinate dehydrogenase [ubiquinone] flavoprotein subunit, mitochondrial	1.2	0.016
K7ERF1	EIF3K	Eukaryotic translation initiation factor 3 subunit K	1.2	0.024
Q9UJS0	SLC25A13	Calcium-binding mitochondrial carrier protein Aralar2	1.2	0.035
Q9H0U4	RAB1B	Ras-related protein Rab-1B	1.2	0.048
P35237	SERPINB6	Serpin B6	1.1	0.006
P62873	GNB1	Guanine nucleotide-binding protein G(I)/G(S)/G(T) subunit beta-1	1.1	0.007
Q9GZS3	WDR61	WD repeat-containing protein 61	1.1	0.012
G3V279	ERH	Enhancer of rudimentary homolog	1.1	0.022
P08240	SRPRA	Signal recognition particle receptor subunit alpha	1.1	0.023
Q14697	GANAB	Neutral alpha-glucosidase AB	1.1	0.026
H7BXI1	ESYT2	Extended synaptotagmin-2	1.1	0.047
P22695	UQCRC2	Cytochrome b-c1 complex subunit 2, mitochondrial	1.0	0.024
**PRDX4 cysteine-dependent interactors**				
I3L1P8	SLC25A11	Mitochondrial 2-oxoglutarate/malate carrier protein	3.9	<0.001
Q9BS26	ERP44	Endoplasmic reticulum resident protein 44	3.8	0.002
Q8NBS9	TXNDC5	Thioredoxin domain-containing protein 5	3.7	<0.001
Q96AC1	FERMT2	Fermitin family homolog 2	3.4	<0.001
Q9Y2R5	MRPS17	28S ribosomal protein S17, mitochondrial	3.2	0.002
F5H6E2	MYO1C	Unconventional myosin-Ic	2.8	0.003
Q9BSJ8	ESYT1	Extended synaptotagmin-1	2.7	0.002
A0A0B4J1Z1	SRSF7	Serine/arginine-rich-splicing factor 7	2.7	0.007
Q66K74	MAP1S	Microtubule-associated protein 1S	2.4	0.008
Q12797	ASPH	Aspartyl/asparaginyl beta-hydroxylase	2.3	0.002
D6RA00	ENOPH1	Enolase-phosphatase E1	2.3	0.008
P30154	PPP2R1B	Serine/threonine-protein phosphatase 2A 65 kDa regulatory subunit A beta isoform	2.3	0.012
P09110	ACAA1	3-ketoacyl-CoA thiolase, peroxisomal	2.2	0.004
P30041	PRDX6	Peroxiredoxin-6	2.2	0.012
Q92783	STAM	Signal transducing adapter molecule 1	2.2	0.015
Q96DH6	MSI2	RNA-binding protein Musashi homolog 2	2.2	0.026
F5GZS6	SLC3A2	4F2 cell-surface antigen heavy chain	2.1	0.008
Q9NW82	WDR70	WD repeat-containing protein 70	2.1	0.029
B4DJ81	NDUFS1	NADH-ubiquinone oxidoreductase 75 kDa subunit, mitochondrial	2.0	0.022
P42345	MTOR	Serine/threonine-protein kinase mTOR	2.0	0.031
Q15084	PDIA6	Protein disulfide-isomerase A6	1.9	0.029
Q93034	CUL5	Cullin-5	1.8	0.041
P22234	PAICS	Multifunctional protein ADE2 [Includes: Phosphoribosylaminoimidazole-succinocarboxamide synthase	1.7	0.003
P62249	RPS16	40S ribosomal protein S16	1.7	0.006
Q14739	LBR	Delta(14)-sterol reductase LBR	1.7	0.010
P62906	RPL10A	60S ribosomal protein L10a	1.7	0.047
C9J9K3	RPSA	40S ribosomal protein SA	1.6	0.004
Q9UHV9	PFDN2	Prefoldin subunit 2	1.6	0.005
O43809	NUDT21	Cleavage and polyadenylation specificity factor subunit 5	1.6	0.013
Q96K76	USP47	Ubiquitin carboxyl-terminal hydrolase 47	1.5	0.002
Q5SW79	CEP170	Centrosomal protein of 170 kDa	1.5	0.006
P22307	SCP2	Non-specific lipid-transfer protein	1.5	0.006
P49411	TUFM	Elongation factor Tu, mitochondrial	1.5	0.011
A0A2R8Y7S2	SMARCA4	Transcription activator BRG1	1.5	0.019
E9PGZ1	CALD1	Caldesmon	1.5	0.045
Q14697	GANAB	Neutral alpha-glucosidase AB	1.4	0.004
P60174	TPI1	Triosephosphate isomerase	1.4	0.011
Q9UQR0	SCML2	Sex comb on midleg-like protein 2	1.4	0.017
Q16513	PKN2	Serine/threonine-protein kinase N2	1.4	0.019
P10599	TXN	Thioredoxin	1.4	0.041
O14974	PPP1R12A	Protein phosphatase 1 regulatory subunit 12A	1.4	0.043
Q14152	EIF3A	Eukaryotic translation initiation factor 3 subunit A	1.4	0.046
Q86UP2	KTN1	Kinectin	1.3	0.006
Q9Y4B6	DCAF1	DDB1- and CUL4-associated factor 1	1.3	0.017
Q96I99	SUCLG2	Succinate--CoA ligase [GDP-forming] subunit beta, mitochondrial	1.3	0.033
Q13257	MAD2L1	Mitotic spindle assembly checkpoint protein MAD2A	1.3	0.038
Q9P2J5	LARS1	Leucine--tRNA ligase, cytoplasmic	1.3	0.049
P06576	ATP5F1B	ATP synthase subunit beta, mitochondrial	1.2	0.002
P06733	ENO1	Alpha-enolase	1.2	0.006
Q9BTZ2	DHRS4	Dehydrogenase/reductase SDR family member 4	1.2	0.011
P34897	SHMT2	Serine hydroxymethyltransferase, mitochondrial	1.2	0.015
P05388	RPLP0	60S acidic ribosomal protein P0	1.2	0.016
P26599	PTBP1	Polypyrimidine tract-binding protein 1	1.2	0.017
P18858	LIG1	DNA ligase 1	1.2	0.020
P18077	RPL35A	60S ribosomal protein L35a	1.2	0.024
Q9HCD5	NCOA5	Nuclear receptor coactivator 5	1.2	0.037
O15372	EIF3H	Eukaryotic translation initiation factor 3 subunit H	1.1	0.024
A0A087WY71	AP2M1	AP-2 complex subunit mu	1.1	0.024
Q12904	AIMP1	Aminoacyl tRNA synthase complex-interacting multifunctional protein 1	1.1	0.036
Q96C36	PYCR2	Pyrroline-5-carboxylate reductase 2	1.0	0.012
Q13347	EIF3I	Eukaryotic translation initiation factor 3 subunit I	1.0	0.017
P34949	MPI	Mannose-6-phosphate isomerase	1.0	0.022
P13489	RNH1	Ribonuclease inhibitor	1.0	0.036
P15880	RPS2	40S ribosomal protein S2	1.0	0.040
**PRDX5 cysteine-dependent interactors**				
P36551	CPOX	Oxygen-dependent coproporphyrinogen-III oxidase, mitochondrial	5.2	<0.001
Q96A49	SYAP1	Synapse-associated protein 1	4.6	<0.001
Q15388	TOMM20	Mitochondrial import receptor subunit TOM20 homolog	4.0	0.001
A0A0C4DGQ6	RPRD1A	Regulation of nuclear pre-mRNA domain-containing protein 1A	3.2	<0.001
Q9H2P9	DPH5	Diphthine methyl ester synthase	3.2	0.003
P26599	PTBP1	Polypyrimidine tract-binding protein 1	3.0	<0.001
Q9GZU8	PSME3IP1	PSME3-interacting protein	3.0	0.002
Q9BS26	ERP44	Endoplasmic reticulum resident protein 44	3.0	0.006
P78371	CCT2	T-complex protein 1 subunit beta	2.9	<0.001
Q8NFC6	BOD1L1	Biorientation of chromosomes in cell division protein 1-like 1	2.9	<0.001
Q53GQ0	HSD17B12	Very-long-chain 3-oxoacyl-CoA reductase	2.9	0.002
E9PGT1	TSN	Component 3 of promoter of RISC	2.8	<0.001
P63167	DYNLL1	Dynein light chain 1, cytoplasmic	2.8	0.007
O95801	TTC4	Tetratricopeptide repeat protein 4	2.7	0.005
Q6DKK2	TTC19	Tetratricopeptide repeat protein 19, mitochondrial	2.7	0.008
Q2TAM5	RELA	RELA protein	2.6	0.004
Q99598	TSNAX	Translin-associated protein X	2.5	<0.001
Q5T760	SRSF11	Serine/arginine-rich-splicing factor 11	2.5	0.006
O60664	PLIN3	Perilipin-3	2.5	0.006
Q16698	DECR1	2,4-dienoyl-CoA reductase, mitochondrial	2.5	0.007
Q10713	PMPCA	Mitochondrial-processing peptidase subunit alpha	2.5	0.008
Q16822	PCK2	Phosphoenolpyruvate carboxykinase [GTP], mitochondrial	2.5	0.016
B7Z7F3	RANBP3	Ran-binding protein 3	2.4	<0.001
Q9Y266	NUDC	Nuclear migration protein nudC	2.4	0.002
Q92990	GLMN	Glomulin	2.4	0.010
Q8N6M0	OTUD6B	Deubiquitinase OTUD6B	2.4	0.014
P85037	FOXK1	Forkhead box protein K1	2.4	0.019
Q6PID6	TTC33	Tetratricopeptide repeat protein 33	2.4	0.020
P22234	PAICS	Multifunctional protein ADE2 [Includes: Phosphoribosylaminoimidazole-succinocarboxamide synthase	2.3	<0.001
P43304	GPD2	Glycerol-3-phosphate dehydrogenase, mitochondrial	2.3	0.001
P48047	ATP5PO	ATP synthase subunit O, mitochondrial	2.3	0.001
P22061	PCMT1	Protein-L-isoaspartate(D-aspartate) O-methyltransferase	2.3	0.009
H7C128	BRD8	Bromodomain-containing protein 8	2.2	0.001
A0A0B4J1Z1	SRSF7	Serine/arginine-rich-splicing factor 7	2.2	0.029
Q9Y277	VDAC3	Voltage-dependent anion-selective channel protein 3	2.2	0.036
Q9HB71	CACYBP	Calcyclin-binding protein	2.1	<0.001
Q8N3X1	FNBP4	Formin-binding protein 4	2.1	<0.001
Q14C86	GAPVD1	GTPase-activating protein and VPS9 domain-containing protein 1	2.1	<0.001
A0A0A0MR02	VDAC2	Outer mitochondrial membrane protein porin 2	2.1	0.001
Q05655	PRKCD	Protein kinase C delta type	2.1	0.001
O00483	NDUFA4	Cytochrome c oxidase subunit NDUFA4	2.1	0.016
Q15750	TAB1	TGF-beta-activated kinase 1 and MAP3K7-binding protein 1	2.1	0.022
Q9H2U1	DHX36	ATP-dependent DNA/RNA helicase DHX36	2.1	0.024
A0A024R442	A0A024R442	>tr|A0A024R442|A0A024R442_HUMAN Aspartyl aminopeptidase, isoform CRA_b OS=Homo sapiens OX=9606 GN=DNPEP PE=1 SV=1;>sp|Q9ULA0|DNPEP_HUMAN Aspartyl aminopeptidase OS=Homo sapiens OX=9606 GN=DNPEP PE=1 SV=1;>tr|E7ETB3|E7ETB3_HUMAN Aspartyl aminopeptidase, iso	2.1	0.032
P40222	TXLNA	Alpha-taxilin	2.0	0.001
P43686	PSMC4	26S proteasome regulatory subunit 6B	2.0	0.003
Q9H773	DCTPP1	dCTP pyrophosphatase 1	2.0	0.005
Q9BTY7	HGH1	Protein HGH1 homolog	2.0	0.007
P12955	PEPD	Xaa-Pro dipeptidase	2.0	0.025
P62979	RPS27A	Ubiquitin-40S ribosomal protein S27a	1.9	0.001
Q9BSJ8	ESYT1	Extended synaptotagmin-1	1.9	0.002
P07355	ANXA2	Annexin A2	1.9	0.007
P30041	PRDX6	Peroxiredoxin-6	1.9	0.015
Q9H0E2	TOLLIP	Toll-interacting protein	1.9	0.016
Q9P2N5	RBM27	RNA-binding protein 27	1.9	0.028
Q5JRA6	MIA3	Transport and Golgi organization protein 1 homolog	1.9	0.030
Q6WKZ4	RAB11FIP1	Rab11 family-interacting protein 1	1.9	0.034
Q15645	TRIP13	Pachytene checkpoint protein 2 homolog	1.9	0.037
E9PJN0	ACOT8	Acyl-coenzyme A thioesterase 8	1.8	<0.001
Q8NC51	SERBP1	Plasminogen activator inhibitor 1 RNA-binding protein	1.8	0.001
F5GZS6	SLC3A2	4F2 cell-surface antigen heavy chain	1.8	0.005
P00492	HPRT1	Hypoxanthine-guanine phosphoribosyltransferase	1.8	0.012
P56385	ATP5ME	ATP synthase subunit e, mitochondrial	1.8	0.016
Q9Y6A5	TACC3	Transforming acidic coiled-coil-containing protein 3	1.8	0.016
E9PGZ1	CALD1	Caldesmon	1.8	0.019
Q9BW92	TARS2	Threonine--tRNA ligase, mitochondrial	1.8	0.019
Q15061	WDR43	WD repeat-containing protein 43	1.8	0.033
Q66PJ3	ARL6IP4	ADP-ribosylation factor-like protein 6-interacting protein 4	1.8	0.034
Q04446	GBE1	1,4-alpha-glucan-branching enzyme	1.8	0.045
A0AVT1	UBA6	Ubiquitin-like modifier-activating enzyme 6	1.7	0.005
Q9UL15	BAG5	BAG family molecular chaperone regulator 5	1.7	0.005
O43815	STRN	Striatin	1.7	0.008
E7EV99	ADD1	Alpha-adducin	1.7	0.019
Q9BQA1	WDR77	Methylosome protein 50	1.7	0.036
Q9NQX3	GPHN	Gephyrin [Includes: Molybdopterin adenylyltransferase	1.7	0.036
P14174	MIF	Macrophage migration inhibitory factor	1.7	0.045
P31689	DNAJA1	DnaJ homolog subfamily A member 1	1.6	<0.001
Q9Y570	PPME1	Protein phosphatase methylesterase 1	1.6	0.009
O14929	HAT1	Histone acetyltransferase type B catalytic subunit	1.6	0.009
O00165	HAX1	HCLS1-associated protein X-1	1.6	0.013
F8W1A4	AK2	Adenylate kinase 2, mitochondrial	1.6	0.026
Q9NT62	ATG3	Ubiquitin-like-conjugating enzyme ATG3	1.6	0.029
Q9NQP4	PFDN4	Prefoldin subunit 4	1.6	0.049
Q9BQ69	MACROD1	ADP-ribose glycohydrolase MACROD1	1.6	0.050
Q15276	RABEP1	Rab GTPase-binding effector protein 1	1.5	0.003
C9J9K3	RPSA	40S ribosomal protein SA	1.5	0.004
Q9BSD7	NTPCR	Cancer-related nucleoside-triphosphatase	1.5	0.006
Q02952	AKAP12	A-kinase anchor protein 12	1.5	0.015
Q9BRS2	RIOK1	Serine/threonine-protein kinase RIO1	1.5	0.016
Q9C0C2	TNKS1BP1	182 kDa tankyrase-1-binding protein	1.5	0.039
Q09666	AHNAK	Neuroblast differentiation-associated protein AHNAK	1.4	0.001
Q16531	DDB1	DNA damage-binding protein 1	1.4	0.003
P05386	RPLP1	60S acidic ribosomal protein P1	1.4	0.004
Q96IZ0	PAWR	PRKC apoptosis WT1 regulator protein	1.4	0.008
Q9NZL9	MAT2B	Methionine adenosyltransferase 2 subunit beta	1.4	0.015
Q8NBU5	ATAD1	ATPase family AAA domain-containing protein 1	1.4	0.016
P52888	THOP1	Thimet oligopeptidase	1.4	0.027
Q12931	TRAP1	Heat shock protein 75 kDa, mitochondrial	1.3	0.003
Q32MZ4	LRRFIP1	Leucine-rich repeat flightless-interacting protein 1	1.3	0.010
P15880	RPS2	40S ribosomal protein S2	1.3	0.012
Q13185	CBX3	Chromobox protein homolog 3	1.3	0.012
Q96RL1	UIMC1	BRCA1-A complex subunit RAP80	1.3	0.014
H7BZM7	ZPR1	Zinc finger protein ZPR1	1.2	0.003
P51665	PSMD7	26S proteasome non-ATPase regulatory subunit 7	1.2	0.006
Q9Y3F4	STRAP	Serine-threonine kinase receptor-associated protein	1.2	0.006
Q9UNM6	PSMD13	26S proteasome non-ATPase regulatory subunit 13	1.2	0.010
P04843	RPN1	Dolichyl-diphosphooligosaccharide--protein glycosyltransferase subunit 1	1.2	0.036
P46821	MAP1B	Microtubule-associated protein 1B	1.2	0.047
E9PLA9	CAPRIN1	Caprin-1	1.1	0.002
A0A087WUT6	EIF5B	Eukaryotic translation initiation factor 5B	1.1	0.008
P22570	FDXR	NADPH:adrenodoxin oxidoreductase, mitochondrial	1.1	0.008
Q9UI12	ATP6V1H	V-type proton ATPase subunit H	1.1	0.014
Q5T6F2	UBAP2	Ubiquitin-associated protein 2	1.1	0.015
E7EVA0	MAP4	Microtubule-associated protein	1.1	0.022
C9J4Z3	RPL37A	60S ribosomal protein L37a	1.1	0.041
P62195	PSMC5	26S proteasome regulatory subunit 8	1.0	0.005
P25398	RPS12	40S ribosomal protein S12	1.0	0.016
P11413	G6PD	Glucose-6-phosphate 1-dehydrogenase	1.0	0.036
P62333	PSMC6	26S proteasome regulatory subunit 10B	1.0	0.037
O14579	COPE	Coatomer subunit epsilon	1.0	0.038

Table S2. PRDX-specific cysteine-dependent interactors

gene	uniprot ID	protein	fold change	adj. p-value	against
**PRDX1 -specific interactors**					
A0A075B6R9	IGKV2D-24	Probable non-functional immunoglobulin kappa variable 2D-24	2.9	0.001	PRDX2
F8W9F9	WNK2	Non-specific serine/threonine protein kinase	2.5	0.001	PRDX2
Q06830	PRDX1	Peroxiredoxin-1	2.4	0.000	PRDX2
O60566	BUB1B	Mitotic checkpoint serine/threonine-protein kinase BUB1 beta	2.4	0.001	PRDX2
G3V3B9	GSTZ1	Maleylacetoacetate isomerase	2.4	0.001	PRDX2
Q6NZY4	ZCCHC8	Zinc finger CCHC domain-containing protein 8	2.4	0.008	PRDX2
P30044	PRDX5	Peroxiredoxin-5, mitochondrial	2.3	0.028	PRDX2
O95218	ZRANB2	Zinc finger Ran-binding domain-containing protein 2	2.2	0.003	PRDX2
P19387	POLR2C	DNA-directed RNA polymerase II subunit RPB3	2.2	0.004	PRDX2
Q9Y3B7	MRPL11	39S ribosomal protein L11, mitochondrial	2.1	0.000	PRDX2
A0A3B3ITJ4	HNRNPL	Heterogeneous nuclear ribonucleoprotein L	2.1	0.004	PRDX2
O00399	DCTN6	Dynactin subunit 6	2.1	0.006	PRDX2
Q9Y3D9	MRPS23	28S ribosomal protein S23, mitochondrial	2.1	0.017	PRDX2
A0A087X1B1	IKBKG	NF-kappa-B essential modulator	2.0	0.009	PRDX2
P14635	CCNB1	G2/mitotic-specific cyclin-B1	2.0	0.010	PRDX2
Q6UWE0	LRSAM1	E3 ubiquitin-protein ligase LRSAM1	2.0	0.011	PRDX2
Q12929	EPS8	Epidermal growth factor receptor kinase substrate 8	1.9	0.007	PRDX2
Q16352	INA	Alpha-internexin	1.8	0.000	PRDX2
P07196	NEFL	Neurofilament light polypeptide	1.8	0.001	PRDX2
Q12933	TRAF2	TNF receptor-associated factor 2	1.8	0.013	PRDX2
Q13418	ILK	Integrin-linked protein kinase	1.8	0.018	PRDX2
Q96DA6	DNAJC19	Mitochondrial import inner membrane translocase subunit TIM14	1.8	0.019	PRDX2
Q99956	DUSP9	Dual specificity protein phosphatase 9	1.8	0.024	PRDX2
P42765	ACAA2	3-ketoacyl-CoA thiolase, mitochondrial	1.7	0.000	PRDX2
Q9H0H5	RACGAP1	Rac GTPase-activating protein 1	1.7	0.000	PRDX2
Q8WVC0	LEO1	RNA polymerase-associated protein LEO1	1.7	0.026	PRDX2
Q9C026	TRIM9	E3 ubiquitin-protein ligase TRIM9	1.7	0.029	PRDX2
O75616	ERAL1	GTPase Era, mitochondrial	1.7	0.036	PRDX2
Q15398	DLGAP5	Disks large-associated protein 5	1.6	0.001	PRDX2
Q9ULX3	NOB1	RNA-binding protein NOB1	1.6	0.002	PRDX2
Q9BR76	CORO1B	Coronin-1B	1.6	0.012	PRDX2
Q9BW83	IFT27	Intraflagellar transport protein 27 homolog	1.6	0.024	PRDX2
Q6R327	RICTOR	Rapamycin-insensitive companion of mTOR	1.6	0.027	PRDX2
Q96B36	AKT1S1	Proline-rich AKT1 substrate 1	1.6	0.031	PRDX2
H3BPJ9	NDUFB10	Complex I-PDSW	1.6	0.032	PRDX2
P09497	CLTB	Clathrin light chain B	1.6	0.040	PRDX2
Q92974	ARHGEF2	Rho guanine nucleotide exchange factor 2	1.5	0.000	PRDX2
A0A0G2JNZ2	SCRIB	Protein scribble homolog	1.5	0.001	PRDX2
Q04837	SSBP1	Single-stranded DNA-binding protein, mitochondrial	1.5	0.001	PRDX2
Q96RT1	ERBIN	Erbin	1.5	0.003	PRDX2
Q6FI81	CIAPIN1	Anamorsin	1.5	0.013	PRDX2
Q13573	SNW1	SNW domain-containing protein 1	1.5	0.020	PRDX2
Q99447	PCYT2	Ethanolamine-phosphate cytidylyltransferase	1.5	0.028	PRDX2
F5H345	HMBS	Hydroxymethylbilane synthase	1.5	0.038	PRDX2
Q13015	MLLT11	Protein AF1q	1.5	0.049	PRDX2
Q9Y5M8	SRPRB	Signal recognition particle receptor subunit beta	1.4	0.000	PRDX2
A0A087WW06	TTC28	Tetratricopeptide repeat protein 28	1.4	0.000	PRDX2
Q86Y56	DNAAF5	Dynein assembly factor 5, axonemal	1.4	0.004	PRDX2
Q8N6R0	EEF1AKNMT	eEF1A lysine and N-terminal methyltransferase	1.4	0.050	PRDX2
Q8ND24	RNF214	RING finger protein 214	1.3	0.000	PRDX2
P39019	RPS19	40S ribosomal protein S19	1.3	0.000	PRDX2
Q9UN37	VPS4A	Vacuolar protein sorting-associated protein 4A	1.3	0.002	PRDX2
Q9UJC3	HOOK1	Protein Hook homolog 1	1.3	0.003	PRDX2
A0A087WUK2	HNRNPDL	Heterogeneous nuclear ribonucleoprotein D-like	1.3	0.006	PRDX2
Q13625	TP53BP2	Apoptosis-stimulating of p53 protein 2	1.3	0.006	PRDX2
Q9Y3S2	ZNF330	Zinc finger protein 330	1.3	0.016	PRDX2
Q99569	PKP4	Plakophilin-4	1.3	0.019	PRDX2
Q5VVQ6	YOD1	Ubiquitin thioesterase OTU1	1.3	0.045	PRDX2
Q4VCS5	AMOT	Angiomotin	1.3	0.049	PRDX2
C9JRJ5	LIMD1	LIM domain-containing protein 1	1.2	0.001	PRDX2
F8WB06	ATXN2	Ataxin-2	1.2	0.003	PRDX2
P35998	PSMC2	26S proteasome regulatory subunit 7	1.2	0.004	PRDX2
A0A0A0MRM8	MYO6	Unconventional myosin-6	1.2	0.004	PRDX2
H7C5E4	XRN1	5'-3' exoribonuclease 1	1.2	0.005	PRDX2
Q92990	GLMN	Glomulin	1.2	0.006	PRDX2
Q9UG54	MAP3K7	Mitogen-activated protein kinase kinase kinase	1.2	0.013	PRDX2
Q13123	IK	Protein Red	1.2	0.023	PRDX2
Q8WTW3	COG1	Conserved oligomeric Golgi complex subunit 1	1.2	0.025	PRDX2
Q92665	MRPS31	28S ribosomal protein S31, mitochondrial	1.2	0.033	PRDX2
Q8N3C0	ASCC3	Activating signal cointegrator 1 complex subunit 3	1.2	0.034	PRDX2
A0A087X0K9	TJP1	Tight junction protein ZO-1	1.2	0.041	PRDX2
Q12955	ANK3	Ankyrin-3	1.1	0.000	PRDX2
P28066	PSMA5	Proteasome subunit alpha type-5	1.1	0.000	PRDX2
Q96S55	WRNIP1	ATPase WRNIP1	1.1	0.001	PRDX2
Q15126	PMVK	Phosphomevalonate kinase	1.1	0.002	PRDX2
Q99543	DNAJC2	DnaJ homolog subfamily C member 2	1.1	0.011	PRDX2
Q9UN86	G3BP2	Ras GTPase-activating protein-binding protein 2	1.1	0.013	PRDX2
P57740	NUP107	Nuclear pore complex protein Nup107	1.1	0.015	PRDX2
Q14126	DSG2	Desmoglein-2	1.1	0.015	PRDX2
P0DMV9	HSPA1B	Heat shock 70 kDa protein 1B	1.1	0.017	PRDX2
Q15424	SAFB	Scaffold attachment factor B1	1.1	0.017	PRDX2
Q9NQT5	EXOSC3	Exosome complex component RRP40	1.1	0.026	PRDX2
P52272	HNRNPM	Heterogeneous nuclear ribonucleoprotein M	1.1	0.029	PRDX2
O00170	AIP	AH receptor-interacting protein	1.1	0.029	PRDX2
Q9BZE4	GTPBP4	Nucleolar GTP-binding protein 1	1.1	0.030	PRDX2
Q13136	PPFIA1	Liprin-alpha-1	1.1	0.032	PRDX2
Q9H1A4	ANAPC1	Anaphase-promoting complex subunit 1	1.1	0.044	PRDX2
Q92785	DPF2	Zinc finger protein ubi-d4	1.1	0.045	PRDX2
Q13464	ROCK1	Rho-associated protein kinase 1	1.1	0.048	PRDX2
O43823	AKAP8	A-kinase anchor protein 8	1.1	0.048	PRDX2
Q96IV0	NGLY1	Peptide-N(4)-(N-acetyl-beta-glucosaminyl)asparagine amidase	1.1	0.050	PRDX2
Q13451	FKBP5	Peptidyl-prolyl cis-trans isomerase FKBP5	1.0	0.005	PRDX2
Q92888	ARHGEF1	Rho guanine nucleotide exchange factor 1	1.0	0.006	PRDX2
P43487	RANBP1	Ran-specific GTPase-activating protein	1.0	0.008	PRDX2
Q86VS8	HOOK3	Protein Hook homolog 3	1.0	0.014	PRDX2
Q9H3K6	BOLA2	BolA-like protein 2	1.0	0.018	PRDX2
P19022	CDH2	Cadherin-2	1.0	0.034	PRDX2
Q06830	PRDX1	Peroxiredoxin-1	3.1	0.000	PRDX3
P30044	PRDX5	Peroxiredoxin-5, mitochondrial	3.1	0.002	PRDX3
Q6WKZ4	RAB11FIP1	Rab11 family-interacting protein 1	2.7	0.000	PRDX3
F8W9F9	WNK2	Non-specific serine/threonine protein kinase	2.7	0.000	PRDX3
A0A075B6R9	IGKV2D-24	Probable non-functional immunoglobulin kappa variable 2D-24	2.7	0.002	PRDX3
G3V3B9	GSTZ1	Maleylacetoacetate isomerase	2.6	0.000	PRDX3
P14174	MIF	Macrophage migration inhibitory factor	2.4	0.002	PRDX3
P19387	POLR2C	DNA-directed RNA polymerase II subunit RPB3	2.4	0.002	PRDX3
O95218	ZRANB2	Zinc finger Ran-binding domain-containing protein 2	2.3	0.001	PRDX3
E7EVA0	MAP4	Microtubule-associated protein	2.3	0.020	PRDX3
Q96A10	ERVK3-1	Endogenous retrovirus group K3 member 1	2.3	0.041	PRDX3
O95831	AIFM1	Apoptosis-inducing factor 1, mitochondrial	2.2	0.000	PRDX3
A0A087X1B1	IKBKG	NF-kappa-B essential modulator	2.2	0.005	PRDX3
Q6UWE0	LRSAM1	E3 ubiquitin-protein ligase LRSAM1	2.2	0.006	PRDX3
O60232	ZNRD2	Protein ZNRD2	2.2	0.014	PRDX3
P21912	SDHB	Succinate dehydrogenase [ubiquinone] iron-sulfur subunit, mitochondrial	2.1	0.004	PRDX3
Q12929	EPS8	Epidermal growth factor receptor kinase substrate 8	2.1	0.004	PRDX3
P14635	CCNB1	G2/mitotic-specific cyclin-B1	2.1	0.005	PRDX3
P11802	CDK4	Cyclin-dependent kinase 4	2.1	0.006	PRDX3
Q13015	MLLT11	Protein AF1q	2.1	0.006	PRDX3
Q9Y6A5	TACC3	Transforming acidic coiled-coil-containing protein 3	2.1	0.008	PRDX3
P62273	RPS29	40S ribosomal protein S29	2.1	0.016	PRDX3
B4DDF4	CNN2	Calponin	2.0	0.000	PRDX3
Q8NC51	SERBP1	Plasminogen activator inhibitor 1 RNA-binding protein	2.0	0.003	PRDX3
Q13541	EIF4EBP1	Eukaryotic translation initiation factor 4E-binding protein 1	2.0	0.005	PRDX3
Q96DA6	DNAJC19	Mitochondrial import inner membrane translocase subunit TIM14	2.0	0.009	PRDX3
Q13418	ILK	Integrin-linked protein kinase	2.0	0.009	PRDX3
Q12948	FOXC1	Forkhead box protein C1	1.9	0.002	PRDX3
H0YNW5	DUT	Deoxyuridine 5'-triphosphate nucleotidohydrolase	1.9	0.002	PRDX3
Q8WVC0	LEO1	RNA polymerase-associated protein LEO1	1.9	0.014	PRDX3
Q9C026	TRIM9	E3 ubiquitin-protein ligase TRIM9	1.9	0.015	PRDX3
P67936	TPM4	Tropomyosin alpha-4 chain	1.9	0.025	PRDX3
Q9BW83	IFT27	Intraflagellar transport protein 27 homolog	1.8	0.012	PRDX3
P35244	RPA3	Replication protein A 14 kDa subunit	1.8	0.020	PRDX3
P09497	CLTB	Clathrin light chain B	1.8	0.021	PRDX3
A8MYK1	MRPL23	39S ribosomal protein L23, mitochondrial	1.8	0.027	PRDX3
Q8TCF1	ZFAND1	AN1-type zinc finger protein 1	1.8	0.028	PRDX3
P47985	UQCRFS1	Cytochrome b-c1 complex subunit Rieske, mitochondrial	1.7	0.004	PRDX3
Q96B36	AKT1S1	Proline-rich AKT1 substrate 1	1.7	0.016	PRDX3
Q13151	HNRNPA0	Heterogeneous nuclear ribonucleoprotein A0	1.7	0.017	PRDX3
F5H345	HMBS	Hydroxymethylbilane synthase	1.7	0.020	PRDX3
O95248	SBF1	Myotubularin-related protein 5	1.7	0.028	PRDX3
O15511	ARPC5	Actin-related protein 2/3 complex subunit 5	1.7	0.028	PRDX3
C9JAW5	C9JAW5	HIG1 domain-containing protein	1.7	0.039	PRDX3
Q9Y3D9	MRPS23	28S ribosomal protein S23, mitochondrial	1.7	0.040	PRDX3
P49914	MTHFS	5-formyltetrahydrofolate cyclo-ligase	1.7	0.049	PRDX3
Q9UN86	G3BP2	Ras GTPase-activating protein-binding protein 2	1.6	0.000	PRDX3
Q9Y265	RUVBL1	RuvB-like 1	1.6	0.000	PRDX3
A0A2R8Y811	RPS14	40S ribosomal protein S14	1.6	0.000	PRDX3
P35520	CBS	Cystathionine beta-synthase	1.6	0.013	PRDX3
P61758	VBP1	Prefoldin subunit 3	1.6	0.025	PRDX3
Q9H4L4	SENP3	Sentrin-specific protease 3	1.6	0.032	PRDX3
P10301	RRAS	Ras-related protein R-Ras	1.6	0.046	PRDX3
Q9H0H5	RACGAP1	Rac GTPase-activating protein 1	1.5	0.000	PRDX3
Q96F86	EDC3	Enhancer of mRNA-decapping protein 3	1.5	0.003	PRDX3
Q13123	IK	Protein Red	1.5	0.008	PRDX3
Q5XKP0	MICOS13	MICOS complex subunit MIC13	1.5	0.041	PRDX3
Q9P2X3	IMPACT	Protein IMPACT	1.5	0.042	PRDX3
Q16576	RBBP7	Histone-binding protein RBBP7	1.4	0.003	PRDX3
Q9NUQ3	TXLNG	Gamma-taxilin	1.4	0.005	PRDX3
Q9BR76	CORO1B	Coronin-1B	1.4	0.021	PRDX3
E9PM92	C11orf58	Small acidic protein	1.4	0.032	PRDX3
Q8NFC6	BOD1L1	Biorientation of chromosomes in cell division protein 1-like 1	1.4	0.038	PRDX3
P30041	PRDX6	Peroxiredoxin-6	1.4	0.043	PRDX3
Q9NRX4	PHPT1	14 kDa phosphohistidine phosphatase	1.3	0.000	PRDX3
Q9Y5M8	SRPRB	Signal recognition particle receptor subunit beta	1.3	0.000	PRDX3
Q6IQ49	SDE2	Replication stress response regulator SDE2	1.3	0.002	PRDX3
Q9HB71	CACYBP	Calcyclin-binding protein	1.3	0.013	PRDX3
Q16204	CCDC6	Coiled-coil domain-containing protein 6	1.3	0.038	PRDX3
Q5VVQ6	YOD1	Ubiquitin thioesterase OTU1	1.3	0.044	PRDX3
P62826	RAN	GTP-binding nuclear protein Ran	1.2	0.000	PRDX3
Q92974	ARHGEF2	Rho guanine nucleotide exchange factor 2	1.2	0.005	PRDX3
Q9H936	SLC25A22	Mitochondrial glutamate carrier 1	1.2	0.005	PRDX3
P0DMV9	HSPA1B	Heat shock 70 kDa protein 1B	1.2	0.007	PRDX3
A0A087WUK2	HNRNPDL	Heterogeneous nuclear ribonucleoprotein D-like	1.2	0.009	PRDX3
Q15654	TRIP6	Thyroid receptor-interacting protein 6	1.2	0.013	PRDX3
Q13356	PPIL2	RING-type E3 ubiquitin-protein ligase PPIL2	1.2	0.016	PRDX3
Q6R327	RICTOR	Rapamycin-insensitive companion of mTOR	1.2	0.017	PRDX3
Q9H840	GEMIN7	Gem-associated protein 7	1.2	0.025	PRDX3
P32119	PRDX2	Peroxiredoxin-2	1.2	0.026	PRDX3
O00399	DCTN6	Dynactin subunit 6	1.2	0.027	PRDX3
P22234	PAICS	Multifunctional protein ADE2 [Includes: Phosphoribosylaminoimidazole-succinocarboxamide synthase	1.2	0.030	PRDX3
P56385	ATP5ME	ATP synthase subunit e, mitochondrial	1.2	0.031	PRDX3
P61927	RPL37	60S ribosomal protein L37	1.2	0.031	PRDX3
H0Y4R1	IMPDH2	Inosine-5'-monophosphate dehydrogenase 2	1.2	0.040	PRDX3
P39019	RPS19	40S ribosomal protein S19	1.1	0.000	PRDX3
Q15181	PPA1	Inorganic pyrophosphatase	1.1	0.006	PRDX3
P82921	MRPS21	28S ribosomal protein S21, mitochondrial	1.1	0.017	PRDX3
Q07666	KHDRBS1	KH domain-containing, RNA-binding, signal transduction-associated protein 1	1.1	0.029	PRDX3
O14965	AURKA	Aurora kinase A	1.1	0.034	PRDX3
Q13247	SRSF6	Serine/arginine-rich splicing factor 6	1.1	0.041	PRDX3
P40222	TXLNA	Alpha-taxilin	1.1	0.046	PRDX3
Q9ULX3	NOB1	RNA-binding protein NOB1	1.1	0.050	PRDX3
P11586	MTHFD1	C-1-tetrahydrofolate synthase, cytoplasmic	1.0	0.000	PRDX3
O95336	PGLS	6-phosphogluconolactonase	1.0	0.017	PRDX3
Q15398	DLGAP5	Disks large-associated protein 5	1.0	0.024	PRDX3
P30044	PRDX5	Peroxiredoxin-5, mitochondrial	3.5	0.001	PRDX4
Q06830	PRDX1	Peroxiredoxin-1	3.4	0.000	PRDX4
H0YNW5	DUT	Deoxyuridine 5'-triphosphate nucleotidohydrolase	3.2	0.000	PRDX4
A0A075B6R9	IGKV2D-24	Probable non-functional immunoglobulin kappa variable 2D-24	3.1	0.000	PRDX4
P62273	RPS29	40S ribosomal protein S29	3.1	0.001	PRDX4
P11802	CDK4	Cyclin-dependent kinase 4	3.0	0.000	PRDX4
P06132	UROD	Uroporphyrinogen decarboxylase	3.0	0.000	PRDX4
Q13015	MLLT11	Protein AF1q	2.9	0.000	PRDX4
Q6FI81	CIAPIN1	Anamorsin	2.9	0.000	PRDX4
Q9H840	GEMIN7	Gem-associated protein 7	2.8	0.000	PRDX4
Q9H2P9	DPH5	Diphthine methyl ester synthase	2.6	0.000	PRDX4
Q6WKZ4	RAB11FIP1	Rab11 family-interacting protein 1	2.5	0.000	PRDX4
F8W9F9	WNK2	Non-specific serine/threonine protein kinase	2.5	0.000	PRDX4
Q9NP97	DYNLRB1	Dynein light chain roadblock-type 1	2.5	0.000	PRDX4
G3V3B9	GSTZ1	Maleylacetoacetate isomerase	2.4	0.000	PRDX4
P19022	CDH2	Cadherin-2	2.4	0.000	PRDX4
B4DDF4	CNN2	Calponin	2.4	0.000	PRDX4
O60566	BUB1B	Mitotic checkpoint serine/threonine-protein kinase BUB1 beta	2.4	0.001	PRDX4
P61758	VBP1	Prefoldin subunit 3	2.4	0.002	PRDX4
P56385	ATP5ME	ATP synthase subunit e, mitochondrial	2.3	0.001	PRDX4
Q9Y6A5	TACC3	Transforming acidic coiled-coil-containing protein 3	2.3	0.009	PRDX4
A0A087X2D5	MRPL45	39S ribosomal protein L45, mitochondrial	2.2	0.000	PRDX4
Q8WVC2	RPS21	40S ribosomal protein S21	2.2	0.001	PRDX4
Q9BTE1	DCTN5	Dynactin subunit 5	2.2	0.001	PRDX4
H3BRE8	RPAP1	RNA polymerase II-associated protein 1	2.2	0.002	PRDX4
P19387	POLR2C	DNA-directed RNA polymerase II subunit RPB3	2.2	0.002	PRDX4
P0DMV9	HSPA1B	Heat shock 70 kDa protein 1B	2.1	0.000	PRDX4
O95218	ZRANB2	Zinc finger Ran-binding domain-containing protein 2	2.1	0.001	PRDX4
P41250	GARS1	Glycine--tRNA ligase	2.1	0.002	PRDX4
Q9NPH2	ISYNA1	Inositol-3-phosphate synthase 1	2.1	0.003	PRDX4
P42771	CDKN2A	Cyclin-dependent kinase inhibitor 2A	2.0	0.000	PRDX4
H7BXI1	ESYT2	Extended synaptotagmin-2	2.0	0.002	PRDX4
Q5JRI1	SRSF10	Serine/arginine-rich-splicing factor 10	2.0	0.004	PRDX4
A0A087X1B1	IKBKG	NF-kappa-B essential modulator	2.0	0.006	PRDX4
Q6UWE0	LRSAM1	E3 ubiquitin-protein ligase LRSAM1	2.0	0.007	PRDX4
E9PM92	C11orf58	Small acidic protein	2.0	0.007	PRDX4
O60232	ZNRD2	Protein ZNRD2	2.0	0.018	PRDX4
Q9UN86	G3BP2	Ras GTPase-activating protein-binding protein 2	1.9	0.000	PRDX4
Q9BT25	HAUS8	HAUS augmin-like complex subunit 8	1.9	0.000	PRDX4
P34897	SHMT2	Serine hydroxymethyltransferase, mitochondrial	1.9	0.000	PRDX4
P22234	PAICS	Multifunctional protein ADE2 [Includes: Phosphoribosylaminoimidazole-succinocarboxamide synthase	1.9	0.001	PRDX4
Q9BPW8	NIPSNAP1	Protein NipSnap homolog 1	1.9	0.004	PRDX4
P14635	CCNB1	G2/mitotic-specific cyclin-B1	1.9	0.007	PRDX4
P85037	FOXK1	Forkhead box protein K1	1.9	0.010	PRDX4
Q96KB5	PBK	Lymphokine-activated killer T-cell-originated protein kinase	1.9	0.016	PRDX4
O14964	HGS	Hepatocyte growth factor-regulated tyrosine kinase substrate	1.8	0.000	PRDX4
Q12933	TRAF2	TNF receptor-associated factor 2	1.8	0.009	PRDX4
O00629	KPNA4	Importin subunit alpha-3	1.8	0.011	PRDX4
Q99956	DUSP9	Dual specificity protein phosphatase 9	1.8	0.017	PRDX4
Q9Y265	RUVBL1	RuvB-like 1	1.7	0.000	PRDX4
P17252	PRKCA	Protein kinase C alpha type	1.7	0.001	PRDX4
P48047	ATP5PO	ATP synthase subunit O, mitochondrial	1.7	0.010	PRDX4
Q13541	EIF4EBP1	Eukaryotic translation initiation factor 4E-binding protein 1	1.7	0.011	PRDX4
Q13418	ILK	Integrin-linked protein kinase	1.7	0.012	PRDX4
Q16186	ADRM1	Proteasomal ubiquitin receptor ADRM1	1.7	0.012	PRDX4
Q9P2B4	CTTNBP2NL	CTTNBP2 N-terminal-like protein	1.7	0.013	PRDX4
Q96DH6	MSI2	RNA-binding protein Musashi homolog 2	1.7	0.013	PRDX4
Q96DA6	DNAJC19	Mitochondrial import inner membrane translocase subunit TIM14	1.7	0.013	PRDX4
Q8WVC0	LEO1	RNA polymerase-associated protein LEO1	1.7	0.018	PRDX4
Q9C026	TRIM9	E3 ubiquitin-protein ligase TRIM9	1.7	0.021	PRDX4
Q8NFC6	BOD1L1	Biorientation of chromosomes in cell division protein 1-like 1	1.7	0.023	PRDX4
Q9HD26	GOPC	Golgi-associated PDZ and coiled-coil motif-containing protein	1.7	0.026	PRDX4
O75616	ERAL1	GTPase Era, mitochondrial	1.7	0.027	PRDX4
P14174	MIF	Macrophage migration inhibitory factor	1.7	0.028	PRDX4
Q5VUJ6	LRCH2	Leucine-rich repeat and calponin homology domain-containing protein 2	1.7	0.029	PRDX4
P67936	TPM4	Tropomyosin alpha-4 chain	1.7	0.035	PRDX4
Q9NXR7	BABAM2	BRISC and BRCA1-A complex member 2	1.7	0.040	PRDX4
Q9Y3D9	MRPS23	28S ribosomal protein S23, mitochondrial	1.7	0.040	PRDX4
Q9Y5Y2	NUBP2	Cytosolic Fe-S cluster assembly factor NUBP2	1.6	0.000	PRDX4
Q9ULX3	NOB1	RNA-binding protein NOB1	1.6	0.001	PRDX4
O15143	ARPC1B	Actin-related protein 2/3 complex subunit 1B	1.6	0.002	PRDX4
Q12948	FOXC1	Forkhead box protein C1	1.6	0.004	PRDX4
Q9Y5K6	CD2AP	CD2-associated protein	1.6	0.012	PRDX4
Q53H96	PYCR3	Pyrroline-5-carboxylate reductase 3	1.6	0.012	PRDX4
Q16204	CCDC6	Coiled-coil domain-containing protein 6	1.6	0.015	PRDX4
Q9BW83	IFT27	Intraflagellar transport protein 27 homolog	1.6	0.016	PRDX4
Q8NI60	COQ8A	Atypical kinase COQ8A, mitochondrial	1.6	0.017	PRDX4
Q6R327	RICTOR	Rapamycin-insensitive companion of mTOR	1.6	0.018	PRDX4
I3L2Z5	MAZ	Myc-associated zinc finger protein	1.6	0.022	PRDX4
P30041	PRDX6	Peroxiredoxin-6	1.6	0.025	PRDX4
O14617	AP3D1	AP-3 complex subunit delta-1	1.6	0.027	PRDX4
P35244	RPA3	Replication protein A 14 kDa subunit	1.6	0.029	PRDX4
P09497	CLTB	Clathrin light chain B	1.6	0.030	PRDX4
A8MYK1	MRPL23	39S ribosomal protein L23, mitochondrial	1.6	0.039	PRDX4
Q8TCF1	ZFAND1	AN1-type zinc finger protein 1	1.6	0.041	PRDX4
P53677	AP3M2	AP-3 complex subunit mu-2	1.5	0.003	PRDX4
P35520	CBS	Cystathionine beta-synthase	1.5	0.021	PRDX4
Q9BZE1	MRPL37	39S ribosomal protein L37, mitochondrial	1.5	0.022	PRDX4
U3KQC1	WDR18	WD repeat-containing protein 18	1.5	0.023	PRDX4
F5H345	HMBS	Hydroxymethylbilane synthase	1.5	0.028	PRDX4
P49903	SEPHS1	Selenide, water dikinase 1	1.5	0.038	PRDX4
P42025	ACTR1B	Beta-centractin	1.5	0.042	PRDX4
Q9NYZ3	GTSE1	G2 and S phase-expressed protein 1	1.4	0.000	PRDX4
P30837	ALDH1B1	Aldehyde dehydrogenase X, mitochondrial	1.4	0.000	PRDX4
A0A0C4DGQ6	RPRD1A	Regulation of nuclear pre-mRNA domain-containing protein 1A	1.4	0.001	PRDX4
Q9Y3B7	MRPL11	39S ribosomal protein L11, mitochondrial	1.4	0.001	PRDX4
Q96FW1	OTUB1	Ubiquitin thioesterase OTUB1	1.4	0.001	PRDX4
O95831	AIFM1	Apoptosis-inducing factor 1, mitochondrial	1.4	0.003	PRDX4
P40937	RFC5	Replication factor C subunit 5	1.4	0.003	PRDX4
Q9BWD1	ACAT2	Acetyl-CoA acetyltransferase, cytosolic	1.4	0.015	PRDX4
Q99569	PKP4	Plakophilin-4	1.4	0.015	PRDX4
A0A3B3IUD2	MSTO1	Protein misato homolog 1	1.4	0.025	PRDX4
E7EX48	NEK4	Serine/threonine-protein kinase Nek4	1.4	0.026	PRDX4
Q9UBI6	GNG12	Guanine nucleotide-binding protein G(I)/G(S)/G(O) subunit gamma-12	1.4	0.029	PRDX4
Q13573	SNW1	SNW domain-containing protein 1	1.4	0.031	PRDX4
Q8N3C0	ASCC3	Activating signal cointegrator 1 complex subunit 3	1.4	0.032	PRDX4
Q9NV56	MRGBP	MRG/MORF4L-binding protein	1.4	0.033	PRDX4
O00291	HIP1	Huntingtin-interacting protein 1	1.4	0.035	PRDX4
O94992	HEXIM1	Protein HEXIM1	1.4	0.038	PRDX4
O95248	SBF1	Myotubularin-related protein 5	1.4	0.040	PRDX4
Q6P087	RPUSD3	Mitochondrial mRNA pseudouridine synthase RPUSD3	1.4	0.040	PRDX4
G3V1Q4	SEPTIN7	Septin-7	1.4	0.045	PRDX4
Q9NPD8	UBE2T	Ubiquitin-conjugating enzyme E2 T	1.4	0.047	PRDX4
Q7L5D6	GET4	Golgi to ER traffic protein 4 homolog	1.4	0.050	PRDX4
P35232	PHB	Prohibitin	1.3	0.000	PRDX4
P06733	ENO1	Alpha-enolase	1.3	0.000	PRDX4
P62937	PPIA	Peptidyl-prolyl cis-trans isomerase A	1.3	0.001	PRDX4
Q9UN37	VPS4A	Vacuolar protein sorting-associated protein 4A	1.3	0.001	PRDX4
P43487	RANBP1	Ran-specific GTPase-activating protein	1.3	0.001	PRDX4
Q96RT1	ERBIN	Erbin	1.3	0.002	PRDX4
P31930	UQCRC1	Cytochrome b-c1 complex subunit 1, mitochondrial	1.3	0.003	PRDX4
P35998	PSMC2	26S proteasome regulatory subunit 7	1.3	0.003	PRDX4
Q6PKG0	LARP1	La-related protein 1	1.3	0.004	PRDX4
A0A087X0K9	TJP1	Tight junction protein ZO-1	1.3	0.004	PRDX4
Q14C86	GAPVD1	GTPase-activating protein and VPS9 domain-containing protein 1	1.3	0.013	PRDX4
J3KN29	PSMD9	26S proteasome non-ATPase regulatory subunit 9	1.3	0.020	PRDX4
Q96F86	EDC3	Enhancer of mRNA-decapping protein 3	1.3	0.020	PRDX4
P26599	PTBP1	Polypyrimidine tract-binding protein 1	1.3	0.025	PRDX4
P63000	RAC1	Ras-related C3 botulinum toxin substrate 1	1.3	0.026	PRDX4
P47985	UQCRFS1	Cytochrome b-c1 complex subunit Rieske, mitochondrial	1.3	0.030	PRDX4
Q13247	SRSF6	Serine/arginine-rich splicing factor 6	1.3	0.030	PRDX4
A0A3B3ITJ4	HNRNPL	Heterogeneous nuclear ribonucleoprotein L	1.3	0.031	PRDX4
P14618	PKM	Pyruvate kinase PKM	1.3	0.033	PRDX4
P43686	PSMC4	26S proteasome regulatory subunit 6B	1.3	0.033	PRDX4
Q5VVQ6	YOD1	Ubiquitin thioesterase OTU1	1.3	0.045	PRDX4
Q4VCS5	AMOT	Angiomotin	1.3	0.045	PRDX4
Q9H4L4	SENP3	Sentrin-specific protease 3	1.3	0.047	PRDX4
Q9Y5M8	SRPRB	Signal recognition particle receptor subunit beta	1.2	0.000	PRDX4
P11586	MTHFD1	C-1-tetrahydrofolate synthase, cytoplasmic	1.2	0.000	PRDX4
O95456	PSMG1	Proteasome assembly chaperone 1	1.2	0.001	PRDX4
P22695	UQCRC2	Cytochrome b-c1 complex subunit 2, mitochondrial	1.2	0.004	PRDX4
P49593	PPM1F	Protein phosphatase 1F	1.2	0.008	PRDX4
O14965	AURKA	Aurora kinase A	1.2	0.026	PRDX4
Q96B36	AKT1S1	Proline-rich AKT1 substrate 1	1.2	0.032	PRDX4
P11142	HSPA8	Heat shock cognate 71 kDa protein	1.1	0.000	PRDX4
P05091	ALDH2	Aldehyde dehydrogenase, mitochondrial	1.1	0.002	PRDX4
Q13347	EIF3I	Eukaryotic translation initiation factor 3 subunit I	1.1	0.004	PRDX4
Q2M1P5	KIF7	Kinesin-like protein KIF7	1.1	0.005	PRDX4
P19838	NFKB1	Nuclear factor NF-kappa-B p105 subunit	1.1	0.006	PRDX4
D6R938	CAMK2D	Calcium/calmodulin-dependent protein kinase	1.1	0.007	PRDX4
Q92974	ARHGEF2	Rho guanine nucleotide exchange factor 2	1.1	0.008	PRDX4
Q9BV44	THUMPD3	THUMP domain-containing protein 3	1.1	0.010	PRDX4
Q9UKK9	NUDT5	ADP-sugar pyrophosphatase	1.1	0.017	PRDX4
Q86WA6	BPHL	Valacyclovir hydrolase	1.1	0.020	PRDX4
Q9NXF7	DCAF16	DDB1- and CUL4-associated factor 16	1.1	0.021	PRDX4
K7EJQ8	HDHD2	Haloacid dehalogenase-like hydrolase domain-containing protein 2	1.1	0.029	PRDX4
P50542	PEX5	Peroxisomal targeting signal 1 receptor	1.1	0.030	PRDX4
O75607	NPM3	Nucleoplasmin-3	1.1	0.033	PRDX4
P21964	COMT	Catechol O-methyltransferase	1.1	0.036	PRDX4
Q6P1L8	MRPL14	39S ribosomal protein L14, mitochondrial	1.1	0.040	PRDX4
Q6DKI1	RPL7L1	60S ribosomal protein L7-like 1	1.1	0.046	PRDX4
P62195	PSMC5	26S proteasome regulatory subunit 8	1.0	0.003	PRDX4
Q14126	DSG2	Desmoglein-2	1.0	0.016	PRDX4
Q8WUM0	NUP133	Nuclear pore complex protein Nup133	1.0	0.016	PRDX4
Q9H223	EHD4	EH domain-containing protein 4	1.0	0.026	PRDX4
A0A0G2JNZ2	SCRIB	Protein scribble homolog	1.0	0.029	PRDX4
B1ALK7	ARHGEF7	Rho guanine nucleotide exchange factor 7	1.0	0.029	PRDX4
Q15398	DLGAP5	Disks large-associated protein 5	1.0	0.033	PRDX4
P23258	TUBG1	Tubulin gamma-1 chain	1.0	0.039	PRDX4
F5H008	VPS33B	Vacuolar protein sorting-associated protein 33B	1.0	0.040	PRDX4
Q53EL6	PDCD4	Programmed cell death protein 4	1.0	0.044	PRDX4
A0A075B6R9	IGKV2D-24	Probable non-functional immunoglobulin kappa variable 2D-24	3.3	0.000	PRDX5
Q4VCS5	AMOT	Angiomotin	3.0	0.000	PRDX5
P06132	UROD	Uroporphyrinogen decarboxylase	2.9	0.000	PRDX5
B4DDF4	CNN2	Calponin	2.9	0.000	PRDX5
Q13015	MLLT11	Protein AF1q	2.8	0.001	PRDX5
P62273	RPS29	40S ribosomal protein S29	2.8	0.003	PRDX5
Q9H840	GEMIN7	Gem-associated protein 7	2.6	0.000	PRDX5
Q06830	PRDX1	Peroxiredoxin-1	2.5	0.000	PRDX5
P19105	MYL12A	Myosin regulatory light chain 12A	2.5	0.001	PRDX5
Q9NP97	DYNLRB1	Dynein light chain roadblock-type 1	2.4	0.000	PRDX5
H0YNW5	DUT	Deoxyuridine 5'-triphosphate nucleotidohydrolase	2.4	0.000	PRDX5
F8W9F9	WNK2	Non-specific serine/threonine protein kinase	2.4	0.001	PRDX5
Q15149	PLEC	Plectin	2.4	0.004	PRDX5
Q9BR76	CORO1B	Coronin-1B	2.3	0.000	PRDX5
G3V3B9	GSTZ1	Maleylacetoacetate isomerase	2.3	0.001	PRDX5
P19022	CDH2	Cadherin-2	2.3	0.001	PRDX5
Q13573	SNW1	SNW domain-containing protein 1	2.3	0.002	PRDX5
O60566	BUB1B	Mitotic checkpoint serine/threonine-protein kinase BUB1 beta	2.3	0.002	PRDX5
P51570	GALK1	Galactokinase	2.2	0.000	PRDX5
A0A0G2JNZ2	SCRIB	Protein scribble homolog	2.2	0.000	PRDX5
O15143	ARPC1B	Actin-related protein 2/3 complex subunit 1B	2.2	0.001	PRDX5
Q96AC1	FERMT2	Fermitin family homolog 2	2.2	0.005	PRDX5
P42765	ACAA2	3-ketoacyl-CoA thiolase, mitochondrial	2.1	0.000	PRDX5
O00629	KPNA4	Importin subunit alpha-3	2.1	0.001	PRDX5
E7EPN9	PRRC2C	Protein PRRC2C	2.1	0.001	PRDX5
Q86WA6	BPHL	Valacyclovir hydrolase	2.1	0.002	PRDX5
P19387	POLR2C	DNA-directed RNA polymerase II subunit RPB3	2.1	0.004	PRDX5
Q13356	PPIL2	RING-type E3 ubiquitin-protein ligase PPIL2	2.0	0.000	PRDX5
Q9H0H5	RACGAP1	Rac GTPase-activating protein 1	2.0	0.000	PRDX5
Q9UN37	VPS4A	Vacuolar protein sorting-associated protein 4A	2.0	0.000	PRDX5
Q9BTE1	DCTN5	Dynactin subunit 5	2.0	0.002	PRDX5
O95218	ZRANB2	Zinc finger Ran-binding domain-containing protein 2	2.0	0.003	PRDX5
Q8WVC2	RPS21	40S ribosomal protein S21	2.0	0.003	PRDX5
Q9BZE1	MRPL37	39S ribosomal protein L37, mitochondrial	2.0	0.004	PRDX5
Q9Y3D9	MRPS23	28S ribosomal protein S23, mitochondrial	2.0	0.017	PRDX5
Q9UN86	G3BP2	Ras GTPase-activating protein-binding protein 2	1.9	0.000	PRDX5
P62879	GNB2	Guanine nucleotide-binding protein G(I)/G(S)/G(T) subunit beta-2	1.9	0.000	PRDX5
A0A087X2D5	MRPL45	39S ribosomal protein L45, mitochondrial	1.9	0.001	PRDX5
P07196	NEFL	Neurofilament light polypeptide	1.9	0.001	PRDX5
Q7L2E3	DHX30	ATP-dependent RNA helicase DHX30	1.9	0.002	PRDX5
Q6P1L8	MRPL14	39S ribosomal protein L14, mitochondrial	1.9	0.009	PRDX5
P14174	MIF	Macrophage migration inhibitory factor	1.9	0.011	PRDX5
Q6UWE0	LRSAM1	E3 ubiquitin-protein ligase LRSAM1	1.9	0.012	PRDX5
Q16822	PCK2	Phosphoenolpyruvate carboxykinase [GTP], mitochondrial	1.9	0.013	PRDX5
O60232	ZNRD2	Protein ZNRD2	1.9	0.024	PRDX5
C9J8P9	CLTA	Clathrin light chain	1.9	0.044	PRDX5
Q8TCG1	CIP2A	Protein CIP2A	1.8	0.001	PRDX5
P09110	ACAA1	3-ketoacyl-CoA thiolase, peroxisomal	1.8	0.005	PRDX5
Q5JRI1	SRSF10	Serine/arginine-rich-splicing factor 10	1.8	0.007	PRDX5
Q96DH6	MSI2	RNA-binding protein Musashi homolog 2	1.8	0.008	PRDX5
Q15477	SKIV2L	Helicase SKI2W	1.8	0.009	PRDX5
Q9BPW8	NIPSNAP1	Protein NipSnap homolog 1	1.8	0.009	PRDX5
O43347	MSI1	RNA-binding protein Musashi homolog 1	1.8	0.010	PRDX5
Q9NXH9	TRMT1	tRNA	1.8	0.012	PRDX5
C9JRJ5	LIMD1	LIM domain-containing protein 1	1.7	0.000	PRDX5
Q96RP9	GFM1	Elongation factor G, mitochondrial	1.7	0.002	PRDX5
Q12948	FOXC1	Forkhead box protein C1	1.7	0.002	PRDX5
Q99569	PKP4	Plakophilin-4	1.7	0.002	PRDX5
Q53H96	PYCR3	Pyrroline-5-carboxylate reductase 3	1.7	0.011	PRDX5
K7ELV2	SEH1L	Nucleoporin SEH1	1.7	0.017	PRDX5
Q9Y3B7	MRPL11	39S ribosomal protein L11, mitochondrial	1.6	0.000	PRDX5
Q9BT25	HAUS8	HAUS augmin-like complex subunit 8	1.6	0.000	PRDX5
P56545	CTBP2	C-terminal-binding protein 2	1.6	0.000	PRDX5
Q16352	INA	Alpha-internexin	1.6	0.001	PRDX5
P53597	SUCLG1	Succinate--CoA ligase [ADP/GDP-forming] subunit alpha, mitochondrial	1.6	0.002	PRDX5
Q6P2E9	EDC4	Enhancer of mRNA-decapping protein 4	1.6	0.003	PRDX5
Q9Y5K6	CD2AP	CD2-associated protein	1.6	0.004	PRDX5
Q8N3C0	ASCC3	Activating signal cointegrator 1 complex subunit 3	1.6	0.004	PRDX5
Q7Z406	MYH14	Myosin-14	1.6	0.005	PRDX5
O14964	HGS	Hepatocyte growth factor-regulated tyrosine kinase substrate	1.6	0.005	PRDX5
O75592	MYCBP2	E3 ubiquitin-protein ligase MYCBP2	1.6	0.005	PRDX5
Q13363	CTBP1	C-terminal-binding protein 1	1.6	0.010	PRDX5
A0A0A0MRT6	ABI1	Abl interactor 1	1.6	0.012	PRDX5
P48047	ATP5PO	ATP synthase subunit O, mitochondrial	1.6	0.013	PRDX5
Q12933	TRAF2	TNF receptor-associated factor 2	1.6	0.016	PRDX5
P42345	MTOR	Serine/threonine-protein kinase mTOR	1.6	0.017	PRDX5
Q13418	ILK	Integrin-linked protein kinase	1.6	0.021	PRDX5
Q96DA6	DNAJC19	Mitochondrial import inner membrane translocase subunit TIM14	1.6	0.022	PRDX5
Q99956	DUSP9	Dual specificity protein phosphatase 9	1.6	0.027	PRDX5
Q9C026	TRIM9	E3 ubiquitin-protein ligase TRIM9	1.6	0.035	PRDX5
P55084	HADHB	Trifunctional enzyme subunit beta, mitochondrial	1.6	0.039	PRDX5
Q5VUJ6	LRCH2	Leucine-rich repeat and calponin homology domain-containing protein 2	1.6	0.043	PRDX5
Q13526	PIN1	Peptidyl-prolyl cis-trans isomerase NIMA-interacting 1	1.5	0.000	PRDX5
Q96FW1	OTUB1	Ubiquitin thioesterase OTUB1	1.5	0.000	PRDX5
O43795	MYO1B	Unconventional myosin-Ib	1.5	0.001	PRDX5
P52272	HNRNPM	Heterogeneous nuclear ribonucleoprotein M	1.5	0.002	PRDX5
Q15398	DLGAP5	Disks large-associated protein 5	1.5	0.002	PRDX5
Q9BW92	TARS2	Threonine--tRNA ligase, mitochondrial	1.5	0.004	PRDX5
A0A087WTZ5	UBXN1	UBX domain-containing protein 1	1.5	0.006	PRDX5
P82921	MRPS21	28S ribosomal protein S21, mitochondrial	1.5	0.006	PRDX5
Q9BWD1	ACAT2	Acetyl-CoA acetyltransferase, cytosolic	1.5	0.008	PRDX5
O95785	WIZ	Protein Wiz	1.5	0.008	PRDX5
P46060	RANGAP1	Ran GTPase-activating protein 1	1.5	0.013	PRDX5
Q9Y2R4	DDX52	Probable ATP-dependent RNA helicase DDX52	1.5	0.013	PRDX5
H3BRE8	RPAP1	RNA polymerase II-associated protein 1	1.5	0.017	PRDX5
Q16186	ADRM1	Proteasomal ubiquitin receptor ADRM1	1.5	0.022	PRDX5
B1ALK7	ARHGEF7	Rho guanine nucleotide exchange factor 7	1.5	0.024	PRDX5
Q9BY32	ITPA	Inosine triphosphate pyrophosphatase	1.5	0.026	PRDX5
P16403	H1-2	Histone H1.2	1.5	0.029	PRDX5
Q8NI60	COQ8A	Atypical kinase COQ8A, mitochondrial	1.5	0.029	PRDX5
A0A3B3IUD2	MSTO1	Protein misato homolog 1	1.5	0.029	PRDX5
Q8WVC0	LEO1	RNA polymerase-associated protein LEO1	1.5	0.031	PRDX5
P17028	ZNF24	Zinc finger protein 24	1.5	0.035	PRDX5
A0A0A0MRM8	MYO6	Unconventional myosin-6	1.4	0.000	PRDX5
F8WB06	ATXN2	Ataxin-2	1.4	0.000	PRDX5
O95456	PSMG1	Proteasome assembly chaperone 1	1.4	0.000	PRDX5
Q9ULX3	NOB1	RNA-binding protein NOB1	1.4	0.001	PRDX5
Q86U42	PABPN1	Polyadenylate-binding protein 2	1.4	0.001	PRDX5
Q92974	ARHGEF2	Rho guanine nucleotide exchange factor 2	1.4	0.001	PRDX5
Q9Y5Y2	NUBP2	Cytosolic Fe-S cluster assembly factor NUBP2	1.4	0.002	PRDX5
Q9BSH4	TACO1	Translational activator of cytochrome c oxidase 1	1.4	0.002	PRDX5
P19838	NFKB1	Nuclear factor NF-kappa-B p105 subunit	1.4	0.002	PRDX5
Q9GZT3	SLIRP	SRA stem-loop-interacting RNA-binding protein, mitochondrial	1.4	0.003	PRDX5
P40937	RFC5	Replication factor C subunit 5	1.4	0.003	PRDX5
P23258	TUBG1	Tubulin gamma-1 chain	1.4	0.004	PRDX5
Q92888	ARHGEF1	Rho guanine nucleotide exchange factor 1	1.4	0.007	PRDX5
Q9P2B4	CTTNBP2NL	CTTNBP2 N-terminal-like protein	1.4	0.008	PRDX5
Q16513	PKN2	Serine/threonine-protein kinase N2	1.4	0.010	PRDX5
Q96PK6	RBM14	RNA-binding protein 14	1.4	0.012	PRDX5
O60783	MRPS14	28S ribosomal protein S14, mitochondrial	1.4	0.013	PRDX5
Q13247	SRSF6	Serine/arginine-rich splicing factor 6	1.4	0.019	PRDX5
Q96GD0	PDXP	Pyridoxal phosphate phosphatase	1.4	0.021	PRDX5
Q6DKI1	RPL7L1	60S ribosomal protein L7-like 1	1.4	0.026	PRDX5
Q99959	PKP2	Plakophilin-2	1.4	0.026	PRDX5
Q9BW83	IFT27	Intraflagellar transport protein 27 homolog	1.4	0.030	PRDX5
Q6R327	RICTOR	Rapamycin-insensitive companion of mTOR	1.4	0.034	PRDX5
O94760	DDAH1	N(G),N(G)-dimethylarginine dimethylaminohydrolase 1	1.4	0.042	PRDX5
P35244	RPA3	Replication protein A 14 kDa subunit	1.4	0.049	PRDX5
Q9NYZ3	GTSE1	G2 and S phase-expressed protein 1	1.3	0.001	PRDX5
Q9BSD7	NTPCR	Cancer-related nucleoside-triphosphatase	1.3	0.002	PRDX5
Q13625	TP53BP2	Apoptosis-stimulating of p53 protein 2	1.3	0.002	PRDX5
P07737	PFN1	Profilin-1	1.3	0.002	PRDX5
Q8IYI6	EXOC8	Exocyst complex component 8	1.3	0.003	PRDX5
P17252	PRKCA	Protein kinase C alpha type	1.3	0.005	PRDX5
Q96RT1	ERBIN	Erbin	1.3	0.006	PRDX5
F5H008	VPS33B	Vacuolar protein sorting-associated protein 33B	1.3	0.006	PRDX5
Q8WUM0	NUP133	Nuclear pore complex protein Nup133	1.3	0.007	PRDX5
Q13464	ROCK1	Rho-associated protein kinase 1	1.3	0.008	PRDX5
P28838	LAP3	Cytosol aminopeptidase	1.3	0.009	PRDX5
A5YKK6	CNOT1	CCR4-NOT transcription complex subunit 1	1.3	0.009	PRDX5
Q86UK7	ZNF598	E3 ubiquitin-protein ligase ZNF598	1.3	0.010	PRDX5
P48634	PRRC2A	Protein PRRC2A	1.3	0.012	PRDX5
O95373	IPO7	Importin-7	1.3	0.013	PRDX5
Q05655	PRKCD	Protein kinase C delta type	1.3	0.015	PRDX5
Q86V48	LUZP1	Leucine zipper protein 1	1.3	0.020	PRDX5
P05783	KRT18	Keratin, type I cytoskeletal 18	1.3	0.024	PRDX5
Q9Y3I0	RTCB	RNA-splicing ligase RtcB homolog	1.3	0.027	PRDX5
H3BPE1	MACF1	Microtubule-actin cross-linking factor 1, isoforms 1/2/3/5	1.3	0.034	PRDX5
Q13613	MTMR1	Myotubularin-related protein 1	1.3	0.039	PRDX5
Q01081	U2AF1	Splicing factor U2AF 35 kDa subunit	1.3	0.040	PRDX5
C9JZR2	CTNND1	Catenin delta-1	1.3	0.042	PRDX5
I3L2Z5	MAZ	Myc-associated zinc finger protein	1.3	0.049	PRDX5
P24752	ACAT1	Acetyl-CoA acetyltransferase, mitochondrial	1.2	0.000	PRDX5
P33240	CSTF2	Cleavage stimulation factor subunit 2	1.2	0.001	PRDX5
A0A087WW06	TTC28	Tetratricopeptide repeat protein 28	1.2	0.001	PRDX5
Q15126	PMVK	Phosphomevalonate kinase	1.2	0.001	PRDX5
P57740	NUP107	Nuclear pore complex protein Nup107	1.2	0.002	PRDX5
Q9P2R3	ANKFY1	Rabankyrin-5	1.2	0.002	PRDX5
Q16527	CSRP2	Cysteine and glycine-rich protein 2	1.2	0.003	PRDX5
Q8NCA5	FAM98A	Protein FAM98A	1.2	0.003	PRDX5
Q6P587	FAHD1	Acylpyruvase FAHD1, mitochondrial	1.2	0.004	PRDX5
A0A087WUK2	HNRNPDL	Heterogeneous nuclear ribonucleoprotein D-like	1.2	0.006	PRDX5
Q86Y56	DNAAF5	Dynein assembly factor 5, axonemal	1.2	0.007	PRDX5
P50213	IDH3A	Isocitrate dehydrogenase [NAD] subunit alpha, mitochondrial	1.2	0.008	PRDX5
Q15654	TRIP6	Thyroid receptor-interacting protein 6	1.2	0.009	PRDX5
P84103	SRSF3	Serine/arginine-rich splicing factor 3	1.2	0.013	PRDX5
Q9UNE7	STUB1	E3 ubiquitin-protein ligase CHIP	1.2	0.013	PRDX5
Q02241	KIF23	Kinesin-like protein KIF23	1.2	0.015	PRDX5
D6R938	CAMK2D	Calcium/calmodulin-dependent protein kinase	1.2	0.019	PRDX5
Q2TAL8	QRICH1	Glutamine-rich protein 1	1.2	0.021	PRDX5
Q13509	TUBB3	Tubulin beta-3 chain	1.2	0.023	PRDX5
Q9NQT5	EXOSC3	Exosome complex component RRP40	1.2	0.026	PRDX5
Q8N9T8	KRI1	Protein KRI1 homolog	1.2	0.030	PRDX5
P21912	SDHB	Succinate dehydrogenase [ubiquinone] iron-sulfur subunit, mitochondrial	1.2	0.039	PRDX5
Q8WWH5	TRUB1	Probable tRNA pseudouridine synthase 1	1.2	0.041	PRDX5
P09622	DLD	Dihydrolipoyl dehydrogenase, mitochondrial	1.1	0.000	PRDX5
P19525	EIF2AK2	Interferon-induced, double-stranded RNA-activated protein kinase	1.1	0.000	PRDX5
Q9Y5M8	SRPRB	Signal recognition particle receptor subunit beta	1.1	0.000	PRDX5
P39019	RPS19	40S ribosomal protein S19	1.1	0.000	PRDX5
Q9Y2Z4	YARS2	Tyrosine--tRNA ligase, mitochondrial	1.1	0.001	PRDX5
P58107	EPPK1	Epiplakin	1.1	0.002	PRDX5
Q9NY93	DDX56	Probable ATP-dependent RNA helicase DDX56	1.1	0.004	PRDX5
Q6P1N0	CC2D1A	Coiled-coil and C2 domain-containing protein 1A	1.1	0.005	PRDX5
P46778	RPL21	60S ribosomal protein L21	1.1	0.006	PRDX5
C9J9K3	RPSA	40S ribosomal protein SA	1.1	0.006	PRDX5
A0A2R8YDQ9	SUCLA2	Succinate--CoA ligase [ADP-forming] subunit beta, mitochondrial	1.1	0.006	PRDX5
O00442	RTCA	RNA 3'-terminal phosphate cyclase	1.1	0.008	PRDX5
P62937	PPIA	Peptidyl-prolyl cis-trans isomerase A	1.1	0.008	PRDX5
Q9P0K7	RAI14	Ankycorbin	1.1	0.011	PRDX5
Q5JSZ5	PRRC2B	Protein PRRC2B	1.1	0.012	PRDX5
Q02252	ALDH6A1	Methylmalonate-semialdehyde dehydrogenase [acylating], mitochondrial	1.1	0.014	PRDX5
Q9NQT4	EXOSC5	Exosome complex component RRP46	1.1	0.015	PRDX5
Q15750	TAB1	TGF-beta-activated kinase 1 and MAP3K7-binding protein 1	1.1	0.022	PRDX5
P50995	ANXA11	Annexin A11	1.1	0.022	PRDX5
Q92665	MRPS31	28S ribosomal protein S31, mitochondrial	1.1	0.023	PRDX5
Q12849	GRSF1	G-rich sequence factor 1	1.1	0.027	PRDX5
Q96F86	EDC3	Enhancer of mRNA-decapping protein 3	1.1	0.027	PRDX5
Q9UQ35	SRRM2	Serine/arginine repetitive matrix protein 2	1.1	0.027	PRDX5
Q9BWF3	RBM4	RNA-binding protein 4	1.1	0.028	PRDX5
Q96N67	DOCK7	Dedicator of cytokinesis protein 7	1.1	0.035	PRDX5
P00568	AK1	Adenylate kinase isoenzyme 1	1.1	0.036	PRDX5
P62266	RPS23	40S ribosomal protein S23	1.1	0.047	PRDX5
Q7KZ85	SUPT6H	Transcription elongation factor SPT6	1.1	0.048	PRDX5
P04637	TP53	Cellular tumor antigen p53	1.1	0.048	PRDX5
F2Z388	RPL35	60S ribosomal protein L35	1.1	0.049	PRDX5
P62081	RPS7	40S ribosomal protein S7	1.0	0.001	PRDX5
P22570	FDXR	NADPH:adrenodoxin oxidoreductase, mitochondrial	1.0	0.005	PRDX5
Q8WX93	PALLD	Palladin	1.0	0.007	PRDX5
P09525	ANXA4	Annexin A4	1.0	0.008	PRDX5
P35580	MYH10	Myosin-10	1.0	0.010	PRDX5
H7C5E4	XRN1	5'-3' exoribonuclease 1	1.0	0.011	PRDX5
Q13595	TRA2A	Transformer-2 protein homolog alpha	1.0	0.012	PRDX5
Q04837	SSBP1	Single-stranded DNA-binding protein, mitochondrial	1.0	0.015	PRDX5
Q12789	GTF3C1	General transcription factor 3C polypeptide 1	1.0	0.021	PRDX5
Q8WXF1	PSPC1	Paraspeckle component 1	1.0	0.022	PRDX5
P11172	UMPS	Uridine 5'-monophosphate synthase	1.0	0.025	PRDX5
E9PEJ4	DLAT	Acetyltransferase component of pyruvate dehydrogenase complex	1.0	0.026	PRDX5
Q9BQ69	MACROD1	ADP-ribose glycohydrolase MACROD1	1.0	0.028	PRDX5
O14965	AURKA	Aurora kinase A	1.0	0.031	PRDX5
O75691	UTP20	Small subunit processome component 20 homolog	1.0	0.038	PRDX5
P30520	ADSS2	Adenylosuccinate synthetase isozyme 2	1.0	0.039	PRDX5
D3YTB5	IRAK1	Interleukin-1 receptor-associated kinase 1	1.0	0.046	PRDX5
P45985	MAP2K4	Dual specificity mitogen-activated protein kinase kinase 4	1.0	0.049	PRDX5
**PRDX2 -specific interactors**					
P30048	PRDX3	Thioredoxin-dependent peroxide reductase, mitochondrial	5.8	0.000	PRDX1
P32119	PRDX2	Peroxiredoxin-2	5.7	0.000	PRDX1
Q9Y224	RTRAF	RNA transcription, translation and transport factor protein	5.4	0.000	PRDX1
P02790	HPX	Hemopexin	4.9	0.000	PRDX1
P62805	H4C1	Histone H4	4.1	0.000	PRDX1
O60506	SYNCRIP	Heterogeneous nuclear ribonucleoprotein Q	3.9	0.000	PRDX1
P40429	RPL13A	60S ribosomal protein L13a	3.9	0.000	PRDX1
P63167	DYNLL1	Dynein light chain 1, cytoplasmic	3.8	0.000	PRDX1
O76094	SRP72	Signal recognition particle subunit SRP72	3.7	0.000	PRDX1
P05386	RPLP1	60S acidic ribosomal protein P1	3.4	0.000	PRDX1
P00167	CYB5A	Cytochrome b5	3.3	0.000	PRDX1
O96007	MOCS2	Molybdopterin synthase catalytic subunit	3.3	0.000	PRDX1
E9PKG1	PRMT1	Protein arginine N-methyltransferase 1	3.2	0.000	PRDX1
P42704	LRPPRC	Leucine-rich PPR motif-containing protein, mitochondrial	3.2	0.000	PRDX1
P19338	NCL	Nucleolin	3.2	0.000	PRDX1
P63244	RACK1	Receptor of activated protein C kinase 1	3.0	0.000	PRDX1
Q9Y3D6	FIS1	Mitochondrial fission 1 protein	2.9	0.000	PRDX1
P62244	RPS15A	40S ribosomal protein S15a	2.9	0.000	PRDX1
P05387	RPLP2	60S acidic ribosomal protein P2	2.9	0.000	PRDX1
A0A0B4J1Z1	SRSF7	Serine/arginine-rich-splicing factor 7	2.9	0.001	PRDX1
Q6UW78	UQCC3	Ubiquinol-cytochrome-c reductase complex assembly factor 3	2.8	0.000	PRDX1
O00483	NDUFA4	Cytochrome c oxidase subunit NDUFA4	2.8	0.000	PRDX1
Q96CT7	CCDC124	Coiled-coil domain-containing protein 124	2.8	0.000	PRDX1
P61353	RPL27	60S ribosomal protein L27	2.7	0.000	PRDX1
Q92688	ANP32B	Acidic leucine-rich nuclear phosphoprotein 32 family member B	2.7	0.000	PRDX1
P61106	RAB14	Ras-related protein Rab-14	2.7	0.002	PRDX1
A0A3B3ISV3	COL4A1	Collagen alpha-1(IV) chain	2.6	0.000	PRDX1
P08758	ANXA5	Annexin A5	2.6	0.000	PRDX1
P68133	ACTA1	Actin, alpha skeletal muscle	2.6	0.004	PRDX1
Q9NX55	HYPK	Huntingtin-interacting protein K	2.6	0.006	PRDX1
D6RA00	ENOPH1	Enolase-phosphatase E1	2.5	0.000	PRDX1
B9A018	USP39	U4/U6.U5 tri-snRNP-associated protein 2	2.5	0.000	PRDX1
Q92769	HDAC2	Histone deacetylase 2	2.5	0.000	PRDX1
P12236	SLC25A6	ADP/ATP translocase 3	2.5	0.000	PRDX1
P26196	DDX6	Probable ATP-dependent RNA helicase DDX6	2.5	0.000	PRDX1
P35637	FUS	RNA-binding protein FUS	2.5	0.001	PRDX1
P18077	RPL35A	60S ribosomal protein L35a	2.4	0.000	PRDX1
P09211	GSTP1	Glutathione S-transferase P	2.4	0.000	PRDX1
G5EA06	MRPS27	28S ribosomal protein S27, mitochondrial	2.4	0.002	PRDX1
P53602	MVD	Diphosphomevalonate decarboxylase	2.4	0.002	PRDX1
Q71UI9	H2AZ2	Histone H2A.V	2.3	0.000	PRDX1
Q9H0U4	RAB1B	Ras-related protein Rab-1B	2.3	0.000	PRDX1
P36957	DLST	Dihydrolipoyllysine-residue succinyltransferase component of 2-oxoglutarate dehydrogenase complex, mitochondrial	2.3	0.000	PRDX1
P62750	RPL23A	60S ribosomal protein L23a	2.3	0.000	PRDX1
C9J4Z3	RPL37A	60S ribosomal protein L37a	2.3	0.000	PRDX1
P27797	CALR	Calreticulin	2.3	0.000	PRDX1
P15121	AKR1B1	Aldo-keto reductase family 1 member B1	2.3	0.000	PRDX1
Q53GQ0	HSD17B12	Very-long-chain 3-oxoacyl-CoA reductase	2.3	0.002	PRDX1
O75223	GGCT	Gamma-glutamylcyclotransferase	2.3	0.002	PRDX1
P00492	HPRT1	Hypoxanthine-guanine phosphoribosyltransferase	2.3	0.004	PRDX1
P12955	PEPD	Xaa-Pro dipeptidase	2.3	0.007	PRDX1
A0A087WTM1	ROBO1	Roundabout homolog 1	2.2	0.000	PRDX1
Q9H8Y8	GORASP2	Golgi reassembly-stacking protein 2	2.2	0.000	PRDX1
P50914	RPL14	60S ribosomal protein L14	2.2	0.000	PRDX1
P32969	RPL9	60S ribosomal protein L9	2.2	0.000	PRDX1
P51149	RAB7A	Ras-related protein Rab-7a	2.2	0.000	PRDX1
P19174	PLCG1	1-phosphatidylinositol 4,5-bisphosphate phosphodiesterase gamma-1	2.2	0.002	PRDX1
O15144	ARPC2	Actin-related protein 2/3 complex subunit 2	2.2	0.002	PRDX1
H3BRL3	UBFD1	Ubiquitin domain-containing protein UBFD1	2.2	0.003	PRDX1
Q13126	MTAP	S-methyl-5'-thioadenosine phosphorylase	2.2	0.004	PRDX1
P35237	SERPINB6	Serpin B6	2.1	0.000	PRDX1
P10301	RRAS	Ras-related protein R-Ras	2.1	0.000	PRDX1
P30740	SERPINB1	Leukocyte elastase inhibitor	2.1	0.000	PRDX1
Q15393	SF3B3	Splicing factor 3B subunit 3	2.1	0.000	PRDX1
Q9NS69	TOMM22	Mitochondrial import receptor subunit TOM22 homolog	2.1	0.000	PRDX1
Q02750	MAP2K1	Dual specificity mitogen-activated protein kinase kinase 1	2.1	0.000	PRDX1
P49321	NASP	Nuclear autoantigenic sperm protein	2.1	0.000	PRDX1
P23921	RRM1	Ribonucleoside-diphosphate reductase large subunit	2.1	0.000	PRDX1
Q9ULC3	RAB23	Ras-related protein Rab-23	2.1	0.002	PRDX1
O75400	PRPF40A	Pre-mRNA-processing factor 40 homolog A	2.1	0.003	PRDX1
O95104	SCAF4	SR-related and CTD-associated factor 4	2.1	0.003	PRDX1
O60547	GMDS	GDP-mannose 4,6 dehydratase	2.1	0.015	PRDX1
O00231	PSMD11	26S proteasome non-ATPase regulatory subunit 11	2.0	0.000	PRDX1
O43747	AP1G1	AP-1 complex subunit gamma-1	2.0	0.000	PRDX1
O95433	AHSA1	Activator of 90 kDa heat shock protein ATPase homolog 1	2.0	0.000	PRDX1
Q02878	RPL6	60S ribosomal protein L6	2.0	0.000	PRDX1
P15880	RPS2	40S ribosomal protein S2	2.0	0.000	PRDX1
P06730	EIF4E	Eukaryotic translation initiation factor 4E	2.0	0.000	PRDX1
P05388	RPLP0	60S acidic ribosomal protein P0	2.0	0.000	PRDX1
K7ERF1	EIF3K	Eukaryotic translation initiation factor 3 subunit K	2.0	0.000	PRDX1
P35606	COPB2	Coatomer subunit beta'	2.0	0.001	PRDX1
P11182	DBT	Lipoamide acyltransferase component of branched-chain alpha-keto acid dehydrogenase complex, mitochondrial	2.0	0.003	PRDX1
Q5SY16	NOL9	Polynucleotide 5'-hydroxyl-kinase NOL9	2.0	0.004	PRDX1
Q9Y570	PPME1	Protein phosphatase methylesterase 1	2.0	0.005	PRDX1
P00491	PNP	Purine nucleoside phosphorylase	2.0	0.005	PRDX1
P48147	PREP	Prolyl endopeptidase	2.0	0.010	PRDX1
A0AVT1	UBA6	Ubiquitin-like modifier-activating enzyme 6	2.0	0.021	PRDX1
P04181	OAT	Ornithine aminotransferase, mitochondrial	1.9	0.000	PRDX1
Q14232	EIF2B1	Translation initiation factor eIF-2B subunit alpha	1.9	0.000	PRDX1
Q9UJS0	SLC25A13	Calcium-binding mitochondrial carrier protein Aralar2	1.9	0.000	PRDX1
P53985	SLC16A1	Monocarboxylate transporter 1	1.9	0.000	PRDX1
O75489	NDUFS3	NADH dehydrogenase [ubiquinone] iron-sulfur protein 3, mitochondrial	1.9	0.005	PRDX1
P84085	ARF5	ADP-ribosylation factor 5	1.9	0.012	PRDX1
E9PHS0	LANCL1	Glutathione S-transferase LANCL1	1.9	0.021	PRDX1
O60664	PLIN3	Perilipin-3	1.9	0.024	PRDX1
Q7Z434	MAVS	Mitochondrial antiviral-signaling protein	1.9	0.036	PRDX1
P10599	TXN	Thioredoxin	1.9	0.043	PRDX1
Q9UBF2	COPG2	Coatomer subunit gamma-2	1.8	0.000	PRDX1
Q15691	MAPRE1	Microtubule-associated protein RP/EB family member 1	1.8	0.000	PRDX1
A6NKB8	RNPEP	Aminopeptidase B	1.8	0.000	PRDX1
P00387	CYB5R3	NADH-cytochrome b5 reductase 3	1.8	0.000	PRDX1
P23396	RPS3	40S ribosomal protein S3	1.8	0.000	PRDX1
P41252	IARS1	Isoleucine--tRNA ligase, cytoplasmic	1.8	0.001	PRDX1
O14980	XPO1	Exportin-1	1.8	0.002	PRDX1
Q4J6C6	PREPL	Prolyl endopeptidase-like	1.8	0.006	PRDX1
P11388	TOP2A	DNA topoisomerase 2-alpha	1.8	0.007	PRDX1
Q9NYB0	TERF2IP	Telomeric repeat-binding factor 2-interacting protein 1	1.8	0.007	PRDX1
P28288	ABCD3	ATP-binding cassette sub-family D member 3	1.8	0.009	PRDX1
E7ETZ4	BZW2	Basic leucine zipper and W2 domain-containing protein 2	1.8	0.010	PRDX1
O94925	GLS	Glutaminase kidney isoform, mitochondrial	1.8	0.012	PRDX1
E9PHN7	GSTM2	Glutathione S-transferase Mu 2	1.8	0.014	PRDX1
Q9NVP1	DDX18	ATP-dependent RNA helicase DDX18	1.8	0.015	PRDX1
P40616	ARL1	ADP-ribosylation factor-like protein 1	1.8	0.025	PRDX1
P38159	RBMX	RNA-binding motif protein, X chromosome	1.8	0.037	PRDX1
Q9P2B4	CTTNBP2NL	CTTNBP2 N-terminal-like protein	1.7	0.000	PRDX1
Q6PI48	DARS2	Aspartate--tRNA ligase, mitochondrial	1.7	0.000	PRDX1
P36542	ATP5F1C	ATP synthase subunit gamma, mitochondrial	1.7	0.000	PRDX1
P05141	SLC25A5	ADP/ATP translocase 2	1.7	0.000	PRDX1
Q5T7C4	HMGB1	High mobility group protein B1	1.7	0.001	PRDX1
P22314	UBA1	Ubiquitin-like modifier-activating enzyme 1	1.7	0.004	PRDX1
O00232	PSMD12	26S proteasome non-ATPase regulatory subunit 12	1.7	0.004	PRDX1
P27824	CANX	Calnexin	1.7	0.005	PRDX1
Q9BQG0	MYBBP1A	Myb-binding protein 1A	1.7	0.007	PRDX1
Q9ULW0	TPX2	Targeting protein for Xklp2	1.7	0.008	PRDX1
Q01085	TIAL1	Nucleolysin TIAR	1.7	0.010	PRDX1

P51398	DAP3	28S ribosomal protein S29, mitochondrial	1.7	0.014	PRDX1
Q96IU4	ABHD14B	Protein ABHD14B	1.7	0.016	PRDX1
O95163	ELP1	Elongator complex protein 1	1.7	0.017	PRDX1
Q9UBS4	DNAJB11	DnaJ homolog subfamily B member 11	1.7	0.018	PRDX1
P31153	MAT2A	S-adenosylmethionine synthase isoform type-2	1.6	0.000	PRDX1
P05455	SSB	Lupus La protein	1.6	0.000	PRDX1
P61313	RPL15	60S ribosomal protein L15	1.6	0.000	PRDX1
P51665	PSMD7	26S proteasome non-ATPase regulatory subunit 7	1.6	0.000	PRDX1
G8JLH9	STAT3	Signal transducer and activator of transcription	1.6	0.000	PRDX1
P53007	SLC25A1	Tricarboxylate transport protein, mitochondrial	1.6	0.000	PRDX1
O75821	EIF3G	Eukaryotic translation initiation factor 3 subunit G	1.6	0.000	PRDX1
P16435	POR	NADPH--cytochrome P450 reductase	1.6	0.003	PRDX1
P39023	RPL3	60S ribosomal protein L3	1.6	0.004	PRDX1
Q8TEX9	IPO4	Importin-4	1.6	0.006	PRDX1
Q8N3X1	FNBP4	Formin-binding protein 4	1.6	0.018	PRDX1
Q9Y4P1	ATG4B	Cysteine protease ATG4B	1.6	0.021	PRDX1
P00390	GSR	Glutathione reductase, mitochondrial	1.6	0.025	PRDX1
Q13542	EIF4EBP2	Eukaryotic translation initiation factor 4E-binding protein 2	1.6	0.025	PRDX1
Q5T4U8	RABGGTB	Geranylgeranyl transferase type-2 subunit beta	1.6	0.033	PRDX1
A6NGP5	JPT2	Jupiter microtubule-associated homolog 2	1.6	0.033	PRDX1
P51991	HNRNPA3	Heterogeneous nuclear ribonucleoprotein A3	1.6	0.037	PRDX1
Q8N806	UBR7	Putative E3 ubiquitin-protein ligase UBR7	1.6	0.047	PRDX1
P43304	GPD2	Glycerol-3-phosphate dehydrogenase, mitochondrial	1.6	0.048	PRDX1
Q9BYN0	SRXN1	Sulfiredoxin-1	1.6	0.048	PRDX1
Q6IBS0	TWF2	Twinfilin-2	1.5	0.000	PRDX1
Q6IQ49	SDE2	Replication stress response regulator SDE2	1.5	0.000	PRDX1
P38117	ETFB	Electron transfer flavoprotein subunit beta	1.5	0.000	PRDX1
Q9UQ80	PA2G4	Proliferation-associated protein 2G4	1.5	0.000	PRDX1
P61586	RHOA	Transforming protein RhoA	1.5	0.000	PRDX1
P43034	PAFAH1B1	Platelet-activating factor acetylhydrolase IB subunit beta	1.5	0.000	PRDX1
O00487	PSMD14	26S proteasome non-ATPase regulatory subunit 14	1.5	0.001	PRDX1
Q53H12	AGK	Acylglycerol kinase, mitochondrial	1.5	0.002	PRDX1
Q16531	DDB1	DNA damage-binding protein 1	1.5	0.002	PRDX1
Q9GZS3	WDR61	WD repeat-containing protein 61	1.5	0.003	PRDX1
P30101	PDIA3	Protein disulfide-isomerase A3	1.5	0.003	PRDX1
Q9NPH2	ISYNA1	Inositol-3-phosphate synthase 1	1.5	0.004	PRDX1
P52597	HNRNPF	Heterogeneous nuclear ribonucleoprotein F	1.5	0.004	PRDX1
Q5VW32	BROX	BRO1 domain-containing protein BROX	1.5	0.007	PRDX1
Q96IZ0	PAWR	PRKC apoptosis WT1 regulator protein	1.5	0.009	PRDX1
Q92621	NUP205	Nuclear pore complex protein Nup205	1.5	0.012	PRDX1
P26599	PTBP1	Polypyrimidine tract-binding protein 1	1.5	0.018	PRDX1
H3BV80	RNPS1	RNA-binding protein with serine-rich domain 1	1.5	0.023	PRDX1
Q8NI27	THOC2	THO complex subunit 2	1.5	0.024	PRDX1
P42574	CASP3	Caspase-3	1.5	0.026	PRDX1
Q9P287	BCCIP	BRCA2 and CDKN1A-interacting protein	1.5	0.029	PRDX1
E9PGT1	TSN	Component 3 of promoter of RISC	1.5	0.031	PRDX1
Q9BSJ8	ESYT1	Extended synaptotagmin-1	1.5	0.032	PRDX1
Q9H9P8	L2HGDH	L-2-hydroxyglutarate dehydrogenase, mitochondrial	1.5	0.033	PRDX1
Q8NFC6	BOD1L1	Biorientation of chromosomes in cell division protein 1-like 1	1.5	0.034	PRDX1
P07384	CAPN1	Calpain-1 catalytic subunit	1.5	0.037	PRDX1
Q9Y6Y0	IVNS1ABP	Influenza virus NS1A-binding protein	1.5	0.037	PRDX1
Q9Y277	VDAC3	Voltage-dependent anion-selective channel protein 3	1.5	0.043	PRDX1
P27694	RPA1	Replication protein A 70 kDa DNA-binding subunit	1.5	0.046	PRDX1
Q92973	TNPO1	Transportin-1	1.4	0.000	PRDX1
P23528	CFL1	Cofilin-1	1.4	0.000	PRDX1
P61247	RPS3A	40S ribosomal protein S3a	1.4	0.000	PRDX1
E9PN17	ATP5MG	ATP synthase subunit g, mitochondrial	1.4	0.001	PRDX1
A0A024R4M0	RPS9	40S ribosomal protein S9	1.4	0.001	PRDX1
O00203	AP3B1	AP-3 complex subunit beta-1	1.4	0.001	PRDX1
P60953	CDC42	Cell division control protein 42 homolog	1.4	0.001	PRDX1
P08133	ANXA6	Annexin A6	1.4	0.003	PRDX1
P16615	ATP2A2	Sarcoplasmic/endoplasmic reticulum calcium ATPase 2	1.4	0.004	PRDX1
Q96SB4	SRPK1	SRSF protein kinase 1	1.4	0.005	PRDX1
Q5T4K5	CRTC2	CREB-regulated transcription coactivator 2	1.4	0.006	PRDX1
G3V529	DDX24	RNA helicase	1.4	0.019	PRDX1
Q9UHQ9	CYB5R1	NADH-cytochrome b5 reductase 1	1.4	0.037	PRDX1
Q9Y2R5	MRPS17	28S ribosomal protein S17, mitochondrial	1.4	0.037	PRDX1
Q9BZK7	TBL1XR1	F-box-like/WD repeat-containing protein TBL1XR1	1.4	0.037	PRDX1
O96019	ACTL6A	Actin-like protein 6A	1.4	0.039	PRDX1
Q5JR04	MOV10	RNA helicase	1.4	0.039	PRDX1
K7EM73	CAPNS1	Calcium-activated neutral proteinase small subunit	1.4	0.040	PRDX1
O95394	PGM3	Phosphoacetylglucosamine mutase	1.3	0.000	PRDX1
P68104	EEF1A1	Elongation factor 1-alpha 1	1.3	0.000	PRDX1
P00338	LDHA	L-lactate dehydrogenase A chain	1.3	0.000	PRDX1
P06737	PYGL	Glycogen phosphorylase, liver form	1.3	0.000	PRDX1
P36578	RPL4	60S ribosomal protein L4	1.3	0.000	PRDX1
P08238	HSP90AB1	Heat shock protein HSP 90-beta	1.3	0.000	PRDX1
P52565	ARHGDIA	Rho GDP-dissociation inhibitor 1	1.3	0.001	PRDX1
E7ESY4	MTA1	Metastasis-associated protein MTA1	1.3	0.001	PRDX1
B4DLN1	B4DLN1	cDNA FLJ60124, highly similar to Mitochondrial dicarboxylate carrier	1.3	0.001	PRDX1
Q9H7D7	WDR26	WD repeat-containing protein 26	1.3	0.002	PRDX1
P61923	COPZ1	Coatomer subunit zeta-1	1.3	0.002	PRDX1
P62269	RPS18	40S ribosomal protein S18	1.3	0.002	PRDX1
Q96HC4	PDLIM5	PDZ and LIM domain protein 5	1.3	0.002	PRDX1
A0A3B3IRI2	CTPS1	CTP synthase	1.3	0.004	PRDX1
E7EV99	ADD1	Alpha-adducin	1.3	0.004	PRDX1
P56192	MARS1	Methionine--tRNA ligase, cytoplasmic	1.3	0.005	PRDX1
Q9Y678	COPG1	Coatomer subunit gamma-1	1.3	0.006	PRDX1
O75832	PSMD10	26S proteasome non-ATPase regulatory subunit 10	1.3	0.007	PRDX1
X1WI28	RPL10	60S ribosomal protein L10	1.3	0.008	PRDX1
P50502	ST13	Hsc70-interacting protein	1.3	0.013	PRDX1
Q9H0A0	NAT10	RNA cytidine acetyltransferase	1.3	0.014	PRDX1
A0A0A0MRM9	NOLC1	Nucleolar and coiled-body phosphoprotein 1	1.3	0.025	PRDX1
Q8N9Q2	SREK1IP1	Protein SREK1IP1	1.3	0.026	PRDX1
P52888	THOP1	Thimet oligopeptidase	1.3	0.032	PRDX1
Q9NUP9	LIN7C	Protein lin-7 homolog C	1.3	0.035	PRDX1
Q93008	USP9X	Probable ubiquitin carboxyl-terminal hydrolase FAF-X	1.3	0.037	PRDX1
Q9Y262	EIF3L	Eukaryotic translation initiation factor 3 subunit L	1.3	0.043	PRDX1
Q96BW9	TAMM41	Phosphatidate cytidylyltransferase, mitochondrial	1.3	0.043	PRDX1
F5GZS6	SLC3A2	4F2 cell-surface antigen heavy chain	1.3	0.047	PRDX1
P49756	RBM25	RNA-binding protein 25	1.2	0.000	PRDX1
P61221	ABCE1	ATP-binding cassette sub-family E member 1	1.2	0.000	PRDX1
Q9H3P7	ACBD3	Golgi resident protein GCP60	1.2	0.001	PRDX1
Q07020	RPL18	60S ribosomal protein L18	1.2	0.001	PRDX1
Q9NQW7	XPNPEP1	Xaa-Pro aminopeptidase 1	1.2	0.002	PRDX1
P55884	EIF3B	Eukaryotic translation initiation factor 3 subunit B	1.2	0.004	PRDX1
P62241	RPS8	40S ribosomal protein S8	1.2	0.005	PRDX1
P49736	MCM2	DNA replication licensing factor MCM2	1.2	0.005	PRDX1
P53618	COPB1	Coatomer subunit beta	1.2	0.005	PRDX1
O75531	BANF1	Barrier-to-autointegration factor	1.2	0.007	PRDX1
P15927	RPA2	Replication protein A 32 kDa subunit	1.2	0.011	PRDX1
P11387	TOP1	DNA topoisomerase 1	1.2	0.012	PRDX1
P15374	UCHL3	Ubiquitin carboxyl-terminal hydrolase isozyme L3	1.2	0.012	PRDX1
Q5QPM7	PSMF1	Proteasome inhibitor PI31 subunit	1.2	0.017	PRDX1
P49750	YLPM1	YLP motif-containing protein 1	1.2	0.019	PRDX1
P20073	ANXA7	Annexin A7	1.2	0.025	PRDX1
Q00610	CLTC	Clathrin heavy chain 1	1.2	0.026	PRDX1
P33992	MCM5	DNA replication licensing factor MCM5	1.2	0.029	PRDX1
P31689	DNAJA1	DnaJ homolog subfamily A member 1	1.2	0.031	PRDX1
A0A0A0MRN5	OGFR	Opioid growth factor receptor	1.2	0.040	PRDX1
O15269	SPTLC1	Serine palmitoyltransferase 1	1.2	0.043	PRDX1
P82663	MRPS25	28S ribosomal protein S25, mitochondrial	1.1	0.000	PRDX1
P62424	RPL7A	60S ribosomal protein L7a	1.1	0.000	PRDX1
P07900	HSP90AA1	Heat shock protein HSP 90-alpha	1.1	0.000	PRDX1
Q00325	SLC25A3	Phosphate carrier protein, mitochondrial	1.1	0.000	PRDX1
P34949	MPI	Mannose-6-phosphate isomerase	1.1	0.001	PRDX1
Q8NE71	ABCF1	ATP-binding cassette sub-family F member 1	1.1	0.001	PRDX1
E5RHG8	ELOC	Elongin-C	1.1	0.001	PRDX1
Q9Y5P6	GMPPB	Mannose-1-phosphate guanyltransferase beta	1.1	0.002	PRDX1
Q8WUH6	TMEM263	Transmembrane protein 263	1.1	0.002	PRDX1
P14324	FDPS	Farnesyl pyrophosphate synthase	1.1	0.002	PRDX1
Q15029	EFTUD2	116 kDa U5 small nuclear ribonucleoprotein component	1.1	0.003	PRDX1
Q9UMS4	PRPF19	Pre-mRNA-processing factor 19	1.1	0.003	PRDX1
Q8TAQ2	SMARCC2	SWI/SNF complex subunit SMARCC2	1.1	0.004	PRDX1
P43246	MSH2	DNA mismatch repair protein Msh2	1.1	0.005	PRDX1
Q99460	PSMD1	26S proteasome non-ATPase regulatory subunit 1	1.1	0.006	PRDX1
Q9UNM6	PSMD13	26S proteasome non-ATPase regulatory subunit 13	1.1	0.009	PRDX1
P53004	BLVRA	Biliverdin reductase A	1.1	0.012	PRDX1
Q9UI12	ATP6V1H	V-type proton ATPase subunit H	1.1	0.013	PRDX1
Q9Y3Z3	SAMHD1	Deoxynucleoside triphosphate triphosphohydrolase SAMHD1	1.1	0.013	PRDX1
Q5T760	SRSF11	Serine/arginine-rich-splicing factor 11	1.1	0.019	PRDX1
F8W6I7	HNRNPA1	Helix-destabilizing protein	1.1	0.026	PRDX1
P62333	PSMC6	26S proteasome regulatory subunit 10B	1.1	0.030	PRDX1
P34932	HSPA4	Heat shock 70 kDa protein 4	1.1	0.032	PRDX1
P26583	HMGB2	High mobility group protein B2	1.1	0.039	PRDX1
P42224	STAT1	Signal transducer and activator of transcription 1-alpha/beta	1.1	0.046	PRDX1
P62314	SNRPD1	Small nuclear ribonucleoprotein Sm D1	1.1	0.049	PRDX1
P60891	PRPS1	Ribose-phosphate pyrophosphokinase 1	1.0	0.000	PRDX1
O75396	SEC22B	Vesicle-trafficking protein SEC22b	1.0	0.004	PRDX1
Q9BT78	COPS4	COP9 signalosome complex subunit 4	1.0	0.005	PRDX1
Q14240	EIF4A2	Eukaryotic initiation factor 4A-II	1.0	0.011	PRDX1
O94903	PLPBP	Pyridoxal phosphate homeostasis protein	1.0	0.020	PRDX1
Q8IWB7	WDFY1	WD repeat and FYVE domain-containing protein 1	1.0	0.020	PRDX1
O00273	DFFA	DNA fragmentation factor subunit alpha	1.0	0.034	PRDX1
P55769	SNU13	NHP2-like protein 1	1.0	0.038	PRDX1
Q8NHH9	ATL2	Atlastin-2	1.0	0.039	PRDX1
O14965	AURKA	Aurora kinase A	1.0	0.044	PRDX1
Q8IYS1	PM20D2	Peptidase M20 domain-containing protein 2	1.0	0.046	PRDX1
P32119	PRDX2	Peroxiredoxin-2	6.8	0.000	PRDX3
P00167	CYB5A	Cytochrome b5	4.4	0.000	PRDX3
O96007	MOCS2	Molybdopterin synthase catalytic subunit	3.7	0.000	PRDX3
P10301	RRAS	Ras-related protein R-Ras	3.7	0.000	PRDX3
Q9Y224	RTRAF	RNA transcription, translation and transport factor protein	3.7	0.000	PRDX3
Q9Y3D6	FIS1	Mitochondrial fission 1 protein	3.2	0.000	PRDX3
Q96CT7	CCDC124	Coiled-coil domain-containing protein 124	3.2	0.000	PRDX3
Q8NFC6	BOD1L1	Biorientation of chromosomes in cell division protein 1-like 1	3.0	0.000	PRDX3
Q9Y6A5	TACC3	Transforming acidic coiled-coil-containing protein 3	3.0	0.001	PRDX3
Q9NX55	HYPK	Huntingtin-interacting protein K	2.9	0.003	PRDX3
E7EVA0	MAP4	Microtubule-associated protein	2.9	0.005	PRDX3
O95831	AIFM1	Apoptosis-inducing factor 1, mitochondrial	2.8	0.000	PRDX3
P53602	MVD	Diphosphomevalonate decarboxylase	2.8	0.001	PRDX3
Q6WKZ4	RAB11FIP1	Rab11 family-interacting protein 1	2.8	0.001	PRDX3
Q6IQ49	SDE2	Replication stress response regulator SDE2	2.7	0.000	PRDX3
Q9ULW0	TPX2	Targeting protein for Xklp2	2.7	0.001	PRDX3
Q96A10	ERVK3-1	Endogenous retrovirus group K3 member 1	2.7	0.024	PRDX3
H0YNW5	DUT	Deoxyuridine 5'-triphosphate nucleotidohydrolase	2.6	0.000	PRDX3
Q9NPH2	ISYNA1	Inositol-3-phosphate synthase 1	2.6	0.000	PRDX3
Q13542	EIF4EBP2	Eukaryotic translation initiation factor 4E-binding protein 2	2.6	0.002	PRDX3
P12955	PEPD	Xaa-Pro dipeptidase	2.6	0.004	PRDX3
Q9C0C2	TNKS1BP1	182 kDa tankyrase-1-binding protein	2.6	0.007	PRDX3
O60664	PLIN3	Perilipin-3	2.5	0.002	PRDX3
Q6PID6	TTC33	Tetratricopeptide repeat protein 33	2.5	0.034	PRDX3
H3BRL3	UBFD1	Ubiquitin domain-containing protein UBFD1	2.4	0.000	PRDX3
P47985	UQCRFS1	Cytochrome b-c1 complex subunit Rieske, mitochondrial	2.4	0.000	PRDX3
B7Z7F3	RANBP3	Ran-binding protein 3	2.4	0.001	PRDX3
P00491	PNP	Purine nucleoside phosphorylase	2.4	0.002	PRDX3
Q8N3X1	FNBP4	Formin-binding protein 4	2.3	0.000	PRDX3
Q9P2B4	CTTNBP2NL	CTTNBP2 N-terminal-like protein	2.3	0.000	PRDX3
Q9NUQ3	TXLNG	Gamma-taxilin	2.3	0.000	PRDX3
Q8NC51	SERBP1	Plasminogen activator inhibitor 1 RNA-binding protein	2.3	0.001	PRDX3
A4D1S0	KLRG2	Killer cell lectin-like receptor subfamily G member 2	2.3	0.003	PRDX3
P11802	CDK4	Cyclin-dependent kinase 4	2.3	0.004	PRDX3
P35244	RPA3	Replication protein A 14 kDa subunit	2.3	0.004	PRDX3
Q9P258	RCC2	Protein RCC2	2.2	0.001	PRDX3
Q7Z4V5	HDGFL2	Hepatoma-derived growth factor-related protein 2	2.2	0.012	PRDX3
E7EV99	ADD1	Alpha-adducin	2.1	0.000	PRDX3
O14965	AURKA	Aurora kinase A	2.1	0.000	PRDX3
Q12948	FOXC1	Forkhead box protein C1	2.1	0.001	PRDX3
P14174	MIF	Macrophage migration inhibitory factor	2.1	0.010	PRDX3
A0A087WTM1	ROBO1	Roundabout homolog 1	2.0	0.000	PRDX3
F5GZS6	SLC3A2	4F2 cell-surface antigen heavy chain	2.0	0.006	PRDX3
P61758	VBP1	Prefoldin subunit 3	2.0	0.006	PRDX3
A6NGP5	JPT2	Jupiter microtubule-associated homolog 2	2.0	0.007	PRDX3
P00390	GSR	Glutathione reductase, mitochondrial	2.0	0.009	PRDX3
O15511	ARPC5	Actin-related protein 2/3 complex subunit 5	2.0	0.014	PRDX3
E9PDD6	BCL2L13	Bcl-2-like protein 13	2.0	0.020	PRDX3
P22234	PAICS	Multifunctional protein ADE2 [Includes: Phosphoribosylaminoimidazole-succinocarboxamide synthase	1.9	0.002	PRDX3
P41250	GARS1	Glycine--tRNA ligase	1.9	0.004	PRDX3
Q13541	EIF4EBP1	Eukaryotic translation initiation factor 4E-binding protein 1	1.9	0.010	PRDX3
Q9Y266	NUDC	Nuclear migration protein nudC	1.9	0.014	PRDX3
Q8IVM0	CCDC50	Coiled-coil domain-containing protein 50	1.9	0.014	PRDX3
J3KS31	ZNF207	BUB3-interacting and GLEBS motif-containing protein ZNF207	1.9	0.032	PRDX3
A0A1B0GUC3	FTO	Alpha-ketoglutarate-dependent dioxygenase FTO	1.8	0.025	PRDX3
P40222	TXLNA	Alpha-taxilin	1.7	0.002	PRDX3
P61927	RPL37	60S ribosomal protein L37	1.7	0.005	PRDX3
Q6UW78	UQCC3	Ubiquinol-cytochrome-c reductase complex assembly factor 3	1.7	0.010	PRDX3
A0AVT1	UBA6	Ubiquitin-like modifier-activating enzyme 6	1.7	0.022	PRDX3
Q96KB5	PBK	Lymphokine-activated killer T-cell-originated protein kinase	1.7	0.027	PRDX3
P13995	MTHFD2	Bifunctional methylenetetrahydrofolate dehydrogenase/cyclohydrolase, mitochondrial [Includes: NAD-dependent methylenetetrahydrofolate dehydrogenase	1.7	0.029	PRDX3
O94903	PLPBP	Pyridoxal phosphate homeostasis protein	1.6	0.000	PRDX3
P26599	PTBP1	Polypyrimidine tract-binding protein 1	1.6	0.012	PRDX3
Q99598	TSNAX	Translin-associated protein X	1.6	0.014	PRDX3
Q9UHR5	SAP30BP	SAP30-binding protein	1.6	0.020	PRDX3
Q13126	MTAP	S-methyl-5'-thioadenosine phosphorylase	1.6	0.030	PRDX3
E9PGZ1	CALD1	Caldesmon	1.6	0.036	PRDX3
Q15181	PPA1	Inorganic pyrophosphatase	1.5	0.000	PRDX3
Q7Z2K8	GPRIN1	G protein-regulated inducer of neurite outgrowth 1	1.5	0.001	PRDX3
P31153	MAT2A	S-adenosylmethionine synthase isoform type-2	1.5	0.001	PRDX3
P62244	RPS15A	40S ribosomal protein S15a	1.5	0.003	PRDX3
Q71UI9	H2AZ2	Histone H2A.V	1.5	0.018	PRDX3
P85037	FOXK1	Forkhead box protein K1	1.5	0.022	PRDX3
Q9HA64	FN3KRP	Ketosamine-3-kinase	1.5	0.030	PRDX3
A0A087WUT6	EIF5B	Eukaryotic translation initiation factor 5B	1.4	0.001	PRDX3
Q5T4K5	CRTC2	CREB-regulated transcription coactivator 2	1.4	0.014	PRDX3
Q5T6F2	UBAP2	Ubiquitin-associated protein 2	1.4	0.018	PRDX3
Q7L2J0	MEPCE	7SK snRNA methylphosphate capping enzyme	1.4	0.021	PRDX3
Q9NUP9	LIN7C	Protein lin-7 homolog C	1.4	0.026	PRDX3
P52888	THOP1	Thimet oligopeptidase	1.4	0.028	PRDX3
Q13137	CALCOCO2	Calcium-binding and coiled-coil domain-containing protein 2	1.4	0.028	PRDX3
P49903	SEPHS1	Selenide, water dikinase 1	1.4	0.031	PRDX3
Q16204	CCDC6	Coiled-coil domain-containing protein 6	1.4	0.034	PRDX3
E9PKG1	PRMT1	Protein arginine N-methyltransferase 1	1.3	0.000	PRDX3
P50914	RPL14	60S ribosomal protein L14	1.3	0.001	PRDX3
Q02878	RPL6	60S ribosomal protein L6	1.3	0.008	PRDX3
Q6PKG0	LARP1	La-related protein 1	1.3	0.008	PRDX3
Q16576	RBBP7	Histone-binding protein RBBP7	1.3	0.011	PRDX3
Q9BQ61	TRIR	Telomerase RNA component interacting RNase	1.3	0.014	PRDX3
Q8N9Q2	SREK1IP1	Protein SREK1IP1	1.3	0.015	PRDX3
Q9GZS3	WDR61	WD repeat-containing protein 61	1.3	0.015	PRDX3
Q96F86	EDC3	Enhancer of mRNA-decapping protein 3	1.3	0.018	PRDX3
P62266	RPS23	40S ribosomal protein S23	1.3	0.020	PRDX3
P56385	ATP5ME	ATP synthase subunit e, mitochondrial	1.3	0.025	PRDX3
P48147	PREP	Prolyl endopeptidase	1.3	0.026	PRDX3
Q96IZ0	PAWR	PRKC apoptosis WT1 regulator protein	1.3	0.032	PRDX3
Q9Y570	PPME1	Protein phosphatase methylesterase 1	1.3	0.038	PRDX3
Q32MZ4	LRRFIP1	Leucine-rich repeat flightless-interacting protein 1	1.3	0.039	PRDX3
P40429	RPL13A	60S ribosomal protein L13a	1.2	0.001	PRDX3
P07195	LDHB	L-lactate dehydrogenase B chain	1.2	0.003	PRDX3
P34897	SHMT2	Serine hydroxymethyltransferase, mitochondrial	1.2	0.004	PRDX3
Q9BRP1	PDCD2L	Programmed cell death protein 2-like	1.2	0.006	PRDX3
J3KN29	PSMD9	26S proteasome non-ATPase regulatory subunit 9	1.2	0.011	PRDX3
P62851	RPS25	40S ribosomal protein S25	1.2	0.015	PRDX3
Q9H0U4	RAB1B	Ras-related protein Rab-1B	1.2	0.023	PRDX3
B4DDF4	CNN2	Calponin	1.2	0.031	PRDX3
O14949	UQCRQ	Cytochrome b-c1 complex subunit 8	1.2	0.046	PRDX3
P62826	RAN	GTP-binding nuclear protein Ran	1.1	0.000	PRDX3
Q8WUH6	TMEM263	Transmembrane protein 263	1.1	0.001	PRDX3
P18077	RPL35A	60S ribosomal protein L35a	1.1	0.004	PRDX3
P78346	RPP30	Ribonuclease P protein subunit p30	1.1	0.004	PRDX3
P62753	RPS6	40S ribosomal protein S6	1.1	0.014	PRDX3
P05388	RPLP0	60S acidic ribosomal protein P0	1.1	0.015	PRDX3
A0A0A0MRN5	OGFR	Opioid growth factor receptor	1.1	0.022	PRDX3
B1AMS2	SEPTIN6	Septin 6, isoform CRA_b	1.1	0.032	PRDX3
Q9NS69	TOMM22	Mitochondrial import receptor subunit TOM22 homolog	1.1	0.039	PRDX3
P23381	WARS1	Tryptophan--tRNA ligase, cytoplasmic	1.0	0.002	PRDX3
P62888	RPL30	60S ribosomal protein L30	1.0	0.005	PRDX3
Q9H936	SLC25A22	Mitochondrial glutamate carrier 1	1.0	0.019	PRDX3
Q8NCW5	NAXE	NAD(P)H-hydrate epimerase	1.0	0.020	PRDX3
P42771	CDKN2A	Cyclin-dependent kinase inhibitor 2A	1.0	0.030	PRDX3
Q9UJZ1	STOML2	Stomatin-like protein 2, mitochondrial	1.0	0.038	PRDX3
X1WI28	RPL10	60S ribosomal protein L10	1.0	0.040	PRDX3
Q9Y224	RTRAF	RNA transcription, translation and transport factor protein	5.1	0.000	PRDX4
P32119	PRDX2	Peroxiredoxin-2	5.1	0.000	PRDX4
P00167	CYB5A	Cytochrome b5	4.2	0.000	PRDX4
H0YNW5	DUT	Deoxyuridine 5'-triphosphate nucleotidohydrolase	4.0	0.000	PRDX4
Q9NPH2	ISYNA1	Inositol-3-phosphate synthase 1	3.6	0.000	PRDX4
P10301	RRAS	Ras-related protein R-Ras	3.5	0.000	PRDX4
Q96CT7	CCDC124	Coiled-coil domain-containing protein 124	3.4	0.000	PRDX4
O96007	MOCS2	Molybdopterin synthase catalytic subunit	3.4	0.000	PRDX4
Q9P2B4	CTTNBP2NL	CTTNBP2 N-terminal-like protein	3.4	0.000	PRDX4
Q8NFC6	BOD1L1	Biorientation of chromosomes in cell division protein 1-like 1	3.3	0.000	PRDX4
P41250	GARS1	Glycine--tRNA ligase	3.3	0.000	PRDX4
O60664	PLIN3	Perilipin-3	3.2	0.000	PRDX4
P11802	CDK4	Cyclin-dependent kinase 4	3.2	0.000	PRDX4
Q9Y6A5	TACC3	Transforming acidic coiled-coil-containing protein 3	3.2	0.001	PRDX4
Q9Y3D6	FIS1	Mitochondrial fission 1 protein	3.0	0.000	PRDX4
Q9ULW0	TPX2	Targeting protein for Xklp2	3.0	0.000	PRDX4
Q6UW78	UQCC3	Ubiquinol-cytochrome-c reductase complex assembly factor 3	3.0	0.000	PRDX4
P61758	VBP1	Prefoldin subunit 3	2.8	0.000	PRDX4
P42574	CASP3	Caspase-3	2.8	0.000	PRDX4
Q9BPW8	NIPSNAP1	Protein NipSnap homolog 1	2.8	0.000	PRDX4
P26599	PTBP1	Polypyrimidine tract-binding protein 1	2.8	0.000	PRDX4
Q9NS69	TOMM22	Mitochondrial import receptor subunit TOM22 homolog	2.8	0.000	PRDX4
Q9NX55	HYPK	Huntingtin-interacting protein K	2.8	0.003	PRDX4
H3BRL3	UBFD1	Ubiquitin domain-containing protein UBFD1	2.7	0.000	PRDX4
B7Z7F3	RANBP3	Ran-binding protein 3	2.6	0.000	PRDX4
Q9H840	GEMIN7	Gem-associated protein 7	2.6	0.000	PRDX4
Q6WKZ4	RAB11FIP1	Rab11 family-interacting protein 1	2.6	0.000	PRDX4
Q8N9Q2	SREK1IP1	Protein SREK1IP1	2.6	0.000	PRDX4
P30740	SERPINB1	Leukocyte elastase inhibitor	2.6	0.000	PRDX4
A0A087WTM1	ROBO1	Roundabout homolog 1	2.6	0.000	PRDX4
G5EA06	MRPS27	28S ribosomal protein S27, mitochondrial	2.6	0.001	PRDX4
P53602	MVD	Diphosphomevalonate decarboxylase	2.6	0.001	PRDX4
Q96EA4	SPDL1	Protein Spindly	2.6	0.002	PRDX4
Q6PID6	TTC33	Tetratricopeptide repeat protein 33	2.6	0.026	PRDX4
Q96A10	ERVK3-1	Endogenous retrovirus group K3 member 1	2.6	0.028	PRDX4
P22234	PAICS	Multifunctional protein ADE2 [Includes: Phosphoribosylaminoimidazole-succinocarboxamide synthase	2.5	0.000	PRDX4
Q6IQ49	SDE2	Replication stress response regulator SDE2	2.5	0.000	PRDX4
P34897	SHMT2	Serine hydroxymethyltransferase, mitochondrial	2.5	0.000	PRDX4
E9PKG1	PRMT1	Protein arginine N-methyltransferase 1	2.5	0.000	PRDX4
Q13542	EIF4EBP2	Eukaryotic translation initiation factor 4E-binding protein 2	2.5	0.002	PRDX4
Q96KB5	PBK	Lymphokine-activated killer T-cell-originated protein kinase	2.5	0.002	PRDX4
P12955	PEPD	Xaa-Pro dipeptidase	2.5	0.003	PRDX4
H7BXI1	ESYT2	Extended synaptotagmin-2	2.4	0.000	PRDX4
O75223	GGCT	Gamma-glutamylcyclotransferase	2.4	0.000	PRDX4
Q9NP97	DYNLRB1	Dynein light chain roadblock-type 1	2.4	0.000	PRDX4
Q9H0U4	RAB1B	Ras-related protein Rab-1B	2.4	0.000	PRDX4
P42771	CDKN2A	Cyclin-dependent kinase inhibitor 2A	2.4	0.000	PRDX4
P12236	SLC25A6	ADP/ATP translocase 3	2.4	0.000	PRDX4
P56385	ATP5ME	ATP synthase subunit e, mitochondrial	2.4	0.001	PRDX4
P06132	UROD	Uroporphyrinogen decarboxylase	2.4	0.003	PRDX4
Q8WVC2	RPS21	40S ribosomal protein S21	2.3	0.001	PRDX4
Q9H2P9	DPH5	Diphthine methyl ester synthase	2.3	0.002	PRDX4
P49903	SEPHS1	Selenide, water dikinase 1	2.3	0.002	PRDX4
A6NGP5	JPT2	Jupiter microtubule-associated homolog 2	2.3	0.002	PRDX4
Q13126	MTAP	S-methyl-5'-thioadenosine phosphorylase	2.3	0.003	PRDX4
O14965	AURKA	Aurora kinase A	2.2	0.000	PRDX4
P48047	ATP5PO	ATP synthase subunit O, mitochondrial	2.2	0.001	PRDX4
Q9Y266	NUDC	Nuclear migration protein nudC	2.2	0.006	PRDX4
Q7Z4V5	HDGFL2	Hepatoma-derived growth factor-related protein 2	2.2	0.008	PRDX4
E7EV99	ADD1	Alpha-adducin	2.1	0.000	PRDX4
Q8N3X1	FNBP4	Formin-binding protein 4	2.1	0.000	PRDX4
O95831	AIFM1	Apoptosis-inducing factor 1, mitochondrial	2.1	0.000	PRDX4
P36542	ATP5F1C	ATP synthase subunit gamma, mitochondrial	2.1	0.000	PRDX4
Q9BTE1	DCTN5	Dynactin subunit 5	2.1	0.001	PRDX4
P47985	UQCRFS1	Cytochrome b-c1 complex subunit Rieske, mitochondrial	2.1	0.001	PRDX4
P00491	PNP	Purine nucleoside phosphorylase	2.1	0.002	PRDX4
P85037	FOXK1	Forkhead box protein K1	2.1	0.003	PRDX4
A0AVT1	UBA6	Ubiquitin-like modifier-activating enzyme 6	2.1	0.006	PRDX4
P07355	ANXA2	Annexin A2	2.1	0.028	PRDX4
P63000	RAC1	Ras-related C3 botulinum toxin substrate 1	2.0	0.000	PRDX4
P63167	DYNLL1	Dynein light chain 1, cytoplasmic	2.0	0.001	PRDX4
Q9NUP9	LIN7C	Protein lin-7 homolog C	2.0	0.003	PRDX4
P84085	ARF5	ADP-ribosylation factor 5	2.0	0.004	PRDX4
P35244	RPA3	Replication protein A 14 kDa subunit	2.0	0.004	PRDX4
O60547	GMDS	GDP-mannose 4,6 dehydratase	2.0	0.009	PRDX4
Q9HA64	FN3KRP	Ketosamine-3-kinase	2.0	0.009	PRDX4
P10599	TXN	Thioredoxin	2.0	0.028	PRDX4
Q9C0C2	TNKS1BP1	182 kDa tankyrase-1-binding protein	2.0	0.034	PRDX4
P62273	RPS29	40S ribosomal protein S29	2.0	0.038	PRDX4
P49642	PRIM1	DNA primase small subunit	1.9	0.000	PRDX4
Q9NUQ3	TXLNG	Gamma-taxilin	1.9	0.001	PRDX4
A0A087X2D5	MRPL45	39S ribosomal protein L45, mitochondrial	1.9	0.002	PRDX4
A4D1S0	KLRG2	Killer cell lectin-like receptor subfamily G member 2	1.9	0.002	PRDX4
P62304	SNRPE	Small nuclear ribonucleoprotein E	1.9	0.005	PRDX4
O94992	HEXIM1	Protein HEXIM1	1.9	0.005	PRDX4
Q5JRI1	SRSF10	Serine/arginine-rich-splicing factor 10	1.9	0.006	PRDX4
E9PGT1	TSN	Component 3 of promoter of RISC	1.9	0.007	PRDX4
Q9BSJ8	ESYT1	Extended synaptotagmin-1	1.9	0.008	PRDX4
P25705	ATP5F1A	ATP synthase subunit alpha, mitochondrial	1.8	0.000	PRDX4
Q12948	FOXC1	Forkhead box protein C1	1.8	0.001	PRDX4
Q9P258	RCC2	Protein RCC2	1.8	0.005	PRDX4
U3KQC1	WDR18	WD repeat-containing protein 18	1.8	0.005	PRDX4
P52888	THOP1	Thimet oligopeptidase	1.8	0.006	PRDX4
Q9Y4P1	ATG4B	Cysteine protease ATG4B	1.8	0.007	PRDX4
Q96DH6	MSI2	RNA-binding protein Musashi homolog 2	1.8	0.007	PRDX4
P00390	GSR	Glutathione reductase, mitochondrial	1.8	0.009	PRDX4
P54646	PRKAA2	5'-AMP-activated protein kinase catalytic subunit alpha-2	1.8	0.009	PRDX4
P61106	RAB14	Ras-related protein Rab-14	1.8	0.009	PRDX4
Q9NVJ2	ARL8B	ADP-ribosylation factor-like protein 8B	1.8	0.017	PRDX4
E9PDD6	BCL2L13	Bcl-2-like protein 13	1.8	0.023	PRDX4
P05091	ALDH2	Aldehyde dehydrogenase, mitochondrial	1.7	0.000	PRDX4
P31939	ATIC	Bifunctional purine biosynthesis protein ATIC	1.7	0.000	PRDX4
O00483	NDUFA4	Cytochrome c oxidase subunit NDUFA4	1.7	0.001	PRDX4
Q6PKG0	LARP1	La-related protein 1	1.7	0.001	PRDX4
P48147	PREP	Prolyl endopeptidase	1.7	0.003	PRDX4
Q9Y5K6	CD2AP	CD2-associated protein	1.7	0.006	PRDX4
Q9BZE1	MRPL37	39S ribosomal protein L37, mitochondrial	1.7	0.006	PRDX4
Q71UI9	H2AZ2	Histone H2A.V	1.7	0.008	PRDX4
Q16204	CCDC6	Coiled-coil domain-containing protein 6	1.7	0.011	PRDX4
Q9Y570	PPME1	Protein phosphatase methylesterase 1	1.7	0.011	PRDX4
P07384	CAPN1	Calpain-1 catalytic subunit	1.7	0.015	PRDX4
P49458	SRP9	Signal recognition particle 9 kDa protein	1.7	0.015	PRDX4
Q13541	EIF4EBP1	Eukaryotic translation initiation factor 4E-binding protein 1	1.7	0.018	PRDX4
J3KS31	ZNF207	BUB3-interacting and GLEBS motif-containing protein ZNF207	1.7	0.039	PRDX4
Q7Z434	MAVS	Mitochondrial antiviral-signaling protein	1.7	0.045	PRDX4
P35237	SERPINB6	Serpin B6	1.6	0.001	PRDX4
Q8TEX9	IPO4	Importin-4	1.6	0.001	PRDX4
Q9UJS0	SLC25A13	Calcium-binding mitochondrial carrier protein Aralar2	1.6	0.001	PRDX4
J3KN29	PSMD9	26S proteasome non-ATPase regulatory subunit 9	1.6	0.003	PRDX4
B4DDF4	CNN2	Calponin	1.6	0.003	PRDX4
Q8IYS1	PM20D2	Peptidase M20 domain-containing protein 2	1.6	0.006	PRDX4
Q32MZ4	LRRFIP1	Leucine-rich repeat flightless-interacting protein 1	1.6	0.013	PRDX4
G3V1Q4	SEPTIN7	Septin-7	1.6	0.017	PRDX4
O14949	UQCRQ	Cytochrome b-c1 complex subunit 8	1.6	0.021	PRDX4
B1AMS2	SEPTIN6	Septin 6, isoform CRA_b	1.6	0.021	PRDX4
J3KN87	J3KN87	>tr|J3KN87|J3KN87_HUMAN Serine/threonine-protein kinase Chk1 OS=Homo sapiens OX=9606 GN=CHEK1 PE=1 SV=1;>sp|O14757|CHK1_HUMAN Serine/threonine-protein kinase Chk1 OS=Homo sapiens OX=9606 GN=CHEK1 PE=1 SV=2;>tr|E7EPP6|E7EPP6_HUMAN Serine/threonine-protein k	1.6	0.022	PRDX4
P30041	PRDX6	Peroxiredoxin-6	1.6	0.028	PRDX4
A0A2R8YFH5	SEC23B	Protein transport protein SEC23	1.6	0.039	PRDX4
P51452	DUSP3	Dual specificity protein phosphatase 3	1.5	0.000	PRDX4
P61586	RHOA	Transforming protein RhoA	1.5	0.000	PRDX4
P00338	LDHA	L-lactate dehydrogenase A chain	1.5	0.000	PRDX4
P51149	RAB7A	Ras-related protein Rab-7a	1.5	0.000	PRDX4
P05141	SLC25A5	ADP/ATP translocase 2	1.5	0.000	PRDX4
P49593	PPM1F	Protein phosphatase 1F	1.5	0.001	PRDX4
E9PN17	ATP5MG	ATP synthase subunit g, mitochondrial	1.5	0.001	PRDX4
Q5QPM7	PSMF1	Proteasome inhibitor PI31 subunit	1.5	0.002	PRDX4
Q9GZS3	WDR61	WD repeat-containing protein 61	1.5	0.003	PRDX4
P62244	RPS15A	40S ribosomal protein S15a	1.5	0.003	PRDX4
Q9NYK5	MRPL39	39S ribosomal protein L39, mitochondrial	1.5	0.008	PRDX4
Q9H974	QTRT2	Queuine tRNA-ribosyltransferase accessory subunit 2	1.5	0.018	PRDX4
O75306	NDUFS2	NADH dehydrogenase [ubiquinone] iron-sulfur protein 2, mitochondrial	1.5	0.020	PRDX4
Q16186	ADRM1	Proteasomal ubiquitin receptor ADRM1	1.5	0.028	PRDX4
Q13190	STX5	Syntaxin-5	1.5	0.029	PRDX4
A0A1B0GUC3	FTO	Alpha-ketoglutarate-dependent dioxygenase FTO	1.5	0.030	PRDX4
P19022	CDH2	Cadherin-2	1.5	0.038	PRDX4
Q9UHV9	PFDN2	Prefoldin subunit 2	1.5	0.038	PRDX4
P61927	RPL37	60S ribosomal protein L37	1.5	0.040	PRDX4
O00487	PSMD14	26S proteasome non-ATPase regulatory subunit 14	1.4	0.000	PRDX4
P68104	EEF1A1	Elongation factor 1-alpha 1	1.4	0.000	PRDX4
O43242	PSMD3	26S proteasome non-ATPase regulatory subunit 3	1.4	0.001	PRDX4
Q9BV44	THUMPD3	THUMP domain-containing protein 3	1.4	0.001	PRDX4
P05388	RPLP0	60S acidic ribosomal protein P0	1.4	0.002	PRDX4
P68371	TUBB4B	Tubulin beta-4B chain	1.4	0.003	PRDX4
Q53EL6	PDCD4	Programmed cell death protein 4	1.4	0.004	PRDX4
Q9UKK9	NUDT5	ADP-sugar pyrophosphatase	1.4	0.004	PRDX4
J3QQZ9	PNPO	Pyridoxal 5'-phosphate synthase	1.4	0.020	PRDX4
Q96I15	SCLY	Selenocysteine lyase	1.4	0.024	PRDX4
Q99598	TSNAX	Translin-associated protein X	1.4	0.026	PRDX4
Q9Y277	VDAC3	Voltage-dependent anion-selective channel protein 3	1.4	0.027	PRDX4
Q96GK7	FAHD2A	Fumarylacetoacetate hydrolase domain-containing protein 2A	1.4	0.044	PRDX4
Q14694	USP10	Ubiquitin carboxyl-terminal hydrolase 10	1.3	0.001	PRDX4
Q92688	ANP32B	Acidic leucine-rich nuclear phosphoprotein 32 family member B	1.3	0.002	PRDX4
D6R938	CAMK2D	Calcium/calmodulin-dependent protein kinase	1.3	0.002	PRDX4
Q9BRP1	PDCD2L	Programmed cell death protein 2-like	1.3	0.003	PRDX4
Q9UHD1	CHORDC1	Cysteine and histidine-rich domain-containing protein 1	1.3	0.005	PRDX4
Q9BT25	HAUS8	HAUS augmin-like complex subunit 8	1.3	0.007	PRDX4
P63244	RACK1	Receptor of activated protein C kinase 1	1.3	0.008	PRDX4
P12235	SLC25A4	ADP/ATP translocase 1	1.3	0.020	PRDX4
Q8IVM0	CCDC50	Coiled-coil domain-containing protein 50	1.3	0.020	PRDX4
O14929	HAT1	Histone acetyltransferase type B catalytic subunit	1.3	0.024	PRDX4
Q8N6M0	OTUD6B	Deubiquitinase OTUD6B	1.3	0.035	PRDX4
P00492	HPRT1	Hypoxanthine-guanine phosphoribosyltransferase	1.3	0.042	PRDX4
Q96HS1	PGAM5	Serine/threonine-protein phosphatase PGAM5, mitochondrial	1.3	0.043	PRDX4
H0YLH3	RABGGTA	Geranylgeranyl transferase type-2 subunit alpha	1.3	0.044	PRDX4
Q92576	PHF3	PHD finger protein 3	1.3	0.047	PRDX4
Q86W42	THOC6	THO complex subunit 6 homolog	1.3	0.048	PRDX4
O95394	PGM3	Phosphoacetylglucosamine mutase	1.2	0.000	PRDX4
P17858	PFKL	ATP-dependent 6-phosphofructokinase, liver type	1.2	0.000	PRDX4
Q92769	HDAC2	Histone deacetylase 2	1.2	0.001	PRDX4
P22102	GART	Trifunctional purine biosynthetic protein adenosine-3 [Includes: Phosphoribosylamine--glycine ligase	1.2	0.001	PRDX4
P25398	RPS12	40S ribosomal protein S12	1.2	0.001	PRDX4
Q9UNM6	PSMD13	26S proteasome non-ATPase regulatory subunit 13	1.2	0.002	PRDX4
P31040	SDHA	Succinate dehydrogenase [ubiquinone] flavoprotein subunit, mitochondrial	1.2	0.002	PRDX4
P61313	RPL15	60S ribosomal protein L15	1.2	0.003	PRDX4
P22695	UQCRC2	Cytochrome b-c1 complex subunit 2, mitochondrial	1.2	0.004	PRDX4
O75400	PRPF40A	Pre-mRNA-processing factor 40 homolog A	1.2	0.004	PRDX4
O94903	PLPBP	Pyridoxal phosphate homeostasis protein	1.2	0.004	PRDX4
P49736	MCM2	DNA replication licensing factor MCM2	1.2	0.004	PRDX4
Q15738	NSDHL	Sterol-4-alpha-carboxylate 3-dehydrogenase, decarboxylating	1.2	0.006	PRDX4
O15144	ARPC2	Actin-related protein 2/3 complex subunit 2	1.2	0.007	PRDX4
P31930	UQCRC1	Cytochrome b-c1 complex subunit 1, mitochondrial	1.2	0.008	PRDX4
P21964	COMT	Catechol O-methyltransferase	1.2	0.008	PRDX4
O95373	IPO7	Importin-7	1.2	0.010	PRDX4
Q14232	EIF2B1	Translation initiation factor eIF-2B subunit alpha	1.2	0.011	PRDX4
Q9UJZ1	STOML2	Stomatin-like protein 2, mitochondrial	1.2	0.015	PRDX4
P47755	CAPZA2	F-actin-capping protein subunit alpha-2	1.2	0.017	PRDX4
P04181	OAT	Ornithine aminotransferase, mitochondrial	1.2	0.019	PRDX4
K7EJQ8	HDHD2	Haloacid dehalogenase-like hydrolase domain-containing protein 2	1.2	0.025	PRDX4
Q7L2J0	MEPCE	7SK snRNA methylphosphate capping enzyme	1.2	0.030	PRDX4
Q13177	PAK2	Serine/threonine-protein kinase PAK 2	1.2	0.032	PRDX4
O00231	PSMD11	26S proteasome non-ATPase regulatory subunit 11	1.2	0.034	PRDX4
P40222	TXLNA	Alpha-taxilin	1.2	0.035	PRDX4
Q9Y262	EIF3L	Eukaryotic translation initiation factor 3 subunit L	1.2	0.044	PRDX4
B4DLN1	B4DLN1	cDNA FLJ60124, highly similar to Mitochondrial dicarboxylate carrier	1.1	0.001	PRDX4
Q06203	PPAT	Amidophosphoribosyltransferase	1.1	0.002	PRDX4
A0A087WUT6	EIF5B	Eukaryotic translation initiation factor 5B	1.1	0.002	PRDX4
Q96BW9	TAMM41	Phosphatidate cytidylyltransferase, mitochondrial	1.1	0.003	PRDX4
P15121	AKR1B1	Aldo-keto reductase family 1 member B1	1.1	0.003	PRDX4
Q2M1P5	KIF7	Kinesin-like protein KIF7	1.1	0.004	PRDX4
P46778	RPL21	60S ribosomal protein L21	1.1	0.004	PRDX4
Q9Y5Y2	NUBP2	Cytosolic Fe-S cluster assembly factor NUBP2	1.1	0.004	PRDX4
P50914	RPL14	60S ribosomal protein L14	1.1	0.004	PRDX4
P35232	PHB	Prohibitin	1.1	0.004	PRDX4
P08574	CYC1	Cytochrome c1, heme protein, mitochondrial	1.1	0.010	PRDX4
P31153	MAT2A	S-adenosylmethionine synthase isoform type-2	1.1	0.012	PRDX4
P15880	RPS2	40S ribosomal protein S2	1.1	0.017	PRDX4
Q13618	CUL3	Cullin-3	1.1	0.017	PRDX4
P62851	RPS25	40S ribosomal protein S25	1.1	0.021	PRDX4
H0YDU8	PPP5C	Serine/threonine-protein phosphatase	1.1	0.022	PRDX4
Q5T760	SRSF11	Serine/arginine-rich-splicing factor 11	1.1	0.023	PRDX4
Q92922	SMARCC1	SWI/SNF complex subunit SMARCC1	1.1	0.027	PRDX4
Q14C86	GAPVD1	GTPase-activating protein and VPS9 domain-containing protein 1	1.1	0.034	PRDX4
A0A1B0GTG2	ALDH7A1	Alpha-aminoadipic semialdehyde dehydrogenase	1.1	0.037	PRDX4
O15143	ARPC1B	Actin-related protein 2/3 complex subunit 1B	1.1	0.038	PRDX4
P40926	MDH2	Malate dehydrogenase, mitochondrial	1.0	0.000	PRDX4
Q06210	GFPT1	Glutamine--fructose-6-phosphate aminotransferase [isomerizing] 1	1.0	0.000	PRDX4
P13639	EEF2	Elongation factor 2	1.0	0.000	PRDX4
O00303	EIF3F	Eukaryotic translation initiation factor 3 subunit F	1.0	0.002	PRDX4
P62888	RPL30	60S ribosomal protein L30	1.0	0.004	PRDX4
P62495	ETF1	Eukaryotic peptide chain release factor subunit 1	1.0	0.005	PRDX4
P49411	TUFM	Elongation factor Tu, mitochondrial	1.0	0.027	PRDX4
Q53H12	AGK	Acylglycerol kinase, mitochondrial	1.0	0.040	PRDX4
Q9Y224	RTRAF	RNA transcription, translation and transport factor protein	6.9	0.000	PRDX5
P32119	PRDX2	Peroxiredoxin-2	5.2	0.000	PRDX5
P00167	CYB5A	Cytochrome b5	4.1	0.000	PRDX5
Q96CT7	CCDC124	Coiled-coil domain-containing protein 124	3.3	0.000	PRDX5
O96007	MOCS2	Molybdopterin synthase catalytic subunit	3.3	0.000	PRDX5
P10301	RRAS	Ras-related protein R-Ras	3.3	0.000	PRDX5
H0YNW5	DUT	Deoxyuridine 5'-triphosphate nucleotidohydrolase	3.2	0.000	PRDX5
Q9P2B4	CTTNBP2NL	CTTNBP2 N-terminal-like protein	3.1	0.000	PRDX5
Q9Y3D6	FIS1	Mitochondrial fission 1 protein	2.9	0.000	PRDX5
Q6UW78	UQCC3	Ubiquinol-cytochrome-c reductase complex assembly factor 3	2.8	0.000	PRDX5
Q9ULW0	TPX2	Targeting protein for Xklp2	2.8	0.000	PRDX5
P63167	DYNLL1	Dynein light chain 1, cytoplasmic	2.7	0.000	PRDX5
Q9NX55	HYPK	Huntingtin-interacting protein K	2.7	0.005	PRDX5
Q9BPW8	NIPSNAP1	Protein NipSnap homolog 1	2.6	0.000	PRDX5
Q8N9Q2	SREK1IP1	Protein SREK1IP1	2.5	0.001	PRDX5
Q96AC1	FERMT2	Fermitin family homolog 2	2.5	0.002	PRDX5
O60664	PLIN3	Perilipin-3	2.5	0.003	PRDX5
P53602	MVD	Diphosphomevalonate decarboxylase	2.4	0.001	PRDX5
Q9H840	GEMIN7	Gem-associated protein 7	2.4	0.001	PRDX5
P55084	HADHB	Trifunctional enzyme subunit beta, mitochondrial	2.4	0.004	PRDX5
Q9NP97	DYNLRB1	Dynein light chain roadblock-type 1	2.3	0.001	PRDX5
Q6IQ49	SDE2	Replication stress response regulator SDE2	2.2	0.000	PRDX5
P62244	RPS15A	40S ribosomal protein S15a	2.2	0.000	PRDX5
Q9BZE1	MRPL37	39S ribosomal protein L37, mitochondrial	2.2	0.001	PRDX5
Q8WVC2	RPS21	40S ribosomal protein S21	2.2	0.003	PRDX5
P19105	MYL12A	Myosin regulatory light chain 12A	2.2	0.003	PRDX5
A0A0B4J1Z1	SRSF7	Serine/arginine-rich-splicing factor 7	2.2	0.004	PRDX5
Q13126	MTAP	S-methyl-5'-thioadenosine phosphorylase	2.2	0.006	PRDX5
P06132	UROD	Uroporphyrinogen decarboxylase	2.2	0.006	PRDX5
P30048	PRDX3	Thioredoxin-dependent peroxide reductase, mitochondrial	2.2	0.009	PRDX5
B4DDF4	CNN2	Calponin	2.1	0.000	PRDX5
Q9NS69	TOMM22	Mitochondrial import receptor subunit TOM22 homolog	2.1	0.000	PRDX5
P19338	NCL	Nucleolin	2.1	0.000	PRDX5
Q9GZS3	WDR61	WD repeat-containing protein 61	2.1	0.000	PRDX5
P48047	ATP5PO	ATP synthase subunit O, mitochondrial	2.1	0.002	PRDX5
Q9BYN0	SRXN1	Sulfiredoxin-1	2.1	0.018	PRDX5
O14965	AURKA	Aurora kinase A	2.0	0.000	PRDX5
O95373	IPO7	Importin-7	2.0	0.000	PRDX5
H3BRL3	UBFD1	Ubiquitin domain-containing protein UBFD1	2.0	0.004	PRDX5
Q9BTE1	DCTN5	Dynactin subunit 5	2.0	0.004	PRDX5
P00491	PNP	Purine nucleoside phosphorylase	2.0	0.004	PRDX5
E9PHS0	LANCL1	Glutathione S-transferase LANCL1	2.0	0.018	PRDX5
E9PKG1	PRMT1	Protein arginine N-methyltransferase 1	1.9	0.000	PRDX5
Q12948	FOXC1	Forkhead box protein C1	1.9	0.001	PRDX5
P53007	SLC25A1	Tricarboxylate transport protein, mitochondrial	1.9	0.004	PRDX5
P26599	PTBP1	Polypyrimidine tract-binding protein 1	1.9	0.004	PRDX5
P84085	ARF5	ADP-ribosylation factor 5	1.9	0.010	PRDX5
P35244	RPA3	Replication protein A 14 kDa subunit	1.9	0.010	PRDX5
P68133	ACTA1	Actin, alpha skeletal muscle	1.9	0.030	PRDX5
P15880	RPS2	40S ribosomal protein S2	1.8	0.000	PRDX5
P46778	RPL21	60S ribosomal protein L21	1.8	0.000	PRDX5
P40429	RPL13A	60S ribosomal protein L13a	1.8	0.000	PRDX5
A0A087WTM1	ROBO1	Roundabout homolog 1	1.8	0.000	PRDX5
P62266	RPS23	40S ribosomal protein S23	1.8	0.001	PRDX5
F2Z388	RPL35	60S ribosomal protein L35	1.8	0.001	PRDX5
Q96DH6	MSI2	RNA-binding protein Musashi homolog 2	1.8	0.006	PRDX5
G5EA06	MRPS27	28S ribosomal protein S27, mitochondrial	1.8	0.010	PRDX5
P62304	SNRPE	Small nuclear ribonucleoprotein E	1.8	0.013	PRDX5
Q86WA6	BPHL	Valacyclovir hydrolase	1.8	0.015	PRDX5
Q5JRI1	SRSF10	Serine/arginine-rich-splicing factor 10	1.8	0.016	PRDX5
P53597	SUCLG1	Succinate--CoA ligase [ADP/GDP-forming] subunit alpha, mitochondrial	1.7	0.001	PRDX5
P04181	OAT	Ornithine aminotransferase, mitochondrial	1.7	0.001	PRDX5
P63244	RACK1	Receptor of activated protein C kinase 1	1.7	0.001	PRDX5
Q9Y5K6	CD2AP	CD2-associated protein	1.7	0.002	PRDX5
O15143	ARPC1B	Actin-related protein 2/3 complex subunit 1B	1.7	0.018	PRDX5
P00390	GSR	Glutathione reductase, mitochondrial	1.7	0.022	PRDX5
Q15477	SKIV2L	Helicase SKI2W	1.7	0.023	PRDX5
Q13542	EIF4EBP2	Eukaryotic translation initiation factor 4E-binding protein 2	1.7	0.031	PRDX5
O95168	NDUFB4	NADH dehydrogenase [ubiquinone] 1 beta subcomplex subunit 4	1.6	0.000	PRDX5
Q02878	RPL6	60S ribosomal protein L6	1.6	0.001	PRDX5
A0A0U1RRM6	ENAH	Protein enabled homolog	1.6	0.001	PRDX5
O95861	BPNT1	3'(2'),5'-bisphosphate nucleotidase 1	1.6	0.003	PRDX5
P82930	MRPS34	28S ribosomal protein S34, mitochondrial	1.6	0.004	PRDX5
Q96I15	SCLY	Selenocysteine lyase	1.6	0.006	PRDX5
A0A087X2D5	MRPL45	39S ribosomal protein L45, mitochondrial	1.6	0.010	PRDX5
Q4VCS5	AMOT	Angiomotin	1.6	0.013	PRDX5
Q9Y4P1	ATG4B	Cysteine protease ATG4B	1.6	0.019	PRDX5
P16403	H1-2	Histone H1.2	1.6	0.031	PRDX5
P07384	CAPN1	Calpain-1 catalytic subunit	1.6	0.034	PRDX5
Q9NXH9	TRMT1	tRNA	1.6	0.039	PRDX5
P14174	MIF	Macrophage migration inhibitory factor	1.6	0.045	PRDX5
E9PDD6	BCL2L13	Bcl-2-like protein 13	1.6	0.045	PRDX5
Q7Z4V5	HDGFL2	Hepatoma-derived growth factor-related protein 2	1.6	0.048	PRDX5
P61313	RPL15	60S ribosomal protein L15	1.5	0.000	PRDX5
P36542	ATP5F1C	ATP synthase subunit gamma, mitochondrial	1.5	0.000	PRDX5
E9PEJ4	DLAT	Acetyltransferase component of pyruvate dehydrogenase complex	1.5	0.001	PRDX5
P35237	SERPINB6	Serpin B6	1.5	0.001	PRDX5
C9J9K3	RPSA	40S ribosomal protein SA	1.5	0.001	PRDX5
G5E9W7	MRPS22	28S ribosomal protein S22, mitochondrial	1.5	0.002	PRDX5
P63000	RAC1	Ras-related C3 botulinum toxin substrate 1	1.5	0.002	PRDX5
Q8TEX9	IPO4	Importin-4	1.5	0.002	PRDX5
P61353	RPL27	60S ribosomal protein L27	1.5	0.004	PRDX5
Q7L2J0	MEPCE	7SK snRNA methylphosphate capping enzyme	1.5	0.011	PRDX5
Q71UI9	H2AZ2	Histone H2A.V	1.5	0.022	PRDX5
Q9Y2R5	MRPS17	28S ribosomal protein S17, mitochondrial	1.5	0.028	PRDX5
Q9H7N4	SCAF1	Splicing factor, arginine/serine-rich 19	1.5	0.032	PRDX5
P42345	MTOR	Serine/threonine-protein kinase mTOR	1.5	0.035	PRDX5
G3V1Q4	SEPTIN7	Septin-7	1.5	0.038	PRDX5
Q16762	TST	Thiosulfate sulfurtransferase	1.5	0.039	PRDX5
Q6P1L8	MRPL14	39S ribosomal protein L14, mitochondrial	1.5	0.048	PRDX5
P23921	RRM1	Ribonucleoside-diphosphate reductase large subunit	1.4	0.000	PRDX5
P12236	SLC25A6	ADP/ATP translocase 3	1.4	0.000	PRDX5
P62081	RPS7	40S ribosomal protein S7	1.4	0.000	PRDX5
P34897	SHMT2	Serine hydroxymethyltransferase, mitochondrial	1.4	0.001	PRDX5
P50914	RPL14	60S ribosomal protein L14	1.4	0.001	PRDX5
P62750	RPL23A	60S ribosomal protein L23a	1.4	0.001	PRDX5
P51452	DUSP3	Dual specificity protein phosphatase 3	1.4	0.001	PRDX5
P62269	RPS18	40S ribosomal protein S18	1.4	0.001	PRDX5
P43897	TSFM	Elongation factor Ts, mitochondrial	1.4	0.001	PRDX5
Q9NQT4	EXOSC5	Exosome complex component RRP46	1.4	0.001	PRDX5
P22570	FDXR	NADPH:adrenodoxin oxidoreductase, mitochondrial	1.4	0.001	PRDX5
P62495	ETF1	Eukaryotic peptide chain release factor subunit 1	1.4	0.002	PRDX5
P68371	TUBB4B	Tubulin beta-4B chain	1.4	0.004	PRDX5
Q7L1Q6	BZW1	Basic leucine zipper and W2 domain-containing protein 1	1.4	0.005	PRDX5
Q96IU4	ABHD14B	Protein ABHD14B	1.4	0.006	PRDX5
O94973	AP2A2	AP-2 complex subunit alpha-2	1.4	0.007	PRDX5
P12277	CKB	Creatine kinase B-type	1.4	0.013	PRDX5
Q86XI2	NCAPG2	Condensin-2 complex subunit G2	1.4	0.024	PRDX5
P62829	RPL23	60S ribosomal protein L23	1.4	0.025	PRDX5
Q13613	MTMR1	Myotubularin-related protein 1	1.4	0.030	PRDX5
O00629	KPNA4	Importin subunit alpha-3	1.4	0.033	PRDX5
Q9UHQ9	CYB5R1	NADH-cytochrome b5 reductase 1	1.4	0.034	PRDX5
Q5VW32	BROX	BRO1 domain-containing protein BROX	1.4	0.041	PRDX5
O75306	NDUFS2	NADH dehydrogenase [ubiquinone] iron-sulfur protein 2, mitochondrial	1.4	0.046	PRDX5
P68104	EEF1A1	Elongation factor 1-alpha 1	1.3	0.000	PRDX5
B4DLN1	B4DLN1	cDNA FLJ60124, highly similar to Mitochondrial dicarboxylate carrier	1.3	0.000	PRDX5
Q8WUH6	TMEM263	Transmembrane protein 263	1.3	0.000	PRDX5
P05091	ALDH2	Aldehyde dehydrogenase, mitochondrial	1.3	0.001	PRDX5
Q92688	ANP32B	Acidic leucine-rich nuclear phosphoprotein 32 family member B	1.3	0.002	PRDX5
O00442	RTCA	RNA 3'-terminal phosphate cyclase	1.3	0.003	PRDX5
P11172	UMPS	Uridine 5'-monophosphate synthase	1.3	0.005	PRDX5
Q86U42	PABPN1	Polyadenylate-binding protein 2	1.3	0.005	PRDX5
A4D1S0	KLRG2	Killer cell lectin-like receptor subfamily G member 2	1.3	0.006	PRDX5
Q9NPH2	ISYNA1	Inositol-3-phosphate synthase 1	1.3	0.008	PRDX5
D6R938	CAMK2D	Calcium/calmodulin-dependent protein kinase	1.3	0.010	PRDX5
P84103	SRSF3	Serine/arginine-rich splicing factor 3	1.3	0.010	PRDX5
E9PHN7	GSTM2	Glutathione S-transferase Mu 2	1.3	0.010	PRDX5
X1WI28	RPL10	60S ribosomal protein L10	1.3	0.012	PRDX5
Q00610	CLTC	Clathrin heavy chain 1	1.3	0.016	PRDX5
O75223	GGCT	Gamma-glutamylcyclotransferase	1.3	0.016	PRDX5
Q14232	EIF2B1	Translation initiation factor eIF-2B subunit alpha	1.3	0.016	PRDX5
P48634	PRRC2A	Protein PRRC2A	1.3	0.024	PRDX5
P35606	COPB2	Coatomer subunit beta'	1.3	0.031	PRDX5
Q9Y394	DHRS7	Dehydrogenase/reductase SDR family member 7	1.3	0.035	PRDX5
E5RGR0	LYPLA1	Acyl-protein thioesterase 1	1.3	0.041	PRDX5
Q9BXW7	HDHD5	Haloacid dehalogenase-like hydrolase domain-containing 5	1.2	0.000	PRDX5
P09211	GSTP1	Glutathione S-transferase P	1.2	0.001	PRDX5
Q14694	USP10	Ubiquitin carboxyl-terminal hydrolase 10	1.2	0.001	PRDX5
Q6NVY1	HIBCH	3-hydroxyisobutyryl-CoA hydrolase, mitochondrial	1.2	0.001	PRDX5
P62888	RPL30	60S ribosomal protein L30	1.2	0.001	PRDX5
P61586	RHOA	Transforming protein RhoA	1.2	0.001	PRDX5
P49327	FASN	Fatty acid synthase	1.2	0.001	PRDX5
Q15645	TRIP13	Pachytene checkpoint protein 2 homolog	1.2	0.006	PRDX5
Q9Y2R9	MRPS7	28S ribosomal protein S7, mitochondrial	1.2	0.007	PRDX5
Q00688	FKBP3	Peptidyl-prolyl cis-trans isomerase FKBP3	1.2	0.008	PRDX5
O75400	PRPF40A	Pre-mRNA-processing factor 40 homolog A	1.2	0.008	PRDX5
Q14240	EIF4A2	Eukaryotic initiation factor 4A-II	1.2	0.011	PRDX5
P42771	CDKN2A	Cyclin-dependent kinase inhibitor 2A	1.2	0.014	PRDX5
Q2TAL8	QRICH1	Glutamine-rich protein 1	1.2	0.017	PRDX5
Q5T7C4	HMGB1	High mobility group protein B1	1.2	0.022	PRDX5
P51570	GALK1	Galactokinase	1.2	0.025	PRDX5
P08758	ANXA5	Annexin A5	1.2	0.025	PRDX5
Q9NTJ5	SACM1L	Phosphatidylinositol-3-phosphatase SAC1	1.2	0.030	PRDX5
Q13283	G3BP1	Ras GTPase-activating protein-binding protein 1	1.2	0.032	PRDX5
Q9BW92	TARS2	Threonine--tRNA ligase, mitochondrial	1.2	0.033	PRDX5
P05141	SLC25A5	ADP/ATP translocase 2	1.1	0.000	PRDX5
P36578	RPL4	60S ribosomal protein L4	1.1	0.001	PRDX5
Q9NQ88	TIGAR	Fructose-2,6-bisphosphatase TIGAR	1.1	0.001	PRDX5
Q9Y2Z4	YARS2	Tyrosine--tRNA ligase, mitochondrial	1.1	0.001	PRDX5
P17858	PFKL	ATP-dependent 6-phosphofructokinase, liver type	1.1	0.001	PRDX5
O95456	PSMG1	Proteasome assembly chaperone 1	1.1	0.003	PRDX5
Q00796	SORD	Sorbitol dehydrogenase	1.1	0.004	PRDX5
P18077	RPL35A	60S ribosomal protein L35a	1.1	0.005	PRDX5
G5EA31	SEC24C	Protein transport protein Sec24C	1.1	0.007	PRDX5
Q13595	TRA2A	Transformer-2 protein homolog alpha	1.1	0.010	PRDX5
P63010	AP2B1	AP-2 complex subunit beta	1.1	0.010	PRDX5
O43795	MYO1B	Unconventional myosin-Ib	1.1	0.012	PRDX5
Q7Z2K8	GPRIN1	G protein-regulated inducer of neurite outgrowth 1	1.1	0.017	PRDX5
Q9GZT3	SLIRP	SRA stem-loop-interacting RNA-binding protein, mitochondrial	1.1	0.021	PRDX5
Q96F45	ZNF503	Zinc finger protein 503	1.1	0.023	PRDX5
P23258	TUBG1	Tubulin gamma-1 chain	1.1	0.024	PRDX5
Q9NR45	NANS	Sialic acid synthase	1.1	0.032	PRDX5
P62879	GNB2	Guanine nucleotide-binding protein G(I)/G(S)/G(T) subunit beta-2	1.1	0.033	PRDX5
P54886	ALDH18A1	Delta-1-pyrroline-5-carboxylate synthase	1.1	0.039	PRDX5
E7ETK0	RPS24	40S ribosomal protein S24	1.0	0.001	PRDX5
P62701	RPS4X	40S ribosomal protein S4, X isoform	1.0	0.006	PRDX5
E9PN17	ATP5MG	ATP synthase subunit g, mitochondrial	1.0	0.011	PRDX5
Q9BT25	HAUS8	HAUS augmin-like complex subunit 8	1.0	0.018	PRDX5
H0YDU8	PPP5C	Serine/threonine-protein phosphatase	1.0	0.027	PRDX5
O00483	NDUFA4	Cytochrome c oxidase subunit NDUFA4	1.0	0.029	PRDX5
P62820	RAB1A	Ras-related protein Rab-1A	1.0	0.032	PRDX5
Q6PKG0	LARP1	La-related protein 1	1.0	0.038	PRDX5
Q9HAV4	XPO5	Exportin-5	1.0	0.038	PRDX5
O94925	GLS	Glutaminase kidney isoform, mitochondrial	1.0	0.040	PRDX5
A0A1B0GW38	CBL	E3 ubiquitin-protein ligase CBL	1.0	0.045	PRDX5
**PRDX3 -specific interactors**					
P62805	H4C1	Histone H4	4.9	0.000	PRDX1
P02790	HPX	Hemopexin	4.5	0.000	PRDX1
O60506	SYNCRIP	Heterogeneous nuclear ribonucleoprotein Q	4.2	0.000	PRDX1
O76094	SRP72	Signal recognition particle subunit SRP72	4.2	0.000	PRDX1
P61106	RAB14	Ras-related protein Rab-14	4.0	0.000	PRDX1
P42704	LRPPRC	Leucine-rich PPR motif-containing protein, mitochondrial	3.9	0.000	PRDX1
P05387	RPLP2	60S acidic ribosomal protein P2	3.4	0.000	PRDX1
P63167	DYNLL1	Dynein light chain 1, cytoplasmic	3.3	0.000	PRDX1

Q4J6C6	PREPL	Prolyl endopeptidase-like	3.3	0.000	PRDX1
Q14257	RCN2	Reticulocalbin-2	3.3	0.000	PRDX1
P40616	ARL1	ADP-ribosylation factor-like protein 1	3.1	0.000	PRDX1
P35637	FUS	RNA-binding protein FUS	3.1	0.000	PRDX1
O95163	ELP1	Elongator complex protein 1	3.1	0.000	PRDX1
Q15388	TOMM20	Mitochondrial import receptor subunit TOM20 homolog	3.1	0.002	PRDX1
P05386	RPLP1	60S acidic ribosomal protein P1	3.0	0.000	PRDX1
P63244	RACK1	Receptor of activated protein C kinase 1	2.9	0.000	PRDX1
P78417	GSTO1	Glutathione S-transferase omega-1	2.9	0.000	PRDX1
Q8N5K1	CISD2	CDGSH iron-sulfur domain-containing protein 2	2.9	0.003	PRDX1
P61353	RPL27	60S ribosomal protein L27	2.8	0.000	PRDX1
P26196	DDX6	Probable ATP-dependent RNA helicase DDX6	2.8	0.000	PRDX1
P35606	COPB2	Coatomer subunit beta'	2.7	0.000	PRDX1
P16435	POR	NADPH--cytochrome P450 reductase	2.7	0.000	PRDX1
Q9UBF2	COPG2	Coatomer subunit gamma-2	2.7	0.000	PRDX1
P40429	RPL13A	60S ribosomal protein L13a	2.7	0.000	PRDX1
E9PHS0	LANCL1	Glutathione S-transferase LANCL1	2.7	0.001	PRDX1
Q8N2K0	ABHD12	Lysophosphatidylserine lipase ABHD12	2.7	0.003	PRDX1
O94925	GLS	Glutaminase kidney isoform, mitochondrial	2.6	0.000	PRDX1
P23588	EIF4B	Eukaryotic translation initiation factor 4B	2.6	0.000	PRDX1
P50502	ST13	Hsc70-interacting protein	2.6	0.000	PRDX1
Q9UBB4	ATXN10	Ataxin-10	2.6	0.000	PRDX1
Q07820	MCL1	Induced myeloid leukemia cell differentiation protein Mcl-1	2.6	0.001	PRDX1
P51148	RAB5C	Ras-related protein Rab-5C	2.6	0.005	PRDX1
Q9NVP1	DDX18	ATP-dependent RNA helicase DDX18	2.5	0.000	PRDX1
P16615	ATP2A2	Sarcoplasmic/endoplasmic reticulum calcium ATPase 2	2.5	0.000	PRDX1
Q96CS3	FAF2	FAS-associated factor 2	2.5	0.000	PRDX1
P98194	ATP2C1	Calcium-transporting ATPase type 2C member 1	2.5	0.001	PRDX1
Q14690	PDCD11	Protein RRP5 homolog	2.5	0.001	PRDX1
P68133	ACTA1	Actin, alpha skeletal muscle	2.5	0.003	PRDX1
Q7Z434	MAVS	Mitochondrial antiviral-signaling protein	2.5	0.003	PRDX1
P11388	TOP2A	DNA topoisomerase 2-alpha	2.4	0.000	PRDX1
Q8TEX9	IPO4	Importin-4	2.4	0.000	PRDX1
Q92769	HDAC2	Histone deacetylase 2	2.4	0.000	PRDX1
P19338	NCL	Nucleolin	2.4	0.000	PRDX1
G5EA06	MRPS27	28S ribosomal protein S27, mitochondrial	2.4	0.001	PRDX1
E7ETZ4	BZW2	Basic leucine zipper and W2 domain-containing protein 2	2.4	0.001	PRDX1
Q92621	NUP205	Nuclear pore complex protein Nup205	2.3	0.000	PRDX1
O14980	XPO1	Exportin-1	2.3	0.000	PRDX1
P62314	SNRPD1	Small nuclear ribonucleoprotein Sm D1	2.3	0.000	PRDX1
P27797	CALR	Calreticulin	2.3	0.000	PRDX1
Q9H3P7	ACBD3	Golgi resident protein GCP60	2.3	0.000	PRDX1
Q9ULC3	RAB23	Ras-related protein Rab-23	2.3	0.001	PRDX1
Q5QNY5	PEX19	Peroxin-19	2.3	0.004	PRDX1
Q93008	USP9X	Probable ubiquitin carboxyl-terminal hydrolase FAF-X	2.2	0.000	PRDX1
P41252	IARS1	Isoleucine--tRNA ligase, cytoplasmic	2.2	0.000	PRDX1
O95373	IPO7	Importin-7	2.2	0.000	PRDX1
Q15393	SF3B3	Splicing factor 3B subunit 3	2.2	0.000	PRDX1
Q9H0A0	NAT10	RNA cytidine acetyltransferase	2.2	0.000	PRDX1
O75832	PSMD10	26S proteasome non-ATPase regulatory subunit 10	2.2	0.000	PRDX1
Q92688	ANP32B	Acidic leucine-rich nuclear phosphoprotein 32 family member B	2.2	0.000	PRDX1
K7ERF1	EIF3K	Eukaryotic translation initiation factor 3 subunit K	2.2	0.000	PRDX1
Q5SY16	NOL9	Polynucleotide 5'-hydroxyl-kinase NOL9	2.2	0.001	PRDX1
Q96EY7	PTCD3	Pentatricopeptide repeat domain-containing protein 3, mitochondrial	2.2	0.001	PRDX1
P28288	ABCD3	ATP-binding cassette sub-family D member 3	2.2	0.001	PRDX1
Q8NI27	THOC2	THO complex subunit 2	2.2	0.001	PRDX1
O96008	TOMM40	Mitochondrial import receptor subunit TOM40 homolog	2.2	0.017	PRDX1
P08758	ANXA5	Annexin A5	2.1	0.000	PRDX1
Q5VW32	BROX	BRO1 domain-containing protein BROX	2.1	0.000	PRDX1
Q5VT66	MTARC1	Mitochondrial amidoxime-reducing component 1	2.1	0.000	PRDX1
Q9H8Y8	GORASP2	Golgi reassembly-stacking protein 2	2.1	0.000	PRDX1
P06730	EIF4E	Eukaryotic translation initiation factor 4E	2.1	0.000	PRDX1
O00203	AP3B1	AP-3 complex subunit beta-1	2.1	0.000	PRDX1
P61923	COPZ1	Coatomer subunit zeta-1	2.1	0.000	PRDX1
Q8TC07	TBC1D15	TBC1 domain family member 15	2.1	0.000	PRDX1
O94826	TOMM70	Mitochondrial import receptor subunit TOM70	2.1	0.000	PRDX1
P43034	PAFAH1B1	Platelet-activating factor acetylhydrolase IB subunit beta	2.1	0.000	PRDX1
Q01085	TIAL1	Nucleolysin TIAR	2.1	0.001	PRDX1
Q9BQG0	MYBBP1A	Myb-binding protein 1A	2.1	0.001	PRDX1
B3KUS5	USP30	Ubiquitin carboxyl-terminal hydrolase	2.1	0.002	PRDX1
Q8N4V1	MMGT1	Membrane magnesium transporter 1	2.1	0.002	PRDX1
D6RA00	ENOPH1	Enolase-phosphatase E1	2.1	0.002	PRDX1
O95104	SCAF4	SR-related and CTD-associated factor 4	2.1	0.002	PRDX1
Q8N335	GPD1L	Glycerol-3-phosphate dehydrogenase 1-like protein	2.1	0.002	PRDX1
A0A3B3ISV3	COL4A1	Collagen alpha-1(IV) chain	2.1	0.002	PRDX1
Q53GQ0	HSD17B12	Very-long-chain 3-oxoacyl-CoA reductase	2.1	0.003	PRDX1
A0A0A0MS29	MFF	Mitochondrial fission factor	2.1	0.004	PRDX1
Q8N806	UBR7	Putative E3 ubiquitin-protein ligase UBR7	2.1	0.006	PRDX1
P36957	DLST	Dihydrolipoyllysine-residue succinyltransferase component of 2-oxoglutarate dehydrogenase complex, mitochondrial	2.0	0.000	PRDX1
O75381	PEX14	Peroxisomal membrane protein PEX14	2.0	0.000	PRDX1
O94874	UFL1	E3 UFM1-protein ligase 1	2.0	0.000	PRDX1
Q9Y678	COPG1	Coatomer subunit gamma-1	2.0	0.000	PRDX1
O00410	IPO5	Importin-5	2.0	0.000	PRDX1
P12236	SLC25A6	ADP/ATP translocase 3	2.0	0.000	PRDX1
P14324	FDPS	Farnesyl pyrophosphate synthase	2.0	0.000	PRDX1
Q92973	TNPO1	Transportin-1	2.0	0.000	PRDX1
P98175	RBM10	RNA-binding protein 10	2.0	0.000	PRDX1
Q9BVP2	GNL3	Guanine nucleotide-binding protein-like 3	2.0	0.001	PRDX1
O15269	SPTLC1	Serine palmitoyltransferase 1	2.0	0.001	PRDX1
O75489	NDUFS3	NADH dehydrogenase [ubiquinone] iron-sulfur protein 3, mitochondrial	2.0	0.002	PRDX1
Q8N6T3	ARFGAP1	ADP-ribosylation factor GTPase-activating protein 1	2.0	0.003	PRDX1
O15144	ARPC2	Actin-related protein 2/3 complex subunit 2	2.0	0.003	PRDX1
Q9NQ29	LUC7L	Putative RNA-binding protein Luc7-like 1	2.0	0.003	PRDX1
Q9H9P8	L2HGDH	L-2-hydroxyglutarate dehydrogenase, mitochondrial	2.0	0.003	PRDX1
P19174	PLCG1	1-phosphatidylinositol 4,5-bisphosphate phosphodiesterase gamma-1	2.0	0.003	PRDX1
Q9BSJ8	ESYT1	Extended synaptotagmin-1	2.0	0.003	PRDX1
P51809	VAMP7	Vesicle-associated membrane protein 7	2.0	0.015	PRDX1
P30048	PRDX3	Thioredoxin-dependent peroxide reductase, mitochondrial	14.7	0.000	PRDX1
O94905	ERLIN2	Erlin-2	1.9	0.000	PRDX1
P15374	UCHL3	Ubiquitin carboxyl-terminal hydrolase isozyme L3	1.9	0.000	PRDX1
P49321	NASP	Nuclear autoantigenic sperm protein	1.9	0.000	PRDX1
E7ESY4	MTA1	Metastasis-associated protein MTA1	1.9	0.000	PRDX1
P42166	TMPO	Lamina-associated polypeptide 2, isoform alpha	1.9	0.000	PRDX1
P00387	CYB5R3	NADH-cytochrome b5 reductase 3	1.9	0.000	PRDX1
P35237	SERPINB6	Serpin B6	1.9	0.001	PRDX1
Q9Y679	AUP1	Lipid droplet-regulating VLDL assembly factor AUP1	1.9	0.002	PRDX1
O96019	ACTL6A	Actin-like protein 6A	1.9	0.003	PRDX1
B9A018	USP39	U4/U6.U5 tri-snRNP-associated protein 2	1.9	0.003	PRDX1
Q9BZK7	TBL1XR1	F-box-like/WD repeat-containing protein TBL1XR1	1.9	0.003	PRDX1
Q5JR04	MOV10	RNA helicase	1.9	0.004	PRDX1
P51398	DAP3	28S ribosomal protein S29, mitochondrial	1.9	0.005	PRDX1
Q13190	STX5	Syntaxin-5	1.9	0.005	PRDX1
E9PKG1	PRMT1	Protein arginine N-methyltransferase 1	1.9	0.006	PRDX1
P84085	ARF5	ADP-ribosylation factor 5	1.9	0.011	PRDX1
Q9NQG6	MIEF1	Mitochondrial dynamics protein MID51	1.9	0.014	PRDX1
P13667	PDIA4	Protein disulfide-isomerase A4	1.9	0.017	PRDX1
Q14964	RAB39A	Ras-related protein Rab-39A	1.9	0.039	PRDX1
P08243	ASNS	Asparagine synthetase [glutamine-hydrolyzing]	1.8	0.000	PRDX1
Q9UIG0	BAZ1B	Tyrosine-protein kinase BAZ1B	1.8	0.000	PRDX1
O00268	TAF4	Transcription initiation factor TFIID subunit 4	1.8	0.000	PRDX1
P09211	GSTP1	Glutathione S-transferase P	1.8	0.000	PRDX1
G8JLH9	STAT3	Signal transducer and activator of transcription	1.8	0.000	PRDX1
Q8TC12	RDH11	Retinol dehydrogenase 11	1.8	0.000	PRDX1
Q9HAV4	XPO5	Exportin-5	1.8	0.001	PRDX1
O00231	PSMD11	26S proteasome non-ATPase regulatory subunit 11	1.8	0.001	PRDX1
P31689	DNAJA1	DnaJ homolog subfamily A member 1	1.8	0.001	PRDX1
O75131	CPNE3	Copine-3	1.8	0.001	PRDX1
Q5VYK3	ECPAS	Proteasome adapter and scaffold protein ECM29	1.8	0.004	PRDX1
Q9P287	BCCIP	BRCA2 and CDKN1A-interacting protein	1.8	0.006	PRDX1
O00483	NDUFA4	Cytochrome c oxidase subunit NDUFA4	1.8	0.006	PRDX1
P07384	CAPN1	Calpain-1 catalytic subunit	1.8	0.006	PRDX1
Q13951	CBFB	Core-binding factor subunit beta	1.8	0.007	PRDX1
G3V1C3	API5	Apoptosis inhibitor 5	1.8	0.007	PRDX1
Q15293	RCN1	Reticulocalbin-1	1.8	0.008	PRDX1
Q9HB07	MYG1	MYG1 exonuclease	1.8	0.008	PRDX1
P46940	IQGAP1	Ras GTPase-activating-like protein IQGAP1	1.8	0.009	PRDX1
H0Y5L2	ECHDC1	Ethylmalonyl-CoA decarboxylase	1.8	0.011	PRDX1
P56962	STX17	Syntaxin-17	1.8	0.014	PRDX1
O60502	OGA	Protein O-GlcNAcase	1.8	0.014	PRDX1
Q8NF37	LPCAT1	Lysophosphatidylcholine acyltransferase 1	1.8	0.018	PRDX1
Q9Y657	SPIN1	Spindlin-1	1.8	0.028	PRDX1
A0A0B4J1Z1	SRSF7	Serine/arginine-rich-splicing factor 7	1.8	0.034	PRDX1
O95376	ARIH2	E3 ubiquitin-protein ligase ARIH2	1.7	0.000	PRDX1
P11387	TOP1	DNA topoisomerase 1	1.7	0.000	PRDX1
O43707	ACTN4	Alpha-actinin-4	1.7	0.000	PRDX1
Q14232	EIF2B1	Translation initiation factor eIF-2B subunit alpha	1.7	0.000	PRDX1
P53618	COPB1	Coatomer subunit beta	1.7	0.000	PRDX1
C9J4Z3	RPL37A	60S ribosomal protein L37a	1.7	0.000	PRDX1
P38117	ETFB	Electron transfer flavoprotein subunit beta	1.7	0.000	PRDX1
O95573	ACSL3	Long-chain-fatty-acid--CoA ligase 3	1.7	0.000	PRDX1
Q15691	MAPRE1	Microtubule-associated protein RP/EB family member 1	1.7	0.000	PRDX1
Q9NV70	EXOC1	Exocyst complex component 1	1.7	0.000	PRDX1
P15121	AKR1B1	Aldo-keto reductase family 1 member B1	1.7	0.000	PRDX1
Q9UKG1	APPL1	DCC-interacting protein 13-alpha	1.7	0.000	PRDX1
O95292	VAPB	Vesicle-associated membrane protein-associated protein B/C	1.7	0.000	PRDX1
Q16531	DDB1	DNA damage-binding protein 1	1.7	0.001	PRDX1
P42224	STAT1	Signal transducer and activator of transcription 1-alpha/beta	1.7	0.002	PRDX1
G3V529	DDX24	RNA helicase	1.7	0.003	PRDX1
Q9BXJ9	NAA15	N-alpha-acetyltransferase 15, NatA auxiliary subunit	1.7	0.006	PRDX1
P22830	FECH	Ferrochelatase, mitochondrial	1.7	0.007	PRDX1
Q9Y262	EIF3L	Eukaryotic translation initiation factor 3 subunit L	1.7	0.007	PRDX1
P04844	RPN2	Dolichyl-diphosphooligosaccharide--protein glycosyltransferase subunit 2	1.7	0.010	PRDX1
D6RB59	EXOC3	Exocyst complex component 3	1.7	0.013	PRDX1
Q9NZ32	ACTR10	Actin-related protein 10	1.7	0.014	PRDX1
Q15717	ELAVL1	ELAV-like protein 1	1.7	0.014	PRDX1
Q9NWB6	ARGLU1	Arginine and glutamate-rich protein 1	1.7	0.015	PRDX1
Q96IU4	ABHD14B	Protein ABHD14B	1.7	0.016	PRDX1
Q14789	GOLGB1	Golgin subfamily B member 1	1.7	0.016	PRDX1
P35579	MYH9	Myosin-9	1.7	0.026	PRDX1
Q8N573	OXR1	Oxidation resistance protein 1	1.7	0.031	PRDX1
J3KPG2	TPT1	Translationally-controlled tumor protein	1.7	0.037	PRDX1
Q9Y3Z3	SAMHD1	Deoxynucleoside triphosphate triphosphohydrolase SAMHD1	1.6	0.000	PRDX1
P05455	SSB	Lupus La protein	1.6	0.000	PRDX1
G5EA31	SEC24C	Protein transport protein Sec24C	1.6	0.000	PRDX1
P23921	RRM1	Ribonucleoside-diphosphate reductase large subunit	1.6	0.000	PRDX1
P04181	OAT	Ornithine aminotransferase, mitochondrial	1.6	0.001	PRDX1
Q15054	POLD3	DNA polymerase delta subunit 3	1.6	0.001	PRDX1
P55769	SNU13	NHP2-like protein 1	1.6	0.001	PRDX1
Q03701	CEBPZ	CCAAT/enhancer-binding protein zeta	1.6	0.002	PRDX1
Q9Y3T9	NOC2L	Nucleolar complex protein 2 homolog	1.6	0.002	PRDX1
P33992	MCM5	DNA replication licensing factor MCM5	1.6	0.003	PRDX1
P18031	PTPN1	Tyrosine-protein phosphatase non-receptor type 1	1.6	0.004	PRDX1
P22314	UBA1	Ubiquitin-like modifier-activating enzyme 1	1.6	0.005	PRDX1
Q8WTT2	NOC3L	Nucleolar complex protein 3 homolog	1.6	0.006	PRDX1
Q96T23	RSF1	Remodeling and spacing factor 1	1.6	0.007	PRDX1
O15397	IPO8	Importin-8	1.6	0.012	PRDX1
Q5T3Q7	HEATR1	HEAT repeat-containing protein 1	1.6	0.013	PRDX1
X6RM00	ERC1	ELKS/Rab6-interacting/CAST family member 1	1.6	0.013	PRDX1
J3QRD1	ALDH3A2	Aldehyde dehydrogenase family 3 member A2	1.6	0.014	PRDX1
Q9NYB0	TERF2IP	Telomeric repeat-binding factor 2-interacting protein 1	1.6	0.014	PRDX1
H3BV80	RNPS1	RNA-binding protein with serine-rich domain 1	1.6	0.015	PRDX1
Q9Y6Y0	IVNS1ABP	Influenza virus NS1A-binding protein	1.6	0.016	PRDX1
Q14204	DYNC1H1	Cytoplasmic dynein 1 heavy chain 1	1.6	0.017	PRDX1
Q9NVI1	FANCI	Fanconi anemia group I protein	1.6	0.018	PRDX1
Q9NP72	RAB18	Ras-related protein Rab-18	1.6	0.024	PRDX1
Q01082	SPTBN1	Spectrin beta chain, non-erythrocytic 1	1.6	0.024	PRDX1
P52565	ARHGDIA	Rho GDP-dissociation inhibitor 1	1.5	0.000	PRDX1
P53004	BLVRA	Biliverdin reductase A	1.5	0.000	PRDX1
O75165	DNAJC13	DnaJ homolog subfamily C member 13	1.5	0.000	PRDX1
P08240	SRPRA	Signal recognition particle receptor subunit alpha	1.5	0.000	PRDX1
Q07020	RPL18	60S ribosomal protein L18	1.5	0.000	PRDX1
Q9BT78	COPS4	COP9 signalosome complex subunit 4	1.5	0.000	PRDX1
Q9UQ80	PA2G4	Proliferation-associated protein 2G4	1.5	0.000	PRDX1
Q14974	KPNB1	Importin subunit beta-1	1.5	0.000	PRDX1
O00299	CLIC1	Chloride intracellular channel protein 1	1.5	0.000	PRDX1
O60763	USO1	General vesicular transport factor p115	1.5	0.000	PRDX1
Q9UL25	RAB21	Ras-related protein Rab-21	1.5	0.001	PRDX1
P30101	PDIA3	Protein disulfide-isomerase A3	1.5	0.002	PRDX1
O43747	AP1G1	AP-1 complex subunit gamma-1	1.5	0.002	PRDX1
Q10713	PMPCA	Mitochondrial-processing peptidase subunit alpha	1.5	0.002	PRDX1
Q14318	FKBP8	Peptidyl-prolyl cis-trans isomerase FKBP8	1.5	0.002	PRDX1
P48735	IDH2	Isocitrate dehydrogenase [NADP], mitochondrial	1.5	0.003	PRDX1
Q86UP2	KTN1	Kinectin	1.5	0.003	PRDX1
O00505	KPNA3	Importin subunit alpha-4	1.5	0.003	PRDX1
P62750	RPL23A	60S ribosomal protein L23a	1.5	0.003	PRDX1
Q02750	MAP2K1	Dual specificity mitogen-activated protein kinase kinase 1	1.5	0.004	PRDX1
P39023	RPL3	60S ribosomal protein L3	1.5	0.004	PRDX1
P04843	RPN1	Dolichyl-diphosphooligosaccharide--protein glycosyltransferase subunit 1	1.5	0.004	PRDX1
Q15075	EEA1	Early endosome antigen 1	1.5	0.005	PRDX1
Q9NW13	RBM28	RNA-binding protein 28	1.5	0.006	PRDX1
A0A0A0MR02	VDAC2	Outer mitochondrial membrane protein porin 2	1.5	0.008	PRDX1
O60264	SMARCA5	SWI/SNF-related matrix-associated actin-dependent regulator of chromatin subfamily A member 5	1.5	0.009	PRDX1
O00232	PSMD12	26S proteasome non-ATPase regulatory subunit 12	1.5	0.011	PRDX1
P11182	DBT	Lipoamide acyltransferase component of branched-chain alpha-keto acid dehydrogenase complex, mitochondrial	1.5	0.015	PRDX1
Q9UHQ9	CYB5R1	NADH-cytochrome b5 reductase 1	1.5	0.018	PRDX1
H0YLH3	RABGGTA	Geranylgeranyl transferase type-2 subunit alpha	1.5	0.021	PRDX1
Q9UBS4	DNAJB11	DnaJ homolog subfamily B member 11	1.5	0.022	PRDX1
Q8NCM8	DYNC2H1	Cytoplasmic dynein 2 heavy chain 1	1.5	0.024	PRDX1
C9J384	CMSS1	Protein CMSS1	1.5	0.024	PRDX1
Q9Y4P1	ATG4B	Cysteine protease ATG4B	1.5	0.024	PRDX1
K7ESE9	BCAS3	Breast carcinoma-amplified sequence 3	1.5	0.025	PRDX1
P62906	RPL10A	60S ribosomal protein L10a	1.5	0.028	PRDX1
P48449	LSS	Lanosterol synthase	1.5	0.046	PRDX1
Q99459	CDC5L	Cell division cycle 5-like protein	1.5	0.049	PRDX1
Q92600	CNOT9	CCR4-NOT transcription complex subunit 9	1.4	0.000	PRDX1
P51665	PSMD7	26S proteasome non-ATPase regulatory subunit 7	1.4	0.000	PRDX1
Q96P70	IPO9	Importin-9	1.4	0.000	PRDX1
Q9UBU9	NXF1	Nuclear RNA export factor 1	1.4	0.000	PRDX1
P32969	RPL9	60S ribosomal protein L9	1.4	0.000	PRDX1
O76031	CLPX	ATP-dependent Clp protease ATP-binding subunit clpX-like, mitochondrial	1.4	0.000	PRDX1
Q5JXI8	FHL1	Four and a half LIM domains protein 1	1.4	0.000	PRDX1
E5RHG8	ELOC	Elongin-C	1.4	0.000	PRDX1
P62424	RPL7A	60S ribosomal protein L7a	1.4	0.000	PRDX1
Q6PI48	DARS2	Aspartate--tRNA ligase, mitochondrial	1.4	0.001	PRDX1
P53985	SLC16A1	Monocarboxylate transporter 1	1.4	0.001	PRDX1
Q15008	PSMD6	26S proteasome non-ATPase regulatory subunit 6	1.4	0.001	PRDX1
P54136	RARS1	Arginine--tRNA ligase, cytoplasmic	1.4	0.001	PRDX1
P62333	PSMC6	26S proteasome regulatory subunit 10B	1.4	0.002	PRDX1
Q9UJS0	SLC25A13	Calcium-binding mitochondrial carrier protein Aralar2	1.4	0.002	PRDX1
Q96QK1	VPS35	Vacuolar protein sorting-associated protein 35	1.4	0.002	PRDX1
Q9UHB9	SRP68	Signal recognition particle subunit SRP68	1.4	0.002	PRDX1
Q96H79	ZC3HAV1L	Zinc finger CCCH-type antiviral protein 1-like	1.4	0.002	PRDX1
O00567	NOP56	Nucleolar protein 56	1.4	0.003	PRDX1
P08133	ANXA6	Annexin A6	1.4	0.003	PRDX1
P62244	RPS15A	40S ribosomal protein S15a	1.4	0.003	PRDX1
Q15291	RBBP5	Retinoblastoma-binding protein 5	1.4	0.004	PRDX1
P20073	ANXA7	Annexin A7	1.4	0.006	PRDX1
P52597	HNRNPF	Heterogeneous nuclear ribonucleoprotein F	1.4	0.006	PRDX1
Q9P0M6	MACROH2A2	Core histone macro-H2A.2	1.4	0.008	PRDX1
O43719	HTATSF1	HIV Tat-specific factor 1	1.4	0.010	PRDX1
F8W038	C17orf49	Chromosome 17 open reading frame 49	1.4	0.015	PRDX1
Q92667	AKAP1	A-kinase anchor protein 1, mitochondrial	1.4	0.022	PRDX1
Q6UWP7	LCLAT1	Lysocardiolipin acyltransferase 1	1.4	0.024	PRDX1
O96017	CHEK2	Serine/threonine-protein kinase Chk2	1.4	0.032	PRDX1
Q12769	NUP160	Nuclear pore complex protein Nup160	1.4	0.034	PRDX1
O75400	PRPF40A	Pre-mRNA-processing factor 40 homolog A	1.4	0.034	PRDX1
A0A2Q2TH77	A0A2Q2TH77	>tr|A0A2Q2TH77|A0A2Q2TH77_HUMAN Golgin subfamily A member 2 OS=Homo sapiens OX=9606 GN=GOLGA2 PE=1 SV=1;>sp|Q08379|GOGA2_HUMAN Golgin subfamily A member 2 OS=Homo sapiens OX=9606 GN=GOLGA2 PE=1 SV=3;>tr|A0A1W2PQY5|A0A1W2PQY5_HUMAN Golgin subfamily A member	1.4	0.040	PRDX1
Q8NDI1	EHBP1	EH domain-binding protein 1	1.4	0.045	PRDX1
P78347	GTF2I	General transcription factor II-I	1.4	0.046	PRDX1
P21796	VDAC1	Voltage-dependent anion-selective channel protein 1	1.4	0.048	PRDX1
Q9Y580	RBM7	RNA-binding protein 7	1.4	0.049	PRDX1
Q6WCQ1	MPRIP	Myosin phosphatase Rho-interacting protein	1.3	0.000	PRDX1
Q15029	EFTUD2	116 kDa U5 small nuclear ribonucleoprotein component	1.3	0.000	PRDX1
Q9BRA2	TXNDC17	Thioredoxin domain-containing protein 17	1.3	0.000	PRDX1
P53007	SLC25A1	Tricarboxylate transport protein, mitochondrial	1.3	0.000	PRDX1
P34949	MPI	Mannose-6-phosphate isomerase	1.3	0.000	PRDX1
P17987	TCP1	T-complex protein 1 subunit alpha	1.3	0.000	PRDX1
A6NKB8	RNPEP	Aminopeptidase B	1.3	0.000	PRDX1
J3QL56	SCO1	Protein SCO1 homolog, mitochondrial	1.3	0.000	PRDX1
P61247	RPS3A	40S ribosomal protein S3a	1.3	0.000	PRDX1
P23396	RPS3	40S ribosomal protein S3	1.3	0.000	PRDX1
Q00325	SLC25A3	Phosphate carrier protein, mitochondrial	1.3	0.000	PRDX1
Q12788	TBL3	Transducin beta-like protein 3	1.3	0.000	PRDX1
P43246	MSH2	DNA mismatch repair protein Msh2	1.3	0.001	PRDX1
P60228	EIF3E	Eukaryotic translation initiation factor 3 subunit E	1.3	0.001	PRDX1
O60934	NBN	Nibrin	1.3	0.001	PRDX1
Q9UIA9	XPO7	Exportin-7	1.3	0.003	PRDX1
P18077	RPL35A	60S ribosomal protein L35a	1.3	0.004	PRDX1
Q9UI12	ATP6V1H	V-type proton ATPase subunit H	1.3	0.005	PRDX1
E9PKP7	UBTF	Nucleolar transcription factor 1	1.3	0.005	PRDX1
Q12996	CSTF3	Cleavage stimulation factor subunit 3	1.3	0.005	PRDX1
Q9Y5J1	UTP18	U3 small nucleolar RNA-associated protein 18 homolog	1.3	0.005	PRDX1
Q9Y2Q3	GSTK1	Glutathione S-transferase kappa 1	1.3	0.006	PRDX1
Q00610	CLTC	Clathrin heavy chain 1	1.3	0.008	PRDX1
O95861	BPNT1	3'(2'),5'-bisphosphate nucleotidase 1	1.3	0.009	PRDX1
O95202	LETM1	Mitochondrial proton/calcium exchanger protein	1.3	0.013	PRDX1
O15042	U2SURP	U2 snRNP-associated SURP motif-containing protein	1.3	0.015	PRDX1
P47897	QARS1	Glutamine--tRNA ligase	1.3	0.018	PRDX1
Q9Y6Y8	SEC23IP	SEC23-interacting protein	1.3	0.018	PRDX1
O00629	KPNA4	Importin subunit alpha-3	1.3	0.022	PRDX1
Q9UG63	ABCF2	ATP-binding cassette sub-family F member 2	1.3	0.023	PRDX1
Q9Y2H1	STK38L	Serine/threonine-protein kinase 38-like	1.3	0.033	PRDX1
Q96T88	UHRF1	E3 ubiquitin-protein ligase UHRF1	1.3	0.045	PRDX1
Q9BYJ9	YTHDF1	YTH domain-containing family protein 1	1.3	0.046	PRDX1
O75367	MACROH2A1	Core histone macro-H2A.1	1.3	0.046	PRDX1
Q8NBY1	STK26	Serine/threonine-protein kinase 26	1.3	0.046	PRDX1
P08572	COL4A2	Collagen alpha-2(IV) chain [Cleaved into: Canstatin]	1.3	0.046	PRDX1
Q86TI2	DPP9	Dipeptidyl peptidase 9	1.3	0.047	PRDX1
Q13439	GOLGA4	Golgin subfamily A member 4	1.3	0.049	PRDX1
P56537	EIF6	Eukaryotic translation initiation factor 6	1.2	0.000	PRDX1
P04899	GNAI2	Guanine nucleotide-binding protein G(i) subunit alpha-2	1.2	0.000	PRDX1
P51149	RAB7A	Ras-related protein Rab-7a	1.2	0.000	PRDX1
O75396	SEC22B	Vesicle-trafficking protein SEC22b	1.2	0.001	PRDX1
Q99613	EIF3C	Eukaryotic translation initiation factor 3 subunit C	1.2	0.001	PRDX1
Q99567	NUP88	Nuclear pore complex protein Nup88	1.2	0.003	PRDX1
Q5JY65	CRNKL1	Crooked neck-like protein 1	1.2	0.003	PRDX1
P06744	GPI	Glucose-6-phosphate isomerase	1.2	0.003	PRDX1
P31943	HNRNPH1	Heterogeneous nuclear ribonucleoprotein H	1.2	0.004	PRDX1
Q5T760	SRSF11	Serine/arginine-rich-splicing factor 11	1.2	0.005	PRDX1
Q6IBS0	TWF2	Twinfilin-2	1.2	0.006	PRDX1
O94973	AP2A2	AP-2 complex subunit alpha-2	1.2	0.007	PRDX1
P43897	TSFM	Elongation factor Ts, mitochondrial	1.2	0.008	PRDX1
O60678	PRMT3	Protein arginine N-methyltransferase 3	1.2	0.009	PRDX1
P56192	MARS1	Methionine--tRNA ligase, cytoplasmic	1.2	0.009	PRDX1
P46199	MTIF2	Translation initiation factor IF-2, mitochondrial	1.2	0.010	PRDX1
Q9H8H0	NOL11	Nucleolar protein 11	1.2	0.010	PRDX1
Q9H0E2	TOLLIP	Toll-interacting protein	1.2	0.013	PRDX1
P0DPB6	POLR1D	DNA-directed RNA polymerases I and III subunit RPAC2	1.2	0.013	PRDX1
Q53H12	AGK	Acylglycerol kinase, mitochondrial	1.2	0.015	PRDX1
O60306	AQR	RNA helicase aquarius	1.2	0.016	PRDX1
Q9Y5L0	TNPO3	Transportin-3	1.2	0.019	PRDX1
P35659	DEK	Protein DEK	1.2	0.019	PRDX1
P62979	RPS27A	Ubiquitin-40S ribosomal protein S27a	1.2	0.022	PRDX1
O75688	PPM1B	Protein phosphatase 1B	1.2	0.025	PRDX1
Q9NQG5	RPRD1B	Regulation of nuclear pre-mRNA domain-containing protein 1B	1.2	0.025	PRDX1
P23284	PPIB	Peptidyl-prolyl cis-trans isomerase B	1.2	0.026	PRDX1
P30740	SERPINB1	Leukocyte elastase inhibitor	1.2	0.027	PRDX1
P27824	CANX	Calnexin	1.2	0.032	PRDX1
Q9Y2X3	NOP58	Nucleolar protein 58	1.2	0.036	PRDX1
Q9BTT0	ANP32E	Acidic leucine-rich nuclear phosphoprotein 32 family member E	1.2	0.038	PRDX1
E7EVH7	E7EVH7	Kinesin light chain	1.2	0.045	PRDX1
M0R026	ILVBL	2-hydroxyacyl-CoA lyase 2	1.1	0.000	PRDX1
Q99733	NAP1L4	Nucleosome assembly protein 1-like 4	1.1	0.000	PRDX1
P09960	LTA4H	Leukotriene A-4 hydrolase	1.1	0.000	PRDX1
P05141	SLC25A5	ADP/ATP translocase 2	1.1	0.000	PRDX1
Q9BTX1	NDC1	Nucleoporin NDC1	1.1	0.000	PRDX1
Q7Z4H7	HAUS6	HAUS augmin-like complex subunit 6	1.1	0.001	PRDX1
J3QLD9	FLOT2	Flotillin	1.1	0.001	PRDX1
Q96A33	CCDC47	Coiled-coil domain-containing protein 47	1.1	0.001	PRDX1
C9J8Q5	ALDH5A1	Succinate-semialdehyde dehydrogenase	1.1	0.001	PRDX1
P49327	FASN	Fatty acid synthase	1.1	0.001	PRDX1
P07814	EPRS1	Bifunctional glutamate/proline--tRNA ligase	1.1	0.002	PRDX1
Q9NQ88	TIGAR	Fructose-2,6-bisphosphatase TIGAR	1.1	0.002	PRDX1
P31942	HNRNPH3	Heterogeneous nuclear ribonucleoprotein H3	1.1	0.004	PRDX1
E9PIF2	DDX10	RNA helicase	1.1	0.005	PRDX1
P35573	AGL	Glycogen debranching enzyme	1.1	0.006	PRDX1
O95782	AP2A1	AP-2 complex subunit alpha-1	1.1	0.006	PRDX1
O75874	IDH1	Isocitrate dehydrogenase [NADP] cytoplasmic	1.1	0.006	PRDX1
A0A024R4M0	RPS9	40S ribosomal protein S9	1.1	0.006	PRDX1
Q9UNM6	PSMD13	26S proteasome non-ATPase regulatory subunit 13	1.1	0.007	PRDX1
P52948	NUP98	Nuclear pore complex protein Nup98-Nup96	1.1	0.008	PRDX1
Q5T3I0	GPATCH4	G patch domain-containing protein 4	1.1	0.008	PRDX1
P46087	NOP2	Probable 28S rRNA	1.1	0.010	PRDX1
Q13085	ACACA	Acetyl-CoA carboxylase 1	1.1	0.011	PRDX1
Q9Y4E8	USP15	Ubiquitin carboxyl-terminal hydrolase 15	1.1	0.014	PRDX1
A0A2R8Y566	RELCH	RAB11-binding protein RELCH	1.1	0.015	PRDX1
O15226	NKRF	NF-kappa-B-repressing factor	1.1	0.015	PRDX1
Q71UM5	RPS27L	40S ribosomal protein S27-like	1.1	0.015	PRDX1
O95433	AHSA1	Activator of 90 kDa heat shock protein ATPase homolog 1	1.1	0.017	PRDX1
Q9Y371	SH3GLB1	Endophilin-B1	1.1	0.018	PRDX1
Q9BYG3	NIFK	MKI67 FHA domain-interacting nucleolar phosphoprotein	1.1	0.019	PRDX1
O14776	TCERG1	Transcription elongation regulator 1	1.1	0.020	PRDX1
P08670	VIM	Vimentin	1.1	0.023	PRDX1
P39687	ANP32A	Acidic leucine-rich nuclear phosphoprotein 32 family member A	1.1	0.024	PRDX1
P52209	PGD	6-phosphogluconate dehydrogenase, decarboxylating	1.1	0.025	PRDX1
Q99426	TBCB	Tubulin-folding cofactor B	1.1	0.025	PRDX1
P26583	HMGB2	High mobility group protein B2	1.1	0.025	PRDX1
Q96TC7	RMDN3	Regulator of microtubule dynamics protein 3	1.1	0.032	PRDX1
Q08945	SSRP1	FACT complex subunit SSRP1	1.1	0.033	PRDX1
Q6IN85	PPP4R3A	Serine/threonine-protein phosphatase 4 regulatory subunit 3A	1.1	0.034	PRDX1
Q9H0U4	RAB1B	Ras-related protein Rab-1B	1.1	0.034	PRDX1
P62258	YWHAE	14-3-3 protein epsilon	1.1	0.034	PRDX1
F8W0J6	NAP1L1	Nucleosome assembly protein 1-like 1	1.1	0.034	PRDX1
Q9C0B0	UNK	RING finger protein unkempt homolog	1.1	0.034	PRDX1
Q8IZ73	RPUSD2	RNA pseudouridylate synthase domain-containing protein 2	1.1	0.036	PRDX1
O43776	NARS1	Asparagine--tRNA ligase, cytoplasmic	1.0	0.000	PRDX1
P23528	CFL1	Cofilin-1	1.0	0.000	PRDX1
Q5H928	HSD17B10	3-hydroxyacyl-CoA dehydrogenase type-2	1.0	0.000	PRDX1
O95168	NDUFB4	NADH dehydrogenase [ubiquinone] 1 beta subcomplex subunit 4	1.0	0.001	PRDX1
P62136	PPP1CA	Serine/threonine-protein phosphatase PP1-alpha catalytic subunit	1.0	0.001	PRDX1
P68104	EEF1A1	Elongation factor 1-alpha 1	1.0	0.002	PRDX1
A0A087WXC5	NDUFA10	NADH dehydrogenase [ubiquinone] 1 alpha subcomplex subunit 10, mitochondrial	1.0	0.002	PRDX1
O75083	WDR1	WD repeat-containing protein 1	1.0	0.004	PRDX1
P31040	SDHA	Succinate dehydrogenase [ubiquinone] flavoprotein subunit, mitochondrial	1.0	0.005	PRDX1
Q9BV20	MRI1	Methylthioribose-1-phosphate isomerase	1.0	0.007	PRDX1
P60953	CDC42	Cell division control protein 42 homolog	1.0	0.008	PRDX1
P55060	CSE1L	Exportin-2	1.0	0.011	PRDX1
Q09161	NCBP1	Nuclear cap-binding protein subunit 1	1.0	0.024	PRDX1
P15927	RPA2	Replication protein A 32 kDa subunit	1.0	0.027	PRDX1
O00273	DFFA	DNA fragmentation factor subunit alpha	1.0	0.027	PRDX1
Q9NVX2	NLE1	Notchless protein homolog 1	1.0	0.034	PRDX1
O76003	GLRX3	Glutaredoxin-3	1.0	0.036	PRDX1
O60885	BRD4	Bromodomain-containing protein 4	1.0	0.046	PRDX1
P30048	PRDX3	Thioredoxin-dependent peroxide reductase, mitochondrial	8.9	0.000	PRDX2
Q15388	TOMM20	Mitochondrial import receptor subunit TOM20 homolog	3.6	0.001	PRDX2
P78417	GSTO1	Glutathione S-transferase omega-1	2.8	0.000	PRDX2
Q8N2K0	ABHD12	Lysophosphatidylserine lipase ABHD12	2.8	0.003	PRDX2
P30519	HMOX2	Heme oxygenase 2	2.7	0.007	PRDX2
P98175	RBM10	RNA-binding protein 10	2.6	0.000	PRDX2
Q8N5K1	CISD2	CDGSH iron-sulfur domain-containing protein 2	2.6	0.007	PRDX2
Q07820	MCL1	Induced myeloid leukemia cell differentiation protein Mcl-1	2.5	0.000	PRDX2
B3KUS5	USP30	Ubiquitin carboxyl-terminal hydrolase	2.5	0.000	PRDX2
A0A3B3ITJ4	HNRNPL	Heterogeneous nuclear ribonucleoprotein L	2.5	0.001	PRDX2
Q16352	INA	Alpha-internexin	2.3	0.000	PRDX2
Q8N6T3	ARFGAP1	ADP-ribosylation factor GTPase-activating protein 1	2.3	0.001	PRDX2
Q8N4V1	MMGT1	Membrane magnesium transporter 1	2.3	0.002	PRDX2
A0A0A0MS29	MFF	Mitochondrial fission factor	2.3	0.003	PRDX2
A0A0A0MR02	VDAC2	Outer mitochondrial membrane protein porin 2	2.2	0.000	PRDX2
Q14257	RCN2	Reticulocalbin-2	2.2	0.001	PRDX2
Q9Y2H1	STK38L	Serine/threonine-protein kinase 38-like	2.2	0.008	PRDX2
O96008	TOMM40	Mitochondrial import receptor subunit TOM40 homolog	2.2	0.021	PRDX2
Q13464	ROCK1	Rho-associated protein kinase 1	2.1	0.000	PRDX2
Q9NQG6	MIEF1	Mitochondrial dynamics protein MID51	2.1	0.010	PRDX2
Q15075	EEA1	Early endosome antigen 1	2.0	0.000	PRDX2
O75688	PPM1B	Protein phosphatase 1B	2.0	0.001	PRDX2
H0Y5L2	ECHDC1	Ethylmalonyl-CoA decarboxylase	2.0	0.008	PRDX2
P56962	STX17	Syntaxin-17	2.0	0.010	PRDX2
Q14789	GOLGB1	Golgin subfamily B member 1	2.0	0.011	PRDX2
Q9Y657	SPIN1	Spindlin-1	2.0	0.021	PRDX2
P51148	RAB5C	Ras-related protein Rab-5C	2.0	0.021	PRDX2
Q5SZR4	TDRKH	Tudor and KH domain containing, isoform CRA_a	2.0	0.040	PRDX2
O00268	TAF4	Transcription initiation factor TFIID subunit 4	1.9	0.000	PRDX2
Q9Y679	AUP1	Lipid droplet-regulating VLDL assembly factor AUP1	1.9	0.002	PRDX2
O00629	KPNA4	Importin subunit alpha-3	1.9	0.002	PRDX2
H3BPE1	MACF1	Microtubule-actin cross-linking factor 1, isoforms 1/2/3/5	1.9	0.003	PRDX2
Q8N6R0	EEF1AKNMT	eEF1A lysine and N-terminal methyltransferase	1.9	0.007	PRDX2
P57076	CFAP298	Cilia- and flagella-associated protein 298	1.9	0.010	PRDX2
Q9Y371	SH3GLB1	Endophilin-B1	1.8	0.000	PRDX2
Q8WTT2	NOC3L	Nucleolar complex protein 3 homolog	1.8	0.002	PRDX2
Q92667	AKAP1	A-kinase anchor protein 1, mitochondrial	1.8	0.005	PRDX2
J3QRD1	ALDH3A2	Aldehyde dehydrogenase family 3 member A2	1.8	0.009	PRDX2
Q6ZVM7	TOM1L2	TOM1-like protein 2	1.8	0.011	PRDX2
Q8TB36	GDAP1	Ganglioside-induced differentiation-associated protein 1	1.8	0.013	PRDX2
O60566	BUB1B	Mitotic checkpoint serine/threonine-protein kinase BUB1 beta	1.8	0.024	PRDX2
Q7Z3C6	ATG9A	Autophagy-related protein 9A	1.8	0.033	PRDX2
Q8TCG1	CIP2A	Protein CIP2A	1.7	0.000	PRDX2
Q5T3I0	GPATCH4	G patch domain-containing protein 4	1.7	0.000	PRDX2
Q13451	FKBP5	Peptidyl-prolyl cis-trans isomerase FKBP5	1.7	0.000	PRDX2
O43823	AKAP8	A-kinase anchor protein 8	1.7	0.002	PRDX2
P18031	PTPN1	Tyrosine-protein phosphatase non-receptor type 1	1.7	0.002	PRDX2
F5H008	VPS33B	Vacuolar protein sorting-associated protein 33B	1.7	0.033	PRDX2
P35580	MYH10	Myosin-10	1.6	0.000	PRDX2
P56182	RRP1	Ribosomal RNA processing protein 1 homolog A	1.6	0.000	PRDX2
Q8TC07	TBC1D15	TBC1 domain family member 15	1.6	0.000	PRDX2
Q4J6C6	PREPL	Prolyl endopeptidase-like	1.6	0.000	PRDX2
Q9UBB4	ATXN10	Ataxin-10	1.6	0.000	PRDX2
Q96CS3	FAF2	FAS-associated factor 2	1.6	0.001	PRDX2
K7ESE9	BCAS3	Breast carcinoma-amplified sequence 3	1.6	0.002	PRDX2
E7ESP9	NEFM	160 kDa neurofilament protein	1.6	0.006	PRDX2
Q9BVP2	GNL3	Guanine nucleotide-binding protein-like 3	1.6	0.006	PRDX2
A0A2Q2TH77	A0A2Q2TH77	>tr|A0A2Q2TH77|A0A2Q2TH77_HUMAN Golgin subfamily A member 2 OS=Homo sapiens OX=9606 GN=GOLGA2 PE=1 SV=1;>sp|Q08379|GOGA2_HUMAN Golgin subfamily A member 2 OS=Homo sapiens OX=9606 GN=GOLGA2 PE=1 SV=3;>tr|A0A1W2PQY5|A0A1W2PQY5_HUMAN Golgin subfamily A member	1.6	0.026	PRDX2
P23284	PPIB	Peptidyl-prolyl cis-trans isomerase B	1.6	0.028	PRDX2
Q96T88	UHRF1	E3 ubiquitin-protein ligase UHRF1	1.6	0.029	PRDX2
P48449	LSS	Lanosterol synthase	1.6	0.036	PRDX2
Q8ND04	SMG8	Protein SMG8	1.6	0.038	PRDX2
Q9BZG1	RAB34	Ras-related protein Rab-34	1.6	0.049	PRDX2
P42166	TMPO	Lamina-associated polypeptide 2, isoform alpha	1.5	0.000	PRDX2
Q6WCQ1	MPRIP	Myosin phosphatase Rho-interacting protein	1.5	0.000	PRDX2
O95373	IPO7	Importin-7	1.5	0.001	PRDX2
Q8IY81	FTSJ3	pre-rRNA 2'-O-ribose RNA methyltransferase FTSJ3	1.5	0.001	PRDX2
Q9NY61	AATF	Protein AATF	1.5	0.001	PRDX2
O14579	COPE	Coatomer subunit epsilon	1.5	0.001	PRDX2
O00505	KPNA3	Importin subunit alpha-4	1.5	0.002	PRDX2
Q5VT66	MTARC1	Mitochondrial amidoxime-reducing component 1	1.5	0.002	PRDX2
O60264	SMARCA5	SWI/SNF-related matrix-associated actin-dependent regulator of chromatin subfamily A member 5	1.5	0.007	PRDX2
Q9Y6Y8	SEC23IP	SEC23-interacting protein	1.5	0.009	PRDX2
P98194	ATP2C1	Calcium-transporting ATPase type 2C member 1	1.5	0.014	PRDX2
X6RCK5	DCTN3	Dynactin subunit 3	1.5	0.024	PRDX2
Q14690	PDCD11	Protein RRP5 homolog	1.5	0.032	PRDX2
Q99447	PCYT2	Ethanolamine-phosphate cytidylyltransferase	1.5	0.032	PRDX2
P46013	MKI67	Proliferation marker protein Ki-67	1.5	0.047	PRDX2
Q9Y4W2	LAS1L	Ribosomal biogenesis protein LAS1L	1.5	0.048	PRDX2
Q9BRA2	TXNDC17	Thioredoxin domain-containing protein 17	1.4	0.000	PRDX2
O95163	ELP1	Elongator complex protein 1	1.4	0.000	PRDX2
Q8TC12	RDH11	Retinol dehydrogenase 11	1.4	0.000	PRDX2
Q9Y4E8	USP15	Ubiquitin carboxyl-terminal hydrolase 15	1.4	0.001	PRDX2
Q15785	TOMM34	Mitochondrial import receptor subunit TOM34	1.4	0.002	PRDX2
Q6P1M0	SLC27A4	Long-chain fatty acid transport protein 4	1.4	0.003	PRDX2
O43615	TIMM44	Mitochondrial import inner membrane translocase subunit TIM44	1.4	0.003	PRDX2
Q5UIP0	RIF1	Telomere-associated protein RIF1	1.4	0.003	PRDX2
Q9P0M6	MACROH2A2	Core histone macro-H2A.2	1.4	0.004	PRDX2
P14923	JUP	Junction plakoglobin	1.4	0.004	PRDX2
O94905	ERLIN2	Erlin-2	1.4	0.005	PRDX2
C9J250	RBM6	RNA-binding protein 6	1.4	0.006	PRDX2
Q9NQ29	LUC7L	Putative RNA-binding protein Luc7-like 1	1.4	0.006	PRDX2
Q01804	OTUD4	OTU domain-containing protein 4	1.4	0.010	PRDX2
Q5T3Q7	HEATR1	HEAT repeat-containing protein 1	1.4	0.010	PRDX2
P23588	EIF4B	Eukaryotic translation initiation factor 4B	1.4	0.015	PRDX2
P29372	MPG	DNA-3-methyladenine glycosylase	1.4	0.029	PRDX2
H0YEN2	PPP6R3	Serine/threonine-protein phosphatase 6 regulatory subunit 3	1.4	0.040	PRDX2
Q676U5	ATG16L1	Autophagy-related protein 16-1	1.4	0.048	PRDX2
P46199	MTIF2	Translation initiation factor IF-2, mitochondrial	1.4	0.048	PRDX2
O94874	UFL1	E3 UFM1-protein ligase 1	1.3	0.000	PRDX2
P50542	PEX5	Peroxisomal targeting signal 1 receptor	1.3	0.000	PRDX2
Q86U86	PBRM1	Protein polybromo-1	1.3	0.000	PRDX2
O75165	DNAJC13	DnaJ homolog subfamily C member 13	1.3	0.000	PRDX2
Q9HAV7	GRPEL1	GrpE protein homolog 1, mitochondrial	1.3	0.001	PRDX2
A0A0A0MRM8	MYO6	Unconventional myosin-6	1.3	0.002	PRDX2
E9PKP7	UBTF	Nucleolar transcription factor 1	1.3	0.002	PRDX2
Q9UJC3	HOOK1	Protein Hook homolog 1	1.3	0.002	PRDX2
O15381	NVL	Nuclear valosin-containing protein-like	1.3	0.003	PRDX2
Q15843	NEDD8	NEDD8	1.3	0.004	PRDX2
Q9HB07	MYG1	MYG1 exonuclease	1.3	0.004	PRDX2
Q9Y3T9	NOC2L	Nucleolar complex protein 2 homolog	1.3	0.005	PRDX2
O60684	KPNA6	Importin subunit alpha-7	1.3	0.005	PRDX2
Q9BYN8	MRPS26	28S ribosomal protein S26, mitochondrial	1.3	0.005	PRDX2
Q15650	TRIP4	Activating signal cointegrator 1	1.3	0.006	PRDX2
Q03701	CEBPZ	CCAAT/enhancer-binding protein zeta	1.3	0.007	PRDX2
Q96QK1	VPS35	Vacuolar protein sorting-associated protein 35	1.3	0.007	PRDX2
P07196	NEFL	Neurofilament light polypeptide	1.3	0.010	PRDX2
P50502	ST13	Hsc70-interacting protein	1.3	0.010	PRDX2
Q08945	SSRP1	FACT complex subunit SSRP1	1.3	0.015	PRDX2
Q99426	TBCB	Tubulin-folding cofactor B	1.3	0.015	PRDX2
Q92665	MRPS31	28S ribosomal protein S31, mitochondrial	1.3	0.016	PRDX2
P40616	ARL1	ADP-ribosylation factor-like protein 1	1.3	0.026	PRDX2
Q7Z406	MYH14	Myosin-14	1.3	0.032	PRDX2
Q7Z4H7	HAUS6	HAUS augmin-like complex subunit 6	1.2	0.000	PRDX2
O76031	CLPX	ATP-dependent Clp protease ATP-binding subunit clpX-like, mitochondrial	1.2	0.000	PRDX2
J3QLD9	FLOT2	Flotillin	1.2	0.000	PRDX2
Q96S55	WRNIP1	ATPase WRNIP1	1.2	0.000	PRDX2
Q8NCA5	FAM98A	Protein FAM98A	1.2	0.001	PRDX2
Q9NX63	CHCHD3	MICOS complex subunit MIC19	1.2	0.001	PRDX2
O95613	PCNT	Pericentrin	1.2	0.001	PRDX2
Q9NZB2	FAM120A	Constitutive coactivator of PPAR-gamma-like protein 1	1.2	0.002	PRDX2
O94826	TOMM70	Mitochondrial import receptor subunit TOM70	1.2	0.002	PRDX2
P62314	SNRPD1	Small nuclear ribonucleoprotein Sm D1	1.2	0.002	PRDX2
G5EA36	CDC27	Cell division cycle 27, isoform CRA_c	1.2	0.002	PRDX2
A0A0D9SF70	ARFGAP2	ADP-ribosylation factor GTPase-activating protein 2	1.2	0.003	PRDX2
Q14126	DSG2	Desmoglein-2	1.2	0.004	PRDX2
P58546	MTPN	Myotrophin	1.2	0.004	PRDX2
Q9NSK0	KLC4	Kinesin light chain 4	1.2	0.005	PRDX2
Q12996	CSTF3	Cleavage stimulation factor subunit 3	1.2	0.006	PRDX2
O43707	ACTN4	Alpha-actinin-4	1.2	0.006	PRDX2
O15305	PMM2	Phosphomannomutase 2	1.2	0.006	PRDX2
Q99567	NUP88	Nuclear pore complex protein Nup88	1.2	0.006	PRDX2
Q9Y673	ALG5	Dolichyl-phosphate beta-glucosyltransferase	1.2	0.007	PRDX2
P63092	GNAS	Guanine nucleotide-binding protein G(s) subunit alpha isoforms short	1.2	0.007	PRDX2
Q96TC7	RMDN3	Regulator of microtubule dynamics protein 3	1.2	0.007	PRDX2
P42696	RBM34	RNA-binding protein 34	1.2	0.007	PRDX2
O15226	NKRF	NF-kappa-B-repressing factor	1.2	0.009	PRDX2
P19022	CDH2	Cadherin-2	1.2	0.011	PRDX2
O95376	ARIH2	E3 ubiquitin-protein ligase ARIH2	1.2	0.011	PRDX2
A0A2R8Y566	RELCH	RAB11-binding protein RELCH	1.2	0.011	PRDX2
O00291	HIP1	Huntingtin-interacting protein 1	1.2	0.011	PRDX2
O00170	AIP	AH receptor-interacting protein	1.2	0.011	PRDX2
Q15042	RAB3GAP1	Rab3 GTPase-activating protein catalytic subunit	1.2	0.011	PRDX2
P52701	MSH6	DNA mismatch repair protein Msh6	1.2	0.015	PRDX2
O75146	HIP1R	Huntingtin-interacting protein 1-related protein	1.2	0.019	PRDX2
P20042	EIF2S2	Eukaryotic translation initiation factor 2 subunit 2	1.2	0.023	PRDX2
P07947	YES1	Tyrosine-protein kinase Yes	1.2	0.023	PRDX2
O14828	SCAMP3	Secretory carrier-associated membrane protein 3	1.2	0.025	PRDX2
F8W0J6	NAP1L1	Nucleosome assembly protein 1-like 1	1.2	0.025	PRDX2
Q9BTT0	ANP32E	Acidic leucine-rich nuclear phosphoprotein 32 family member E	1.2	0.026	PRDX2
O14646	CHD1	Chromodomain-helicase-DNA-binding protein 1	1.2	0.027	PRDX2
E9PNW8	FAR1	Fatty acyl-CoA reductase	1.2	0.028	PRDX2
Q9NW13	RBM28	RNA-binding protein 28	1.2	0.032	PRDX2
Q96I51	RCC1L	RCC1-like G exchanging factor-like protein	1.2	0.033	PRDX2
Q71UM5	RPS27L	40S ribosomal protein S27-like	1.2	0.035	PRDX2
O15160	POLR1C	DNA-directed RNA polymerases I and III subunit RPAC1	1.2	0.040	PRDX2
Q9Y3B7	MRPL11	39S ribosomal protein L11, mitochondrial	1.2	0.046	PRDX2
P35251	RFC1	Replication factor C subunit 1	1.1	0.000	PRDX2
O43396	TXNL1	Thioredoxin-like protein 1	1.1	0.000	PRDX2
Q8IYB8	SUPV3L1	ATP-dependent RNA helicase SUPV3L1, mitochondrial	1.1	0.000	PRDX2
Q9UPT5	EXOC7	Exocyst complex component 7	1.1	0.000	PRDX2
B8ZZC5	GLS	Glutaminase	1.1	0.001	PRDX2
P60983	GMFB	Glia maturation factor beta	1.1	0.001	PRDX2
Q14974	KPNB1	Importin subunit beta-1	1.1	0.001	PRDX2
Q9NV70	EXOC1	Exocyst complex component 1	1.1	0.001	PRDX2
Q8N122	RPTOR	Regulatory-associated protein of mTOR	1.1	0.002	PRDX2
O75116	ROCK2	Rho-associated protein kinase 2	1.1	0.002	PRDX2
Q96EY7	PTCD3	Pentatricopeptide repeat domain-containing protein 3, mitochondrial	1.1	0.002	PRDX2
Q9UKG1	APPL1	DCC-interacting protein 13-alpha	1.1	0.002	PRDX2
Q96A33	CCDC47	Coiled-coil domain-containing protein 47	1.1	0.002	PRDX2
Q9H3P7	ACBD3	Golgi resident protein GCP60	1.1	0.003	PRDX2
Q13907	IDI1	Isopentenyl-diphosphate Delta-isomerase 1	1.1	0.004	PRDX2
Q86VS8	HOOK3	Protein Hook homolog 3	1.1	0.005	PRDX2
Q96P70	IPO9	Importin-9	1.1	0.005	PRDX2
Q13085	ACACA	Acetyl-CoA carboxylase 1	1.1	0.006	PRDX2
Q7Z7K6	CENPV	Centromere protein V	1.1	0.006	PRDX2
O00410	IPO5	Importin-5	1.1	0.006	PRDX2
Q5QJE6	DNTTIP2	Deoxynucleotidyltransferase terminal-interacting protein 2	1.1	0.006	PRDX2
P16435	POR	NADPH--cytochrome P450 reductase	1.1	0.007	PRDX2
P42765	ACAA2	3-ketoacyl-CoA thiolase, mitochondrial	1.1	0.009	PRDX2
Q04837	SSBP1	Single-stranded DNA-binding protein, mitochondrial	1.1	0.011	PRDX2
P57740	NUP107	Nuclear pore complex protein Nup107	1.1	0.013	PRDX2
Q6P996	PDXDC1	Pyridoxal-dependent decarboxylase domain-containing protein 1	1.1	0.013	PRDX2
O75691	UTP20	Small subunit processome component 20 homolog	1.1	0.017	PRDX2
Q5T4S7	UBR4	E3 ubiquitin-protein ligase UBR4	1.1	0.018	PRDX2
P16615	ATP2A2	Sarcoplasmic/endoplasmic reticulum calcium ATPase 2	1.1	0.021	PRDX2
D6RB59	EXOC3	Exocyst complex component 3	1.1	0.024	PRDX2
P62879	GNB2	Guanine nucleotide-binding protein G(I)/G(S)/G(T) subunit beta-2	1.1	0.025	PRDX2
Q14318	FKBP8	Peptidyl-prolyl cis-trans isomerase FKBP8	1.1	0.033	PRDX2
P50995	ANXA11	Annexin A11	1.1	0.033	PRDX2
Q9NQT5	EXOSC3	Exosome complex component RRP40	1.1	0.036	PRDX2
Q9H0E2	TOLLIP	Toll-interacting protein	1.1	0.036	PRDX2
Q9H6S0	YTHDC2	3'-5' RNA helicase YTHDC2	1.1	0.041	PRDX2
Q9UI30	TRMT112	Multifunctional methyltransferase subunit TRM112-like protein	1.1	0.042	PRDX2
P62979	RPS27A	Ubiquitin-40S ribosomal protein S27a	1.1	0.047	PRDX2
O43719	HTATSF1	HIV Tat-specific factor 1	1.1	0.048	PRDX2
Q8ND24	RNF214	RING finger protein 214	1.0	0.001	PRDX2
Q6UWP7	LCLAT1	Lysocardiolipin acyltransferase 1	1.0	0.001	PRDX2
Q86UP2	KTN1	Kinectin	1.0	0.002	PRDX2
Q9NY93	DDX56	Probable ATP-dependent RNA helicase DDX56	1.0	0.002	PRDX2
P56537	EIF6	Eukaryotic translation initiation factor 6	1.0	0.002	PRDX2
Q15555	MAPRE2	Microtubule-associated protein RP/EB family member 2	1.0	0.003	PRDX2
P58107	EPPK1	Epiplakin	1.0	0.007	PRDX2
A0A1B0GV47	KIF21A	Kinesin-like protein KIF21A	1.0	0.007	PRDX2
Q13951	CBFB	Core-binding factor subunit beta	1.0	0.011	PRDX2
Q8IWS0	PHF6	PHD finger protein 6	1.0	0.011	PRDX2
P53384	NUBP1	Cytosolic Fe-S cluster assembly factor NUBP1	1.0	0.013	PRDX2
Q93034	CUL5	Cullin-5	1.0	0.014	PRDX2
Q71RC2	LARP4	La-related protein 4	1.0	0.016	PRDX2
Q92990	GLMN	Glomulin	1.0	0.017	PRDX2
O43149	ZZEF1	Zinc finger ZZ-type and EF-hand domain-containing protein 1	1.0	0.018	PRDX2
F8VYE8	PPP1CC	Serine/threonine-protein phosphatase	1.0	0.022	PRDX2
P41567	EIF1	Eukaryotic translation initiation factor 1	1.0	0.033	PRDX2
P08670	VIM	Vimentin	1.0	0.038	PRDX2
Q9BZE4	GTPBP4	Nucleolar GTP-binding protein 1	1.0	0.038	PRDX2
O60502	OGA	Protein O-GlcNAcase	1.0	0.044	PRDX2
P52209	PGD	6-phosphogluconate dehydrogenase, decarboxylating	1.0	0.048	PRDX2
P30048	PRDX3	Thioredoxin-dependent peroxide reductase, mitochondrial	5.4	0.000	PRDX4
P61106	RAB14	Ras-related protein Rab-14	3.1	0.000	PRDX4
O00629	KPNA4	Importin subunit alpha-3	3.1	0.000	PRDX4
Q9Y679	AUP1	Lipid droplet-regulating VLDL assembly factor AUP1	3.1	0.000	PRDX4
Q8N5K1	CISD2	CDGSH iron-sulfur domain-containing protein 2	3.1	0.003	PRDX4
Q07820	MCL1	Induced myeloid leukemia cell differentiation protein Mcl-1	2.8	0.001	PRDX4
P51148	RAB5C	Ras-related protein Rab-5C	2.8	0.004	PRDX4
Q5SZR4	TDRKH	Tudor and KH domain containing, isoform CRA_a	2.8	0.007	PRDX4
P19022	CDH2	Cadherin-2	2.7	0.000	PRDX4
O95373	IPO7	Importin-7	2.7	0.000	PRDX4
O96008	TOMM40	Mitochondrial import receptor subunit TOM40 homolog	2.7	0.005	PRDX4
Q15388	TOMM20	Mitochondrial import receptor subunit TOM20 homolog	2.7	0.007	PRDX4
P30519	HMOX2	Heme oxygenase 2	2.7	0.007	PRDX4
G5EA06	MRPS27	28S ribosomal protein S27, mitochondrial	2.6	0.001	PRDX4
Q9H2P9	DPH5	Diphthine methyl ester synthase	2.6	0.001	PRDX4
P06132	UROD	Uroporphyrinogen decarboxylase	2.6	0.002	PRDX4
Q8N6T3	ARFGAP1	ADP-ribosylation factor GTPase-activating protein 1	2.5	0.000	PRDX4
P98194	ATP2C1	Calcium-transporting ATPase type 2C member 1	2.5	0.000	PRDX4
H7BXI1	ESYT2	Extended synaptotagmin-2	2.4	0.000	PRDX4
Q8TEX9	IPO4	Importin-4	2.4	0.000	PRDX4
Q9BSJ8	ESYT1	Extended synaptotagmin-1	2.4	0.001	PRDX4
Q7Z434	MAVS	Mitochondrial antiviral-signaling protein	2.4	0.006	PRDX4
Q8N4V1	MMGT1	Membrane magnesium transporter 1	2.3	0.001	PRDX4
P42574	CASP3	Caspase-3	2.3	0.003	PRDX4
A0A0A0MS29	MFF	Mitochondrial fission factor	2.3	0.003	PRDX4
A0A2R8YFH5	SEC23B	Protein transport protein SEC23	2.3	0.004	PRDX4
Q7Z3C6	ATG9A	Autophagy-related protein 9A	2.3	0.015	PRDX4
O00505	KPNA3	Importin subunit alpha-4	2.2	0.000	PRDX4
Q9BTE1	DCTN5	Dynactin subunit 5	2.2	0.002	PRDX4
P51809	VAMP7	Vesicle-associated membrane protein 7	2.2	0.011	PRDX4
A0A0A0MR02	VDAC2	Outer mitochondrial membrane protein porin 2	2.1	0.000	PRDX4
Q5VT66	MTARC1	Mitochondrial amidoxime-reducing component 1	2.1	0.000	PRDX4
P50542	PEX5	Peroxisomal targeting signal 1 receptor	2.1	0.000	PRDX4
Q96DH6	MSI2	RNA-binding protein Musashi homolog 2	2.1	0.002	PRDX4
Q92667	AKAP1	A-kinase anchor protein 1, mitochondrial	2.1	0.002	PRDX4
Q13190	STX5	Syntaxin-5	2.1	0.003	PRDX4
P84085	ARF5	ADP-ribosylation factor 5	2.1	0.007	PRDX4
Q9NQG6	MIEF1	Mitochondrial dynamics protein MID51	2.1	0.009	PRDX4
Q96EA4	SPDL1	Protein Spindly	2.1	0.014	PRDX4
P07384	CAPN1	Calpain-1 catalytic subunit	2.0	0.003	PRDX4
E9PHS0	LANCL1	Glutathione S-transferase LANCL1	2.0	0.006	PRDX4
P56962	STX17	Syntaxin-17	2.0	0.009	PRDX4
Q8WVC2	RPS21	40S ribosomal protein S21	2.0	0.009	PRDX4
P49642	PRIM1	DNA primase small subunit	1.9	0.000	PRDX4
Q9H0E2	TOLLIP	Toll-interacting protein	1.9	0.001	PRDX4
P18031	PTPN1	Tyrosine-protein phosphatase non-receptor type 1	1.9	0.001	PRDX4
B3KUS5	USP30	Ubiquitin carboxyl-terminal hydrolase	1.9	0.002	PRDX4
P40616	ARL1	ADP-ribosylation factor-like protein 1	1.9	0.003	PRDX4
Q9BPW8	NIPSNAP1	Protein NipSnap homolog 1	1.9	0.005	PRDX4
Q9NZ32	ACTR10	Actin-related protein 10	1.9	0.009	PRDX4
Q14789	GOLGB1	Golgin subfamily B member 1	1.9	0.010	PRDX4
Q6FI81	CIAPIN1	Anamorsin	1.9	0.013	PRDX4
A0A0A0MR09	PTPN9	Tyrosine-protein phosphatase non-receptor type 9	1.9	0.015	PRDX4
P12236	SLC25A6	ADP/ATP translocase 3	1.8	0.000	PRDX4
P48047	ATP5PO	ATP synthase subunit O, mitochondrial	1.8	0.007	PRDX4
Q6ZVM7	TOM1L2	TOM1-like protein 2	1.8	0.009	PRDX4
P49458	SRP9	Signal recognition particle 9 kDa protein	1.8	0.012	PRDX4
Q9NP72	RAB18	Ras-related protein Rab-18	1.8	0.015	PRDX4
O95163	ELP1	Elongator complex protein 1	1.7	0.000	PRDX4
Q14318	FKBP8	Peptidyl-prolyl cis-trans isomerase FKBP8	1.7	0.001	PRDX4
P31689	DNAJA1	DnaJ homolog subfamily A member 1	1.7	0.004	PRDX4
Q9NS69	TOMM22	Mitochondrial import receptor subunit TOM22 homolog	1.7	0.007	PRDX4
O14828	SCAMP3	Secretory carrier-associated membrane protein 3	1.7	0.008	PRDX4
U3KQC1	WDR18	WD repeat-containing protein 18	1.7	0.008	PRDX4
A0A3B3ITJ4	HNRNPL	Heterogeneous nuclear ribonucleoprotein L	1.7	0.010	PRDX4
P42025	ACTR1B	Beta-centractin	1.7	0.011	PRDX4
O00291	HIP1	Huntingtin-interacting protein 1	1.7	0.012	PRDX4
Q8NI60	COQ8A	Atypical kinase COQ8A, mitochondrial	1.7	0.014	PRDX4
Q16186	ADRM1	Proteasomal ubiquitin receptor ADRM1	1.7	0.014	PRDX4
Q9Y4P1	ATG4B	Cysteine protease ATG4B	1.7	0.014	PRDX4
Q9NP97	DYNLRB1	Dynein light chain roadblock-type 1	1.7	0.015	PRDX4
P30740	SERPINB1	Leukocyte elastase inhibitor	1.7	0.017	PRDX4
Q5JRI1	SRSF10	Serine/arginine-rich-splicing factor 10	1.7	0.021	PRDX4
O60566	BUB1B	Mitotic checkpoint serine/threonine-protein kinase BUB1 beta	1.7	0.023	PRDX4
Q7L5D6	GET4	Golgi to ER traffic protein 4 homolog	1.7	0.027	PRDX4
Q9NVJ2	ARL8B	ADP-ribosylation factor-like protein 8B	1.7	0.027	PRDX4
P48449	LSS	Lanosterol synthase	1.7	0.032	PRDX4
P08243	ASNS	Asparagine synthetase [glutamine-hydrolyzing]	1.6	0.000	PRDX4
P16435	POR	NADPH--cytochrome P450 reductase	1.6	0.000	PRDX4
Q8TC07	TBC1D15	TBC1 domain family member 15	1.6	0.000	PRDX4
Q9UBB4	ATXN10	Ataxin-10	1.6	0.000	PRDX4
P21964	COMT	Catechol O-methyltransferase	1.6	0.001	PRDX4
Q8TCG1	CIP2A	Protein CIP2A	1.6	0.001	PRDX4
O14964	HGS	Hepatocyte growth factor-regulated tyrosine kinase substrate	1.6	0.002	PRDX4
P04843	RPN1	Dolichyl-diphosphooligosaccharide--protein glycosyltransferase subunit 1	1.6	0.004	PRDX4
Q53H96	PYCR3	Pyrroline-5-carboxylate reductase 3	1.6	0.012	PRDX4
Q95295	SNAPIN	SNARE-associated protein Snapin	1.6	0.012	PRDX4

O95295	SNAPIN	SNARE-associated protein Snapin	1.6	0.012	PRDX4
H0YLH3	RABGGTA	Geranylgeranyl transferase type-2 subunit alpha	1.6	0.012	PRDX4
Q8NF37	LPCAT1	Lysophosphatidylcholine acyltransferase 1	1.6	0.026	PRDX4
Q9H840	GEMIN7	Gem-associated protein 7	1.6	0.033	PRDX4
Q8N573	OXR1	Oxidation resistance protein 1	1.6	0.043	PRDX4
Q9Y371	SH3GLB1	Endophilin-B1	1.5	0.000	PRDX4
P35232	PHB	Prohibitin	1.5	0.000	PRDX4
Q9Y262	EIF3L	Eukaryotic translation initiation factor 3 subunit L	1.5	0.010	PRDX4
Q9Y6Y8	SEC23IP	SEC23-interacting protein	1.5	0.013	PRDX4
Q6DKI1	RPL7L1	60S ribosomal protein L7-like 1	1.5	0.015	PRDX4
Q9BZE1	MRPL37	39S ribosomal protein L37, mitochondrial	1.5	0.019	PRDX4
Q86W42	THOC6	THO complex subunit 6 homolog	1.5	0.027	PRDX4
Q9Y580	RBM7	RNA-binding protein 7	1.5	0.031	PRDX4
Q9NUY8	TBC1D23	TBC1 domain family member 23	1.5	0.037	PRDX4
P54646	PRKAA2	5'-AMP-activated protein kinase catalytic subunit alpha-2	1.5	0.038	PRDX4
O95573	ACSL3	Long-chain-fatty-acid--CoA ligase 3	1.4	0.000	PRDX4
Q9UKG1	APPL1	DCC-interacting protein 13-alpha	1.4	0.000	PRDX4
O95292	VAPB	Vesicle-associated membrane protein-associated protein B/C	1.4	0.000	PRDX4
P35573	AGL	Glycogen debranching enzyme	1.4	0.001	PRDX4
Q9Y4E8	USP15	Ubiquitin carboxyl-terminal hydrolase 15	1.4	0.001	PRDX4
Q6P1M0	SLC27A4	Long-chain fatty acid transport protein 4	1.4	0.002	PRDX4
A0A0C4DGQ6	RPRD1A	Regulation of nuclear pre-mRNA domain-containing protein 1A	1.4	0.002	PRDX4
P14923	JUP	Junction plakoglobin	1.4	0.004	PRDX4
Q96CS3	FAF2	FAS-associated factor 2	1.4	0.004	PRDX4
P35237	SERPINB6	Serpin B6	1.4	0.004	PRDX4
Q53EL6	PDCD4	Programmed cell death protein 4	1.4	0.005	PRDX4
P42771	CDKN2A	Cyclin-dependent kinase inhibitor 2A	1.4	0.005	PRDX4
A0A3B3IUD2	MSTO1	Protein misato homolog 1	1.4	0.008	PRDX4
Q9BTT0	ANP32E	Acidic leucine-rich nuclear phosphoprotein 32 family member E	1.4	0.008	PRDX4
A0A0D9SF70	ARFGAP2	ADP-ribosylation factor GTPase-activating protein 2	1.4	0.009	PRDX4
P63167	DYNLL1	Dynein light chain 1, cytoplasmic	1.4	0.021	PRDX4
Q5QNY5	PEX19	Peroxin-19	1.4	0.027	PRDX4
P41250	GARS1	Glycine--tRNA ligase	1.4	0.030	PRDX4
A0A087X2D5	MRPL45	39S ribosomal protein L45, mitochondrial	1.4	0.039	PRDX4
Q6PGP7	TTC37	Tetratricopeptide repeat protein 37	1.4	0.044	PRDX4
Q9H974	QTRT2	Queuine tRNA-ribosyltransferase accessory subunit 2	1.4	0.045	PRDX4
Q9Y673	ALG5	Dolichyl-phosphate beta-glucosyltransferase	1.4	0.047	PRDX4
G5EA36	CDC27	Cell division cycle 27, isoform CRA_c	1.3	0.000	PRDX4
Q4J6C6	PREPL	Prolyl endopeptidase-like	1.3	0.000	PRDX4
E7ETZ4	BZW2	Basic leucine zipper and W2 domain-containing protein 2	1.3	0.001	PRDX4
Q5T3I0	GPATCH4	G patch domain-containing protein 4	1.3	0.001	PRDX4
D6R938	CAMK2D	Calcium/calmodulin-dependent protein kinase	1.3	0.002	PRDX4
P31930	UQCRC1	Cytochrome b-c1 complex subunit 1, mitochondrial	1.3	0.003	PRDX4
P34897	SHMT2	Serine hydroxymethyltransferase, mitochondrial	1.3	0.003	PRDX4
P51452	DUSP3	Dual specificity protein phosphatase 3	1.3	0.003	PRDX4
K7ESE9	BCAS3	Breast carcinoma-amplified sequence 3	1.3	0.003	PRDX4
A0A2Q2TH77	A0A2Q2TH77	>tr|A0A2Q2TH77|A0A2Q2TH77_HUMAN Golgin subfamily A member 2 OS=Homo sapiens OX=9606 GN=GOLGA2 PE=1 SV=1;>sp|Q08379|GOGA2_HUMAN Golgin subfamily A member 2 OS=Homo sapiens OX=9606 GN=GOLGA2 PE=1 SV=3;>tr|A0A1W2PQY5|A0A1W2PQY5_HUMAN Golgin subfamily A member	1.3	0.004	PRDX4
Q15276	RABEP1	Rab GTPase-binding effector protein 1	1.3	0.004	PRDX4
Q6UWP7	LCLAT1	Lysocardiolipin acyltransferase 1	1.3	0.004	PRDX4
O60678	PRMT3	Protein arginine N-methyltransferase 3	1.3	0.004	PRDX4
Q9NQG5	RPRD1B	Regulation of nuclear pre-mRNA domain-containing protein 1B	1.3	0.010	PRDX4
F8W0J6	NAP1L1	Nucleosome assembly protein 1-like 1	1.3	0.014	PRDX4
P19367	HK1	Hexokinase-1	1.3	0.018	PRDX4
Q9Y394	DHRS7	Dehydrogenase/reductase SDR family member 7	1.3	0.022	PRDX4
F5H008	VPS33B	Vacuolar protein sorting-associated protein 33B	1.3	0.028	PRDX4
P35606	COPB2	Coatomer subunit beta'	1.3	0.028	PRDX4
P25705	ATP5F1A	ATP synthase subunit alpha, mitochondrial	1.2	0.000	PRDX4
P04899	GNAI2	Guanine nucleotide-binding protein G(i) subunit alpha-2	1.2	0.000	PRDX4
P36542	ATP5F1C	ATP synthase subunit gamma, mitochondrial	1.2	0.000	PRDX4
P68104	EEF1A1	Elongation factor 1-alpha 1	1.2	0.001	PRDX4
Q96A33	CCDC47	Coiled-coil domain-containing protein 47	1.2	0.001	PRDX4
Q9BVG4	PBDC1	Protein PBDC1	1.2	0.002	PRDX4
O95168	NDUFB4	NADH dehydrogenase [ubiquinone] 1 beta subcomplex subunit 4	1.2	0.002	PRDX4
Q8IXI1	RHOT2	Mitochondrial Rho GTPase 2	1.2	0.002	PRDX4
P31040	SDHA	Succinate dehydrogenase [ubiquinone] flavoprotein subunit, mitochondrial	1.2	0.002	PRDX4
Q9UNM6	PSMD13	26S proteasome non-ATPase regulatory subunit 13	1.2	0.003	PRDX4
Q92769	HDAC2	Histone deacetylase 2	1.2	0.003	PRDX4
O14579	COPE	Coatomer subunit epsilon	1.2	0.004	PRDX4
Q14126	DSG2	Desmoglein-2	1.2	0.006	PRDX4
E9PKG1	PRMT1	Protein arginine N-methyltransferase 1	1.2	0.007	PRDX4
Q5T760	SRSF11	Serine/arginine-rich-splicing factor 11	1.2	0.009	PRDX4
Q9BT25	HAUS8	HAUS augmin-like complex subunit 8	1.2	0.014	PRDX4
P63244	RACK1	Receptor of activated protein C kinase 1	1.2	0.016	PRDX4
Q13464	ROCK1	Rho-associated protein kinase 1	1.2	0.021	PRDX4
P63000	RAC1	Ras-related C3 botulinum toxin substrate 1	1.2	0.022	PRDX4
D6REA0	GATB	Glutamyl-tRNA(Gln) amidotransferase subunit B, mitochondrial	1.2	0.039	PRDX4
O75396	SEC22B	Vesicle-trafficking protein SEC22b	1.1	0.001	PRDX4
P28288	ABCD3	ATP-binding cassette sub-family D member 3	1.1	0.002	PRDX4
C9J8Q5	ALDH5A1	Succinate-semialdehyde dehydrogenase	1.1	0.002	PRDX4
O15397	IPO8	Importin-8	1.1	0.002	PRDX4
P56545	CTBP2	C-terminal-binding protein 2	1.1	0.002	PRDX4
Q9HAV7	GRPEL1	GrpE protein homolog 1, mitochondrial	1.1	0.002	PRDX4
Q8N122	RPTOR	Regulatory-associated protein of mTOR	1.1	0.003	PRDX4
Q7Z4Q2	HEATR3	HEAT repeat-containing protein 3	1.1	0.003	PRDX4
P23526	AHCY	Adenosylhomocysteinase	1.1	0.003	PRDX4
Q9BRA2	TXNDC17	Thioredoxin domain-containing protein 17	1.1	0.004	PRDX4
Q96P70	IPO9	Importin-9	1.1	0.004	PRDX4
Q96TC7	RMDN3	Regulator of microtubule dynamics protein 3	1.1	0.006	PRDX4
Q8WUM0	NUP133	Nuclear pore complex protein Nup133	1.1	0.008	PRDX4
P63092	GNAS	Guanine nucleotide-binding protein G(s) subunit alpha isoforms short	1.1	0.010	PRDX4
Q15738	NSDHL	Sterol-4-alpha-carboxylate 3-dehydrogenase, decarboxylating	1.1	0.015	PRDX4
Q9HAV4	XPO5	Exportin-5	1.1	0.024	PRDX4
P16615	ATP2A2	Sarcoplasmic/endoplasmic reticulum calcium ATPase 2	1.1	0.024	PRDX4
Q16352	INA	Alpha-internexin	1.1	0.025	PRDX4
Q15075	EEA1	Early endosome antigen 1	1.1	0.030	PRDX4
Q9Y6I9	TEX264	Testis-expressed protein 264	1.1	0.033	PRDX4
Q9H0U4	RAB1B	Ras-related protein Rab-1B	1.1	0.036	PRDX4
P62879	GNB2	Guanine nucleotide-binding protein G(I)/G(S)/G(T) subunit beta-2	1.1	0.036	PRDX4
P53677	AP3M2	AP-3 complex subunit mu-2	1.1	0.037	PRDX4
Q9UI30	TRMT112	Multifunctional methyltransferase subunit TRM112-like protein	1.1	0.040	PRDX4
O60306	AQR	RNA helicase aquarius	1.1	0.044	PRDX4
P47755	CAPZA2	F-actin-capping protein subunit alpha-2	1.1	0.047	PRDX4
Q14C86	GAPVD1	GTPase-activating protein and VPS9 domain-containing protein 1	1.1	0.049	PRDX4
P30520	ADSS2	Adenylosuccinate synthetase isozyme 2	1.1	0.049	PRDX4
P19525	EIF2AK2	Interferon-induced, double-stranded RNA-activated protein kinase	1.0	0.001	PRDX4
Q92973	TNPO1	Transportin-1	1.0	0.002	PRDX4
P60983	GMFB	Glia maturation factor beta	1.0	0.003	PRDX4
Q96EY7	PTCD3	Pentatricopeptide repeat domain-containing protein 3, mitochondrial	1.0	0.003	PRDX4
Q6P996	PDXDC1	Pyridoxal-dependent decarboxylase domain-containing protein 1	1.0	0.003	PRDX4
P41240	CSK	Tyrosine-protein kinase CSK	1.0	0.003	PRDX4
Q8NCA5	FAM98A	Protein FAM98A	1.0	0.004	PRDX4
H3BTA2	PPP4C	Serine/threonine-protein phosphatase	1.0	0.006	PRDX4
Q13951	CBFB	Core-binding factor subunit beta	1.0	0.012	PRDX4
Q16718	NDUFA5	NADH dehydrogenase [ubiquinone] 1 alpha subcomplex subunit 5	1.0	0.012	PRDX4
O43765	SGTA	Small glutamine-rich tetratricopeptide repeat-containing protein alpha	1.0	0.013	PRDX4
E9PKP7	UBTF	Nucleolar transcription factor 1	1.0	0.021	PRDX4
Q9UJS0	SLC25A13	Calcium-binding mitochondrial carrier protein Aralar2	1.0	0.025	PRDX4
O15144	ARPC2	Actin-related protein 2/3 complex subunit 2	1.0	0.030	PRDX4
P61353	RPL27	60S ribosomal protein L27	1.0	0.031	PRDX4
Q03701	CEBPZ	CCAAT/enhancer-binding protein zeta	1.0	0.035	PRDX4
O60684	KPNA6	Importin subunit alpha-7	1.0	0.035	PRDX4
P15374	UCHL3	Ubiquitin carboxyl-terminal hydrolase isozyme L3	1.0	0.036	PRDX4
O95373	IPO7	Importin-7	3.5	0.000	PRDX5
O00629	KPNA4	Importin subunit alpha-3	3.3	0.000	PRDX5
Q9Y224	RTRAF	RNA transcription, translation and transport factor protein	3.2	0.000	PRDX5
Q96AC1	FERMT2	Fermitin family homolog 2	3.0	0.000	PRDX5
Q15149	PLEC	Plectin	2.9	0.001	PRDX5
Q8TCG1	CIP2A	Protein CIP2A	2.8	0.000	PRDX5
E9PHS0	LANCL1	Glutathione S-transferase LANCL1	2.7	0.000	PRDX5
P61106	RAB14	Ras-related protein Rab-14	2.7	0.000	PRDX5
Q07820	MCL1	Induced myeloid leukemia cell differentiation protein Mcl-1	2.7	0.000	PRDX5
Q8N2K0	ABHD12	Lysophosphatidylserine lipase ABHD12	2.7	0.002	PRDX5
P30519	HMOX2	Heme oxygenase 2	2.6	0.005	PRDX5
P19105	MYL12A	Myosin regulatory light chain 12A	2.5	0.000	PRDX5
P19022	CDH2	Cadherin-2	2.5	0.000	PRDX5
P06132	UROD	Uroporphyrinogen decarboxylase	2.5	0.001	PRDX5
Q8TEX9	IPO4	Importin-4	2.4	0.000	PRDX5
H3BPE1	MACF1	Microtubule-actin cross-linking factor 1, isoforms 1/2/3/5	2.3	0.000	PRDX5
Q7Z406	MYH14	Myosin-14	2.3	0.000	PRDX5
Q13464	ROCK1	Rho-associated protein kinase 1	2.3	0.000	PRDX5
B3KUS5	USP30	Ubiquitin carboxyl-terminal hydrolase	2.2	0.000	PRDX5
Q9Y679	AUP1	Lipid droplet-regulating VLDL assembly factor AUP1	2.2	0.000	PRDX5
O00505	KPNA3	Importin subunit alpha-4	2.2	0.000	PRDX5
P62879	GNB2	Guanine nucleotide-binding protein G(I)/G(S)/G(T) subunit beta-2	2.2	0.000	PRDX5
Q16352	INA	Alpha-internexin	2.2	0.000	PRDX5
Q8N4V1	MMGT1	Membrane magnesium transporter 1	2.2	0.001	PRDX5
Q15477	SKIV2L	Helicase SKI2W	2.2	0.001	PRDX5
P98194	ATP2C1	Calcium-transporting ATPase type 2C member 1	2.2	0.001	PRDX5
Q96DH6	MSI2	RNA-binding protein Musashi homolog 2	2.2	0.001	PRDX5
P55084	HADHB	Trifunctional enzyme subunit beta, mitochondrial	2.2	0.004	PRDX5
Q7Z434	MAVS	Mitochondrial antiviral-signaling protein	2.2	0.004	PRDX5
Q9BVP2	GNL3	Guanine nucleotide-binding protein-like 3	2.1	0.000	PRDX5
P40616	ARL1	ADP-ribosylation factor-like protein 1	2.1	0.000	PRDX5
Q9BTT0	ANP32E	Acidic leucine-rich nuclear phosphoprotein 32 family member E	2.1	0.000	PRDX5
O95861	BPNT1	3'(2'),5'-bisphosphate nucleotidase 1	2.1	0.000	PRDX5
Q9UBB4	ATXN10	Ataxin-10	2.1	0.000	PRDX5
P63167	DYNLL1	Dynein light chain 1, cytoplasmic	2.1	0.001	PRDX5
A0A0A0MR09	PTPN9	Tyrosine-protein phosphatase non-receptor type 9	2.1	0.001	PRDX5
Q9BTE1	DCTN5	Dynactin subunit 5	2.1	0.002	PRDX5
A0A0A0MS29	MFF	Mitochondrial fission factor	2.1	0.003	PRDX5
P51809	VAMP7	Vesicle-associated membrane protein 7	2.1	0.009	PRDX5
Q5T3Q7	HEATR1	HEAT repeat-containing protein 1	2.0	0.000	PRDX5
Q13085	ACACA	Acetyl-CoA carboxylase 1	2.0	0.000	PRDX5
G5EA31	SEC24C	Protein transport protein Sec24C	2.0	0.000	PRDX5
Q9NY93	DDX56	Probable ATP-dependent RNA helicase DDX56	2.0	0.000	PRDX5
O95163	ELP1	Elongator complex protein 1	2.0	0.000	PRDX5
Q6P2E9	EDC4	Enhancer of mRNA-decapping protein 4	2.0	0.001	PRDX5
Q9BZE1	MRPL37	39S ribosomal protein L37, mitochondrial	2.0	0.002	PRDX5
Q5VW32	BROX	BRO1 domain-containing protein BROX	2.0	0.003	PRDX5
H0YMZ1	PSMA4	Proteasome subunit alpha type	2.0	0.004	PRDX5
Q8IZ07	ANKRD13A	Ankyrin repeat domain-containing protein 13A	2.0	0.025	PRDX5
Q15388	TOMM20	Mitochondrial import receptor subunit TOM20 homolog	2.0	0.025	PRDX5
P30048	PRDX3	Thioredoxin-dependent peroxide reductase, mitochondrial	11.1	0.000	PRDX5
Q9P0M6	MACROH2A2	Core histone macro-H2A.2	1.9	0.000	PRDX5
P08670	VIM	Vimentin	1.9	0.000	PRDX5
O94925	GLS	Glutaminase kidney isoform, mitochondrial	1.9	0.000	PRDX5
Q9HAV4	XPO5	Exportin-5	1.9	0.000	PRDX5
P35580	MYH10	Myosin-10	1.9	0.000	PRDX5
E9PKP7	UBTF	Nucleolar transcription factor 1	1.9	0.000	PRDX5
O14579	COPE	Coatomer subunit epsilon	1.9	0.000	PRDX5
P35606	COPB2	Coatomer subunit beta'	1.9	0.001	PRDX5
P51570	GALK1	Galactokinase	1.9	0.001	PRDX5
P42345	MTOR	Serine/threonine-protein kinase mTOR	1.9	0.003	PRDX5
Q16762	TST	Thiosulfate sulfurtransferase	1.9	0.003	PRDX5
P46940	IQGAP1	Ras GTPase-activating-like protein IQGAP1	1.9	0.004	PRDX5
O60678	PRMT3	Protein arginine N-methyltransferase 3	1.9	0.004	PRDX5
P11388	TOP2A	DNA topoisomerase 2-alpha	1.9	0.005	PRDX5
Q14690	PDCD11	Protein RRP5 homolog	1.9	0.005	PRDX5
Q86WA6	BPHL	Valacyclovir hydrolase	1.9	0.006	PRDX5
Q6DKI1	RPL7L1	60S ribosomal protein L7-like 1	1.9	0.006	PRDX5
P84085	ARF5	ADP-ribosylation factor 5	1.9	0.007	PRDX5
P46013	MKI67	Proliferation marker protein Ki-67	1.9	0.008	PRDX5
Q9NQG6	MIEF1	Mitochondrial dynamics protein MID51	1.9	0.009	PRDX5
P56962	STX17	Syntaxin-17	1.9	0.009	PRDX5
Q7Z3C6	ATG9A	Autophagy-related protein 9A	1.9	0.014	PRDX5
Q5VT66	MTARC1	Mitochondrial amidoxime-reducing component 1	1.8	0.000	PRDX5
O94905	ERLIN2	Erlin-2	1.8	0.000	PRDX5
P08240	SRPRA	Signal recognition particle receptor subunit alpha	1.8	0.000	PRDX5
O95168	NDUFB4	NADH dehydrogenase [ubiquinone] 1 beta subcomplex subunit 4	1.8	0.000	PRDX5
Q6WCQ1	MPRIP	Myosin phosphatase Rho-interacting protein	1.8	0.000	PRDX5
Q9NW13	RBM28	RNA-binding protein 28	1.8	0.001	PRDX5
Q02880	TOP2B	DNA topoisomerase 2-beta	1.8	0.002	PRDX5
Q96RP9	GFM1	Elongation factor G, mitochondrial	1.8	0.002	PRDX5
Q9Y6Y8	SEC23IP	SEC23-interacting protein	1.8	0.002	PRDX5
K7ESE9	BCAS3	Breast carcinoma-amplified sequence 3	1.8	0.002	PRDX5
E7EVH7	E7EVH7	Kinesin light chain	1.8	0.003	PRDX5
Q4VCS5	AMOT	Angiomotin	1.8	0.003	PRDX5
P07384	CAPN1	Calpain-1 catalytic subunit	1.8	0.004	PRDX5
Q13951	CBFB	Core-binding factor subunit beta	1.8	0.005	PRDX5
Q9BPW8	NIPSNAP1	Protein NipSnap homolog 1	1.8	0.006	PRDX5
P42704	LRPPRC	Leucine-rich PPR motif-containing protein, mitochondrial	1.8	0.006	PRDX5
G5EA06	MRPS27	28S ribosomal protein S27, mitochondrial	1.8	0.006	PRDX5
O60502	OGA	Protein O-GlcNAcase	1.8	0.009	PRDX5
Q8WVC2	RPS21	40S ribosomal protein S21	1.8	0.009	PRDX5
Q14789	GOLGB1	Golgin subfamily B member 1	1.8	0.010	PRDX5
P68133	ACTA1	Actin, alpha skeletal muscle	1.8	0.019	PRDX5
O96008	TOMM40	Mitochondrial import receptor subunit TOM40 homolog	1.8	0.033	PRDX5
Q9BYN0	SRXN1	Sulfiredoxin-1	1.8	0.038	PRDX5
Q15075	EEA1	Early endosome antigen 1	1.7	0.000	PRDX5
O94973	AP2A2	AP-2 complex subunit alpha-2	1.7	0.000	PRDX5
Q9GZT3	SLIRP	SRA stem-loop-interacting RNA-binding protein, mitochondrial	1.7	0.000	PRDX5
O15381	NVL	Nuclear valosin-containing protein-like	1.7	0.000	PRDX5
Q6P996	PDXDC1	Pyridoxal-dependent decarboxylase domain-containing protein 1	1.7	0.000	PRDX5
P58107	EPPK1	Epiplakin	1.7	0.000	PRDX5
P04899	GNAI2	Guanine nucleotide-binding protein G(i) subunit alpha-2	1.7	0.000	PRDX5
P56545	CTBP2	C-terminal-binding protein 2	1.7	0.000	PRDX5
P50995	ANXA11	Annexin A11	1.7	0.001	PRDX5
P53597	SUCLG1	Succinate--CoA ligase [ADP/GDP-forming] subunit alpha, mitochondrial	1.7	0.001	PRDX5
P18031	PTPN1	Tyrosine-protein phosphatase non-receptor type 1	1.7	0.001	PRDX5
Q8WTT2	NOC3L	Nucleolar complex protein 3 homolog	1.7	0.003	PRDX5
P09110	ACAA1	3-ketoacyl-CoA thiolase, peroxisomal	1.7	0.005	PRDX5
P48047	ATP5PO	ATP synthase subunit O, mitochondrial	1.7	0.006	PRDX5
Q53H96	PYCR3	Pyrroline-5-carboxylate reductase 3	1.7	0.007	PRDX5
C9JZR2	CTNND1	Catenin delta-1	1.7	0.008	PRDX5
P78316	NOP14	Nucleolar protein 14	1.7	0.008	PRDX5
Q6ZVM7	TOM1L2	TOM1-like protein 2	1.7	0.010	PRDX5
Q9NP72	RAB18	Ras-related protein Rab-18	1.7	0.015	PRDX5
Q9H6R0	DHX33	ATP-dependent RNA helicase DHX33	1.7	0.015	PRDX5
P15924	DSP	Desmoplakin	1.7	0.016	PRDX5
Q9NZ45	CISD1	CDGSH iron-sulfur domain-containing protein 1	1.7	0.040	PRDX5
Q8N5K1	CISD2	CDGSH iron-sulfur domain-containing protein 2	1.7	0.047	PRDX5
P52948	NUP98	Nuclear pore complex protein Nup98-Nup96	1.6	0.000	PRDX5
Q5UIP0	RIF1	Telomere-associated protein RIF1	1.6	0.000	PRDX5
Q5T3I0	GPATCH4	G patch domain-containing protein 4	1.6	0.000	PRDX5
Q86U42	PABPN1	Polyadenylate-binding protein 2	1.6	0.000	PRDX5
O75439	PMPCB	Mitochondrial-processing peptidase subunit beta	1.6	0.000	PRDX5
P43897	TSFM	Elongation factor Ts, mitochondrial	1.6	0.000	PRDX5
A0A0D9SF70	ARFGAP2	ADP-ribosylation factor GTPase-activating protein 2	1.6	0.000	PRDX5
B8ZZC5	GLS	Glutaminase	1.6	0.000	PRDX5
O00410	IPO5	Importin-5	1.6	0.000	PRDX5
Q9Y2R9	MRPS7	28S ribosomal protein S7, mitochondrial	1.6	0.000	PRDX5
P16435	POR	NADPH--cytochrome P450 reductase	1.6	0.000	PRDX5
E9PFR3	PPP2R5D	Serine/threonine-protein phosphatase 2A 56 kDa regulatory subunit	1.6	0.000	PRDX5
O95573	ACSL3	Long-chain-fatty-acid--CoA ligase 3	1.6	0.000	PRDX5
Q8TC07	TBC1D15	TBC1 domain family member 15	1.6	0.000	PRDX5
Q9NQ88	TIGAR	Fructose-2,6-bisphosphatase TIGAR	1.6	0.000	PRDX5
A0A087X1A5	STAU1	Double-stranded RNA-binding protein Staufen homolog 1	1.6	0.000	PRDX5
D6RB59	EXOC3	Exocyst complex component 3	1.6	0.001	PRDX5
E9PF10	NUP155	Nuclear pore complex protein Nup155	1.6	0.001	PRDX5
Q03701	CEBPZ	CCAAT/enhancer-binding protein zeta	1.6	0.001	PRDX5
P63244	RACK1	Receptor of activated protein C kinase 1	1.6	0.001	PRDX5
O43615	TIMM44	Mitochondrial import inner membrane translocase subunit TIM44	1.6	0.001	PRDX5
O75691	UTP20	Small subunit processome component 20 homolog	1.6	0.001	PRDX5
P14923	JUP	Junction plakoglobin	1.6	0.001	PRDX5
P61353	RPL27	60S ribosomal protein L27	1.6	0.002	PRDX5
F5H008	VPS33B	Vacuolar protein sorting-associated protein 33B	1.6	0.003	PRDX5
Q9BW92	TARS2	Threonine--tRNA ligase, mitochondrial	1.6	0.003	PRDX5
Q9H3U1	UNC45A	Protein unc-45 homolog A	1.6	0.003	PRDX5
Q9NTJ5	SACM1L	Phosphatidylinositol-3-phosphatase SAC1	1.6	0.003	PRDX5
Q9UHI6	DDX20	Probable ATP-dependent RNA helicase DDX20	1.6	0.004	PRDX5
Q7L2E3	DHX30	ATP-dependent RNA helicase DHX30	1.6	0.007	PRDX5
Q5VYK3	ECPAS	Proteasome adapter and scaffold protein ECM29	1.6	0.008	PRDX5
Q9Y2R4	DDX52	Probable ATP-dependent RNA helicase DDX52	1.6	0.008	PRDX5
P53007	SLC25A1	Tricarboxylate transport protein, mitochondrial	1.6	0.010	PRDX5
P08559	PDHA1	Pyruvate dehydrogenase E1 component subunit alpha, somatic form, mitochondrial	1.6	0.012	PRDX5
Q8TB36	GDAP1	Ganglioside-induced differentiation-associated protein 1	1.6	0.013	PRDX5
Q01082	SPTBN1	Spectrin beta chain, non-erythrocytic 1	1.6	0.016	PRDX5
Q16186	ADRM1	Proteasomal ubiquitin receptor ADRM1	1.6	0.016	PRDX5
Q9Y4P1	ATG4B	Cysteine protease ATG4B	1.6	0.016	PRDX5
Q13813	SPTAN1	Spectrin alpha chain, non-erythrocytic 1	1.6	0.021	PRDX5
Q5JRI1	SRSF10	Serine/arginine-rich-splicing factor 10	1.6	0.022	PRDX5
O60566	BUB1B	Mitotic checkpoint serine/threonine-protein kinase BUB1 beta	1.6	0.024	PRDX5
K7ELV2	SEH1L	Nucleoporin SEH1	1.6	0.025	PRDX5
H3BRY6	INTS14	Integrator complex subunit 14	1.6	0.027	PRDX5
P42765	ACAA2	3-ketoacyl-CoA thiolase, mitochondrial	1.5	0.000	PRDX5
P08243	ASNS	Asparagine synthetase [glutamine-hydrolyzing]	1.5	0.000	PRDX5
Q9UN37	VPS4A	Vacuolar protein sorting-associated protein 4A	1.5	0.000	PRDX5
A0A0A0MRM8	MYO6	Unconventional myosin-6	1.5	0.000	PRDX5
Q9Y2Q3	GSTK1	Glutathione S-transferase kappa 1	1.5	0.000	PRDX5
P49327	FASN	Fatty acid synthase	1.5	0.000	PRDX5
Q92973	TNPO1	Transportin-1	1.5	0.000	PRDX5
P19525	EIF2AK2	Interferon-induced, double-stranded RNA-activated protein kinase	1.5	0.000	PRDX5
Q9Y371	SH3GLB1	Endophilin-B1	1.5	0.001	PRDX5
O15226	NKRF	NF-kappa-B-repressing factor	1.5	0.001	PRDX5
Q9Y4E8	USP15	Ubiquitin carboxyl-terminal hydrolase 15	1.5	0.003	PRDX5
P04181	OAT	Ornithine aminotransferase, mitochondrial	1.5	0.003	PRDX5
Q13363	CTBP1	C-terminal-binding protein 1	1.5	0.004	PRDX5
O60264	SMARCA5	SWI/SNF-related matrix-associated actin-dependent regulator of chromatin subfamily A member 5	1.5	0.004	PRDX5
P82930	MRPS34	28S ribosomal protein S34, mitochondrial	1.5	0.005	PRDX5
Q92616	GCN1	eIF-2-alpha kinase activator GCN1	1.5	0.006	PRDX5
Q9Y394	DHRS7	Dehydrogenase/reductase SDR family member 7	1.5	0.008	PRDX5
A0A3B3IUD2	MSTO1	Protein misato homolog 1	1.5	0.010	PRDX5
Q9UHQ9	CYB5R1	NADH-cytochrome b5 reductase 1	1.5	0.012	PRDX5
P07947	YES1	Tyrosine-protein kinase Yes	1.5	0.013	PRDX5
P42694	HELZ	Probable helicase with zinc finger domain	1.5	0.013	PRDX5
H0YLH3	RABGGTA	Geranylgeranyl transferase type-2 subunit alpha	1.5	0.014	PRDX5
Q13561	DCTN2	Dynactin subunit 2	1.5	0.015	PRDX5
Q8NI60	COQ8A	Atypical kinase COQ8A, mitochondrial	1.5	0.016	PRDX5
A0A0A0MRT6	ABI1	Abl interactor 1	1.5	0.016	PRDX5
P31327	CPS1	Carbamoyl-phosphate synthase [ammonia], mitochondrial	1.5	0.017	PRDX5
Q9NP97	DYNLRB1	Dynein light chain roadblock-type 1	1.5	0.018	PRDX5
O15143	ARPC1B	Actin-related protein 2/3 complex subunit 1B	1.5	0.019	PRDX5
Q14204	DYNC1H1	Cytoplasmic dynein 1 heavy chain 1	1.5	0.021	PRDX5
A0A2R8YFH5	SEC23B	Protein transport protein SEC23	1.5	0.026	PRDX5
Q9NXH9	TRMT1	tRNA	1.5	0.028	PRDX5
Q7L5D6	GET4	Golgi to ER traffic protein 4 homolog	1.5	0.028	PRDX5
O43347	MSI1	RNA-binding protein Musashi homolog 1	1.5	0.029	PRDX5
Q9H840	GEMIN7	Gem-associated protein 7	1.5	0.034	PRDX5
Q99459	CDC5L	Cell division cycle 5-like protein	1.5	0.040	PRDX5
Q9NY61	AATF	Protein AATF	1.4	0.000	PRDX5
E7ETZ4	BZW2	Basic leucine zipper and W2 domain-containing protein 2	1.4	0.000	PRDX5
Q9HAV7	GRPEL1	GrpE protein homolog 1, mitochondrial	1.4	0.000	PRDX5
P50542	PEX5	Peroxisomal targeting signal 1 receptor	1.4	0.000	PRDX5
Q4J6C6	PREPL	Prolyl endopeptidase-like	1.4	0.000	PRDX5
Q99798	ACO2	Aconitate hydratase, mitochondrial	1.4	0.000	PRDX5
O76031	CLPX	ATP-dependent Clp protease ATP-binding subunit clpX-like, mitochondrial	1.4	0.000	PRDX5
A0A0U1RRM6	ENAH	Protein enabled homolog	1.4	0.001	PRDX5
Q9BSH4	TACO1	Translational activator of cytochrome c oxidase 1	1.4	0.001	PRDX5
O60684	KPNA6	Importin subunit alpha-7	1.4	0.001	PRDX5
P54136	RARS1	Arginine--tRNA ligase, cytoplasmic	1.4	0.001	PRDX5
Q8WUM0	NUP133	Nuclear pore complex protein Nup133	1.4	0.002	PRDX5
Q8WXF1	PSPC1	Paraspeckle component 1	1.4	0.002	PRDX5
G5E9W7	MRPS22	28S ribosomal protein S22, mitochondrial	1.4	0.002	PRDX5
Q12849	GRSF1	G-rich sequence factor 1	1.4	0.002	PRDX5
O15397	IPO8	Importin-8	1.4	0.002	PRDX5
Q93034	CUL5	Cullin-5	1.4	0.002	PRDX5
A0A0G2JNZ2	SCRIB	Protein scribble homolog	1.4	0.002	PRDX5
Q92621	NUP205	Nuclear pore complex protein Nup205	1.4	0.002	PRDX5
P11172	UMPS	Uridine 5'-monophosphate synthase	1.4	0.003	PRDX5
Q7L1Q6	BZW1	Basic leucine zipper and W2 domain-containing protein 1	1.4	0.003	PRDX5
Q9Y678	COPG1	Coatomer subunit gamma-1	1.4	0.003	PRDX5
P84103	SRSF3	Serine/arginine-rich splicing factor 3	1.4	0.003	PRDX5
B7Z6D5	DDX27	RNA helicase	1.4	0.004	PRDX5
Q00610	CLTC	Clathrin heavy chain 1	1.4	0.004	PRDX5
P52209	PGD	6-phosphogluconate dehydrogenase, decarboxylating	1.4	0.004	PRDX5
P04843	RPN1	Dolichyl-diphosphooligosaccharide--protein glycosyltransferase subunit 1	1.4	0.004	PRDX5
D6R938	CAMK2D	Calcium/calmodulin-dependent protein kinase	1.4	0.005	PRDX5
O14980	XPO1	Exportin-1	1.4	0.006	PRDX5
P19367	HK1	Hexokinase-1	1.4	0.006	PRDX5
P33992	MCM5	DNA replication licensing factor MCM5	1.4	0.006	PRDX5
Q5JTH9	RRP12	RRP12-like protein	1.4	0.006	PRDX5
Q6P1M0	SLC27A4	Long-chain fatty acid transport protein 4	1.4	0.006	PRDX5
P07196	NEFL	Neurofilament light polypeptide	1.4	0.007	PRDX5
Q96IU4	ABHD14B	Protein ABHD14B	1.4	0.007	PRDX5
Q6P2Q9	PRPF8	Pre-mRNA-processing-splicing factor 8	1.4	0.007	PRDX5
Q9HB07	MYG1	MYG1 exonuclease	1.4	0.007	PRDX5
Q15061	WDR43	WD repeat-containing protein 43	1.4	0.009	PRDX5
Q7Z4Q2	HEATR3	HEAT repeat-containing protein 3	1.4	0.010	PRDX5
Q9Y262	EIF3L	Eukaryotic translation initiation factor 3 subunit L	1.4	0.012	PRDX5
Q5JRX3	PITRM1	Presequence protease, mitochondrial	1.4	0.012	PRDX5
Q14684	RRP1B	Ribosomal RNA processing protein 1 homolog B	1.4	0.018	PRDX5
Q9NWB6	ARGLU1	Arginine and glutamate-rich protein 1	1.4	0.021	PRDX5
O00411	POLRMT	DNA-directed RNA polymerase, mitochondrial	1.4	0.024	PRDX5
Q96KM6	ZNF512B	Zinc finger protein 512B	1.4	0.026	PRDX5
O60306	AQR	RNA helicase aquarius	1.4	0.029	PRDX5
P23284	PPIB	Peptidyl-prolyl cis-trans isomerase B	1.4	0.030	PRDX5
Q86W42	THOC6	THO complex subunit 6 homolog	1.4	0.031	PRDX5
Q9Y580	RBM7	RNA-binding protein 7	1.4	0.033	PRDX5
Q96L92	SNX27	Sorting nexin-27	1.4	0.034	PRDX5
O75146	HIP1R	Huntingtin-interacting protein 1-related protein	1.4	0.039	PRDX5
Q6P1N0	CC2D1A	Coiled-coil and C2 domain-containing protein 1A	1.3	0.000	PRDX5
Q8N335	GPD1L	Glycerol-3-phosphate dehydrogenase 1-like protein	1.3	0.000	PRDX5
Q8NCA5	FAM98A	Protein FAM98A	1.3	0.000	PRDX5
P26196	DDX6	Probable ATP-dependent RNA helicase DDX6	1.3	0.000	PRDX5
A0A2R8Y855	SMARCE1	SWI/SNF-related matrix-associated actin-dependent regulator of chromatin subfamily E member 1	1.3	0.001	PRDX5
Q14137	BOP1	Ribosome biogenesis protein BOP1	1.3	0.001	PRDX5
P55060	CSE1L	Exportin-2	1.3	0.001	PRDX5
J3QLD9	FLOT2	Flotillin	1.3	0.001	PRDX5
Q9NV70	EXOC1	Exocyst complex component 1	1.3	0.001	PRDX5
A1X283	SH3PXD2B	SH3 and PX domain-containing protein 2B	1.3	0.001	PRDX5
P35237	SERPINB6	Serpin B6	1.3	0.002	PRDX5
P62495	ETF1	Eukaryotic peptide chain release factor subunit 1	1.3	0.002	PRDX5
Q15785	TOMM34	Mitochondrial import receptor subunit TOM34	1.3	0.002	PRDX5
P50213	IDH3A	Isocitrate dehydrogenase [NAD] subunit alpha, mitochondrial	1.3	0.002	PRDX5
Q96EY7	PTCD3	Pentatricopeptide repeat domain-containing protein 3, mitochondrial	1.3	0.002	PRDX5
Q8N122	RPTOR	Regulatory-associated protein of mTOR	1.3	0.002	PRDX5
P23258	TUBG1	Tubulin gamma-1 chain	1.3	0.003	PRDX5
Q9P0K7	RAI14	Ankycorbin	1.3	0.003	PRDX5
P82933	MRPS9	28S ribosomal protein S9, mitochondrial	1.3	0.003	PRDX5
Q02252	ALDH6A1	Methylmalonate-semialdehyde dehydrogenase [acylating], mitochondrial	1.3	0.003	PRDX5
O00567	NOP56	Nucleolar protein 56	1.3	0.003	PRDX5
A5YKK6	CNOT1	CCR4-NOT transcription complex subunit 1	1.3	0.004	PRDX5
Q14258	TRIM25	E3 ubiquitin/ISG15 ligase TRIM25	1.3	0.004	PRDX5
Q9BYN8	MRPS26	28S ribosomal protein S26, mitochondrial	1.3	0.004	PRDX5
Q96N67	DOCK7	Dedicator of cytokinesis protein 7	1.3	0.006	PRDX5
P35659	DEK	Protein DEK	1.3	0.007	PRDX5
P48735	IDH2	Isocitrate dehydrogenase [NADP], mitochondrial	1.3	0.007	PRDX5
Q02241	KIF23	Kinesin-like protein KIF23	1.3	0.007	PRDX5
Q8TEM1	NUP210	Nuclear pore membrane glycoprotein 210	1.3	0.007	PRDX5
Q13190	STX5	Syntaxin-5	1.3	0.009	PRDX5
O14964	HGS	Hepatocyte growth factor-regulated tyrosine kinase substrate	1.3	0.010	PRDX5
Q7KZ85	SUPT6H	Transcription elongation factor SPT6	1.3	0.012	PRDX5
E7ESP9	NEFM	160 kDa neurofilament protein	1.3	0.015	PRDX5
Q86XI2	NCAPG2	Condensin-2 complex subunit G2	1.3	0.017	PRDX5
A0A087WZ13	RAVER1	Ribonucleoprotein PTB-binding 1	1.3	0.020	PRDX5
Q96GD0	PDXP	Pyridoxal phosphate phosphatase	1.3	0.023	PRDX5
O14974	PPP1R12A	Protein phosphatase 1 regulatory subunit 12A	1.3	0.024	PRDX5
Q9BXJ9	NAA15	N-alpha-acetyltransferase 15, NatA auxiliary subunit	1.3	0.024	PRDX5
P62318	SNRPD3	Small nuclear ribonucleoprotein Sm D3	1.3	0.024	PRDX5
Q99959	PKP2	Plakophilin-2	1.3	0.027	PRDX5
Q9Y2R5	MRPS17	28S ribosomal protein S17, mitochondrial	1.3	0.030	PRDX5
F8W108	ARID2	AT-rich interactive domain-containing protein 2	1.3	0.031	PRDX5
Q92878	RAD50	DNA repair protein RAD50	1.3	0.034	PRDX5
Q15058	KIF14	Kinesin-like protein KIF14	1.3	0.037	PRDX5
P42025	ACTR1B	Beta-centractin	1.3	0.038	PRDX5
Q9Y5L0	TNPO3	Transportin-3	1.3	0.039	PRDX5
Q96MW1	CCDC43	Coiled-coil domain-containing protein 43	1.3	0.040	PRDX5
Q969Z0	TBRG4	FAST kinase domain-containing protein 4	1.3	0.046	PRDX5
Q6PGP7	TTC37	Tetratricopeptide repeat protein 37	1.3	0.047	PRDX5
O75643	SNRNP200	U5 small nuclear ribonucleoprotein 200 kDa helicase	1.3	0.048	PRDX5
P48449	LSS	Lanosterol synthase	1.3	0.049	PRDX5
P60983	GMFB	Glia maturation factor beta	1.2	0.000	PRDX5
Q9BSJ2	TUBGCP2	Gamma-tubulin complex component 2	1.2	0.000	PRDX5
Q9UPT5	EXOC7	Exocyst complex component 7	1.2	0.000	PRDX5
Q15555	MAPRE2	Microtubule-associated protein RP/EB family member 2	1.2	0.000	PRDX5
O95613	PCNT	Pericentrin	1.2	0.000	PRDX5
Q5H928	HSD17B10	3-hydroxyacyl-CoA dehydrogenase type-2	1.2	0.000	PRDX5
Q9BTW9	TBCD	Tubulin-specific chaperone D	1.2	0.000	PRDX5
Q9P2R3	ANKFY1	Rabankyrin-5	1.2	0.001	PRDX5
P35249	RFC4	Replication factor C subunit 4	1.2	0.001	PRDX5
A0A140T9R1	FLOT1	Flotillin	1.2	0.001	PRDX5
Q5QJE6	DNTTIP2	Deoxynucleotidyltransferase terminal-interacting protein 2	1.2	0.002	PRDX5
P35573	AGL	Glycogen debranching enzyme	1.2	0.002	PRDX5
P57740	NUP107	Nuclear pore complex protein Nup107	1.2	0.002	PRDX5
P56182	RRP1	Ribosomal RNA processing protein 1 homolog A	1.2	0.002	PRDX5
Q5JTZ9	AARS2	Alanine--tRNA ligase, mitochondrial	1.2	0.003	PRDX5
Q8TEQ6	GEMIN5	Gem-associated protein 5	1.2	0.003	PRDX5
P63092	GNAS	Guanine nucleotide-binding protein G(s) subunit alpha isoforms short	1.2	0.003	PRDX5
Q9Y2H6	FNDC3A	Fibronectin type-III domain-containing protein 3A	1.2	0.003	PRDX5
P51452	DUSP3	Dual specificity protein phosphatase 3	1.2	0.003	PRDX5
Q9BSD7	NTPCR	Cancer-related nucleoside-triphosphatase	1.2	0.003	PRDX5
Q5T4S7	UBR4	E3 ubiquitin-protein ligase UBR4	1.2	0.004	PRDX5
O43795	MYO1B	Unconventional myosin-Ib	1.2	0.004	PRDX5
Q9Y3T9	NOC2L	Nucleolar complex protein 2 homolog	1.2	0.004	PRDX5
Q15031	LARS2	Probable leucine--tRNA ligase, mitochondrial	1.2	0.004	PRDX5
Q00688	FKBP3	Peptidyl-prolyl cis-trans isomerase FKBP3	1.2	0.005	PRDX5
Q09666	AHNAK	Neuroblast differentiation-associated protein AHNAK	1.2	0.005	PRDX5
P09543	CNP	2',3'-cyclic-nucleotide 3'-phosphodiesterase	1.2	0.006	PRDX5
Q15042	RAB3GAP1	Rab3 GTPase-activating protein catalytic subunit	1.2	0.006	PRDX5
Q96QK1	VPS35	Vacuolar protein sorting-associated protein 35	1.2	0.006	PRDX5
P25685	DNAJB1	DnaJ homolog subfamily B member 1	1.2	0.006	PRDX5
Q8WYP5	AHCTF1	Protein ELYS	1.2	0.008	PRDX5
Q92665	MRPS31	28S ribosomal protein S31, mitochondrial	1.2	0.008	PRDX5
Q8IWX8	CHERP	Calcium homeostasis endoplasmic reticulum protein	1.2	0.009	PRDX5
D6REA0	GATB	Glutamyl-tRNA(Gln) amidotransferase subunit B, mitochondrial	1.2	0.009	PRDX5
P16615	ATP2A2	Sarcoplasmic/endoplasmic reticulum calcium ATPase 2	1.2	0.009	PRDX5
A0A0A0MRJ0	CDC42BPA	Non-specific serine/threonine protein kinase	1.2	0.010	PRDX5
P19338	NCL	Nucleolin	1.2	0.010	PRDX5
P30520	ADSS2	Adenylosuccinate synthetase isozyme 2	1.2	0.011	PRDX5
P52701	MSH6	DNA mismatch repair protein Msh6	1.2	0.013	PRDX5
Q8WVM8	SCFD1	Sec1 family domain-containing protein 1	1.2	0.018	PRDX5
O14828	SCAMP3	Secretory carrier-associated membrane protein 3	1.2	0.018	PRDX5
Q9NU22	MDN1	Midasin	1.2	0.019	PRDX5
O00566	MPHOSPH10	U3 small nucleolar ribonucleoprotein protein MPP10	1.2	0.021	PRDX5
A0A3B3ITJ4	HNRNPL	Heterogeneous nuclear ribonucleoprotein L	1.2	0.022	PRDX5
P49642	PRIM1	DNA primase small subunit	1.2	0.039	PRDX5
Q5TG40	DMAP1	DNA methyltransferase 1-associated protein 1	1.2	0.044	PRDX5
P46060	RANGAP1	Ran GTPase-activating protein 1	1.2	0.045	PRDX5
Q9H2M9	RAB3GAP2	Rab3 GTPase-activating protein non-catalytic subunit	1.1	0.000	PRDX5
O94874	UFL1	E3 UFM1-protein ligase 1	1.1	0.000	PRDX5
O95292	VAPB	Vesicle-associated membrane protein-associated protein B/C	1.1	0.000	PRDX5
Q6UWP7	LCLAT1	Lysocardiolipin acyltransferase 1	1.1	0.000	PRDX5
Q6P3W7	SCYL2	SCY1-like protein 2	1.1	0.000	PRDX5
Q8TC12	RDH11	Retinol dehydrogenase 11	1.1	0.000	PRDX5
O75396	SEC22B	Vesicle-trafficking protein SEC22b	1.1	0.001	PRDX5
P35221	CTNNA1	Catenin alpha-1	1.1	0.001	PRDX5
P23919	DTYMK	Thymidylate kinase	1.1	0.001	PRDX5
O14530	TXNDC9	Thioredoxin domain-containing protein 9	1.1	0.001	PRDX5
C9JRJ5	LIMD1	LIM domain-containing protein 1	1.1	0.001	PRDX5
Q6P9B6	MEAK7	MTOR-associated protein MEAK7	1.1	0.001	PRDX5
Q96A33	CCDC47	Coiled-coil domain-containing protein 47	1.1	0.001	PRDX5
E9PS17	SCYL1	N-terminal kinase-like protein	1.1	0.001	PRDX5
Q14166	TTLL12	Tubulin--tyrosine ligase-like protein 12	1.1	0.002	PRDX5
Q6NVY1	HIBCH	3-hydroxyisobutyryl-CoA hydrolase, mitochondrial	1.1	0.002	PRDX5
P68104	EEF1A1	Elongation factor 1-alpha 1	1.1	0.002	PRDX5
Q8IXI1	RHOT2	Mitochondrial Rho GTPase 2	1.1	0.003	PRDX5
Q96P70	IPO9	Importin-9	1.1	0.003	PRDX5
Q4G0F5	VPS26B	Vacuolar protein sorting-associated protein 26B	1.1	0.004	PRDX5
P63010	AP2B1	AP-2 complex subunit beta	1.1	0.004	PRDX5
Q8IWC1	MAP7D3	MAP7 domain-containing protein 3	1.1	0.004	PRDX5
A0A3B3IS71	RB1	Retinoblastoma-associated protein	1.1	0.004	PRDX5
A0A2R8YDQ9	SUCLA2	Succinate--CoA ligase [ADP-forming] subunit beta, mitochondrial	1.1	0.004	PRDX5
A0A1B0GV47	KIF21A	Kinesin-like protein KIF21A	1.1	0.005	PRDX5
Q01968	OCRL	Inositol polyphosphate 5-phosphatase OCRL	1.1	0.005	PRDX5
Q9NZL4	HSPBP1	Hsp70-binding protein 1	1.1	0.006	PRDX5
Q10713	PMPCA	Mitochondrial-processing peptidase subunit alpha	1.1	0.006	PRDX5
P33993	MCM7	DNA replication licensing factor MCM7	1.1	0.006	PRDX5
Q8IX01	SUGP2	SURP and G-patch domain-containing protein 2	1.1	0.007	PRDX5
Q8IY81	FTSJ3	pre-rRNA 2'-O-ribose RNA methyltransferase FTSJ3	1.1	0.007	PRDX5
Q9BPX3	NCAPG	Condensin complex subunit 3	1.1	0.007	PRDX5
P53618	COPB1	Coatomer subunit beta	1.1	0.007	PRDX5
Q9Y237	PIN4	Peptidyl-prolyl cis-trans isomerase NIMA-interacting 4	1.1	0.007	PRDX5
P46778	RPL21	60S ribosomal protein L21	1.1	0.007	PRDX5
Q9BYG3	NIFK	MKI67 FHA domain-interacting nucleolar phosphoprotein	1.1	0.008	PRDX5
Q9BXY0	MAK16	Protein MAK16 homolog	1.1	0.008	PRDX5
P55735	SEC13	Protein SEC13 homolog	1.1	0.009	PRDX5
Q16512	PKN1	Serine/threonine-protein kinase N1	1.1	0.009	PRDX5
O76094	SRP72	Signal recognition particle subunit SRP72	1.1	0.010	PRDX5
Q7L0Y3	TRMT10C	tRNA methyltransferase 10 homolog C	1.1	0.011	PRDX5
Q9NSK0	KLC4	Kinesin light chain 4	1.1	0.012	PRDX5
Q8IYI6	EXOC8	Exocyst complex component 8	1.1	0.013	PRDX5
Q14240	EIF4A2	Eukaryotic initiation factor 4A-II	1.1	0.014	PRDX5
Q9BQ69	MACROD1	ADP-ribose glycohydrolase MACROD1	1.1	0.016	PRDX5
P78344	EIF4G2	Eukaryotic translation initiation factor 4 gamma 2	1.1	0.016	PRDX5
Q14318	FKBP8	Peptidyl-prolyl cis-trans isomerase FKBP8	1.1	0.017	PRDX5
Q8N3C0	ASCC3	Activating signal cointegrator 1 complex subunit 3	1.1	0.017	PRDX5
Q9UFC0	LRWD1	Leucine-rich repeat and WD repeat-containing protein 1	1.1	0.018	PRDX5
Q9ULX6	AKAP8L	A-kinase anchor protein 8-like	1.1	0.019	PRDX5
Q05655	PRKCD	Protein kinase C delta type	1.1	0.019	PRDX5
Q5T7U1	GTF3C5	General transcription factor 3C polypeptide 5	1.1	0.021	PRDX5
Q9BUF5	TUBB6	Tubulin beta-6 chain	1.1	0.021	PRDX5
P33176	KIF5B	Kinesin-1 heavy chain	1.1	0.022	PRDX5
Q8N9T8	KRI1	Protein KRI1 homolog	1.1	0.023	PRDX5
E7EVI4	TACC1	Transforming acidic coiled-coil-containing protein 1	1.1	0.024	PRDX5
P00568	AK1	Adenylate kinase isoenzyme 1	1.1	0.024	PRDX5
Q08945	SSRP1	FACT complex subunit SSRP1	1.1	0.024	PRDX5
P08754	GNAI3	Guanine nucleotide-binding protein G(i) subunit alpha	1.1	0.025	PRDX5
Q5SY16	NOL9	Polynucleotide 5'-hydroxyl-kinase NOL9	1.1	0.026	PRDX5
O75436	VPS26A	Vacuolar protein sorting-associated protein 26A	1.1	0.027	PRDX5
P48507	GCLM	Glutamate--cysteine ligase regulatory subunit	1.1	0.029	PRDX5
F2Z388	RPL35	60S ribosomal protein L35	1.1	0.030	PRDX5
Q9H0E2	TOLLIP	Toll-interacting protein	1.1	0.030	PRDX5
Q9NQT5	EXOSC3	Exosome complex component RRP40	1.1	0.031	PRDX5
Q99961	SH3GL1	Endophilin-A2	1.1	0.037	PRDX5
Q9UG63	ABCF2	ATP-binding cassette sub-family F member 2	1.1	0.038	PRDX5
H3BTL2	BCKDK	Protein-serine/threonine kinase	1.1	0.038	PRDX5
P49458	SRP9	Signal recognition particle 9 kDa protein	1.1	0.039	PRDX5
P47897	QARS1	Glutamine--tRNA ligase	1.1	0.039	PRDX5
A0A087WU06	TUBGCP3	Gamma-tubulin complex component	1.1	0.039	PRDX5
Q9HCD5	NCOA5	Nuclear receptor coactivator 5	1.1	0.041	PRDX5
O60783	MRPS14	28S ribosomal protein S14, mitochondrial	1.1	0.047	PRDX5
G8JLH9	STAT3	Signal transducer and activator of transcription	1.0	0.000	PRDX5
P11498	PC	Pyruvate carboxylase, mitochondrial	1.0	0.000	PRDX5
Q6IAA8	LAMTOR1	Ragulator complex protein LAMTOR1	1.0	0.000	PRDX5
P41240	CSK	Tyrosine-protein kinase CSK	1.0	0.001	PRDX5
Q96SK2	TMEM209	Transmembrane protein 209	1.0	0.001	PRDX5
Q9BRA2	TXNDC17	Thioredoxin domain-containing protein 17	1.0	0.002	PRDX5
O00203	AP3B1	AP-3 complex subunit beta-1	1.0	0.002	PRDX5
Q86VS8	HOOK3	Protein Hook homolog 3	1.0	0.002	PRDX5
Q13451	FKBP5	Peptidyl-prolyl cis-trans isomerase FKBP5	1.0	0.003	PRDX5
Q9H3P7	ACBD3	Golgi resident protein GCP60	1.0	0.003	PRDX5
Q9UPN9	TRIM33	E3 ubiquitin-protein ligase TRIM33	1.0	0.003	PRDX5
Q9NQT4	EXOSC5	Exosome complex component RRP46	1.0	0.004	PRDX5
Q15650	TRIP4	Activating signal cointegrator 1	1.0	0.005	PRDX5
Q12788	TBL3	Transducin beta-like protein 3	1.0	0.005	PRDX5
Q8IWZ3	ANKHD1	Ankyrin repeat and KH domain-containing protein 1	1.0	0.006	PRDX5
Q12769	NUP160	Nuclear pore complex protein Nup160	1.0	0.006	PRDX5
Q16527	CSRP2	Cysteine and glycine-rich protein 2	1.0	0.007	PRDX5
P23921	RRM1	Ribonucleoside-diphosphate reductase large subunit	1.0	0.007	PRDX5
Q92600	CNOT9	CCR4-NOT transcription complex subunit 9	1.0	0.009	PRDX5
Q9UHB9	SRP68	Signal recognition particle subunit SRP68	1.0	0.009	PRDX5
P21964	COMT	Catechol O-methyltransferase	1.0	0.012	PRDX5
P00519	ABL1	Tyrosine-protein kinase ABL1	1.0	0.014	PRDX5
Q8NI27	THOC2	THO complex subunit 2	1.0	0.015	PRDX5
P61163	ACTR1A	Alpha-centractin	1.0	0.015	PRDX5
Q9BQ39	DDX50	ATP-dependent RNA helicase DDX50	1.0	0.015	PRDX5
Q96RQ3	MCCC1	Methylcrotonoyl-CoA carboxylase subunit alpha, mitochondrial	1.0	0.017	PRDX5
O75150	RNF40	E3 ubiquitin-protein ligase BRE1B	1.0	0.024	PRDX5
Q9UNX4	WDR3	WD repeat-containing protein 3	1.0	0.025	PRDX5
Q14232	EIF2B1	Translation initiation factor eIF-2B subunit alpha	1.0	0.027	PRDX5
F8W0J6	NAP1L1	Nucleosome assembly protein 1-like 1	1.0	0.039	PRDX5
Q99569	PKP4	Plakophilin-4	1.0	0.039	PRDX5
Q5T760	SRSF11	Serine/arginine-rich-splicing factor 11	1.0	0.039	PRDX5
Q9Y5K6	CD2AP	CD2-associated protein	1.0	0.046	PRDX5
O75131	CPNE3	Copine-3	1.0	0.047	PRDX5
P12277	CKB	Creatine kinase B-type	1.0	0.049	PRDX5
**PRDX4 -specific interactors**					
P30048	PRDX3	Thioredoxin-dependent peroxide reductase, mitochondrial	9.3	0.000	PRDX1
P13667	PDIA4	Protein disulfide-isomerase A4	6.7	0.000	PRDX1
Q15084	PDIA6	Protein disulfide-isomerase A6	5.1	0.000	PRDX1
P27797	CALR	Calreticulin	5.1	0.000	PRDX1
P30101	PDIA3	Protein disulfide-isomerase A3	4.4	0.000	PRDX1
P62805	H4C1	Histone H4	4.1	0.000	PRDX1
O76094	SRP72	Signal recognition particle subunit SRP72	4.0	0.000	PRDX1
P02790	HPX	Hemopexin	3.9	0.000	PRDX1
Q12797	ASPH	Aspartyl/asparaginyl beta-hydroxylase	3.8	0.000	PRDX1
Q9NYU2	UGGT1	UDP-glucose:glycoprotein glucosyltransferase 1	3.8	0.000	PRDX1
P07237	P4HB	Protein disulfide-isomerase	3.8	0.000	PRDX1
P27824	CANX	Calnexin	3.8	0.000	PRDX1
O60506	SYNCRIP	Heterogeneous nuclear ribonucleoprotein Q	3.7	0.000	PRDX1
P42704	LRPPRC	Leucine-rich PPR motif-containing protein, mitochondrial	3.7	0.000	PRDX1
Q14257	RCN2	Reticulocalbin-2	3.6	0.000	PRDX1
A0A087X054	HYOU1	Hypoxia up-regulated protein 1	3.5	0.000	PRDX1
P11047	LAMC1	Laminin subunit gamma-1	3.3	0.000	PRDX1
P05387	RPLP2	60S acidic ribosomal protein P2	3.3	0.000	PRDX1
P11021	HSPA5	Endoplasmic reticulum chaperone BiP	3.3	0.000	PRDX1
P35637	FUS	RNA-binding protein FUS	3.2	0.000	PRDX1
Q13217	DNAJC3	DnaJ homolog subfamily C member 3	3.2	0.000	PRDX1
O00469	PLOD2	Procollagen-lysine,2-oxoglutarate 5-dioxygenase 2	3.2	0.001	PRDX1
Q9BS26	ERP44	Endoplasmic reticulum resident protein 44	3.2	0.016	PRDX1
P40429	RPL13A	60S ribosomal protein L13a	3.1	0.000	PRDX1
P05386	RPLP1	60S acidic ribosomal protein P1	3.0	0.000	PRDX1
Q9UBS4	DNAJB11	DnaJ homolog subfamily B member 11	2.9	0.000	PRDX1
P98175	RBM10	RNA-binding protein 10	2.9	0.000	PRDX1
P78417	GSTO1	Glutathione S-transferase omega-1	2.7	0.000	PRDX1
C9J4Z3	RPL37A	60S ribosomal protein L37a	2.7	0.000	PRDX1
P14625	HSP90B1	Endoplasmin	2.7	0.000	PRDX1
B9A018	USP39	U4/U6.U5 tri-snRNP-associated protein 2	2.6	0.000	PRDX1
P23588	EIF4B	Eukaryotic translation initiation factor 4B	2.6	0.000	PRDX1
P19338	NCL	Nucleolin	2.6	0.000	PRDX1
P50454	SERPINH1	Serpin H1	2.6	0.000	PRDX1
Q9BZQ6	EDEM3	ER degradation-enhancing alpha-mannosidase-like protein 3	2.6	0.001	PRDX1
D6RBV2	LMAN2	Vesicular integral-membrane protein VIP36	2.6	0.002	PRDX1
P62314	SNRPD1	Small nuclear ribonucleoprotein Sm D1	2.5	0.000	PRDX1
P18077	RPL35A	60S ribosomal protein L35a	2.5	0.000	PRDX1
O00268	TAF4	Transcription initiation factor TFIID subunit 4	2.5	0.000	PRDX1
P12109	COL6A1	Collagen alpha-1(VI) chain	2.5	0.002	PRDX1
Q9UBF2	COPG2	Coatomer subunit gamma-2	2.4	0.000	PRDX1
Q8TEM1	NUP210	Nuclear pore membrane glycoprotein 210	2.4	0.001	PRDX1
Q15293	RCN1	Reticulocalbin-1	2.4	0.001	PRDX1
Q9Y680	FKBP7	Peptidyl-prolyl cis-trans isomerase FKBP7	2.4	0.003	PRDX1
Q15459	SF3A1	Splicing factor 3A subunit 1	2.4	0.049	PRDX1
P49257	LMAN1	Protein ERGIC-53	2.3	0.001	PRDX1
P36957	DLST	Dihydrolipoyllysine-residue succinyltransferase component of 2-oxoglutarate dehydrogenase complex, mitochondrial	2.2	0.000	PRDX1
Q02750	MAP2K1	Dual specificity mitogen-activated protein kinase kinase 1	2.1	0.000	PRDX1
Q9H8Y8	GORASP2	Golgi reassembly-stacking protein 2	2.1	0.000	PRDX1
P06730	EIF4E	Eukaryotic translation initiation factor 4E	2.1	0.000	PRDX1
Q9UIG0	BAZ1B	Tyrosine-protein kinase BAZ1B	2.1	0.000	PRDX1
P31942	HNRNPH3	Heterogeneous nuclear ribonucleoprotein H3	2.1	0.000	PRDX1
P26196	DDX6	Probable ATP-dependent RNA helicase DDX6	2.1	0.000	PRDX1
Q13257	MAD2L1	Mitotic spindle assembly checkpoint protein MAD2A	2.1	0.001	PRDX1
Q8NI27	THOC2	THO complex subunit 2	2.1	0.001	PRDX1
Q9BQG0	MYBBP1A	Myb-binding protein 1A	2.1	0.001	PRDX1
Q8IXB1	DNAJC10	DnaJ homolog subfamily C member 10	2.1	0.002	PRDX1
Q5SY16	NOL9	Polynucleotide 5'-hydroxyl-kinase NOL9	2.1	0.002	PRDX1
P55268	LAMB2	Laminin subunit beta-2	2.1	0.003	PRDX1
P53350	PLK1	Serine/threonine-protein kinase PLK1	2.1	0.025	PRDX1
P09972	ALDOC	Fructose-bisphosphate aldolase C	2.1	0.048	PRDX1
P13674	P4HA1	Prolyl 4-hydroxylase subunit alpha-1	2.0	0.000	PRDX1
O75821	EIF3G	Eukaryotic translation initiation factor 3 subunit G	2.0	0.000	PRDX1
Q4J6C6	PREPL	Prolyl endopeptidase-like	2.0	0.001	PRDX1
D6RA00	ENOPH1	Enolase-phosphatase E1	2.0	0.003	PRDX1
P11388	TOP2A	DNA topoisomerase 2-alpha	2.0	0.003	PRDX1
Q13162	PRDX4	Peroxiredoxin-4	11.3	0.000	PRDX1
Q9H0A0	NAT10	RNA cytidine acetyltransferase	1.9	0.000	PRDX1
P50502	ST13	Hsc70-interacting protein	1.9	0.000	PRDX1
P55769	SNU13	NHP2-like protein 1	1.9	0.000	PRDX1
O75832	PSMD10	26S proteasome non-ATPase regulatory subunit 10	1.9	0.000	PRDX1
Q9Y3Z3	SAMHD1	Deoxynucleoside triphosphate triphosphohydrolase SAMHD1	1.9	0.000	PRDX1
O00203	AP3B1	AP-3 complex subunit beta-1	1.9	0.000	PRDX1
Q96H79	ZC3HAV1L	Zinc finger CCCH-type antiviral protein 1-like	1.9	0.000	PRDX1
P14324	FDPS	Farnesyl pyrophosphate synthase	1.9	0.000	PRDX1
G3V529	DDX24	RNA helicase	1.9	0.001	PRDX1
Q9ULC3	RAB23	Ras-related protein Rab-23	1.9	0.003	PRDX1
P11182	DBT	Lipoamide acyltransferase component of branched-chain alpha-keto acid dehydrogenase complex, mitochondrial	1.9	0.004	PRDX1
O94925	GLS	Glutaminase kidney isoform, mitochondrial	1.9	0.006	PRDX1
P63167	DYNLL1	Dynein light chain 1, cytoplasmic	1.9	0.019	PRDX1
A0A0B4J1Z1	SRSF7	Serine/arginine-rich-splicing factor 7	1.9	0.028	PRDX1
P38159	RBMX	RNA-binding motif protein, X chromosome	1.9	0.029	PRDX1
P11387	TOP1	DNA topoisomerase 1	1.8	0.000	PRDX1
P49321	NASP	Nuclear autoantigenic sperm protein	1.8	0.000	PRDX1
P53985	SLC16A1	Monocarboxylate transporter 1	1.8	0.000	PRDX1
P40938	RFC3	Replication factor C subunit 3	1.8	0.000	PRDX1
O75688	PPM1B	Protein phosphatase 1B	1.8	0.001	PRDX1
P52597	HNRNPF	Heterogeneous nuclear ribonucleoprotein F	1.8	0.001	PRDX1
Q92621	NUP205	Nuclear pore complex protein Nup205	1.8	0.002	PRDX1
Q93008	USP9X	Probable ubiquitin carboxyl-terminal hydrolase FAF-X	1.8	0.002	PRDX1
O95104	SCAF4	SR-related and CTD-associated factor 4	1.8	0.006	PRDX1
C9J384	CMSS1	Protein CMSS1	1.8	0.009	PRDX1
Q9NQ29	LUC7L	Putative RNA-binding protein Luc7-like 1	1.8	0.009	PRDX1
P62750	RPL23A	60S ribosomal protein L23a	1.7	0.000	PRDX1
P52565	ARHGDIA	Rho GDP-dissociation inhibitor 1	1.7	0.000	PRDX1
E9PIF2	DDX10	RNA helicase	1.7	0.000	PRDX1
Q15691	MAPRE1	Microtubule-associated protein RP/EB family member 1	1.7	0.000	PRDX1
Q15393	SF3B3	Splicing factor 3B subunit 3	1.7	0.002	PRDX1
Q09028	RBBP4	Histone-binding protein RBBP4	1.7	0.002	PRDX1
P41252	IARS1	Isoleucine--tRNA ligase, cytoplasmic	1.7	0.002	PRDX1
P08758	ANXA5	Annexin A5	1.7	0.003	PRDX1
P22307	SCP2	Non-specific lipid-transfer protein	1.7	0.003	PRDX1
Q01085	TIAL1	Nucleolysin TIAR	1.7	0.007	PRDX1
Q9Y2H1	STK38L	Serine/threonine-protein kinase 38-like	1.7	0.007	PRDX1
P63244	RACK1	Receptor of activated protein C kinase 1	1.7	0.010	PRDX1
P04844	RPN2	Dolichyl-diphosphooligosaccharide--protein glycosyltransferase subunit 2	1.7	0.013	PRDX1
P61353	RPL27	60S ribosomal protein L27	1.7	0.016	PRDX1
E9PFZ2	CP	Ceruloplasmin	1.7	0.024	PRDX1
P35579	MYH9	Myosin-9	1.7	0.026	PRDX1
Q53GQ0	HSD17B12	Very-long-chain 3-oxoacyl-CoA reductase	1.7	0.028	PRDX1
Q15291	RBBP5	Retinoblastoma-binding protein 5	1.6	0.000	PRDX1
Q14697	GANAB	Neutral alpha-glucosidase AB	1.6	0.000	PRDX1
O60934	NBN	Nibrin	1.6	0.000	PRDX1
P09211	GSTP1	Glutathione S-transferase P	1.6	0.000	PRDX1
P32969	RPL9	60S ribosomal protein L9	1.6	0.000	PRDX1
P43034	PAFAH1B1	Platelet-activating factor acetylhydrolase IB subunit beta	1.6	0.000	PRDX1
P34949	MPI	Mannose-6-phosphate isomerase	1.6	0.000	PRDX1
P62424	RPL7A	60S ribosomal protein L7a	1.6	0.000	PRDX1
P56192	MARS1	Methionine--tRNA ligase, cytoplasmic	1.6	0.001	PRDX1
Q16531	DDB1	DNA damage-binding protein 1	1.6	0.001	PRDX1
O43747	AP1G1	AP-1 complex subunit gamma-1	1.6	0.001	PRDX1
Q01831	XPC	DNA repair protein complementing XP-C cells	1.6	0.001	PRDX1
O43852	CALU	Calumenin	1.6	0.002	PRDX1
Q96T23	RSF1	Remodeling and spacing factor 1	1.6	0.008	PRDX1
Q5VW32	BROX	BRO1 domain-containing protein BROX	1.6	0.008	PRDX1
P46821	MAP1B	Microtubule-associated protein 1B	1.6	0.013	PRDX1
O75367	MACROH2A1	Core histone macro-H2A.1	1.6	0.015	PRDX1
Q9BZK7	TBL1XR1	F-box-like/WD repeat-containing protein TBL1XR1	1.6	0.016	PRDX1
Q01082	SPTBN1	Spectrin beta chain, non-erythrocytic 1	1.6	0.026	PRDX1
P07942	LAMB1	Laminin subunit beta-1	1.6	0.027	PRDX1
Q9NVP1	DDX18	ATP-dependent RNA helicase DDX18	1.6	0.038	PRDX1
Q9BYN0	SRXN1	Sulfiredoxin-1	1.6	0.041	PRDX1
O15372	EIF3H	Eukaryotic translation initiation factor 3 subunit H	1.5	0.000	PRDX1
P38117	ETFB	Electron transfer flavoprotein subunit beta	1.5	0.000	PRDX1
O94826	TOMM70	Mitochondrial import receptor subunit TOM70	1.5	0.000	PRDX1
Q68E01	INTS3	Integrator complex subunit 3	1.5	0.000	PRDX1
A0A2R8YD50	HSD17B4	Peroxisomal multifunctional enzyme type 2	1.5	0.000	PRDX1
O00299	CLIC1	Chloride intracellular channel protein 1	1.5	0.000	PRDX1
P61247	RPS3A	40S ribosomal protein S3a	1.5	0.000	PRDX1
O60763	USO1	General vesicular transport factor p115	1.5	0.000	PRDX1
O95376	ARIH2	E3 ubiquitin-protein ligase ARIH2	1.5	0.001	PRDX1
Q15054	POLD3	DNA polymerase delta subunit 3	1.5	0.001	PRDX1

Q15054	POLD3	DNA polymerase delta subunit 3	1.5	0.001	PRDX1
Q9Y678	COPG1	Coatomer subunit gamma-1	1.5	0.001	PRDX1
O95433	AHSA1	Activator of 90 kDa heat shock protein ATPase homolog 1	1.5	0.002	PRDX1
Q9Y2X3	NOP58	Nucleolar protein 58	1.5	0.010	PRDX1
A0A3B3ISV3	COL4A1	Collagen alpha-1(IV) chain	1.5	0.019	PRDX1
O96019	ACTL6A	Actin-like protein 6A	1.5	0.020	PRDX1
Q9NYB0	TERF2IP	Telomeric repeat-binding factor 2-interacting protein 1	1.5	0.021	PRDX1
P19174	PLCG1	1-phosphatidylinositol 4,5-bisphosphate phosphodiesterase gamma-1	1.5	0.029	PRDX1
Q9UKN8	GTF3C4	General transcription factor 3C polypeptide 4	1.5	0.032	PRDX1
P11717	IGF2R	Cation-independent mannose-6-phosphate receptor	1.5	0.037	PRDX1
P27694	RPA1	Replication protein A 70 kDa DNA-binding subunit	1.5	0.049	PRDX1
Q2TAY7	SMU1	WD40 repeat-containing protein SMU1	1.4	0.000	PRDX1
P42166	TMPO	Lamina-associated polypeptide 2, isoform alpha	1.4	0.000	PRDX1
J3QQW9	SUZ12	Polycomb protein SUZ12	1.4	0.000	PRDX1
Q5T9A4	ATAD3B	ATPase family AAA domain-containing protein 3B	1.4	0.000	PRDX1
Q5H928	HSD17B10	3-hydroxyacyl-CoA dehydrogenase type-2	1.4	0.000	PRDX1
P53618	COPB1	Coatomer subunit beta	1.4	0.001	PRDX1
Q9Y5J1	UTP18	U3 small nucleolar RNA-associated protein 18 homolog	1.4	0.001	PRDX1
Q8IWB7	WDFY1	WD repeat and FYVE domain-containing protein 1	1.4	0.001	PRDX1
P62244	RPS15A	40S ribosomal protein S15a	1.4	0.004	PRDX1
P16615	ATP2A2	Sarcoplasmic/endoplasmic reticulum calcium ATPase 2	1.4	0.004	PRDX1
P26583	HMGB2	High mobility group protein B2	1.4	0.008	PRDX1
P33992	MCM5	DNA replication licensing factor MCM5	1.4	0.008	PRDX1
P20073	ANXA7	Annexin A7	1.4	0.008	PRDX1
P39023	RPL3	60S ribosomal protein L3	1.4	0.010	PRDX1
O43719	HTATSF1	HIV Tat-specific factor 1	1.4	0.013	PRDX1
O14980	XPO1	Exportin-1	1.4	0.015	PRDX1
O00232	PSMD12	26S proteasome non-ATPase regulatory subunit 12	1.4	0.015	PRDX1
P35606	COPB2	Coatomer subunit beta'	1.4	0.016	PRDX1
Q5VYK3	ECPAS	Proteasome adapter and scaffold protein ECM29	1.4	0.027	PRDX1
O76021	RSL1D1	Ribosomal L1 domain-containing protein 1	1.4	0.030	PRDX1
P31327	CPS1	Carbamoyl-phosphate synthase [ammonia], mitochondrial	1.4	0.032	PRDX1
Q8N335	GPD1L	Glycerol-3-phosphate dehydrogenase 1-like protein	1.4	0.035	PRDX1
P39748	FEN1	Flap endonuclease 1	1.4	0.042	PRDX1
P78347	GTF2I	General transcription factor II-I	1.4	0.045	PRDX1
Q86TI2	DPP9	Dipeptidyl peptidase 9	1.4	0.046	PRDX1
Q9Y383	LUC7L2	Putative RNA-binding protein Luc7-like 2	1.3	0.000	PRDX1
Q9UQ80	PA2G4	Proliferation-associated protein 2G4	1.3	0.000	PRDX1
Q8NFH5	NUP35	Nucleoporin NUP35	1.3	0.000	PRDX1
Q12788	TBL3	Transducin beta-like protein 3	1.3	0.001	PRDX1
P49916	LIG3	DNA ligase 3	1.3	0.001	PRDX1
Q9H3P7	ACBD3	Golgi resident protein GCP60	1.3	0.001	PRDX1
K7ERF1	EIF3K	Eukaryotic translation initiation factor 3 subunit K	1.3	0.001	PRDX1
P29401	TKT	Transketolase	1.3	0.001	PRDX1
P62877	RBX1	E3 ubiquitin-protein ligase RBX1	1.3	0.001	PRDX1
P53004	BLVRA	Biliverdin reductase A	1.3	0.002	PRDX1
P61923	COPZ1	Coatomer subunit zeta-1	1.3	0.002	PRDX1
Q96HC4	PDLIM5	PDZ and LIM domain protein 5	1.3	0.002	PRDX1
P62241	RPS8	40S ribosomal protein S8	1.3	0.003	PRDX1
O00116	AGPS	Alkyldihydroxyacetonephosphate synthase, peroxisomal	1.3	0.004	PRDX1
Q92688	ANP32B	Acidic leucine-rich nuclear phosphoprotein 32 family member B	1.3	0.004	PRDX1
J3KMX5	RPS13	40S ribosomal protein S13	1.3	0.010	PRDX1
O94874	UFL1	E3 UFM1-protein ligase 1	1.3	0.010	PRDX1
P48735	IDH2	Isocitrate dehydrogenase [NADP], mitochondrial	1.3	0.015	PRDX1
P62979	RPS27A	Ubiquitin-40S ribosomal protein S27a	1.3	0.017	PRDX1
O75131	CPNE3	Copine-3	1.3	0.018	PRDX1
Q92769	HDAC2	Histone deacetylase 2	1.3	0.027	PRDX1
O95163	ELP1	Elongator complex protein 1	1.3	0.048	PRDX1
Q8ND82	ZNF280C	Zinc finger protein 280C	1.2	0.000	PRDX1
G8JLH9	STAT3	Signal transducer and activator of transcription	1.2	0.000	PRDX1
E5RHG8	ELOC	Elongin-C	1.2	0.000	PRDX1
P52594	AGFG1	Arf-GAP domain and FG repeat-containing protein 1	1.2	0.000	PRDX1
Q5JXI8	FHL1	Four and a half LIM domains protein 1	1.2	0.000	PRDX1
Q13112	CHAF1B	Chromatin assembly factor 1 subunit B	1.2	0.000	PRDX1
Q07020	RPL18	60S ribosomal protein L18	1.2	0.001	PRDX1
Q92600	CNOT9	CCR4-NOT transcription complex subunit 9	1.2	0.001	PRDX1
P15121	AKR1B1	Aldo-keto reductase family 1 member B1	1.2	0.001	PRDX1
E7ESY4	MTA1	Metastasis-associated protein MTA1	1.2	0.003	PRDX1
Q15008	PSMD6	26S proteasome non-ATPase regulatory subunit 6	1.2	0.004	PRDX1
M0QY97	ZC3H4	Zinc finger CCCH domain-containing protein 4	1.2	0.006	PRDX1
P55795	HNRNPH2	Heterogeneous nuclear ribonucleoprotein H2	1.2	0.006	PRDX1
Q9UKM9	RALY	RNA-binding protein Raly	1.2	0.007	PRDX1
Q02878	RPL6	60S ribosomal protein L6	1.2	0.010	PRDX1
Q09161	NCBP1	Nuclear cap-binding protein subunit 1	1.2	0.010	PRDX1
P49750	YLPM1	YLP motif-containing protein 1	1.2	0.010	PRDX1
O95202	LETM1	Mitochondrial proton/calcium exchanger protein	1.2	0.019	PRDX1
Q86UP2	KTN1	Kinectin	1.2	0.022	PRDX1
P42224	STAT1	Signal transducer and activator of transcription 1-alpha/beta	1.2	0.026	PRDX1
Q9Y3T9	NOC2L	Nucleolar complex protein 2 homolog	1.2	0.028	PRDX1
Q9Y5V3	MAGED1	Melanoma-associated antigen D1	1.1	0.001	PRDX1
Q9UBU9	NXF1	Nuclear RNA export factor 1	1.1	0.002	PRDX1
Q9NTI5	PDS5B	Sister chromatid cohesion protein PDS5 homolog B	1.1	0.003	PRDX1
Q9BT78	COPS4	COP9 signalosome complex subunit 4	1.1	0.004	PRDX1
P53007	SLC25A1	Tricarboxylate transport protein, mitochondrial	1.1	0.004	PRDX1
P23921	RRM1	Ribonucleoside-diphosphate reductase large subunit	1.1	0.006	PRDX1
P05455	SSB	Lupus La protein	1.1	0.006	PRDX1
P27144	AK4	Adenylate kinase 4, mitochondrial	1.1	0.007	PRDX1
A0A024R4M0	RPS9	40S ribosomal protein S9	1.1	0.007	PRDX1
P31943	HNRNPH1	Heterogeneous nuclear ribonucleoprotein H	1.1	0.009	PRDX1
O43390	HNRNPR	Heterogeneous nuclear ribonucleoprotein R	1.1	0.012	PRDX1
Q96CS3	FAF2	FAS-associated factor 2	1.1	0.013	PRDX1
P50914	RPL14	60S ribosomal protein L14	1.1	0.013	PRDX1
Q9UHB9	SRP68	Signal recognition particle subunit SRP68	1.1	0.015	PRDX1
Q9UI12	ATP6V1H	V-type proton ATPase subunit H	1.1	0.019	PRDX1
P08133	ANXA6	Annexin A6	1.1	0.022	PRDX1
Q71UM5	RPS27L	40S ribosomal protein S27-like	1.1	0.025	PRDX1
O76003	GLRX3	Glutaredoxin-3	1.1	0.026	PRDX1
P39687	ANP32A	Acidic leucine-rich nuclear phosphoprotein 32 family member A	1.1	0.026	PRDX1
Q96SB4	SRPK1	SRSF protein kinase 1	1.1	0.029	PRDX1
P16435	POR	NADPH--cytochrome P450 reductase	1.1	0.030	PRDX1
F8W6I7	HNRNPA1	Helix-destabilizing protein	1.1	0.031	PRDX1
O75381	PEX14	Peroxisomal membrane protein PEX14	1.1	0.037	PRDX1
P06748	NPM1	Nucleophosmin	1.1	0.039	PRDX1
Q99426	TBCB	Tubulin-folding cofactor B	1.1	0.045	PRDX1
Q9NW13	RBM28	RNA-binding protein 28	1.1	0.048	PRDX1
P78406	RAE1	mRNA export factor	1.0	0.001	PRDX1
Q14974	KPNB1	Importin subunit beta-1	1.0	0.002	PRDX1
Q86VM9	ZC3H18	Zinc finger CCCH domain-containing protein 18	1.0	0.005	PRDX1
Q9Y5P6	GMPPB	Mannose-1-phosphate guanyltransferase beta	1.0	0.005	PRDX1
P45973	CBX5	Chromobox protein homolog 5	1.0	0.008	PRDX1
O00410	IPO5	Importin-5	1.0	0.008	PRDX1
C9JRT6	NUB1	NEDD8 ultimate buster 1	1.0	0.009	PRDX1
P43897	TSFM	Elongation factor Ts, mitochondrial	1.0	0.022	PRDX1
Q9Y2Q3	GSTK1	Glutathione S-transferase kappa 1	1.0	0.026	PRDX1
Q9BQ52	ELAC2	Zinc phosphodiesterase ELAC protein 2	1.0	0.031	PRDX1
P43490	NAMPT	Nicotinamide phosphoribosyltransferase	1.0	0.046	PRDX1
Q13162	PRDX4	Peroxiredoxin-4	9.7	0.000	PRDX2
P13667	PDIA4	Protein disulfide-isomerase A4	5.9	0.000	PRDX2
Q12797	ASPH	Aspartyl/asparaginyl beta-hydroxylase	4.2	0.000	PRDX2
Q9NYU2	UGGT1	UDP-glucose:glycoprotein glucosyltransferase 1	4.0	0.000	PRDX2
Q15084	PDIA6	Protein disulfide-isomerase A6	3.9	0.000	PRDX2
P07237	P4HB	Protein disulfide-isomerase	3.9	0.000	PRDX2
P30048	PRDX3	Thioredoxin-dependent peroxide reductase, mitochondrial	3.6	0.000	PRDX2
P98175	RBM10	RNA-binding protein 10	3.5	0.000	PRDX2
Q13217	DNAJC3	DnaJ homolog subfamily C member 3	3.4	0.000	PRDX2
P11021	HSPA5	Endoplasmic reticulum chaperone BiP	3.4	0.000	PRDX2
A0A087X054	HYOU1	Hypoxia up-regulated protein 1	3.3	0.000	PRDX2
O00469	PLOD2	Procollagen-lysine,2-oxoglutarate 5-dioxygenase 2	3.3	0.001	PRDX2
P11047	LAMC1	Laminin subunit gamma-1	3.1	0.000	PRDX2
Q9BS26	ERP44	Endoplasmic reticulum resident protein 44	3.1	0.020	PRDX2
P30101	PDIA3	Protein disulfide-isomerase A3	2.9	0.000	PRDX2
P27797	CALR	Calreticulin	2.8	0.000	PRDX2
D6RBV2	LMAN2	Vesicular integral-membrane protein VIP36	2.8	0.001	PRDX2
Q9BZQ6	EDEM3	ER degradation-enhancing alpha-mannosidase-like protein 3	2.8	0.001	PRDX2
P78417	GSTO1	Glutathione S-transferase omega-1	2.7	0.000	PRDX2
P12109	COL6A1	Collagen alpha-1(VI) chain	2.7	0.001	PRDX2
O75688	PPM1B	Protein phosphatase 1B	2.6	0.000	PRDX2
P50454	SERPINH1	Serpin H1	2.6	0.000	PRDX2
O00268	TAF4	Transcription initiation factor TFIID subunit 4	2.6	0.000	PRDX2
Q9Y2H1	STK38L	Serine/threonine-protein kinase 38-like	2.6	0.002	PRDX2
Q9Y680	FKBP7	Peptidyl-prolyl cis-trans isomerase FKBP7	2.6	0.002	PRDX2
Q14257	RCN2	Reticulocalbin-2	2.5	0.000	PRDX2
Q8TEM1	NUP210	Nuclear pore membrane glycoprotein 210	2.5	0.000	PRDX2
P49257	LMAN1	Protein ERGIC-53	2.5	0.001	PRDX2
P55268	LAMB2	Laminin subunit beta-2	2.3	0.002	PRDX2
P27824	CANX	Calnexin	2.2	0.000	PRDX2
P09972	ALDOC	Fructose-bisphosphate aldolase C	2.1	0.046	PRDX2
Q15424	SAFB	Scaffold attachment factor B1	2.0	0.000	PRDX2
Q13257	MAD2L1	Mitotic spindle assembly checkpoint protein MAD2A	2.0	0.002	PRDX2
P14625	HSP90B1	Endoplasmin	1.9	0.000	PRDX2
O00399	DCTN6	Dynactin subunit 6	1.9	0.011	PRDX2
E9PFZ2	CP	Ceruloplasmin	1.9	0.013	PRDX2
Q12929	EPS8	Epidermal growth factor receptor kinase substrate 8	1.8	0.010	PRDX2
P57076	CFAP298	Cilia- and flagella-associated protein 298	1.8	0.011	PRDX2
P07942	LAMB1	Laminin subunit beta-1	1.8	0.015	PRDX2
P11717	IGF2R	Cation-independent mannose-6-phosphate receptor	1.8	0.021	PRDX2
O43852	CALU	Calumenin	1.7	0.001	PRDX2
Q5RKV6	EXOSC6	Exosome complex component MTR3	1.7	0.006	PRDX2
Q9UKN8	GTF3C4	General transcription factor 3C polypeptide 4	1.7	0.018	PRDX2
Q9HAU5	UPF2	Regulator of nonsense transcripts 2	1.7	0.019	PRDX2
P13674	P4HA1	Prolyl 4-hydroxylase subunit alpha-1	1.6	0.003	PRDX2
Q32P28	P3H1	Prolyl 3-hydroxylase 1	1.6	0.014	PRDX2
P09110	ACAA1	3-ketoacyl-CoA thiolase, peroxisomal	1.6	0.015	PRDX2
Q8IXB1	DNAJC10	DnaJ homolog subfamily C member 10	1.5	0.002	PRDX2
P23588	EIF4B	Eukaryotic translation initiation factor 4B	1.5	0.011	PRDX2
P67809	YBX1	Y-box-binding protein 1	1.5	0.034	PRDX2
H0YEN2	PPP6R3	Serine/threonine-protein phosphatase 6 regulatory subunit 3	1.5	0.036	PRDX2
Q8N6R0	EEF1AKNMT	eEF1A lysine and N-terminal methyltransferase	1.5	0.037	PRDX2
P62314	SNRPD1	Small nuclear ribonucleoprotein Sm D1	1.4	0.000	PRDX2
Q15020	SART3	Squamous cell carcinoma antigen recognized by T-cells 3	1.4	0.001	PRDX2
Q99504	EYA3	Eyes absent homolog 3	1.4	0.001	PRDX2
P56182	RRP1	Ribosomal RNA processing protein 1 homolog A	1.4	0.001	PRDX2
Q9H0H5	RACGAP1	Rac GTPase-activating protein 1	1.4	0.002	PRDX2
O00116	AGPS	Alkyldihydroxyacetonephosphate synthase, peroxisomal	1.4	0.004	PRDX2
O00170	AIP	AH receptor-interacting protein	1.4	0.005	PRDX2
E7ESP9	NEFM	160 kDa neurofilament protein	1.4	0.017	PRDX2
O60934	NBN	Nibrin	1.3	0.000	PRDX2
A6NHR9	SMCHD1	Structural maintenance of chromosomes flexible hinge domain-containing protein 1	1.3	0.000	PRDX2
Q9UIG0	BAZ1B	Tyrosine-protein kinase BAZ1B	1.3	0.001	PRDX2
Q04837	SSBP1	Single-stranded DNA-binding protein, mitochondrial	1.3	0.002	PRDX2
Q01831	XPC	DNA repair protein complementing XP-C cells	1.3	0.003	PRDX2
P42696	RBM34	RNA-binding protein 34	1.3	0.005	PRDX2
Q9UBE0	SAE1	SUMO-activating enzyme subunit 1	1.3	0.012	PRDX2
P22307	SCP2	Non-specific lipid-transfer protein	1.3	0.014	PRDX2
Q14676	MDC1	Mediator of DNA damage checkpoint protein 1	1.3	0.034	PRDX2
P40938	RFC3	Replication factor C subunit 3	1.2	0.000	PRDX2
P25786	PSMA1	Proteasome subunit alpha type-1	1.2	0.001	PRDX2
B8ZZC5	GLS	Glutaminase	1.2	0.001	PRDX2
Q68E01	INTS3	Integrator complex subunit 3	1.2	0.002	PRDX2
P49916	LIG3	DNA ligase 3	1.2	0.003	PRDX2
P31942	HNRNPH3	Heterogeneous nuclear ribonucleoprotein H3	1.2	0.004	PRDX2
Q9UBS4	DNAJB11	DnaJ homolog subfamily B member 11	1.2	0.004	PRDX2
P18583	SON	Protein SON	1.2	0.008	PRDX2
O94906	PRPF6	Pre-mRNA-processing factor 6	1.2	0.009	PRDX2
M0QY97	ZC3H4	Zinc finger CCCH domain-containing protein 4	1.2	0.009	PRDX2
Q16352	INA	Alpha-internexin	1.2	0.012	PRDX2
Q9BZE4	GTPBP4	Nucleolar GTP-binding protein 1	1.2	0.013	PRDX2
Q02241	KIF23	Kinesin-like protein KIF23	1.2	0.015	PRDX2
P05783	KRT18	Keratin, type I cytoskeletal 18	1.2	0.021	PRDX2
Q99426	TBCB	Tubulin-folding cofactor B	1.2	0.023	PRDX2
O14646	CHD1	Chromodomain-helicase-DNA-binding protein 1	1.2	0.027	PRDX2
Q9Y3D0	CIAO2B	Cytosolic iron-sulfur assembly component 2B	1.2	0.031	PRDX2
O75152	ZC3H11A	Zinc finger CCCH domain-containing protein 11A	1.2	0.042	PRDX2
Q9NVI7	ATAD3A	ATPase family AAA domain-containing protein 3A	1.1	0.000	PRDX2
Q96KR1	ZFR	Zinc finger RNA-binding protein	1.1	0.003	PRDX2
Q15785	TOMM34	Mitochondrial import receptor subunit TOM34	1.1	0.011	PRDX2
Q8IY81	FTSJ3	pre-rRNA 2'-O-ribose RNA methyltransferase FTSJ3	1.1	0.011	PRDX2
C9J384	CMSS1	Protein CMSS1	1.1	0.014	PRDX2
Q9NQ29	LUC7L	Putative RNA-binding protein Luc7-like 1	1.1	0.017	PRDX2
Q8N163	CCAR2	Cell cycle and apoptosis regulator protein 2	1.1	0.021	PRDX2
Q9Y2T2	AP3M1	AP-3 complex subunit mu-1	1.1	0.023	PRDX2
P52701	MSH6	DNA mismatch repair protein Msh6	1.1	0.027	PRDX2
P07196	NEFL	Neurofilament light polypeptide	1.1	0.030	PRDX2
C9J250	RBM6	RNA-binding protein 6	1.1	0.032	PRDX2
P62979	RPS27A	Ubiquitin-40S ribosomal protein S27a	1.1	0.033	PRDX2
Q71UM5	RPS27L	40S ribosomal protein S27-like	1.1	0.045	PRDX2
P04792	HSPB1	Heat shock protein beta-1	1.0	0.000	PRDX2
P02545	LMNA	Prelamin-A/C [Cleaved into: Lamin-A/C	1.0	0.001	PRDX2
Q9BY42	RTF2	Replication termination factor 2	1.0	0.004	PRDX2
Q13451	FKBP5	Peptidyl-prolyl cis-trans isomerase FKBP5	1.0	0.006	PRDX2
Q9NZB2	FAM120A	Constitutive coactivator of PPAR-gamma-like protein 1	1.0	0.007	PRDX2
A0A0A0MRM8	MYO6	Unconventional myosin-6	1.0	0.013	PRDX2
Q96H79	ZC3HAV1L	Zinc finger CCCH-type antiviral protein 1-like	1.0	0.013	PRDX2
P42765	ACAA2	3-ketoacyl-CoA thiolase, mitochondrial	1.0	0.017	PRDX2
Q15042	RAB3GAP1	Rab3 GTPase-activating protein catalytic subunit	1.0	0.023	PRDX2
O95376	ARIH2	E3 ubiquitin-protein ligase ARIH2	1.0	0.027	PRDX2
Q9P0M6	MACROH2A2	Core histone macro-H2A.2	1.0	0.046	PRDX2
Q15084	PDIA6	Protein disulfide-isomerase A6	4.9	0.000	PRDX3
P13667	PDIA4	Protein disulfide-isomerase A4	4.8	0.000	PRDX3
Q9NYU2	UGGT1	UDP-glucose:glycoprotein glucosyltransferase 1	4.2	0.000	PRDX3
P11047	LAMC1	Laminin subunit gamma-1	4.0	0.000	PRDX3
O00469	PLOD2	Procollagen-lysine,2-oxoglutarate 5-dioxygenase 2	3.9	0.000	PRDX3
P07237	P4HB	Protein disulfide-isomerase	3.9	0.000	PRDX3
A0A087X054	HYOU1	Hypoxia up-regulated protein 1	3.9	0.000	PRDX3
Q12797	ASPH	Aspartyl/asparaginyl beta-hydroxylase	3.6	0.000	PRDX3
P11021	HSPA5	Endoplasmic reticulum chaperone BiP	3.5	0.000	PRDX3
P30101	PDIA3	Protein disulfide-isomerase A3	2.9	0.000	PRDX3
Q13217	DNAJC3	DnaJ homolog subfamily C member 3	2.9	0.000	PRDX3
P12109	COL6A1	Collagen alpha-1(VI) chain	2.9	0.002	PRDX3
P50454	SERPINH1	Serpin H1	2.8	0.000	PRDX3
P27797	CALR	Calreticulin	2.8	0.000	PRDX3
Q9BS26	ERP44	Endoplasmic reticulum resident protein 44	2.8	0.047	PRDX3
Q9Y680	FKBP7	Peptidyl-prolyl cis-trans isomerase FKBP7	2.7	0.002	PRDX3
P27824	CANX	Calnexin	2.6	0.000	PRDX3
P09972	ALDOC	Fructose-bisphosphate aldolase C	2.6	0.016	PRDX3
P14625	HSP90B1	Endoplasmin	2.4	0.000	PRDX3
D6RBV2	LMAN2	Vesicular integral-membrane protein VIP36	2.4	0.006	PRDX3
P55268	LAMB2	Laminin subunit beta-2	2.2	0.001	PRDX3
Q13257	MAD2L1	Mitotic spindle assembly checkpoint protein MAD2A	2.1	0.002	PRDX3
E9PFZ2	CP	Ceruloplasmin	2.1	0.012	PRDX3
Q12929	EPS8	Epidermal growth factor receptor kinase substrate 8	2.0	0.008	PRDX3
P07942	LAMB1	Laminin subunit beta-1	2.0	0.013	PRDX3
Q13162	PRDX4	Peroxiredoxin-4	11.0	0.000	PRDX3
Q8IXB1	DNAJC10	DnaJ homolog subfamily C member 10	1.9	0.000	PRDX3
Q9BZQ6	EDEM3	ER degradation-enhancing alpha-mannosidase-like protein 3	1.9	0.012	PRDX3
Q9UKN8	GTF3C4	General transcription factor 3C polypeptide 4	1.9	0.015	PRDX3
P11717	IGF2R	Cation-independent mannose-6-phosphate receptor	1.9	0.018	PRDX3
P32119	PRDX2	Peroxiredoxin-2	1.8	0.002	PRDX3
Q8NBJ5	COLGALT1	Procollagen galactosyltransferase 1	1.7	0.031	PRDX3
Q16576	RBBP7	Histone-binding protein RBBP7	1.6	0.002	PRDX3
Q13151	HNRNPA0	Heterogeneous nuclear ribonucleoprotein A0	1.6	0.035	PRDX3
Q8TEM1	NUP210	Nuclear pore membrane glycoprotein 210	1.5	0.001	PRDX3
P13674	P4HA1	Prolyl 4-hydroxylase subunit alpha-1	1.5	0.002	PRDX3
A0A2R8Y811	RPS14	40S ribosomal protein S14	1.4	0.000	PRDX3
Q9UBS4	DNAJB11	DnaJ homolog subfamily B member 11	1.4	0.000	PRDX3
P62753	RPS6	40S ribosomal protein S6	1.4	0.003	PRDX3
P06748	NPM1	Nucleophosmin	1.4	0.017	PRDX3
Q14696	MESD	LRP chaperone MESD	1.4	0.045	PRDX3
Q14697	GANAB	Neutral alpha-glucosidase AB	1.3	0.003	PRDX3
P62280	RPS11	40S ribosomal protein S11	1.3	0.004	PRDX3
O00116	AGPS	Alkyldihydroxyacetonephosphate synthase, peroxisomal	1.3	0.006	PRDX3
P18077	RPL35A	60S ribosomal protein L35a	1.2	0.003	PRDX3
P49257	LMAN1	Protein ERGIC-53	1.2	0.010	PRDX3
Q15424	SAFB	Scaffold attachment factor B1	1.2	0.014	PRDX3
O43852	CALU	Calumenin	1.2	0.014	PRDX3
O75821	EIF3G	Eukaryotic translation initiation factor 3 subunit G	1.1	0.003	PRDX3
Q9H0H5	RACGAP1	Rac GTPase-activating protein 1	1.1	0.008	PRDX3
Q9HCN8	SDF2L1	Stromal cell-derived factor 2-like protein 1	1.1	0.011	PRDX3
Q15020	SART3	Squamous cell carcinoma antigen recognized by T-cells 3	1.1	0.015	PRDX3
P18124	RPL7	60S ribosomal protein L7	1.1	0.032	PRDX3
P13667	PDIA4	Protein disulfide-isomerase A4	6.0	0.000	PRDX5
P30048	PRDX3	Thioredoxin-dependent peroxide reductase, mitochondrial	5.7	0.000	PRDX5
Q15084	PDIA6	Protein disulfide-isomerase A6	5.0	0.000	PRDX5
A0A087X054	HYOU1	Hypoxia up-regulated protein 1	4.2	0.000	PRDX5
Q12797	ASPH	Aspartyl/asparaginyl beta-hydroxylase	3.9	0.000	PRDX5
Q9NYU2	UGGT1	UDP-glucose:glycoprotein glucosyltransferase 1	3.8	0.000	PRDX5
O00469	PLOD2	Procollagen-lysine,2-oxoglutarate 5-dioxygenase 2	3.7	0.000	PRDX5
P07237	P4HB	Protein disulfide-isomerase	3.5	0.000	PRDX5
P11047	LAMC1	Laminin subunit gamma-1	3.4	0.000	PRDX5
P50454	SERPINH1	Serpin H1	3.4	0.000	PRDX5
P11021	HSPA5	Endoplasmic reticulum chaperone BiP	3.2	0.000	PRDX5
Q13217	DNAJC3	DnaJ homolog subfamily C member 3	2.9	0.000	PRDX5
Q8TEM1	NUP210	Nuclear pore membrane glycoprotein 210	2.9	0.000	PRDX5
P30101	PDIA3	Protein disulfide-isomerase A3	2.8	0.000	PRDX5
Q9BS26	ERP44	Endoplasmic reticulum resident protein 44	2.8	0.036	PRDX5
Q9BZQ6	EDEM3	ER degradation-enhancing alpha-mannosidase-like protein 3	2.7	0.001	PRDX5
D6RBV2	LMAN2	Vesicular integral-membrane protein VIP36	2.7	0.002	PRDX5
P12109	COL6A1	Collagen alpha-1(VI) chain	2.6	0.001	PRDX5
P49257	LMAN1	Protein ERGIC-53	2.4	0.001	PRDX5
Q9Y680	FKBP7	Peptidyl-prolyl cis-trans isomerase FKBP7	2.4	0.002	PRDX5
P27824	CANX	Calnexin	2.2	0.000	PRDX5
P55268	LAMB2	Laminin subunit beta-2	2.2	0.002	PRDX5
P09110	ACAA1	3-ketoacyl-CoA thiolase, peroxisomal	2.1	0.001	PRDX5
Q96AC1	FERMT2	Fermitin family homolog 2	2.1	0.011	PRDX5
Q9BYN0	SRXN1	Sulfiredoxin-1	2.1	0.016	PRDX5
P22307	SCP2	Non-specific lipid-transfer protein	2.0	0.000	PRDX5
Q7Z406	MYH14	Myosin-14	2.0	0.001	PRDX5
Q13162	PRDX4	Peroxiredoxin-4	10.2	0.000	PRDX5
P27797	CALR	Calreticulin	1.9	0.000	PRDX5
Q8IXB1	DNAJC10	DnaJ homolog subfamily C member 10	1.9	0.001	PRDX5
Q13257	MAD2L1	Mitotic spindle assembly checkpoint protein MAD2A	1.9	0.001	PRDX5
P19105	MYL12A	Myosin regulatory light chain 12A	1.9	0.011	PRDX5
Q15149	PLEC	Plectin	1.9	0.025	PRDX5
P05783	KRT18	Keratin, type I cytoskeletal 18	1.8	0.003	PRDX5
E9PFZ2	CP	Ceruloplasmin	1.8	0.022	PRDX5
Q9Y224	RTRAF	RNA transcription, translation and transport factor protein	1.8	0.027	PRDX5
P55084	HADHB	Trifunctional enzyme subunit beta, mitochondrial	1.8	0.034	PRDX5
B8ZZC5	GLS	Glutaminase	1.7	0.000	PRDX5
P14625	HSP90B1	Endoplasmin	1.7	0.000	PRDX5
Q9BW92	TARS2	Threonine--tRNA ligase, mitochondrial	1.7	0.003	PRDX5
P31327	CPS1	Carbamoyl-phosphate synthase [ammonia], mitochondrial	1.7	0.005	PRDX5
G3V5X4	SYNE2	Nesprin-2	1.7	0.022	PRDX5
Q01082	SPTBN1	Spectrin beta chain, non-erythrocytic 1	1.7	0.025	PRDX5
Q9H0H5	RACGAP1	Rac GTPase-activating protein 1	1.6	0.000	PRDX5
Q4VCS5	AMOT	Angiomotin	1.6	0.012	PRDX5
Q96KM6	ZNF512B	Zinc finger protein 512B	1.6	0.012	PRDX5
P07942	LAMB1	Laminin subunit beta-1	1.6	0.025	PRDX5
P42704	LRPPRC	Leucine-rich PPR motif-containing protein, mitochondrial	1.6	0.025	PRDX5
P42345	MTOR	Serine/threonine-protein kinase mTOR	1.6	0.026	PRDX5
Q13813	SPTAN1	Spectrin alpha chain, non-erythrocytic 1	1.6	0.034	PRDX5
P46013	MKI67	Proliferation marker protein Ki-67	1.6	0.035	PRDX5
P43897	TSFM	Elongation factor Ts, mitochondrial	1.5	0.000	PRDX5
A0A2R8YDQ9	SUCLA2	Succinate--CoA ligase [ADP-forming] subunit beta, mitochondrial	1.5	0.000	PRDX5
Q9NY93	DDX56	Probable ATP-dependent RNA helicase DDX56	1.5	0.000	PRDX5
Q5H928	HSD17B10	3-hydroxyacyl-CoA dehydrogenase type-2	1.5	0.000	PRDX5
Q02241	KIF23	Kinesin-like protein KIF23	1.5	0.001	PRDX5
P53597	SUCLG1	Succinate--CoA ligase [ADP/GDP-forming] subunit alpha, mitochondrial	1.5	0.004	PRDX5
Q9P0M6	MACROH2A2	Core histone macro-H2A.2	1.5	0.005	PRDX5
Q5JRX3	PITRM1	Presequence protease, mitochondrial	1.5	0.007	PRDX5
Q6P2E9	EDC4	Enhancer of mRNA-decapping protein 4	1.5	0.008	PRDX5
Q5VW32	BROX	BRO1 domain-containing protein BROX	1.5	0.040	PRDX5
P53007	SLC25A1	Tricarboxylate transport protein, mitochondrial	1.5	0.040	PRDX5
Q15477	SKIV2L	Helicase SKI2W	1.5	0.044	PRDX5
P27144	AK4	Adenylate kinase 4, mitochondrial	1.4	0.000	PRDX5
P98175	RBM10	RNA-binding protein 10	1.4	0.000	PRDX5
A0A2R8YD50	HSD17B4	Peroxisomal multifunctional enzyme type 2	1.4	0.000	PRDX5
P42765	ACAA2	3-ketoacyl-CoA thiolase, mitochondrial	1.4	0.001	PRDX5
Q8BSH4	TACO1	Translational activator of cytochrome c oxidase 1	1.4	0.002	PRDX5
Q02252	ALDH6A1	Methylmalonate-semialdehyde dehydrogenase [acylating], mitochondrial	1.4	0.002	PRDX5
Q5JTZ9	AARS2	Alanine--tRNA ligase, mitochondrial	1.4	0.002	PRDX5
Q8IYI6	EXOC8	Exocyst complex component 8	1.4	0.003	PRDX5
Q12849	GRSF1	G-rich sequence factor 1	1.4	0.003	PRDX5
P19338	NCL	Nucleolin	1.4	0.005	PRDX5
Q92616	GCN1	eIF-2-alpha kinase activator GCN1	1.4	0.013	PRDX5
Q15061	WDR43	WD repeat-containing protein 43	1.4	0.019	PRDX5
Q7L2E3	DHX30	ATP-dependent RNA helicase DHX30	1.4	0.027	PRDX5
O75367	MACROH2A1	Core histone macro-H2A.1	1.4	0.034	PRDX5
Q16762	TST	Thiosulfate sulfurtransferase	1.4	0.040	PRDX5
P11388	TOP2A	DNA topoisomerase 2-alpha	1.4	0.042	PRDX5
Q9Y2Q3	GSTK1	Glutathione S-transferase kappa 1	1.3	0.000	PRDX5
A0A0A0MRM8	MYO6	Unconventional myosin-6	1.3	0.003	PRDX5
P13674	P4HA1	Prolyl 4-hydroxylase subunit alpha-1	1.3	0.006	PRDX5
Q9NW13	RBM28	RNA-binding protein 28	1.3	0.012	PRDX5
P82930	MRPS34	28S ribosomal protein S34, mitochondrial	1.3	0.025	PRDX5
Q13356	PPIL2	RING-type E3 ubiquitin-protein ligase PPIL2	1.3	0.030	PRDX5
P04792	HSPB1	Heat shock protein beta-1	1.2	0.000	PRDX5
P30084	ECHS1	Enoyl-CoA hydratase, mitochondrial	1.2	0.001	PRDX5
P18077	RPL35A	60S ribosomal protein L35a	1.2	0.002	PRDX5
G5EA31	SEC24C	Protein transport protein Sec24C	1.2	0.003	PRDX5
Q9Y2R9	MRPS7	28S ribosomal protein S7, mitochondrial	1.2	0.004	PRDX5
P35580	MYH10	Myosin-10	1.2	0.004	PRDX5
O94925	GLS	Glutaminase kidney isoform, mitochondrial	1.2	0.017	PRDX5
Q13509	TUBB3	Tubulin beta-3 chain	1.2	0.023	PRDX5
P33992	MCM5	DNA replication licensing factor MCM5	1.2	0.024	PRDX5
P07196	NEFL	Neurofilament light polypeptide	1.2	0.035	PRDX5
Q9HCD5	NCOA5	Nuclear receptor coactivator 5	1.2	0.036	PRDX5
Q9H3U1	UNC45A	Protein unc-45 homolog A	1.2	0.044	PRDX5
Q9UBS4	DNAJB11	DnaJ homolog subfamily B member 11	1.1	0.001	PRDX5
Q9UBQ7	GRHPR	Glyoxylate reductase/hydroxypyruvate reductase	1.1	0.002	PRDX5
Q96KR1	ZFR	Zinc finger RNA-binding protein	1.1	0.003	PRDX5
O15381	NVL	Nuclear valosin-containing protein-like	1.1	0.004	PRDX5
P31942	HNRNPH3	Heterogeneous nuclear ribonucleoprotein H3	1.1	0.005	PRDX5
Q13085	ACACA	Acetyl-CoA carboxylase 1	1.1	0.005	PRDX5
Q9GZT3	SLIRP	SRA stem-loop-interacting RNA-binding protein, mitochondrial	1.1	0.011	PRDX5
P18583	SON	Protein SON	1.1	0.016	PRDX5
A0A0G2JNZ2	SCRIB	Protein scribble homolog	1.1	0.016	PRDX5
A0A0U1RRM6	ENAH	Protein enabled homolog	1.1	0.016	PRDX5
E9PEJ4	DLAT	Acetyltransferase component of pyruvate dehydrogenase complex	1.1	0.016	PRDX5
G5E9W7	MRPS22	28S ribosomal protein S22, mitochondrial	1.1	0.019	PRDX5
O43615	TIMM44	Mitochondrial import inner membrane translocase subunit TIM44	1.1	0.021	PRDX5
Q05655	PRKCD	Protein kinase C delta type	1.1	0.023	PRDX5
Q14258	TRIM25	E3 ubiquitin/ISG15 ligase TRIM25	1.1	0.025	PRDX5
Q9BQ69	MACROD1	ADP-ribose glycohydrolase MACROD1	1.1	0.025	PRDX5
P25685	DNAJB1	DnaJ homolog subfamily B member 1	1.1	0.026	PRDX5
P62879	GNB2	Guanine nucleotide-binding protein G(I)/G(S)/G(T) subunit beta-2	1.1	0.028	PRDX5
O95861	BPNT1	3'(2'),5'-bisphosphate nucleotidase 1	1.1	0.031	PRDX5
Q13464	ROCK1	Rho-associated protein kinase 1	1.1	0.034	PRDX5
P50995	ANXA11	Annexin A11	1.1	0.034	PRDX5
Q5JTH9	RRP12	RRP12-like protein	1.1	0.035	PRDX5
P51570	GALK1	Galactokinase	1.1	0.042	PRDX5
Q00610	CLTC	Clathrin heavy chain 1	1.1	0.046	PRDX5
Q99798	ACO2	Aconitate hydratase, mitochondrial	1.0	0.001	PRDX5
Q15555	MAPRE2	Microtubule-associated protein RP/EB family member 2	1.0	0.002	PRDX5
P78406	RAE1	mRNA export factor	1.0	0.002	PRDX5
P40429	RPL13A	60S ribosomal protein L13a	1.0	0.005	PRDX5
O60701	UGDH	UDP-glucose 6-dehydrogenase	1.0	0.008	PRDX5
Q09666	AHNAK	Neuroblast differentiation-associated protein AHNAK	1.0	0.014	PRDX5
O75439	PMPCB	Mitochondrial-processing peptidase subunit beta	1.0	0.016	PRDX5
P55795	HNRNPH2	Heterogeneous nuclear ribonucleoprotein H2	1.0	0.017	PRDX5
Q15042	RAB3GAP1	Rab3 GTPase-activating protein catalytic subunit	1.0	0.023	PRDX5
Q16352	INA	Alpha-internexin	1.0	0.032	PRDX5
Q8WYP5	AHCTF1	Protein ELYS	1.0	0.036	PRDX5
P52701	MSH6	DNA mismatch repair protein Msh6	1.0	0.040	PRDX5
P48735	IDH2	Isocitrate dehydrogenase [NADP], mitochondrial	1.0	0.041	PRDX5
P60174	TPI1	Triosephosphate isomerase	1.0	0.043	PRDX5
**PRDX5 -specific interactors**					
O94776	MTA2	Metastasis-associated protein MTA2	7.0	0.000	PRDX1
P49366	DHPS	Deoxyhypusine synthase	6.6	0.000	PRDX1
Q92769	HDAC2	Histone deacetylase 2	5.0	0.000	PRDX1
Q5QNY5	PEX19	Peroxin-19	4.9	0.000	PRDX1
P61289	PSME3	Proteasome activator complex subunit 3	4.6	0.001	PRDX1
Q09028	RBBP4	Histone-binding protein RBBP4	4.5	0.000	PRDX1
P05386	RPLP1	60S acidic ribosomal protein P1	4.1	0.000	PRDX1
P62805	H4C1	Histone H4	4.1	0.000	PRDX1
P63151	PPP2R2A	Serine/threonine-protein phosphatase 2A 55 kDa regulatory subunit B alpha isoform	3.9	0.000	PRDX1
O14744	PRMT5	Protein arginine N-methyltransferase 5	3.8	0.000	PRDX1
Q16576	RBBP7	Histone-binding protein RBBP7	3.7	0.000	PRDX1
P30048	PRDX3	Thioredoxin-dependent peroxide reductase, mitochondrial	3.6	0.000	PRDX1
P41250	GARS1	Glycine--tRNA ligase	3.6	0.000	PRDX1
P02790	HPX	Hemopexin	3.6	0.001	PRDX1
O00165	HAX1	HCLS1-associated protein X-1	3.5	0.000	PRDX1
Q9NQP4	PFDN4	Prefoldin subunit 4	3.5	0.000	PRDX1
H0YEN2	PPP6R3	Serine/threonine-protein phosphatase 6 regulatory subunit 3	3.5	0.000	PRDX1
H0Y4R1	IMPDH2	Inosine-5'-monophosphate dehydrogenase 2	3.5	0.000	PRDX1
P62258	YWHAE	14-3-3 protein epsilon	3.5	0.000	PRDX1
O60506	SYNCRIP	Heterogeneous nuclear ribonucleoprotein Q	3.4	0.000	PRDX1
Q13547	HDAC1	Histone deacetylase 1	3.3	0.000	PRDX1
P27797	CALR	Calreticulin	3.3	0.000	PRDX1
P10599	TXN	Thioredoxin	3.2	0.000	PRDX1
O76094	SRP72	Signal recognition particle subunit SRP72	3.2	0.000	PRDX1
P05387	RPLP2	60S acidic ribosomal protein P2	3.2	0.000	PRDX1
Q9BQA1	WDR77	Methylosome protein 50	3.2	0.002	PRDX1
Q9GZU8	PSME3IP1	PSME3-interacting protein	3.1	0.000	PRDX1
P48147	PREP	Prolyl endopeptidase	3.1	0.000	PRDX1
P67936	TPM4	Tropomyosin alpha-4 chain	3.1	0.000	PRDX1
P22234	PAICS	Multifunctional protein ADE2 [Includes: Phosphoribosylaminoimidazole-succinocarboxamide synthase	3.1	0.000	PRDX1
Q9BSJ8	ESYT1	Extended synaptotagmin-1	3.0	0.000	PRDX1
Q14257	RCN2	Reticulocalbin-2	3.0	0.000	PRDX1
Q6DKK2	TTC19	Tetratricopeptide repeat protein 19, mitochondrial	3.0	0.001	PRDX1
Q96BR5	COA7	Cytochrome c oxidase assembly factor 7	3.0	0.001	PRDX1
P54619	PRKAG1	5'-AMP-activated protein kinase subunit gamma-1	3.0	0.001	PRDX1
A0A024R442	A0A024R442	>tr|A0A024R442|A0A024R442_HUMAN Aspartyl aminopeptidase, isoform CRA_b OS=Homo sapiens OX=9606 GN=DNPEP PE=1 SV=1;>sp|Q9ULA0|DNPEP_HUMAN Aspartyl aminopeptidase OS=Homo sapiens OX=9606 GN=DNPEP PE=1 SV=1;>tr|E7ETB3|E7ETB3_HUMAN Aspartyl aminopeptidase, iso	2.9	0.000	PRDX1
Q8N806	UBR7	Putative E3 ubiquitin-protein ligase UBR7	2.9	0.000	PRDX1
P20839	IMPDH1	Inosine-5'-monophosphate dehydrogenase 1	2.9	0.000	PRDX1
Q96EW2	HSPBAP1	HSPB1-associated protein 1	2.9	0.000	PRDX1
P49321	NASP	Nuclear autoantigenic sperm protein	2.9	0.000	PRDX1
P53985	SLC16A1	Monocarboxylate transporter 1	2.9	0.000	PRDX1
A0A1C7CYX9	DPYSL2	Dihydropyrimidinase-related protein 2	2.8	0.000	PRDX1
Q15008	PSMD6	26S proteasome non-ATPase regulatory subunit 6	2.8	0.000	PRDX1
E7ESY4	MTA1	Metastasis-associated protein MTA1	2.8	0.000	PRDX1
P23526	AHCY	Adenosylhomocysteinase	2.8	0.000	PRDX1
Q96DB5	RMDN1	Regulator of microtubule dynamics protein 1	2.8	0.001	PRDX1
Q96RS6	NUDCD1	NudC domain-containing protein 1	2.7	0.000	PRDX1
Q9UEY8	ADD3	Gamma-adducin	2.7	0.000	PRDX1
P22314	UBA1	Ubiquitin-like modifier-activating enzyme 1	2.7	0.000	PRDX1
O43143	DHX15	Pre-mRNA-splicing factor ATP-dependent RNA helicase DHX15	2.6	0.000	PRDX1
Q9UHV9	PFDN2	Prefoldin subunit 2	2.6	0.000	PRDX1
Q5T4U8	RABGGTB	Geranylgeranyl transferase type-2 subunit beta	2.6	0.000	PRDX1
P78417	GSTO1	Glutathione S-transferase omega-1	2.6	0.000	PRDX1
Q15276	RABEP1	Rab GTPase-binding effector protein 1	2.6	0.000	PRDX1
P31153	MAT2A	S-adenosylmethionine synthase isoform type-2	2.6	0.000	PRDX1
O75821	EIF3G	Eukaryotic translation initiation factor 3 subunit G	2.6	0.000	PRDX1
P23528	CFL1	Cofilin-1	2.6	0.000	PRDX1
A0A2U3TZY2	CLPB	Caseinolytic peptidase B protein homolog	2.6	0.001	PRDX1
P54646	PRKAA2	5'-AMP-activated protein kinase catalytic subunit alpha-2	2.5	0.000	PRDX1
Q9BPU6	DPYSL5	Dihydropyrimidinase-related protein 5	2.5	0.000	PRDX1
Q13200	PSMD2	26S proteasome non-ATPase regulatory subunit 2	2.5	0.000	PRDX1
B9A018	USP39	U4/U6.U5 tri-snRNP-associated protein 2	2.4	0.000	PRDX1
P23588	EIF4B	Eukaryotic translation initiation factor 4B	2.4	0.000	PRDX1
Q16531	DDB1	DNA damage-binding protein 1	2.4	0.000	PRDX1
P00492	HPRT1	Hypoxanthine-guanine phosphoribosyltransferase	2.4	0.000	PRDX1
Q15459	SF3A1	Splicing factor 3A subunit 1	2.4	0.038	PRDX1
J3QQZ9	PNPO	Pyridoxal 5'-phosphate synthase	2.3	0.000	PRDX1
P78371	CCT2	T-complex protein 1 subunit beta	2.3	0.000	PRDX1
A0A0A0MR02	VDAC2	Outer mitochondrial membrane protein porin 2	2.3	0.000	PRDX1
O00268	TAF4	Transcription initiation factor TFIID subunit 4	2.3	0.000	PRDX1
P35637	FUS	RNA-binding protein FUS	2.3	0.001	PRDX1
P38159	RBMX	RNA-binding motif protein, X chromosome	2.3	0.005	PRDX1
Q32MZ4	LRRFIP1	Leucine-rich repeat flightless-interacting protein 1	2.2	0.000	PRDX1
P27348	YWHAQ	14-3-3 protein theta	2.2	0.000	PRDX1
Q9C0C9	UBE2O	(E3-independent) E2 ubiquitin-conjugating enzyme	2.2	0.000	PRDX1
Q13177	PAK2	Serine/threonine-protein kinase PAK 2	2.2	0.000	PRDX1
Q9Y3F4	STRAP	Serine-threonine kinase receptor-associated protein	2.2	0.000	PRDX1
Q9ULC3	RAB23	Ras-related protein Rab-23	2.2	0.001	PRDX1
P42704	LRPPRC	Leucine-rich PPR motif-containing protein, mitochondrial	2.2	0.001	PRDX1
P12955	PEPD	Xaa-Pro dipeptidase	2.2	0.004	PRDX1
P43304	GPD2	Glycerol-3-phosphate dehydrogenase, mitochondrial	2.2	0.004	PRDX1
O00232	PSMD12	26S proteasome non-ATPase regulatory subunit 12	2.1	0.000	PRDX1
Q96RG2	PASK	PAS domain-containing serine/threonine-protein kinase	2.1	0.000	PRDX1
P15374	UCHL3	Ubiquitin carboxyl-terminal hydrolase isozyme L3	2.1	0.000	PRDX1
Q9H8Y8	GORASP2	Golgi reassembly-stacking protein 2	2.1	0.000	PRDX1
Q96HC4	PDLIM5	PDZ and LIM domain protein 5	2.1	0.000	PRDX1
P40429	RPL13A	60S ribosomal protein L13a	2.1	0.001	PRDX1
P45973	CBX5	Chromobox protein homolog 5	2.1	0.001	PRDX1
A0A087WT45	GRIPAP1	GRIP1-associated protein 1	2.1	0.002	PRDX1
O00231	PSMD11	26S proteasome non-ATPase regulatory subunit 11	2.0	0.000	PRDX1
P62979	RPS27A	Ubiquitin-40S ribosomal protein S27a	2.0	0.000	PRDX1
P36957	DLST	Dihydrolipoyllysine-residue succinyltransferase component of 2-oxoglutarate dehydrogenase complex, mitochondrial	2.0	0.000	PRDX1
P31689	DNAJA1	DnaJ homolog subfamily A member 1	2.0	0.000	PRDX1
Q02750	MAP2K1	Dual specificity mitogen-activated protein kinase kinase 1	2.0	0.000	PRDX1
C9J4Z3	RPL37A	60S ribosomal protein L37a	2.0	0.000	PRDX1
Q93009	USP7	Ubiquitin carboxyl-terminal hydrolase 7	2.0	0.000	PRDX1
P61221	ABCE1	ATP-binding cassette sub-family E member 1	2.0	0.001	PRDX1
E5KLJ9	OPA1	Dynamin-like 120 kDa protein, form S1	2.0	0.003	PRDX1
E9PGT1	TSN	Component 3 of promoter of RISC	2.0	0.004	PRDX1
H7C3C4	SLC4A7	Anion exchange protein	2.0	0.004	PRDX1
P52788	SMS	Spermine synthase	2.0	0.007	PRDX1
H0YKU1	TMOD3	Tropomodulin-3	2.0	0.008	PRDX1
P30044	PRDX5	Peroxiredoxin-5, mitochondrial	10.1	0.000	PRDX1
O43852	CALU	Calumenin	1.9	0.000	PRDX1
Q9UBF2	COPG2	Coatomer subunit gamma-2	1.9	0.000	PRDX1
O95433	AHSA1	Activator of 90 kDa heat shock protein ATPase homolog 1	1.9	0.000	PRDX1
P34932	HSPA4	Heat shock 70 kDa protein 4	1.9	0.000	PRDX1
Q4J6C6	PREPL	Prolyl endopeptidase-like	1.9	0.001	PRDX1
Q13564	NAE1	NEDD8-activating enzyme E1 regulatory subunit	1.9	0.001	PRDX1
P62314	SNRPD1	Small nuclear ribonucleoprotein Sm D1	1.9	0.001	PRDX1
Q13185	CBX3	Chromobox protein homolog 3	1.9	0.002	PRDX1
Q9Y478	PRKAB1	5'-AMP-activated protein kinase subunit beta-1	1.9	0.004	PRDX1
P30041	PRDX6	Peroxiredoxin-6	1.9	0.005	PRDX1
Q96KB5	PBK	Lymphokine-activated killer T-cell-originated protein kinase	1.9	0.010	PRDX1
J3KPG2	TPT1	Translationally-controlled tumor protein	1.9	0.017	PRDX1
Q96A49	SYAP1	Synapse-associated protein 1	1.9	0.023	PRDX1
P61981	YWHAG	14-3-3 protein gamma	1.8	0.000	PRDX1
P06730	EIF4E	Eukaryotic translation initiation factor 4E	1.8	0.000	PRDX1
Q99460	PSMD1	26S proteasome non-ATPase regulatory subunit 1	1.8	0.000	PRDX1
E9PLK3	NPEPPS	Aminopeptidase	1.8	0.000	PRDX1
Q9BRP1	PDCD2L	Programmed cell death protein 2-like	1.8	0.000	PRDX1
Q15393	SF3B3	Splicing factor 3B subunit 3	1.8	0.001	PRDX1
Q8WXD5	GEMIN6	Gem-associated protein 6	1.8	0.001	PRDX1
Q9UBS4	DNAJB11	DnaJ homolog subfamily B member 11	1.8	0.002	PRDX1
Q8N3X1	FNBP4	Formin-binding protein 4	1.8	0.006	PRDX1
O00483	NDUFA4	Cytochrome c oxidase subunit NDUFA4	1.8	0.009	PRDX1
Q15293	RCN1	Reticulocalbin-1	1.8	0.009	PRDX1
P12814	ACTN1	Alpha-actinin-1	1.8	0.012	PRDX1
Q9NVP1	DDX18	ATP-dependent RNA helicase DDX18	1.8	0.013	PRDX1
P62333	PSMC6	26S proteasome regulatory subunit 10B	1.7	0.000	PRDX1
P50502	ST13	Hsc70-interacting protein	1.7	0.001	PRDX1
P27824	CANX	Calnexin	1.7	0.004	PRDX1
P52888	THOP1	Thimet oligopeptidase	1.7	0.005	PRDX1
P46821	MAP1B	Microtubule-associated protein 1B	1.7	0.007	PRDX1
X6RM00	ERC1	ELKS/Rab6-interacting/CAST family member 1	1.7	0.009	PRDX1
Q9UHY1	NRBP1	Nuclear receptor-binding protein	1.7	0.009	PRDX1
O96019	ACTL6A	Actin-like protein 6A	1.7	0.010	PRDX1
D6RA00	ENOPH1	Enolase-phosphatase E1	1.7	0.012	PRDX1
B4DHR0	RABEP2	Rab GTPase-binding effector protein 2	1.7	0.013	PRDX1
Q53GQ0	HSD17B12	Very-long-chain 3-oxoacyl-CoA reductase	1.7	0.032	PRDX1
Q96CS3	FAF2	FAS-associated factor 2	1.6	0.000	PRDX1
Q9UJS0	SLC25A13	Calcium-binding mitochondrial carrier protein Aralar2	1.6	0.000	PRDX1
O00487	PSMD14	26S proteasome non-ATPase regulatory subunit 14	1.6	0.000	PRDX1
E9PIF2	DDX10	RNA helicase	1.6	0.000	PRDX1
P52565	ARHGDIA	Rho GDP-dissociation inhibitor 1	1.6	0.000	PRDX1
P61923	COPZ1	Coatomer subunit zeta-1	1.6	0.000	PRDX1
P98175	RBM10	RNA-binding protein 10	1.6	0.000	PRDX1
Q9UQ80	PA2G4	Proliferation-associated protein 2G4	1.6	0.000	PRDX1
Q8NE71	ABCF1	ATP-binding cassette sub-family F member 1	1.6	0.000	PRDX1
P34949	MPI	Mannose-6-phosphate isomerase	1.6	0.000	PRDX1
P26196	DDX6	Probable ATP-dependent RNA helicase DDX6	1.6	0.000	PRDX1
O43164	PJA2	E3 ubiquitin-protein ligase Praja-2	1.6	0.001	PRDX1
O15212	PFDN6	Prefoldin subunit 6	1.6	0.001	PRDX1
B1AK87	CAPZB	F-actin-capping protein subunit beta	1.6	0.002	PRDX1
O75688	PPM1B	Protein phosphatase 1B	1.6	0.004	PRDX1
P29218	IMPA1	Inositol monophosphatase 1	1.6	0.014	PRDX1
A0A087WXS7	A0A087WXS7	>tr|A0A087WXS7|A0A087WXS7_HUMAN ATPase ASNA1 OS=Homo sapiens OX=9606 GN=ASNA1 PE=1 SV=1;>sp|O43681|ASNA_HUMAN ATPase ASNA1 OS=Homo sapiens OX=9606 GN=ASNA1 PE=1 SV=2;>tr|K7ERW9|K7ERW9_HUMAN Arsenical pump-driving ATPase (Fragment) OS=Homo sapiens OX=9606 G	1.6	0.030	PRDX1
O15372	EIF3H	Eukaryotic translation initiation factor 3 subunit H	1.5	0.000	PRDX1
Q9UIG0	BAZ1B	Tyrosine-protein kinase BAZ1B	1.5	0.000	PRDX1
K7ERF1	EIF3K	Eukaryotic translation initiation factor 3 subunit K	1.5	0.000	PRDX1
Q96D09	GPRASP2	G-protein coupled receptor-associated sorting protein 2	1.5	0.000	PRDX1
P06737	PYGL	Glycogen phosphorylase, liver form	1.5	0.000	PRDX1
Q15029	EFTUD2	116 kDa U5 small nuclear ribonucleoprotein component	1.5	0.000	PRDX1
P63104	YWHAZ	14-3-3 protein zeta/delta	1.5	0.000	PRDX1
E5RHG8	ELOC	Elongin-C	1.5	0.000	PRDX1
P30101	PDIA3	Protein disulfide-isomerase A3	1.5	0.001	PRDX1
O75832	PSMD10	26S proteasome non-ATPase regulatory subunit 10	1.5	0.001	PRDX1
O00273	DFFA	DNA fragmentation factor subunit alpha	1.5	0.002	PRDX1
O43747	AP1G1	AP-1 complex subunit gamma-1	1.5	0.002	PRDX1
Q6IN85	PPP4R3A	Serine/threonine-protein phosphatase 4 regulatory subunit 3A	1.5	0.005	PRDX1
Q96T23	RSF1	Remodeling and spacing factor 1	1.5	0.010	PRDX1
H7C128	BRD8	Bromodomain-containing protein 8	1.5	0.012	PRDX1
Q9HA64	FN3KRP	Ketosamine-3-kinase	1.5	0.017	PRDX1
Q9P287	BCCIP	BRCA2 and CDKN1A-interacting protein	1.5	0.018	PRDX1
Q13033	STRN3	Striatin-3	1.5	0.022	PRDX1
Q9NQ29	LUC7L	Putative RNA-binding protein Luc7-like 1	1.5	0.023	PRDX1
P19174	PLCG1	1-phosphatidylinositol 4,5-bisphosphate phosphodiesterase gamma-1	1.5	0.024	PRDX1
Q86TI2	DPP9	Dipeptidyl peptidase 9	1.5	0.027	PRDX1
Q9NTK5	OLA1	Obg-like ATPase 1	1.5	0.031	PRDX1
P28288	ABCD3	ATP-binding cassette sub-family D member 3	1.5	0.034	PRDX1
O15144	ARPC2	Actin-related protein 2/3 complex subunit 2	1.5	0.035	PRDX1
Q9Y277	VDAC3	Voltage-dependent anion-selective channel protein 3	1.5	0.044	PRDX1
Q9UL15	BAG5	BAG family molecular chaperone regulator 5	1.5	0.046	PRDX1
Q15691	MAPRE1	Microtubule-associated protein RP/EB family member 1	1.4	0.000	PRDX1
P15121	AKR1B1	Aldo-keto reductase family 1 member B1	1.4	0.000	PRDX1
E9PMI6	CLNS1A	Chloride channel, nucleotide sensitive 1A	1.4	0.000	PRDX1
Q92598	HSPH1	Heat shock protein 105 kDa	1.4	0.000	PRDX1
P60900	PSMA6	Proteasome subunit alpha type-6	1.4	0.000	PRDX1
Q8IWB7	WDFY1	WD repeat and FYVE domain-containing protein 1	1.4	0.001	PRDX1
H7BZM7	ZPR1	Zinc finger protein ZPR1	1.4	0.001	PRDX1
P45974	USP5	Ubiquitin carboxyl-terminal hydrolase 5	1.4	0.001	PRDX1
O43707	ACTN4	Alpha-actinin-4	1.4	0.002	PRDX1
E7EV99	ADD1	Alpha-adducin	1.4	0.002	PRDX1
Q9H0A0	NAT10	RNA cytidine acetyltransferase	1.4	0.006	PRDX1
F8W038	C17orf49	Chromosome 17 open reading frame 49	1.4	0.008	PRDX1
P42574	CASP3	Caspase-3	1.4	0.025	PRDX1
Q99598	TSNAX	Translin-associated protein X	1.4	0.029	PRDX1
Q01085	TIAL1	Nucleolysin TIAR	1.4	0.033	PRDX1
Q9NZL9	MAT2B	Methionine adenosyltransferase 2 subunit beta	1.4	0.037	PRDX1
Q9UKN8	GTF3C4	General transcription factor 3C polypeptide 4	1.4	0.045	PRDX1
Q8N999	C12orf29	Uncharacterized protein C12orf29	1.4	0.047	PRDX1
P32969	RPL9	60S ribosomal protein L9	1.3	0.000	PRDX1
Q9NSD9	FARSB	Phenylalanine--tRNA ligase beta subunit	1.3	0.000	PRDX1
Q9Y383	LUC7L2	Putative RNA-binding protein Luc7-like 2	1.3	0.000	PRDX1
P05023	ATP1A1	Sodium/potassium-transporting ATPase subunit alpha-1	1.3	0.000	PRDX1
A6NKB8	RNPEP	Aminopeptidase B	1.3	0.000	PRDX1
P61247	RPS3A	40S ribosomal protein S3a	1.3	0.000	PRDX1
P62424	RPL7A	60S ribosomal protein L7a	1.3	0.000	PRDX1
P60953	CDC42	Cell division control protein 42 homolog	1.3	0.001	PRDX1
Q68E01	INTS3	Integrator complex subunit 3	1.3	0.001	PRDX1
F8VVL1	DENR	Density-regulated protein	1.3	0.002	PRDX1
Q92688	ANP32B	Acidic leucine-rich nuclear phosphoprotein 32 family member B	1.3	0.003	PRDX1
P18077	RPL35A	60S ribosomal protein L35a	1.3	0.003	PRDX1
O00186	STXBP3	Syntaxin-binding protein 3	1.3	0.005	PRDX1
P16615	ATP2A2	Sarcoplasmic/endoplasmic reticulum calcium ATPase 2	1.3	0.005	PRDX1
P08133	ANXA6	Annexin A6	1.3	0.005	PRDX1
Q15291	RBBP5	Retinoblastoma-binding protein 5	1.3	0.005	PRDX1
P49750	YLPM1	YLP motif-containing protein 1	1.3	0.005	PRDX1
P55769	SNU13	NHP2-like protein 1	1.3	0.006	PRDX1
O75381	PEX14	Peroxisomal membrane protein PEX14	1.3	0.011	PRDX1
P41252	IARS1	Isoleucine--tRNA ligase, cytoplasmic	1.3	0.018	PRDX1
P08758	ANXA5	Annexin A5	1.3	0.022	PRDX1
A0A0D9SG77	UBE3A	Ubiquitin-protein ligase E3A	1.3	0.039	PRDX1
E9PJN0	ACOT8	Acyl-coenzyme A thioesterase 8	1.3	0.040	PRDX1
P63244	RACK1	Receptor of activated protein C kinase 1	1.3	0.045	PRDX1
Q9NYB0	TERF2IP	Telomeric repeat-binding factor 2-interacting protein 1	1.3	0.045	PRDX1
A0A3B3ISV3	COL4A1	Collagen alpha-1(IV) chain	1.3	0.049	PRDX1
Q13131	PRKAA1	5'-AMP-activated protein kinase catalytic subunit alpha-1	1.3	0.050	PRDX1
P40938	RFC3	Replication factor C subunit 3	1.2	0.000	PRDX1
O00299	CLIC1	Chloride intracellular channel protein 1	1.2	0.000	PRDX1
P00387	CYB5R3	NADH-cytochrome b5 reductase 3	1.2	0.000	PRDX1
Q9Y5V3	MAGED1	Melanoma-associated antigen D1	1.2	0.000	PRDX1
P51149	RAB7A	Ras-related protein Rab-7a	1.2	0.000	PRDX1
O60763	USO1	General vesicular transport factor p115	1.2	0.000	PRDX1
P23396	RPS3	40S ribosomal protein S3	1.2	0.000	PRDX1
C9J2Y9	POLR2B	DNA-directed RNA polymerase subunit beta	1.2	0.001	PRDX1
Q3ZCQ8	TIMM50	Mitochondrial import inner membrane translocase subunit TIM50	1.2	0.001	PRDX1
Q9H3P7	ACBD3	Golgi resident protein GCP60	1.2	0.001	PRDX1
P51665	PSMD7	26S proteasome non-ATPase regulatory subunit 7	1.2	0.001	PRDX1
C9J0I9	ZC3HC1	Nuclear-interacting partner of ALK	1.2	0.001	PRDX1
P43034	PAFAH1B1	Platelet-activating factor acetylhydrolase IB subunit beta	1.2	0.001	PRDX1
P14324	FDPS	Farnesyl pyrophosphate synthase	1.2	0.001	PRDX1
Q8ND82	ZNF280C	Zinc finger protein 280C	1.2	0.001	PRDX1
P09211	GSTP1	Glutathione S-transferase P	1.2	0.001	PRDX1
P62877	RBX1	E3 ubiquitin-protein ligase RBX1	1.2	0.002	PRDX1
Q07020	RPL18	60S ribosomal protein L18	1.2	0.002	PRDX1
P25490	YY1	Transcriptional repressor protein YY1	1.2	0.003	PRDX1
Q96H79	ZC3HAV1L	Zinc finger CCCH-type antiviral protein 1-like	1.2	0.004	PRDX1
Q9Y3Z3	SAMHD1	Deoxynucleoside triphosphate triphosphohydrolase SAMHD1	1.2	0.005	PRDX1
Q01831	XPC	DNA repair protein complementing XP-C cells	1.2	0.005	PRDX1
P55884	EIF3B	Eukaryotic translation initiation factor 3 subunit B	1.2	0.005	PRDX1
Q9UI12	ATP6V1H	V-type proton ATPase subunit H	1.2	0.006	PRDX1
Q9Y5J1	UTP18	U3 small nucleolar RNA-associated protein 18 homolog	1.2	0.006	PRDX1
Q15843	NEDD8	NEDD8	1.2	0.008	PRDX1
P56192	MARS1	Methionine--tRNA ligase, cytoplasmic	1.2	0.009	PRDX1
P19338	NCL	Nucleolin	1.2	0.015	PRDX1
Q9H0U4	RAB1B	Ras-related protein Rab-1B	1.2	0.017	PRDX1
Q96RL1	UIMC1	BRCA1-A complex subunit RAP80	1.2	0.030	PRDX1
P30740	SERPINB1	Leukocyte elastase inhibitor	1.2	0.033	PRDX1
Q93008	USP9X	Probable ubiquitin carboxyl-terminal hydrolase FAF-X	1.2	0.037	PRDX1
O75369	FLNB	Filamin-B	1.2	0.041	PRDX1
E7EMC7	SQSTM1	Sequestosome-1	1.2	0.046	PRDX1
Q00325	SLC25A3	Phosphate carrier protein, mitochondrial	1.1	0.000	PRDX1
P11310	ACADM	Medium-chain specific acyl-CoA dehydrogenase, mitochondrial	1.1	0.000	PRDX1
Q96CX2	KCTD12	BTB/POZ domain-containing protein KCTD12	1.1	0.000	PRDX1
J3QQW9	SUZ12	Polycomb protein SUZ12	1.1	0.001	PRDX1
O60934	NBN	Nibrin	1.1	0.002	PRDX1
O94826	TOMM70	Mitochondrial import receptor subunit TOM70	1.1	0.002	PRDX1
P45954	ACADSB	Short/branched chain specific acyl-CoA dehydrogenase, mitochondrial	1.1	0.002	PRDX1
O94992	HEXIM1	Protein HEXIM1	1.1	0.003	PRDX1
P12236	SLC25A6	ADP/ATP translocase 3	1.1	0.003	PRDX1
P42166	TMPO	Lamina-associated polypeptide 2, isoform alpha	1.1	0.004	PRDX1
P43246	MSH2	DNA mismatch repair protein Msh2	1.1	0.004	PRDX1
P53004	BLVRA	Biliverdin reductase A	1.1	0.006	PRDX1
A0A0C4DGX4	CUL1	Cullin-1	1.1	0.010	PRDX1
O95376	ARIH2	E3 ubiquitin-protein ligase ARIH2	1.1	0.014	PRDX1
Q9UL25	RAB21	Ras-related protein Rab-21	1.1	0.015	PRDX1
O60784	TOM1	Target of Myb protein 1	1.1	0.020	PRDX1
Q15054	POLD3	DNA polymerase delta subunit 3	1.1	0.020	PRDX1
Q8IZ73	RPUSD2	RNA pseudouridylate synthase domain-containing protein 2	1.1	0.031	PRDX1
Q96SB4	SRPK1	SRSF protein kinase 1	1.1	0.033	PRDX1
P16435	POR	NADPH--cytochrome P450 reductase	1.1	0.035	PRDX1
G3V529	DDX24	RNA helicase	1.1	0.044	PRDX1
P09960	LTA4H	Leukotriene A-4 hydrolase	1.0	0.000	PRDX1
Q99623	PHB2	Prohibitin-2	1.0	0.001	PRDX1
Q99613	EIF3C	Eukaryotic translation initiation factor 3 subunit C	1.0	0.002	PRDX1
Q9NQW7	XPNPEP1	Xaa-Pro aminopeptidase 1	1.0	0.005	PRDX1
P07195	LDHB	L-lactate dehydrogenase B chain	1.0	0.006	PRDX1
Q9BY42	RTF2	Replication termination factor 2	1.0	0.007	PRDX1
Q9UPN7	PPP6R1	Serine/threonine-protein phosphatase 6 regulatory subunit 1	1.0	0.008	PRDX1
Q6PI48	DARS2	Aspartate--tRNA ligase, mitochondrial	1.0	0.012	PRDX1
O00203	AP3B1	AP-3 complex subunit beta-1	1.0	0.013	PRDX1
O76003	GLRX3	Glutaredoxin-3	1.0	0.035	PRDX1
F8W1A4	AK2	Adenylate kinase 2, mitochondrial	1.0	0.043	PRDX1
P20073	ANXA7	Annexin A7	1.0	0.047	PRDX1
P49366	DHPS	Deoxyhypusine synthase	6.9	0.000	PRDX2
O94776	MTA2	Metastasis-associated protein MTA2	6.7	0.000	PRDX2
H0YEN2	PPP6R3	Serine/threonine-protein phosphatase 6 regulatory subunit 3	4.1	0.000	PRDX2
Q16576	RBBP7	Histone-binding protein RBBP7	3.8	0.000	PRDX2
P63151	PPP2R2A	Serine/threonine-protein phosphatase 2A 55 kDa regulatory subunit B alpha isoform	3.7	0.000	PRDX2
Q5QNY5	PEX19	Peroxin-19	3.7	0.000	PRDX2

H0Y4R1	IMPDH2	Inosine-5'-monophosphate dehydrogenase 2	3.7	0.000	PRDX2
P67936	TPM4	Tropomyosin alpha-4 chain	3.6	0.000	PRDX2
Q13547	HDAC1	Histone deacetylase 1	3.6	0.000	PRDX2
Q96BR5	COA7	Cytochrome c oxidase assembly factor 7	3.5	0.000	PRDX2
O00165	HAX1	HCLS1-associated protein X-1	3.4	0.000	PRDX2
Q09028	RBBP4	Histone-binding protein RBBP4	3.4	0.000	PRDX2
P61289	PSME3	Proteasome activator complex subunit 3	3.4	0.023	PRDX2
Q96EW2	HSPBAP1	HSPB1-associated protein 1	3.1	0.000	PRDX2
Q9GZU8	PSME3IP1	PSME3-interacting protein	3.0	0.000	PRDX2
A0A0A0MR02	VDAC2	Outer mitochondrial membrane protein porin 2	3.0	0.000	PRDX2
Q96RS6	NUDCD1	NudC domain-containing protein 1	2.9	0.000	PRDX2
Q9NQP4	PFDN4	Prefoldin subunit 4	2.9	0.000	PRDX2
P62258	YWHAE	14-3-3 protein epsilon	2.9	0.000	PRDX2
A0A1C7CYX9	DPYSL2	Dihydropyrimidinase-related protein 2	2.8	0.000	PRDX2
O14744	PRMT5	Protein arginine N-methyltransferase 5	2.8	0.011	PRDX2
P23526	AHCY	Adenosylhomocysteinase	2.7	0.000	PRDX2
Q6DKK2	TTC19	Tetratricopeptide repeat protein 19, mitochondrial	2.7	0.003	PRDX2
P20839	IMPDH1	Inosine-5'-monophosphate dehydrogenase 1	2.6	0.000	PRDX2
Q96DB5	RMDN1	Regulator of microtubule dynamics protein 1	2.6	0.002	PRDX2
P78417	GSTO1	Glutathione S-transferase omega-1	2.5	0.000	PRDX2
Q15276	RABEP1	Rab GTPase-binding effector protein 1	2.5	0.000	PRDX2
P41250	GARS1	Glycine--tRNA ligase	2.4	0.000	PRDX2
P22234	PAICS	Multifunctional protein ADE2 [Includes: Phosphoribosylaminoimidazole-succinocarboxamide synthase	2.4	0.000	PRDX2
P78371	CCT2	T-complex protein 1 subunit beta	2.4	0.000	PRDX2
O75688	PPM1B	Protein phosphatase 1B	2.4	0.000	PRDX2
P27348	YWHAQ	14-3-3 protein theta	2.4	0.000	PRDX2
Q92769	HDAC2	Histone deacetylase 2	2.4	0.000	PRDX2
O00268	TAF4	Transcription initiation factor TFIID subunit 4	2.3	0.000	PRDX2
P60900	PSMA6	Proteasome subunit alpha type-6	2.2	0.002	PRDX2
H7C3C4	SLC4A7	Anion exchange protein	2.2	0.003	PRDX2
H0YKU1	TMOD3	Tropomodulin-3	2.2	0.006	PRDX2
P98175	RBM10	RNA-binding protein 10	2.1	0.000	PRDX2
Q9Y478	PRKAB1	5'-AMP-activated protein kinase subunit beta-1	2.1	0.003	PRDX2
O43143	DHX15	Pre-mRNA-splicing factor ATP-dependent RNA helicase DHX15	2.1	0.004	PRDX2
A0A2U3TZY2	CLPB	Caseinolytic peptidase B protein homolog	2.1	0.004	PRDX2
P52788	SMS	Spermine synthase	2.1	0.008	PRDX2
O43852	CALU	Calumenin	2.0	0.000	PRDX2
H7BZM7	ZPR1	Zinc finger protein ZPR1	2.0	0.000	PRDX2
F8W1A4	AK2	Adenylate kinase 2, mitochondrial	2.0	0.005	PRDX2
P12814	ACTN1	Alpha-actinin-1	2.0	0.009	PRDX2
P30044	PRDX5	Peroxiredoxin-5, mitochondrial	12.4	0.000	PRDX2
Q9C0C9	UBE2O	(E3-independent) E2 ubiquitin-conjugating enzyme	1.9	0.000	PRDX2
P31946	YWHAB	14-3-3 protein beta/alpha	1.9	0.000	PRDX2
Q9BPU6	DPYSL5	Dihydropyrimidinase-related protein 5	1.9	0.000	PRDX2
P45973	CBX5	Chromobox protein homolog 5	1.9	0.000	PRDX2
P30041	PRDX6	Peroxiredoxin-6	1.9	0.012	PRDX2
P61981	YWHAG	14-3-3 protein gamma	1.8	0.000	PRDX2
Q15008	PSMD6	26S proteasome non-ATPase regulatory subunit 6	1.8	0.000	PRDX2
Q13200	PSMD2	26S proteasome non-ATPase regulatory subunit 2	1.8	0.000	PRDX2
P11142	HSPA8	Heat shock cognate 71 kDa protein	1.8	0.000	PRDX2
O43164	PJA2	E3 ubiquitin-protein ligase Praja-2	1.8	0.001	PRDX2
P54646	PRKAA2	5'-AMP-activated protein kinase catalytic subunit alpha-2	1.8	0.001	PRDX2
J3QQZ9	PNPO	Pyridoxal 5'-phosphate synthase	1.8	0.001	PRDX2
P62979	RPS27A	Ubiquitin-40S ribosomal protein S27a	1.8	0.001	PRDX2
Q14257	RCN2	Reticulocalbin-2	1.8	0.003	PRDX2
Q96RG2	PASK	PAS domain-containing serine/threonine-protein kinase	1.8	0.003	PRDX2
Q96B36	AKT1S1	Proline-rich AKT1 substrate 1	1.8	0.015	PRDX2
O00399	DCTN6	Dynactin subunit 6	1.8	0.018	PRDX2
A0A087WXS7	A0A087WXS7	>tr|A0A087WXS7|A0A087WXS7_HUMAN ATPase ASNA1 OS=Homo sapiens OX=9606 GN=ASNA1 PE=1 SV=1;>sp|O43681|ASNA_HUMAN ATPase ASNA1 OS=Homo sapiens OX=9606 GN=ASNA1 PE=1 SV=2;>tr|K7ERW9|K7ERW9_HUMAN Arsenical pump-driving ATPase (Fragment) OS=Homo sapiens OX=9606 G	1.8	0.023	PRDX2
Q9UHV9	PFDN2	Prefoldin subunit 2	1.8	0.024	PRDX2
P63104	YWHAZ	14-3-3 protein zeta/delta	1.7	0.000	PRDX2
A0A024R442	A0A024R442	>tr|A0A024R442|A0A024R442_HUMAN Aspartyl aminopeptidase, isoform CRA_b OS=Homo sapiens OX=9606 GN=DNPEP PE=1 SV=1;>sp|Q9ULA0|DNPEP_HUMAN Aspartyl aminopeptidase OS=Homo sapiens OX=9606 GN=DNPEP PE=1 SV=1;>tr|E7ETB3|E7ETB3_HUMAN Aspartyl aminopeptidase, iso	1.7	0.005	PRDX2
Q9NTK5	OLA1	Obg-like ATPase 1	1.7	0.022	PRDX2
Q9NV56	MRGBP	MRG/MORF4L-binding protein	1.7	0.025	PRDX2
B4DHR0	RABEP2	Rab GTPase-binding effector protein 2	1.7	0.025	PRDX2
Q13131	PRKAA1	5'-AMP-activated protein kinase catalytic subunit alpha-1	1.7	0.030	PRDX2
E9PLK3	NPEPPS	Aminopeptidase	1.6	0.000	PRDX2
Q93009	USP7	Ubiquitin carboxyl-terminal hydrolase 7	1.6	0.000	PRDX2
P28066	PSMA5	Proteasome subunit alpha type-5	1.6	0.000	PRDX2
O15212	PFDN6	Prefoldin subunit 6	1.6	0.001	PRDX2
Q32MZ4	LRRFIP1	Leucine-rich repeat flightless-interacting protein 1	1.6	0.012	PRDX2
Q9UKN8	GTF3C4	General transcription factor 3C polypeptide 4	1.6	0.033	PRDX2
P57076	CFAP298	Cilia- and flagella-associated protein 298	1.6	0.038	PRDX2
C9J0I9	ZC3HC1	Nuclear-interacting partner of ALK	1.5	0.000	PRDX2
E7ESY4	MTA1	Metastasis-associated protein MTA1	1.5	0.000	PRDX2
Q15424	SAFB	Scaffold attachment factor B1	1.5	0.001	PRDX2
Q15843	NEDD8	NEDD8	1.5	0.001	PRDX2
Q9UBE0	SAE1	SUMO-activating enzyme subunit 1	1.5	0.004	PRDX2
Q9UEY8	ADD3	Gamma-adducin	1.5	0.011	PRDX2
A0A087X0K9	TJP1	Tight junction protein ZO-1	1.5	0.011	PRDX2
Q5RKV6	EXOSC6	Exosome complex component MTR3	1.5	0.012	PRDX2
A0A087WT45	GRIPAP1	GRIP1-associated protein 1	1.5	0.017	PRDX2
P67809	YBX1	Y-box-binding protein 1	1.5	0.035	PRDX2
Q9BSJ8	ESYT1	Extended synaptotagmin-1	1.5	0.037	PRDX2
Q8N6R0	EEF1AKNMT	eEF1A lysine and N-terminal methyltransferase	1.5	0.040	PRDX2
Q9BY42	RTF2	Replication termination factor 2	1.4	0.000	PRDX2
P35998	PSMC2	26S proteasome regulatory subunit 7	1.4	0.001	PRDX2
Q9Y3F4	STRAP	Serine-threonine kinase receptor-associated protein	1.4	0.002	PRDX2
Q13185	CBX3	Chromobox protein homolog 3	1.4	0.021	PRDX2
H7C128	BRD8	Bromodomain-containing protein 8	1.4	0.031	PRDX2
Q8N806	UBR7	Putative E3 ubiquitin-protein ligase UBR7	1.3	0.032	PRDX2
P15104	GLUL	Glutamine synthetase	1.2	0.000	PRDX2
P23528	CFL1	Cofilin-1	1.2	0.000	PRDX2
P12270	TPR	Nucleoprotein TPR	1.2	0.000	PRDX2
Q96D09	GPRASP2	G-protein coupled receptor-associated sorting protein 2	1.2	0.001	PRDX2
P62191	PSMC1	26S proteasome regulatory subunit 4	1.2	0.001	PRDX2
Q9UPN7	PPP6R1	Serine/threonine-protein phosphatase 6 regulatory subunit 1	1.2	0.008	PRDX2
E5KLJ9	OPA1	Dynamin-like 120 kDa protein, form S1	1.2	0.011	PRDX2
Q13564	NAE1	NEDD8-activating enzyme E1 regulatory subunit	1.2	0.025	PRDX2
P23588	EIF4B	Eukaryotic translation initiation factor 4B	1.2	0.039	PRDX2
O14545	TRAFD1	TRAF-type zinc finger domain-containing protein 1	1.1	0.000	PRDX2
Q3ZCQ8	TIMM50	Mitochondrial import inner membrane translocase subunit TIM50	1.1	0.001	PRDX2
Q9NX63	CHCHD3	MICOS complex subunit MIC19	1.1	0.001	PRDX2
C9J2Y9	POLR2B	DNA-directed RNA polymerase subunit beta	1.1	0.003	PRDX2
P25786	PSMA1	Proteasome subunit alpha type-1	1.1	0.003	PRDX2
O15355	PPM1G	Protein phosphatase 1G	1.1	0.008	PRDX2
Q9BTE6	AARSD1	Alanyl-tRNA editing protein Aarsd1	1.1	0.011	PRDX2
Q9NXF7	DCAF16	DDB1- and CUL4-associated factor 16	1.1	0.013	PRDX2
Q99504	EYA3	Eyes absent homolog 3	1.1	0.015	PRDX2
P48147	PREP	Prolyl endopeptidase	1.1	0.031	PRDX2
P29218	IMPA1	Inositol monophosphatase 1	1.1	0.031	PRDX2
Q8N999	C12orf29	Uncharacterized protein C12orf29	1.1	0.035	PRDX2
Q13177	PAK2	Serine/threonine-protein kinase PAK 2	1.1	0.039	PRDX2
O00429	DNM1L	Dynamin-1-like protein	1.1	0.047	PRDX2
P20020	ATP2B1	Plasma membrane calcium-transporting ATPase 1	1.0	0.003	PRDX2
Q68E01	INTS3	Integrator complex subunit 3	1.0	0.012	PRDX2
Q96K76	USP47	Ubiquitin carboxyl-terminal hydrolase 47	1.0	0.016	PRDX2
P53985	SLC16A1	Monocarboxylate transporter 1	1.0	0.020	PRDX2
O60784	TOM1	Target of Myb protein 1	1.0	0.022	PRDX2
P49588	AARS1	Alanine--tRNA ligase, cytoplasmic	1.0	0.033	PRDX2
X6RM00	ERC1	ELKS/Rab6-interacting/CAST family member 1	1.0	0.035	PRDX2
O94776	MTA2	Metastasis-associated protein MTA2	7.0	0.000	PRDX3
P49366	DHPS	Deoxyhypusine synthase	7.0	0.000	PRDX3
P61289	PSME3	Proteasome activator complex subunit 3	5.0	0.000	PRDX3
Q16576	RBBP7	Histone-binding protein RBBP7	5.0	0.000	PRDX3
P67936	TPM4	Tropomyosin alpha-4 chain	4.9	0.000	PRDX3
H0Y4R1	IMPDH2	Inosine-5'-monophosphate dehydrogenase 2	4.6	0.000	PRDX3
P41250	GARS1	Glycine--tRNA ligase	4.3	0.000	PRDX3
P22234	PAICS	Multifunctional protein ADE2 [Includes: Phosphoribosylaminoimidazole-succinocarboxamide synthase	4.3	0.000	PRDX3
O14744	PRMT5	Protein arginine N-methyltransferase 5	3.9	0.000	PRDX3
Q9NQP4	PFDN4	Prefoldin subunit 4	3.7	0.000	PRDX3
Q96BR5	COA7	Cytochrome c oxidase assembly factor 7	3.6	0.000	PRDX3
Q09028	RBBP4	Histone-binding protein RBBP4	3.6	0.000	PRDX3
O00165	HAX1	HCLS1-associated protein X-1	3.4	0.000	PRDX3
E7EVA0	MAP4	Microtubule-associated protein	3.4	0.001	PRDX3
P20839	IMPDH1	Inosine-5'-monophosphate dehydrogenase 1	3.3	0.000	PRDX3
P30041	PRDX6	Peroxiredoxin-6	3.3	0.000	PRDX3
Q9GZU8	PSME3IP1	PSME3-interacting protein	3.2	0.000	PRDX3
Q96EW2	HSPBAP1	HSPB1-associated protein 1	3.2	0.000	PRDX3
Q96RS6	NUDCD1	NudC domain-containing protein 1	3.1	0.000	PRDX3
Q9UEY8	ADD3	Gamma-adducin	3.1	0.000	PRDX3
Q96KB5	PBK	Lymphokine-activated killer T-cell-originated protein kinase	3.1	0.000	PRDX3
Q9UHV9	PFDN2	Prefoldin subunit 2	3.0	0.000	PRDX3
Q13547	HDAC1	Histone deacetylase 1	3.0	0.000	PRDX3
Q6DKK2	TTC19	Tetratricopeptide repeat protein 19, mitochondrial	3.0	0.001	PRDX3
P63151	PPP2R2A	Serine/threonine-protein phosphatase 2A 55 kDa regulatory subunit B alpha isoform	3.0	0.001	PRDX3
O43143	DHX15	Pre-mRNA-splicing factor ATP-dependent RNA helicase DHX15	2.9	0.000	PRDX3
Q32MZ4	LRRFIP1	Leucine-rich repeat flightless-interacting protein 1	2.9	0.000	PRDX3
J3QQZ9	PNPO	Pyridoxal 5'-phosphate synthase	2.9	0.000	PRDX3
A0A024R442	A0A024R442	>tr|A0A024R442|A0A024R442_HUMAN Aspartyl aminopeptidase, isoform CRA_b OS=Homo sapiens OX=9606 GN=DNPEP PE=1 SV=1;>sp|Q9ULA0|DNPEP_HUMAN Aspartyl aminopeptidase OS=Homo sapiens OX=9606 GN=DNPEP PE=1 SV=1;>tr|E7ETB3|E7ETB3_HUMAN Aspartyl aminopeptidase, iso	2.7	0.000	PRDX3
Q8NFC6	BOD1L1	Biorientation of chromosomes in cell division protein 1-like 1	2.7	0.000	PRDX3
H0YEN2	PPP6R3	Serine/threonine-protein phosphatase 6 regulatory subunit 3	2.7	0.000	PRDX3
P10599	TXN	Thioredoxin	2.7	0.002	PRDX3
Q99598	TSNAX	Translin-associated protein X	2.6	0.000	PRDX3
Q5QNY5	PEX19	Peroxin-19	2.6	0.000	PRDX3
Q9C0C9	UBE2O	(E3-independent) E2 ubiquitin-conjugating enzyme	2.5	0.000	PRDX3
P31153	MAT2A	S-adenosylmethionine synthase isoform type-2	2.5	0.000	PRDX3
Q92769	HDAC2	Histone deacetylase 2	2.5	0.000	PRDX3
A0A2U3TZY2	CLPB	Caseinolytic peptidase B protein homolog	2.5	0.001	PRDX3
A0A087WT45	GRIPAP1	GRIP1-associated protein 1	2.5	0.001	PRDX3
P12955	PEPD	Xaa-Pro dipeptidase	2.5	0.002	PRDX3
Q9C0C2	TNKS1BP1	182 kDa tankyrase-1-binding protein	2.5	0.005	PRDX3
Q9BQA1	WDR77	Methylosome protein 50	2.5	0.008	PRDX3
Q8N3X1	FNBP4	Formin-binding protein 4	2.4	0.000	PRDX3
P62258	YWHAE	14-3-3 protein epsilon	2.4	0.000	PRDX3
P48147	PREP	Prolyl endopeptidase	2.4	0.000	PRDX3
H7C3C4	SLC4A7	Anion exchange protein	2.4	0.001	PRDX3
Q96DB5	RMDN1	Regulator of microtubule dynamics protein 1	2.4	0.003	PRDX3
Q9BPU6	DPYSL5	Dihydropyrimidinase-related protein 5	2.3	0.000	PRDX3
Q9Y478	PRKAB1	5'-AMP-activated protein kinase subunit beta-1	2.3	0.001	PRDX3
Q8NC51	SERBP1	Plasminogen activator inhibitor 1 RNA-binding protein	2.3	0.001	PRDX3
H0YKU1	TMOD3	Tropomodulin-3	2.3	0.002	PRDX3
P36551	CPOX	Oxygen-dependent coproporphyrinogen-III oxidase, mitochondrial	2.3	0.034	PRDX3
O95831	AIFM1	Apoptosis-inducing factor 1, mitochondrial	2.2	0.000	PRDX3
Q15276	RABEP1	Rab GTPase-binding effector protein 1	2.2	0.000	PRDX3
P23526	AHCY	Adenosylhomocysteinase	2.2	0.000	PRDX3
E7EV99	ADD1	Alpha-adducin	2.1	0.000	PRDX3
Q9BRP1	PDCD2L	Programmed cell death protein 2-like	2.1	0.000	PRDX3
Q9NT62	ATG3	Ubiquitin-like-conjugating enzyme ATG3	2.1	0.001	PRDX3
Q9HA64	FN3KRP	Ketosamine-3-kinase	2.1	0.002	PRDX3
Q13131	PRKAA1	5'-AMP-activated protein kinase catalytic subunit alpha-1	2.1	0.004	PRDX3
P52788	SMS	Spermine synthase	2.1	0.004	PRDX3
A0A0C4DGB5	CAST	Calpain inhibitor	2.1	0.006	PRDX3
P54646	PRKAA2	5'-AMP-activated protein kinase catalytic subunit alpha-2	2.0	0.000	PRDX3
O15212	PFDN6	Prefoldin subunit 6	2.0	0.000	PRDX3
Q9Y3F4	STRAP	Serine-threonine kinase receptor-associated protein	2.0	0.000	PRDX3
Q96D09	GPRASP2	G-protein coupled receptor-associated sorting protein 2	2.0	0.000	PRDX3
P29218	IMPA1	Inositol monophosphatase 1	2.0	0.004	PRDX3
Q9Y266	NUDC	Nuclear migration protein nudC	2.0	0.006	PRDX3
P30044	PRDX5	Peroxiredoxin-5, mitochondrial	13.2	0.000	PRDX3
A0A1C7CYX9	DPYSL2	Dihydropyrimidinase-related protein 2	1.9	0.000	PRDX3
Q15181	PPA1	Inorganic pyrophosphatase	1.9	0.000	PRDX3
Q13200	PSMD2	26S proteasome non-ATPase regulatory subunit 2	1.9	0.000	PRDX3
F5GZS6	SLC3A2	4F2 cell-surface antigen heavy chain	1.9	0.006	PRDX3
Q96B36	AKT1S1	Proline-rich AKT1 substrate 1	1.9	0.006	PRDX3
A0A087WXS7	A0A087WXS7	>tr|A0A087WXS7|A0A087WXS7_HUMAN ATPase ASNA1 OS=Homo sapiens OX=9606 GN=ASNA1 PE=1 SV=1;>sp|O43681|ASNA_HUMAN ATPase ASNA1 OS=Homo sapiens OX=9606 GN=ASNA1 PE=1 SV=2;>tr|K7ERW9|K7ERW9_HUMAN Arsenical pump-driving ATPase (Fragment) OS=Homo sapiens OX=9606 G	1.9	0.008	PRDX3
Q9NTK5	OLA1	Obg-like ATPase 1	1.9	0.008	PRDX3
Q13177	PAK2	Serine/threonine-protein kinase PAK 2	1.8	0.000	PRDX3
Q13564	NAE1	NEDD8-activating enzyme E1 regulatory subunit	1.8	0.000	PRDX3
B7Z7F3	RANBP3	Ran-binding protein 3	1.8	0.004	PRDX3
F8W1A4	AK2	Adenylate kinase 2, mitochondrial	1.8	0.004	PRDX3
P54619	PRKAG1	5'-AMP-activated protein kinase subunit gamma-1	1.8	0.005	PRDX3
P61758	VBP1	Prefoldin subunit 3	1.8	0.005	PRDX3
H7C128	BRD8	Bromodomain-containing protein 8	1.8	0.006	PRDX3
Q9H773	DCTPP1	dCTP pyrophosphatase 1	1.8	0.007	PRDX3
Q8IVM0	CCDC50	Coiled-coil domain-containing protein 50	1.8	0.011	PRDX3
Q9UKN8	GTF3C4	General transcription factor 3C polypeptide 4	1.8	0.012	PRDX3
Q9BRS2	RIOK1	Serine/threonine-protein kinase RIO1	1.8	0.012	PRDX3
Q6WKZ4	RAB11FIP1	Rab11 family-interacting protein 1	1.8	0.016	PRDX3
O43164	PJA2	E3 ubiquitin-protein ligase Praja-2	1.7	0.000	PRDX3
Q5T4U8	RABGGTB	Geranylgeranyl transferase type-2 subunit beta	1.7	0.003	PRDX3
E9PGT1	TSN	Component 3 of promoter of RISC	1.7	0.005	PRDX3
P52888	THOP1	Thimet oligopeptidase	1.7	0.005	PRDX3
P85037	FOXK1	Forkhead box protein K1	1.7	0.006	PRDX3
Q8N999	C12orf29	Uncharacterized protein C12orf29	1.7	0.012	PRDX3
B1ANM7	FAF1	FAS-associated factor 1	1.7	0.019	PRDX3
Q9Y6A5	TACC3	Transforming acidic coiled-coil-containing protein 3	1.7	0.022	PRDX3
Q96HC4	PDLIM5	PDZ and LIM domain protein 5	1.6	0.000	PRDX3
P07195	LDHB	L-lactate dehydrogenase B chain	1.6	0.000	PRDX3
O75821	EIF3G	Eukaryotic translation initiation factor 3 subunit G	1.6	0.000	PRDX3
H7BZM7	ZPR1	Zinc finger protein ZPR1	1.6	0.000	PRDX3
P23528	CFL1	Cofilin-1	1.6	0.000	PRDX3
P32119	PRDX2	Peroxiredoxin-2	1.6	0.002	PRDX3
Q9HB71	CACYBP	Calcyclin-binding protein	1.6	0.002	PRDX3
B4DHR0	RABEP2	Rab GTPase-binding effector protein 2	1.6	0.013	PRDX3
P11802	CDK4	Cyclin-dependent kinase 4	1.6	0.021	PRDX3
P53985	SLC16A1	Monocarboxylate transporter 1	1.5	0.000	PRDX3
P45973	CBX5	Chromobox protein homolog 5	1.5	0.000	PRDX3
E5KLJ9	OPA1	Dynamin-like 120 kDa protein, form S1	1.5	0.000	PRDX3
Q9NSD9	FARSB	Phenylalanine--tRNA ligase beta subunit	1.5	0.000	PRDX3
P23381	WARS1	Tryptophan--tRNA ligase, cytoplasmic	1.5	0.000	PRDX3
O43852	CALU	Calumenin	1.5	0.001	PRDX3
P60900	PSMA6	Proteasome subunit alpha type-6	1.5	0.001	PRDX3
O94992	HEXIM1	Protein HEXIM1	1.5	0.002	PRDX3
P27348	YWHAQ	14-3-3 protein theta	1.5	0.005	PRDX3
Q12929	EPS8	Epidermal growth factor receptor kinase substrate 8	1.5	0.030	PRDX3
Q13541	EIF4EBP1	Eukaryotic translation initiation factor 4E-binding protein 1	1.5	0.030	PRDX3
O95825	CRYZL1	Quinone oxidoreductase-like protein 1	1.5	0.034	PRDX3
E9PLK3	NPEPPS	Aminopeptidase	1.4	0.000	PRDX3
Q9NXF7	DCAF16	DDB1- and CUL4-associated factor 16	1.4	0.001	PRDX3
P61981	YWHAG	14-3-3 protein gamma	1.4	0.001	PRDX3
Q15008	PSMD6	26S proteasome non-ATPase regulatory subunit 6	1.4	0.001	PRDX3
P40222	TXLNA	Alpha-taxilin	1.4	0.004	PRDX3
P47985	UQCRFS1	Cytochrome b-c1 complex subunit Rieske, mitochondrial	1.4	0.008	PRDX3
P78371	CCT2	T-complex protein 1 subunit beta	1.4	0.011	PRDX3
Q9P258	RCC2	Protein RCC2	1.4	0.016	PRDX3
F8VXG7	SCAF11	Protein SCAF11	1.4	0.035	PRDX3
E5RGS4	PFDN1	Prefoldin subunit 1	1.4	0.045	PRDX3
C9J0I9	ZC3HC1	Nuclear-interacting partner of ALK	1.3	0.000	PRDX3
F8VVL1	DENR	Density-regulated protein	1.3	0.000	PRDX3
A0A2R8Y811	RPS14	40S ribosomal protein S14	1.3	0.000	PRDX3
Q3ZCQ8	TIMM50	Mitochondrial import inner membrane translocase subunit TIM50	1.3	0.000	PRDX3
P61221	ABCE1	ATP-binding cassette sub-family E member 1	1.3	0.000	PRDX3
Q06203	PPAT	Amidophosphoribosyltransferase	1.3	0.001	PRDX3
Q9NUQ3	TXLNG	Gamma-taxilin	1.3	0.004	PRDX3
P06748	NPM1	Nucleophosmin	1.3	0.014	PRDX3
Q9NPH2	ISYNA1	Inositol-3-phosphate synthase 1	1.3	0.017	PRDX3
P78356	PIP4K2B	Phosphatidylinositol 5-phosphate 4-kinase type-2 beta	1.3	0.035	PRDX3
P62191	PSMC1	26S proteasome regulatory subunit 4	1.2	0.000	PRDX3
P63104	YWHAZ	14-3-3 protein zeta/delta	1.2	0.000	PRDX3
Q9Y230	RUVBL2	RuvB-like 2	1.2	0.000	PRDX3
Q93009	USP7	Ubiquitin carboxyl-terminal hydrolase 7	1.2	0.000	PRDX3
Q9UHY1	NRBP1	Nuclear receptor-binding protein	1.2	0.000	PRDX3
P62826	RAN	GTP-binding nuclear protein Ran	1.2	0.000	PRDX3
Q9Y265	RUVBL1	RuvB-like 1	1.2	0.001	PRDX3
Q96RL1	UIMC1	BRCA1-A complex subunit RAP80	1.2	0.011	PRDX3
Q9UBE0	SAE1	SUMO-activating enzyme subunit 1	1.2	0.015	PRDX3
P43304	GPD2	Glycerol-3-phosphate dehydrogenase, mitochondrial	1.2	0.022	PRDX3
Q9NV56	MRGBP	MRG/MORF4L-binding protein	1.2	0.031	PRDX3
P11142	HSPA8	Heat shock cognate 71 kDa protein	1.1	0.000	PRDX3
P54578	USP14	Ubiquitin carboxyl-terminal hydrolase 14	1.1	0.002	PRDX3
Q92598	HSPH1	Heat shock protein 105 kDa	1.1	0.002	PRDX3
P55072	VCP	Transitional endoplasmic reticulum ATPase	1.1	0.006	PRDX3
P34932	HSPA4	Heat shock 70 kDa protein 4	1.1	0.012	PRDX3
A0A087X0K9	TJP1	Tight junction protein ZO-1	1.1	0.016	PRDX3
P05386	RPLP1	60S acidic ribosomal protein P1	1.1	0.016	PRDX3
O00487	PSMD14	26S proteasome non-ATPase regulatory subunit 14	1.0	0.004	PRDX3
P49321	NASP	Nuclear autoantigenic sperm protein	1.0	0.018	PRDX3
P31946	YWHAB	14-3-3 protein beta/alpha	1.0	0.025	PRDX3
Q9UKK9	NUDT5	ADP-sugar pyrophosphatase	1.0	0.028	PRDX3
Q96RG2	PASK	PAS domain-containing serine/threonine-protein kinase	1.0	0.035	PRDX3
Q9BRX5	GINS3	DNA replication complex GINS protein PSF3	1.0	0.037	PRDX3
Q13185	CBX3	Chromobox protein homolog 3	1.0	0.046	PRDX3
O94776	MTA2	Metastasis-associated protein MTA2	7.0	0.000	PRDX4
P49366	DHPS	Deoxyhypusine synthase	6.8	0.000	PRDX4
P41250	GARS1	Glycine--tRNA ligase	5.7	0.000	PRDX4
P61289	PSME3	Proteasome activator complex subunit 3	5.5	0.000	PRDX4
P22234	PAICS	Multifunctional protein ADE2 [Includes: Phosphoribosylaminoimidazole-succinocarboxamide synthase	5.0	0.000	PRDX4
P63151	PPP2R2A	Serine/threonine-protein phosphatase 2A 55 kDa regulatory subunit B alpha isoform	4.9	0.000	PRDX4
P67936	TPM4	Tropomyosin alpha-4 chain	4.8	0.000	PRDX4
H0Y4R1	IMPDH2	Inosine-5'-monophosphate dehydrogenase 2	4.2	0.000	PRDX4
Q5QNY5	PEX19	Peroxin-19	4.0	0.000	PRDX4
Q96KB5	PBK	Lymphokine-activated killer T-cell-originated protein kinase	3.9	0.000	PRDX4
Q92769	HDAC2	Histone deacetylase 2	3.7	0.000	PRDX4
O00165	HAX1	HCLS1-associated protein X-1	3.6	0.000	PRDX4
O14744	PRMT5	Protein arginine N-methyltransferase 5	3.6	0.001	PRDX4
Q96BR5	COA7	Cytochrome c oxidase assembly factor 7	3.5	0.000	PRDX4
P30041	PRDX6	Peroxiredoxin-6	3.5	0.000	PRDX4
P54646	PRKAA2	5'-AMP-activated protein kinase catalytic subunit alpha-2	3.5	0.000	PRDX4
Q9NQP4	PFDN4	Prefoldin subunit 4	3.5	0.000	PRDX4
Q15276	RABEP1	Rab GTPase-binding effector protein 1	3.5	0.000	PRDX4
Q9GZU8	PSME3IP1	PSME3-interacting protein	3.4	0.000	PRDX4
Q9BSJ8	ESYT1	Extended synaptotagmin-1	3.4	0.000	PRDX4
Q13547	HDAC1	Histone deacetylase 1	3.4	0.000	PRDX4
Q16576	RBBP7	Histone-binding protein RBBP7	3.4	0.000	PRDX4
P10599	TXN	Thioredoxin	3.3	0.000	PRDX4
Q9UHV9	PFDN2	Prefoldin subunit 2	3.3	0.000	PRDX4
P23526	AHCY	Adenosylhomocysteinase	3.3	0.000	PRDX4
O43143	DHX15	Pre-mRNA-splicing factor ATP-dependent RNA helicase DHX15	3.2	0.000	PRDX4
Q32MZ4	LRRFIP1	Leucine-rich repeat flightless-interacting protein 1	3.2	0.000	PRDX4
J3QQZ9	PNPO	Pyridoxal 5'-phosphate synthase	3.2	0.000	PRDX4
Q6DKK2	TTC19	Tetratricopeptide repeat protein 19, mitochondrial	3.2	0.001	PRDX4
A0A024R442	A0A024R442	>tr|A0A024R442|A0A024R442_HUMAN Aspartyl aminopeptidase, isoform CRA_b OS=Homo sapiens OX=9606 GN=DNPEP PE=1 SV=1;>sp|Q9ULA0|DNPEP_HUMAN Aspartyl aminopeptidase OS=Homo sapiens OX=9606 GN=DNPEP PE=1 SV=1;>tr|E7ETB3|E7ETB3_HUMAN Aspartyl aminopeptidase, iso	3.1	0.000	PRDX4
Q8NFC6	BOD1L1	Biorientation of chromosomes in cell division protein 1-like 1	3.0	0.000	PRDX4
Q96EW2	HSPBAP1	HSPB1-associated protein 1	3.0	0.000	PRDX4
P20839	IMPDH1	Inosine-5'-monophosphate dehydrogenase 1	3.0	0.000	PRDX4
Q96DB5	RMDN1	Regulator of microtubule dynamics protein 1	3.0	0.001	PRDX4
A0A0A0MR02	VDAC2	Outer mitochondrial membrane protein porin 2	2.9	0.000	PRDX4
Q9BQA1	WDR77	Methylosome protein 50	2.9	0.003	PRDX4
P42574	CASP3	Caspase-3	2.8	0.000	PRDX4
Q09028	RBBP4	Histone-binding protein RBBP4	2.8	0.000	PRDX4
P48147	PREP	Prolyl endopeptidase	2.8	0.000	PRDX4
Q96A49	SYAP1	Synapse-associated protein 1	2.8	0.001	PRDX4
A0A2U3TZY2	CLPB	Caseinolytic peptidase B protein homolog	2.8	0.001	PRDX4
P61758	VBP1	Prefoldin subunit 3	2.6	0.000	PRDX4
O15212	PFDN6	Prefoldin subunit 6	2.6	0.000	PRDX4
H0YEN2	PPP6R3	Serine/threonine-protein phosphatase 6 regulatory subunit 3	2.6	0.000	PRDX4
P11802	CDK4	Cyclin-dependent kinase 4	2.6	0.001	PRDX4
P54619	PRKAG1	5'-AMP-activated protein kinase subunit gamma-1	2.6	0.001	PRDX4
O94992	HEXIM1	Protein HEXIM1	2.5	0.000	PRDX4
P62258	YWHAE	14-3-3 protein epsilon	2.5	0.000	PRDX4
Q9HA64	FN3KRP	Ketosamine-3-kinase	2.5	0.001	PRDX4
Q6FI81	CIAPIN1	Anamorsin	2.5	0.001	PRDX4
P12955	PEPD	Xaa-Pro dipeptidase	2.5	0.002	PRDX4
Q96RS6	NUDCD1	NudC domain-containing protein 1	2.4	0.000	PRDX4
H7BXI1	ESYT2	Extended synaptotagmin-2	2.4	0.000	PRDX4
Q99598	TSNAX	Translin-associated protein X	2.4	0.000	PRDX4
A0A1C7CYX9	DPYSL2	Dihydropyrimidinase-related protein 2	2.4	0.000	PRDX4
Q9C0C9	UBE2O	(E3-independent) E2 ubiquitin-conjugating enzyme	2.4	0.000	PRDX4
E9PGT1	TSN	Component 3 of promoter of RISC	2.4	0.001	PRDX4
Q9UEY8	ADD3	Gamma-adducin	2.3	0.000	PRDX4
Q8N3X1	FNBP4	Formin-binding protein 4	2.3	0.000	PRDX4
Q13564	NAE1	NEDD8-activating enzyme E1 regulatory subunit	2.3	0.000	PRDX4
Q13177	PAK2	Serine/threonine-protein kinase PAK 2	2.3	0.000	PRDX4
Q9Y3F4	STRAP	Serine-threonine kinase receptor-associated protein	2.3	0.000	PRDX4
Q9BRP1	PDCD2L	Programmed cell death protein 2-like	2.3	0.000	PRDX4
Q9NPH2	ISYNA1	Inositol-3-phosphate synthase 1	2.3	0.001	PRDX4
P85037	FOXK1	Forkhead box protein K1	2.3	0.001	PRDX4
A0A087WT45	GRIPAP1	GRIP1-associated protein 1	2.3	0.001	PRDX4
Q9Y266	NUDC	Nuclear migration protein nudC	2.3	0.004	PRDX4
P78371	CCT2	T-complex protein 1 subunit beta	2.2	0.000	PRDX4
F8W1A4	AK2	Adenylate kinase 2, mitochondrial	2.2	0.001	PRDX4
Q5T4U8	RABGGTB	Geranylgeranyl transferase type-2 subunit beta	2.2	0.002	PRDX4
Q13131	PRKAA1	5'-AMP-activated protein kinase catalytic subunit alpha-1	2.2	0.005	PRDX4
Q96EA4	SPDL1	Protein Spindly	2.2	0.008	PRDX4
E7EV99	ADD1	Alpha-adducin	2.1	0.000	PRDX4
P31153	MAT2A	S-adenosylmethionine synthase isoform type-2	2.1	0.000	PRDX4
Q13200	PSMD2	26S proteasome non-ATPase regulatory subunit 2	2.1	0.000	PRDX4
H7C3C4	SLC4A7	Anion exchange protein	2.1	0.001	PRDX4
Q9NT62	ATG3	Ubiquitin-like-conjugating enzyme ATG3	2.1	0.001	PRDX4
P52888	THOP1	Thimet oligopeptidase	2.1	0.001	PRDX4
P60900	PSMA6	Proteasome subunit alpha type-6	2.1	0.001	PRDX4
Q9Y478	PRKAB1	5'-AMP-activated protein kinase subunit beta-1	2.1	0.002	PRDX4
H0YKU1	TMOD3	Tropomodulin-3	2.1	0.004	PRDX4
A0A0C4DGB5	CAST	Calpain inhibitor	2.1	0.005	PRDX4
H7BZM7	ZPR1	Zinc finger protein ZPR1	2.0	0.000	PRDX4
P11142	HSPA8	Heat shock cognate 71 kDa protein	2.0	0.000	PRDX4
Q9H974	QTRT2	Queuine tRNA-ribosyltransferase accessory subunit 2	2.0	0.002	PRDX4
Q96RG2	PASK	PAS domain-containing serine/threonine-protein kinase	2.0	0.003	PRDX4
B7Z7F3	RANBP3	Ran-binding protein 3	2.0	0.003	PRDX4
P49903	SEPHS1	Selenide, water dikinase 1	2.0	0.006	PRDX4
P52788	SMS	Spermine synthase	2.0	0.009	PRDX4
P30044	PRDX5	Peroxiredoxin-5, mitochondrial	13.5	0.000	PRDX4
P49593	PPM1F	Protein phosphatase 1F	1.9	0.000	PRDX4
Q9BPU6	DPYSL5	Dihydropyrimidinase-related protein 5	1.9	0.000	PRDX4
E9PLK3	NPEPPS	Aminopeptidase	1.9	0.000	PRDX4
P23528	CFL1	Cofilin-1	1.9	0.000	PRDX4
P31689	DNAJA1	DnaJ homolog subfamily A member 1	1.9	0.001	PRDX4
P27348	YWHAQ	14-3-3 protein theta	1.9	0.001	PRDX4
Q8N806	UBR7	Putative E3 ubiquitin-protein ligase UBR7	1.9	0.001	PRDX4
E5KLJ9	OPA1	Dynamin-like 120 kDa protein, form S1	1.9	0.001	PRDX4
Q9NV56	MRGBP	MRG/MORF4L-binding protein	1.9	0.002	PRDX4
O43164	PJA2	E3 ubiquitin-protein ligase Praja-2	1.9	0.004	PRDX4
Q9Y6A5	TACC3	Transforming acidic coiled-coil-containing protein 3	1.9	0.032	PRDX4
Q9C0C2	TNKS1BP1	182 kDa tankyrase-1-binding protein	1.9	0.046	PRDX4
B1AK87	CAPZB	F-actin-capping protein subunit beta	1.8	0.000	PRDX4
P22314	UBA1	Ubiquitin-like modifier-activating enzyme 1	1.8	0.003	PRDX4
H7C128	BRD8	Bromodomain-containing protein 8	1.8	0.007	PRDX4
Q9H773	DCTPP1	dCTP pyrophosphatase 1	1.8	0.012	PRDX4
Q9BV44	THUMPD3	THUMP domain-containing protein 3	1.7	0.000	PRDX4
E7ESY4	MTA1	Metastasis-associated protein MTA1	1.7	0.000	PRDX4
Q96D09	GPRASP2	G-protein coupled receptor-associated sorting protein 2	1.7	0.000	PRDX4
P31946	YWHAB	14-3-3 protein beta/alpha	1.7	0.001	PRDX4
P43304	GPD2	Glycerol-3-phosphate dehydrogenase, mitochondrial	1.7	0.008	PRDX4
A0A087WXS7	A0A087WXS7	>tr|A0A087WXS7|A0A087WXS7_HUMAN ATPase ASNA1 OS=Homo sapiens OX=9606 GN=ASNA1 PE=1 SV=1;>sp|O43681|ASNA_HUMAN ATPase ASNA1 OS=Homo sapiens OX=9606 GN=ASNA1 PE=1 SV=2;>tr|K7ERW9|K7ERW9_HUMAN Arsenical pump-driving ATPase (Fragment) OS=Homo sapiens OX=9606 G	1.7	0.017	PRDX4
Q9UL15	BAG5	BAG family molecular chaperone regulator 5	1.7	0.027	PRDX4
A0A087X0K9	TJP1	Tight junction protein ZO-1	1.6	0.000	PRDX4
Q15738	NSDHL	Sterol-4-alpha-carboxylate 3-dehydrogenase, decarboxylating	1.6	0.000	PRDX4
Q15008	PSMD6	26S proteasome non-ATPase regulatory subunit 6	1.6	0.000	PRDX4
Q9NXF7	DCAF16	DDB1- and CUL4-associated factor 16	1.6	0.001	PRDX4
P30740	SERPINB1	Leukocyte elastase inhibitor	1.6	0.019	PRDX4
Q9BRS2	RIOK1	Serine/threonine-protein kinase RIO1	1.6	0.024	PRDX4
B4DHR0	RABEP2	Rab GTPase-binding effector protein 2	1.6	0.032	PRDX4
Q6WKZ4	RAB11FIP1	Rab11 family-interacting protein 1	1.6	0.033	PRDX4
A0AVT1	UBA6	Ubiquitin-like modifier-activating enzyme 6	1.6	0.040	PRDX4
Q96K76	USP47	Ubiquitin carboxyl-terminal hydrolase 47	1.5	0.000	PRDX4
E9PMI6	CLNS1A	Chloride channel, nucleotide sensitive 1A	1.5	0.000	PRDX4
Q06203	PPAT	Amidophosphoribosyltransferase	1.5	0.000	PRDX4
Q92598	HSPH1	Heat shock protein 105 kDa	1.5	0.000	PRDX4
O00487	PSMD14	26S proteasome non-ATPase regulatory subunit 14	1.5	0.000	PRDX4
P62191	PSMC1	26S proteasome regulatory subunit 4	1.5	0.000	PRDX4
Q13033	STRN3	Striatin-3	1.5	0.000	PRDX4
P63104	YWHAZ	14-3-3 protein zeta/delta	1.5	0.000	PRDX4
P34932	HSPA4	Heat shock 70 kDa protein 4	1.5	0.001	PRDX4
Q9UKK9	NUDT5	ADP-sugar pyrophosphatase	1.5	0.001	PRDX4
P35998	PSMC2	26S proteasome regulatory subunit 7	1.5	0.001	PRDX4
A0A0C4DGQ6	RPRD1A	Regulation of nuclear pre-mRNA domain-containing protein 1A	1.5	0.002	PRDX4
O95831	AIFM1	Apoptosis-inducing factor 1, mitochondrial	1.5	0.003	PRDX4
Q13185	CBX3	Chromobox protein homolog 3	1.5	0.006	PRDX4
O95295	SNAPIN	SNARE-associated protein Snapin	1.5	0.011	PRDX4
U3KQC1	WDR18	WD repeat-containing protein 18	1.5	0.017	PRDX4
Q8N6T3	ARFGAP1	ADP-ribosylation factor GTPase-activating protein 1	1.5	0.018	PRDX4
P00492	HPRT1	Hypoxanthine-guanine phosphoribosyltransferase	1.5	0.019	PRDX4
Q8N999	C12orf29	Uncharacterized protein C12orf29	1.5	0.025	PRDX4
J3KN87	J3KN87	>tr|J3KN87|J3KN87_HUMAN Serine/threonine-protein kinase Chk1 OS=Homo sapiens OX=9606 GN=CHEK1 PE=1 SV=1;>sp|O14757|CHK1_HUMAN Serine/threonine-protein kinase Chk1 OS=Homo sapiens OX=9606 GN=CHEK1 PE=1 SV=2;>tr|E7EPP6|E7EPP6_HUMAN Serine/threonine-protein k	1.5	0.032	PRDX4
P29966	MARCKS	Myristoylated alanine-rich C-kinase substrate	1.5	0.046	PRDX4
Q3ZCQ8	TIMM50	Mitochondrial import inner membrane translocase subunit TIM50	1.4	0.000	PRDX4
Q9NSD9	FARSB	Phenylalanine--tRNA ligase beta subunit	1.4	0.000	PRDX4
Q93009	USP7	Ubiquitin carboxyl-terminal hydrolase 7	1.4	0.000	PRDX4
P61981	YWHAG	14-3-3 protein gamma	1.4	0.001	PRDX4
P31930	UQCRC1	Cytochrome b-c1 complex subunit 1, mitochondrial	1.4	0.001	PRDX4
P29218	IMPA1	Inositol monophosphatase 1	1.4	0.004	PRDX4
Q14C86	GAPVD1	GTPase-activating protein and VPS9 domain-containing protein 1	1.4	0.008	PRDX4
B1ANM7	FAF1	FAS-associated factor 1	1.4	0.039	PRDX4
Q9Y277	VDAC3	Voltage-dependent anion-selective channel protein 3	1.4	0.043	PRDX4
P31939	ATIC	Bifunctional purine biosynthesis protein ATIC	1.3	0.000	PRDX4
Q9Y230	RUVBL2	RuvB-like 2	1.3	0.000	PRDX4
P23381	WARS1	Tryptophan--tRNA ligase, cytoplasmic	1.3	0.000	PRDX4
P15104	GLUL	Glutamine synthetase	1.3	0.000	PRDX4
Q9Y265	RUVBL1	RuvB-like 1	1.3	0.001	PRDX4
C9J0I9	ZC3HC1	Nuclear-interacting partner of ALK	1.3	0.001	PRDX4
P22695	UQCRC2	Cytochrome b-c1 complex subunit 2, mitochondrial	1.3	0.003	PRDX4
Q9UJS0	SLC25A13	Calcium-binding mitochondrial carrier protein Aralar2	1.3	0.004	PRDX4
P0DMV9	HSPA1B	Heat shock 70 kDa protein 1B	1.3	0.006	PRDX4
Q9UHD1	CHORDC1	Cysteine and histidine-rich domain-containing protein 1	1.3	0.009	PRDX4
Q96B36	AKT1S1	Proline-rich AKT1 substrate 1	1.3	0.012	PRDX4
Q9H0U4	RAB1B	Ras-related protein Rab-1B	1.3	0.016	PRDX4
J3KN29	PSMD9	26S proteasome non-ATPase regulatory subunit 9	1.3	0.020	PRDX4
P12814	ACTN1	Alpha-actinin-1	1.3	0.035	PRDX4
Q16204	CCDC6	Coiled-coil domain-containing protein 6	1.3	0.043	PRDX4
P78356	PIP4K2B	Phosphatidylinositol 5-phosphate 4-kinase type-2 beta	1.3	0.045	PRDX4
P25705	ATP5F1A	ATP synthase subunit alpha, mitochondrial	1.2	0.000	PRDX4
P05023	ATP1A1	Sodium/potassium-transporting ATPase subunit alpha-1	1.2	0.000	PRDX4
Q8WXD5	GEMIN6	Gem-associated protein 6	1.2	0.000	PRDX4
P20020	ATP2B1	Plasma membrane calcium-transporting ATPase 1	1.2	0.001	PRDX4
P34897	SHMT2	Serine hydroxymethyltransferase, mitochondrial	1.2	0.005	PRDX4
K7EJQ8	HDHD2	Haloacid dehalogenase-like hydrolase domain-containing protein 2	1.2	0.009	PRDX4
P42771	CDKN2A	Cyclin-dependent kinase inhibitor 2A	1.2	0.010	PRDX4
Q5QPM7	PSMF1	Proteasome inhibitor PI31 subunit	1.2	0.013	PRDX4
P62333	PSMC6	26S proteasome regulatory subunit 10B	1.2	0.014	PRDX4
P15374	UCHL3	Ubiquitin carboxyl-terminal hydrolase isozyme L3	1.2	0.014	PRDX4
Q8IVM0	CCDC50	Coiled-coil domain-containing protein 50	1.2	0.032	PRDX4
O00231	PSMD11	26S proteasome non-ATPase regulatory subunit 11	1.2	0.032	PRDX4
Q13045	FLII	Protein flightless-1 homolog	1.1	0.000	PRDX4
Q9Y285	FARSA	Phenylalanine--tRNA ligase alpha subunit	1.1	0.000	PRDX4
P11310	ACADM	Medium-chain specific acyl-CoA dehydrogenase, mitochondrial	1.1	0.000	PRDX4
P36871	PGM1	Phosphoglucomutase-1	1.1	0.001	PRDX4
P61221	ABCE1	ATP-binding cassette sub-family E member 1	1.1	0.001	PRDX4
P06733	ENO1	Alpha-enolase	1.1	0.002	PRDX4
P62195	PSMC5	26S proteasome regulatory subunit 8	1.1	0.002	PRDX4
P00338	LDHA	L-lactate dehydrogenase A chain	1.1	0.002	PRDX4
Q9BTE6	AARSD1	Alanyl-tRNA editing protein Aarsd1	1.1	0.004	PRDX4
P45973	CBX5	Chromobox protein homolog 5	1.1	0.005	PRDX4
O43242	PSMD3	26S proteasome non-ATPase regulatory subunit 3	1.1	0.006	PRDX4
P07195	LDHB	L-lactate dehydrogenase B chain	1.1	0.007	PRDX4
Q15181	PPA1	Inorganic pyrophosphatase	1.1	0.008	PRDX4
P55072	VCP	Transitional endoplasmic reticulum ATPase	1.1	0.009	PRDX4
P53985	SLC16A1	Monocarboxylate transporter 1	1.1	0.009	PRDX4
O95453	PARN	Poly(A)-specific ribonuclease PARN	1.1	0.010	PRDX4
P05386	RPLP1	60S acidic ribosomal protein P1	1.1	0.015	PRDX4
P52907	CAPZA1	F-actin-capping protein subunit alpha-1	1.1	0.017	PRDX4
Q9HB71	CACYBP	Calcyclin-binding protein	1.1	0.022	PRDX4
P49321	NASP	Nuclear autoantigenic sperm protein	1.1	0.023	PRDX4
Q99536	VAT1	Synaptic vesicle membrane protein VAT-1 homolog	1.0	0.001	PRDX4
O60884	DNAJA2	DnaJ homolog subfamily A member 2	1.0	0.001	PRDX4
Q99460	PSMD1	26S proteasome non-ATPase regulatory subunit 1	1.0	0.003	PRDX4
Q8NBU5	ATAD1	ATPase family AAA domain-containing protein 1	1.0	0.029	PRDX4

Table S3. Peroxidatic cysteine-dependent interactors (PRDX-C_RS/PRDX-$C_{PR}S$)

uniprot ID	gene	protein	log ratio	p-value
**PRDX1 cysteine-dependent interactors**				
Q53H12	AGK	Acylglycerol kinase, mitochondrial	4.1	<0.001
O75531	BANF1	Barrier-to-autointegration factor	3.9	<0.001
P27824	CANX	Calnexin	3.7	<0.001
P22061	PCMT1	Protein-L-isoaspartate(D-aspartate) O-methyltransferase	3.4	0.001
P68133	ACTA1	Actin, alpha skeletal muscle	3.4	0.006
A0A024R4E5	HDLBP	High density lipoprotein binding protein	3.3	<0.001
P55084	HADHB	Trifunctional enzyme subunit beta, mitochondrial	3.3	<0.001
Q9NY93	DDX56	Probable ATP-dependent RNA helicase DDX56	3.3	0.001
Q9Y6A5	TACC3	Transforming acidic coiled-coil-containing protein 3	3.2	<0.001
Q9UHV9	PFDN2	Prefoldin subunit 2	3.2	<0.001
P55735	SEC13	Protein SEC13 homolog	3.2	0.001
P43897	TSFM	Elongation factor Ts, mitochondrial	3.2	0.001
P21964	COMT	Catechol O-methyltransferase	3.2	0.001
P62316	SNRPD2	Small nuclear ribonucleoprotein Sm D2	3.1	<0.001
P53621	COPA	Coatomer subunit alpha	3.1	<0.001
Q6P587	FAHD1	Acylpyruvase FAHD1, mitochondrial	3.1	0.001
Q01081	U2AF1	Splicing factor U2AF 35 kDa subunit	3.1	0.006
Q9P2J5	LARS1	Leucine--tRNA ligase, cytoplasmic	3.0	<0.001
P06132	UROD	Uroporphyrinogen decarboxylase	3.0	0.009
B7Z7F3	RANBP3	Ran-binding protein 3	2.9	<0.001
Q16513	PKN2	Serine/threonine-protein kinase N2	2.9	0.003
Q15654	TRIP6	Thyroid receptor-interacting protein 6	2.9	0.003
O00148	DDX39A	ATP-dependent RNA helicase DDX39A	2.9	0.008
P35268	RPL22	60S ribosomal protein L22	2.8	<0.001
C9JZR2	CTNND1	Catenin delta-1	2.8	<0.001
P62829	RPL23	60S ribosomal protein L23	2.8	<0.001
Q86V48	LUZP1	Leucine zipper protein 1	2.8	<0.001
P24666	ACP1	Low molecular weight phosphotyrosine protein phosphatase	2.8	0.001
Q9C0C2	TNKS1BP1	182 kDa tankyrase-1-binding protein	2.8	0.001
O14744	PRMT5	Protein arginine N-methyltransferase 5	2.8	0.002
Q96DH6	MSI2	RNA-binding protein Musashi homolog 2	2.8	0.003
Q15149	PLEC	Plectin	2.8	0.004
E7ETK0	RPS24	40S ribosomal protein S24	2.8	0.004
P39023	RPL3	60S ribosomal protein L3	2.7	<0.001
Q7Z406	MYH14	Myosin-14	2.7	<0.001
Q06124	PTPN11	Tyrosine-protein phosphatase non-receptor type 11	2.7	<0.001
Q9UHB6	LIMA1	LIM domain and actin-binding protein 1	2.7	<0.001
P52788	SMS	Spermine synthase	2.7	0.001
P27694	RPA1	Replication protein A 70 kDa DNA-binding subunit	2.7	0.002
Q9Y262	EIF3L	Eukaryotic translation initiation factor 3 subunit L	2.7	0.013
A0A087WTZ5	UBXN1	UBX domain-containing protein 1	2.6	<0.001
O94979	SEC31A	Protein transport protein Sec31A	2.6	<0.001
P48634	PRRC2A	Protein PRRC2A	2.6	<0.001
P31689	DNAJA1	DnaJ homolog subfamily A member 1	2.6	<0.001
E7EX90	DCTN1	Dynactin subunit 1	2.6	0.002
P51452	DUSP3	Dual specificity protein phosphatase 3	2.6	0.004
Q9GZT3	SLIRP	SRA stem-loop-interacting RNA-binding protein, mitochondrial	2.6	0.005
Q99460	PSMD1	26S proteasome non-ATPase regulatory subunit 1	2.6	0.005
Q6WKZ4	RAB11FIP1	Rab11 family-interacting protein 1	2.6	0.006
Q5VYK3	ECPAS	Proteasome adapter and scaffold protein ECM29	2.6	0.007
Q96AC1	FERMT2	Fermitin family homolog 2	2.6	0.009
O43143	DHX15	Pre-mRNA-splicing factor ATP-dependent RNA helicase DHX15	2.6	0.009
O95373	IPO7	Importin-7	2.6	0.010
Q8IWZ3	ANKHD1	Ankyrin repeat and KH domain-containing protein 1	2.5	<0.001
P22314	UBA1	Ubiquitin-like modifier-activating enzyme 1	2.5	<0.001
P48444	ARCN1	Coatomer subunit delta	2.5	<0.001
P62258	YWHAE	14-3-3 protein epsilon	2.5	<0.001
Q13185	CBX3	Chromobox protein homolog 3	2.5	<0.001
Q15075	EEA1	Early endosome antigen 1	2.5	0.010
P11387	TOP1	DNA topoisomerase 1	2.5	0.011
Q5SW79	CEP170	Centrosomal protein of 170 kDa	2.4	<0.001
Q9BSH4	TACO1	Translational activator of cytochrome c oxidase 1	2.4	<0.001
P15170	GSPT1	Eukaryotic peptide chain release factor GTP-binding subunit ERF3A	2.4	<0.001
P16615	ATP2A2	Sarcoplasmic/endoplasmic reticulum calcium ATPase 2	2.4	<0.001
Q14204	DYNC1H1	Cytoplasmic dynein 1 heavy chain 1	2.4	0.001
P15924	DSP	Desmoplakin	2.4	0.002
O95747	OXSR1	Serine/threonine-protein kinase OSR1	2.4	0.005
Q6IAA8	LAMTOR1	Ragulator complex protein LAMTOR1	2.4	0.006
P08559	PDHA1	Pyruvate dehydrogenase E1 component subunit alpha, somatic form, mitochondrial	2.4	0.006
Q9Y5Y2	NUBP2	Cytosolic Fe-S cluster assembly factor NUBP2	2.4	0.009
Q12948	FOXC1	Forkhead box protein C1	2.4	0.011
Q8TAT6	NPLOC4	Nuclear protein localization protein 4 homolog	2.4	0.014
Q5T7U1	GTF3C5	General transcription factor 3C polypeptide 5	2.3	<0.001
Q7Z6Z7	HUWE1	E3 ubiquitin-protein ligase HUWE1	2.3	<0.001
Q14247	CTTN	Src substrate cortactin	2.3	<0.001
Q9UHB9	SRP68	Signal recognition particle subunit SRP68	2.3	0.001
Q9BQ67	GRWD1	Glutamate-rich WD repeat-containing protein 1	2.3	0.002
A0A087WZ13	RAVER1	Ribonucleoprotein PTB-binding 1	2.3	0.003
P19525	EIF2AK2	Interferon-induced, double-stranded RNA-activated protein kinase	2.3	0.009
A0A087X1A5	STAU1	Double-stranded RNA-binding protein Staufen homolog 1	2.3	0.011
Q9NP97	DYNLRB1	Dynein light chain roadblock-type 1	2.3	0.014
P27708	CAD	CAD protein [Includes: Glutamine-dependent carbamoyl-phosphate synthase	2.3	0.017
Q9BYG3	NIFK	MKI67 FHA domain-interacting nucleolar phosphoprotein	2.3	0.020
Q15061	WDR43	WD repeat-containing protein 43	2.3	0.025
E5RGR0	LYPLA1	Acyl-protein thioesterase 1	2.3	0.049
P41252	IARS1	Isoleucine--tRNA ligase, cytoplasmic	2.2	<0.001
P35658	NUP214	Nuclear pore complex protein Nup214	2.2	<0.001
Q9UG54	MAP3K7	Mitogen-activated protein kinase kinase kinase	2.2	<0.001
P55809	OXCT1	Succinyl-CoA:3-ketoacid coenzyme A transferase 1, mitochondrial	2.2	0.001
Q14684	RRP1B	Ribosomal RNA processing protein 1 homolog B	2.2	0.002
P39748	FEN1	Flap endonuclease 1	2.2	0.007
P37108	SRP14	Signal recognition particle 14 kDa protein	2.2	0.008
F5H6E2	MYO1C	Unconventional myosin-Ic	2.2	0.010
P13807	GYS1	Glycogen [starch] synthase, muscle	2.2	0.013
Q8WUH6	TMEM263	Transmembrane protein 263	2.2	0.015
O43795	MYO1B	Unconventional myosin-Ib	2.2	0.016
O75663	TIPRL	TIP41-like protein	2.2	0.018
P49792	RANBP2	E3 SUMO-protein ligase RanBP2	2.2	0.030
E9PF10	NUP155	Nuclear pore complex protein Nup155	2.2	0.032
E9PDU5	WDR6	WD repeat-containing protein 6	2.1	<0.001
Q5T6F2	UBAP2	Ubiquitin-associated protein 2	2.1	<0.001
P22307	SCP2	Non-specific lipid-transfer protein	2.1	0.001
Q9Y6Y8	SEC23IP	SEC23-interacting protein	2.1	0.001
Q09666	AHNAK	Neuroblast differentiation-associated protein AHNAK	2.1	0.001
Q9BSJ8	ESYT1	Extended synaptotagmin-1	2.1	0.002
O75436	VPS26A	Vacuolar protein sorting-associated protein 26A	2.1	0.003
Q9UQE7	SMC3	Structural maintenance of chromosomes protein 3	2.1	0.007
P35579	MYH9	Myosin-9	2.1	0.012
Q92797	SYMPK	Symplekin	2.1	0.015
O75874	IDH1	Isocitrate dehydrogenase [NADP] cytoplasmic	2.1	0.018
Q4KMP7	TBC1D10B	TBC1 domain family member 10B	2.1	0.019
Q7L0Y3	TRMT10C	tRNA methyltransferase 10 homolog C	2.1	0.020
Q16822	PCK2	Phosphoenolpyruvate carboxykinase [GTP], mitochondrial	2.1	0.021
Q07021	C1QBP	Complement component 1 Q subcomponent-binding protein, mitochondrial	2.1	0.021
P29144	TPP2	Tripeptidyl-peptidase 2	2.1	0.023
Q15645	TRIP13	Pachytene checkpoint protein 2 homolog	2.1	0.025
Q8NCA5	FAM98A	Protein FAM98A	2.1	0.026
P50542	PEX5	Peroxisomal targeting signal 1 receptor	2.1	0.028
Q9HD26	GOPC	Golgi-associated PDZ and coiled-coil motif-containing protein	2.1	0.048
Q9Y2R4	DDX52	Probable ATP-dependent RNA helicase DDX52	2.1	0.049
Q9UNE7	STUB1	E3 ubiquitin-protein ligase CHIP	2.0	<0.001
Q14166	TTLL12	Tubulin--tyrosine ligase-like protein 12	2.0	<0.001
Q32MZ4	LRRFIP1	Leucine-rich repeat flightless-interacting protein 1	2.0	<0.001
Q14157	UBAP2L	Ubiquitin-associated protein 2-like	2.0	<0.001
P63272	SUPT4H1	Transcription elongation factor SPT4	2.0	0.001
P08708	RPS17	40S ribosomal protein S17	2.0	0.001
P30520	ADSS2	Adenylosuccinate synthetase isozyme 2	2.0	0.001
A0A087WVM4	MTHFD1L	Formyltetrahydrofolate synthetase	2.0	0.001
Q9ULV4	CORO1C	Coronin-1C	2.0	0.002
P61011	SRP54	Signal recognition particle 54 kDa protein	2.0	0.005
Q9UKD2	MRTO4	mRNA turnover protein 4 homolog	2.0	0.005
Q14683	SMC1A	Structural maintenance of chromosomes protein 1A	2.0	0.005
P42704	LRPPRC	Leucine-rich PPR motif-containing protein, mitochondrial	2.0	0.007
Q9H0C8	ILKAP	Integrin-linked kinase-associated serine/threonine phosphatase 2C	2.0	0.007
O14929	HAT1	Histone acetyltransferase type B catalytic subunit	2.0	0.008
Q86VP6	CAND1	Cullin-associated NEDD8-dissociated protein 1	2.0	0.014
P11388	TOP2A	DNA topoisomerase 2-alpha	2.0	0.015
P30048	PRDX3	Thioredoxin-dependent peroxide reductase, mitochondrial	2.0	0.016
Q13144	EIF2B5	Translation initiation factor eIF-2B subunit epsilon	2.0	0.017
P34949	MPI	Mannose-6-phosphate isomerase	2.0	0.018
O43660	PLRG1	Pleiotropic regulator 1	2.0	0.024
Q15750	TAB1	TGF-beta-activated kinase 1 and MAP3K7-binding protein 1	2.0	0.026
P23258	TUBG1	Tubulin gamma-1 chain	2.0	0.029
G3V1C3	API5	Apoptosis inhibitor 5	2.0	0.035
Q9H840	GEMIN7	Gem-associated protein 7	2.0	0.048
Q16204	CCDC6	Coiled-coil domain-containing protein 6	2.0	0.048
P19105	MYL12A	Myosin regulatory light chain 12A	2.0	0.049
Q9NXF7	DCAF16	DDB1- and CUL4-associated factor 16	1.9	<0.001
E7EVA0	MAP4	Microtubule-associated protein	1.9	<0.001
Q9UHD1	CHORDC1	Cysteine and histidine-rich domain-containing protein 1	1.9	<0.001
P49321	NASP	Nuclear autoantigenic sperm protein	1.9	<0.001
A0A087X0M4	SLC4A1AP	Kanadaptin	1.9	<0.001
Q7LBC6	KDM3B	Lysine-specific demethylase 3B	1.9	<0.001
A0A0U1RRM6	ENAH	Protein enabled homolog	1.9	<0.001
P48643	CCT5	T-complex protein 1 subunit epsilon	1.9	<0.001
Q8ND83	SLAIN1	SLAIN motif-containing protein 1	1.9	<0.001
O60784	TOM1	Target of Myb protein 1	1.9	0.001
P00568	AK1	Adenylate kinase isoenzyme 1	1.9	0.001
Q96GD0	PDXP	Pyridoxal phosphate phosphatase	1.9	0.001
O75832	PSMD10	26S proteasome non-ATPase regulatory subunit 10	1.9	0.001
P57740	NUP107	Nuclear pore complex protein Nup107	1.9	0.002
P46459	NSF	Vesicle-fusing ATPase	1.9	0.002
P48047	ATP5PO	ATP synthase subunit O, mitochondrial	1.9	0.003
O43837	IDH3B	Isocitrate dehydrogenase [NAD] subunit beta, mitochondrial	1.9	0.004
P33991	MCM4	DNA replication licensing factor MCM4	1.9	0.007
E7ESP9	NEFM	160 kDa neurofilament protein	1.9	0.008
Q9Y3I0	RTCB	RNA-splicing ligase RtcB homolog	1.9	0.008
O43719	HTATSF1	HIV Tat-specific factor 1	1.9	0.009
O00399	DCTN6	Dynactin subunit 6	1.9	0.010
Q9Y2L1	DIS3	Exosome complex exonuclease RRP44	1.9	0.015
Q9Y2V2	CARHSP1	Calcium-regulated heat-stable protein 1	1.9	0.015
H3BPE1	MACF1	Microtubule-actin cross-linking factor 1, isoforms 1/2/3/5	1.9	0.016
Q9NQ88	TIGAR	Fructose-2,6-bisphosphatase TIGAR	1.9	0.027
P09110	ACAA1	3-ketoacyl-CoA thiolase, peroxisomal	1.9	0.028
P23919	DTYMK	Thymidylate kinase	1.9	0.029
E9PLK3	NPEPPS	Aminopeptidase	1.9	0.031
P46940	IQGAP1	Ras GTPase-activating-like protein IQGAP1	1.9	0.034
P40939	HADHA	Trifunctional enzyme subunit alpha, mitochondrial	1.9	0.038
Q8NFC6	BOD1L1	Biorientation of chromosomes in cell division protein 1-like 1	1.9	0.039
P40222	TXLNA	Alpha-taxilin	1.9	0.041
E9PKP7	UBTF	Nucleolar transcription factor 1	1.9	0.043
O00487	PSMD14	26S proteasome non-ATPase regulatory subunit 14	1.9	0.045
P62333	PSMC6	26S proteasome regulatory subunit 10B	1.8	<0.001
E7EPN9	PRRC2C	Protein PRRC2C	1.8	<0.001
B1AK87	CAPZB	F-actin-capping protein subunit beta	1.8	<0.001
Q9BQ69	MACROD1	ADP-ribose glycohydrolase MACROD1	1.8	<0.001
Q14126	DSG2	Desmoglein-2	1.8	<0.001
P46776	RPL27A	60S ribosomal protein L27a	1.8	<0.001
P13861	PRKAR2A	cAMP-dependent protein kinase type II-alpha regulatory subunit	1.8	<0.001
Q13428	TCOF1	Treacle protein	1.8	0.001
A0A3B3IRI2	CTPS1	CTP synthase	1.8	0.001
P62753	RPS6	40S ribosomal protein S6	1.8	0.001
A0A3B3IRP5	CDC73	Parafibromin	1.8	0.002
P11802	CDK4	Cyclin-dependent kinase 4	1.8	0.003
B4DDF4	CNN2	Calponin	1.8	0.003
Q9UPN7	PPP6R1	Serine/threonine-protein phosphatase 6 regulatory subunit 1	1.8	0.003
P43490	NAMPT	Nicotinamide phosphoribosyltransferase	1.8	0.003
P49790	NUP153	Nuclear pore complex protein Nup153	1.8	0.004
Q13561	DCTN2	Dynactin subunit 2	1.8	0.005
Q9BZE4	GTPBP4	Nucleolar GTP-binding protein 1	1.8	0.005
O00154	ACOT7	Cytosolic acyl coenzyme A thioester hydrolase	1.8	0.006
M0QXL5	FBL	rRNA 2'-O-methyltransferase fibrillarin	1.8	0.008
Q6P2E9	EDC4	Enhancer of mRNA-decapping protein 4	1.8	0.008
Q14152	EIF3A	Eukaryotic translation initiation factor 3 subunit A	1.8	0.009
Q9BVP2	GNL3	Guanine nucleotide-binding protein-like 3	1.8	0.013
Q99959	PKP2	Plakophilin-2	1.8	0.017
Q14008	CKAP5	Cytoskeleton-associated protein 5	1.8	0.017
P25205	MCM3	DNA replication licensing factor MCM3	1.8	0.022
Q9NQX3	GPHN	Gephyrin [Includes: Molybdopterin adenylyltransferase	1.8	0.026
P82673	MRPS35	28S ribosomal protein S35, mitochondrial	1.8	0.030
P52594	AGFG1	Arf-GAP domain and FG repeat-containing protein 1	1.8	0.034
Q15084	PDIA6	Protein disulfide-isomerase A6	1.8	0.036
Q6P9B6	MEAK7	MTOR-associated protein MEAK7	1.8	0.036
Q9BR76	CORO1B	Coronin-1B	1.8	0.037
O60832	DKC1	H/ACA ribonucleoprotein complex subunit DKC1	1.8	0.037
Q92973	TNPO1	Transportin-1	1.8	0.040
P30154	PPP2R1B	Serine/threonine-protein phosphatase 2A 65 kDa regulatory subunit A beta isoform	1.8	0.041
O76021	RSL1D1	Ribosomal L1 domain-containing protein 1	1.8	0.050
Q6PKG0	LARP1	La-related protein 1	1.7	<0.001
Q9BV20	MRI1	Methylthioribose-1-phosphate isomerase	1.7	<0.001
Q9H7E9	C8orf33	UPF0488 protein C8orf33	1.7	<0.001
Q00796	SORD	Sorbitol dehydrogenase	1.7	<0.001
Q9H0B6	KLC2	Kinesin light chain 2	1.7	0.001
X1WI28	RPL10	60S ribosomal protein L10	1.7	0.001
P30566	ADSL	Adenylosuccinate lyase	1.7	0.001
Q99497	PARK7	Parkinson disease protein 7	1.7	0.002
P51553	IDH3G	Isocitrate dehydrogenase [NAD] subunit gamma, mitochondrial	1.7	0.002
P12004	PCNA	Proliferating cell nuclear antigen	1.7	0.002
P50502	ST13	Hsc70-interacting protein	1.7	0.002
Q99615	DNAJC7	DnaJ homolog subfamily C member 7	1.7	0.003
O60684	KPNA6	Importin subunit alpha-7	1.7	0.004
Q14694	USP10	Ubiquitin carboxyl-terminal hydrolase 10	1.7	0.004
Q15417	CNN3	Calponin-3	1.7	0.005
Q9UG63	ABCF2	ATP-binding cassette sub-family F member 2	1.7	0.006
Q9UHI6	DDX20	Probable ATP-dependent RNA helicase DDX20	1.7	0.006
O00178	GTPBP1	GTP-binding protein 1	1.7	0.006
O15160	POLR1C	DNA-directed RNA polymerases I and III subunit RPAC1	1.7	0.006
P46060	RANGAP1	Ran GTPase-activating protein 1	1.7	0.012
Q92667	AKAP1	A-kinase anchor protein 1, mitochondrial	1.7	0.014
Q8IY81	FTSJ3	pre-rRNA 2'-O-ribose RNA methyltransferase FTSJ3	1.7	0.016
Q12849	GRSF1	G-rich sequence factor 1	1.7	0.018
P42765	ACAA2	3-ketoacyl-CoA thiolase, mitochondrial	1.7	0.022
O75533	SF3B1	Splicing factor 3B subunit 1	1.7	0.029
A0A0A0MRT6	ABI1	Abl interactor 1	1.7	0.030
Q9Y2X3	NOP58	Nucleolar protein 58	1.7	0.032
P14174	MIF	Macrophage migration inhibitory factor	1.7	0.035
P62906	RPL10A	60S ribosomal protein L10a	1.7	0.043
Q9UGV2	NDRG3	Protein NDRG3	1.7	0.045
Q96P70	IPO9	Importin-9	1.7	0.048
Q3ZCQ8	TIMM50	Mitochondrial import inner membrane translocase subunit TIM50	1.7	0.048
A0A0C4DGQ6	RPRD1A	Regulation of nuclear pre-mRNA domain-containing protein 1A	1.6	<0.001
O95816	BAG2	BAG family molecular chaperone regulator 2	1.6	<0.001
Q12955	ANK3	Ankyrin-3	1.6	<0.001
O75569	PRKRA	Interferon-inducible double-stranded RNA-dependent protein kinase activator A	1.6	<0.001
Q15365	PCBP1	Poly(rC)-binding protein 1	1.6	<0.001
Q9UQ80	PA2G4	Proliferation-associated protein 2G4	1.6	<0.001
P13797	PLS3	Plastin-3	1.6	<0.001
Q9BTE6	AARSD1	Alanyl-tRNA editing protein Aarsd1	1.6	<0.001
Q9UNY4	TTF2	Transcription termination factor 2	1.6	0.001
P33993	MCM7	DNA replication licensing factor MCM7	1.6	0.001
O00170	AIP	AH receptor-interacting protein	1.6	0.002
P30153	PPP2R1A	Serine/threonine-protein phosphatase 2A 65 kDa regulatory subunit A alpha isoform	1.6	0.002
Q01469	FABP5	Fatty acid-binding protein 5	1.6	0.002
Q9H2G2	SLK	STE20-like serine/threonine-protein kinase	1.6	0.003
P62241	RPS8	40S ribosomal protein S8	1.6	0.003
Q8NC51	SERBP1	Plasminogen activator inhibitor 1 RNA-binding protein	1.6	0.003
A0A0G2JH68	DIAPH1	Protein diaphanous homolog 1	1.6	0.003
Q8WXF1	PSPC1	Paraspeckle component 1	1.6	0.004
Q8IWX8	CHERP	Calcium homeostasis endoplasmic reticulum protein	1.6	0.004
P49588	AARS1	Alanine--tRNA ligase, cytoplasmic	1.6	0.004
P53618	COPB1	Coatomer subunit beta	1.6	0.004
Q9C0C9	UBE2O	(E3-independent) E2 ubiquitin-conjugating enzyme	1.6	0.005
P26368	U2AF2	Splicing factor U2AF 65 kDa subunit	1.6	0.006
Q15185	PTGES3	Prostaglandin E synthase 3	1.6	0.006
A0A1C7CYX9	DPYSL2	Dihydropyrimidinase-related protein 2	1.6	0.006
Q9BUF5	TUBB6	Tubulin beta-6 chain	1.6	0.006
Q9Y450	HBS1L	HBS1-like protein	1.6	0.008
P40937	RFC5	Replication factor C subunit 5	1.6	0.009
P47897	QARS1	Glutamine--tRNA ligase	1.6	0.009
P09543	CNP	2',3'-cyclic-nucleotide 3'-phosphodiesterase	1.6	0.010
Q8WWY3	PRPF31	U4/U6 small nuclear ribonucleoprotein Prp31	1.6	0.010
O43395	PRPF3	U4/U6 small nuclear ribonucleoprotein Prp3	1.6	0.011
Q2TAY7	SMU1	WD40 repeat-containing protein SMU1	1.6	0.012
O95861	BPNT1	3'(2'),5'-bisphosphate nucleotidase 1	1.6	0.014
Q5JRX3	PITRM1	Presequence protease, mitochondrial	1.6	0.014
O75152	ZC3H11A	Zinc finger CCCH domain-containing protein 11A	1.6	0.015
O75131	CPNE3	Copine-3	1.6	0.016
Q96RP9	GFM1	Elongation factor G, mitochondrial	1.6	0.016
Q9BWD1	ACAT2	Acetyl-CoA acetyltransferase, cytosolic	1.6	0.017
P08754	GNAI3	Guanine nucleotide-binding protein G(i) subunit alpha	1.6	0.017
Q92879	CELF1	CUGBP Elav-like family member 1	1.6	0.030
P54646	PRKAA2	5'-AMP-activated protein kinase catalytic subunit alpha-2	1.6	0.037
P42566	EPS15	Epidermal growth factor receptor substrate 15	1.6	0.046
Q6YN16	HSDL2	Hydroxysteroid dehydrogenase-like protein 2	1.5	<0.001
Q5JSZ5	PRRC2B	Protein PRRC2B	1.5	<0.001
Q8N0X7	SPART	Spartin	1.5	<0.001
Q6Y7W6	GIGYF2	GRB10-interacting GYF protein 2	1.5	<0.001
P26640	VARS1	Valine--tRNA ligase	1.5	0.001
O15371	EIF3D	Eukaryotic translation initiation factor 3 subunit D	1.5	0.001
O14802	POLR3A	DNA-directed RNA polymerase III subunit RPC1	1.5	0.001
P22102	GART	Trifunctional purine biosynthetic protein adenosine-3 [Includes: Phosphoribosylamine--glycine ligase	1.5	0.001
P62873	GNB1	Guanine nucleotide-binding protein G(I)/G(S)/G(T) subunit beta-1	1.5	0.001
Q96S44	TP53RK	EKC/KEOPS complex subunit TP53RK	1.5	0.002
O43159	RRP8	Ribosomal RNA-processing protein 8	1.5	0.002
M0R0F0	RPS5	40S ribosomal protein S5	1.5	0.003
F8WB06	ATXN2	Ataxin-2	1.5	0.003
Q15003	NCAPH	Condensin complex subunit 2	1.5	0.003
P05198	EIF2S1	Eukaryotic translation initiation factor 2 subunit 1	1.5	0.003
Q15042	RAB3GAP1	Rab3 GTPase-activating protein catalytic subunit	1.5	0.003
P78344	EIF4G2	Eukaryotic translation initiation factor 4 gamma 2	1.5	0.004
O95336	PGLS	6-phosphogluconolactonase	1.5	0.004
P62244	RPS15A	40S ribosomal protein S15a	1.5	0.005
O95573	ACSL3	Long-chain-fatty-acid--CoA ligase 3	1.5	0.005
P55795	HNRNPH2	Heterogeneous nuclear ribonucleoprotein H2	1.5	0.005
P55081	MFAP1	Microfibrillar-associated protein 1	1.5	0.007
Q9UBT2	UBA2	SUMO-activating enzyme subunit 2	1.5	0.007
Q13136	PPFIA1	Liprin-alpha-1	1.5	0.008
A0A0G2JNZ2	SCRIB	Protein scribble homolog	1.5	0.009
Q9NQT5	EXOSC3	Exosome complex component RRP40	1.5	0.010
Q9UJC3	HOOK1	Protein Hook homolog 1	1.5	0.014
A1X283	SH3PXD2B	SH3 and PX domain-containing protein 2B	1.5	0.014
Q8N1G4	LRRC47	Leucine-rich repeat-containing protein 47	1.5	0.014
Q86Y56	DNAAF5	Dynein assembly factor 5, axonemal	1.5	0.015
B4E1N1	ARMC6	Armadillo repeat-containing protein 6	1.5	0.016
Q00610	CLTC	Clathrin heavy chain 1	1.5	0.017
P20042	EIF2S2	Eukaryotic translation initiation factor 2 subunit 2	1.5	0.017
P04637	TP53	Cellular tumor antigen p53	1.5	0.018
P63165	SUMO1	Small ubiquitin-related modifier 1	1.5	0.018
Q9HB71	CACYBP	Calcyclin-binding protein	1.5	0.020
Q8WVM8	SCFD1	Sec1 family domain-containing protein 1	1.5	0.026
Q08J23	NSUN2	RNA cytosine C(5)-methyltransferase NSUN2	1.5	0.026
Q9Y696	CLIC4	Chloride intracellular channel protein 4	1.5	0.029
O75934	BCAS2	Pre-mRNA-splicing factor SPF27	1.5	0.031
Q8N9T8	KRI1	Protein KRI1 homolog	1.5	0.036
Q9BY32	ITPA	Inosine triphosphate pyrophosphatase	1.5	0.041
E9PGZ1	CALD1	Caldesmon	1.5	0.047
Q9BQG0	MYBBP1A	Myb-binding protein 1A	1.5	0.049
Q9NSD9	FARSB	Phenylalanine--tRNA ligase beta subunit	1.4	<0.001
Q8WUM4	PDCD6IP	Programmed cell death 6-interacting protein	1.4	<0.001
Q86TB9	PATL1	Protein PAT1 homolog 1	1.4	<0.001
O94826	TOMM70	Mitochondrial import receptor subunit TOM70	1.4	0.001
Q14151	SAFB2	Scaffold attachment factor B2	1.4	0.001
Q16658	FSCN1	Fascin	1.4	0.001
P17987	TCP1	T-complex protein 1 subunit alpha	1.4	0.001
Q96FW1	OTUB1	Ubiquitin thioesterase OTUB1	1.4	0.002
P25325	MPST	3-mercaptopyruvate sulfurtransferase	1.4	0.005
P52701	MSH6	DNA mismatch repair protein Msh6	1.4	0.011
Q7L2H7	EIF3M	Eukaryotic translation initiation factor 3 subunit M	1.4	0.015
O95782	AP2A1	AP-2 complex subunit alpha-1	1.4	0.020
Q96F45	ZNF503	Zinc finger protein 503	1.4	0.023
Q96N67	DOCK7	Dedicator of cytokinesis protein 7	1.4	0.023
P55265	ADAR	Double-stranded RNA-specific adenosine deaminase	1.4	0.023
Q9NR12	PDLIM7	PDZ and LIM domain protein 7	1.4	0.027
O00425	IGF2BP3	Insulin-like growth factor 2 mRNA-binding protein 3	1.4	0.031
Q8IYS1	PM20D2	Peptidase M20 domain-containing protein 2	1.4	0.031
Q9Y6G9	DYNC1LI1	Cytoplasmic dynein 1 light intermediate chain 1	1.4	0.034
Q9P258	RCC2	Protein RCC2	1.4	0.037
Q96I25	RBM17	Splicing factor 45	1.4	0.041
O75439	PMPCB	Mitochondrial-processing peptidase subunit beta	1.4	0.041
P62851	RPS25	40S ribosomal protein S25	1.4	0.041
P61163	ACTR1A	Alpha-centractin	1.4	0.042
Q99569	PKP4	Plakophilin-4	1.4	0.042
E9PD53	SMC4	Structural maintenance of chromosomes protein	1.4	0.044
P46821	MAP1B	Microtubule-associated protein 1B	1.4	0.049
P63173	RPL38	60S ribosomal protein L38	1.3	<0.001
Q96A33	CCDC47	Coiled-coil domain-containing protein 47	1.3	0.001
P11908	PRPS2	Ribose-phosphate pyrophosphokinase 2	1.3	0.001
Q16643	DBN1	Drebrin	1.3	0.001
P62195	PSMC5	26S proteasome regulatory subunit 8	1.3	0.001
P61158	ACTR3	Actin-related protein 3	1.3	0.001
Q13347	EIF3I	Eukaryotic translation initiation factor 3 subunit I	1.3	0.003
P46939	UTRN	Utrophin	1.3	0.003
P31948	STIP1	Stress-induced-phosphoprotein 1	1.3	0.003
O60343	TBC1D4	TBC1 domain family member 4	1.3	0.003
O75792	RNASEH2A	Ribonuclease H2 subunit A	1.3	0.004
P51114	FXR1	Fragile X mental retardation syndrome-related protein 1	1.3	0.004
Q8WYP5	AHCTF1	Protein ELYS	1.3	0.007
P13804	ETFA	Electron transfer flavoprotein subunit alpha, mitochondrial	1.3	0.007
Q9Y5A9	YTHDF2	YTH domain-containing family protein 2	1.3	0.007
Q14C86	GAPVD1	GTPase-activating protein and VPS9 domain-containing protein 1	1.3	0.007
P42696	RBM34	RNA-binding protein 34	1.3	0.007
P55060	CSE1L	Exportin-2	1.3	0.007
Q02252	ALDH6A1	Methylmalonate-semialdehyde dehydrogenase [acylating], mitochondrial	1.3	0.007
P63241	EIF5A	Eukaryotic translation initiation factor 5A-1	1.3	0.009
Q9Y2U8	LEMD3	Inner nuclear membrane protein Man1	1.3	0.011
P54136	RARS1	Arginine--tRNA ligase, cytoplasmic	1.3	0.011
Q96AG4	LRRC59	Leucine-rich repeat-containing protein 59	1.3	0.011
P46779	RPL28	60S ribosomal protein L28	1.3	0.012
Q9NZI8	IGF2BP1	Insulin-like growth factor 2 mRNA-binding protein 1	1.3	0.013
P45974	USP5	Ubiquitin carboxyl-terminal hydrolase 5	1.3	0.013
P35580	MYH10	Myosin-10	1.3	0.013
Q9BRX2	PELO	Protein pelota homolog	1.3	0.014
P49736	MCM2	DNA replication licensing factor MCM2	1.3	0.014
C9JRJ5	LIMD1	LIM domain-containing protein 1	1.3	0.014
P42285	MTREX	Exosome RNA helicase MTR4	1.3	0.016
H0Y5D5	CIZ1	Cip1-interacting zinc finger protein	1.3	0.017
Q9BPU6	DPYSL5	Dihydropyrimidinase-related protein 5	1.3	0.017
P18615	NELFE	Negative elongation factor E	1.3	0.018
Q14318	FKBP8	Peptidyl-prolyl cis-trans isomerase FKBP8	1.3	0.018
Q52LJ0	FAM98B	Protein FAM98B	1.3	0.020
P19367	HK1	Hexokinase-1	1.3	0.025
Q13509	TUBB3	Tubulin beta-3 chain	1.3	0.026
Q1KMD3	HNRNPUL2	Heterogeneous nuclear ribonucleoprotein U-like protein 2	1.3	0.026
P52272	HNRNPM	Heterogeneous nuclear ribonucleoprotein M	1.3	0.027
F8W0J6	NAP1L1	Nucleosome assembly protein 1-like 1	1.3	0.029
J3QR09	RPL19	Ribosomal protein L19	1.3	0.031
Q13526	PIN1	Peptidyl-prolyl cis-trans isomerase NIMA-interacting 1	1.3	0.031
Q9UBE0	SAE1	SUMO-activating enzyme subunit 1	1.3	0.034
Q9P0K7	RAI14	Ankycorbin	1.3	0.035
Q16543	CDC37	Hsp90 co-chaperone Cdc37	1.3	0.036
P62854	RPS26	40S ribosomal protein S26	1.3	0.041
Q9NR45	NANS	Sialic acid synthase	1.3	0.043
P25685	DNAJB1	DnaJ homolog subfamily B member 1	1.3	0.048
Q13045	FLII	Protein flightless-1 homolog	1.2	<0.001
Q9P2I0	CPSF2	Cleavage and polyadenylation specificity factor subunit 2	1.2	0.001
P42771	CDKN2A	Cyclin-dependent kinase inhibitor 2A	1.2	0.002
Q9HC35	EML4	Echinoderm microtubule-associated protein-like 4	1.2	0.003
A0A0A0MRM8	MYO6	Unconventional myosin-6	1.2	0.003
Q96C36	PYCR2	Pyrroline-5-carboxylate reductase 2	1.2	0.003
Q9UN37	VPS4A	Vacuolar protein sorting-associated protein 4A	1.2	0.004
Q9UMS4	PRPF19	Pre-mRNA-processing factor 19	1.2	0.006
Q9Y2Z0	SUGT1	Protein SGT1 homolog	1.2	0.008
P31939	ATIC	Bifunctional purine biosynthesis protein ATIC	1.2	0.008
K7EJL1	AP1M1	AP-1 complex subunit mu-1	1.2	0.008
F8VYE8	PPP1CC	Serine/threonine-protein phosphatase	1.2	0.008
Q9Y490	TLN1	Talin-1	1.2	0.009
Q96HC4	PDLIM5	PDZ and LIM domain protein 5	1.2	0.009
P98175	RBM10	RNA-binding protein 10	1.2	0.014
P49368	CCT3	T-complex protein 1 subunit gamma	1.2	0.015
P13489	RNH1	Ribonuclease inhibitor	1.2	0.017
Q9Y3S2	ZNF330	Zinc finger protein 330	1.2	0.017
Q9H0D6	XRN2	5'-3' exoribonuclease 2	1.2	0.018
Q12904	AIMP1	Aminoacyl tRNA synthase complex-interacting multifunctional protein 1	1.2	0.019
P36542	ATP5F1C	ATP synthase subunit gamma, mitochondrial	1.2	0.019
P36915	GNL1	Guanine nucleotide-binding protein-like 1	1.2	0.020
Q13907	IDI1	Isopentenyl-diphosphate Delta-isomerase 1	1.2	0.020
P37802	TAGLN2	Transgelin-2	1.2	0.021
A0A2R8YDQ9	SUCLA2	Succinate--CoA ligase [ADP-forming] subunit beta, mitochondrial	1.2	0.023
G5EA31	SEC24C	Protein transport protein Sec24C	1.2	0.027
Q07666	KHDRBS1	KH domain-containing, RNA-binding, signal transduction-associated protein 1	1.2	0.029
O43390	HNRNPR	Heterogeneous nuclear ribonucleoprotein R	1.2	0.029
P41240	CSK	Tyrosine-protein kinase CSK	1.2	0.033
Q9NW82	WDR70	WD repeat-containing protein 70	1.2	0.033
Q9NWH9	SLTM	SAFB-like transcription modulator	1.2	0.033
O94906	PRPF6	Pre-mRNA-processing factor 6	1.2	0.035
Q8WWQ0	PHIP	PH-interacting protein	1.2	0.035
P33176	KIF5B	Kinesin-1 heavy chain	1.2	0.036
P28838	LAP3	Cytosol aminopeptidase	1.2	0.037
P27540	ARNT	Aryl hydrocarbon receptor nuclear translocator	1.2	0.045
P39687	ANP32A	Acidic leucine-rich nuclear phosphoprotein 32 family member A	1.2	0.046
Q9BY44	EIF2A	Eukaryotic translation initiation factor 2A	1.2	0.047
P53384	NUBP1	Cytosolic Fe-S cluster assembly factor NUBP1	1.2	0.049
P29692	EEF1D	Elongation factor 1-delta	1.1	0.001
Q5JXI8	FHL1	Four and a half LIM domains protein 1	1.1	0.001
O43684	BUB3	Mitotic checkpoint protein BUB3	1.1	0.004
C9J0I9	ZC3HC1	Nuclear-interacting partner of ALK	1.1	0.004
Q13451	FKBP5	Peptidyl-prolyl cis-trans isomerase FKBP5	1.1	0.004
Q2TAM5	RELA	RELA protein	1.1	0.006
P43487	RANBP1	Ran-specific GTPase-activating protein	1.1	0.007
Q9H3K6	BOLA2	BolA-like protein 2	1.1	0.009
P30084	ECHS1	Enoyl-CoA hydratase, mitochondrial	1.1	0.009
P62701	RPS4X	40S ribosomal protein S4, X isoform	1.1	0.010
A0A1B0GWF2	STXBP1	Syntaxin-binding protein 1	1.1	0.010
P46778	RPL21	60S ribosomal protein L21	1.1	0.010
Q6ZN17	LIN28B	Protein lin-28 homolog B	1.1	0.010
P43246	MSH2	DNA mismatch repair protein Msh2	1.1	0.011
Q16527	CSRP2	Cysteine and glycine-rich protein 2	1.1	0.011
Q8IWS0	PHF6	PHD finger protein 6	1.1	0.012
Q5TDH0	DDI2	Protein DDI1 homolog 2	1.1	0.012
G3XAH6	PAPOLA	Poly(A) polymerase	1.1	0.012
P45973	CBX5	Chromobox protein homolog 5	1.1	0.012
Q15126	PMVK	Phosphomevalonate kinase	1.1	0.014
P84095	RHOG	Rho-related GTP-binding protein RhoG	1.1	0.014
Q02790	FKBP4	Peptidyl-prolyl cis-trans isomerase FKBP4	1.1	0.015
A0A087WXM6	RPL17	60S ribosomal protein L17	1.1	0.016
Q9UPQ9	TNRC6B	Trinucleotide repeat-containing gene 6B protein	1.1	0.017
M0R3C3	TECR	Very-long-chain enoyl-CoA reductase	1.1	0.017
Q99829	CPNE1	Copine-1	1.1	0.019
Q71RC2	LARP4	La-related protein 4	1.1	0.019
P60660	MYL6	Myosin light polypeptide 6	1.1	0.021
P49207	RPL34	60S ribosomal protein L34	1.1	0.021
Q10567	AP1B1	AP-1 complex subunit beta-1	1.1	0.023
P63092	GNAS	Guanine nucleotide-binding protein G(s) subunit alpha isoforms short	1.1	0.025
Q9UN86	G3BP2	Ras GTPase-activating protein-binding protein 2	1.1	0.027
P51116	FXR2	Fragile X mental retardation syndrome-related protein 2	1.1	0.027
H0YNW5	DUT	Deoxyuridine 5'-triphosphate nucleotidohydrolase	1.1	0.028
H7C5E4	XRN1	5'-3' exoribonuclease 1	1.1	0.031
P52294	KPNA1	Importin subunit alpha-5	1.1	0.031
P15880	RPS2	40S ribosomal protein S2	1.1	0.031
P31946	YWHAB	14-3-3 protein beta/alpha	1.1	0.036
O14893	GEMIN2	Gem-associated protein 2	1.1	0.037
K4DI93	CUL4B	Cullin 4B, isoform CRA_e	1.1	0.042
Q9UKM9	RALY	RNA-binding protein Raly	1.1	0.043
P54578	USP14	Ubiquitin carboxyl-terminal hydrolase 14	1.1	0.046
C9J0J7	PFN2	Profilin	1.1	0.046
Q8IYI6	EXOC8	Exocyst complex component 8	1.1	0.048
P50991	CCT4	T-complex protein 1 subunit delta	1.0	0.007
P32322	PYCR1	Pyrroline-5-carboxylate reductase 1, mitochondrial	1.0	0.008
P07814	EPRS1	Bifunctional glutamate/proline--tRNA ligase	1.0	0.011
Q9NUQ9	CYRIB	CYFIP-related Rac1 interactor B	1.0	0.013
Q16576	RBBP7	Histone-binding protein RBBP7	1.0	0.017
A0A087WY55	VTA1	Chromosome 6 open reading frame 55, isoform CRA_b	1.0	0.022
Q7RTV0	PHF5A	PHD finger-like domain-containing protein 5A	1.0	0.022
P38117	ETFB	Electron transfer flavoprotein subunit beta	1.0	0.024
B5MCF9	PES1	Pescadillo homolog	1.0	0.024
A0A2R8Y855	SMARCE1	SWI/SNF-related matrix-associated actin-dependent regulator of chromatin subfamily E member 1	1.0	0.027
Q9UJ83	HACL1	2-hydroxyacyl-CoA lyase 1	1.0	0.029
Q9BZE9	ASPSCR1	Tether containing UBX domain for GLUT4	1.0	0.034
P55884	EIF3B	Eukaryotic translation initiation factor 3 subunit B	1.0	0.036
Q15370	ELOB	Elongin-B	1.0	0.040
Q9ULC4	MCTS1	Malignant T-cell-amplified sequence 1	1.0	0.043
P26358	DNMT1	DNA	1.0	0.045
A0A024RAC6	ELOA	Elongin-A	1.0	0.047
**PRDX2 cysteine-dependent interactors**				
Q9C0C2	TNKS1BP1	182 kDa tankyrase-1-binding protein	6.4	<0.001
Q9Y6A5	TACC3	Transforming acidic coiled-coil-containing protein 3	5.2	<0.001
Q8NFC6	BOD1L1	Biorientation of chromosomes in cell division protein 1-like 1	5.2	<0.001
P07355	ANXA2	Annexin A2	5.1	<0.001
Q7Z434	MAVS	Mitochondrial antiviral-signaling protein	4.9	<0.001
Q9NQX3	GPHN	Gephyrin [Includes: Molybdopterin adenylyltransferase	4.9	<0.001
E7EVA0	MAP4	Microtubule-associated protein	4.9	<0.001
Q9UHV9	PFDN2	Prefoldin subunit 2	4.5	<0.001
Q9BQA1	WDR77	Methylosome protein 50	4.2	<0.001
O60547	GMDS	GDP-mannose 4,6 dehydratase	4.2	<0.001
Q9Y266	NUDC	Nuclear migration protein nudC	4.1	<0.001
A0AVT1	UBA6	Ubiquitin-like modifier-activating enzyme 6	4.1	<0.001
P11802	CDK4	Cyclin-dependent kinase 4	4.1	<0.001
Q32MZ4	LRRFIP1	Leucine-rich repeat flightless-interacting protein 1	4.1	<0.001
Q9NPH2	ISYNA1	Inositol-3-phosphate synthase 1	4.0	<0.001
P22061	PCMT1	Protein-L-isoaspartate(D-aspartate) O-methyltransferase	4.0	<0.001
Q9HBH5	RDH14	Retinol dehydrogenase 14	4.0	0.004
P12955	PEPD	Xaa-Pro dipeptidase	3.9	0.001
P61289	PSME3	Proteasome activator complex subunit 3	3.9	0.005
B7Z7F3	RANBP3	Ran-binding protein 3	3.8	<0.001
O14744	PRMT5	Protein arginine N-methyltransferase 5	3.7	<0.001
A6NGP5	JPT2	Jupiter microtubule-associated homolog 2	3.7	<0.001
Q9ULW0	TPX2	Targeting protein for Xklp2	3.7	0.001
P46821	MAP1B	Microtubule-associated protein 1B	3.6	<0.001
Q14247	CTTN	Src substrate cortactin	3.6	<0.001
O00483	NDUFA4	Cytochrome c oxidase subunit NDUFA4	3.5	<0.001
Q96EA4	SPDL1	Protein Spindly	3.5	0.002
Q5T6F2	UBAP2	Ubiquitin-associated protein 2	3.4	<0.001
Q9H773	DCTPP1	dCTP pyrophosphatase 1	3.3	<0.001
Q8NC51	SERBP1	Plasminogen activator inhibitor 1 RNA-binding protein	3.3	<0.001
Q5SW79	CEP170	Centrosomal protein of 170 kDa	3.2	<0.001
Q96KB5	PBK	Lymphokine-activated killer T-cell-originated protein kinase	3.2	0.002
Q9BRP1	PDCD2L	Programmed cell death protein 2-like	3.1	0.001
P06132	UROD	Uroporphyrinogen decarboxylase	3.1	0.004
P07910	HNRNPC	Heterogeneous nuclear ribonucleoproteins C1/C2	3.1	0.042
Q9NUQ3	TXLNG	Gamma-taxilin	3.0	0.002
Q86V48	LUZP1	Leucine zipper protein 1	2.9	<0.001
O43809	NUDT21	Cleavage and polyadenylation specificity factor subunit 5	2.9	0.001
Q9H2U2	PPA2	Inorganic pyrophosphatase 2, mitochondrial	2.9	0.001
Q9NUP9	LIN7C	Protein lin-7 homolog C	2.9	0.002
P40222	TXLNA	Alpha-taxilin	2.9	0.002
P61758	VBP1	Prefoldin subunit 3	2.9	0.004
O60664	PLIN3	Perilipin-3	2.9	0.008
H0YNW5	DUT	Deoxyuridine 5'-triphosphate nucleotidohydrolase	2.8	<0.001
E7EPN9	PRRC2C	Protein PRRC2C	2.8	<0.001
Q8N3X1	FNBP4	Formin-binding protein 4	2.8	0.001
Q13541	EIF4EBP1	Eukaryotic translation initiation factor 4E-binding protein 1	2.8	0.006
O75223	GGCT	Gamma-glutamylcyclotransferase	2.8	0.011
P19174	PLCG1	1-phosphatidylinositol 4,5-bisphosphate phosphodiesterase gamma-1	2.7	0.002
O75381	PEX14	Peroxisomal membrane protein PEX14	2.7	0.004
E9PGZ1	CALD1	Caldesmon	2.7	0.004
Q8IYS1	PM20D2	Peptidase M20 domain-containing protein 2	2.7	0.004
Q8IWB7	WDFY1	WD repeat and FYVE domain-containing protein 1	2.7	0.004
P10599	TXN	Thioredoxin	2.6	<0.001
Q9P258	RCC2	Protein RCC2	2.6	<0.001
P26599	PTBP1	Polypyrimidine tract-binding protein 1	2.6	<0.001
P30041	PRDX6	Peroxiredoxin-6	2.6	0.002
O43143	DHX15	Pre-mRNA-splicing factor ATP-dependent RNA helicase DHX15	2.6	0.002
K7EJQ8	HDHD2	Haloacid dehalogenase-like hydrolase domain-containing protein 2	2.6	0.004
P30740	SERPINB1	Leukocyte elastase inhibitor	2.6	0.005
Q8WVC2	RPS21	40S ribosomal protein S21	2.6	0.006
Q16204	CCDC6	Coiled-coil domain-containing protein 6	2.6	0.011
H3BRL3	UBFD1	Ubiquitin domain-containing protein UBFD1	2.6	0.014
P52888	THOP1	Thimet oligopeptidase	2.5	<0.001
J3KN29	PSMD9	26S proteasome non-ATPase regulatory subunit 9	2.5	<0.001
Q9Y570	PPME1	Protein phosphatase methylesterase 1	2.5	<0.001
O14929	HAT1	Histone acetyltransferase type B catalytic subunit	2.5	<0.001
P48634	PRRC2A	Protein PRRC2A	2.5	<0.001
Q9HA64	FN3KRP	Ketosamine-3-kinase	2.5	0.002
Q8TAQ2	SMARCC2	SWI/SNF complex subunit SMARCC2	2.5	0.003
A0A087WTM1	ROBO1	Roundabout homolog 1	2.5	0.004
Q6IQ49	SDE2	Replication stress response regulator SDE2	2.5	0.004
Q6WKZ4	RAB11FIP1	Rab11 family-interacting protein 1	2.5	0.005
Q9P2B4	CTTNBP2NL	CTTNBP2 N-terminal-like protein	2.5	0.006
Q99598	TSNAX	Translin-associated protein X	2.5	0.008
P49903	SEPHS1	Selenide, water dikinase 1	2.5	0.009
K7ELV2	SEH1L	Nucleoporin SEH1	2.5	0.010
Q13542	EIF4EBP2	Eukaryotic translation initiation factor 4E-binding protein 2	2.5	0.045
F5GZS6	SLC3A2	4F2 cell-surface antigen heavy chain	2.4	<0.001
Q9BXJ9	NAA15	N-alpha-acetyltransferase 15, NatA auxiliary subunit	2.4	0.003
P35520	CBS	Cystathionine beta-synthase	2.3	<0.001
Q13428	TCOF1	Treacle protein	2.3	<0.001
P42771	CDKN2A	Cyclin-dependent kinase inhibitor 2A	2.3	<0.001
Q15019	SEPTIN2	Septin-2	2.3	0.009
Q9Y3B7	MRPL11	39S ribosomal protein L11, mitochondrial	2.3	0.017
Q8N6M0	OTUD6B	Deubiquitinase OTUD6B	2.3	0.017
Q49A26	GLYR1	Putative oxidoreductase GLYR1	2.3	0.037
P52907	CAPZA1	F-actin-capping protein subunit alpha-1	2.2	<0.001
Q13547	HDAC1	Histone deacetylase 1	2.2	0.002
Q14257	RCN2	Reticulocalbin-2	2.2	0.004
Q9UQE7	SMC3	Structural maintenance of chromosomes protein 3	2.2	0.006
Q5JRA6	MIA3	Transport and Golgi organization protein 1 homolog	2.2	0.007
E9PS17	SCYL1	N-terminal kinase-like protein	2.2	0.008
O00629	KPNA4	Importin subunit alpha-3	2.2	0.009
O94992	HEXIM1	Protein HEXIM1	2.2	0.010
Q96F45	ZNF503	Zinc finger protein 503	2.2	0.013
E7EV99	ADD1	Alpha-adducin	2.2	0.014
P38159	RBMX	RNA-binding motif protein, X chromosome	2.2	0.014
Q96GD0	PDXP	Pyridoxal phosphate phosphatase	2.2	0.015
E9PM92	C11orf58	Small acidic protein	2.2	0.017
A4D1S0	KLRG2	Killer cell lectin-like receptor subfamily G member 2	2.2	0.027
C9J0I9	ZC3HC1	Nuclear-interacting partner of ALK	2.1	<0.001
P35237	SERPINB6	Serpin B6	2.1	<0.001
P00492	HPRT1	Hypoxanthine-guanine phosphoribosyltransferase	2.1	0.004
Q9UBS4	DNAJB11	DnaJ homolog subfamily B member 11	2.1	0.014
Q9Y2R9	MRPS7	28S ribosomal protein S7, mitochondrial	2.1	0.015
O95104	SCAF4	SR-related and CTD-associated factor 4	2.1	0.016
F5H8D7	XRCC1	DNA repair protein XRCC1	2.1	0.016
Q9NVP1	DDX18	ATP-dependent RNA helicase DDX18	2.1	0.018
Q86WA6	BPHL	Valacyclovir hydrolase	2.1	0.026
Q6PKG0	LARP1	La-related protein 1	2.0	<0.001
Q06124	PTPN11	Tyrosine-protein phosphatase non-receptor type 11	2.0	<0.001
B1AK87	CAPZB	F-actin-capping protein subunit beta	2.0	<0.001
P54646	PRKAA2	5'-AMP-activated protein kinase catalytic subunit alpha-2	2.0	0.007
P41250	GARS1	Glycine--tRNA ligase	2.0	0.013
Q9H3S7	PTPN23	Tyrosine-protein phosphatase non-receptor type 23	2.0	0.014
Q9NXF7	DCAF16	DDB1- and CUL4-associated factor 16	2.0	0.016
O94903	PLPBP	Pyridoxal phosphate homeostasis protein	2.0	0.017
P29373	CRABP2	Cellular retinoic acid-binding protein 2	2.0	0.018
Q8IVM0	CCDC50	Coiled-coil domain-containing protein 50	2.0	0.021
O15143	ARPC1B	Actin-related protein 2/3 complex subunit 1B	2.0	0.025
P23588	EIF4B	Eukaryotic translation initiation factor 4B	2.0	0.049
Q16576	RBBP7	Histone-binding protein RBBP7	1.9	<0.001
Q93034	CUL5	Cullin-5	1.9	0.001
Q9C0C9	UBE2O	(E3-independent) E2 ubiquitin-conjugating enzyme	1.9	0.001
P52732	KIF11	Kinesin-like protein KIF11	1.9	0.002
Q8N6T3	ARFGAP1	ADP-ribosylation factor GTPase-activating protein 1	1.9	0.016
Q96K76	USP47	Ubiquitin carboxyl-terminal hydrolase 47	1.9	0.020
Q14240	EIF4A2	Eukaryotic initiation factor 4A-II	1.9	0.020
A1X283	SH3PXD2B	SH3 and PX domain-containing protein 2B	1.9	0.026
Q9Y3D0	CIAO2B	Cytosolic iron-sulfur assembly component 2B	1.9	0.031
P42574	CASP3	Caspase-3	1.9	0.042
Q16531	DDB1	DNA damage-binding protein 1	1.8	<0.001
P51452	DUSP3	Dual specificity protein phosphatase 3	1.8	<0.001
Q9Y3F4	STRAP	Serine-threonine kinase receptor-associated protein	1.8	<0.001
P31153	MAT2A	S-adenosylmethionine synthase isoform type-2	1.8	0.001
A0A1B0GW38	CBL	E3 ubiquitin-protein ligase CBL	1.8	0.003
Q9Y6Y8	SEC23IP	SEC23-interacting protein	1.8	0.005
Q12948	FOXC1	Forkhead box protein C1	1.8	0.014
Q12800	TFCP2	Alpha-globin transcription factor CP2	1.8	0.024
Q9HCN8	SDF2L1	Stromal cell-derived factor 2-like protein 1	1.8	0.026
Q2M1P5	KIF7	Kinesin-like protein KIF7	1.8	0.026
E9PQV9	DCUN1D5	DCN1-like protein	1.8	0.032
Q9BT25	HAUS8	HAUS augmin-like complex subunit 8	1.8	0.038
O60826	CCDC22	Coiled-coil domain-containing protein 22	1.8	0.047
Q06203	PPAT	Amidophosphoribosyltransferase	1.7	<0.001
Q96F86	EDC3	Enhancer of mRNA-decapping protein 3	1.7	0.004
Q9UBE0	SAE1	SUMO-activating enzyme subunit 1	1.7	0.006
A0A087X054	HYOU1	Hypoxia up-regulated protein 1	1.7	0.008
A0A3B3ISG5	IDE	Insulin-degrading enzyme	1.7	0.014
Q6P2Q9	PRPF8	Pre-mRNA-processing-splicing factor 8	1.7	0.014
P52788	SMS	Spermine synthase	1.7	0.023
E9PGT1	TSN	Component 3 of promoter of RISC	1.7	0.031
O43815	STRN	Striatin	1.7	0.039
Q6UN15	FIP1L1	Pre-mRNA 3'-end-processing factor FIP1	1.7	0.042
Q9BXP5	SRRT	Serrate RNA effector molecule homolog	1.7	0.045
Q92783	STAM	Signal transducing adapter molecule 1	1.7	0.048
Q5QPM7	PSMF1	Proteasome inhibitor PI31 subunit	1.6	<0.001
Q9UKG1	APPL1	DCC-interacting protein 13-alpha	1.6	<0.001
Q14C86	GAPVD1	GTPase-activating protein and VPS9 domain-containing protein 1	1.6	0.001
Q15393	SF3B3	Splicing factor 3B subunit 3	1.6	0.001
Q9UN37	VPS4A	Vacuolar protein sorting-associated protein 4A	1.6	0.004
O75663	TIPRL	TIP41-like protein	1.6	0.007
A0A087WTZ5	UBXN1	UBX domain-containing protein 1	1.6	0.010
P63000	RAC1	Ras-related C3 botulinum toxin substrate 1	1.6	0.018
O00165	HAX1	HCLS1-associated protein X-1	1.6	0.025
P49750	YLPM1	YLP motif-containing protein 1	1.6	0.038
O75533	SF3B1	Splicing factor 3B subunit 1	1.6	0.039
Q9Y265	RUVBL1	RuvB-like 1	1.5	0.001
P11413	G6PD	Glucose-6-phosphate 1-dehydrogenase	1.5	0.002
E9PLA9	CAPRIN1	Caprin-1	1.5	0.002
A0A087X2B5	BSG	Basigin	1.5	0.005
Q07666	KHDRBS1	KH domain-containing, RNA-binding, signal transduction-associated protein 1	1.5	0.010
Q9NYZ3	GTSE1	G2 and S phase-expressed protein 1	1.5	0.010
Q9BWJ5	SF3B5	Splicing factor 3B subunit 5	1.5	0.014
Q92667	AKAP1	A-kinase anchor protein 1, mitochondrial	1.5	0.022
P61106	RAB14	Ras-related protein Rab-14	1.5	0.035
O95347	SMC2	Structural maintenance of chromosomes protein 2	1.5	0.041
O60493	SNX3	Sorting nexin-3	1.5	0.047
Q96A33	CCDC47	Coiled-coil domain-containing protein 47	1.4	<0.001
Q8N5F7	NKAP	NF-kappa-B-activating protein	1.4	<0.001
Q13045	FLII	Protein flightless-1 homolog	1.4	<0.001
A0A0A0MRM8	MYO6	Unconventional myosin-6	1.4	0.001
Q92598	HSPH1	Heat shock protein 105 kDa	1.4	0.001
Q96HC4	PDLIM5	PDZ and LIM domain protein 5	1.4	0.002
O60343	TBC1D4	TBC1 domain family member 4	1.4	0.002
P20020	ATP2B1	Plasma membrane calcium-transporting ATPase 1	1.4	0.002
A0A024RCR6	BAG6	BCL2-associated athanogene 6	1.4	0.003
Q5BKZ1	ZNF326	DBIRD complex subunit ZNF326	1.4	0.003
Q9UNM6	PSMD13	26S proteasome non-ATPase regulatory subunit 13	1.4	0.003
P61981	YWHAG	14-3-3 protein gamma	1.4	0.005
P62333	PSMC6	26S proteasome regulatory subunit 10B	1.4	0.006
P15374	UCHL3	Ubiquitin carboxyl-terminal hydrolase isozyme L3	1.4	0.006
O00170	AIP	AH receptor-interacting protein	1.4	0.007
P35998	PSMC2	26S proteasome regulatory subunit 7	1.4	0.008
P50213	IDH3A	Isocitrate dehydrogenase [NAD] subunit alpha, mitochondrial	1.4	0.012
P31946	YWHAB	14-3-3 protein beta/alpha	1.4	0.013
Q7Z6Z7	HUWE1	E3 ubiquitin-protein ligase HUWE1	1.4	0.018
Q9UPN7	PPP6R1	Serine/threonine-protein phosphatase 6 regulatory subunit 1	1.4	0.024
O94905	ERLIN2	Erlin-2	1.4	0.028

O95801	TTC4	Tetratricopeptide repeat protein 4	1.4	0.030
Q96T23	RSF1	Remodeling and spacing factor 1	1.4	0.033
O14974	PPP1R12A	Protein phosphatase 1 regulatory subunit 12A	1.4	0.038
P13797	PLS3	Plastin-3	1.3	<0.001
P62191	PSMC1	26S proteasome regulatory subunit 4	1.3	0.001
Q9Y230	RUVBL2	RuvB-like 2	1.3	0.001
P25786	PSMA1	Proteasome subunit alpha type-1	1.3	0.001
O00151	PDLIM1	PDZ and LIM domain protein 1	1.3	0.002
Q7LBC6	KDM3B	Lysine-specific demethylase 3B	1.3	0.004
Q96D09	GPRASP2	G-protein coupled receptor-associated sorting protein 2	1.3	0.006
P08240	SRPRA	Signal recognition particle receptor subunit alpha	1.3	0.011
P55072	VCP	Transitional endoplasmic reticulum ATPase	1.3	0.012
Q9UJV9	DDX41	Probable ATP-dependent RNA helicase DDX41	1.3	0.021
Q92769	HDAC2	Histone deacetylase 2	1.3	0.023
P41214	EIF2D	Eukaryotic translation initiation factor 2D	1.3	0.024
A0MZ66	SHTN1	Shootin-1	1.3	0.024
A0A1C7CYX9	DPYSL2	Dihydropyrimidinase-related protein 2	1.3	0.025
Q15417	CNN3	Calponin-3	1.3	0.027
O00267	SUPT5H	Transcription elongation factor SPT5	1.3	0.027
P22314	UBA1	Ubiquitin-like modifier-activating enzyme 1	1.3	0.029
Q15276	RABEP1	Rab GTPase-binding effector protein 1	1.3	0.039
Q9H6S0	YTHDC2	3'-5' RNA helicase YTHDC2	1.3	0.044
Q9H0U4	RAB1B	Ras-related protein Rab-1B	1.3	0.046
Q9H9B4	SFXN1	Sideroflexin-1	1.2	0.001
Q6NUK1	SLC25A24	Calcium-binding mitochondrial carrier protein SCaMC-1	1.2	0.001
Q9UKX7	NUP50	Nuclear pore complex protein Nup50	1.2	0.002
Q15046	KARS1	Lysine--tRNA ligase	1.2	0.003
O00487	PSMD14	26S proteasome non-ATPase regulatory subunit 14	1.2	0.005
P06737	PYGL	Glycogen phosphorylase, liver form	1.2	0.005
Q9UJZ1	STOML2	Stomatin-like protein 2, mitochondrial	1.2	0.005
Q9UNF1	MAGED2	Melanoma-associated antigen D2	1.2	0.006
Q7Z4H7	HAUS6	HAUS augmin-like complex subunit 6	1.2	0.006
Q9UHD1	CHORDC1	Cysteine and histidine-rich domain-containing protein 1	1.2	0.010
P22695	UQCRC2	Cytochrome b-c1 complex subunit 2, mitochondrial	1.2	0.011
Q9GZT9	EGLN1	Egl nine homolog 1	1.2	0.012
Q9Y5A9	YTHDF2	YTH domain-containing family protein 2	1.2	0.013
O00273	DFFA	DNA fragmentation factor subunit alpha	1.2	0.014
P58546	MTPN	Myotrophin	1.2	0.020
P42677	RPS27	40S ribosomal protein S27	1.2	0.026
S4R3H4	ACIN1	Apoptotic chromatin condensation inducer in the nucleus	1.2	0.027
Q9BTE3	MCMBP	Mini-chromosome maintenance complex-binding protein	1.2	0.027
M0QY97	ZC3H4	Zinc finger CCCH domain-containing protein 4	1.2	0.028
Q14739	LBR	Delta(14)-sterol reductase LBR	1.2	0.030
P52701	MSH6	DNA mismatch repair protein Msh6	1.2	0.031
P22234	PAICS	Multifunctional protein ADE2 [Includes: Phosphoribosylaminoimidazole-succinocarboxamide synthase	1.2	0.035
P55265	ADAR	Double-stranded RNA-specific adenosine deaminase	1.2	0.040
Q8NI27	THOC2	THO complex subunit 2	1.2	0.046
P49790	NUP153	Nuclear pore complex protein Nup153	1.2	0.048
Q14137	BOP1	Ribosome biogenesis protein BOP1	1.2	0.049
P00387	CYB5R3	NADH-cytochrome b5 reductase 3	1.1	0.001
Q6P4A7	SFXN4	Sideroflexin-4	1.1	0.002
Q9Y295	DRG1	Developmentally-regulated GTP-binding protein 1	1.1	0.003
Q14566	MCM6	DNA replication licensing factor MCM6	1.1	0.003
P00338	LDHA	L-lactate dehydrogenase A chain	1.1	0.005
P49593	PPM1F	Protein phosphatase 1F	1.1	0.005
A0A0C4DGX4	CUL1	Cullin-1	1.1	0.006
Q13200	PSMD2	26S proteasome non-ATPase regulatory subunit 2	1.1	0.006
P22570	FDXR	NADPH:adrenodoxin oxidoreductase, mitochondrial	1.1	0.007
P13861	PRKAR2A	cAMP-dependent protein kinase type II-alpha regulatory subunit	1.1	0.007
E9PLK3	NPEPPS	Aminopeptidase	1.1	0.009
P05023	ATP1A1	Sodium/potassium-transporting ATPase subunit alpha-1	1.1	0.009
O95573	ACSL3	Long-chain-fatty-acid--CoA ligase 3	1.1	0.009
Q9NZB2	FAM120A	Constitutive coactivator of PPAR-gamma-like protein 1	1.1	0.012
Q96P70	IPO9	Importin-9	1.1	0.013
H0YDU8	PPP5C	Serine/threonine-protein phosphatase	1.1	0.014
Q04917	YWHAH	14-3-3 protein eta	1.1	0.016
Q96KR1	ZFR	Zinc finger RNA-binding protein	1.1	0.016
Q6UWP7	LCLAT1	Lysocardiolipin acyltransferase 1	1.1	0.018
Q8WWK9	CKAP2	Cytoskeleton-associated protein 2	1.1	0.021
Q9UIG0	BAZ1B	Tyrosine-protein kinase BAZ1B	1.1	0.024
A0A087WWP4	RBM15	RNA-binding protein 15	1.1	0.024
Q99504	EYA3	Eyes absent homolog 3	1.1	0.025
Q9H936	SLC25A22	Mitochondrial glutamate carrier 1	1.1	0.028
Q14694	USP10	Ubiquitin carboxyl-terminal hydrolase 10	1.1	0.028
Q9UBT2	UBA2	SUMO-activating enzyme subunit 2	1.1	0.038
P56385	ATP5ME	ATP synthase subunit e, mitochondrial	1.1	0.038
Q9NZL9	MAT2B	Methionine adenosyltransferase 2 subunit beta	1.1	0.040
O60832	DKC1	H/ACA ribonucleoprotein complex subunit DKC1	1.1	0.040
Q8WXF1	PSPC1	Paraspeckle component 1	1.1	0.041
P49588	AARS1	Alanine--tRNA ligase, cytoplasmic	1.1	0.043
P98175	RBM10	RNA-binding protein 10	1.1	0.049
P78346	RPP30	Ribonuclease P protein subunit p30	1.0	0.006
G8JLH9	STAT3	Signal transducer and activator of transcription	1.0	0.009
A0A087WUX8	APOOL	MICOS complex subunit	1.0	0.010
Q92597	NDRG1	Protein NDRG1	1.0	0.011
P46939	UTRN	Utrophin	1.0	0.014
Q2NKX8	ERCC6L	DNA excision repair protein ERCC-6-like	1.0	0.016
P09936	UCHL1	Ubiquitin carboxyl-terminal hydrolase isozyme L1	1.0	0.017
Q86YP4	GATAD2A	Transcriptional repressor p66-alpha	1.0	0.024
Q16512	PKN1	Serine/threonine-protein kinase N1	1.0	0.025
P60228	EIF3E	Eukaryotic translation initiation factor 3 subunit E	1.0	0.026
Q8ND82	ZNF280C	Zinc finger protein 280C	1.0	0.035
O95684	CEP43	Centrosomal protein 43	1.0	0.038
O43765	SGTA	Small glutamine-rich tetratricopeptide repeat-containing protein alpha	1.0	0.044
O43242	PSMD3	26S proteasome non-ATPase regulatory subunit 3	1.0	0.048
**PRDX3 cysteine-dependent interactors**				
P40616	ARL1	ADP-ribosylation factor-like protein 1	4.8	<0.001
Q5SZR4	TDRKH	Tudor and KH domain containing, isoform CRA_a	4.6	<0.001
P11766	ADH5	Alcohol dehydrogenase class-3	4.6	<0.001
F8VYE8	PPP1CC	Serine/threonine-protein phosphatase	4.4	<0.001
B3KUS5	USP30	Ubiquitin carboxyl-terminal hydrolase	4.1	<0.001
Q9NWV4	CZIB	CXXC motif containing zinc binding protein	4.1	<0.001
Q7L2H7	EIF3M	Eukaryotic translation initiation factor 3 subunit M	4.0	<0.001
Q07820	MCL1	Induced myeloid leukemia cell differentiation protein Mcl-1	3.9	<0.001
A0A0B4J1Z1	SRSF7	Serine/arginine-rich-splicing factor 7	3.9	<0.001
P05386	RPLP1	60S acidic ribosomal protein P1	3.9	<0.001
Q07021	C1QBP	Complement component 1 Q subcomponent-binding protein, mitochondrial	3.9	<0.001
H3BTA2	PPP4C	Serine/threonine-protein phosphatase	3.9	<0.001
P07355	ANXA2	Annexin A2	3.9	<0.001
Q14964	RAB39A	Ras-related protein Rab-39A	3.9	0.036
Q7Z434	MAVS	Mitochondrial antiviral-signaling protein	3.8	<0.001
Q9BS26	ERP44	Endoplasmic reticulum resident protein 44	3.7	0.001
Q8N5K1	CISD2	CDGSH iron-sulfur domain-containing protein 2	3.6	0.001
P22061	PCMT1	Protein-L-isoaspartate(D-aspartate) O-methyltransferase	3.6	0.001
C9JAW5	C9JAW5	HIG1 domain-containing protein	3.6	0.001
Q9BYN0	SRXN1	Sulfiredoxin-1	3.5	0.002
G5EA06	MRPS27	28S ribosomal protein S27, mitochondrial	3.5	0.009
Q6IBS0	TWF2	Twinfilin-2	3.4	<0.001
Q9HD26	GOPC	Golgi-associated PDZ and coiled-coil motif-containing protein	3.4	0.001
P06132	UROD	Uroporphyrinogen decarboxylase	3.4	0.001
P00492	HPRT1	Hypoxanthine-guanine phosphoribosyltransferase	3.4	0.001
O43809	NUDT21	Cleavage and polyadenylation specificity factor subunit 5	3.3	<0.001
P61106	RAB14	Ras-related protein Rab-14	3.3	<0.001
Q6DKI1	RPL7L1	60S ribosomal protein L7-like 1	3.3	0.001
P14174	MIF	Macrophage migration inhibitory factor	3.3	0.001
P19022	CDH2	Cadherin-2	3.3	0.001
Q9BQA1	WDR77	Methylosome protein 50	3.2	<0.001
Q92667	AKAP1	A-kinase anchor protein 1, mitochondrial	3.2	<0.001
P51809	VAMP7	Vesicle-associated membrane protein 7	3.2	0.006
Q9NQG6	MIEF1	Mitochondrial dynamics protein MID51	3.2	0.046
P47755	CAPZA2	F-actin-capping protein subunit alpha-2	3.1	<0.001
D6RA00	ENOPH1	Enolase-phosphatase E1	3.1	<0.001
P51148	RAB5C	Ras-related protein Rab-5C	3.1	0.008
Q16698	DECR1	2,4-dienoyl-CoA reductase, mitochondrial	3.0	0.001
P30041	PRDX6	Peroxiredoxin-6	3.0	0.002
O75600	GCAT	2-amino-3-ketobutyrate coenzyme A ligase, mitochondrial	3.0	0.002
Q9NS69	TOMM22	Mitochondrial import receptor subunit TOM22 homolog	3.0	0.002
Q96EA4	SPDL1	Protein Spindly	3.0	0.005
Q92990	GLMN	Glomulin	2.9	<0.001
Q9Y3D0	CIAO2B	Cytosolic iron-sulfur assembly component 2B	2.9	0.001
Q9BTE1	DCTN5	Dynactin subunit 5	2.9	0.002
Q8WVC2	RPS21	40S ribosomal protein S21	2.9	0.002
P02790	HPX	Hemopexin	2.9	0.007
Q15814	TBCC	Tubulin-specific chaperone C	2.9	0.030
Q9BSJ8	ESYT1	Extended synaptotagmin-1	2.8	<0.001
Q9Y5Y2	NUBP2	Cytosolic Fe-S cluster assembly factor NUBP2	2.8	0.001
Q5SY16	NOL9	Polynucleotide 5'-hydroxyl-kinase NOL9	2.8	0.001
Q15084	PDIA6	Protein disulfide-isomerase A6	2.8	0.001
Q9H9P8	L2HGDH	L-2-hydroxyglutarate dehydrogenase, mitochondrial	2.8	0.002
K7EJQ8	HDHD2	Haloacid dehalogenase-like hydrolase domain-containing protein 2	2.8	0.002
Q5T749	KPRP	Keratinocyte proline-rich protein	2.8	0.006
Q8N6T3	ARFGAP1	ADP-ribosylation factor GTPase-activating protein 1	2.7	0.001
M0R026	ILVBL	2-hydroxyacyl-CoA lyase 2	2.7	0.001
Q8IYS1	PM20D2	Peptidase M20 domain-containing protein 2	2.7	0.002
Q9NP61	ARFGAP3	ADP-ribosylation factor GTPase-activating protein 3	2.7	0.003
B4DJ81	NDUFS1	NADH-ubiquinone oxidoreductase 75 kDa subunit, mitochondrial	2.6	0.001
Q96S44	TP53RK	EKC/KEOPS complex subunit TP53RK	2.6	0.002
Q96IU4	ABHD14B	Protein ABHD14B	2.6	0.005
A0A087WU06	TUBGCP3	Gamma-tubulin complex component	2.6	0.007
Q96KB5	PBK	Lymphokine-activated killer T-cell-originated protein kinase	2.6	0.008
Q5VW32	BROX	BRO1 domain-containing protein BROX	2.6	0.011
P18669	PGAM1	Phosphoglycerate mutase 1	2.6	0.015
Q15388	TOMM20	Mitochondrial import receptor subunit TOM20 homolog	2.6	0.020
Q9NZ45	CISD1	CDGSH iron-sulfur domain-containing protein 1	2.6	0.025
O60566	BUB1B	Mitotic checkpoint serine/threonine-protein kinase BUB1 beta	2.6	0.035
Q9P287	BCCIP	BRCA2 and CDKN1A-interacting protein	2.5	0.001
Q96A49	SYAP1	Synapse-associated protein 1	2.5	0.002
P78346	RPP30	Ribonuclease P protein subunit p30	2.5	0.002
E9PJN0	ACOT8	Acyl-coenzyme A thioesterase 8	2.5	0.003
P54646	PRKAA2	5'-AMP-activated protein kinase catalytic subunit alpha-2	2.5	0.005
P30740	SERPINB1	Leukocyte elastase inhibitor	2.5	0.006
Q8N6M0	OTUD6B	Deubiquitinase OTUD6B	2.5	0.007
O95295	SNAPIN	SNARE-associated protein Snapin	2.5	0.007
Q8N6R0	EEF1AKNMT	eEF1A lysine and N-terminal methyltransferase	2.5	0.008
F5H008	VPS33B	Vacuolar protein sorting-associated protein 33B	2.5	0.009
Q9H840	GEMIN7	Gem-associated protein 7	2.5	0.009
Q16186	ADRM1	Proteasomal ubiquitin receptor ADRM1	2.5	0.021
Q5VUJ6	LRCH2	Leucine-rich repeat and calponin homology domain-containing protein 2	2.5	0.028
A6NDU8	C5orf51	UPF0600 protein C5orf51	2.5	0.034
Q7L5D6	GET4	Golgi to ER traffic protein 4 homolog	2.5	0.036
X6RM00	ERC1	ELKS/Rab6-interacting/CAST family member 1	2.4	0.003
Q9UKK9	NUDT5	ADP-sugar pyrophosphatase	2.4	0.004
Q9NYK5	MRPL39	39S ribosomal protein L39, mitochondrial	2.4	0.006
P56385	ATP5ME	ATP synthase subunit e, mitochondrial	2.4	0.008
Q86WA6	BPHL	Valacyclovir hydrolase	2.4	0.011
Q66PJ3	ARL6IP4	ADP-ribosylation factor-like protein 6-interacting protein 4	2.4	0.011
P63151	PPP2R2A	Serine/threonine-protein phosphatase 2A 55 kDa regulatory subunit B alpha isoform	2.4	0.016
P21796	VDAC1	Voltage-dependent anion-selective channel protein 1	2.4	0.026
Q9H6R0	DHX33	ATP-dependent RNA helicase DHX33	2.4	0.032
P11802	CDK4	Cyclin-dependent kinase 4	2.3	<0.001
Q9BQC3	DPH2	2-(3-amino-3-carboxypropyl)histidine synthase subunit 2	2.3	0.003
H3BV80	RNPS1	RNA-binding protein with serine-rich domain 1	2.3	0.005
Q8IVS2	MCAT	Malonyl-CoA-acyl carrier protein transacylase, mitochondrial	2.3	0.006
Q8IUF8	RIOX2	Ribosomal oxygenase 2	2.3	0.007
Q9ULX3	NOB1	RNA-binding protein NOB1	2.3	0.010
P57076	CFAP298	Cilia- and flagella-associated protein 298	2.3	0.011
Q9HCN8	SDF2L1	Stromal cell-derived factor 2-like protein 1	2.2	0.005
O14744	PRMT5	Protein arginine N-methyltransferase 5	2.2	0.006
O60547	GMDS	GDP-mannose 4,6 dehydratase	2.2	0.008
Q9NP97	DYNLRB1	Dynein light chain roadblock-type 1	2.2	0.011
Q9BPZ2	SPIN2B	Spindlin-2B	2.2	0.026
Q6UW78	UQCC3	Ubiquinol-cytochrome-c reductase complex assembly factor 3	2.2	0.045
P62879	GNB2	Guanine nucleotide-binding protein G(I)/G(S)/G(T) subunit beta-2	2.1	0.001
O43237	DYNC1LI2	Cytoplasmic dynein 1 light intermediate chain 2	2.1	0.007
A0A0A0MTB8	WDR36	WD repeat-containing protein 36	2.1	0.009
Q13509	TUBB3	Tubulin beta-3 chain	2.1	0.012
Q92785	DPF2	Zinc finger protein ubi-d4	2.1	0.014
Q16204	CCDC6	Coiled-coil domain-containing protein 6	2.1	0.037
Q9Y6Y8	SEC23IP	SEC23-interacting protein	2.0	0.001
Q14318	FKBP8	Peptidyl-prolyl cis-trans isomerase FKBP8	2.0	0.001
O95801	TTC4	Tetratricopeptide repeat protein 4	2.0	0.008
Q13257	MAD2L1	Mitotic spindle assembly checkpoint protein MAD2A	2.0	0.009
Q8NDI1	EHBP1	EH domain-binding protein 1	2.0	0.010
Q96BW9	TAMM41	Phosphatidate cytidylyltransferase, mitochondrial	2.0	0.010
Q9BZX2	UCK2	Uridine-cytidine kinase 2	2.0	0.011
Q9UQR0	SCML2	Sex comb on midleg-like protein 2	2.0	0.011
O43815	STRN	Striatin	2.0	0.015
Q9H974	QTRT2	Queuine tRNA-ribosyltransferase accessory subunit 2	2.0	0.016
Q15019	SEPTIN2	Septin-2	2.0	0.024
P49903	SEPHS1	Selenide, water dikinase 1	2.0	0.045
P35520	CBS	Cystathionine beta-synthase	1.9	0.003
Q9UI30	TRMT112	Multifunctional methyltransferase subunit TRM112-like protein	1.9	0.004
P52788	SMS	Spermine synthase	1.9	0.012
Q16718	NDUFA5	NADH dehydrogenase [ubiquinone] 1 alpha subcomplex subunit 5	1.9	0.013
Q15291	RBBP5	Retinoblastoma-binding protein 5	1.9	0.014
Q9Y679	AUP1	Lipid droplet-regulating VLDL assembly factor AUP1	1.9	0.014
Q8NBU5	ATAD1	ATPase family AAA domain-containing protein 1	1.9	0.016
P45954	ACADSB	Short/branched chain specific acyl-CoA dehydrogenase, mitochondrial	1.9	0.016
Q96AT9	RPE	Ribulose-phosphate 3-epimerase	1.9	0.018
Q9HB71	CACYBP	Calcyclin-binding protein	1.9	0.019
Q9UHD2	TBK1	Serine/threonine-protein kinase TBK1	1.9	0.022
O95551	TDP2	Tyrosyl-DNA phosphodiesterase 2	1.9	0.025
O75146	HIP1R	Huntingtin-interacting protein 1-related protein	1.9	0.036
Q96S59	RANBP9	Ran-binding protein 9	1.9	0.037
P62979	RPS27A	Ubiquitin-40S ribosomal protein S27a	1.8	0.002
P10599	TXN	Thioredoxin	1.8	0.011
Q9NQX3	GPHN	Gephyrin [Includes: Molybdopterin adenylyltransferase	1.8	0.025
P34896	SHMT1	Serine hydroxymethyltransferase, cytosolic	1.8	0.038
P17252	PRKCA	Protein kinase C alpha type	1.8	0.042
P18031	PTPN1	Tyrosine-protein phosphatase non-receptor type 1	1.7	0.007
Q9Y4B6	DCAF1	DDB1- and CUL4-associated factor 1	1.7	0.045
Q5QPM7	PSMF1	Proteasome inhibitor PI31 subunit	1.6	<0.001
Q8TDH9	BLOC1S5	Biogenesis of lysosome-related organelles complex 1 subunit 5	1.6	0.002
P21964	COMT	Catechol O-methyltransferase	1.6	0.003
Q9UH62	ARMCX3	Armadillo repeat-containing X-linked protein 3	1.6	0.006
Q2M2I8	AAK1	AP2-associated protein kinase 1	1.6	0.036
H7C155	RAF1	Non-specific serine/threonine protein kinase	1.6	0.047
O95831	AIFM1	Apoptosis-inducing factor 1, mitochondrial	1.5	0.002
Q96TC7	RMDN3	Regulator of microtubule dynamics protein 3	1.5	0.002
B4E1N1	ARMC6	Armadillo repeat-containing protein 6	1.5	0.007
Q9UL25	RAB21	Ras-related protein Rab-21	1.5	0.012
A0A0A0MR02	VDAC2	Outer mitochondrial membrane protein porin 2	1.5	0.014
Q16543	CDC37	Hsp90 co-chaperone Cdc37	1.5	0.016
O15269	SPTLC1	Serine palmitoyltransferase 1	1.5	0.018
P35268	RPL22	60S ribosomal protein L22	1.5	0.035
O75688	PPM1B	Protein phosphatase 1B	1.5	0.038
Q9Y266	NUDC	Nuclear migration protein nudC	1.5	0.047
O60825	PFKFB2	6-phosphofructo-2-kinase/fructose-2,6-bisphosphatase 2	1.5	0.049
Q16576	RBBP7	Histone-binding protein RBBP7	1.4	0.002
P60953	CDC42	Cell division control protein 42 homolog	1.4	0.002
Q8TC07	TBC1D15	TBC1 domain family member 15	1.4	0.005
Q9UGV2	NDRG3	Protein NDRG3	1.4	0.010
P62851	RPS25	40S ribosomal protein S25	1.4	0.010
Q5T760	SRSF11	Serine/arginine-rich-splicing factor 11	1.4	0.010
O75663	TIPRL	TIP41-like protein	1.4	0.034
Q9UL15	BAG5	BAG family molecular chaperone regulator 5	1.4	0.038
Q147X3	NAA30	N-alpha-acetyltransferase 30	1.4	0.047
Q8IXI1	RHOT2	Mitochondrial Rho GTPase 2	1.3	0.001
Q9GZS3	WDR61	WD repeat-containing protein 61	1.3	0.003
P51553	IDH3G	Isocitrate dehydrogenase [NAD] subunit gamma, mitochondrial	1.3	0.014
Q8TEX9	IPO4	Importin-4	1.3	0.024
Q5VT66	MTARC1	Mitochondrial amidoxime-reducing component 1	1.3	0.028
P19367	HK1	Hexokinase-1	1.3	0.035
P62820	RAB1A	Ras-related protein Rab-1A	1.3	0.040
Q9Y570	PPME1	Protein phosphatase methylesterase 1	1.3	0.047
P35237	SERPINB6	Serpin B6	1.2	0.002
O60784	TOM1	Target of Myb protein 1	1.2	0.006
P55072	VCP	Transitional endoplasmic reticulum ATPase	1.2	0.018
P20042	EIF2S2	Eukaryotic translation initiation factor 2 subunit 2	1.2	0.023
Q9NQT5	EXOSC3	Exosome complex component RRP40	1.2	0.026
P29558	RBMS1	RNA-binding motif, single-stranded-interacting protein 1	1.2	0.027
P26368	U2AF2	Splicing factor U2AF 65 kDa subunit	1.2	0.038
Q9H0U4	RAB1B	Ras-related protein Rab-1B	1.2	0.045
P62873	GNB1	Guanine nucleotide-binding protein G(I)/G(S)/G(T) subunit beta-1	1.1	0.009
Q15738	NSDHL	Sterol-4-alpha-carboxylate 3-dehydrogenase, decarboxylating	1.1	0.024
P31040	SDHA	Succinate dehydrogenase [ubiquinone] flavoprotein subunit, mitochondrial	1.1	0.027
P09211	GSTP1	Glutathione S-transferase P	1.1	0.027
Q53EL6	PDCD4	Programmed cell death protein 4	1.1	0.047
Q14126	DSG2	Desmoglein-2	1.0	0.012
Q96FW1	OTUB1	Ubiquitin thioesterase OTUB1	1.0	0.019
P56182	RRP1	Ribosomal RNA processing protein 1 homolog A	1.0	0.026
P50454	SERPINH1	Serpin H1	1.0	0.029
Q14498	RBM39	RNA-binding protein 39	1.0	0.035
P62081	RPS7	40S ribosomal protein S7	1.0	0.037
P16435	POR	NADPH--cytochrome P450 reductase	1.0	0.041
**PRDX4 cysteine-dependent interactors**				
Q12797	ASPH	Aspartyl/asparaginyl beta-hydroxylase	3.9	<0.001
I3L1P8	SLC25A11	Mitochondrial 2-oxoglutarate/malate carrier protein	3.5	<0.001
A0A0B4J1Z1	SRSF7	Serine/arginine-rich-splicing factor 7	3.1	0.002
F5H6E2	MYO1C	Unconventional myosin-Ic	2.9	0.001
O00469	PLOD2	Procollagen-lysine,2-oxoglutarate 5-dioxygenase 2	2.8	0.030
Q9NVP1	DDX18	ATP-dependent RNA helicase DDX18	2.6	0.007
Q9BSJ8	ESYT1	Extended synaptotagmin-1	2.5	0.003
Q9Y2R4	DDX52	Probable ATP-dependent RNA helicase DDX52	2.3	0.024
Q13618	CUL3	Cullin-3	2.2	0.013
Q9Y2R5	MRPS17	28S ribosomal protein S17, mitochondrial	2.2	0.030
D6RA00	ENOPH1	Enolase-phosphatase E1	2.1	0.017
P30154	PPP2R1B	Serine/threonine-protein phosphatase 2A 65 kDa regulatory subunit A beta isoform	2.1	0.021
O75592	MYCBP2	E3 ubiquitin-protein ligase MYCBP2	2.1	0.027
Q8NBS9	TXNDC5	Thioredoxin domain-containing protein 5	1.9	0.007
P84103	SRSF3	Serine/arginine-rich splicing factor 3	1.9	0.028
Q12905	ILF2	Interleukin enhancer-binding factor 2	1.9	0.032
P29372	MPG	DNA-3-methyladenine glycosylase	1.9	0.039
Q7KZ85	SUPT6H	Transcription elongation factor SPT6	1.9	0.043
Q92783	STAM	Signal transducing adapter molecule 1	1.8	0.041
Q66K74	MAP1S	Microtubule-associated protein 1S	1.8	0.049
A0A2R8Y7S2	SMARCA4	Transcription activator BRG1	1.7	0.008
B4DJ81	NDUFS1	NADH-ubiquinone oxidoreductase 75 kDa subunit, mitochondrial	1.7	0.039
Q8IUI8	CRLF3	Cytokine receptor-like factor 3	1.7	0.049
Q8WX93	PALLD	Palladin	1.7	0.049
Q14697	GANAB	Neutral alpha-glucosidase AB	1.6	0.001
P11802	CDK4	Cyclin-dependent kinase 4	1.6	0.011
A0A087X054	HYOU1	Hypoxia up-regulated protein 1	1.6	0.019
P49593	PPM1F	Protein phosphatase 1F	1.5	0.015
O75607	NPM3	Nucleoplasmin-3	1.5	0.022
Q14152	EIF3A	Eukaryotic translation initiation factor 3 subunit A	1.5	0.025
O14974	PPP1R12A	Protein phosphatase 1 regulatory subunit 12A	1.5	0.032
Q9BVP2	GNL3	Guanine nucleotide-binding protein-like 3	1.5	0.034
Q9C0C2	TNKS1BP1	182 kDa tankyrase-1-binding protein	1.5	0.043
Q9HCN8	SDF2L1	Stromal cell-derived factor 2-like protein 1	1.4	0.002
Q16513	PKN2	Serine/threonine-protein kinase N2	1.4	0.017
O43809	NUDT21	Cleavage and polyadenylation specificity factor subunit 5	1.4	0.028
Q92974	ARHGEF2	Rho guanine nucleotide exchange factor 2	1.3	0.002
Q5SW79	CEP170	Centrosomal protein of 170 kDa	1.3	0.013
Q9UQR0	SCML2	Sex comb on midleg-like protein 2	1.3	0.016
Q14318	FKBP8	Peptidyl-prolyl cis-trans isomerase FKBP8	1.3	0.020
P27824	CANX	Calnexin	1.3	0.022
P49411	TUFM	Elongation factor Tu, mitochondrial	1.3	0.022
Q9BWF3	RBM4	RNA-binding protein 4	1.3	0.038
Q16543	CDC37	Hsp90 co-chaperone Cdc37	1.3	0.039
A0A087WY71	AP2M1	AP-2 complex subunit mu	1.2	0.014
P18858	LIG1	DNA ligase 1	1.2	0.014
Q12904	AIMP1	Aminoacyl tRNA synthase complex-interacting multifunctional protein 1	1.2	0.019
P22234	PAICS	Multifunctional protein ADE2 [Includes: Phosphoribosylaminoimidazole-succinocarboxamide synthase	1.2	0.033
Q8TCG1	CIP2A	Protein CIP2A	1.2	0.040
P62249	RPS16	40S ribosomal protein S16	1.2	0.043
Q09666	AHNAK	Neuroblast differentiation-associated protein AHNAK	1.1	0.006
E7EPN9	PRRC2C	Protein PRRC2C	1.1	0.018
Q99567	NUP88	Nuclear pore complex protein Nup88	1.1	0.032
P30520	ADSS2	Adenylosuccinate synthetase isozyme 2	1.1	0.035
Q07020	RPL18	60S ribosomal protein L18	1.1	0.036
P13489	RNH1	Ribonuclease inhibitor	1.0	0.030
**PRDX5 cysteine-dependent interactors**				
P36551	CPOX	Oxygen-dependent coproporphyrinogen-III oxidase, mitochondrial	6.4	<0.001
Q6DKK2	TTC19	Tetratricopeptide repeat protein 19, mitochondrial	3.9	<0.001
Q96A49	SYAP1	Synapse-associated protein 1	3.9	<0.001
O00165	HAX1	HCLS1-associated protein X-1	3.7	<0.001
A0A0C4DGQ6	RPRD1A	Regulation of nuclear pre-mRNA domain-containing protein 1A	2.9	0.002
P78371	CCT2	T-complex protein 1 subunit beta	2.7	<0.001
P85037	FOXK1	Forkhead box protein K1	2.6	0.012
A0A024R442	A0A024R442	>tr|A0A024R442|A0A024R442_HUMAN Aspartyl aminopeptidase, isoform CRA_b OS=Homo sapiens OX=9606 GN=DNPEP PE=1 SV=1;>sp|Q9ULA0|DNPEP_HUMAN Aspartyl aminopeptidase OS=Homo sapiens OX=9606 GN=DNPEP PE=1 SV=1;>tr|E7ETB3|E7ETB3_HUMAN Aspartyl aminopeptidase, iso	2.6	0.012
P30740	SERPINB1	Leukocyte elastase inhibitor	2.3	0.016
Q2TAM5	RELA	RELA protein	2.2	0.013
Q10713	PMPCA	Mitochondrial-processing peptidase subunit alpha	2.2	0.018
A0A075B6R9	IGKV2D-24	Probable non-functional immunoglobulin kappa variable 2D-24	2.2	0.041
Q8NFC6	BOD1L1	Biorientation of chromosomes in cell division protein 1-like 1	2.1	<0.001
O95801	TTC4	Tetratricopeptide repeat protein 4	2.1	0.025
Q6PID6	TTC33	Tetratricopeptide repeat protein 33	2.1	0.043
Q96HS1	PGAM5	Serine/threonine-protein phosphatase PGAM5, mitochondrial	2.0	0.005
P13667	PDIA4	Protein disulfide-isomerase A4	2.0	0.018
O00483	NDUFA4	Cytochrome c oxidase subunit NDUFA4	2.0	0.025
Q96DH6	MSI2	RNA-binding protein Musashi homolog 2	2.0	0.044
P62979	RPS27A	Ubiquitin-40S ribosomal protein S27a	1.9	0.001
P07355	ANXA2	Annexin A2	1.9	0.007
E9PGZ1	CALD1	Caldesmon	1.8	0.023
O95831	AIFM1	Apoptosis-inducing factor 1, mitochondrial	1.6	0.001
Q9NZL9	MAT2B	Methionine adenosyltransferase 2 subunit beta	1.6	0.006
H7C128	BRD8	Bromodomain-containing protein 8	1.6	0.011
P48047	ATP5PO	ATP synthase subunit O, mitochondrial	1.6	0.013
P43304	GPD2	Glycerol-3-phosphate dehydrogenase, mitochondrial	1.6	0.015
P43686	PSMC4	26S proteasome regulatory subunit 6B	1.6	0.016
E5KLJ9	OPA1	Dynamin-like 120 kDa protein, form S1	1.5	0.004
P26599	PTBP1	Polypyrimidine tract-binding protein 1	1.5	0.006
Q13257	MAD2L1	Mitotic spindle assembly checkpoint protein MAD2A	1.5	0.020
Q96IZ0	PAWR	PRKC apoptosis WT1 regulator protein	1.4	0.013
A0A0A0MR02	VDAC2	Outer mitochondrial membrane protein porin 2	1.4	0.016
Q8N3X1	FNBP4	Formin-binding protein 4	1.3	0.009
C9J4Z3	RPL37A	60S ribosomal protein L37a	1.3	0.022
P31689	DNAJA1	DnaJ homolog subfamily A member 1	1.2	0.003
Q9HB71	CACYBP	Calcyclin-binding protein	1.2	0.018
Q8NC51	SERBP1	Plasminogen activator inhibitor 1 RNA-binding protein	1.2	0.028
P22234	PAICS	Multifunctional protein ADE2 [Includes: Phosphoribosylaminoimidazole-succinocarboxamide synthase	1.2	0.035
Q9NX63	CHCHD3	MICOS complex subunit MIC19	1.1	0.004
Q5QPM7	PSMF1	Proteasome inhibitor PI31 subunit	1.1	0.007
Q14C86	GAPVD1	GTPase-activating protein and VPS9 domain-containing protein 1	1.1	0.020
P40222	TXLNA	Alpha-taxilin	1.1	0.048

Table 4. Cysteine-dependent interactors (wild-type PRDX and PRDX-C$_R$S/PRDX-C$_{PR}$S)

uniprot ID	gene	protein	interaction
**PRDX1 cysteine-dependent interactors**			
P62273	RPS29	40S ribosomal protein S29	WT
P13667	PDIA4	Protein disulfide-isomerase A4	WT
O60762	DPM1	Dolichol-phosphate mannosyltransferase subunit 1	WT
Q15185	PTGES3	Prostaglandin E synthase 3	WT, CrS
P31323	PRKAR2B	cAMP-dependent protein kinase type II-beta regulatory subunit	WT
P50570	DNM2	Dynamin-2	WT
Q13257	MAD2L1	Mitotic spindle assembly checkpoint protein MAD2A	WT
A0A3B3IUD2	MSTO1	Protein misato homolog 1	WT
Q96FV9	THOC1	THO complex subunit 1	WT
P15924	DSP	Desmoplakin	WT, CrS
Q9Y2V2	CARHSP1	Calcium-regulated heat-stable protein 1	WT, CrS
Q9H845	ACAD9	Complex I assembly factor ACAD9, mitochondrial	WT
P83731	RPL24	60S ribosomal protein L24	WT
Q9BY32	ITPA	Inosine triphosphate pyrophosphatase	WT, CrS
O94760	DDAH1	N(G),N(G)-dimethylarginine dimethylaminohydrolase 1	WT
P62266	RPS23	40S ribosomal protein S23	WT
Q8WWH5	TRUB1	Probable tRNA pseudouridine synthase 1	WT
Q6P1L8	MRPL14	39S ribosomal protein L14, mitochondrial	WT
Q99961	SH3GL1	Endophilin-A2	WT
P08559	PDHA1	Pyruvate dehydrogenase E1 component subunit alpha, somatic form, mitochondrial	WT, CrS
Q01082	SPTBN1	Spectrin beta chain, non-erythrocytic 1	WT
I3L2Z5	MAZ	Myc-associated zinc finger protein	WT
P49792	RANBP2	E3 SUMO-protein ligase RanBP2	WT, CrS
Q14008	CKAP5	Cytoskeleton-associated protein 5	WT, CrS
Q7L2E3	DHX30	ATP-dependent RNA helicase DHX30	WT
O14964	HGS	Hepatocyte growth factor-regulated tyrosine kinase substrate	WT
Q5JTH9	RRP12	RRP12-like protein	WT
P49841	GSK3B	Glycogen synthase kinase-3 beta	WT
P48735	IDH2	Isocitrate dehydrogenase [NADP], mitochondrial	WT
Q15149	PLEC	Plectin	WT, CrS
E3W994	CLASP2	CLIP-associating protein 2	WT
Q16698	DECR1	2,4-dienoyl-CoA reductase, mitochondrial	WT
P48047	ATP5PO	ATP synthase subunit O, mitochondrial	WT, CrS
E7EVH7	E7EVH7	Kinesin light chain	WT
Q15717	ELAVL1	ELAV-like protein 1	WT
P22626	HNRNPA2B1	Heterogeneous nuclear ribonucleoproteins A2/B1	WT
Q99615	DNAJC7	DnaJ homolog subfamily C member 7	WT, CrS
Q7L1Q6	BZW1	Basic leucine zipper and W2 domain-containing protein 1	WT
Q01813	PFKP	ATP-dependent 6-phosphofructokinase, platelet type	WT
Q8N1F7	NUP93	Nuclear pore complex protein Nup93	WT
O95801	TTC4	Tetratricopeptide repeat protein 4	WT
P63165	SUMO1	Small ubiquitin-related modifier 1	WT, CrS
P30048	PRDX3	Thioredoxin-dependent peroxide reductase, mitochondrial	WT, CrS
D6RBD7	EEF1E1	Eukaryotic translation elongation factor 1 epsilon-1	WT
Q99543	DNAJC2	DnaJ homolog subfamily C member 2	WT
Q9NW13	RBM28	RNA-binding protein 28	WT
Q08211	DHX9	ATP-dependent RNA helicase A	WT
A0A1W2PRV5	SMN2	Survival motor neuron protein	WT
O60493	SNX3	Sorting nexin-3	WT
P62318	SNRPD3	Small nuclear ribonucleoprotein Sm D3	WT
Q9Y5K6	CD2AP	CD2-associated protein	WT
Q02241	KIF23	Kinesin-like protein KIF23	WT
P19623	SRM	Spermidine synthase	WT
P24534	EEF1B2	Elongation factor 1-beta	WT
Q9BV44	THUMPD3	THUMP domain-containing protein 3	WT
Q9BQG0	MYBBP1A	Myb-binding protein 1A	WT, CrS
A0A0C4DGX4	CUL1	Cullin-1	WT
P51153	RAB13	Ras-related protein Rab-13	WT
Q8TCG1	CIP2A	Protein CIP2A	WT
P62851	RPS25	40S ribosomal protein S25	WT, CrS
Q92879	CELF1	CUGBP Elav-like family member 1	WT, CrS
O14617	AP3D1	AP-3 complex subunit delta-1	WT
P53582	METAP1	Methionine aminopeptidase 1	WT
Q71UI9	H2AZ2	Histone H2A.V	WT
Q96I25	RBM17	Splicing factor 45	WT, CrS
O75369	FLNB	Filamin-B	WT
P43686	PSMC4	26S proteasome regulatory subunit 6B	WT
O43447	PPIH	Peptidyl-prolyl cis-trans isomerase H	WT
Q13595	TRA2A	Transformer-2 protein homolog alpha	WT
P36915	GNL1	Guanine nucleotide-binding protein-like 1	WT, CrS
P24666	ACP1	Low molecular weight phosphotyrosine protein phosphatase	WT, CrS
P61081	UBE2M	NEDD8-conjugating enzyme Ubc12	WT
P46940	IQGAP1	Ras GTPase-activating-like protein IQGAP1	WT, CrS
P61011	SRP54	Signal recognition particle 54 kDa protein	WT, CrS
Q13247	SRSF6	Serine/arginine-rich splicing factor 6	WT
Q9HAV4	XPO5	Exportin-5	WT
E9PD53	SMC4	Structural maintenance of chromosomes protein	WT, CrS
O15446	POLR1G	DNA-directed RNA polymerase I subunit RPA34	WT
Q96N67	DOCK7	Dedicator of cytokinesis protein 7	WT, CrS
A0A2R8YDQ9	SUCLA2	Succinate--CoA ligase [ADP-forming] subunit beta, mitochondrial	WT, CrS
P49207	RPL34	60S ribosomal protein L34	WT, CrS
Q9GZT3	SLIRP	SRA stem-loop-interacting RNA-binding protein, mitochondrial	WT, CrS
Q8WTT2	NOC3L	Nucleolar complex protein 3 homolog	WT
P62280	RPS11	40S ribosomal protein S11	WT
P61160	ACTR2	Actin-related protein 2	WT
Q92615	LARP4B	La-related protein 4B	WT
A0A0A0MRM9	NOLC1	Nucleolar and coiled-body phosphoprotein 1	WT
O14974	PPP1R12A	Protein phosphatase 1 regulatory subunit 12A	WT
P49790	NUP153	Nuclear pore complex protein Nup153	WT, CrS
Q9H6T3	RPAP3	RNA polymerase II-associated protein 3	WT
P07196	NEFL	Neurofilament light polypeptide	WT
P61313	RPL15	60S ribosomal protein L15	WT
A0A087WZ13	RAVER1	Ribonucleoprotein PTB-binding 1	WT, CrS
O15305	PMM2	Phosphomannomutase 2	WT
E7EX90	DCTN1	Dynactin subunit 1	WT, CrS
Q9H0C8	ILKAP	Integrin-linked kinase-associated serine/threonine phosphatase 2C	WT, CrS
Q9BUQ8	DDX23	Probable ATP-dependent RNA helicase DDX23	WT
Q9BQ39	DDX50	ATP-dependent RNA helicase DDX50	WT
Q9UQE7	SMC3	Structural maintenance of chromosomes protein 3	WT, CrS
Q9H2G2	SLK	STE20-like serine/threonine-protein kinase	WT, CrS
Q13363	CTBP1	C-terminal-binding protein 1	WT
Q13813	SPTAN1	Spectrin alpha chain, non-erythrocytic 1	WT
A0A1B0GV47	KIF21A	Kinesin-like protein KIF21A	WT
O43707	ACTN4	Alpha-actinin-4	WT
Q8N9T8	KRI1	Protein KRI1 homolog	WT, CrS
Q7Z434	MAVS	Mitochondrial antiviral-signaling protein	WT
P61163	ACTR1A	Alpha-centractin	WT, CrS
A0A1W2PPZ5	TCEA1	Transcription elongation factor A protein 1	WT
F8W8I6	TIA1	Nucleolysin TIA-1 isoform p40	WT
P23258	TUBG1	Tubulin gamma-1 chain	WT, CrS
Q02878	RPL6	60S ribosomal protein L6	WT
P25490	YY1	Transcriptional repressor protein YY1	WT
A0A2R8Y804	CTNNB1	Catenin beta-1	WT
B5MCF9	PES1	Pescadillo homolog	WT, CrS
Q9NSK0	KLC4	Kinesin light chain 4	WT
Q96PK6	RBM14	RNA-binding protein 14	WT
Q9UHI6	DDX20	Probable ATP-dependent RNA helicase DDX20	WT, CrS
H3BPE1	MACF1	Microtubule-actin cross-linking factor 1, isoforms 1/2/3/5	WT, CrS
Q15813	TBCE	Tubulin-specific chaperone E	WT
Q8WVM8	SCFD1	Sec1 family domain-containing protein 1	WT, CrS
Q16352	INA	Alpha-internexin	WT
P19338	NCL	Nucleolin	WT
A0A087X2D5	MRPL45	39S ribosomal protein L45, mitochondrial	WT
A0A0A0MRT6	ABI1	Abl interactor 1	WT, CrS
P48507	GCLM	Glutamate--cysteine ligase regulatory subunit	WT
Q9H9A6	LRRC40	Leucine-rich repeat-containing protein 40	WT
P46060	RANGAP1	Ran GTPase-activating protein 1	WT, CrS
Q9ULV4	CORO1C	Coronin-1C	WT, CrS
F5GWT4	WNK1	Non-specific serine/threonine protein kinase	WT
A0A3B3ISG5	IDE	Insulin-degrading enzyme	WT
P14174	MIF	Macrophage migration inhibitory factor	WT, CrS
Q92974	ARHGEF2	Rho guanine nucleotide exchange factor 2	WT
Q13561	DCTN2	Dynactin subunit 2	WT, CrS
Q9H0B6	KLC2	Kinesin light chain 2	WT, CrS
Q9NR12	PDLIM7	PDZ and LIM domain protein 7	WT, CrS
Q14684	RRP1B	Ribosomal RNA processing protein 1 homolog B	WT, CrS
A5YKK6	CNOT1	CCR4-NOT transcription complex subunit 1	WT
P31942	HNRNPH3	Heterogeneous nuclear ribonucleoprotein H3	WT
P08243	ASNS	Asparagine synthetase [glutamine-hydrolyzing]	WT
Q96MU7	YTHDC1	YTH domain-containing protein 1	WT
P78316	NOP14	Nucleolar protein 14	WT
Q9NZB2	FAM120A	Constitutive coactivator of PPAR-gamma-like protein 1	WT
Q5JRX3	PITRM1	Presequence protease, mitochondrial	WT, CrS
Q96D71	REPS1	RalBP1-associated Eps domain-containing protein 1	WT
Q14683	SMC1A	Structural maintenance of chromosomes protein 1A	WT, CrS
Q9UG63	ABCF2	ATP-binding cassette sub-family F member 2	WT, CrS
Q5TDH0	DDI2	Protein DDI1 homolog 2	WT, CrS
P82921	MRPS21	28S ribosomal protein S21, mitochondrial	WT
O75717	WDHD1	WD repeat and HMG-box DNA-binding protein 1	WT
Q96KB5	PBK	Lymphokine-activated killer T-cell-originated protein kinase	WT
P27144	AK4	Adenylate kinase 4, mitochondrial	WT
E7ESP9	NEFM	160 kDa neurofilament protein	WT, CrS
Q9Y237	PIN4	Peptidyl-prolyl cis-trans isomerase NIMA-interacting 4	WT
F8WB06	ATXN2	Ataxin-2	WT, CrS
P54578	USP14	Ubiquitin carboxyl-terminal hydrolase 14	WT, CrS
P20042	EIF2S2	Eukaryotic translation initiation factor 2 subunit 2	WT, CrS
Q8WWK9	CKAP2	Cytoskeleton-associated protein 2	WT
P52948	NUP98	Nuclear pore complex protein Nup98-Nup96	WT
Q07666	KHDRBS1	KH domain-containing, RNA-binding, signal transduction-associated protein 1	WT, CrS
P46013	MKI67	Proliferation marker protein Ki-67	WT
P55084	HADHB	Trifunctional enzyme subunit beta, mitochondrial	WT, CrS
P18031	PTPN1	Tyrosine-protein phosphatase non-receptor type 1	WT
Q15654	TRIP6	Thyroid receptor-interacting protein 6	WT, CrS
Q9Y2Z4	YARS2	Tyrosine--tRNA ligase, mitochondrial	WT
Q14258	TRIM25	E3 ubiquitin/ISG15 ligase TRIM25	WT
O00425	IGF2BP3	Insulin-like growth factor 2 mRNA-binding protein 3	WT, CrS
P35659	DEK	Protein DEK	WT
O75592	MYCBP2	E3 ubiquitin-protein ligase MYCBP2	WT
O00273	DFFA	DNA fragmentation factor subunit alpha	WT
Q9H2U1	DHX36	ATP-dependent DNA/RNA helicase DHX36	WT
P29558	RBMS1	RNA-binding motif, single-stranded-interacting protein 1	WT
Q9BXP5	SRRT	Serrate RNA effector molecule homolog	WT
Q14974	KPNB1	Importin subunit beta-1	WT
Q96RP9	GFM1	Elongation factor G, mitochondrial	WT, CrS
Q02252	ALDH6A1	Methylmalonate-semialdehyde dehydrogenase [acylating], mitochondrial	WT, CrS
A0A024R4E5	HDLBP	High density lipoprotein binding protein	WT, CrS
Q9GZS1	POLR1E	DNA-directed RNA polymerase I subunit RPA49	WT
P38432	COIL	Coilin	WT
Q9ULT8	HECTD1	E3 ubiquitin-protein ligase HECTD1	WT
Q13838	DDX39B	Spliceosome RNA helicase DDX39B	WT
Q71RC2	LARP4	La-related protein 4	WT, CrS
Q9UGI8	TES	Testin	WT
Q02543	RPL18A	60S ribosomal protein L18a	WT
P78344	EIF4G2	Eukaryotic translation initiation factor 4 gamma 2	WT, CrS
P60174	TPI1	Triosephosphate isomerase	WT
O76003	GLRX3	Glutaredoxin-3	WT
O43252	PAPSS1	Bifunctional 3'-phosphoadenosine 5'-phosphosulfate synthase 1	WT
P55010	EIF5	Eukaryotic translation initiation factor 5	WT
Q9UQN3	CHMP2B	Charged multivesicular body protein 2b	WT
Q14677	CLINT1	Clathrin interactor 1	WT
Q13283	G3BP1	Ras GTPase-activating protein-binding protein 1	WT
P35249	RFC4	Replication factor C subunit 4	WT
Q9Y5X1	SNX9	Sorting nexin-9	WT
Q9NYJ8	TAB2	TGF-beta-activated kinase 1 and MAP3K7-binding protein 2	WT
P19367	HK1	Hexokinase-1	WT, CrS
Q9UNE7	STUB1	E3 ubiquitin-protein ligase CHIP	WT, CrS
E9PB90	HK2	Hexokinase	WT
Q7Z6Z7	HUWE1	E3 ubiquitin-protein ligase HUWE1	WT, CrS
F6T1Q0	PDE12	2',5'-phosphodiesterase 12	WT
Q5JTZ9	AARS2	Alanine--tRNA ligase, mitochondrial	WT
O14497	ARID1A	AT-rich interactive domain-containing protein 1A	WT
Q86U42	PABPN1	Polyadenylate-binding protein 2	WT
P37802	TAGLN2	Transgelin-2	WT, CrS
Q9BVJ6	UTP14A	U3 small nucleolar RNA-associated protein 14 homolog A	WT
Q8N163	CCAR2	Cell cycle and apoptosis regulator protein 2	WT
Q86VS8	HOOK3	Protein Hook homolog 3	WT
P39023	RPL3	60S ribosomal protein L3	WT, CrS
Q14161	GIT2	ARF GTPase-activating protein GIT2	WT
Q86UK7	ZNF598	E3 ubiquitin-protein ligase ZNF598	WT
P04637	TP53	Cellular tumor antigen p53	WT, CrS
O95453	PARN	Poly(A)-specific ribonuclease PARN	WT
Q86YP4	GATAD2A	Transcriptional repressor p66-alpha	WT
P22307	SCP2	Non-specific lipid-transfer protein	WT, CrS
Q15042	RAB3GAP1	Rab3 GTPase-activating protein catalytic subunit	WT, CrS
P23246	SFPQ	Splicing factor, proline- and glutamine-rich	WT
P00519	ABL1	Tyrosine-protein kinase ABL1	WT
Q9UI30	TRMT112	Multifunctional methyltransferase subunit TRM112-like protein	WT
P26373	RPL13	60S ribosomal protein L13	WT
A0A087WVZ9	POLR2E	DNA-directed RNA polymerase II subunit E	WT
M0R0F0	RPS5	40S ribosomal protein S5	WT, CrS
Q86WJ1	CHD1L	Chromodomain-helicase-DNA-binding protein 1-like	WT
Q14151	SAFB2	Scaffold attachment factor B2	WT, CrS
P28838	LAP3	Cytosol aminopeptidase	WT, CrS
P46777	RPL5	60S ribosomal protein L5	WT
O95782	AP2A1	AP-2 complex subunit alpha-1	WT, CrS
Q9H3U1	UNC45A	Protein unc-45 homolog A	WT
Q9H0H5	RACGAP1	Rac GTPase-activating protein 1	WT
Q01081	U2AF1	Splicing factor U2AF 35 kDa subunit	WT, CrS
O95239	KIF4A	Chromosome-associated kinesin KIF4A	WT
P12277	CKB	Creatine kinase B-type	WT
A0A087WY71	AP2M1	AP-2 complex subunit mu	WT
P55809	OXCT1	Succinyl-CoA:3-ketoacid coenzyme A transferase 1, mitochondrial	WT, CrS
C9JP00	MBNL1	Muscleblind-like protein 1	WT
P52732	KIF11	Kinesin-like protein KIF11	WT
Q14204	DYNC1H1	Cytoplasmic dynein 1 heavy chain 1	WT, CrS
Q13509	TUBB3	Tubulin beta-3 chain	WT, CrS
P46063	RECQL	ATP-dependent DNA helicase Q1	WT
P51114	FXR1	Fragile X mental retardation syndrome-related protein 1	WT, CrS
Q5HYI8	RABL3	Rab-like protein 3	WT
P42166	TMPO	Lamina-associated polypeptide 2, isoform alpha	WT
Q8TD19	NEK9	Serine/threonine-protein kinase Nek9	WT
Q7Z4H3	HDDC2	5'-deoxynucleotidase HDDC2	WT
Q9HD26	GOPC	Golgi-associated PDZ and coiled-coil motif-containing protein	WT, CrS
Q53H96	PYCR3	Pyrroline-5-carboxylate reductase 3	WT
Q9NPI6	DCP1A	mRNA-decapping enzyme 1A	WT
Q9BYN8	MRPS26	28S ribosomal protein S26, mitochondrial	WT
Q9NYZ3	GTSE1	G2 and S phase-expressed protein 1	WT
O94927	HAUS5	HAUS augmin-like complex subunit 5	WT
P57740	NUP107	Nuclear pore complex protein Nup107	WT, CrS
P54920	NAPA	Alpha-soluble NSF attachment protein	WT
P17252	PRKCA	Protein kinase C alpha type	WT
F8W8R3	POLD2	DNA polymerase delta subunit 2	WT
Q9H814	PHAX	Phosphorylated adapter RNA export protein	WT
P15170	GSPT1	Eukaryotic peptide chain release factor GTP-binding subunit ERF3A	WT, CrS
O43837	IDH3B	Isocitrate dehydrogenase [NAD] subunit beta, mitochondrial	WT, CrS
Q14966	ZNF638	Zinc finger protein 638	WT
Q7RTV0	PHF5A	PHD finger-like domain-containing protein 5A	WT, CrS
P53597	SUCLG1	Succinate--CoA ligase [ADP/GDP-forming] subunit alpha, mitochondrial	WT
Q9NR30	DDX21	Nucleolar RNA helicase 2	WT
E9PQV9	DCUN1D5	DCN1-like protein	WT
O00231	PSMD11	26S proteasome non-ATPase regulatory subunit 11	WT
P25786	PSMA1	Proteasome subunit alpha type-1	WT
P51570	GALK1	Galactokinase	WT
Q8NB90	SPATA5	ATPase family protein 2 homolog	WT
A0A3B3IRP5	CDC73	Parafibromin	WT, CrS
Q9H2P0	ADNP	Activity-dependent neuroprotector homeobox protein	WT
Q13464	ROCK1	Rho-associated protein kinase 1	WT
Q52LJ0	FAM98B	Protein FAM98B	WT, CrS
Q15233	NONO	Non-POU domain-containing octamer-binding protein	WT
Q9P2J5	LARS1	Leucine--tRNA ligase, cytoplasmic	WT, CrS
P60228	EIF3E	Eukaryotic translation initiation factor 3 subunit E	WT
C9JZR2	CTNND1	Catenin delta-1	WT, CrS
P49959	MRE11	Double-strand break repair protein MRE11	WT
P33240	CSTF2	Cleavage stimulation factor subunit 2	WT
Q16513	PKN2	Serine/threonine-protein kinase N2	WT, CrS
P15880	RPS2	40S ribosomal protein S2	WT, CrS
O43264	ZW10	Centromere/kinetochore protein zw10 homolog	WT
Q08752	PPID	Peptidyl-prolyl cis-trans isomerase D	WT
P35221	CTNNA1	Catenin alpha-1	WT
Q14669	TRIP12	E3 ubiquitin-protein ligase TRIP12	WT
P46778	RPL21	60S ribosomal protein L21	WT, CrS
P50213	IDH3A	Isocitrate dehydrogenase [NAD] subunit alpha, mitochondrial	WT
Q8N1G4	LRRC47	Leucine-rich repeat-containing protein 47	WT, CrS
O43237	DYNC1LI2	Cytoplasmic dynein 1 light intermediate chain 2	WT
Q14152	EIF3A	Eukaryotic translation initiation factor 3 subunit A	WT, CrS
Q6P2E9	EDC4	Enhancer of mRNA-decapping protein 4	WT, CrS
P23919	DTYMK	Thymidylate kinase	WT, CrS
P63272	SUPT4H1	Transcription elongation factor SPT4	WT, CrS
P52597	HNRNPF	Heterogeneous nuclear ribonucleoprotein F	WT
Q15785	TOMM34	Mitochondrial import receptor subunit TOM34	WT
P13804	ETFA	Electron transfer flavoprotein subunit alpha, mitochondrial	WT, CrS
Q10567	AP1B1	AP-1 complex subunit beta-1	WT, CrS
P47897	QARS1	Glutamine--tRNA ligase	WT, CrS
P62249	RPS16	40S ribosomal protein S16	WT
Q92616	GCN1	eIF-2-alpha kinase activator GCN1	WT
P19525	EIF2AK2	Interferon-induced, double-stranded RNA-activated protein kinase	WT, CrS
Q8NCW5	NAXE	NAD(P)H-hydrate epimerase	WT
Q16527	CSRP2	Cysteine and glycine-rich protein 2	WT, CrS
Q15637	SF1	Splicing factor 1	WT
P31689	DNAJA1	DnaJ homolog subfamily A member 1	WT, CrS
A0A087WXM6	RPL17	60S ribosomal protein L17	WT, CrS
Q9UG54	MAP3K7	Mitogen-activated protein kinase kinase kinase	WT, CrS
Q6Y7W6	GIGYF2	GRB10-interacting GYF protein 2	WT, CrS
P08708	RPS17	40S ribosomal protein S17	WT, CrS
K4DI93	CUL4B	Cullin 4B, isoform CRA_e	WT, CrS
P63010	AP2B1	AP-2 complex subunit beta	WT
Q9NZI8	IGF2BP1	Insulin-like growth factor 2 mRNA-binding protein 1	WT, CrS
P57772	EEFSEC	Selenocysteine-specific elongation factor	WT
Q96AG4	LRRC59	Leucine-rich repeat-containing protein 59	WT, CrS
P43246	MSH2	DNA mismatch repair protein Msh2	WT, CrS
Q13136	PPFIA1	Liprin-alpha-1	WT, CrS
O76021	RSL1D1	Ribosomal L1 domain-containing protein 1	WT, CrS
Q9UNM6	PSMD13	26S proteasome non-ATPase regulatory subunit 13	WT
A0A1B0GWF2	STXBP1	Syntaxin-binding protein 1	WT, CrS
Q08J23	NSUN2	RNA cytosine C(5)-methyltransferase NSUN2	WT, CrS
Q96F86	EDC3	Enhancer of mRNA-decapping protein 3	WT
O75569	PRKRA	Interferon-inducible double-stranded RNA-dependent protein kinase activator A	WT, CrS
A0A087X0K9	TJP1	Tight junction protein ZO-1	WT
J3QR09	RPL19	Ribosomal protein L19	WT, CrS
Q15154	PCM1	Pericentriolar material 1 protein	WT
O60701	UGDH	UDP-glucose 6-dehydrogenase	WT
D6R938	CAMK2D	Calcium/calmodulin-dependent protein kinase	WT
Q13451	FKBP5	Peptidyl-prolyl cis-trans isomerase FKBP5	WT, CrS
P62829	RPL23	60S ribosomal protein L23	WT, CrS
O75874	IDH1	Isocitrate dehydrogenase [NADP] cytoplasmic	WT, CrS
P56545	CTBP2	C-terminal-binding protein 2	WT
P30084	ECHS1	Enoyl-CoA hydratase, mitochondrial	WT, CrS
O75150	RNF40	E3 ubiquitin-protein ligase BRE1B	WT
Q9Y613	FHOD1	FH1/FH2 domain-containing protein 1	WT
H7C5E4	XRN1	5'-3' exoribonuclease 1	WT, CrS
Q15021	NCAPD2	Condensin complex subunit 1	WT
P50995	ANXA11	Annexin A11	WT
O43290	SART1	U4/U6.U5 tri-snRNP-associated protein 1	WT
P30153	PPP2R1A	Serine/threonine-protein phosphatase 2A 65 kDa regulatory subunit A alpha isoform	WT, CrS
Q15555	MAPRE2	Microtubule-associated protein RP/EB family member 2	WT
Q8WYP5	AHCTF1	Protein ELYS	WT, CrS
O43390	HNRNPR	Heterogeneous nuclear ribonucleoprotein R	WT, CrS
Q9Y2Z0	SUGT1	Protein SGT1 homolog	WT, CrS
Q96G46	DUS3L	tRNA-dihydrouridine(47) synthase [NAD(P)(+)]-like	WT
Q9ULX3	NOB1	RNA-binding protein NOB1	WT
Q14847	LASP1	LIM and SH3 domain protein 1	WT
O60783	MRPS14	28S ribosomal protein S14, mitochondrial	WT
P61586	RHOA	Transforming protein RhoA	WT
P08754	GNAI3	Guanine nucleotide-binding protein G(i) subunit alpha	WT, CrS
P49591	SARS1	Serine--tRNA ligase, cytoplasmic	WT
A0A087WY55	VTA1	Chromosome 6 open reading frame 55, isoform CRA_b	WT, CrS
P56192	MARS1	Methionine--tRNA ligase, cytoplasmic	WT
Q5SW79	CEP170	Centrosomal protein of 170 kDa	WT, CrS
Q9Y490	TLN1	Talin-1	WT, CrS
P42765	ACAA2	3-ketoacyl-CoA thiolase, mitochondrial	WT, CrS
Q9BY77	POLDIP3	Polymerase delta-interacting protein 3	WT
P55265	ADAR	Double-stranded RNA-specific adenosine deaminase	WT, CrS
B4DY08	HNRNPC	Heterogeneous nuclear ribonucleoproteins C1/C2	WT
Q92878	RAD50	DNA repair protein RAD50	WT
Q99569	PKP4	Plakophilin-4	WT, CrS
P42704	LRPPRC	Leucine-rich PPR motif-containing protein, mitochondrial	WT, CrS
Q9UPQ9	TNRC6B	Trinucleotide repeat-containing gene 6B protein	WT, CrS
O14654	IRS4	Insulin receptor substrate 4	WT
P33991	MCM4	DNA replication licensing factor MCM4	WT, CrS
Q9UHD8	SEPTIN9	Septin-9	WT
P00568	AK1	Adenylate kinase isoenzyme 1	WT, CrS
Q9BW92	TARS2	Threonine--tRNA ligase, mitochondrial	WT
Q96J01	THOC3	THO complex subunit 3	WT
Q9BXJ9	NAA15	N-alpha-acetyltransferase 15, NatA auxiliary subunit	WT
P46939	UTRN	Utrophin	WT, CrS
P11387	TOP1	DNA topoisomerase 1	WT, CrS
H0YDU8	PPP5C	Serine/threonine-protein phosphatase	WT
P49368	CCT3	T-complex protein 1 subunit gamma	WT, CrS
P18615	NELFE	Negative elongation factor E	WT, CrS
P27708	CAD	CAD protein [Includes: Glutamine-dependent carbamoyl-phosphate synthase	WT, CrS
J3KTA4	DDX5	DEAD box protein 5	WT
P60660	MYL6	Myosin light polypeptide 6	WT, CrS
O95785	WIZ	Protein Wiz	WT
A6NDG6	PGP	Glycerol-3-phosphate phosphatase	WT
P62269	RPS18	40S ribosomal protein S18	WT
A0A0U1RRM6	ENAH	Protein enabled homolog	WT, CrS
P38919	EIF4A3	Eukaryotic initiation factor 4A-III	WT
O75643	SNRNP200	U5 small nuclear ribonucleoprotein 200 kDa helicase	WT
Q9H840	GEMIN7	Gem-associated protein 7	WT, CrS
Q15424	SAFB	Scaffold attachment factor B1	WT
O15160	POLR1C	DNA-directed RNA polymerases I and III subunit RPAC1	WT, CrS
P55795	HNRNPH2	Heterogeneous nuclear ribonucleoprotein H2	WT, CrS
B1AK87	CAPZB	F-actin-capping protein subunit beta	WT, CrS
Q5QJE6	DNTTIP2	Deoxynucleotidyltransferase terminal-interacting protein 2	WT
Q8WXF1	PSPC1	Paraspeckle component 1	WT, CrS
Q9NTI5	PDS5B	Sister chromatid cohesion protein PDS5 homolog B	WT, CrS
O00571	DDX3X	ATP-dependent RNA helicase DDX3X	WT
Q8WX93	PALLD	Palladin	WT
Q99497	PARK7	Parkinson disease protein 7	WT, CrS
P62913	RPL11	60S ribosomal protein L11	WT
A0A1C7CYX9	DPYSL2	Dihydropyrimidinase-related protein 2	WT, CrS
P40939	HADHA	Trifunctional enzyme subunit alpha, mitochondrial	WT, CrS
A0A0A0MRM8	MYO6	Unconventional myosin-6	WT, CrS
P29144	TPP2	Tripeptidyl-peptidase 2	WT, CrS
Q9BWF3	RBM4	RNA-binding protein 4	WT
P35658	NUP214	Nuclear pore complex protein Nup214	WT, CrS
F8W0J6	NAP1L1	Nucleosome assembly protein 1-like 1	WT, CrS
O95347	SMC2	Structural maintenance of chromosomes protein 2	WT
Q99613	EIF3C	Eukaryotic translation initiation factor 3 subunit C	WT
P38117	ETFB	Electron transfer flavoprotein subunit beta	WT, CrS
A0A0A6YYL4	CORO7-PAM16	Coronin	WT
O75534	CSDE1	Cold shock domain-containing protein E1	WT
O15226	NKRF	NF-kappa-B-repressing factor	WT
P46776	RPL27A	60S ribosomal protein L27a	WT, CrS
P41252	IARS1	Isoleucine--tRNA ligase, cytoplasmic	WT, CrS
Q9NVH2	INTS7	Integrator complex subunit 7	WT
Q9GZL7	WDR12	Ribosome biogenesis protein WDR12	WT
P55060	CSE1L	Exportin-2	WT, CrS
P25325	MPST	3-mercaptopyruvate sulfurtransferase	WT, CrS
P61758	VBP1	Prefoldin subunit 3	WT
P51572	BCAP31	B-cell receptor-associated protein 31	WT
Q86Y56	DNAAF5	Dynein assembly factor 5, axonemal	WT, CrS
Q13573	SNW1	SNW domain-containing protein 1	WT
P78371	CCT2	T-complex protein 1 subunit beta	WT
Q9P0K7	RAI14	Ankycorbin	WT, CrS
Q9BTY7	HGH1	Protein HGH1 homolog	WT
Q13625	TP53BP2	Apoptosis-stimulating of p53 protein 2	WT
P09525	ANXA4	Annexin A4	WT
P52701	MSH6	DNA mismatch repair protein Msh6	WT, CrS
Q14690	PDCD11	Protein RRP5 homolog	WT
P62633	CNBP	Cellular nucleic acid-binding protein	WT
P13861	PRKAR2A	cAMP-dependent protein kinase type II-alpha regulatory subunit	WT, CrS
Q9BRX2	PELO	Protein pelota homolog	WT, CrS
O60884	DNAJA2	DnaJ homolog subfamily A member 2	WT
P19105	MYL12A	Myosin regulatory light chain 12A	WT, CrS
A0A087WVM4	MTHFD1L	Formyltetrahydrofolate synthetase	WT, CrS
P10606	COX5B	Cytochrome c oxidase subunit 5B, mitochondrial	WT
Q99460	PSMD1	26S proteasome non-ATPase regulatory subunit 1	WT, CrS
B4DLN1	B4DLN1	cDNA FLJ60124, highly similar to Mitochondrial dicarboxylate carrier	WT
Q9NXH9	TRMT1	tRNA	WT
P33176	KIF5B	Kinesin-1 heavy chain	WT, CrS
P26640	VARS1	Valine--tRNA ligase	WT, CrS
Q99959	PKP2	Plakophilin-2	WT, CrS
P40227	CCT6A	T-complex protein 1 subunit zeta	WT
Q9UJU6	DBNL	Drebrin-like protein	WT
P46783	RPS10	40S ribosomal protein S10	WT
P29692	EEF1D	Elongation factor 1-delta	WT, CrS
A0A2R8Y5F1	TSC2	Tuberin	WT
Q9NR45	NANS	Sialic acid synthase	WT, CrS
A0A0G2JNZ2	SCRIB	Protein scribble homolog	WT, CrS
P52272	HNRNPM	Heterogeneous nuclear ribonucleoprotein M	WT, CrS
Q9P2R3	ANKFY1	Rabankyrin-5	WT
O75152	ZC3H11A	Zinc finger CCCH domain-containing protein 11A	WT, CrS
O75792	RNASEH2A	Ribonuclease H2 subunit A	WT, CrS
P62906	RPL10A	60S ribosomal protein L10a	WT, CrS
A0A2R8Y880	RBBP6	E3 ubiquitin-protein ligase RBBP6	WT
P36578	RPL4	60S ribosomal protein L4	WT
Q9BZE9	ASPSCR1	Tether containing UBX domain for GLUT4	WT, CrS
P52294	KPNA1	Importin subunit alpha-5	WT, CrS
G5EA36	CDC27	Cell division cycle 27, isoform CRA_c	WT
Q9Y6G9	DYNC1LI1	Cytoplasmic dynein 1 light intermediate chain 1	WT, CrS
Q13155	AIMP2	Aminoacyl tRNA synthase complex-interacting multifunctional protein 2	WT
P09110	ACAA1	3-ketoacyl-CoA thiolase, peroxisomal	WT, CrS
P11172	UMPS	Uridine 5'-monophosphate synthase	WT
M0R3C3	TECR	Very-long-chain enoyl-CoA reductase	WT, CrS
Q5TFE4	NT5DC1	5'-nucleotidase domain-containing protein 1	WT
P34897	SHMT2	Serine hydroxymethyltransferase, mitochondrial	WT
Q8IWB7	WDFY1	WD repeat and FYVE domain-containing protein 1	WT
Q9UBT2	UBA2	SUMO-activating enzyme subunit 2	WT, CrS
Q8WVJ2	NUDCD2	NudC domain-containing protein 2	WT
Q7Z417	NUFIP2	Nuclear fragile X mental retardation-interacting protein 2	WT
Q02790	FKBP4	Peptidyl-prolyl cis-trans isomerase FKBP4	WT, CrS
Q9NSV4	DIAPH3	Protein diaphanous homolog 3	WT
P07814	EPRS1	Bifunctional glutamate/proline--tRNA ligase	WT, CrS
Q16658	FSCN1	Fascin	WT, CrS
Q13148	TARDBP	TAR DNA-binding protein 43	WT
P53621	COPA	Coatomer subunit alpha	WT, CrS
Q9Y3I0	RTCB	RNA-splicing ligase RtcB homolog	WT, CrS
Q9BT25	HAUS8	HAUS augmin-like complex subunit 8	WT
O00399	DCTN6	Dynactin subunit 6	WT, CrS
O43159	RRP8	Ribosomal RNA-processing protein 8	WT, CrS
Q8IYI6	EXOC8	Exocyst complex component 8	WT, CrS
A0A140T9R1	FLOT1	Flotillin	WT
K7EJL1	AP1M1	AP-1 complex subunit mu-1	WT, CrS
Q92973	TNPO1	Transportin-1	WT, CrS
Q5BKZ1	ZNF326	DBIRD complex subunit ZNF326	WT
Q15084	PDIA6	Protein disulfide-isomerase A6	WT, CrS
C9JRJ5	LIMD1	LIM domain-containing protein 1	WT, CrS
Q9Y2L1	DIS3	Exosome complex exonuclease RRP44	WT, CrS
A0A0C4DGA6	HLTF	Helicase-like transcription factor	WT
Q14157	UBAP2L	Ubiquitin-associated protein 2-like	WT, CrS
Q9Y5A9	YTHDF2	YTH domain-containing family protein 2	WT, CrS
P25685	DNAJB1	DnaJ homolog subfamily B member 1	WT, CrS
P50502	ST13	Hsc70-interacting protein	WT, CrS
M0QXL5	FBL	rRNA 2'-O-methyltransferase fibrillarin	WT, CrS
Q8WUM4	PDCD6IP	Programmed cell death 6-interacting protein	WT, CrS
Q9UKF6	CPSF3	Cleavage and polyadenylation specificity factor subunit 3	WT
P62316	SNRPD2	Small nuclear ribonucleoprotein Sm D2	WT, CrS
Q13435	SF3B2	Splicing factor 3B subunit 2	WT
P11177	PDHB	Pyruvate dehydrogenase E1 component subunit beta, mitochondrial	WT
Q9UQ35	SRRM2	Serine/arginine repetitive matrix protein 2	WT
O94979	SEC31A	Protein transport protein Sec31A	WT, CrS
P37108	SRP14	Signal recognition particle 14 kDa protein	WT, CrS
Q7Z406	MYH14	Myosin-14	WT, CrS
G3V1C3	API5	Apoptosis inhibitor 5	WT, CrS
P13807	GYS1	Glycogen [starch] synthase, muscle	WT, CrS
E9PF10	NUP155	Nuclear pore complex protein Nup155	WT, CrS
Q1KMD3	HNRNPUL2	Heterogeneous nuclear ribonucleoprotein U-like protein 2	WT, CrS
P41240	CSK	Tyrosine-protein kinase CSK	WT, CrS
O15020	SPTBN2	Spectrin beta chain, non-erythrocytic 2	WT
P35250	RFC2	Replication factor C subunit 2	WT
Q8WUH6	TMEM263	Transmembrane protein 263	WT, CrS
P46779	RPL28	60S ribosomal protein L28	WT, CrS
Q9BSD7	NTPCR	Cancer-related nucleoside-triphosphatase	WT
Q06210	GFPT1	Glutamine--fructose-6-phosphate aminotransferase [isomerizing] 1	WT
Q2TAL8	QRICH1	Glutamine-rich protein 1	WT
Q9BQ69	MACROD1	ADP-ribose glycohydrolase MACROD1	WT, CrS
P48444	ARCN1	Coatomer subunit delta	WT, CrS
Q16204	CCDC6	Coiled-coil domain-containing protein 6	WT, CrS
Q15031	LARS2	Probable leucine--tRNA ligase, mitochondrial	WT
Q06124	PTPN11	Tyrosine-protein phosphatase non-receptor type 11	WT, CrS
P14866	HNRNPL	Heterogeneous nuclear ribonucleoprotein L	WT
X1WI28	RPL10	60S ribosomal protein L10	WT, CrS
Q9UGV2	NDRG3	Protein NDRG3	WT, CrS
O95336	PGLS	6-phosphogluconolactonase	WT, CrS
Q9C0C2	TNKS1BP1	182 kDa tankyrase-1-binding protein	WT, CrS
Q07021	C1QBP	Complement component 1 Q subcomponent-binding protein, mitochondrial	WT, CrS
P18583	SON	Protein SON	WT
Q6YN16	HSDL2	Hydroxysteroid dehydrogenase-like protein 2	WT, CrS
Q92665	MRPS31	28S ribosomal protein S31, mitochondrial	WT
Q8IZ83	ALDH16A1	Aldehyde dehydrogenase family 16 member A1	WT
P62917	RPL8	60S ribosomal protein L8	WT
Q13618	CUL3	Cullin-3	WT
P31948	STIP1	Stress-induced-phosphoprotein 1	WT, CrS
Q9HC35	EML4	Echinoderm microtubule-associated protein-like 4	WT, CrS
P19022	CDH2	Cadherin-2	WT
B4DDF4	CNN2	Calponin	WT, CrS
Q99426	TBCB	Tubulin-folding cofactor B	WT
Q5JXI8	FHL1	Four and a half LIM domains protein 1	WT, CrS
Q9UKM9	RALY	RNA-binding protein Raly	WT, CrS
P78347	GTF2I	General transcription factor II-I	WT
O95861	BPNT1	3'(2'),5'-bisphosphate nucleotidase 1	WT, CrS
P50991	CCT4	T-complex protein 1 subunit delta	WT, CrS
Q92597	NDRG1	Protein NDRG1	WT
A0A087X1A5	STAU1	Double-stranded RNA-binding protein Staufen homolog 1	WT, CrS
P09622	DLD	Dihydrolipoyl dehydrogenase, mitochondrial	WT
O75131	CPNE3	Copine-3	WT, CrS
P61158	ACTR3	Actin-related protein 3	WT, CrS
O00267	SUPT5H	Transcription elongation factor SPT5	WT
P18858	LIG1	DNA ligase 1	WT
Q53EL6	PDCD4	Programmed cell death protein 4	WT
Q9H0E2	TOLLIP	Toll-interacting protein	WT
P39748	FEN1	Flap endonuclease 1	WT, CrS
Q15750	TAB1	TGF-beta-activated kinase 1 and MAP3K7-binding protein 1	WT, CrS
C9J4Z3	RPL37A	60S ribosomal protein L37a	WT
Q05048	CSTF1	Cleavage stimulation factor subunit 1	WT
Q00610	CLTC	Clathrin heavy chain 1	WT, CrS
Q12904	AIMP1	Aminoacyl tRNA synthase complex-interacting multifunctional protein 1	WT, CrS
Q96HC4	PDLIM5	PDZ and LIM domain protein 5	WT, CrS
O43660	PLRG1	Pleiotropic regulator 1	WT, CrS
O14980	XPO1	Exportin-1	WT
P53396	ACLY	ATP-citrate synthase	WT
Q9BV20	MRI1	Methylthioribose-1-phosphate isomerase	WT, CrS
Q8IWS0	PHF6	PHD finger protein 6	WT, CrS
P30520	ADSS2	Adenylosuccinate synthetase isozyme 2	WT, CrS
Q14126	DSG2	Desmoglein-2	WT, CrS
Q9BUF5	TUBB6	Tubulin beta-6 chain	WT, CrS
P26368	U2AF2	Splicing factor U2AF 65 kDa subunit	WT, CrS
O95232	LUC7L3	Luc7-like protein 3	WT
P27540	ARNT	Aryl hydrocarbon receptor nuclear translocator	WT, CrS
Q2TAM5	RELA	RELA protein	WT, CrS
F2Z2T2	XPA	DNA repair protein-complementing XP-A cells	WT
O94906	PRPF6	Pre-mRNA-processing factor 6	WT, CrS
E7EPN9	PRRC2C	Protein PRRC2C	WT, CrS
Q15003	NCAPH	Condensin complex subunit 2	WT, CrS
Q9UPT5	EXOC7	Exocyst complex component 7	WT
P26639	TARS1	Threonine--tRNA ligase 1, cytoplasmic	WT
Q9UQ80	PA2G4	Proliferation-associated protein 2G4	WT, CrS
O43615	TIMM44	Mitochondrial import inner membrane translocase subunit TIM44	WT
Q12849	GRSF1	G-rich sequence factor 1	WT, CrS
P63241	EIF5A	Eukaryotic translation initiation factor 5A-1	WT, CrS
B4E1N1	ARMC6	Armadillo repeat-containing protein 6	WT, CrS
P46459	NSF	Vesicle-fusing ATPase	WT, CrS
P16615	ATP2A2	Sarcoplasmic/endoplasmic reticulum calcium ATPase 2	WT, CrS
P11388	TOP2A	DNA topoisomerase 2-alpha	WT, CrS
Q5UIP0	RIF1	Telomere-associated protein RIF1	WT
Q15008	PSMD6	26S proteasome non-ATPase regulatory subunit 6	WT
Q9BR76	CORO1B	Coronin-1B	WT, CrS
Q15075	EEA1	Early endosome antigen 1	WT, CrS
O15294	OGT	UDP-N-acetylglucosamine--peptide N-acetylglucosaminyltransferase 110 kDa subunit	WT
P55884	EIF3B	Eukaryotic translation initiation factor 3 subunit B	WT, CrS
O60684	KPNA6	Importin subunit alpha-7	WT, CrS
F5H6E2	MYO1C	Unconventional myosin-Ic	WT, CrS
Q5VYK3	ECPAS	Proteasome adapter and scaffold protein ECM29	WT, CrS
Q13045	FLII	Protein flightless-1 homolog	WT, CrS
Q9NWH9	SLTM	SAFB-like transcription modulator	WT, CrS
Q8TAT6	NPLOC4	Nuclear protein localization protein 4 homolog	WT, CrS
Q9NSD9	FARSB	Phenylalanine--tRNA ligase beta subunit	WT, CrS
Q8ND24	RNF214	RING finger protein 214	WT
E7EVA0	MAP4	Microtubule-associated protein	WT, CrS
Q9UHB6	LIMA1	LIM domain and actin-binding protein 1	WT, CrS
Q8WWQ0	PHIP	PH-interacting protein	WT, CrS
Q6IAA8	LAMTOR1	Ragulator complex protein LAMTOR1	WT, CrS
A0A2R8Y855	SMARCE1	SWI/SNF-related matrix-associated actin-dependent regulator of chromatin subfamily E member 1	WT, CrS
Q7Z478	DHX29	ATP-dependent RNA helicase DHX29	WT
P49327	FASN	Fatty acid synthase	WT
O15371	EIF3D	Eukaryotic translation initiation factor 3 subunit D	WT, CrS
Q9HCD5	NCOA5	Nuclear receptor coactivator 5	WT
O15047	SETD1A	Histone-lysine N-methyltransferase SETD1A	WT
P12004	PCNA	Proliferating cell nuclear antigen	WT, CrS
Q9Y3S2	ZNF330	Zinc finger protein 330	WT, CrS
P42696	RBM34	RNA-binding protein 34	WT, CrS
A0A0G2JH68	DIAPH1	Protein diaphanous homolog 1	WT, CrS
Q5JSZ5	PRRC2B	Protein PRRC2B	WT, CrS
O00487	PSMD14	26S proteasome non-ATPase regulatory subunit 14	WT, CrS
Q09666	AHNAK	Neuroblast differentiation-associated protein AHNAK	WT, CrS
Q14C86	GAPVD1	GTPase-activating protein and VPS9 domain-containing protein 1	WT, CrS
Q8ND83	SLAIN1	SLAIN motif-containing protein 1	WT, CrS
Q86TB9	PATL1	Protein PAT1 homolog 1	WT, CrS
P30154	PPP2R1B	Serine/threonine-protein phosphatase 2A 65 kDa regulatory subunit A beta isoform	WT, CrS
Q8IY81	FTSJ3	pre-rRNA 2'-O-ribose RNA methyltransferase FTSJ3	WT, CrS
Q9BVP2	GNL3	Guanine nucleotide-binding protein-like 3	WT, CrS
P62701	RPS4X	40S ribosomal protein S4, X isoform	WT, CrS
Q9Y5Y2	NUBP2	Cytosolic Fe-S cluster assembly factor NUBP2	WT, CrS
P48634	PRRC2A	Protein PRRC2A	WT, CrS
Q9H7E9	C8orf33	UPF0488 protein C8orf33	WT, CrS
Q9Y6Y8	SEC23IP	SEC23-interacting protein	WT, CrS
Q9C0C9	UBE2O	(E3-independent) E2 ubiquitin-conjugating enzyme	WT, CrS
P33993	MCM7	DNA replication licensing factor MCM7	WT, CrS
P22102	GART	Trifunctional purine biosynthetic protein adenosine-3 [Includes: Phosphoribosylamine--glycine ligase	WT, CrS
O00170	AIP	AH receptor-interacting protein	WT, CrS
Q8IWZ3	ANKHD1	Ankyrin repeat and KH domain-containing protein 1	WT, CrS
P51452	DUSP3	Dual specificity protein phosphatase 3	WT, CrS
Q9NQT5	EXOSC3	Exosome complex component RRP40	WT, CrS
O94826	TOMM70	Mitochondrial import receptor subunit TOM70	WT, CrS
Q16543	CDC37	Hsp90 co-chaperone Cdc37	WT, CrS
Q9UMS4	PRPF19	Pre-mRNA-processing factor 19	WT, CrS
Q9UPN7	PPP6R1	Serine/threonine-protein phosphatase 6 regulatory subunit 1	WT, CrS
Q9Y450	HBS1L	HBS1-like protein	WT, CrS
H0Y5D5	CIZ1	Cip1-interacting zinc finger protein	WT, CrS
E9PKP7	UBTF	Nucleolar transcription factor 1	WT, CrS
Q9BSH4	TACO1	Translational activator of cytochrome c oxidase 1	WT, CrS
O60832	DKC1	H/ACA ribonucleoprotein complex subunit DKC1	WT, CrS
Q9NY93	DDX56	Probable ATP-dependent RNA helicase DDX56	WT, CrS
Q13347	EIF3I	Eukaryotic translation initiation factor 3 subunit I	WT, CrS
O95573	ACSL3	Long-chain-fatty-acid--CoA ligase 3	WT, CrS
Q15370	ELOB	Elongin-B	CrS
P62244	RPS15A	40S ribosomal protein S15a	WT, CrS
Q00796	SORD	Sorbitol dehydrogenase	WT, CrS
E9PGZ1	CALD1	Caldesmon	CrS
P30566	ADSL	Adenylosuccinate lyase	WT, CrS
P49736	MCM2	DNA replication licensing factor MCM2	WT, CrS
Q15126	PMVK	Phosphomevalonate kinase	WT, CrS
Q9BY44	EIF2A	Eukaryotic translation initiation factor 2A	CrS
P25205	MCM3	DNA replication licensing factor MCM3	WT, CrS
P55735	SEC13	Protein SEC13 homolog	WT, CrS
Q9BWD1	ACAT2	Acetyl-CoA acetyltransferase, cytosolic	WT, CrS
Q9UN86	G3BP2	Ras GTPase-activating protein-binding protein 2	WT, CrS
Q9H0D6	XRN2	5'-3' exoribonuclease 2	WT, CrS
O95816	BAG2	BAG family molecular chaperone regulator 2	WT, CrS
Q8N0X7	SPART	Spartin	WT, CrS
P62333	PSMC6	26S proteasome regulatory subunit 10B	WT, CrS
Q96C36	PYCR2	Pyrroline-5-carboxylate reductase 2	WT, CrS
Q8IWX8	CHERP	Calcium homeostasis endoplasmic reticulum protein	WT, CrS
C9J0J7	PFN2	Profilin	CrS
Q16822	PCK2	Phosphoenolpyruvate carboxykinase [GTP], mitochondrial	WT, CrS
Q13144	EIF2B5	Translation initiation factor eIF-2B subunit epsilon	WT, CrS
P45974	USP5	Ubiquitin carboxyl-terminal hydrolase 5	WT, CrS
Q15417	CNN3	Calponin-3	WT, CrS
A0A087WTZ5	UBXN1	UBX domain-containing protein 1	WT, CrS
O43395	PRPF3	U4/U6 small nuclear ribonucleoprotein Prp3	WT, CrS
Q8NCA5	FAM98A	Protein FAM98A	WT, CrS
P27694	RPA1	Replication protein A 70 kDa DNA-binding subunit	WT, CrS
Q13526	PIN1	Peptidyl-prolyl cis-trans isomerase NIMA-interacting 1	CrS
P63092	GNAS	Guanine nucleotide-binding protein G(s) subunit alpha isoforms short	CrS
O95747	OXSR1	Serine/threonine-protein kinase OSR1	WT, CrS
P21964	COMT	Catechol O-methyltransferase	WT, CrS
Q3ZCQ8	TIMM50	Mitochondrial import inner membrane translocase subunit TIM50	CrS
Q5T7U1	GTF3C5	General transcription factor 3C polypeptide 5	WT, CrS
Q86V48	LUZP1	Leucine zipper protein 1	WT, CrS
P35579	MYH9	Myosin-9	WT, CrS
P49321	NASP	Nuclear autoantigenic sperm protein	WT, CrS
E5RGR0	LYPLA1	Acyl-protein thioesterase 1	CrS
P54136	RARS1	Arginine--tRNA ligase, cytoplasmic	WT, CrS
P50542	PEX5	Peroxisomal targeting signal 1 receptor	WT, CrS
P54646	PRKAA2	5'-AMP-activated protein kinase catalytic subunit alpha-2	CrS
Q9BZE4	GTPBP4	Nucleolar GTP-binding protein 1	WT, CrS
Q13907	IDI1	Isopentenyl-diphosphate Delta-isomerase 1	WT, CrS
E7ETK0	RPS24	40S ribosomal protein S24	WT, CrS
P35580	MYH10	Myosin-10	WT, CrS
P49588	AARS1	Alanine--tRNA ligase, cytoplasmic	WT, CrS
Q9ULC4	MCTS1	Malignant T-cell-amplified sequence 1	CrS
P52594	AGFG1	Arf-GAP domain and FG repeat-containing protein 1	CrS
Q14247	CTTN	Src substrate cortactin	WT, CrS
P45973	CBX5	Chromobox protein homolog 5	CrS
P52788	SMS	Spermine synthase	WT, CrS
P62195	PSMC5	26S proteasome regulatory subunit 8	WT, CrS
Q9UN37	VPS4A	Vacuolar protein sorting-associated protein 4A	WT, CrS
O75436	VPS26A	Vacuolar protein sorting-associated protein 26A	WT, CrS
P17987	TCP1	T-complex protein 1 subunit alpha	WT, CrS
Q9BTE6	AARSD1	Alanyl-tRNA editing protein Aarsd1	WT, CrS
Q96GD0	PDXP	Pyridoxal phosphate phosphatase	WT, CrS
Q15365	PCBP1	Poly(rC)-binding protein 1	WT, CrS
Q7L0Y3	TRMT10C	tRNA methyltransferase 10 homolog C	WT, CrS
Q9UNY4	TTF2	Transcription termination factor 2	WT, CrS
O95373	IPO7	Importin-7	WT, CrS
P36542	ATP5F1C	ATP synthase subunit gamma, mitochondrial	CrS
Q6ZN17	LIN28B	Protein lin-28 homolog B	CrS
O75934	BCAS2	Pre-mRNA-splicing factor SPF27	CrS
Q99829	CPNE1	Copine-1	CrS
P06132	UROD	Uroporphyrinogen decarboxylase	WT, CrS
Q14166	TTLL12	Tubulin--tyrosine ligase-like protein 12	WT, CrS
P43490	NAMPT	Nicotinamide phosphoribosyltransferase	WT, CrS
Q9BSJ8	ESYT1	Extended synaptotagmin-1	WT, CrS
O43143	DHX15	Pre-mRNA-splicing factor ATP-dependent RNA helicase DHX15	WT, CrS
P22314	UBA1	Ubiquitin-like modifier-activating enzyme 1	WT, CrS
P62241	RPS8	40S ribosomal protein S8	WT, CrS
O43719	HTATSF1	HIV Tat-specific factor 1	WT, CrS
O14893	GEMIN2	Gem-associated protein 2	CrS
P09543	CNP	2',3'-cyclic-nucleotide 3'-phosphodiesterase	WT, CrS
P13489	RNH1	Ribonuclease inhibitor	CrS
Q01469	FABP5	Fatty acid-binding protein 5	WT, CrS
E9PDU5	WDR6	WD repeat-containing protein 6	WT, CrS
Q92797	SYMPK	Symplekin	WT, CrS

Q13428	TCOF1	Treacle protein	WT, CrS
Q4KMP7	TBC1D10B	TBC1 domain family member 10B	WT, CrS
P26358	DNMT1	DNA	CrS
Q5T6F2	UBAP2	Ubiquitin-associated protein 2	WT, CrS
P43487	RANBP1	Ran-specific GTPase-activating protein	CrS
Q92667	AKAP1	A-kinase anchor protein 1, mitochondrial	WT, CrS
P98175	RBM10	RNA-binding protein 10	CrS
P31946	YWHAB	14-3-3 protein beta/alpha	CrS
P13797	PLS3	Plastin-3	WT, CrS
Q9UJC3	HOOK1	Protein Hook homolog 1	WT, CrS
Q9NW82	WDR70	WD repeat-containing protein 70	CrS
P43897	TSFM	Elongation factor Ts, mitochondrial	WT, CrS
Q9Y696	CLIC4	Chloride intracellular channel protein 4	CrS
P40937	RFC5	Replication factor C subunit 5	WT, CrS
P39687	ANP32A	Acidic leucine-rich nuclear phosphoprotein 32 family member A	CrS
Q9BYG3	NIFK	MKI67 FHA domain-interacting nucleolar phosphoprotein	WT, CrS
Q9Y2R4	DDX52	Probable ATP-dependent RNA helicase DDX52	CrS
Q86VP6	CAND1	Cullin-associated NEDD8-dissociated protein 1	WT, CrS
P48643	CCT5	T-complex protein 1 subunit epsilon	WT, CrS
A0A3B3IRI2	CTPS1	CTP synthase	WT, CrS
O00154	ACOT7	Cytosolic acyl coenzyme A thioester hydrolase	WT, CrS
P31939	ATIC	Bifunctional purine biosynthesis protein ATIC	CrS
Q9NQ88	TIGAR	Fructose-2,6-bisphosphatase TIGAR	CrS
Q14318	FKBP8	Peptidyl-prolyl cis-trans isomerase FKBP8	CrS
Q9UHB9	SRP68	Signal recognition particle subunit SRP68	WT, CrS
P51116	FXR2	Fragile X mental retardation syndrome-related protein 2	CrS
Q9UJ83	HACL1	2-hydroxyacyl-CoA lyase 1	CrS
P53618	COPB1	Coatomer subunit beta	WT, CrS
P42566	EPS15	Epidermal growth factor receptor substrate 15	CrS
O75439	PMPCB	Mitochondrial-processing peptidase subunit beta	CrS
G5EA31	SEC24C	Protein transport protein Sec24C	CrS
F8VYE8	PPP1CC	Serine/threonine-protein phosphatase	CrS
Q9NUQ9	CYRIB	CYFIP-related Rac1 interactor B	CrS
Q9BQ67	GRWD1	Glutamate-rich WD repeat-containing protein 1	WT, CrS
G3XAH6	PAPOLA	Poly(A) polymerase	CrS
P84095	RHOG	Rho-related GTP-binding protein RhoG	CrS
Q9NP97	DYNLRB1	Dynein light chain roadblock-type 1	WT, CrS
Q6PKG0	LARP1	La-related protein 1	WT, CrS
Q9HB71	CACYBP	Calcyclin-binding protein	CrS
Q12948	FOXC1	Forkhead box protein C1	WT, CrS
P62753	RPS6	40S ribosomal protein S6	WT, CrS
B7Z7F3	RANBP3	Ran-binding protein 3	WT, CrS
O43795	MYO1B	Unconventional myosin-Ib	CrS
P62258	YWHAE	14-3-3 protein epsilon	WT, CrS
P53384	NUBP1	Cytosolic Fe-S cluster assembly factor NUBP1	CrS
O75663	TIPRL	TIP41-like protein	CrS
P82673	MRPS35	28S ribosomal protein S35, mitochondrial	CrS
P68133	ACTA1	Actin, alpha skeletal muscle	WT, CrS
Q9NQX3	GPHN	Gephyrin [Includes: Molybdopterin adenylyltransferase	CrS
P32322	PYCR1	Pyrroline-5-carboxylate reductase 1, mitochondrial	CrS
Q2TAY7	SMU1	WD40 repeat-containing protein SMU1	CrS
Q96S44	TP53RK	EKC/KEOPS complex subunit TP53RK	CrS
Q9UHD1	CHORDC1	Cysteine and histidine-rich domain-containing protein 1	WT, CrS
Q53H12	AGK	Acylglycerol kinase, mitochondrial	WT, CrS
Q7LBC6	KDM3B	Lysine-specific demethylase 3B	WT, CrS
H0YNW5	DUT	Deoxyuridine 5'-triphosphate nucleotidohydrolase	CrS
P34949	MPI	Mannose-6-phosphate isomerase	CrS
Q16643	DBN1	Drebrin	CrS
Q6P587	FAHD1	Acylpyruvase FAHD1, mitochondrial	WT, CrS
Q8NFC6	BOD1L1	Biorientation of chromosomes in cell division protein 1-like 1	CrS
P42771	CDKN2A	Cyclin-dependent kinase inhibitor 2A	CrS
Q9UBE0	SAE1	SUMO-activating enzyme subunit 1	CrS
Q9UKD2	MRTO4	mRNA turnover protein 4 homolog	WT, CrS
Q9P2I0	CPSF2	Cleavage and polyadenylation specificity factor subunit 2	CrS
A0A024RAC6	ELOA	Elongin-A	CrS
Q96AC1	FERMT2	Fermitin family homolog 2	WT, CrS
A0A0C4DGQ6	RPRD1A	Regulation of nuclear pre-mRNA domain-containing protein 1A	CrS
P35268	RPL22	60S ribosomal protein L22	WT, CrS
Q9Y2U8	LEMD3	Inner nuclear membrane protein Man1	CrS
Q14694	USP10	Ubiquitin carboxyl-terminal hydrolase 10	CrS
O75533	SF3B1	Splicing factor 3B subunit 1	CrS
Q9BPU6	DPYSL5	Dihydropyrimidinase-related protein 5	CrS
E9PLK3	NPEPPS	Aminopeptidase	CrS
A1X283	SH3PXD2B	SH3 and PX domain-containing protein 2B	CrS
Q9Y2X3	NOP58	Nucleolar protein 58	CrS
P55081	MFAP1	Microfibrillar-associated protein 1	CrS
O60343	TBC1D4	TBC1 domain family member 4	CrS
P42285	MTREX	Exosome RNA helicase MTR4	CrS
Q96A33	CCDC47	Coiled-coil domain-containing protein 47	CrS
O14802	POLR3A	DNA-directed RNA polymerase III subunit RPC1	CrS
P63173	RPL38	60S ribosomal protein L38	CrS
P40222	TXLNA	Alpha-taxilin	CrS
Q8WWY3	PRPF31	U4/U6 small nuclear ribonucleoprotein Prp31	CrS
O00178	GTPBP1	GTP-binding protein 1	CrS
Q12955	ANK3	Ankyrin-3	CrS
Q9UHV9	PFDN2	Prefoldin subunit 2	WT, CrS
P62854	RPS26	40S ribosomal protein S26	CrS
O75832	PSMD10	26S proteasome non-ATPase regulatory subunit 10	WT, CrS
O75531	BANF1	Barrier-to-autointegration factor	WT, CrS
Q15061	WDR43	WD repeat-containing protein 43	CrS
Q8NC51	SERBP1	Plasminogen activator inhibitor 1 RNA-binding protein	CrS
Q13185	CBX3	Chromobox protein homolog 3	WT, CrS
O14744	PRMT5	Protein arginine N-methyltransferase 5	WT, CrS
Q96DH6	MSI2	RNA-binding protein Musashi homolog 2	WT, CrS
P11908	PRPS2	Ribose-phosphate pyrophosphokinase 2	CrS
Q96FW1	OTUB1	Ubiquitin thioesterase OTUB1	CrS
Q96P70	IPO9	Importin-9	CrS
Q15645	TRIP13	Pachytene checkpoint protein 2 homolog	CrS
Q9NXF7	DCAF16	DDB1- and CUL4-associated factor 16	CrS
Q32MZ4	LRRFIP1	Leucine-rich repeat flightless-interacting protein 1	WT, CrS
P05198	EIF2S1	Eukaryotic translation initiation factor 2 subunit 1	CrS
Q6P9B6	MEAK7	MTOR-associated protein MEAK7	CrS
O43684	BUB3	Mitotic checkpoint protein BUB3	CrS
P51553	IDH3G	Isocitrate dehydrogenase [NAD] subunit gamma, mitochondrial	CrS
Q9H3K6	BOLA2	BolA-like protein 2	CrS
Q9P258	RCC2	Protein RCC2	CrS
C9J0I9	ZC3HC1	Nuclear-interacting partner of ALK	CrS
Q96F45	ZNF503	Zinc finger protein 503	CrS
P62873	GNB1	Guanine nucleotide-binding protein G(I)/G(S)/G(T) subunit beta-1	CrS
O14929	HAT1	Histone acetyltransferase type B catalytic subunit	CrS
Q6WKZ4	RAB11FIP1	Rab11 family-interacting protein 1	CrS
P46821	MAP1B	Microtubule-associated protein 1B	CrS
Q16576	RBBP7	Histone-binding protein RBBP7	CrS
P27824	CANX	Calnexin	WT, CrS
A0A087X0M4	SLC4A1AP	Kanadaptin	CrS
Q8IYS1	PM20D2	Peptidase M20 domain-containing protein 2	CrS
P11802	CDK4	Cyclin-dependent kinase 4	CrS
Q9Y6A5	TACC3	Transforming acidic coiled-coil-containing protein 3	WT, CrS
O60784	TOM1	Target of Myb protein 1	CrS
Q7L2H7	EIF3M	Eukaryotic translation initiation factor 3 subunit M	CrS
Q9Y262	EIF3L	Eukaryotic translation initiation factor 3 subunit L	CrS
O00148	DDX39A	ATP-dependent RNA helicase DDX39A	CrS
P22061	PCMT1	Protein-L-isoaspartate(D-aspartate) O-methyltransferase	CrS
**PRDX2 cysteine-dependent interactors**			
Q9BQA1	WDR77	Methylosome protein 50	WT, CrS
Q6FI81	CIAPIN1	Anamorsin	WT
E9PGZ1	CALD1	Caldesmon	WT, CrS
O14744	PRMT5	Protein arginine N-methyltransferase 5	WT, CrS
O14965	AURKA	Aurora kinase A	WT
Q9H3K6	BOLA2	BolA-like protein 2	WT
P48507	GCLM	Glutamate--cysteine ligase regulatory subunit	WT
O60664	PLIN3	Perilipin-3	WT, CrS
Q9NQX3	GPHN	Gephyrin [Includes: Molybdopterin adenylyltransferase	WT, CrS
P10301	RRAS	Ras-related protein R-Ras	WT
Q96KB5	PBK	Lymphokine-activated killer T-cell-originated protein kinase	WT, CrS
Q9UL15	BAG5	BAG family molecular chaperone regulator 5	WT
E7EVA0	MAP4	Microtubule-associated protein	WT, CrS
Q9BTE6	AARSD1	Alanyl-tRNA editing protein Aarsd1	WT
Q5T4K5	CRTC2	CREB-regulated transcription coactivator 2	WT
Q8NC51	SERBP1	Plasminogen activator inhibitor 1 RNA-binding protein	WT, CrS
B4DDF4	CNN2	Calponin	WT
Q9NXV2	KCTD5	BTB/POZ domain-containing protein KCTD5	WT
A0A0A0MRN5	OGFR	Opioid growth factor receptor	WT
Q9Y570	PPME1	Protein phosphatase methylesterase 1	WT, CrS
O00566	MPHOSPH10	U3 small nucleolar ribonucleoprotein protein MPP10	WT
P53985	SLC16A1	Monocarboxylate transporter 1	WT
P35520	CBS	Cystathionine beta-synthase	WT, CrS
Q9H0S4	DDX47	Probable ATP-dependent RNA helicase DDX47	WT
P10599	TXN	Thioredoxin	WT, CrS
Q96IZ0	PAWR	PRKC apoptosis WT1 regulator protein	WT
P45985	MAP2K4	Dual specificity mitogen-activated protein kinase kinase 4	WT
Q13428	TCOF1	Treacle protein	WT, CrS
P85037	FOXK1	Forkhead box protein K1	WT
P07355	ANXA2	Annexin A2	WT, CrS
Q13541	EIF4EBP1	Eukaryotic translation initiation factor 4E-binding protein 1	WT, CrS
Q9ULX6	AKAP8L	A-kinase anchor protein 8-like	WT
O95816	BAG2	BAG family molecular chaperone regulator 2	WT
O96007	MOCS2	Molybdopterin synthase catalytic subunit	WT
Q9HA64	FN3KRP	Ketosamine-3-kinase	WT, CrS
Q15417	CNN3	Calponin-3	WT, CrS
O95104	SCAF4	SR-related and CTD-associated factor 4	WT, CrS
Q15365	PCBP1	Poly(rC)-binding protein 1	WT
Q9P2B4	CTTNBP2NL	CTTNBP2 N-terminal-like protein	WT, CrS
Q12948	FOXC1	Forkhead box protein C1	WT, CrS
Q96F45	ZNF503	Zinc finger protein 503	WT, CrS
O15511	ARPC5	Actin-related protein 2/3 complex subunit 5	WT
Q9NUQ3	TXLNG	Gamma-taxilin	WT, CrS
Q8WYA6	CTNNBL1	Beta-catenin-like protein 1	WT
Q9UI12	ATP6V1H	V-type proton ATPase subunit H	WT
P52888	THOP1	Thimet oligopeptidase	WT, CrS
Q16204	CCDC6	Coiled-coil domain-containing protein 6	WT, CrS
G3XAG1	ZNF512	Zinc finger protein 512	WT
Q9H7E9	C8orf33	UPF0488 protein C8orf33	WT
Q14247	CTTN	Src substrate cortactin	WT, CrS
P82921	MRPS21	28S ribosomal protein S21, mitochondrial	WT
P52907	CAPZA1	F-actin-capping protein subunit alpha-1	WT, CrS
P61758	VBP1	Prefoldin subunit 3	WT, CrS
P49790	NUP153	Nuclear pore complex protein Nup153	WT, CrS
Q8N806	UBR7	Putative E3 ubiquitin-protein ligase UBR7	WT
Q92945	KHSRP	Far upstream element-binding protein 2	WT
O00273	DFFA	DNA fragmentation factor subunit alpha	WT, CrS
P63167	DYNLL1	Dynein light chain 1, cytoplasmic	WT
Q13542	EIF4EBP2	Eukaryotic translation initiation factor 4E-binding protein 2	WT, CrS
Q9UNF1	MAGED2	Melanoma-associated antigen D2	WT, CrS
J3QQZ9	PNPO	Pyridoxal 5'-phosphate synthase	WT
E7EV99	ADD1	Alpha-adducin	WT, CrS
A0A1B0GW38	CBL	E3 ubiquitin-protein ligase CBL	WT, CrS
Q5QPM7	PSMF1	Proteasome inhibitor PI31 subunit	WT, CrS
F5H8D7	XRCC1	DNA repair protein XRCC1	WT, CrS
P26599	PTBP1	Polypyrimidine tract-binding protein 1	WT, CrS
Q16576	RBBP7	Histone-binding protein RBBP7	WT, CrS
Q06124	PTPN11	Tyrosine-protein phosphatase non-receptor type 11	WT, CrS
Q9UHD1	CHORDC1	Cysteine and histidine-rich domain-containing protein 1	WT, CrS
Q8IYS1	PM20D2	Peptidase M20 domain-containing protein 2	WT, CrS
O60826	CCDC22	Coiled-coil domain-containing protein 22	WT, CrS
Q5T6F2	UBAP2	Ubiquitin-associated protein 2	WT, CrS
A0A087WTM1	ROBO1	Roundabout homolog 1	WT, CrS
Q5SW79	CEP170	Centrosomal protein of 170 kDa	WT, CrS
Q7Z434	MAVS	Mitochondrial antiviral-signaling protein	WT, CrS
P49458	SRP9	Signal recognition particle 9 kDa protein	WT
Q9Y3F4	STRAP	Serine-threonine kinase receptor-associated protein	WT, CrS
Q6WKZ4	RAB11FIP1	Rab11 family-interacting protein 1	WT, CrS
P22314	UBA1	Ubiquitin-like modifier-activating enzyme 1	WT, CrS
P49321	NASP	Nuclear autoantigenic sperm protein	WT
Q8WWK9	CKAP2	Cytoskeleton-associated protein 2	WT, CrS
Q96F86	EDC3	Enhancer of mRNA-decapping protein 3	WT, CrS
Q96K76	USP47	Ubiquitin carboxyl-terminal hydrolase 47	WT, CrS
O00629	KPNA4	Importin subunit alpha-3	WT, CrS
Q68EM7	ARHGAP17	Rho GTPase-activating protein 17	WT
Q6PKG0	LARP1	La-related protein 1	WT, CrS
Q8N6M0	OTUD6B	Deubiquitinase OTUD6B	WT, CrS
Q93034	CUL5	Cullin-5	WT, CrS
Q96P47	AGAP3	Arf-GAP with GTPase, ANK repeat and PH domain-containing protein 3	WT
P11413	G6PD	Glucose-6-phosphate 1-dehydrogenase	WT, CrS
Q2NKX8	ERCC6L	DNA excision repair protein ERCC-6-like	WT, CrS
P22234	PAICS	Multifunctional protein ADE2 [Includes: Phosphoribosylaminoimidazole-succinocarboxamide synthase	WT, CrS
Q92783	STAM	Signal transducing adapter molecule 1	WT, CrS
Q86YP4	GATAD2A	Transcriptional repressor p66-alpha	WT, CrS
E7EPN9	PRRC2C	Protein PRRC2C	WT, CrS
P40222	TXLNA	Alpha-taxilin	WT, CrS
P35237	SERPINB6	Serpin B6	WT, CrS
P54646	PRKAA2	5'-AMP-activated protein kinase catalytic subunit alpha-2	WT, CrS
Q9BT25	HAUS8	HAUS augmin-like complex subunit 8	WT, CrS
Q96D09	GPRASP2	G-protein coupled receptor-associated sorting protein 2	WT, CrS
Q9NVP1	DDX18	ATP-dependent RNA helicase DDX18	WT, CrS
A0MZ66	SHTN1	Shootin-1	WT, CrS
P48634	PRRC2A	Protein PRRC2A	WT, CrS
P30740	SERPINB1	Leukocyte elastase inhibitor	WT, CrS
O60832	DKC1	H/ACA ribonucleoprotein complex subunit DKC1	CrS
O14974	PPP1R12A	Protein phosphatase 1 regulatory subunit 12A	WT, CrS
Q13200	PSMD2	26S proteasome non-ATPase regulatory subunit 2	WT, CrS
Q8IWB7	WDFY1	WD repeat and FYVE domain-containing protein 1	WT, CrS
Q6UN15	FIP1L1	Pre-mRNA 3'-end-processing factor FIP1	CrS
Q9UHV9	PFDN2	Prefoldin subunit 2	WT, CrS
C9J0I9	ZC3HC1	Nuclear-interacting partner of ALK	WT, CrS
Q9HCN8	SDF2L1	Stromal cell-derived factor 2-like protein 1	WT, CrS
Q92667	AKAP1	A-kinase anchor protein 1, mitochondrial	WT, CrS
P13797	PLS3	Plastin-3	WT, CrS
Q07666	KHDRBS1	KH domain-containing, RNA-binding, signal transduction-associated protein 1	WT, CrS
Q9ULW0	TPX2	Targeting protein for Xklp2	WT, CrS
P55265	ADAR	Double-stranded RNA-specific adenosine deaminase	CrS
E9PGT1	TSN	Component 3 of promoter of RISC	WT, CrS
Q92769	HDAC2	Histone deacetylase 2	WT, CrS
O00267	SUPT5H	Transcription elongation factor SPT5	CrS
P46939	UTRN	Utrophin	CrS
Q7Z6Z7	HUWE1	E3 ubiquitin-protein ligase HUWE1	WT, CrS
Q92597	NDRG1	Protein NDRG1	CrS
A4D1S0	KLRG2	Killer cell lectin-like receptor subfamily G member 2	WT, CrS
Q9H3S7	PTPN23	Tyrosine-protein phosphatase non-receptor type 23	WT, CrS
O95573	ACSL3	Long-chain-fatty-acid--CoA ligase 3	CrS
Q6P4A7	SFXN4	Sideroflexin-4	CrS
Q9UBT2	UBA2	SUMO-activating enzyme subunit 2	CrS
B1AK87	CAPZB	F-actin-capping protein subunit beta	WT, CrS
Q9NYZ3	GTSE1	G2 and S phase-expressed protein 1	WT, CrS
Q96HC4	PDLIM5	PDZ and LIM domain protein 5	WT, CrS
G8JLH9	STAT3	Signal transducer and activator of transcription	CrS
Q9UN37	VPS4A	Vacuolar protein sorting-associated protein 4A	WT, CrS
Q9H2U2	PPA2	Inorganic pyrophosphatase 2, mitochondrial	WT, CrS
Q5JRA6	MIA3	Transport and Golgi organization protein 1 homolog	WT, CrS
O75223	GGCT	Gamma-glutamylcyclotransferase	WT, CrS
Q7Z4H7	HAUS6	HAUS augmin-like complex subunit 6	CrS
Q9NXF7	DCAF16	DDB1- and CUL4-associated factor 16	WT, CrS
Q2M1P5	KIF7	Kinesin-like protein KIF7	CrS
Q9Y266	NUDC	Nuclear migration protein nudC	WT, CrS
Q14694	USP10	Ubiquitin carboxyl-terminal hydrolase 10	CrS
Q7LBC6	KDM3B	Lysine-specific demethylase 3B	CrS
O75663	TIPRL	TIP41-like protein	WT, CrS
E9PLA9	CAPRIN1	Caprin-1	WT, CrS
O00170	AIP	AH receptor-interacting protein	WT, CrS
Q14240	EIF4A2	Eukaryotic initiation factor 4A-II	WT, CrS
Q9C0C9	UBE2O	(E3-independent) E2 ubiquitin-conjugating enzyme	WT, CrS
O15143	ARPC1B	Actin-related protein 2/3 complex subunit 1B	CrS
Q96T23	RSF1	Remodeling and spacing factor 1	CrS
O60493	SNX3	Sorting nexin-3	CrS
Q9UKX7	NUP50	Nuclear pore complex protein Nup50	CrS
Q92598	HSPH1	Heat shock protein 105 kDa	WT, CrS
S4R3H4	ACIN1	Apoptotic chromatin condensation inducer in the nucleus	CrS
Q8ND82	ZNF280C	Zinc finger protein 280C	CrS
Q9UIG0	BAZ1B	Tyrosine-protein kinase BAZ1B	CrS
Q8NI27	THOC2	THO complex subunit 2	CrS
Q15276	RABEP1	Rab GTPase-binding effector protein 1	CrS
P49903	SEPHS1	Selenide, water dikinase 1	WT, CrS
Q8TAQ2	SMARCC2	SWI/SNF complex subunit SMARCC2	WT, CrS
Q9P258	RCC2	Protein RCC2	WT, CrS
J3KN29	PSMD9	26S proteasome non-ATPase regulatory subunit 9	WT, CrS
O00151	PDLIM1	PDZ and LIM domain protein 1	CrS
P13861	PRKAR2A	cAMP-dependent protein kinase type II-alpha regulatory subunit	CrS
Q5BKZ1	ZNF326	DBIRD complex subunit ZNF326	CrS
Q96KR1	ZFR	Zinc finger RNA-binding protein	CrS
Q9NPH2	ISYNA1	Inositol-3-phosphate synthase 1	WT, CrS
P50213	IDH3A	Isocitrate dehydrogenase [NAD] subunit alpha, mitochondrial	CrS
O60343	TBC1D4	TBC1 domain family member 4	WT, CrS
P49588	AARS1	Alanine--tRNA ligase, cytoplasmic	CrS
A0A3B3ISG5	IDE	Insulin-degrading enzyme	CrS
O43815	STRN	Striatin	CrS
A1X283	SH3PXD2B	SH3 and PX domain-containing protein 2B	CrS
Q6UWP7	LCLAT1	Lysocardiolipin acyltransferase 1	CrS
O95347	SMC2	Structural maintenance of chromosomes protein 2	CrS
P78346	RPP30	Ribonuclease P protein subunit p30	CrS
P63000	RAC1	Ras-related C3 botulinum toxin substrate 1	CrS
Q9NZB2	FAM120A	Constitutive coactivator of PPAR-gamma-like protein 1	CrS
P49750	YLPM1	YLP motif-containing protein 1	CrS
Q8N6T3	ARFGAP1	ADP-ribosylation factor GTPase-activating protein 1	CrS
Q9Y2R9	MRPS7	28S ribosomal protein S7, mitochondrial	CrS
E9PQV9	DCUN1D5	DCN1-like protein	CrS
Q9BXP5	SRRT	Serrate RNA effector molecule homolog	CrS
Q8N3X1	FNBP4	Formin-binding protein 4	WT, CrS
O43143	DHX15	Pre-mRNA-splicing factor ATP-dependent RNA helicase DHX15	WT, CrS
Q9Y5A9	YTHDF2	YTH domain-containing family protein 2	CrS
Q99598	TSNAX	Translin-associated protein X	WT, CrS
P00387	CYB5R3	NADH-cytochrome b5 reductase 3	CrS
P52701	MSH6	DNA mismatch repair protein Msh6	CrS
O94903	PLPBP	Pyridoxal phosphate homeostasis protein	CrS
P42677	RPS27	40S ribosomal protein S27	CrS
Q14739	LBR	Delta(14)-sterol reductase LBR	CrS
A0A024RCR6	BAG6	BCL2-associated athanogene 6	CrS
P51452	DUSP3	Dual specificity protein phosphatase 3	WT, CrS
Q8WVC2	RPS21	40S ribosomal protein S21	WT, CrS
Q9Y295	DRG1	Developmentally-regulated GTP-binding protein 1	CrS
Q6IQ49	SDE2	Replication stress response regulator SDE2	WT, CrS
M0QY97	ZC3H4	Zinc finger CCCH domain-containing protein 4	CrS
Q04917	YWHAH	14-3-3 protein eta	CrS
P52788	SMS	Spermine synthase	CrS
Q96A33	CCDC47	Coiled-coil domain-containing protein 47	CrS
P38159	RBMX	RNA-binding motif protein, X chromosome	CrS
P23588	EIF4B	Eukaryotic translation initiation factor 4B	CrS
A0A087WWP4	RBM15	RNA-binding protein 15	CrS
A0A087WTZ5	UBXN1	UBX domain-containing protein 1	CrS
Q9UQE7	SMC3	Structural maintenance of chromosomes protein 3	WT, CrS
P46821	MAP1B	Microtubule-associated protein 1B	WT, CrS
O95801	TTC4	Tetratricopeptide repeat protein 4	CrS
Q9UJV9	DDX41	Probable ATP-dependent RNA helicase DDX41	CrS
O00487	PSMD14	26S proteasome non-ATPase regulatory subunit 14	CrS
Q8N5F7	NKAP	NF-kappa-B-activating protein	CrS
A0A0A0MRM8	MYO6	Unconventional myosin-6	CrS
P49593	PPM1F	Protein phosphatase 1F	CrS
P25786	PSMA1	Proteasome subunit alpha type-1	CrS
O43809	NUDT21	Cleavage and polyadenylation specificity factor subunit 5	WT, CrS
Q13547	HDAC1	Histone deacetylase 1	WT, CrS
P56385	ATP5ME	ATP synthase subunit e, mitochondrial	CrS
H0YNW5	DUT	Deoxyuridine 5'-triphosphate nucleotidohydrolase	WT, CrS
P11802	CDK4	Cyclin-dependent kinase 4	WT, CrS
P55072	VCP	Transitional endoplasmic reticulum ATPase	CrS
P09936	UCHL1	Ubiquitin carboxyl-terminal hydrolase isozyme L1	CrS
P08240	SRPRA	Signal recognition particle receptor subunit alpha	CrS
P22695	UQCRC2	Cytochrome b-c1 complex subunit 2, mitochondrial	CrS
Q13045	FLII	Protein flightless-1 homolog	CrS
Q9C0C2	TNKS1BP1	182 kDa tankyrase-1-binding protein	WT, CrS
P98175	RBM10	RNA-binding protein 10	CrS
E9PLK3	NPEPPS	Aminopeptidase	CrS
Q8WXF1	PSPC1	Paraspeckle component 1	CrS
P62191	PSMC1	26S proteasome regulatory subunit 4	CrS
Q9GZT9	EGLN1	Egl nine homolog 1	CrS
Q9UPN7	PPP6R1	Serine/threonine-protein phosphatase 6 regulatory subunit 1	CrS
O75533	SF3B1	Splicing factor 3B subunit 1	CrS
Q9BRP1	PDCD2L	Programmed cell death protein 2-like	WT, CrS
Q86V48	LUZP1	Leucine zipper protein 1	WT, CrS
Q15019	SEPTIN2	Septin-2	CrS
Q99504	EYA3	Eyes absent homolog 3	CrS
Q9BWJ5	SF3B5	Splicing factor 3B subunit 5	CrS
A6NGP5	JPT2	Jupiter microtubule-associated homolog 2	WT, CrS
Q9BTE3	MCMBP	Mini-chromosome maintenance complex-binding protein	CrS
Q15046	KARS1	Lysine--tRNA ligase	CrS
Q96GD0	PDXP	Pyridoxal phosphate phosphatase	CrS
P61981	YWHAG	14-3-3 protein gamma	CrS
Q12800	TFCP2	Alpha-globin transcription factor CP2	CrS
Q06203	PPAT	Amidophosphoribosyltransferase	CrS
Q9H773	DCTPP1	dCTP pyrophosphatase 1	WT, CrS
A0A1C7CYX9	DPYSL2	Dihydropyrimidinase-related protein 2	CrS
P19174	PLCG1	1-phosphatidylinositol 4,5-bisphosphate phosphodiesterase gamma-1	WT, CrS
Q14566	MCM6	DNA replication licensing factor MCM6	CrS
Q9UNM6	PSMD13	26S proteasome non-ATPase regulatory subunit 13	CrS
P41214	EIF2D	Eukaryotic translation initiation factor 2D	CrS
H3BRL3	UBFD1	Ubiquitin domain-containing protein UBFD1	CrS
P60228	EIF3E	Eukaryotic translation initiation factor 3 subunit E	CrS
Q9NZL9	MAT2B	Methionine adenosyltransferase 2 subunit beta	CrS
P52732	KIF11	Kinesin-like protein KIF11	CrS
Q96P70	IPO9	Importin-9	CrS
Q14137	BOP1	Ribosome biogenesis protein BOP1	CrS
Q6NUK1	SLC25A24	Calcium-binding mitochondrial carrier protein SCaMC-1	CrS
O43765	SGTA	Small glutamine-rich tetratricopeptide repeat-containing protein alpha	CrS
Q16531	DDB1	DNA damage-binding protein 1	CrS
Q9Y230	RUVBL2	RuvB-like 2	CrS
P06737	PYGL	Glycogen phosphorylase, liver form	CrS
P05023	ATP1A1	Sodium/potassium-transporting ATPase subunit alpha-1	CrS
O14929	HAT1	Histone acetyltransferase type B catalytic subunit	WT, CrS
A0A087WUX8	APOOL	MICOS complex subunit	CrS
Q9Y6Y8	SEC23IP	SEC23-interacting protein	CrS
P58546	MTPN	Myotrophin	CrS
E9PS17	SCYL1	N-terminal kinase-like protein	CrS
P20020	ATP2B1	Plasma membrane calcium-transporting ATPase 1	CrS
O94992	HEXIM1	Protein HEXIM1	CrS
Q32MZ4	LRRFIP1	Leucine-rich repeat flightless-interacting protein 1	WT, CrS
O75381	PEX14	Peroxisomal membrane protein PEX14	CrS
Q15393	SF3B3	Splicing factor 3B subunit 3	CrS
Q8IVM0	CCDC50	Coiled-coil domain-containing protein 50	CrS
P42771	CDKN2A	Cyclin-dependent kinase inhibitor 2A	WT, CrS
O94905	ERLIN2	Erlin-2	CrS
H0YDU8	PPP5C	Serine/threonine-protein phosphatase	CrS
K7EJQ8	HDHD2	Haloacid dehalogenase-like hydrolase domain-containing protein 2	CrS
O43242	PSMD3	26S proteasome non-ATPase regulatory subunit 3	CrS
Q9H936	SLC25A22	Mitochondrial glutamate carrier 1	CrS
P30041	PRDX6	Peroxiredoxin-6	CrS
P15374	UCHL3	Ubiquitin carboxyl-terminal hydrolase isozyme L3	CrS
P29373	CRABP2	Cellular retinoic acid-binding protein 2	CrS
P61106	RAB14	Ras-related protein Rab-14	CrS
P00338	LDHA	L-lactate dehydrogenase A chain	CrS
Q9H6S0	YTHDC2	3'-5' RNA helicase YTHDC2	CrS
Q14C86	GAPVD1	GTPase-activating protein and VPS9 domain-containing protein 1	CrS
A0A0C4DGX4	CUL1	Cullin-1	CrS
Q9UBE0	SAE1	SUMO-activating enzyme subunit 1	CrS
P22570	FDXR	NADPH:adrenodoxin oxidoreductase, mitochondrial	CrS
P31946	YWHAB	14-3-3 protein beta/alpha	CrS
Q9UJZ1	STOML2	Stomatin-like protein 2, mitochondrial	CrS
P22061	PCMT1	Protein-L-isoaspartate(D-aspartate) O-methyltransferase	WT, CrS
Q9H9B4	SFXN1	Sideroflexin-1	CrS
Q9H0U4	RAB1B	Ras-related protein Rab-1B	CrS
P31153	MAT2A	S-adenosylmethionine synthase isoform type-2	CrS
Q6P2Q9	PRPF8	Pre-mRNA-processing-splicing factor 8	CrS
O95684	CEP43	Centrosomal protein 43	CrS
B7Z7F3	RANBP3	Ran-binding protein 3	WT, CrS
P42574	CASP3	Caspase-3	CrS
Q96EA4	SPDL1	Protein Spindly	WT, CrS
Q9UKG1	APPL1	DCC-interacting protein 13-alpha	CrS
F5GZS6	SLC3A2	4F2 cell-surface antigen heavy chain	CrS
P00492	HPRT1	Hypoxanthine-guanine phosphoribosyltransferase	CrS
P06132	UROD	Uroporphyrinogen decarboxylase	CrS
Q9BXJ9	NAA15	N-alpha-acetyltransferase 15, NatA auxiliary subunit	CrS
Q9Y265	RUVBL1	RuvB-like 1	CrS
P62333	PSMC6	26S proteasome regulatory subunit 10B	CrS
P12955	PEPD	Xaa-Pro dipeptidase	WT, CrS
P41250	GARS1	Glycine--tRNA ligase	CrS
Q9NUP9	LIN7C	Protein lin-7 homolog C	CrS
A0A087X054	HYOU1	Hypoxia up-regulated protein 1	CrS
Q9Y6A5	TACC3	Transforming acidic coiled-coil-containing protein 3	WT, CrS
Q9HBH5	RDH14	Retinol dehydrogenase 14	CrS
O60547	GMDS	GDP-mannose 4,6 dehydratase	WT, CrS
P35998	PSMC2	26S proteasome regulatory subunit 7	CrS
Q16512	PKN1	Serine/threonine-protein kinase N1	CrS
Q8NFC6	BOD1L1	Biorientation of chromosomes in cell division protein 1-like 1	WT, CrS
O00165	HAX1	HCLS1-associated protein X-1	CrS
A0AVT1	UBA6	Ubiquitin-like modifier-activating enzyme 6	WT, CrS
P61289	PSME3	Proteasome activator complex subunit 3	CrS
Q9Y3D0	CIAO2B	Cytosolic iron-sulfur assembly component 2B	CrS
Q86WA6	BPHL	Valacyclovir hydrolase	CrS
Q9UBS4	DNAJB11	DnaJ homolog subfamily B member 11	CrS
E9PM92	C11orf58	Small acidic protein	CrS
Q9Y3B7	MRPL11	39S ribosomal protein L11, mitochondrial	CrS
Q49A26	GLYR1	Putative oxidoreductase GLYR1	CrS
A0A087X2B5	BSG	Basigin	CrS
K7ELV2	SEH1L	Nucleoporin SEH1	CrS
P07910	HNRNPC	Heterogeneous nuclear ribonucleoproteins C1/C2	CrS
Q14257	RCN2	Reticulocalbin-2	CrS
O00483	NDUFA4	Cytochrome c oxidase subunit NDUFA4	CrS
**PRDX3 cysteine-dependent interactors**			
B7WPG3	HNRNPLL	Heterogeneous nuclear ribonucleoprotein L-like	WT
Q14161	GIT2	ARF GTPase-activating protein GIT2	WT
Q9Y4B6	DCAF1	DDB1- and CUL4-associated factor 1	WT, CrS
Q92804	TAF15	TATA-binding protein-associated factor 2N	WT
Q9UBE0	SAE1	SUMO-activating enzyme subunit 1	WT
F8W038	C17orf49	Chromosome 17 open reading frame 49	WT
Q6PKG0	LARP1	La-related protein 1	WT
Q15084	PDIA6	Protein disulfide-isomerase A6	WT, CrS
Q92785	DPF2	Zinc finger protein ubi-d4	WT, CrS
P51148	RAB5C	Ras-related protein Rab-5C	WT, CrS
Q9ULX3	NOB1	RNA-binding protein NOB1	WT, CrS
P04843	RPN1	Dolichyl-diphosphooligosaccharide--protein glycosyltransferase subunit 1	WT
Q86WA6	BPHL	Valacyclovir hydrolase	WT, CrS
A0A0A0MR02	VDAC2	Outer mitochondrial membrane protein porin 2	WT, CrS
O14828	SCAMP3	Secretory carrier-associated membrane protein 3	WT
Q5JRI1	SRSF10	Serine/arginine-rich-splicing factor 10	WT
G3V279	ERH	Enhancer of rudimentary homolog	WT
P10599	TXN	Thioredoxin	WT, CrS
Q8N5K1	CISD2	CDGSH iron-sulfur domain-containing protein 2	WT, CrS
A0A0A0MR09	PTPN9	Tyrosine-protein phosphatase non-receptor type 9	WT
Q9H6R0	DHX33	ATP-dependent RNA helicase DHX33	WT, CrS
Q15291	RBBP5	Retinoblastoma-binding protein 5	WT, CrS
H7BXI1	ESYT2	Extended synaptotagmin-2	WT
H3BRY6	INTS14	Integrator complex subunit 14	WT
Q5TDH0	DDI2	Protein DDI1 homolog 2	WT
P08240	SRPRA	Signal recognition particle receptor subunit alpha	WT
P27824	CANX	Calnexin	WT
P22695	UQCRC2	Cytochrome b-c1 complex subunit 2, mitochondrial	WT
Q16718	NDUFA5	NADH dehydrogenase [ubiquinone] 1 alpha subcomplex subunit 5	WT, CrS
Q14697	GANAB	Neutral alpha-glucosidase AB	WT
O75146	HIP1R	Huntingtin-interacting protein 1-related protein	WT, CrS
Q5VW32	BROX	BRO1 domain-containing protein BROX	WT, CrS
P05386	RPLP1	60S acidic ribosomal protein P1	WT, CrS
A6NDU8	C5orf51	UPF0600 protein C5orf51	WT, CrS
Q6DKI1	RPL7L1	60S ribosomal protein L7-like 1	WT, CrS
P30041	PRDX6	Peroxiredoxin-6	WT, CrS
Q9UKK9	NUDT5	ADP-sugar pyrophosphatase	WT, CrS
Q2M2I8	AAK1	AP2-associated protein kinase 1	WT, CrS
P57076	CFAP298	Cilia- and flagella-associated protein 298	WT, CrS
Q66PJ3	ARL6IP4	ADP-ribosylation factor-like protein 6-interacting protein 4	WT, CrS
Q15814	TBCC	Tubulin-specific chaperone C	WT, CrS
Q9NYK5	MRPL39	39S ribosomal protein L39, mitochondrial	WT, CrS
P78346	RPP30	Ribonuclease P protein subunit p30	WT, CrS
P06132	UROD	Uroporphyrinogen decarboxylase	WT, CrS
P62081	RPS7	40S ribosomal protein S7	WT, CrS
Q9UJS0	SLC25A13	Calcium-binding mitochondrial carrier protein Aralar2	WT
K7EMF1	EIF3K	Eukaryotic translation initiation factor 3 subunit K	WT
Q9H0E2	TOLLIP	Toll-interacting protein	WT
Q5SY16	NOL9	Polynucleotide 5'-hydroxyl-kinase NOL9	WT, CrS
O15269	SPTLC1	Serine palmitoyltransferase 1	WT, CrS
Q9Y5Y2	NUBP2	Cytosolic Fe-S cluster assembly factor NUBP2	WT, CrS
Q8IVS2	MCAT	Malonyl-CoA-acyl carrier protein transacylase, mitochondrial	WT, CrS
P50454	SERPINH1	Serpin H1	WT, CrS
Q9NP97	DYNLRB1	Dynein light chain roadblock-type 1	WT, CrS
Q9NS69	TOMM22	Mitochondrial import receptor subunit TOM22 homolog	WT, CrS
Q6UW78	UQCC3	Ubiquinol-cytochrome-c reductase complex assembly factor 3	CrS
A0A2R8YFH5	SEC23B	Protein transport protein SEC23	WT
Q9UHD2	TBK1	Serine/threonine-protein kinase TBK1	WT, CrS
P31040	SDHA	Succinate dehydrogenase [ubiquinone] flavoprotein subunit, mitochondrial	WT, CrS
Q8NBU5	ATAD1	ATPase family AAA domain-containing protein 1	WT, CrS
Q96AT9	RPE	Ribulose-phosphate 3-epimerase	WT, CrS
P35268	RPL22	60S ribosomal protein L22	WT, CrS
P19022	CDH2	Cadherin-2	WT, CrS
F8VYE8	PPP1CC	Serine/threonine-protein phosphatase	WT, CrS
Q6IBS0	TWF2	Twinfilin-2	WT, CrS
O60784	TOM1	Target of Myb protein 1	WT, CrS
P62873	GNB1	Guanine nucleotide-binding protein G(I)/G(S)/G(T) subunit beta-1	WT, CrS
Q13509	TUBB3	Tubulin beta-3 chain	WT, CrS
Q96EA4	SPDL1	Protein Spindly	WT, CrS
A0A0A0MTB8	WDR36	WD repeat-containing protein 36	WT, CrS
Q92990	GLMN	Glomulin	WT, CrS
Q9UL15	BAG5	BAG family molecular chaperone regulator 5	WT, CrS
O43815	STRN	Striatin	WT, CrS
Q9H0U4	RAB1B	Ras-related protein Rab-1B	WT, CrS
Q9UQR0	SCML2	Sex comb on midleg-like protein 2	WT, CrS
F5H008	VPS33B	Vacuolar protein sorting-associated protein 33B	WT, CrS
Q9BSJ8	ESYT1	Extended synaptotagmin-1	WT, CrS
P14174	MIF	Macrophage migration inhibitory factor	WT, CrS
P51809	VAMP7	Vesicle-associated membrane protein 7	WT, CrS
Q9NZ45	CISD1	CDGSH iron-sulfur domain-containing protein 1	WT, CrS
Q9BZX2	UCK2	Uridine-cytidine kinase 2	WT, CrS
Q8TDH9	BLOC1S5	Biogenesis of lysosome-related organelles complex 1 subunit 5	WT, CrS
Q9BQC3	DPH2	2-(3-amino-3-carboxypropyl)histidine synthase subunit 2	WT, CrS
O75663	TIPRL	TIP41-like protein	CrS
Q9GZS3	WDR61	WD repeat-containing protein 61	WT, CrS
O75688	PPM1B	Protein phosphatase 1B	CrS
P35237	SERPINB6	Serpin B6	WT, CrS
P62979	RPS27A	Ubiquitin-40S ribosomal protein S27a	WT, CrS
Q9NP61	ARFGAP3	ADP-ribosylation factor GTPase-activating protein 3	WT, CrS
H7C155	RAF1	Non-specific serine/threonine protein kinase	CrS
P54646	PRKAA2	5'-AMP-activated protein kinase catalytic subunit alpha-2	WT, CrS
P26368	U2AF2	Splicing factor U2AF 65 kDa subunit	CrS
Q9HB71	CACYBP	Calcyclin-binding protein	WT, CrS
A0A087WU06	TUBGCP3	Gamma-tubulin complex component	WT, CrS
Q8N6M0	OTUD6B	Deubiquitinase OTUD6B	WT, CrS
P62820	RAB1A	Ras-related protein Rab-1A	CrS
B4DJ81	NDUFS1	NADH-ubiquinone oxidoreductase 75 kDa subunit, mitochondrial	WT, CrS
Q8N6R0	EEF1AKNMT	eEF1A lysine and N-terminal methyltransferase	WT, CrS
P11802	CDK4	Cyclin-dependent kinase 4	WT, CrS
Q9H974	QTRT2	Queuine tRNA-ribosyltransferase accessory subunit 2	WT, CrS
P21964	COMT	Catechol O-methyltransferase	WT, CrS
Q9P287	BCCIP	BRCA2 and CDKN1A-interacting protein	WT, CrS
Q9UGV2	NDRG3	Protein NDRG3	CrS
P62879	GNB2	Guanine nucleotide-binding protein G(I)/G(S)/G(T) subunit beta-2	WT, CrS
H3BV80	RNPS1	RNA-binding protein with serine-rich domain 1	WT, CrS
Q8IYS1	PM20D2	Peptidase M20 domain-containing protein 2	WT, CrS
Q9HCN8	SDF2L1	Stromal cell-derived factor 2-like protein 1	WT, CrS
X6RM00	ERC1	ELKS/Rab6-interacting/CAST family member 1	WT, CrS
Q15738	NSDHL	Sterol-4-alpha-carboxylate 3-dehydrogenase, decarboxylating	CrS
Q9Y679	AUP1	Lipid droplet-regulating VLDL assembly factor AUP1	WT, CrS
P22061	PCMT1	Protein-L-isoaspartate(D-aspartate) O-methyltransferase	WT, CrS
Q7L2H7	EIF3M	Eukaryotic translation initiation factor 3 subunit M	WT, CrS
P18669	PGAM1	Phosphoglycerate mutase 1	WT, CrS
Q96S59	RANBP9	Ran-binding protein 9	CrS
O95295	SNAPIN	SNARE-associated protein Snapin	WT, CrS
Q15019	SEPTIN2	Septin-2	CrS
Q96BW9	TAMM41	Phosphatidate cytidylyltransferase, mitochondrial	WT, CrS
Q9BTE1	DCTN5	Dynactin subunit 5	WT, CrS
P18031	PTPN1	Tyrosine-protein phosphatase non-receptor type 1	WT, CrS
Q147X3	NAA30	N-alpha-acetyltransferase 30	CrS
Q9NQT5	EXOSC3	Exosome complex component RRP40	CrS
M0R026	ILVBL	2-hydroxyacyl-CoA lyase 2	WT, CrS
Q96FW1	OTUB1	Ubiquitin thioesterase OTUB1	CrS
Q9Y3D0	CIAO2B	Cytosolic iron-sulfur assembly component 2B	WT, CrS
P29558	RBMS1	RNA-binding motif, single-stranded-interacting protein 1	CrS
O95551	TDP2	Tyrosyl-DNA phosphodiesterase 2	CrS
Q5QPM7	PSMF1	Proteasome inhibitor PI31 subunit	WT, CrS
Q5VT66	MTARC1	Mitochondrial amidoxime-reducing component 1	CrS
P30740	SERPINB1	Leukocyte elastase inhibitor	WT, CrS
Q9H9P8	L2HGDH	L-2-hydroxyglutarate dehydrogenase, mitochondrial	WT, CrS
O43237	DYNC1LI2	Cytoplasmic dynein 1 light intermediate chain 2	WT, CrS
Q96IU4	ABHD14B	Protein ABHD14B	WT, CrS
H3BTA2	PPP4C	Serine/threonine-protein phosphatase	WT, CrS
P19367	HK1	Hexokinase-1	CrS
Q53EL6	PDCD4	Programmed cell death protein 4	CrS
Q07820	MCL1	Induced myeloid leukemia cell differentiation protein Mcl-1	WT, CrS
Q8N6T3	ARFGAP1	ADP-ribosylation factor GTPase-activating protein 1	WT, CrS
O43809	NUDT21	Cleavage and polyadenylation specificity factor subunit 5	WT, CrS
P17252	PRKCA	Protein kinase C alpha type	CrS
A0A0A0MS29	MFF	Mitochondrial fission factor	WT
Q16698	DECR1	2,4-dienoyl-CoA reductase, mitochondrial	WT, CrS
Q14318	FKBP8	Peptidyl-prolyl cis-trans isomerase FKBP8	WT, CrS
P16435	POR	NADPH--cytochrome P450 reductase	CrS
G5EA06	MRPS27	28S ribosomal protein S27, mitochondrial	WT, CrS
P35520	CBS	Cystathionine beta-synthase	CrS
P52788	SMS	Spermine synthase	CrS
P07355	ANXA2	Annexin A2	WT, CrS
Q14126	DSG2	Desmoglein-2	CrS
O60566	BUB1B	Mitotic checkpoint serine/threonine-protein kinase BUB1 beta	CrS
Q16204	CCDC6	Coiled-coil domain-containing protein 6	CrS
P11766	ADH5	Alcohol dehydrogenase class-3	WT, CrS
Q9Y570	PPME1	Protein phosphatase methylesterase 1	CrS
P62851	RPS25	40S ribosomal protein S25	CrS
P61106	RAB14	Ras-related protein Rab-14	WT, CrS
Q8WVC2	RPS21	40S ribosomal protein S21	WT, CrS
P45954	ACADSB	Short/branched chain specific acyl-CoA dehydrogenase, mitochondrial	CrS
P34896	SHMT1	Serine hydroxymethyltransferase, cytosolic	CrS
Q16543	CDC37	Hsp90 co-chaperone Cdc37	CrS
Q07021	C1QBP	Complement component 1 Q subcomponent-binding protein, mitochondrial	CrS
P55072	VCP	Transitional endoplasmic reticulum ATPase	CrS
D6RA00	ENOPH1	Enolase-phosphatase E1	WT, CrS
Q9UL25	RAB21	Ras-related protein Rab-21	CrS
Q7L5D6	GET4	Golgi to ER traffic protein 4 homolog	CrS
Q5T749	KPRP	Keratinocyte proline-rich protein	WT, CrS
Q9NQX3	GPHN	Gephyrin [Includes: Molybdopterin adenylyltransferase	CrS
Q96TC7	RMDN3	Regulator of microtubule dynamics protein 3	CrS
P09211	GSTP1	Glutathione S-transferase P	CrS
O14744	PRMT5	Protein arginine N-methyltransferase 5	CrS
Q16576	RBBP7	Histone-binding protein RBBP7	CrS
Q8IUF8	RIOX2	Ribosomal oxygenase 2	CrS
P60953	CDC42	Cell division control protein 42 homolog	CrS
Q8TEX9	IPO4	Importin-4	CrS
Q8TC07	TBC1D15	TBC1 domain family member 15	CrS
Q9Y266	NUDC	Nuclear migration protein nudC	CrS
Q9UI30	TRMT112	Multifunctional methyltransferase subunit TRM112-like protein	CrS
Q96A49	SYAP1	Synapse-associated protein 1	WT, CrS
P21796	VDAC1	Voltage-dependent anion-selective channel protein 1	CrS
P56182	RRP1	Ribosomal RNA processing protein 1 homolog A	CrS
Q96S44	TP53RK	EKC/KEOPS complex subunit TP53RK	WT, CrS
O95831	AIFM1	Apoptosis-inducing factor 1, mitochondrial	CrS
Q16186	ADRM1	Proteasomal ubiquitin receptor ADRM1	CrS
Q96KB5	PBK	Lymphokine-activated killer T-cell-originated protein kinase	CrS
Q9H840	GEMIN7	Gem-associated protein 7	CrS
Q9HD26	GOPC	Golgi-associated PDZ and coiled-coil motif-containing protein	WT, CrS
E9PJN0	ACOT8	Acyl-coenzyme A thioesterase 8	WT, CrS
Q15388	TOMM20	Mitochondrial import receptor subunit TOM20 homolog	CrS
Q8NDI1	EHBP1	EH domain-binding protein 1	CrS
Q9NWV4	CZIB	CXXC motif containing zinc binding protein	WT, CrS
P02790	HPX	Hemopexin	CrS
Q5T760	SRSF11	Serine/arginine-rich-splicing factor 11	CrS
Q9Y6Y8	SEC23IP	SEC23-interacting protein	CrS
O60825	PFKFB2	6-phosphofructo-2-kinase/fructose-2,6-bisphosphatase 2	CrS
P00492	HPRT1	Hypoxanthine-guanine phosphoribosyltransferase	WT, CrS
P51553	IDH3G	Isocitrate dehydrogenase [NAD] subunit gamma, mitochondrial	CrS
Q8IXI1	RHOT2	Mitochondrial Rho GTPase 2	CrS
O75600	GCAT	2-amino-3-ketobutyrate coenzyme A ligase, mitochondrial	WT, CrS
C9JAW5	C9JAW5	HIG1 domain-containing protein	WT, CrS
P40616	ARL1	ADP-ribosylation factor-like protein 1	WT, CrS
Q7Z434	MAVS	Mitochondrial antiviral-signaling protein	WT, CrS
P56385	ATP5ME	ATP synthase subunit e, mitochondrial	CrS
Q92667	AKAP1	A-kinase anchor protein 1, mitochondrial	WT, CrS
Q9UH62	ARMCX3	Armadillo repeat-containing X-linked protein 3	CrS
Q9BYN0	SRXN1	Sulfiredoxin-1	WT, CrS
Q5SZR4	TDRKH	Tudor and KH domain containing, isoform CRA_a	WT, CrS
B3KUS5	USP30	Ubiquitin carboxyl-terminal hydrolase	WT, CrS
Q14498	RBM39	RNA-binding protein 39	CrS
Q5VUJ6	LRCH2	Leucine-rich repeat and calponin homology domain-containing protein 2	CrS
Q9NQG6	MIEF1	Mitochondrial dynamics protein MID51	CrS
O60547	GMDS	GDP-mannose 4,6 dehydratase	CrS
O95801	TTC4	Tetratricopeptide repeat protein 4	CrS
Q9BQA1	WDR77	Methylosome protein 50	CrS
Q14964	RAB39A	Ras-related protein Rab-39A	CrS
P49903	SEPHS1	Selenide, water dikinase 1	CrS
Q13257	MAD2L1	Mitotic spindle assembly checkpoint protein MAD2A	CrS
Q9BS26	ERP44	Endoplasmic reticulum resident protein 44	CrS
Q9BPZ2	SPIN2B	Spindlin-2B	CrS
P20042	EIF2S2	Eukaryotic translation initiation factor 2 subunit 2	CrS
B4E1N1	ARMC6	Armadillo repeat-containing protein 6	CrS
P63151	PPP2R2A	Serine/threonine-protein phosphatase 2A 55 kDa regulatory subunit B alpha isoform	CrS
K7EJQ8	HDHD2	Haloacid dehalogenase-like hydrolase domain-containing protein 2	CrS
P47755	CAPZA2	F-actin-capping protein subunit alpha-2	CrS
A0A0B4J1Z1	SRSF7	Serine/arginine-rich-splicing factor 7	CrS
**PRDX4 cysteine-dependent interactors**			
Q96AC1	FERMT2	Fermitin family homolog 2	WT
Q9BS26	ERP44	Endoplasmic reticulum resident protein 44	WT
Q96DH6	MSI2	RNA-binding protein Musashi homolog 2	WT
Q9NW82	WDR70	WD repeat-containing protein 70	WT
P09110	ACAA1	3-ketoacyl-CoA thiolase, peroxisomal	WT
P22307	SCP2	Non-specific lipid-transfer protein	WT
F5GZS6	SLC3A2	4F2 cell-surface antigen heavy chain	WT
Q8NBS9	TXNDC5	Thioredoxin domain-containing protein 5	WT, CrS
P60174	TPI1	Triosephosphate isomerase	WT
P30041	PRDX6	Peroxiredoxin-6	WT
Q9BTZ2	DHRS4	Dehydrogenase/reductase SDR family member 4	WT
Q9Y2R5	MRPS17	28S ribosomal protein S17, mitochondrial	WT, CrS
Q96K76	USP47	Ubiquitin carboxyl-terminal hydrolase 47	WT
Q15084	PDIA6	Protein disulfide-isomerase A6	WT
Q13257	MAD2L1	Mitotic spindle assembly checkpoint protein MAD2A	WT
Q14739	LBR	Delta(14)-sterol reductase LBR	WT
Q86UP2	KTN1	Kinectin	WT
E9PGZ1	CALD1	Caldesmon	WT
Q9UHV9	PFDN2	Prefoldin subunit 2	WT
C9J9K3	RPSA	40S ribosomal protein SA	WT
P26599	PTBP1	Polypyrimidine tract-binding protein 1	WT
Q96I99	SUCLG2	Succinate--CoA ligase [GDP-forming] subunit beta, mitochondrial	WT
Q66K74	MAP1S	Microtubule-associated protein 1S	WT, CrS
P34897	SHMT2	Serine hydroxymethyltransferase, mitochondrial	WT
P10599	TXN	Thioredoxin	WT
P22234	PAICS	Multifunctional protein ADE2 [Includes: Phosphoribosylaminoimidazole-succinocarboxamide synthase	WT, CrS
P62249	RPS16	40S ribosomal protein S16	WT, CrS
I3L1P8	SLC25A11	Mitochondrial 2-oxoglutarate/malate carrier protein	WT, CrS
Q9Y4B6	DCAF1	DDB1- and CUL4-associated factor 1	WT
P15880	RPS2	40S ribosomal protein S2	WT
O15372	EIF3H	Eukaryotic translation initiation factor 3 subunit H	WT
P34949	MPI	Mannose-6-phosphate isomerase	WT
Q93034	CUL5	Cullin-5	WT
Q92783	STAM	Signal transducing adapter molecule 1	WT, CrS
P05388	RPLP0	60S acidic ribosomal protein P0	WT
P06576	ATP5F1B	ATP synthase subunit beta, mitochondrial	WT
P42345	MTOR	Serine/threonine-protein kinase mTOR	WT
P06733	ENO1	Alpha-enolase	WT
P18077	RPL35A	60S ribosomal protein L35a	WT
Q9HCD5	NCOA5	Nuclear receptor coactivator 5	WT
Q9BSJ8	ESYT1	Extended synaptotagmin-1	WT, CrS
P30154	PPP2R1B	Serine/threonine-protein phosphatase 2A 65 kDa regulatory subunit A beta isoform	WT, CrS

P30154	PPP2R1B	Serine/threonine-protein phosphatase 2A 65 kDa regulatory subunit A beta isoform	WT, CrS
Q96C36	PYCR2	Pyrroline-5-carboxylate reductase 2	WT
B4DJ81	NDUFS1	NADH-ubiquinone oxidoreductase 75 kDa subunit, mitochondrial	WT, CrS
D6RA00	ENOPH1	Enolase-phosphatase E1	WT, CrS
O43809	NUDT21	Cleavage and polyadenylation specificity factor subunit 5	WT, CrS
Q13347	EIF3I	Eukaryotic translation initiation factor 3 subunit I	WT
P49411	TUFM	Elongation factor Tu, mitochondrial	WT, CrS
Q5SW79	CEP170	Centrosomal protein of 170 kDa	WT, CrS
Q9P2J5	LARS1	Leucine--tRNA ligase, cytoplasmic	WT
P62906	RPL10A	60S ribosomal protein L10a	WT
Q9UQR0	SCML2	Sex comb on midleg-like protein 2	WT, CrS
Q16513	PKN2	Serine/threonine-protein kinase N2	WT, CrS
P13489	RNH1	Ribonuclease inhibitor	WT, CrS
A0A087WY71	AP2M1	AP-2 complex subunit mu	WT, CrS
P18858	LIG1	DNA ligase 1	WT, CrS
O14974	PPP1R12A	Protein phosphatase 1 regulatory subunit 12A	WT, CrS
Q8IUI8	CRLF3	Cytokine receptor-like factor 3	CrS
F5H6E2	MYO1C	Unconventional myosin-Ic	WT, CrS
Q12904	AIMP1	Aminoacyl tRNA synthase complex-interacting multifunctional protein 1	WT, CrS
Q07020	RPL18	60S ribosomal protein L18	CrS
Q8WX93	PALLD	Palladin	CrS
Q14152	EIF3A	Eukaryotic translation initiation factor 3 subunit A	WT, CrS
Q14697	GANAB	Neutral alpha-glucosidase AB	WT, CrS
Q14318	FKBP8	Peptidyl-prolyl cis-trans isomerase FKBP8	CrS
Q9BWF3	RBM4	RNA-binding protein 4	CrS
A0A2R8Y7S2	SMARCA4	Transcription activator BRG1	WT, CrS
O75607	NPM3	Nucleoplasmin-3	CrS
O75592	MYCBP2	E3 ubiquitin-protein ligase MYCBP2	CrS
Q09666	AHNAK	Neuroblast differentiation-associated protein AHNAK	CrS
Q12905	ILF2	Interleukin enhancer-binding factor 2	CrS
P30520	ADSS2	Adenylosuccinate synthetase isozyme 2	CrS
Q9C0C2	TNKS1BP1	182 kDa tankyrase-1-binding protein	CrS
P84103	SRSF3	Serine/arginine-rich splicing factor 3	CrS
Q8TCG1	CIP2A	Protein CIP2A	CrS
Q9BVP2	GNL3	Guanine nucleotide-binding protein-like 3	CrS
A0A0B4J1Z1	SRSF7	Serine/arginine-rich-splicing factor 7	WT, CrS
E7EPN9	PRRC2C	Protein PRRC2C	CrS
Q16543	CDC37	Hsp90 co-chaperone Cdc37	CrS
Q13618	CUL3	Cullin-3	CrS
Q7KZ85	SUPT6H	Transcription elongation factor SPT6	CrS
Q92974	ARHGEF2	Rho guanine nucleotide exchange factor 2	CrS
Q9Y2R4	DDX52	Probable ATP-dependent RNA helicase DDX52	CrS
Q99567	NUP88	Nuclear pore complex protein Nup88	CrS
P49593	PPM1F	Protein phosphatase 1F	CrS
P27824	CANX	Calnexin	CrS
Q9HCN8	SDF2L1	Stromal cell-derived factor 2-like protein 1	CrS
P29372	MPG	DNA-3-methyladenine glycosylase	CrS
A0A087X054	HYOU1	Hypoxia up-regulated protein 1	CrS
P11802	CDK4	Cyclin-dependent kinase 4	CrS
Q12797	ASPH	Aspartyl/asparaginyl beta-hydroxylase	WT, CrS
O00469	PLOD2	Procollagen-lysine,2-oxoglutarate 5-dioxygenase 2	CrS
Q9NVP1	DDX18	ATP-dependent RNA helicase DDX18	CrS
**PRDX5 cysteine-dependent interactors**			
Q15388	TOMM20	Mitochondrial import receptor subunit TOM20 homolog	WT
Q9BS26	ERP44	Endoplasmic reticulum resident protein 44	WT
Q9H2P9	DPH5	Diphthine methyl ester synthase	WT
Q53GQ0	HSD17B12	Very-long-chain 3-oxoacyl-CoA reductase	WT
P63167	DYNLL1	Dynein light chain 1, cytoplasmic	WT
Q5T760	SRSF11	Serine/arginine-rich splicing factor 11	WT
Q16822	PCK2	Phosphoenolpyruvate carboxykinase [GTP], mitochondrial	WT
Q16698	DECR1	2,4-dienoyl-CoA reductase, mitochondrial	WT
E9PGT1	TSN	Component 3 of promoter of RISC	WT
Q92990	GLMN	Glomulin	WT
Q9NT62	ATG3	Ubiquitin-like-conjugating enzyme ATG3	WT
Q99598	TSNAX	Translin-associated protein X	WT
A0A0B4J1Z1	SRSF7	Serine/arginine-rich-splicing factor 7	WT
P22061	PCMT1	Protein-L-isoaspartate(D-aspartate) O-methyltransferase	WT
Q15750	TAB1	TGF-beta-activated kinase 1 and MAP3K7-binding protein 1	WT
Q9Y266	NUDC	Nuclear migration protein nudC	WT
Q9BW92	TARS2	Threonine--tRNA ligase, mitochondrial	WT
P52888	THOP1	Thimet oligopeptidase	WT
Q9H773	DCTPP1	dCTP pyrophosphatase 1	WT
Q9P2N5	RBM27	RNA-binding protein 27	WT
Q6WKZ4	RAB11FIP1	Rab11 family-interacting protein 1	WT
F5GZS6	SLC3A2	4F2 cell-surface antigen heavy chain	WT
E9PJN0	ACOT8	Acyl-coenzyme A thioesterase 8	WT
Q9NQX3	GPHN	Gephyrin [Includes: Molybdopterin adenylyltransferase	WT
Q9GZU8	PSME3IP1	PSME3-interacting protein	WT
A0AVT1	UBA6	Ubiquitin-like modifier-activating enzyme 6	WT
Q12931	TRAP1	Heat shock protein 75 kDa, mitochondrial	WT
B7Z7F3	RANBP3	Ran-binding protein 3	WT
Q9UL15	BAG5	BAG family molecular chaperone regulator 5	WT
Q15061	WDR43	WD repeat-containing protein 43	WT
Q8NBU5	ATAD1	ATPase family AAA domain-containing protein 1	WT
Q13185	CBX3	Chromobox protein homolog 3	WT
P30041	PRDX6	Peroxiredoxin-6	WT
P26599	PTBP1	Polypyrimidine tract-binding protein 1	WT, CrS
P14174	MIF	Macrophage migration inhibitory factor	WT
O14929	HAT1	Histone acetyltransferase type B catalytic subunit	WT
Q9BQA1	WDR77	Methylosome protein 50	WT
Q9Y570	PPME1	Protein phosphatase methylesterase 1	WT
O14579	COPE	Coatomer subunit epsilon	WT
Q5T6F2	UBAP2	Ubiquitin-associated protein 2	WT
Q9C0C2	TNKS1BP1	182 kDa tankyrase-1-binding protein	WT
Q9BSJ8	ESYT1	Extended synaptotagmin-1	WT
P00492	HPRT1	Hypoxanthine-guanine phosphoribosyltransferase	WT
Q9Y6A5	TACC3	Transforming acidic coiled-coil-containing protein 3	WT
Q09666	AHNAK	Neuroblast differentiation-associated protein AHNAK	WT
H7BZM7	ZPR1	Zinc finger protein ZPR1	WT
P12955	PEPD	Xaa-Pro dipeptidase	WT
P22234	PAICS	Multifunctional protein ADE2 [Includes: Phosphoribosylaminoimidazole-succinocarboxamide synthase	WT, CrS
O60664	PLIN3	Perilipin-3	WT
Q9BRS2	RIOK1	Serine/threonine-protein kinase RIO1	WT
P11413	G6PD	Glucose-6-phosphate 1-dehydrogenase	WT
C9J9K3	RPSA	40S ribosomal protein SA	WT
Q14C86	GAPVD1	GTPase-activating protein and VPS9 domain-containing protein 1	WT, CrS
Q96RL1	UIMC1	BRCA1-A complex subunit RAP80	WT
O43815	STRN	Striatin	WT
Q9NQP4	PFDN4	Prefoldin subunit 4	WT
P15880	RPS2	40S ribosomal protein S2	WT
Q9HB71	CACYBP	Calcyclin-binding protein	WT, CrS
P40222	TXLNA	Alpha-taxilin	WT, CrS
Q15276	RABEP1	Rab GTPase-binding effector protein 1	WT
Q9UNM6	PSMD13	26S proteasome non-ATPase regulatory subunit 13	WT
Q66PJ3	ARL6IP4	ADP-ribosylation factor-like protein 6-interacting protein 4	WT
Q9H0E2	TOLLIP	Toll-interacting protein	WT
Q8NFC6	BOD1L1	Biorientation of chromosomes in cell division protein 1-like 1	WT, CrS
Q04446	GBE1	1,4-alpha-glucan-branching enzyme	WT
Q8N3X1	FNBP4	Formin-binding protein 4	WT, CrS
Q05655	PRKCD	Protein kinase C delta type	WT
P48047	ATP5PO	ATP synthase subunit O, mitochondrial	WT, CrS
Q5JRA6	MIA3	Transport and Golgi organization protein 1 homolog	WT
P43304	GPD2	Glycerol-3-phosphate dehydrogenase, mitochondrial	WT, CrS
Q9Y3F4	STRAP	Serine-threonine kinase receptor-associated protein	WT
Q16531	DDB1	DNA damage-binding protein 1	WT
H7C128	BRD8	Bromodomain-containing protein 8	WT, CrS
P05386	RPLP1	60S acidic ribosomal protein P1	WT
Q96A49	SYAP1	Synapse-associated protein 1	WT, CrS
P51665	PSMD7	26S proteasome non-ATPase regulatory subunit 7	WT
Q32MZ4	LRRFIP1	Leucine-rich repeat flightless-interacting protein 1	WT
Q8NC51	SERBP1	Plasminogen activator inhibitor 1 RNA-binding protein	WT, CrS
A0A0A0MR02	VDAC2	Outer mitochondrial membrane protein porin 2	WT, CrS
Q8N6M0	OTUD6B	Deubiquitinase OTUD6B	WT
Q9UI12	ATP6V1H	V-type proton ATPase subunit H	WT
O95801	TTC4	Tetratricopeptide repeat protein 4	WT, CrS
Q9BSD7	NTPCR	Cancer-related nucleoside-triphosphatase	WT
Q9BTY7	HGH1	Protein HGH1 homolog	WT
E7EVA0	MAP4	Microtubule-associated protein	WT
Q9Y277	VDAC3	Voltage-dependent anion-selective channel protein 3	WT
A0A087WUT6	EIF5B	Eukaryotic translation initiation factor 5B	WT
E7EV99	ADD1	Alpha-adducin	WT
E9PLA9	CAPRIN1	Caprin-1	WT
Q9H2U1	DHX36	ATP-dependent DNA/RNA helicase DHX36	WT
Q9BQ69	MACROD1	ADP-ribose glycohydrolase MACROD1	WT
P22570	FDXR	NADPH:adrenodoxin oxidoreductase, mitochondrial	WT
P43686	PSMC4	26S proteasome regulatory subunit 6B	WT, CrS
Q02952	AKAP12	A-kinase anchor protein 12	WT
P62195	PSMC5	26S proteasome regulatory subunit 8	WT
P31689	DNAJA1	DnaJ homolog subfamily A member 1	WT, CrS
Q2TAM5	RELA	RELA protein	WT, CrS
P25398	RPS12	40S ribosomal protein S12	WT
P62333	PSMC6	26S proteasome regulatory subunit 10B	WT
P56385	ATP5ME	ATP synthase subunit e, mitochondrial	WT
A0A0C4DGQ6	RPRD1A	Regulation of nuclear pre-mRNA domain-containing protein 1A	WT, CrS
P04843	RPN1	Dolichyl-diphosphooligosaccharide--protein glycosyltransferase subunit 1	WT
Q10713	PMPCA	Mitochondrial-processing peptidase subunit alpha	WT
P46821	MAP1B	Microtubule-associated protein 1B	WT
F8W1A4	AK2	Adenylate kinase 2, mitochondrial	WT
Q6PID6	TTC33	Tetratricopeptide repeat protein 33	WT, CrS
Q15645	TRIP13	Pachytene checkpoint protein 2 homolog	WT
O00483	NDUFA4	Cytochrome c oxidase subunit NDUFA4	WT, CrS
P78371	CCT2	T-complex protein 1 subunit beta	WT, CrS
P07355	ANXA2	Annexin A2	WT, CrS
Q96IZ0	PAWR	PRKC apoptosis WT1 regulator protein	WT, CrS
P62979	RPS27A	Ubiquitin-40S ribosomal protein S27a	WT, CrS
E9PGZ1	CALD1	Caldesmon	WT, CrS
C9J4Z3	RPL37A	60S ribosomal protein L37a	WT, CrS
Q5QPM7	PSMF1	Proteasome inhibitor PI31 subunit	CrS
Q9NZL9	MAT2B	Methionine adenosyltransferase 2 subunit beta	WT, CrS
P85037	FOXK1	Forkhead box protein K1	WT, CrS
Q9NX63	CHCHD3	MICOS complex subunit MIC19	CrS
A0A024R442	A0A024R442	>tr|A0A024R442|A0A024R442_HUMAN Aspartyl aminopeptidase, isoform CRA_b OS=Homo sapiens OX=9606 GN=DNPEP PE=1 SV=1;>sp|Q9ULA0|DNPEP_HUMAN Aspartyl aminopeptidase OS=Homo sapiens OX=9606 GN=DNPEP PE=1 SV=1;>tr|E7ETB3|E7ETB3_HUMAN Aspartyl aminopeptidase, iso	WT, CrS
P13667	PDIA4	Protein disulfide-isomerase A4	CrS
Q96HS1	PGAM5	Serine/threonine-protein phosphatase PGAM5, mitochondrial	CrS
O95831	AIFM1	Apoptosis-inducing factor 1, mitochondrial	CrS
A0A075B6R9	IGKV2D-24	Probable non-functional immunoglobulin kappa variable 2D-24	CrS
Q13257	MAD2L1	Mitotic spindle assembly checkpoint protein MAD2A	CrS
E5KLJ9	OPA1	Dynamin-like 120 kDa protein, form S1	CrS
P30740	SERPINB1	Leukocyte elastase inhibitor	CrS
Q6DKK2	TTC19	Tetratricopeptide repeat protein 19, mitochondrial	WT, CrS
P36551	CPOX	Oxygen-dependent coproporphyrinogen-III oxidase, mitochondrial	WT, CrS
O00165	HAX1	HCLS1-associated protein X-1	WT, CrS
Q96DH6	MSI2	RNA-binding protein Musashi homolog 2	CrS

Table S5. Amino acid motifs in C_P-SOH-mediated interactors

	motif	score	fold increase
1	..W.C..C.	319.4	15.3
2	.C..C..C.	315.7	7.6
3	.C..CG...	315.7	5.2
4	....C..CG	315.3	4.8
5	.H..C..C.	315.2	7.5
6	.C..C...I	311.7	4.5
7	.C..C...N	311.7	4.7
8	...KC..C.	311.6	3.7
9	.C..CQ...	310.5	3.6
10	.CD.C....	17.2	3.2
11	....CD.C.	16.4	3.3
12	L..LC..L.	16.2	2.7
13	.CV.C....	14	2.8
14	.A..C..C.	13	2.9
15	L...C..L.	12.3	1.6
16	F.Q.C....	11.6	2.6
17	.CG.C....	11.3	2.7
18	G...C..C.	10.1	2.8
19	V.K.C....	9.8	1.9
20	L..FC....	9.1	1.9
21	F..YC....	9.1	3
22	..E.C..L.	8.8	1.7
23	.L..C..D.	8.6	1.9
24	.I..C.E..	8.3	1.9
25	L.N.C....	8	1.8
26	V..VC....	7.9	1.9
27	....CI..N	7.9	2.8
28	I...C..Y.	7.6	2.4
29	L.E.C....	7.5	1.6
30	.C..C....	7.4	1.6
31	.G..C..C.	7	2.5
32	.V..C..A.	7	1.6
33	...GC.V..	6.9	1.8
34	.E..CR...	6.8	1.8
35	...LC..L.	6.8	1.6
36	I..FC....	6.7	2.2
37	F..IC....	6.7	2.2
38	.Y..C..L.	6.3	2.3
39	.V..C...P	6.2	1.8
40	.I..C..N.	6	2
41	I..AC....	6	1.9
42	....C.VI.	5.9	2
43	L.A.C....	5.8	1.6
44	..V.CL...	5.8	1.7
45	.V..C...Y	5.7	2.1
46	.LQ.C....	5.7	1.7
47	...HC...A	5.6	2.3
48	...VCR...	5.6	2.1
49	....CN.N.	5.6	2.9
50	....CFQ..	5.5	2.3
51	..A.CY...	5.5	2.4
52	.I..C..G.	5.4	1.8
53	V...C..L.	5.3	1.6
54	..I.CV...	5.3	2
55	...FC..V.	5.2	2
56	....CV..L	5.2	1.8
57	....CS.F.	5.2	2.4
58	F...C..P.	5	2.1
59	.Y..C...L	5	2.1
60	L...C..V.	4.9	1.6
61	...IC.A..	4.9	1.9
62	...DC...I	4.9	2.6
63	.V..C..R.	4.8	1.7
64	V...C...I	4.8	1.8
65	I...C..I.	4.8	2
66	...AC..A.	4.8	2
67	.S.AC....	4.7	1.9
68	...SCS...	4.6	1.9
69	.AL.C....	4.6	2.2
70	...LCG...	4.5	1.5
71	L..LC....	4.5	1.5
72	.L..C...I	4.5	1.7
73	T...CS...	4.5	1.8
74	AA..C....	4.5	1.8
75	.P..C..P.	4.5	2.2
76	...FC.V..	4.4	2.1
77	G.I.C....	4.4	1.8
78	G.Y.C....	4.4	2.4
79	F...C..A.	4.3	1.9
80	....CRV..	4.3	2
81	L...C.I..	4.3	2.2
82	V...C..A.	4.3	2.4
83	L...C...F	4.2	2
84	....CRI..	4.2	2
85	...GCG...	4.2	1.9
86	..V.C.S..	4.2	2.1
87	V.R.C....	4.1	1.7
88	.I..CT...	4.1	1.9
89	..I.C..T.	4.1	2.1
90	..V.C..F.	4	1.9
91	.F..C.D..	4	2
92	.A..CL...	4	1.7
93	....C..TS	4	2.1
94	D...CF...	3.9	1.9
95	..V.CI...	3.9	1.8
96	K...C.L..	3.9	2
97	....C..SS	3.9	2.1
98	V...CI...	3.8	1.8
99	I...C...G	3.8	1.8
100	AI..C....	3.8	2
101	..L.C...V	3.8	1.7
102	.V..C...T	3.7	1.7
103	...ICD...	3.7	1.9
104	T...CL...	3.7	1.6
105	..G.C...D	3.7	1.9
106	.L..CK...	3.7	1.8
107	.L..C...Y	3.6	1.8
108	V...C...S	3.6	1.8
109	....C..C.	3.5	1.4
110	.L..C...L	3.4	1.4
111	F..LC....	3.4	1.8
112	.V.SC....	3.4	1.7
113	.I..C.D..	3.4	1.8
114	...LC..A.	3.3	1.5
115	...SCT...	3.3	1.7
116	L.V.C....	3.2	1.6
117	.L..CI...	3.2	1.7
118	.L..CE...	3	1.7
119	Y...C....	2.9	1.2
120	..G.C.L..	2.9	1.7
121	W...C....	2.5	1.4
122	....C..W.	2.1	1.3
123	H...C....	2.1	1.3
124	....CY...	2	1.2
125	..I.C....	2	1.2
126	....CF...	1.9	1.2
127	F...C....	1.9	1.2
128	.M..C....	1.9	1.3
129	...VC....	1.9	1.2
130	....C...M	1.9	1.4
131	....C...V	1.9	1.3
132	....C.L..	1.9	1.3
133	....C...W	1.8	1.4
134	..V.C....	1.8	1.2
135	.I..C....	1.8	1.3
136	....C..M.	1.7	1.2
137	....C..Y.	1.7	1.2
138	....CW...	1.7	1.3
139	...YC....	1.7	1.2
140	...FC....	1.7	1.2
141	I...C....	1.7	1.2
142	.L..C....	1.7	1.2
143	....CR...	1.7	1.3
144	...SC....	1.7	1.3
145	....CI...	1.7	1.3
146	....C...S	1.7	1.4
147	....C...P	1.7	1.5
148	.P..C....	1.7	1.6
149	....C.W..	1.6	1.4
150	.V..C....	1.6	1.2
151	.F..C....	1.6	1.2
152	....C..H.	1.6	1.3
153	..F.C....	1.6	1.2
154	....C...I	1.6	1.2
155	.Q..C....	1.6	1.2
156	....CS...	1.6	1.2
157	....CL...	1.6	1.2
158	..Q.C....	1.6	1.4
159	P...C....	1.6	1.5
160	....C...T	1.6	1.5
161	....C..F.	1.5	1.2
162	....C.V..	1.5	1.3
163	....C..V.	1.5	1.3
164	....C...A	1.5	1.3
165	...IC....	1.4	1.5
166	....CE...	1.4	1.4
167	....C.A..	1.3	1.3
168	....C.K..	1.3	1.3

Table S6. Amino acid motifs in C_P-S-S-C_R-mediated interactors

	motif	score	fold increase
1	....C..CG	322.2	9.4
2	.C..CG...	322	9.4
3	....CP.C.	313.4	7.1
4	.CP.C....	312.9	6.8
5	VC..C....	311.3	5.2
6	....C..CK	310.6	4.5
7	.C..CK...	18.7	4.6
8	....C..C.	15.2	2.1
9	L..LC....	11.5	1.9
10	.C..C....	9.9	1.9
11	.D..C.V..	9.4	2.3
12	V..LC....	7.9	2.1
13	..I.C..P.	7.7	4
14	..R.C..L.	7.3	2.1
15	..V.C.V..	7.1	2
16	L...C..F.	6.9	2
17	.V..CV...	6.3	2
18	..V.C..G.	6.3	2.1
19	L...C...N	6.1	2
20	..T.C.V..	5.7	2.1
21	..A.C.L..	5.4	2.2
22	...LC..L.	5.3	2.1
23	I...C...G	5.2	1.9
24	...ICD...	5.1	2.4
25	..V.CL...	5.1	1.9
26	....CP.I.	5	2.3
27	FL..C....	4.9	1.9
28	...RC..L.	4.9	1.9
29	.VI.C....	4.8	2.1
30	.L..C..V.	4.7	1.8
31	L..FC....	4.6	1.9
32	I...C..L.	4.5	1.8
33	.T..CL...	4.5	2.1
34	V...C.D..	4.2	2.1
35	.AS.C....	4.1	1.6
36	.V..CL...	4.1	1.8
37	.L..C..D.	4	1.9
38	L...C.V..	3.9	1.7
39	L.D.C....	3.9	1.8
40	....C.V.L	3.7	1.9
41	.L..CV...	3.7	1.9
42	AA..C....	3.6	1.7
43	.A..C.L..	3.6	1.7
44	LA..C....	3.6	2.1
45	....CL..L	3.5	1.7
46	...VC...S	3.4	1.8
47	AL..C....	3.3	1.8
48	.L..C.L..	3.2	1.8
49	...FC....	2.6	1.3
50	....C.T..	2.6	1.4
51	...YC....	2.5	1.3
52	....CL...	2.4	1.3
53	....C.I..	2.3	1.2
54	...IC....	2.2	1.2
55	...HC....	2.2	1.3
56	.V..C....	2.2	1.4
57	I...C....	2.1	1.2
58	.L..C....	2.1	1.3
59	...WC....	2	1.5
60	...VC....	2	1.3
61	....C.F..	1.9	1.3
62	....C.V..	1.9	1.2
63	.H..C....	1.9	1.4
64	.F..C....	1.9	1.3
65	....C.S..	1.9	1.2
66	..E.C....	1.9	1.2
67	V...C....	1.8	1.2
68	..V.C....	1.8	1.2
69	.Y..C....	1.8	1.3
70	....CP...	1.6	1.2
71	....C...D	1.6	1.4
72	G...C....	1.5	1.2
73	....C.A..	1.4	1.2
74	....C.N..	1.4	1.3
